# A bifunctional iminophosphorane squaramide catalyzed enantioselective synthesis of hydroquinazolines *via* intramolecular aza-Michael reaction to α,β-unsaturated esters†

**DOI:** 10.1039/d1sc00856k

**Published:** 2021-03-18

**Authors:** Guanglong Su, Connor J. Thomson, Ken Yamazaki, Daniel Rozsar, Kirsten E. Christensen, Trevor A. Hamlin, Darren J. Dixon

**Affiliations:** Department of Chemistry, Chemistry Research Laboratory, University of Oxford Mansfield Road Oxford OX1 3TA UK darren.dixon@chem.ox.ac.uk; Department of Theoretical Chemistry, Amsterdam Institute of Molecular and Life Sciences (AIMMS), Amsterdam Center for Multiscale Modeling (ACMM), Vrije Universiteit Amsterdam De Boelelaan 1083, 1081 HV Amsterdam The Netherlands t.a.hamlin@vu.nl

## Abstract

An efficient synthesis of enantioenriched hydroquinazoline cores *via* a novel bifunctional iminophosphorane squaramide catalyzed intramolecular aza-Michael reaction of urea-linked α,β-unsaturated esters is described. The methodology exhibits a high degree of functional group tolerance around the forming hydroquinazoline aryl core and wide structural variance on the nucleophilic N atom of the urea moiety. Excellent yields (up to 99%) and high enantioselectivities (up to 97 : 3 er) using both aromatic and less acidic aliphatic ureas were realized. The potential industrial applicability of the transformation was demonstrated in a 20 mmol scale-up experiment using an adjusted catalyst loading of 2 mol%. The origin of enantioselectivity and reactivity enhancement provided by the squaramide motif has been uncovered computationally using density functional theory (DFT) calculations, combined with the activation strain model (ASM) and energy decomposition analysis (EDA).

## Abstract 2

The activation of both aromatic and aliphatic ureas as N-centered nucleophiles in intramolecular Michael addition reactions to α,β-unsaturated esters was achieved under bifunctional iminophosphorane squaramide superbase catalysis.

## Graphical abstract

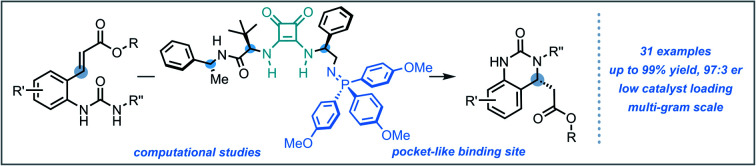

## Introduction

**Fig. 1:**
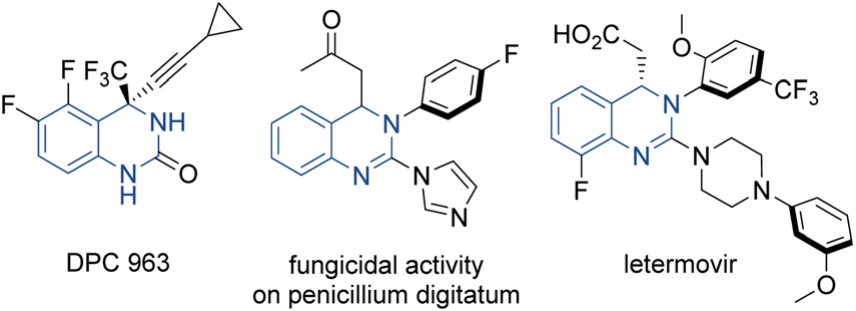
Representative pharmaceutically active compounds containing a hydroquinazoline core.

Heterocyclic organic compounds containing a hydroquinazoline core are commonplace amongst various natural products and potent drug substances used in the clinic.^1^ These include, for instance, DPC 963, a second-generation non-nucleoside reverse transcriptase inhibitor (NNRTI) for HIV treatment,^2^ fungicidal 2-azolyl-3,4-dihydroquinazolines compounds^3^ and the anti-human cytomegalovirus drug, letermovir (Fig. 1).^4^

**Scheme 1:**
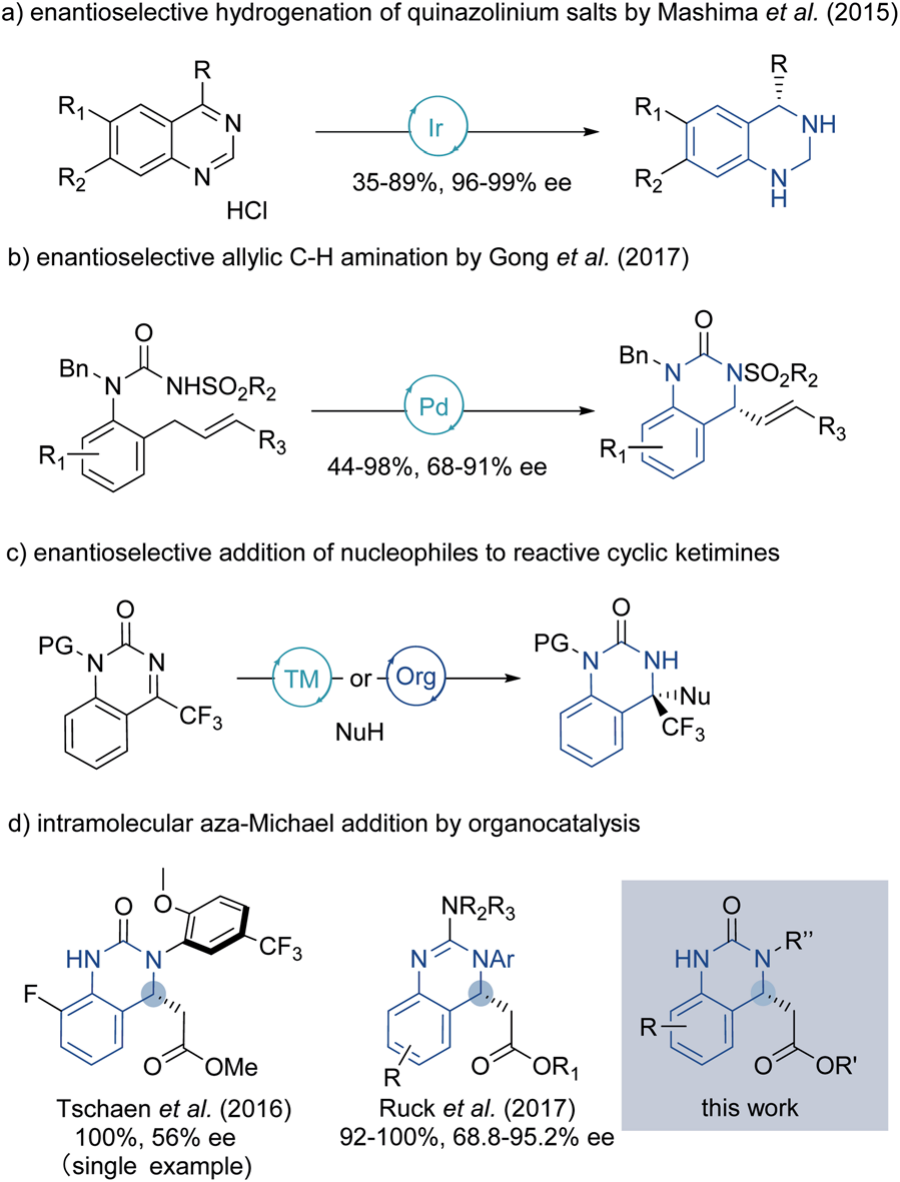
Previous enantioselective syntheses of hydroquinazoline.

Although much effort has been directed towards the synthesis of hydroquinazoline compounds,^5^ highly enantioselective catalytic methods are still relatively uncommon, especially for unbiased/unactivated systems (Scheme 1). In 2015, the Mashima group developed an enantioselective hydrogenation of quinazolinium salts to yield chiral tetrahydroquinazolines with excellent enantioselectivity under chiral iridium catalysis.^6^ A palladium-catalyzed enantioselective allylic C–H amination to generate the chiral hydroquinazoline core in good yield and high enantioselectivity was later described by Gong and coworkers.^7^

Specifically, for dihydroquinazolines bearing a trifluoromethyl group attached to a newly generated quaternary carbon center, an extensive range of metal and metal-free catalyzed enantioselective addition reactions to reactive cyclic ketimines using alkyne,^8^ ketone,^9^ nitroalkane,^10^ β-keto acid,^11^ nitrile,^12^ alcohol^13^ and isocyanoacetate^14^ nucleophiles, have been developed.^15^

Enantioselective aza-Michael reactions enabled by metal-free catalysts are other powerful and promising approaches to access such pharmaceutically relevant *N*-heterocycles.^16^ However, in this field, catalyst promoted addition of pronucleophilic ureas to tethered β-substituted α,β-unsaturated esters remains largely unsolved due to the high p*K*_a_ of the urea and low electrophilicity of the Michael acceptor.^17,18^ To our knowledge only two reports describe the synthesis of the chiral hydroquinazoline core in such a way. In 2016, a single moderately enantioselective phase-transfer-catalyzed intramolecular aza-Michael reaction (IAMR) was described by Tschaen and coworkers en route to letermovir.^19^ In 2017, Ruck and co-workers then developed the enantioselective IAMR reaction of related guanidine containing substrates.^20^ However, only *N*-aryl nucleophiles were compatible and transformation of the guanidine IAMR product to drug molecules bearing urea motifs – such as in DPC 963 (shown in Fig. 1) – was not feasible. Against this backdrop, we envisaged that the enhanced Brønsted basicity and broadly tunable structure of the bifunctional iminophosphorane (BIMP) superbase catalyst system developed in our group^21^ could provide the solution to the challenging p*K*_a_ related reactivity and modest stereocontrol in the IAMR, and herein we wish to report our findings.

## Results and discussion

Urea **1a** bearing an α,β-unsaturated *tert*-butyl ester was chosen as the model substrate for the IAMR reaction. An initial reactivity study of various bifunctional organocatalysts revealed that moderately Brønsted basic cinchona-derived bifunctional catalyst **A** failed to promote any detectable reaction in Et_2_O at room temperature after 24 hours (Table 1, entry 1). In contrast, catalyst **B** bearing a superbasic iminophosphorane motif smoothly gave the desired product **2a** in 96% isolated yield and 68.5 : 31.5 er under identical conditions (Table 1, entry 2). With excellent reactivity identified, a series of modifications to the BIMP catalyst structure was then performed to optimise the IAMR reaction. Changing the H-bond donor from a urea to the more acidic thiourea improved the enantioselectivity to 74 : 26 er but lowered the isolated yield to 73% (Table 1, entry 3). The introduction of a second stereogenic center adjacent to the thiourea motif in the catalyst allowed for rapid library generation and solved the issue of poor reactivity (Table 1, entries 4–6). Variation of the chiral backbone and optimization of reaction conditions revealed that 10 mol% catalyst **F** in 0.025 M toluene at room temperature gave the desired product in almost quantitative yield and 78.5 : 21.5 er (Table 1, entry 7).

**Table 1:** (A) Optimization of reaction conditions. (B) Selected catalysts investigated

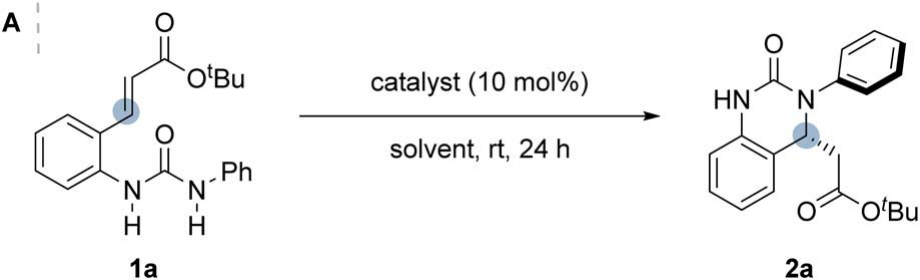					
Entry	Catalyst	Solvent	Concentration (M)	Yield (%)^a^	er^b^
1^c^	A	Et_2_O	0.1	<1	N.D.
2	B	Et_2_O	0.1	96	68.5 : 31.5
3	C	Et_2_O	0.1	73	74 : 26
4	D	Et_2_O	0.1	99	60 : 40
5	E	Et_2_O	0.1	96	74.5 : 25.5
6	F	Et_2_O	0.1	>99	75 : 25
7	F	Toluene	0.025	>99	78.5 : 21.5
8^d^	G	Toluene	0.025	53	67.5 : 32.5
9^d^	H	Toluene	0.025	11	65.5 : 34.5
10^e^	I	Toluene	0.025	76	79 : 21
11^e^	K	Toluene	0.025	71	80.5 : 19.5
**12**	**L**	**Toluene**	**0.025**	**>99**	**94.5 : 5.5**
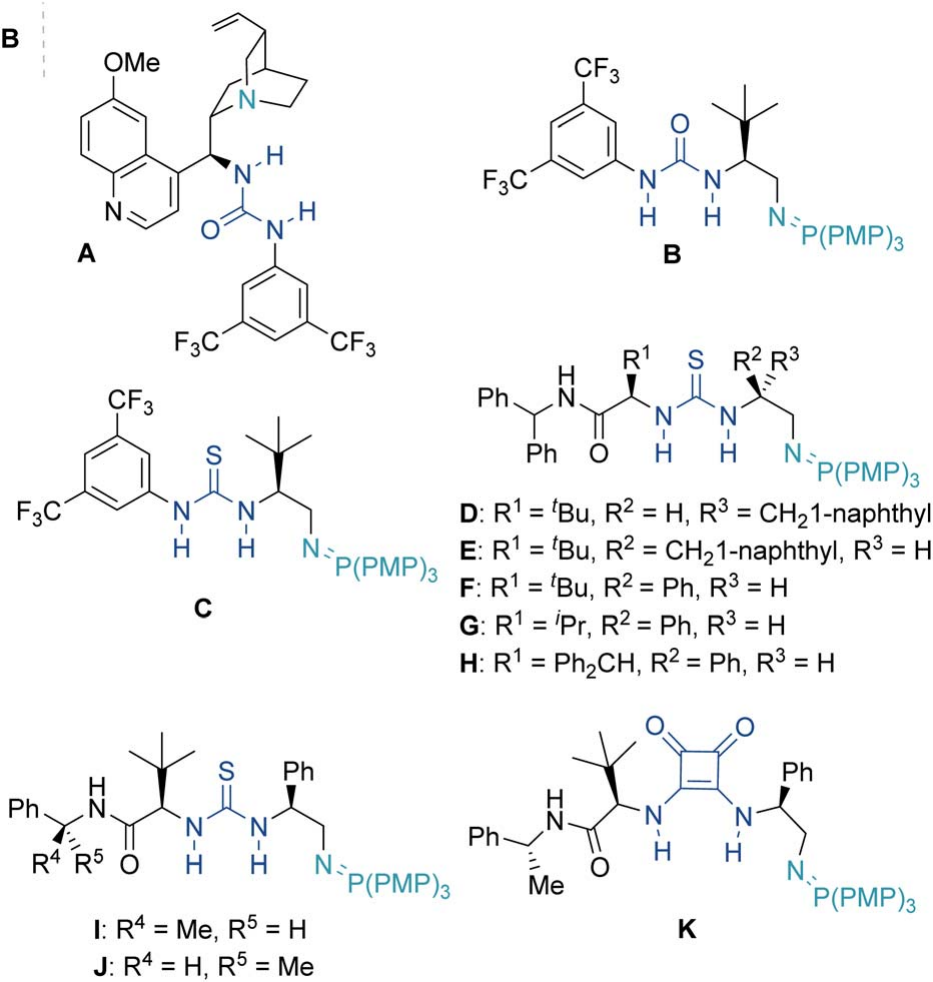					

a Yields of isolated products.

b Determined by HPLC analysis on chiral stationary phase.

c 12 days reaction time.

d 4 hours reaction time.

e 10 hours reaction time. N.D. = not determined.

A third stereogenic center adjacent to the amide motif was then incorporated and enantioselectivity increased to 80.5 : 19.5 er with catalyst **J** slightly outcompeting diastereomeric catalyst **I** (Table 1, entries 10 & 11). Excitingly, a squaramide substitution for the thiourea (catalyst **K**) boosted the enantiocontrol to 94.5 : 5.5 er.

The major enhancement in selectivity likely arises from the higher acidity/H-bond donor ability of the squaramide and/or the modified 3D structure resulting from the differing bond angles at the squaramide.^22^ Additional catalyst structure-performance studies gave no further improvement (see ESI† for optimization details).

**Scheme 2:**
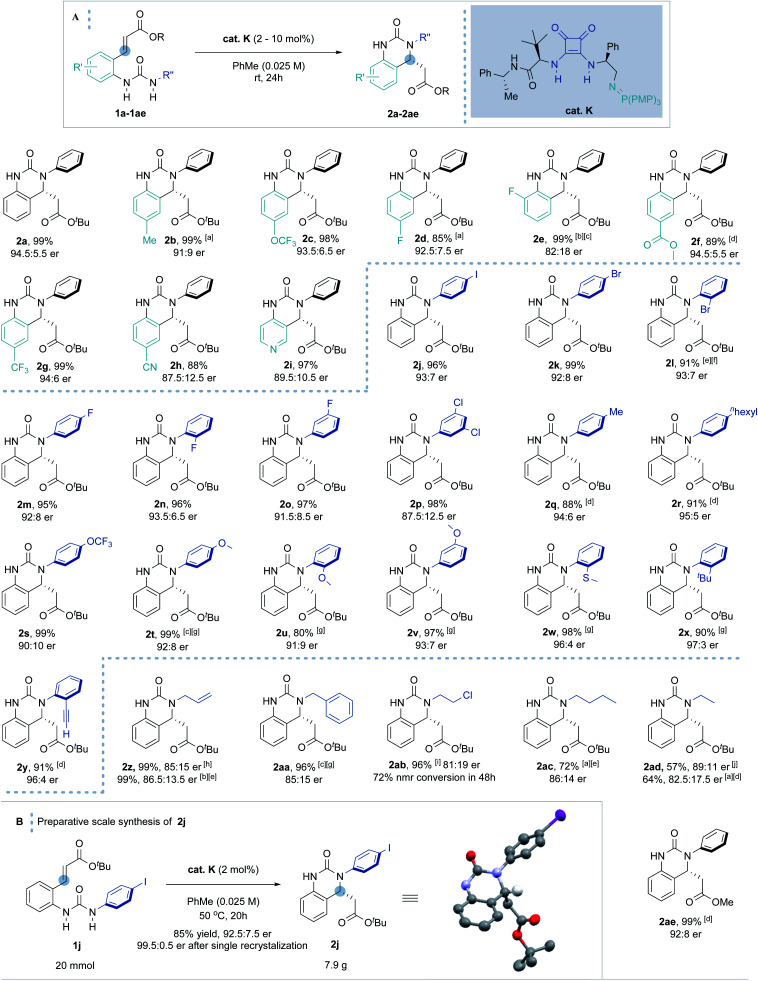
(A) Scope of the BIMP-catalyzed intramolecular aza-Michael reaction to α,β-unsaturated ester. [a] Reaction carried out at 80 °C. [b] Reaction carried out at 40 °C. [c] 30 hours reaction time. [d] 48 hours reaction time. [e] 72 hours reaction time. [f] 5 mol% cat. **K** was used. [g] Reaction carried out at 50 °C. [h] Reaction carried out at 60 °C. [i] 120 hours reaction time. [j] 216 hours reaction time. (B) Preparative scale synthesis of **2j**. Stereochemical configuration was assigned by analogy with (*R*)-**2j** (determined by single crystal X-ray diffraction studies).^33^

With the optimal conditions in hand, the scope of the enantioselective IAMR reaction was then explored (Scheme 2A). Notably, the IAMR reactions were found to typically have very clean reaction profiles and no effort was made to exclude moisture or air from the scoping experiments. Varying the substituents on the quinazolinone aryl core gave rise to minimal fluctuation in enantioselectivity and compatible functionalities varied from electron-donating groups to electron-withdrawing groups. Elevated temperatures of up to 80 °C were required to ensure solubility of the substrates in some cases (**1b** and **1d**). A pyridine-based substrate (**1i**) was also found to be well-tolerated affording the desired product in excellent yield and enantioselectivity under the standard reaction conditions. The substituent effect on the *N*-aryl ring was then examined. Substrates possessing single iodine, bromine and fluorine atoms at various ring positions as well as a 3,5-dichloro example, performed typically well providing the desired hydroquinazoline core in excellent yield and good er (**1j** to **1p**). The rates of the cyclization reactions were found to decrease with increasing electron-richness of the *N*-aryl rings. For substrates **1q** to **1y**, extra reaction time or heating to 50 °C was required to maintain the high yield without compromising enantioselectivity. However, the positional effect of the substituents on reaction enantioselectivity was negligible (**1t** to **1v**). Interestingly, *ortho* substituents (such as thiomethyl, *tert*-butyl and ethynyl in **1w–1y**) gave rise to a slight uplift in enantioselectivity (96 : 4–97 : 3 er). The methodology was also applicable to less activated, higher p*K*_a_, alkyl-substituted ureas. The high Brønsted basicity of the BIMP catalyst system indeed smoothly provided *N*-allyl and *N*-benzyl substituted hydroquinazolines in almost quantitative yield and good er (85 : 15). Even less activated ureas (**1ab** to **1ad**) demanded harsher reaction conditions to deliver the cyclized product in moderate to excellent yield and good er. Finally, the methyl ester acceptor (**1ae**) also proved to be a good substrate. After 48 hours hydroquinazoline product **2ae** was obtained in almost quantitative yield in high enantioselectivity (92 : 8 er). Other conjugate acceptors including phenyl esters, enones and α,β-unsaturated amides were also examined, however satisfactory enantioselectivities were not obtained (see ESI† for details).

**Scheme 3:**
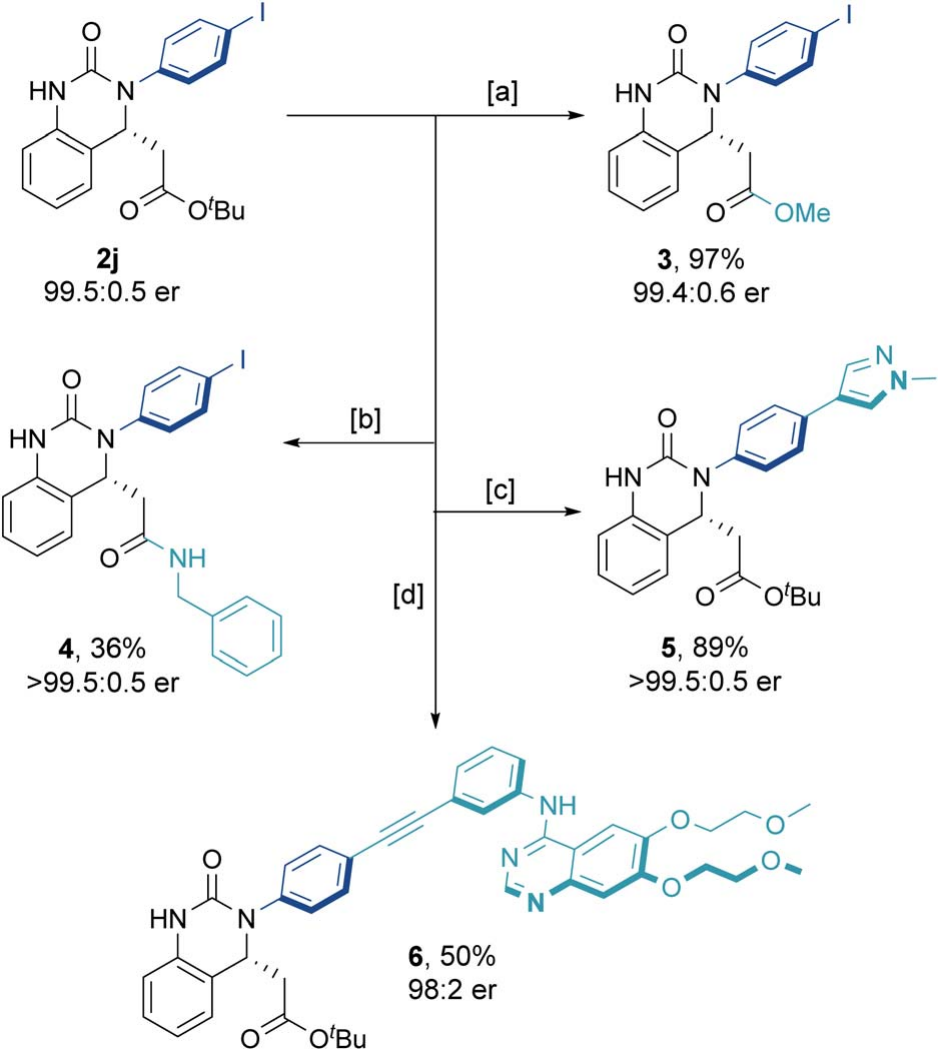
Derivatization of enantioenriched **2j**. [a] (i) TFA, CH_2_Cl_2,_ 0 °C to RT, 5 h. (ii) SOCl_2_, MeOH, 20 h. [b] (i) TFA, CH_2_Cl_2_, 0 °C to RT, 5 h. (ii) (COCl)_2_, DMF (cat.), CH_2_Cl_2_, 2 h. (iii) BnNH_2_, Et_3_N, CH_2_Cl_2_, 18 h. [c] 1-Methyl-1*H*-pyrazole-3-boronic acid pinacol ester, Pd(dppf)Cl_2_CH_2_Cl_2_, Cs_2_CO_3_, 1,4-dioxane, H_2_O. [d] Erlotinib (HCl complex), PdCl_2_(PPh_3_)_2_, CuI, PPh_3_, Et_3_N.

Increasing the reaction scale 100 fold (to 20 mmol) and decreasing the catalyst loading to 2 mol% delivered the desired product in good yield (7.9 g, 85%) without compromising enantioselectivity (92.5 : 7.5 er). Pleasingly, only a single recrystallization was required to afford essentially enantiopure **2j** (Scheme 2B). Furthermore, and to demonstrate potential industrial applicability of the chemistry, various derivatizations of this product were carried out (Scheme 3). For example, removal of the *tert*-butyl carboxylate ester with TFA, activation as the acid chloride, and subsequent treatment with benzyl amine and methanol gave the methyl ester (**3**) and amide (**4**) in excellent to moderate yield, respectively. Suzuki coupling with an *N*-methyl substituted pyrazole boronic acid and Sonogashira coupling with erlotinib successfully installed various functionalities in the *para*-position of the *N*-aryl ring.

In order to paint a mechanistic picture, density functional theory calculations on the aza-Michael reaction step were performed. All calculations reported in this paper were performed using the Amsterdam Density Functional (ADF) software.^23^ Equilibrium structures and transition structure geometries were optimized using the BLYP functional^24,25^ and the DZP basis set.^26^ Solvent effects of toluene were accounted for using the conductor-like screening model (COSMO) of solvation.^27^ Dispersion interactions were included using Grimme's DFT-D3 correction with Becke–Johnson damping.^28^ The zeroth-order regular approximation (ZORA) was used to account for scalar relativistic effects.^29^ This level is referred to as COSMO(toluene)-ZORA-BLYP-D3(BJ)/DZP. All stationary points have been verified, through vibrational analysis, to be minima (zero imaginary frequencies) or transition structures (one imaginary frequency). The character of the normal mode associated with the imaginary frequency has been analyzed to ensure it resembles the reaction under consideration. Optimized structures were illustrated using CYLview20.^30^ Potential energies were refined by means of single point calculations using the M06-2X functional^31^ and the TZ2P basis set.^26^ This level is denoted COSMO(toluene)-ZORA-M06-2X/TZ2P//COSMO(toluene)-ZORA BLYP-D3(BJ)/DZP. The reported Gibbs free energies in solution are calculated by adding thermal corrections computed at 298 K from vibrational frequencies obtained through numerical differentiation of the analytical gradient at COSMO(toluene)-ZORA-BLYP-D3(BJ)/DZP and a standard concentration (1 mol L^−1^) to the total electronic energy at COSMO(toluene)-ZORA-M06-2X/TZ2P//COSMO(toluene)-ZORA-BLYP-D3(BJ)/DZP.

**Scheme 4:**
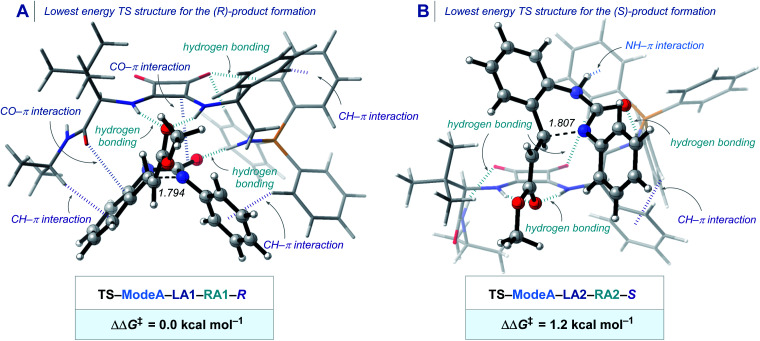
(A) Lowest energy TS structure for the formation of (*R*)-product and (B) lowest energy TS structure for the formation of (*S*)-product of the BIMP squaramide-catalyzed intramolecular aza-Michael reaction computed at COSMO(toluene)-ZORA-M06-2X/TZ2P//COSMO(toluene)-ZORA-BLYP-D3(BJ)/DZP. Energies (kcal mol^−1^) and forming bond lengths (Å) of TS geometries are provided in the insert.

To elucidate the origin of stereocontrol in the novel BIMP squaramide catalyzed IAMR reaction, we performed a state-of-the-art DFT study. Due to the conformational freedom and the existence of two potential activation modes of the BIMP catalyst, we computed and compared all the possible TSs for the enantio-determining Michael reaction step involving substrate **1ae** (see the ESI† for additional details).^32^ The most energetically preferred transition structures that lead to either (*R*)- or (*S*)-product are shown in Scheme 4. The **TS–ModeA–LA1–RA1–R** that forms the (*R*)-product was found to be favoured by 1.2 kcal mol^−1^, which agrees with the experimentally confirmed absolute stereochemical configuration by single crystal X-ray diffraction studies. Pleasingly, our computational approach predicted the enantioselectivity for the formation of product **2ae** in 88 : 12 er, which was in excellent agreement with the experimental selectivity of 92 : 8 er. The energetically preferred TS conformation in **TS–ModeA–LA1–RA1–R** engages in several weak stabilizing interactions. The squaramide moiety interacts with the ester carbonyl group by hydrogen bonding and with the urea carbonyl group by CO–π interaction to activate both the electrophile and the nucleophile. The “*left arm*” of the BIMP catalyst bearing the amide group additionally interacts with the aromatic scaffold in the substrate by both CH–π, and CO–π interactions without significant steric repulsion. The “*right arm*” of the BIMP catalyst bearing the iminophosphorane moiety activates the nucleophilic urea by both hydrogen bonding and through CH–π interactions between the PPh_3_ and the aromatic ring on the N atom in the case of the *N*-aryl substrates. In addition to these catalyst/substrate interactions, the hydrogen bonding and the CH–π interactions within the catalyst also provides the rigidity of this particular lowest energy transition structure.^34^ This conformation creates an ideal-fit pocket within which the substrate can perfectly fit that maximizes stabilizing interactions and minimizes steric repulsion during the C–N bond forming step of the Michael reaction. Analysis of non-covalent interaction (NCI) plots allows one to qualitatively visualize these weak interactions between the catalyst and the substrate (Scheme S5 and S6†).^35^ Therefore, the TS that has a catalyst conformation and coordination mode of the substrate that both reduces steric repulsion and maximizes interactions is energetically preferred in this reaction.

In order to obtain deeper insight into the origin of the catalytic activity imparted by the squaramide motif of the BIMP catalyst, an activation strain analysis (ASA) and an energy decomposition analysis (EDA) were carried out on archetypal model systems. The ASM involves decomposing the electronic energy Δ*E* into the strain energy Δ*E*_strain_ associated with the structural deformation of the hydrogen bond donor (HB) and methyl acrylate (MA) from their equilibrium geometry and the interaction energy Δ*E*_int_ between the deformed reactants [eqn (1)].^36^ The EDA separates the interaction energy (Δ*E*_int_) into the following three chemically meaningful energy terms: classical electrostatic interaction (Δ*V*_elstat_), Pauli repulsion (Δ*E*_Pauli_) between closed-shell orbitals which is responsible for steric repulsion, and stabilizing orbital interaction (Δ*E*_oi_) that accounts, among others, for HOMO–LUMO interactions [eqn (2)].^37^

1 Δ*E* = Δ*E*_strain_ + Δ*E*_int_

2 Δ*E*_int_ = Δ*V*_elstat_ + Δ*E*_Pauli_ + Δ*E*_oi_

**Scheme 5:**
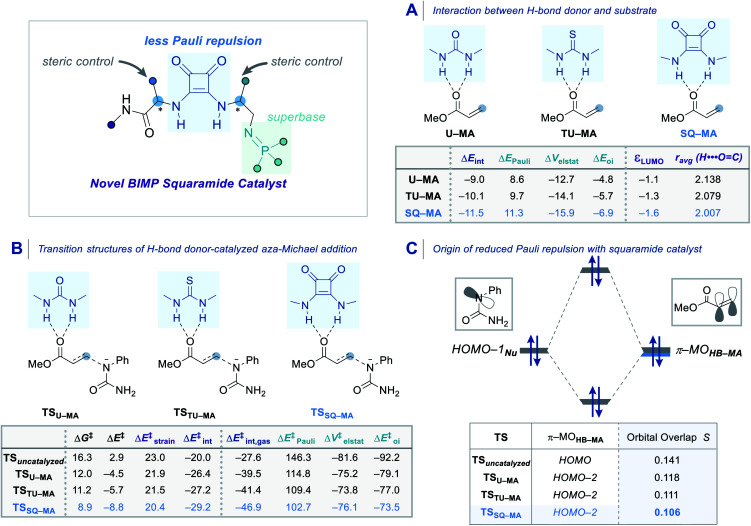
(A) Interaction energies and the energy decomposition analysis (EDA) of the hydrogen bond donor–methyl acrylate complexes (HB–MA). (B) Energy barriers of the aza-Michael reaction transition structures and the activation strain analysis (ASA) and the energy decomposition analysis (EDA). (C) Molecular orbital diagram and the most significant occupied orbital overlaps computed at COSMO(toluene)-ZORA-M06-2X/TZ2P//COSMO(toluene)-ZORA-BLYP-D3(BJ)/DZP. Energies (kcal mol^−1^) are provided in the insert.

First, we analyzed the interaction between HB (urea, thiourea, and squaramide) and MA in the formation of complexes **U–MA**, **TU–MA**, **SQ–MA** (Scheme 5A). The interaction becomes more stabilizing from **U–MA**, **TU–MA**, **SQ–MA** (Δ*E*_int_ = −9.0 to −10.1 to −11.5 kcal mol^−1^) mainly due to the more stabilizing Δ*V*_elstat_ term as a result of the electrostatic nature of hydrogen bonds. The Δ*E*_oi_ term is also very important and involves significant charge transfer from the lone pair of the oxygen atom of MA and the two 
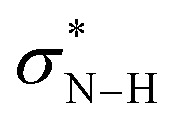
 orbitals on the HB. This flow of charge out of the substrate to the catalyst induces a polarization of the π–MO away from the C=C bond (the importance of which is explained below). The stabilizing Δ*V*_elstat_ and Δ*E*_oi_ interactions play a significant role in the hydrogen bonding stabilization by the squaramide catalyst that can be seen by the decreased H⋯O=C bond length in **SQ–MA** compared to **U–MA** and **TU–MA**.

We then analyzed the transition structures and the energy barriers for the aza-Michael reaction (Scheme 5B). The uncatalyzed reaction goes with the highest reaction barrier (Δ*G*^‡^ = 16.3 kcal mol^−1^). The urea, thiourea, and squaramide catalyzed reactions go with barriers of 12.0, 11.2, and 8.9 kcal mol^−1^, respectively. In order to elucidate the trend in the reactivity, we performed the ASA on the transition structures. The trend in Gibbs free energy activation barriers is the same as for the electronic activation energy barriers. The lower, more favourable, barrier for the squaramide catalyzed reaction compared to the uncatalyzed one (Δ*E*^‡^ = −8.8 *vs.* 2.9 kcal mol^−1^) originates from a more stabilizing interaction energy (Δ*E*^‡^_int_ = −29.2 *vs.* −20.0 kcal mol^−1^). The differences in the Δ*E*^‡^_strain_ also contribute to the trend but are less decisive for the overall reactivity trends (Δ*E*^‡^_strain_ = 20.4 *vs.* 23.0 kcal mol^−1^). Next, using the EDA method, the trend in the more stabilizing Δ*E*^‡^_int_ was analyzed. This successfully identified the role of a reduction in Pauli repulsion between the reactants being the reason for the more enhanced reactivity of the catalyzed reactions.^37^ The origin of the less destabilizing Pauli repulsion (Δ*E*^‡^_Pauli_) for the transformation was quantified by performing a Kohn–Sham molecular orbital (KS–MO) analysis (Scheme 5C). The occupied π–MO**HB–MA** (2_p_ atomic orbitals on the reacting C=C double bonds) contributes to the trend in the Pauli repulsion. The computed orbital overlap *S* between the π–MO**HB–MA** and the lone pair of the nucleophile HOMO−1_Nu_ decreases from 0.141 for TS_uncatalyzed_ to 0.106 for TS**SQ–MA**, which is caused by the aforementioned polarization of the π–MO**HB–MA** away from the reactive carbon center of the C=C bond due to the charge transfer interaction with the hydrogen bond donor catalyst. The squaramide catalyst emerges as the best of our studied bifunctional iminophosphorane squaramide catalysts as it is able to reduce the destabilizing Pauli repulsion between the reactants and thereby impart the greatest reactivity enhancement of our intramolecular aza-Michael reaction.^38^ These systematic computational analyzes explain the origin of reactivity and enantioselectivity in this BIMP squaramide catalyzed aza-Michael reaction.

## Conclusions

In summary, an efficient and highly enantioselective BIMP-catalyzed intramolecular aza-Michael reaction affording decorated hydroquinazoline structures with excellent yields and enantiomeric ratios has been developed. A novel BIMP squaramide system was found to be effective in activating both aromatic and aliphatic ureas. DFT calculations uncovered the fact that the optimal catalyst conformation creates a pocket-like binding site for the substrate to impart enantiofacial selectivity, whilst the squaramide motif demonstrates advantages over urea and thiourea H-bond donor groups on decreasing the destabilizing Pauli repulsion between the reactants (combined ASM and EDA). The catalytic ring formation strategy demonstrated broad functional group tolerance including of esters, nitriles, heterocycles, alkenes, and alkynes, and catalyst loading can be lowered down to 2 mol% in a multi-gram scale synthesis. The hydroquinazoline aza-Michael reaction products were stable towards a series of late-stage structural derivatizations thus demonstrating relevance to pharmaceutical development.

## Author contributions

D. J. D., G. S. and C. J. T. designed the experiments. G. S. performed all the experiments. T. A. H. designed and guided the computational work. K. Y. performed the computations. K. E. C. performed the crystallographic study. G. S., K. Y. and T. A. H. prepared the manuscript and all other authors contributed to the final version of the manuscript.

## Conflicts of interest

There are no conflicts to declare.

## Supplementary Material

SC-012-D1SC00856K-s001

SC-012-D1SC00856K-s002
