# Supplementary material for: A bifunctional iminophosphorane squaramide catalyzed enantioselective synthesis of hydroquinazolines via intramolecular aza-Michael reaction to α,β-unsaturated esters
Source: Chem Sci. 2021 Mar 18;12(17):6064–72. doi: 10.1039/d1sc00856k (PMC8098679; doi:10.1039/d1sc00856k)
Supplement: SC-012-D1SC00856K-s001 [file SC-012-D1SC00856K-s001.pdf]

Electronic Supplementary Material (ESI) for Chemical Science.
This journal is © The Royal Society of Chemistry 2021

Supporting Information

A Bifunctional Iminophosphorane Squaramide Catalyzed Enantioselective Synthesis of Hydroquinazolines via Intramolecular Aza-Michael Reaction to α,β-Unsaturated Esters

Guanglong Su,[a] Connor J. Thomson,[†a] Ken Yamazaki,[†a,b] Daniel Rozsar,[a] Kirsten E. Christensen,[a] Trevor A. Hamlin*[b] and Darren J. Dixon*[a]

[a] Department of Chemistry, Chemistry Research Laboratory, University of Oxford, Mansfield Road, Oxford OX1 3TA (UK). E-mail: darren.dixon@chem.ox.ac.uk
[b] Department of Theoretical Chemistry, Amsterdam Institute of Molecular and Life Sciences (AIMMS), Amsterdam Center for Multiscale Modeling (ACMM), Vrije Universiteit Amsterdam, De Boelelaan 1083, 1081 HV Amsterdam, The Netherlands. E-mail: t.a.hamlin@vu.nl

Supporting Information

Contents

1. General Experimental Data 1

2. Optimization Results 3

3. Preparation of Catalyst Precursor and Catalyst 9

4. Preparation of Starting Materials 20

5. Preparation of Racemic Compounds 54

6. Preparation of Enantioenriched Products 54

7. Derivatization and Scale-up 86

8. Single Crystal X-Ray Diffraction Data 95

9. Computational Studies 98

10. NMR Spectra 169

11. HPLC Traces 250

12. References 286

1. General Experimental Details

Reagents and solvents were purchased at reagent-grade from Acros Organics, Sigma-Aldrich, Alfa Aeser, and Fluorochem and used without further purification unless stated. Solvents for extraction or column chromatography were of technical quality. All water used was purified via a Merck Millipore reverse osmosis purification system prior to use. All reactions were performed under N_2 atmosphere if not stated otherwise. Anhydrous solvents (tetrahydrofuran, toluene, dichloromethane, and diethyl ether) were dried by filtration through activated alumina (Sigma-Aldrich, 58 Å pore size, powder 150 mesh, basic) columns and stored under N_2 atmosphere prior to use. Solvents were removed under reduced pressure using Büchi Rotavapor apparatus.

Thin-layer chromatography was performed on SiO_2-60 UV_{254} coated aluminium sheets from Merck (silica gel 60 F254). Visualisation was achieved with a UV lamp at a wavelength of 254 nm, or with a $KMnO_4$ solution.

Flash column chromatography was carried out on silica gel 60 (VWR, 40-63 μm). Solvents for extraction and chromatography were of technical quality. Solvent mixtures are individually reported in parenthesis.

Proton, Carbon, and Fluorine nuclear magnetic resonance (^{1}H, ^{13}C, and ^{19}F NMR) spectra were recorded on Bruker DPX200 (200 MHz) Bruker AVG400 (400/101 MHz), Bruker AVH400 (400/101 MHz), Bruker AVC500 (500/126 MHz) and Bruker AVB500 (500/126 MHz) NMR spectrometers at 25 °C. Chemical shifts (δ) are given in ppm, coupling constants (*J*) in Hz. Peak multiplicities are described as singlet (s), doublet (d), triplet (t), pentet (p), or a combination e.g. doublet of doublets, or as a multiplet over a peak range. Some peaks are described as broad (b). The residual deuterated solvent was used as internal standard ($CDCl_3$: δ_H = 7.26 ppm; CD_3OD: δ_H = 3.31 ppm; $(CD_3)_2SO$: δ_H = 2.50 ppm). HSQC, COSY and HMBC experiments were used for ^{1}H and ^{13}C NMR signals assignment where required.

Melting points (m.p.) were determined with a Leica Galen III Hot-stage melting point apparatus and microscope and on a Kofler hot block and are reported uncorrected.

Infrared (IR) spectra were recorded on a Bruker Tensor 27 FT-IR spectrometer as a thin film.

High-resolution mass spectrometry (HR-MS-ESI) was performed on a Bruker μTOF mass spectrometer. The molecular ion (M^+) is reported in *m/z* units.

Chiral HPLC The enantiomeric excesses were determined by HPLC analysis on an Agilent 1200 Series instrument employing a chiral stationary phase column specified in the individual experiment and by comparing the samples with the appropriate racemic mixtures.

$[\alpha]_D{}^T$ Optical rotations were recorded using a Perkin Elmer 341 polarimeter; **$[\alpha]_D{}^T$** values are reported in 10^{-1} deg·cm^2 g^{-1} ; concentrations (c) are quoted in g/100 mL; D refers to the D-line of sodium (589 nm); temperatures (T) are given in degrees Celsius (°C). (+) and (–) compound number prefixes indicate the sign of the optical rotation.

2. Optimization Results

General Procedure for Optimization of Model Reaction:

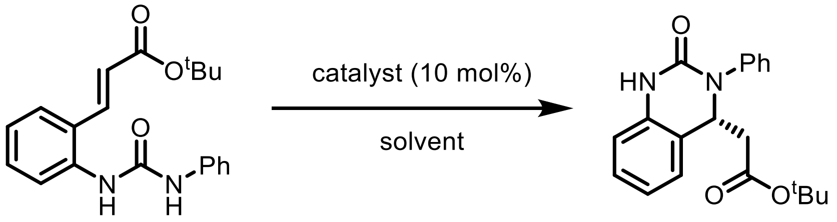

1a 2a

Urea **1a** (16.9 mg, 0.05 mmol, 1.0 eq) was added to a solution of catalyst (0.005 mmol, 0.1 eq) in the appropriate solvent under N_2 at room temperature. The reaction mixture was stirred at the specified temperature for 24 hours unless otherwise indicated. Purification by silica gel chromatography (pentane/EtOAc) afforded the pure products, which were taken the isolated yield and analysed by chiral HPLC. (Chiralcel OD, hexane/isopropanol = 95/5, 1.0 ml/min, λ = 220 nm, t_R (major) = 36.6 min, t_R (minor) = 57.4 min)

Table S1: Catalyst Screen

entry	cat. X	Conc.	temp/°C	solvent	reaction time	Yield (%)[a]	er[b]
1	**A**	0.1M	rt	toluene	24h	0	N.D.
2	**B**	0.1M	rt	toluene	24h	0	N.D.
3	**C**	0.1M	rt	toluene	24h	0	N.D.
4	**D**	0.1M	rt	toluene	24h	0	N.D.
5	**E**	0.1M	rt	toluene	24h	0	N.D.
6	**F**	0.1M	rt	Et_2O	24h	96	68.5:31.5
7	**G**	0.1M	rt	Et_2O	24h	95	63:37
8	**H**	0.1M	rt	Et_2O	24h	73	74:26
9	**I**	0.1M	rt	Et_2O	24h	22	75.5:24.5
10	**J**	0.1M	rt	Et_2O	24h	51	71:29
11	**K**	0.1M	rt	Et_2O	24h	16	62:38
12	**L**	0.1M	rt	Et_2O	24h	32	66.5:33.5
13	**M**	0.1M	rt	Et_2O	24h	36	69:31
14	**N**	0.1M	rt	Et_2O	24h	99	53.5:46.5
15	**O**	0.1M	rt	Et_2O	24h	99	60:40
16	**P**	0.1M	rt	Et_2O	24h	99	60:40
17	**Q**	0.1M	rt	Et_2O	24h	>99	74.5:25.5

entry	cat. X	Conc.	temp/ºC	solvent	reaction time	Yield (%)[a]	er[b]
18	**R**	0.1M	rt	Et_2O	24h	>99	71.5:28.5
19	**S**	0.1M	rt	Et_2O	24h	0	N.D.
20	**T**	0.1M	rt	Et_2O	24h	>99	75:25
21	**U**	0.1M	rt	Et_2O	24h	>99	72.5:27.5
22	**V**	0.025M	rt	toluene	4h	11	65.5:34.5
23	**W**	0.025M	rt	toluene	4h	53	67.5:32.5
24	**X**	0.025M	rt	toluene	4h	36	70:30
25	**Y**	0.025M	rt	toluene	4h	17	68.5:31.5
26	**Z**	0.025M	rt	toluene	10h	76	79:21
27	**AA**	0.025M	rt	toluene	10h	71	80.5:19.5
28	**AB**	0.025M	rt	toluene	4h	23	70:30
29	**AC**	0.025M	rt	toluene	4h	24	71.5:28.5
30	**AD**	0.025M	rt	toluene	4h	62	80:20
31	**AE**	0.1M	rt	Et_2O	24h	10	67:33
32	**AF**	0.025M	rt	toluene	24h	91	54.5:45.5
33	**AG**	0.025M	rt	toluene	24h	>99	91.5:8.5
**34**	**AH**	**0.025M**	**rt**	**toluene**	**24h**	**>99**	**94.5:5.5**
35	**AI**	0.025M	rt	toluene	24h	>99	83.5:16.5
36	**AJ**	0.025M	rt	toluene	24h	>99	92.5:7.5
37	**AK**	0.025M	rt	toluene	24h	>99	92:8

Table S1. Detailed catalyst screen results for the intramolecular aza-Michael reaction of urea **1a**. [a] determined by ^1H NMR analysis of crude reaction mixture. [b] determined by HPLC analysis on chiral stationary phase. rt = room temperature. N.D. = not determined.

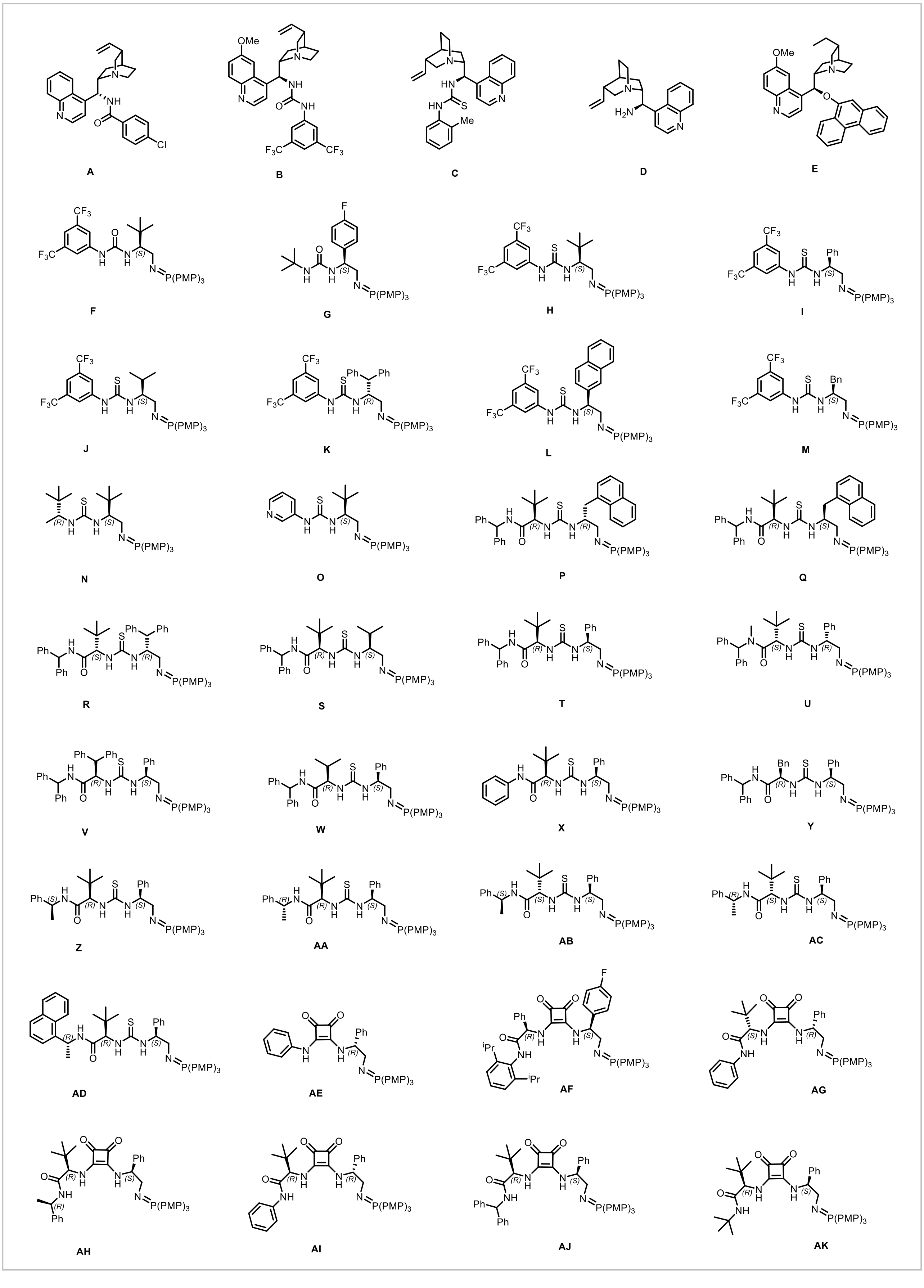

Figure S1. Selected catalysts studied in the optimization of the intramolecular aza-Michael reaction of urea **1a**.

Table S2: Solvent and Conditions Optimization

entry	cat. X	Conc.	temp/°C	solvent	reaction time	Yield (%)[a]	er[b]
1	**Q**	0.1M	rt	Et_2O	4h	>99	74.5:25.5
2	**Q**	0.1M	rt	Et_2O	9h	97	75:25
3	**Q**	0.1M	rt	Et_2O	24h	>99	74.5:25.5
4	**Q**	0.1M	rt	EtOAc	9h	60	67.5:32.5
5	**Q**	0.1M	rt	TBME	9h	>99	76:24
6	**Q**	0.1M	rt	dioxane	9h	51	77.5:22.5
7	**Q**	0.1M	rt	toluene	9h	>99	76.5:23.5
8	**Q**	0.1M	rt	THF	9h	73	70:30
9	**Q**	0.1M	rt	CPME	9h	>99	76:24
10	**Q**	0.1M	rt	PhF	4h	>99	69.5:30.5
11	**Q**	0.1M	rt	2-methyl THF	4h	64	74:26
12	**Q**	0.1M	-15	toluene	4h	47	63.5:36.5
13	**Q**	0.1M	10	toluene	4h	67	70.5:29.5
14	**Q**	0.1M	35	toluene	4h	>99	77.5:22.5
15	**Q**	0.1M	55	toluene	4h	>99	77.5:22.5
16	**Q**	0.3M	rt	toluene	4h	98	74.5:25.5
17	**Q**	0.025M	rt	toluene	4h	99	78:22
18	**Q**	0.025M	55	toluene	4h	>99	78.5:21.5
19	**Q**	0.025M	75	toluene	4h	>99	77.5:22.5
20	**Q**	0.025M	95	toluene	4h	>99	77:23
21	**AG**	0.1M	rt	toluene	24h	>99	90.5:9.5
22	**AG**	0.025M	rt	toluene	24h	>99	91.5:8.5
23	**AG**	0.025M	-15	toluene	24h	trace	N.D.
24	**AG**	0.025M	50	toluene	24h	>99	90.5:9.5
25	**AG**	0.025M	5	toluene	24h	22	91.5:8.5
26	**AG**	0.025M	rt	$PhCF_3$	24h	0	N.D.
27	**AG**	0.025M	rt	MeCN	24h	>99	56:44
28	**AG**	0.025M	rt	EtOAc	24h	77	80.5:19.5
29	**AG**	0.025M	rt	DCM	24h	>99	72.5:27.5
30	**AG**	0.025M	rt	CPME	24h	79	85.5:14.5
31	**AG**	0.025M	rt	THF	24h	>99	66.5:33.5
**32**	**AH**	**0.025M**	**rt**	**toluene**	**24h**	**>99**	**94.5:5.5**
33	**AH**	0.025M	rt	PhF	24h	91	89:11
34	**AH**	0.025M	rt	*o*-xylene	24h	>99	94.2:5.8
35	**AH**	0.025M	rt	*p*-xylene	24h	87	91.5:8.5
36	**AH**	0.025M	rt	*m*-xylene	24h	92	94.5:5.5

Table S2. Detailed solvent and conditions optimization results for the intramolecular aza-Michael reaction of urea **1a**. [a] yields of isolated products. [b] determined by HPLC analysis on chiral stationary phase. rt = room temperature. N.D. = not determined. TBME = *tert*-butyl methyl ether. CPME = cyclopentyl methyl ether.

Table S3: Phosphine Screen[a]

entry	cat. X	Conc.	PR_3	solvent	Yield (%)[b]	er[c]
**1[d]**	**Q**	**0.1M**	**$P(PMP)_3$**	**toluene**	**>99**	**76.5:23.5**
2	**Q**	0.1M	PPh3	toluene	24	76:24
3	**Q**	0.1M	P(4-Cl-Ph)3	toluene	0	N.D.
4[e]	**Q**	0.1M	$P(^nBu)_3$	toluene	>99	65:35
5	**Q**	0.1M	dppf	toluene	44	69:31
6	**Q**	0.1M	dppe	toluene	34	65:35
7	**Q**	0.1M	dppm	toluene	>99	67:33
8	**Q**	0.1M	I	toluene	>99	60:40
9	**Q**	0.1M	II	toluene	>99	64:36
10	**Q**	0.1M	III	toluene	0	N.D.
11[e]	**R**	0.1M	$P(PMP)_3$	Et_2O	>99	71.5:28.5
12	**R**	0.1M	IV	Et_2O	76	66:34
13	**R**	0.1M	V	Et_2O	24	62.5:37.5
14	**R**	0.1M	VI	Et_2O	0	N.D.
15	**R**	0.1M	VII	Et_2O	31	60:40
16	**R**	0.1M	VIII	Et_2O	0	N.D.

Table S3. Detailed phosphine screen results for the intramolecular aza-Michael reaction of urea **1a**. [a] reaction was carried out at room temperature for 4 hours unless otherwise indicated. [b] yields of isolated products. [c] determined by HPLC analysis on chiral stationary phase. [d] reaction was carried out for 9 hours. [e] reaction was carried out for 24 hours. N.D. = not determined. PMP = *para*-methoxy phenyl. dppm = 1,1-Bis(diphenylphosphino)methane. dppf = 1,1'-Bis(diphenylphosphino)ferrocene. dppe = 1,2-Bis(diphenylphosphino)ethane.

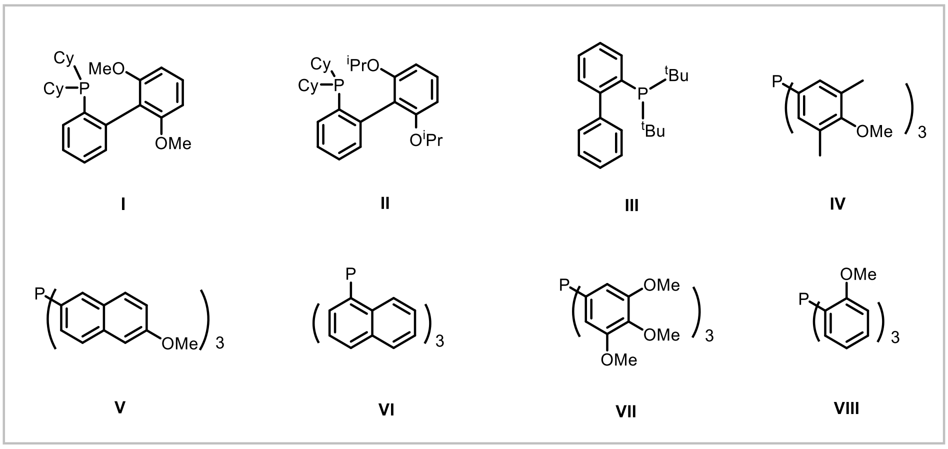

Figure S2. Selected phosphines studied in the optimization of the intramolecular aza-Michael reaction of urea **1a**.

Scheme S1: Evaluation of Other Conjugate Acceptors

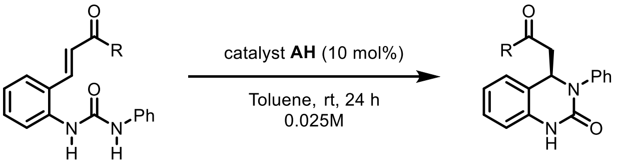

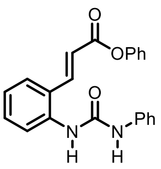

99% yield, < 75:25 e.r.

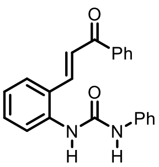

99% yield, 91.5:8.5 e.r.

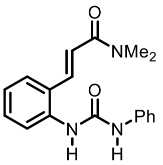

99% yield, 57:43 e.r.

3. Preparation of Catalyst Precursor and Catalyst

3.1 Synthesis of Catalyst Precursor

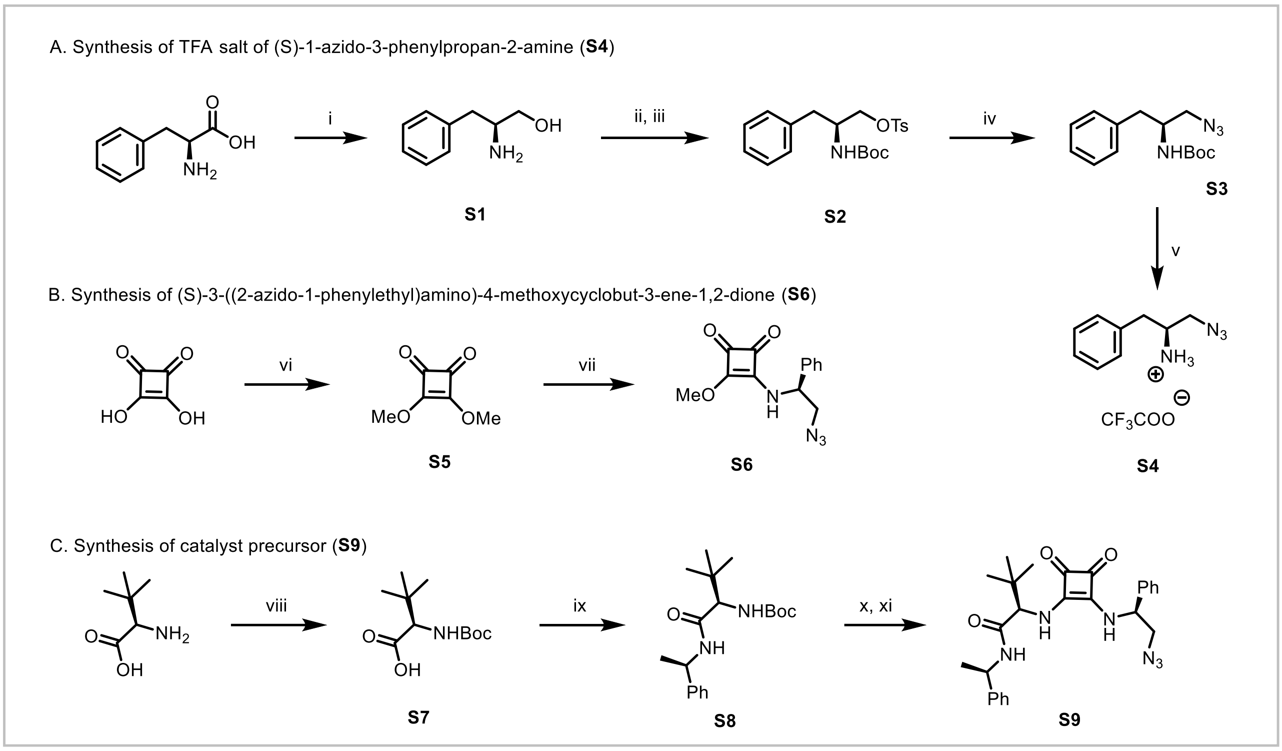

Scheme S2. A). Synthesis of (*S*)-1-azido-3-phenylpropan-2-amine (**S4**). i. $NaBH_4$, I_2, THF, reflux, 20h, 75%; ii. Et_3N, $(Boc)_2O$, DCM, rt, 8h; iii. Et_3N, TsCl, DCM, rt, 16h, 59% over ii and iii; iv. NaN_3, DMF, 45 ^oC, 14h, 46%; v. TFA, 0 ^oC to rt, 2h, quantative yield. **B).** Synthesis of (S)-3-((2-azido-1-phenylethyl)amino)-4-methoxycyclobut-3-ene-1,2-dione (**S6**). vi. trimethyl orthoformate, MeOH, reflux, 24h, 82%; vii. compound **XX**, Et_3N, MeOH, rt, 16h, 51%. **C).** Synthesis of catalyst precursor (**S9**). viii. $(Boc)_2O$, Na_2CO_3, $MeOH/H_2O$, rt, 24h, 96%; ix. (*R*)-1-phenylethan-1-amine, HATU, Et_3N, DCM, rt, 20h, 94%; x. TFA, 0 ^oC to rt, 2h; xi. compound **S6**, Et_3N, MeOH, 50 ^oC, 20h, 80%.

3.2 Synthesis of Catalyst

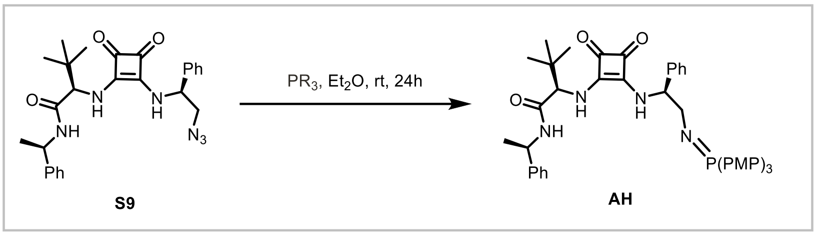

Staudinger reaction for *in-situ* generation of the active BIMP catalysts: Et_2O (0.025 M) was added to the BIMP catalysts precursors (1.0 eq) and the appropriate phosphine (1.0 eq) in a mass spectrometry vial under N_2 at room temperature. The reaction mixture was stirred for 24 hours before evaporating to dryness under a stream of nitrogen gas. The iminophosphorane product was confirmed by LRMS and TLC. The resulting mixture was used as crude for enantioselective reactions without any purification.

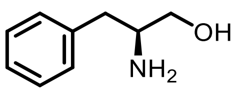

(*S*)-2-amino-3-phenylpropan-1-ol (S1) was prepared according to the literature procedure reported by S. Ruppenthal *et. al.* [1] A solution of I_2 (15.37 g, 60.54 mmol, 1.0 eq) in THF (40 mL) was added dropwise to a solution of $NaBH_4$ (5.50 g, 145.30 mmol, 2.4 eq) and *L*-phenylalanine (10.00 g, 60.54 mmol, 1.0 eq) in THF (160 mL) over 30 mins under N_2 at 0 °C. The reaction mixture was heated to reflux for 20 hours. After cooled back to room temperature, methanol (25 mL) was added dropwise to the mixture. The resulting clear solution was stirred for 30 mins and evaporated to dryness under reduced pressure. 20% KOH solution (200 mL) was then added and stirring was maintained for 4 hours. The mixture was extracted with CH_2Cl_2 (3 x 100 mL). The combined organic layers were washed with brine (100 mL), dried over Na_2SO_4, filtered and evaporated to dryness under reduced pressure. Purification by recrystallization from toluene afforded the title compound as a white solid (6.86 g, 75%). Data is consistent with the published literature.

^{1}H NMR (400 MHz, $CDCl_3$): δ 7.34 – 7.16 (m, 5H, Ar-$\underline{H}$), 3.63 (dd, J = 10.7, 3.8 Hz, 1H, C$\underline{H}_2$OH), 3.39 (dd, J = 10.7, 7.2 Hz, 1H, C$\underline{H}_2$OH), 3.12 (m, 1H, C$\underline{H}$NH$_2$), 2.79 (dd, J = 13.4, 5.2 Hz, 1H, PhC$\underline{H}_2$), 2.52 (dd, J = 13.4, 8.7 Hz, 1H, PhC$\underline{H}_2$), 2.12 (brs, 3H, N$\underline{H}_2$ and O$\underline{H}$) ppm.

^{13}C NMR (101 MHz, $CDCl_3$) δ 138.8 (Ar$\underline{C}$), 129.3 (*m*-Ar$\underline{C}$H), 128.7 (*o*-Ar$\underline{C}$H), 126.5 (*p*-Ar$\underline{C}$H), 66.4 ($\underline{C}$H$_2$OH), 54.3 (NH$_2\underline{C}$H), 40.9 (Ph$\underline{C}$H$_2$) ppm.

HRMS (ESI) m/z calcd. for $C_9H_{14}NO$ ($[M+H]^+$) 152.10699, found 152.10725

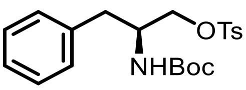

(*S*)-2-((*tert*-butoxycarbonyl)amino)-3-phenylpropyl 4-methylbenzenesulfonate (S2) was prepared according to the literature procedure reported by S. Bera, *et. al.* [2] Triethylamine (3.95 g, 39.02 mmol, 1.2 eq) was added to a solution of (*S*)-2-amino-3-phenylpropan-1-ol (**S1**) (5.00 g, 33.07 mmol, 1.0 eq) in CH_2Cl_2 (116 ml) under N_2 at room temperature. The reaction mixture was cooled to 0 °C and di-*tert*-butyl decarbonate (8.52 g, 39.02 mmol, 1.2 eq) was added. The stirring was maintained at 0 °C for 1 hour and then room temperature for 8 hours. The mixture was washed with water (100 mL) and brine (100 mL). The combined organic layers were dried over $MgSO_4$, filtered and evaporated to dryness under reduced pressure. Purification by silica gel chromatography (pentane/EtOAC = 3/2 then 2/3) afforded the *N*-boc protected amino alcohol as a white solid (7.32 g, 88%).

Triethylamine (3.1 g, 30.64 mmol, 1.1 eq) and *p*-toluenesulfonyl chloride (5.31g, 27.85 mmol, 1.0 eq) were added sequentially to a solution of the *N*-boc protected amino alcohol (7.00 g, 27.85 mmol, 1.0 eq) in CH_2Cl_2 (103 mL) under N_2 at room temperature. The reaction mixture was stirred for 16 hours and partitioned by the addition of H_2O (100mL). The aqueous layer was extracted with CH_2Cl_2 (3 x 100 mL). The combined organic layers were washed with brine (200 mL), dried over $MgSO_4$, filtered and evaporated to dryness under reduced pressure. Purification by silica gel chromatography (pentane/EtOAC = 9/1) afforded the title compound as a pale yellow solid (7.56 g, 67%). Data is consistent with the published literature.

^{1}H NMR (400 MHz, $CDCl_3$): δ 7.78 (d, *J* = 8.0 Hz, 2H, $C_6\underline{H}_4CH_3$), 7.35 (d, *J* = 8.0 Hz, 2H, $C_6\underline{H}_4CH_3$), 7.25 – 7.15 (m, 3H, $C_6\underline{H}_5CH_2$), 7.12 – 7.04 (m, 2H, $C_6\underline{H}_5CH_2$), 4.76 – 4.70 (brs, 1H, N$\underline{H}$), 4.06 – 3.85 (m, 3H, NHC$\underline{H}$ and C$\underline{H}_2$O), 2.93 – 2.72 (m, 2H, PhC$\underline{H}_2$), 2.46 (s, 3H, $C_6H_4C\underline{H}_3$), 1.38 (s, 9H, $C(C\underline{H}_3)_3$) ppm.

^{13}C NMR (101 MHz, $CDCl_3$) δ 155.1 (O$\underline{C}$(O)N), 145.2 ($SO_2Ar\underline{C}$), 136.9 (Ar$\underline{C}$), 132.7 ($SO_2Ar\underline{C}CH_3$), 130.1 (*m*-$SO_2Ar\underline{C}H$), 129.4 (*m*-Ar$\underline{C}$H), 128.7 (*o*-Ar$\underline{C}$H), 128.1 (*o*-$SO_2Ar\underline{C}H$), 126.9 (*p*-Ar$\underline{C}$H), 80.0 ($\underline{C}(CH_3)_3$), 70.1 ($\underline{C}H_2O$), 50.9 (NH$\underline{C}$H), 37.3 (Ph$\underline{C}H_2$), 28.4 ($C(\underline{C}H_3)_3$), 21.8 ($C_6H_4\underline{C}H_3$) ppm.

HRMS (ESI) m/z calcd. for $C_{21}H_{27}NO_5{}^{23}Na^{32}S$ ($[M+Na]^+$) 428.15021, found 428.14987

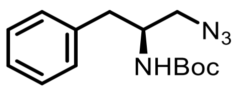

***tert*-Butyl (*S*)-(1-azido-3-phenylpropan-2-yl) carbamate (S3)** was prepared according to the literature procedure reported by S. Bera, *et. al.* [2] Sodium azide (1.30 g, 19.96 mmol, 1.1 eq) was added to a solution of (*S*)-2-((*tert*-butoxycarbonyl)amino)-3-phenylpropyl 4-methylbenzenesulfonate (**S2**) (7.36 g, 18.15 mmol, 1.0 eq) in DMF (61 mL) under N_2 at room temperature. The reaction mixture was warmed to 45 ^oC and stirred for 14 hours. The mixture was cooled to room temperature and diluted with water (100 mL). The aqueous layer was extracted with Et_2O (3 x 100 mL). The combined organic layers were washed with brine (100 mL), dried over $MgSO_4$, filtered and evaporated to dryness under reduced pressure. Purification by silica gel chromatography (pentane/Et_2O = 9/1 then 4/1) afforded the title compound as a white solid (2.33 g, 46%). Data is consistent with the published literature.

^{1}H NMR (400 MHz, $CDCl_3$): δ 7.35 – 7.17 (m, 5H, Ar-H), 4.65 (brs, 1H, NH), 3.97 (brs, 1H, NHCH), 3.46 – 3.27 (m, 2H, CH_2N_3), 2.94 – 2.74 (m, 2H, $PhCH_2$), 1.43 (s, 9H, $C(CH_3)_3$) ppm.

^{13}C NMR (101 MHz, $CDCl_3$) δ 155.2 (OC(O)N), 137.3 (ArC), 129.4 (*m*-ArCH), 128.8 (*o*-ArCH), 126.9 (*p*-ArCH), 79.9 ($C(CH_3)_3$), 53.3 (CH_2N_3), 51.5 (NHCH), 38.3 ($PhCH_2$), 28.5 ($C(CH_3)_3$) ppm.

HRMS (ESI) m/z calcd. for $C_{14}H_{20}N_4O_2{}^{23}Na$ ($[M+Na]^+$) 299.14785, found 299.14781

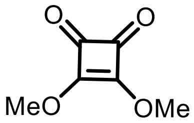

3,4-dimethoxycyclobut-3-ene-1,2-dione (S5) was prepared according to the literature procedure reported by M. Lu *et. al.* [3] Trimethyl orthoformate (13.70 g, 129.00 mmol, 2.1 eq) was added to a solution of 3,4-Dihydroxy-3-cyclobutene-1,2-dione (7.00 g, 61.40 mmol, 1.0 eq) in anhydrous MeOH (62 mL) under N_2 at room temperature. The reaction mixture was heated to reflux for 24 hours. After cooled back to room temperature, the mixture was evaporated to dryness under reduced pressure. Purification by silica gel chromatography (pentane/EtOAc= 2/1) afforded the title compound as a white solid (7.11 g, 82%). Data is consistent with the published literature.

^{1}H NMR (400 MHz, CDCl$_3$): δ 4.36 (s, 6H, C$\underline{H}_3$) ppm.

^{13}C NMR (101 MHz, CDCl$_3$) δ 189.3 ($\underline{C}$=O), 184.6 ($\underline{C}$OCH$_3$), 61.1 ($\underline{C}$H$_3$) ppm.

HRMS (ESI) m/z calcd. for $C_6H_7O_4$ ([M+H]$^+$) 143.0339, found 143.0340

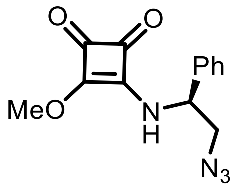

(*S*)-3-((2-azido-1-phenylethyl)amino)-4-methoxycyclobut-3-ene-1,2-dione (S6) was prepared according to the following procedure. According to the modified literature procedure reported by D. M. Rotstein *et. al.*, [4] trifluoroacetic acid (7.8 mL) was added to the *tert*-butyl (*S*)-(1-azido-3-phenylpropan-2-yl) carbamate **(S3)** (2.03 g, 7.74 mmol, 1.0 eq) under N_2 at 0 °C. The reaction mixture was stirred for 2 hours at 0 °C, before warming to room temperature. Volatiles were removed under a stream of nitrogen gas behind a blast shield to afford TFA salt of (*S*)-1-azido-3-phenylpropan-2-amine (**S4**) which was used as crude for next step without any purification.

A solution of 3,4-dimethoxycyclobut-3-ene-1,2-dione (**S5**) (2.21 g, 15.50 mmol, 2.0 eq) and triethylamine (5.48 g, 54.20 mmol, 7.0 eq) in anhydrous MeOH (39 mL) was added to a solution of the TFA salt of (*S*)-1-azido-3-phenylpropan-2-amine (**S4**) in anhydrous MeOH (116 mL) over 2h under N_2 at room temperature. The reaction mixture was stirred for 24 hours before evaporating to dryness under reduced pressure. Purification by silica gel chromatography (CH_2Cl_2/MeCN = 9/1) afforded the title compound as a pale yellow oil (1.07 g, 51%).

^{1}H NMR (400 MHz, $CDCl_3$): δ 7.74 (brs, 1H, NH), 7.43 – 7.32 (m, 5H, Ar-H), 4.87 (brs, 1H, CH), 4.41 (s, 3H, CH_3), 3.83 (dd, *J* = 12.7, 8.2 Hz, 1H, CH_2), 3.71 (brs, 1H, CH_2) ppm.

^{13}C NMR (101 MHz, $CDCl_3$) δ 179.1 (CO), 171.7 (CO), 137.9 (ArC), 129.4 (ArCH), 129.0 (ArCH), 126.6 (ArCH), 61.0 (CH_3), 59.1 (CH), 55.6 (CH_2) ppm. (C=C not observed)

HRMS (ESI) m/z calcd. for $C_{13}H_{13}N_4O_3$ ($[M+H]^+$) 273.0982, found 273.0981

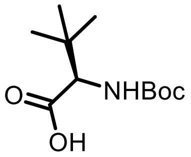

(*R*)-2-((tert-butoxycarbonyl)amino)-3,3-dimethylbutanoic acid (S7) was prepared according to the literature procedure reported by M. S. Manna, *et. al.* [5] Na_2CO_3 (4.55 g, 42.90 mmol, 1.1 eq) was added to a solution of (*D*)-tert-leucine (5.13 g, 39.00 mmol, 1.0 eq) in MeOH/H_2O (1:1) (156 mL) under N_2 at room temperature. Di-*tert*-butyl decarbonate (9.37 g, 42.90 mmol, 1.1 eq) was added and the resulting mixture was stirred at room temperature for 20 h. MeOH was evaporated under reduced pressure. The aqueous mixture was acidified to pH = 4 with saturated aqueous solution of citric acid (200 mL) and then extracted with CH_2Cl_2 (4 × 100 mL) and Et_2O (2 × 50 mL). The combined organic layers were washed with brine (100 mL), dried over Na_2SO_4, filtered and evaporated to dryness under reduced pressure to afford title compound as a white foam (4:1 (**A**:**B**) mixture of rotamers), (8.71 g, 96%) which was used as crude for next step without any purification. Data is consistent with the published literature.

^{1}H NMR (400 MHz, CD_3OD): δ 3.95 (s, 0.8H, C<u>H</u> (**A**)), 3.83 (s, 0.2H, C<u>H</u> (**B**)), 1.45 (s, 9H, OC(C<u>H</u>$_3$)$_3$), 1.00 (s, 9H, CHC(C<u>H</u>$_3$)$_3$) ppm.

^{13}C NMR (101 MHz, CD_3OD) δ 174.8 (<u>C</u>O$_2$), 158.0 (NH<u>C</u>O$_2$), 80.5 (O<u>C</u>(CH$_3$)$_3$), 63.4 (<u>C</u>HC(CH$_3$)$_3$), 34.9 (CH<u>C</u>(CH$_3$)$_3$), 28.7 (OC(<u>C</u>H$_3$)$_3$), 27.1 (CHC(<u>C</u>H$_3$)$_3$) ppm.

HRMS (ESI) m/z calcd. for $C_{11}H_{21}NO_4{}^{23}Na$([M+Na]$^+$) 254.1374, found 254.1364

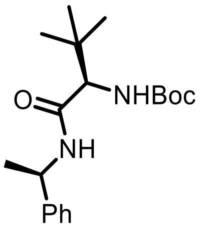

***tert*-Butyl ((*R*)-3,3-dimethyl-1-oxo-1-(((*R*)-1-phenylethyl)amino)butan-2-yl)carbamate (S8)** was prepared according to the following procedure. Triethylamine (0.90 g, 8.80 mmol, 2.0 eq) was added to a solution of (*R*)-2-((*tert*-butoxycarbonyl)amino)-3,3-dimethylbutanoic acid **(S7)** (1.02 g, 4.40 mmol, 1.0 eq), (*R*)-1-phenylethan-1-amine (1.07 g, 8.80 mmol, 2.0 eq) and HATU (2.01 g, 5.28 mmol, 1.2 eq) in CH_2Cl_2 (22 mL) under N_2 at room temperature. The reaction mixture was stirred at room temperature for 24 hours before quenching with a saturated aqueous solution of $NaHCO_3$ (50 mL). The aqueous layer was extracted with CH_2Cl_2 (3 x 50 mL). The combined organic layers were washed with brine (100 mL), dried over Na_2SO4, filtered and evaporated to dryness under reduced pressure. Purification by silica gel chromatography (pentane/EtOAc= 9/1) afforded the title compound as a white solid (1.39 g, 94%).

^{1}H NMR (400 MHz, CDCl$_3$): δ 7.38 – 7.22 (m, 5H, Ar-H), 5.88 (d, *J* = 7.5 Hz, 1H, NHCO), 5.32 – 5.19 (m, 1H, $NHCO_2$), 5.11 (p, *J* = 7.0 Hz, 1H, $CHCH_3$), 3.76 (d, *J* = 9.3 Hz, 1H, $CHC(CH_3)_3$), 1.49 (d, *J* = 7.0 Hz, 3H, $CHCH_3$), 1.44 (s, 9H, $OC(CH_3)_3$), 0.93 (s, 9H, $CHC(CH_3)_3$) ppm.

^{13}C NMR (101 MHz, CDCl$_3$) δ 170.1 (NHCO), 156.0 ($NHCO_2$), 143.1 (ArC), 128.8 (*m*-ArCH), 127.6 (*p*-ArCH), 126.4 (*o*-ArCH), 79.8 ($OC(CH_3)_3$), 62.7 ($CHC(CH_3)_3$), 49.1 ($CHCH_3$), 34.8 ($CHC(CH_3)_3$), 28.5 ($OC(CH_3)_3$), 26.7 ($CHC(CH_3)_3$), 21.7 ($CHCH_3$) ppm.

HRMS (ESI) m/z calcd. for $C_{19}H_{30}N_2O_3{}^{23}Na$ ($[M+Na]^+$) 357.2149, found 357.2149

FT-IR (thin film) ν_{max} 3312, 2972, 2524, 2160, 2030, 1977, 1685, 1648, 1523, 1454, 1391, 1366, 1322, 1246, 1173, 1128, 1062, 1009, 913, 863, 759, 700 cm^{-1}.

m .p.: 141-142 °C

$[\alpha]_D^{25}$ = +47.5 (c=0.99, $CHCl_3$).

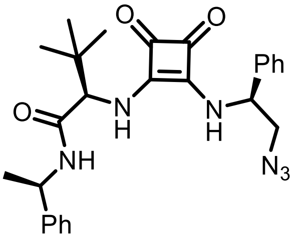

Catalyst Precursor (*R*)-2-((2-(((*S*)-2-azido-1-phenylethyl)amino)-3,4-dioxocyclobut-1-en-1-yl)amino)-3,3-dimethyl-*N*-((*R*)-1-phenylethyl)butanamide (S9) was prepared according to the following procedure. According to the modified literature procedure reported by D. M. Rotstein *et. al.,* [4] trifluoroacetic acid (2.7 mL) was added to the *tert*-butyl ((*R*)-3,3-dimethyl-1-oxo-1-(((*R*)-1-phenylethyl)amino)butan-2-yl)carbamate **(S8)** (0.89 g, 2.67 mmol, 1.0 eq) under N_2 at 0 °C. The reaction mixture was stirred for 2 hours at 0 °C, before warming to room temperature. Volatiles were removed under a stream of nitrogen gas behind a blast shield to afford TFA salt of (*R*)-2-amino-3,3-dimethyl-*N*-((*R*)-1-phenylethyl)butanamide which was used as crude for next step without any purification.

A solution of the TFA salt of (*R*)-2-amino-3,3-dimethyl-*N*-((*R*)-1-phenylethyl)butanamide in anhydrous MeOH (5.7 mL) was added to a solution of (*S*)-3-((2-azido-1-phenylethyl)amino)-4-methoxycyclobut-3-ene-1,2-dione **(S6)** (0.73 g, 2.67 mmol, 1.0 eq) and triethylamine (1.89 g, 18.70 mmol, 7.0 eq) in anhydrous MeOH (3.2 mL) under N_2 at room temperature. The reaction mixture was heated to 50 °C and stirred for 20 hours. The reaction mixture was cooled to room temperature and evaporated to dryness under reduced pressure. Purification by silica gel chromatography (CH_2Cl_2/MeCN = 4/1) afforded the title compound as a white solid (1.02 g, 80%).

^{1}H NMR (400 MHz, $(CD_3)_2SO$): δ 8.93 (d, *J* = 8.2 Hz, 1H, NHCO), 8.33 (d, *J* = 9.1 Hz, 1H, NHCHCH$_2$), 7.95 (d, *J* = 10.0 Hz, 1H, NHCHCO), 7.47 – 7.39 (m, 4H, Ar-H), 7.38 – 7.29 (m, 5H, Ar-H), 7.25 – 7.19 (m, 1H, Ar-H), 5.46 – 5.25 (m, 1H, NHCHCH$_2$), 4.98 (p, *J* = 7.0 Hz, 1H, NHCHCH$_3$), 4.51 (d, *J* = 10.0 Hz, 1H, NHCHCO), 3.94 – 3.74 (m, 2H, NHCHCH$_2$), 1.37 (d, *J* = 7.0 Hz, 3H, NHCHCH$_3$), 0.82 (s, 9H, CHC(CH$_3$)$_3$) ppm.

^{13}C NMR (101 MHz, $(CD_3)_2SO$) δ 182.31 (CCO), 182.25 (CCO), 168.2 (NHCO), 167.5 (CCO), 166.7 (CCO), 144.3 (ArCCHCH$_3$), 139.1 (ArCCHCH$_2$), 128.8 (*m*-ArCH), 128.2 (*m*-ArCH), 128.0 (*p*-ArCH),

126.7 (*p*-Ar<u>C</u>H), 126.6 (*o*-Ar<u>C</u>H), 126.2 (*o*-Ar<u>C</u>H), 63.6 (<u>C</u>HC(CH_3)$_3$), 56.4 (NH<u>C</u>HCH_2), 55.6 (NHCH<u>C</u>H_2), 48.1 (<u>C</u>HCH_3), 35.5 (CH<u>C</u>(CH_3)$_3$), 26.0 (CHC(<u>C</u>H_3)$_3$), 22.5 (CH<u>C</u>H_3) ppm.

HRMS (ESI) m/z calcd. for $C_{26}H_{31}N_6O_3$ ($[M+H]^+$) 475.2452, found 475.2452

FT-IR (thin film) ν_{max} 3448, 2996, 2913, 2360, 2160, 1978, 1665, 1437, 1407, 1311, 1043, 1024, 953, 932, 898, 698, 668 cm^{-1}.

m.p.: 235-237 °C

$[\alpha]_D^{25}$ = +1.7 (c=0.65, $(CH_3)_2SO$).

4. Preparation of Starting Materials

4.1. General Procedure A for Preparation of Starting Materials 1a to 1q, 1s to 1t and 1z to 1ae

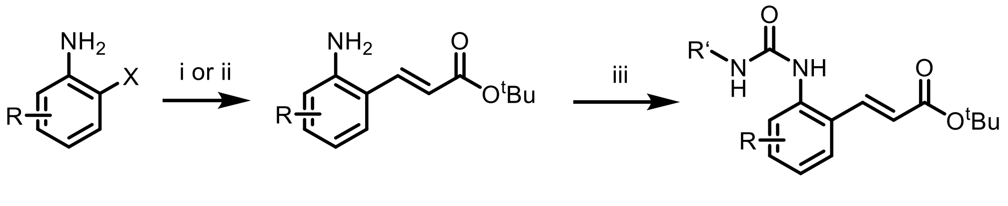

Scheme S3. Synthesis of starting materials **1a** to **1q**, **1s** to **1t** and **1z** to **1ae**. i. $PdCl_2(PPh_3)_2$, *tert*-butyl acrylate, triethylamine, 100 °C, 24h; ii. $Pd(OAc)_2$, *tert*-butyl acrylate, triethylamine, tris(*o*-tolyl)phosphine, acetonitrile, 120 °C, 24h; iii. Isocyanate, toluene, rt, 16h.

Aniline-ester

i. (for starting materials **1b** to **1i**) According to the modified literature procedure reported by M. M. Ahire *et. al.,* [6] *tert*-butyl acrylate (1.2 eq) was added to a solution of 2-halogen-aniline (1.0 eq) and $PdCl_2(PPh_3)_2$ (0.05 eq) in triethylamine (1.2M) in a sealed tube under N_2 at room temperature. The sealed tube was flushed twice with argon gas before sealed with a screw cap. The reaction mixture was heated to 100 °C and stirred for 24 hours. After cooled back to room temperature, the resulting mixture was diluted with H_2O (50 mL) and extracted with EtOAc (4 x 50 mL). The combined organic layers were washed with brine (100 mL), dried over Na_2SO_4, filtered and evaporated to dryness under reduced pressure. The resulting paste was flushed through silica gel with (pentane/Et_2O = 4/1) to afford the aniline-ester as a yellow solid which was used as crude for next step without any further purification.

ii. (for starting materials **1a**, **1j** to **1q**, **1s** to **1t** and **1z** to **1ae**) According to the modified literature procedure reported by Yamanaka *et. al.,* [7] *tert*-butyl acrylate (2.0 eq) and triethylamine (2.0 eq) were added to a solution of 2-halogen-aniline (1.0 eq), tris(*o*-tolyl)phosphine (0.06 eq) and $Pd(OAc)_2$ (0.03 eq) in acetonitrile (0.42M) in a sealed tube under N_2 at room temperature. The sealed tube was flushed twice with argon gas before sealed with a screw cap. The reaction mixture was heated to 120 °C and stirred for 24 hours. After cooled back to room temperature, the resulting mixture was diluted with H_2O (50 mL), made alkaline with K_2CO_3 and then extracted with $CHCl_3$ (4 x 50 mL). The combined organic layers were

washed with brine (100 mL), dried over Na_2SO_4, filtered and evaporated to dryness under reduced pressure. The resulting paste was flushed through silica gel with (CH_2Cl_2/MeOH = 95/5) to afford the aniline-ester as a yellow solid which was used as crude for next step without any further purification.

Starting materials 1a to 1q, 1s to 1t and 1z to 1ae. According to the modified literature procedure reported by S. J. Park *et. al.,* [8] the corresponding isocyanate (2.0 eq) was added to a solution of the aniline-ester (1.0 eq) in toluene (0.24M) under N_2 at room temperature. The reaction mixture was stirred for 20 hours, filtered and washed with pentane (2 x 100 mL) to afford starting materials **1a** to **1q**, **1s** to **1t** and **1z** to **1ae** as an off-white solid.

4.2 General Procedure B for Preparation of Starting Materials 1r and 1u to 1y

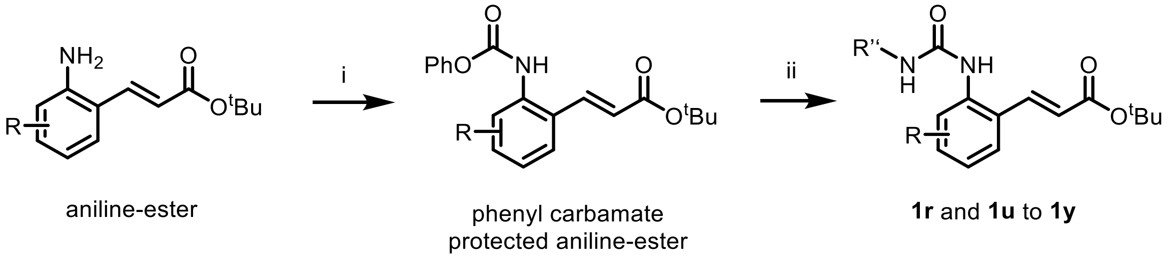

Scheme S4. Synthesis of starting materials **1r** and **1u** to **1y**. i. Phenyl chloroformate, Na_2HPO_4, EtOAc/H_2O, 12h at rt then 2h at 60 °C; ii. Amine, triethylamine, THF, reflux, 7h.

Phenyl carbamate aniline-ester. According to the modified literature procedure reported by G. R. Humphrey *et. al.,* [9] Na_2HPO_4 (1.5 eq) and H_2O (1.1M) were added to a solution of the aniline-ester (1.0 eq) in EtOAc (0.35M) at room temperature, followed by dropwise addition of phenyl chloroformate (1.25 eq). The reaction mixture was stirred for 12h at room temperature and then 2h at 60 °C. More EtOAc was added at 60 °C until all solids were dissolved and the layer was then separated. The organic layer was washed with H_2O (100 mL), dried over Na_2SO_4, filtered and evaporated to dryness under reduced pressure. The resulting paste was flushed through silica gel with (pentane/EtOAc = 9/1) to afford the phenyl carbamate protected aniline-ester as an orange solid which was used as crude for next step without any further purification.

Starting materials 1r and 1u to 1y. Amine (2.0 eq) was added to a solution of the phenyl carbamate protected aniline-ester (1.0 eq) and triethylamine (2.0 eq) in THF (0.5M) in a sealed tube under N_2 at room temperature. The sealed tube was flushed twice with argon gas before sealed with a screw cap. The reaction mixture was heated to 50 °C and stirred for 7 hours. After cooled back to room temperature, the resulting mixture was evaporated to dryness under reduced pressure. Purification by silica gel chromatography (EtOAc in pentane) afforded the starting materials **1r** and **1u** to **1y** as an off-white solid.

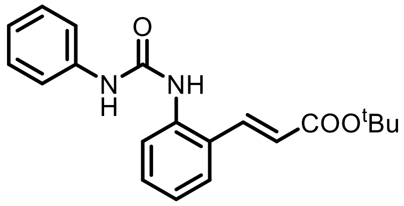

***tert*-Butyl (*E*)-3-(2-(3-phenylureido)phenyl)acrylate (1a)** was prepared following **General Procedure A**, using 2-iodoaniline (1.70 g, 7.75 mmol, 1.0 eq) and phenyl isocyanate (1.85 g, 15.50 mmol, 2.0 eq) to afford the title compound as an off-white powder (2.32 g, 89%).

^{1}H NMR (400 MHz, $(CD_3)_2SO$) δ 8.95 (s, 1H, NH), 8.46 (s, 1H, NH), 7.83 (d, *J* = 15.8 Hz, 1H, CH), 7.77 (dd, *J* = 8.3, 1.3 Hz, 1H, CH=CHAr-H), 7.73 (dd, *J* = 7.9, 1.6 Hz, 1H, CH=CHAr-H), 7.52 – 7.44 (m, 2H, Ar-H), 7.38 (ddd, *J* = 8.3, 7.3, 1.6 Hz, 1H, CH=CHAr-H), 7.33 – 7.25 (m, 2H, Ar-H), 7.11 (tdd, *J* = 7.3, 1.3, 0.6 Hz, 1H, CH=CHAr-H), 6.98 (tt, *J* = 7.3, 1.3 Hz, 1H, Ar-H), 6.47 (d, *J* = 15.8 Hz, 1H, CH), 1.49 (s, 9H, $OC(CH_3)_3$) ppm.

^{13}C NMR (101 MHz, $(CD_3)_2SO$) δ 165.6 (C(O)O), 152.7 (C(O)NH), 139.6 (ArC), 138.9 (CH), 137.7 (CH=CHArC), 130.4 (CH=CHArCH), 128.75 (ArCH), 127.0 (CH=CHArC), 126.1 (CH=CHArCH), 123.8 (CH=CHArCH), 123.7 (CH=CHArCH), 121.9 (ArCH), 120.7 (CH), 118.2 (ArCH), 80.0 ($OC(CH_3)_3$), 27.8 ($OC(CH_3)_3$) ppm.

HRMS (ESI) m/z calcd. for $C_{20}H_{22}N_2O_3{}^{23}Na$ ($[M+Na]^+$) 361.15226, found 361.15231

FT-IR (thin film) ν_{max} 3319, 2978, 2160, 2027, 1707, 1650, 1600, 1581, 1553, 1499, 1444, 1392, 1367, 1320, 1235, 1150, 981, 873, 752, 693 cm^{-1}.

m.p.: 181-184 °C

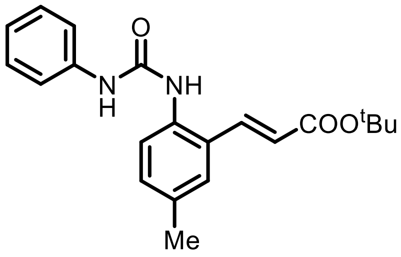

***tert*-Butyl (*E*)-3-(5-methyl-2-(3-phenylureido)phenyl)acrylate (1b)** was prepared following **General Procedure A**, using 2-bromo-4-methylaniline (1.86 g, 10.00 mmol, 1.0 eq) and phenyl isocyanate (2.39 g, 20.00 mmol, 2.0 eq) to afford the title compound as a white powder (1.03 g, 76%).

^{1}H NMR (400 MHz, $CDCl_3$) δ 7.86 (d, *J* = 15.9 Hz, 1H, C<u>H</u>), 7.50 (d, *J* = 8.2 Hz, 1H, Ar-<u>H</u>), 7.45 – 7.30 (m, 3H, N<u>H</u> and Ar-<u>H</u>), 7.22 – 7.19 (m, 4H, Ar-<u>H</u>), 7.15 – 7.08 (m, 1H, Ar-<u>H</u>), 7.01 (qd, *J* = 4.6, 3.7 Hz, 1H, Ar-<u>H</u>), 6.31 (d, *J* = 15.9 Hz, 1H, C<u>H</u>), 2.28 (s, 3H, C<u>H</u>$_3$), 1.45 (s, 9H, OC(C<u>H</u>$_3$)$_3$) ppm.

^{13}C NMR (101 MHz, $CDCl_3$) δ 166.7 (<u>C</u>(O)OC(CH$_3$)$_3$), 154.4 (<u>C</u>(O)NH), 138.8 (<u>C</u>H), 138.3 (Ar<u>C</u>), 135.5 (Ar<u>C</u>), 134.1 (Ar<u>C</u>), 131.8 (Ar<u>C</u>H), 129.1 (Ar<u>C</u>H), 128.7 (Ar<u>C</u>), 127.5 (Ar<u>C</u>H), 126.0 (Ar<u>C</u>H), 123.9 (Ar<u>C</u>H), 121.8 (<u>C</u>H), 121.1 (Ar<u>C</u>H), 81.0 (O<u>C</u>(CH$_3$)$_3$), 28.3 (OC(<u>C</u>H$_3$)$_3$), 21.0 (<u>C</u>H$_3$) ppm.

HRMS (ESI) m/z calcd. for $C_{21}H_{24}N_2O_3{}^{23}Na$ ($[M+Na]^+$) 375.1679, found 375.1680

FT-IR (thin film) ν_{max} 3323, 2977, 2160, 2032, 1707, 1648, 1598, 1553, 1498, 1443, 1392, 1367, 1317, 1240, 1151, 981 864, 752, 693 cm^{-1}.

m.p.: 201-202 ^{O}C

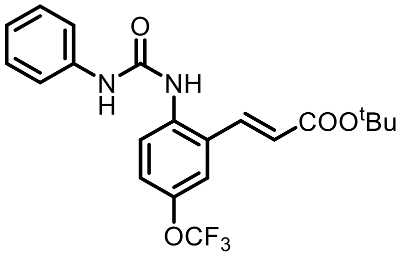

***tert*-Butyl (*E*)-3-(2-(3-phenylureido)-5-(trifluoromethoxy)phenyl)acrylate (1c)** was prepared following **General Procedure A**, using 2-bromo-4-(trifluoromethoxy)aniline (0.72 g, 2.80 mmol, 1.0 eq) and phenyl isocyanate (0.58 g, 4.88 mmol, 2.00 eq) to afford the title compound as a white powder (0.84 g, 72%).

^{1}H NMR (400 MHz, CDCl$_3$) δ 7.81 – 7.72 (m, 2H, C$\underline{H}$ and N$\underline{H}$), 7.71 – 7.64 (m, 2H, N$\underline{H}$ and Ar-$\underline{H}$), 7.32 – 7.28 (m, 1H, Ar-$\underline{H}$), 7.20 – 7.12 (m, 4H, Ar-$\underline{H}$), 7.10 (dq, *J* = 8.9, 1.1 Hz, 1H, Ar-$\underline{H}$), 7.00 (ddt, *J* = 7.4, 6.4, 1.7 Hz, 1H, Ar-$\underline{H}$), 6.26 (d, *J* = 15.8 Hz, 1H, C$\underline{H}$), 1.43 (s, 9H, OC(C$\underline{H}_3$)$_3$) ppm.

^{13}C NMR (101 MHz, CDCl$_3$) δ 166.2 ($\underline{C}$(O)OC(CH$_3$)$_3$), 154.3 ($\underline{C}$(O)NH), 146.1 (Ar$\underline{C}$), 137.8 (Ar$\underline{C}$), 137.3 ($\underline{C}$H), 135.3 (Ar$\underline{C}$), 129.5 (Ar$\underline{C}$), 129.2 (Ar$\underline{C}$H), 126.7 (Ar$\underline{C}$H), 124.4 (Ar$\underline{C}$H), 123.3 ($\underline{C}$H), 123.2 (Ar$\underline{C}$H), 121.8 ($\underline{C}$F$_3$), 121.4 (Ar$\underline{C}$H), 119.3 (Ar$\underline{C}$H), 81.5 (O$\underline{C}$(CH$_3$)$_3$), 28.2 (OC($\underline{C}$H$_3$)$_3$) ppm. (C-F coupling not observed)

^{19}F NMR (377 MHz, CDCl$_3$) δ -58.07 (s) ppm.

HRMS (ESI) m/z calcd. for $C_{21}H_{21}F_3N_2O_4{}^{23}Na$ ([M+Na]$^+$) 445.1346, found 445.1346

FT-IR (thin film) ν_{max} 3689, 3308, 2980, 2519, 2362, 2160, 2028, 1978, 1711, 1652, 1601, 1557, 1498, 1445, 1369, 1324, 1259, 1221, 1154, 977, 752, 693, 625 cm^{-1}.

m.p.: 192-193 ^{O}C

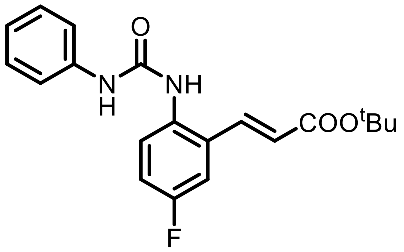

tert**-Butyl (*****E*****)-3-(5-fluoro-2-(3-phenylureido)phenyl)acrylate (1d)** was prepared following **General Procedure A**, using 2-bromo-4-fluoroaniline (0.67 g, 3.53 mmol, 1.0 eq) and phenyl isocyanate (0.72, 6.00 mmol, 2.0 eq) to afford the title compound as an off-white powder (1.00 g, 80%).

^{1}H NMR (400 MHz, $CDCl_3$) δ 7.73 (dd, *J* = 15.8, 1.6 Hz, 1H, ArCH=CH), 7.64 (brs, 2H, NH), 7.46 (dd, *J* = 8.9, 5.3 Hz, 1H, Ar-H), 7.14 (qt, *J* = 8.5, 3.4 Hz, 5H, Ar-H), 7.03 – 6.95 (m, 1H, Ar-H), 6.91 (ddd, *J* = 8.9, 7.7, 2.9 Hz, 1H, Ar-H), 6.22 (d, *J* = 15.8 Hz, 1H, ArCH=CH), 1.44 (s, 9H, $OC(CH_3)_3$) ppm.

^{13}C NMR (101 MHz, $CDCl_3$) δ 166.2 ($C(O)OC(CH_3)_3$), 160.3 (d, *J* = 245.6 Hz, CF), 154.8 (C(O)NH), 138.0 (ArC), 137.6 (d, *J* = 2.2 Hz, ArCH=CH), 132.6 (d, *J* = 2.7 Hz, CH=CHArC), 130.9 (d, *J* = 7.8 Hz, CH=CHArC), 129.1 (ArCH), 128.1 (d, *J* = 8.3 Hz, CH=CHArCH), 124.1 (ArCH), 122.9 (ArCH=CH), 121.2 (ArCH), 117.7 (d, *J* = 22.7 Hz, CH=CHArCH), 113.0 (d, *J* = 23.4 Hz, CH=CHArCH), 81.2 ($OC(CH_3)_3$), 28.2 ($OC(CH_3)_3$) ppm.

^{19}F NMR (377 MHz, $CDCl_3$) δ -116.17 (s) ppm. (H-F coupling not observed)

HRMS (ESI) m/z calcd. for $C_{20}H_{21}FN_2O_3{}^{23}Na$ ($[M+Na]^+$) 379.1428, found 379.1429

FT-IR (thin film) ν_{max} 3310, 2519, 2364, 2160, 2029, 1977, 1709, 1648, 1599, 1556, 1491, 1444, 1368, 1319, 1245, 1154, 980, 860, 752, 693 cm^{-1}.

m.p.: 177-179 ^{O}C

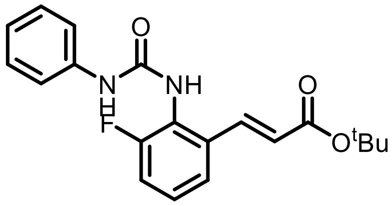

***tert*-Butyl (*E*)-3-(3-fluoro-2-(3-phenylureido)phenyl)acrylate (1e)** was prepared following **General Procedure A**, using 2-bromo-6-fluoroaniline (0.40 g, 2.10 mmol, 1.0 eq) and phenyl isocyanate (0.50 g, 4.20 mmol, 2.0 eq) to afford the title compound as an off-white powder (0.40 g, 50%).

^{1}H NMR (400 MHz, CDCl$_3$) δ 7.83 (d, *J* = 16.0 Hz, 1H, CH), 7.55 – 7.38 (m, 2H, Ar-H), 7.35 – 7.27 (m, 3H, Ar-H), 7.25 – 7.19 (m, 1H, Ar-H), 7.18 – 7.10 (m, 1H, Ar-H), 7.10 – 7.02 (m, 1H, Ar-H), 6.91 (s, 1H, NH), 6.54 (s, 1H, NH), 6.40 (d, *J* = 16.0 Hz, 1H, CH), 1.49 (s, 9H, OC(CH$_3$)$_3$) ppm.

^{19}F NMR (377 MHz, CDCl$_3$) δ -120.45 (t, *J* = 7.8 Hz) ppm.

HRMS (ESI) m/z calcd. for $C_{20}H_{21}FN_2O_3{}^{23}Na$ ([M+Na]$^+$) 379.1428, found 379.1428

FT-IR (thin film) ν_{max} 3310, 2529, 2160, 2031, 1977, 1709, 1651, 1600, 1556, 1499, 1476, 1444, 1368, 1321, 1252, 1153, 984, 753, 693, 620 cm^{-1}.

m.p.: 209-210 ^{O}C

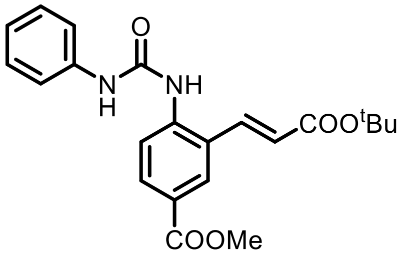

Methyl (*E*)-3-(3-(*tert*-butoxy)-3-oxoprop-1-en-1-yl)-4-(3-phenylureido)benzoate (1f) was prepared following **General Procedure A**, using methyl 4-amino-3-bromobenzoate (0.53 g, 2.29 mmol, 1.0 eq) and phenyl isocyanate (0.44 g, 3.70 mmol, 2.0 eq) to afford the title compound as an off-white powder (0.37 g, 41%).

^{1}H NMR (400 MHz, $CDCl_3$) δ 8.16 (d, *J* = 2.0 Hz, 1H, CH=CHAr-H), 8.03 (d, *J* = 8.6 Hz, 1H, CH=CHAr-H), 7.95 (dd, *J* = 8.6, 2.0 Hz, 1H, CH=CHAr-H), 7.82 (d, *J* = 15.7 Hz, 1H, CH), 7.67 (s, 1H, NH), 7.64 (s, 1H, NH), 7.28 – 7.25 (m, 4H, Ar-H), 7.08 (ddd, *J* = 5.6, 4.4, 2.7 Hz, 1H, Ar-H), 6.41 (d, *J* = 15.7 Hz, 1H, CH), 3.91 (s, 3H, OCH_3), 1.48 (s, 9H, $OC(CH_3)_3$) ppm.

^{13}C NMR (101 MHz, $CDCl_3$) δ 166.6 ($C(O)OCH_3$), 166.4 ($C(O)OC(CH_3)_3$), 153.3 (C(O)NH), 141.0 (CH=CHArC), 137.8 (CH), 137.7 (ArC), 131.9 (CH=CHArCH), 129.4 (ArCH), 129.1 (CH=CHArCH), 126.0 (CH=CHArC), 125.8 (CH=CHArC), 124.6 (ArCH), 123.6 (CH), 122.9 (CH=CHArCH), 121.5 (ArCH), 81.5 ($OC(CH_3)_3$), 52.3 (OCH_3), 28.3 ($OC(CH_3)_3$) ppm.

HRMS (ESI) m/z calcd. for $C_{22}H_{24}N_2O_5{}^{23}Na$ ($[M+Na]^+$) 419.1577, found 419.1577

FT-IR (thin film) ν_{max} 3345, 2979, 2518, 2364, 2160, 2030, 1977, 1711, 1655, 1602, 1585, 1552, 1499, 1438, 1368, 1289, 1252, 1196, 1152, 982, 849, 758, 693, 626 cm^{-1}.

m.p.: 187-189 ^{O}C

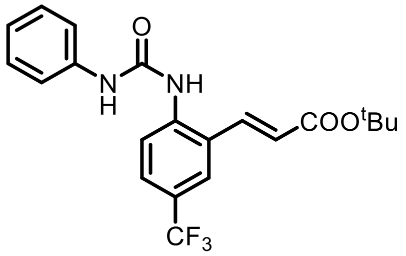

***tert*-Butyl (*E*)-3-(2-(3-phenylureido)-5-(trifluoromethyl)phenyl)acrylate (1g)** was prepared following **General Procedure A**, using 2-iodo-4-(trifluoromethyl)aniline (0.70 g, 2.43 mmol, 1.0 eq) and phenyl isocyanate (0.58 g, 4.86 mmol, 2.00 eq) to afford the title compound as a white powder (0.49 g, 50%).

^{1}H NMR (400 MHz, CDCl$_3$) δ 8.02 (d, *J* = 8.6 Hz, 1H, CH=CHAr-H), 7.79 (d, *J* = 15.8 Hz, 1H, CH), 7.72 (brs, 1H, NH), 7.70 (brs, 1H, NH), 7.68 (d, *J* = 1.6 Hz, 1H, CH=CHAr-H), 7.50 (dd, *J* = 8.6, 2.1 Hz, 1H, CH=CHAr-H), 7.25 – 7.19 (m, 4H, Ar-H), 7.10 – 7.01 (m, 1H, Ar-H), 6.34 (d, *J* = 15.8 Hz, 1H, CH), 1.46 (s, 9H, OC(CH$_3$)$_3$) ppm.

^{13}C NMR (101 MHz, CDCl$_3$) δ δ 166.4 (C(O)OC(CH$_3$)$_3$), 153.5 (C(O)NH), 139.8 (ArC), 137.6 (ArC), 137.5 (CH), 129.4 (ArCH), 127.4 (q, *J* = 3.3 Hz, ArCH), 126.6 (ArC), 125.8 (q, *J* = 103.8 Hz, ArC), 125.7 (q, *J* = 198.2 Hz, ArCF$_3$), 124.7 (ArCH), 124.3 (q, *J* = 3.9 Hz, ArCH), 123.9 (CH), 123.7 (ArCH), 121.5 (ArCH), 81.7 (OC(CH$_3$)$_3$), 28.2 (OC(CH$_3$)$_3$) ppm.

^{19}F NMR (377 MHz, CDCl$_3$) δ -62.40 (s) ppm.

HRMS (ESI) m/z calcd. for $C_{21}H_{20}F_3N_2O_3$ ([M - H]$^+$) 405.1432, found 405.1426

FT-IR (thin film) ν_{max} 3020, 2360, 2161, 2032, 1699, 1593, 1541, 1499, 1445, 1370, 1322, 1293, 1215, 1153, 1127, 1080, 980, 908, 844, 747, 668 cm^{-1}.

m.p.: 203-205 ^{O}C

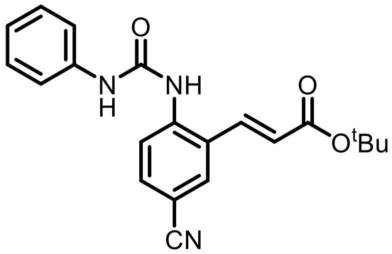

***tert*-Butyl (*E*)-3-(5-cyano-2-(3-phenylureido)phenyl)acrylate (1h)** was prepared following **General Procedure A**, using 4-amino-3-iodobenzonitrile (0.81 g, 3.31 mmol, 1.0 eq) and phenyl isocyanate (0.79 g, 6.62 mmol, 2.0 eq) to afford the title compound as a white powder (0.48 g, 40%).

^{1}H NMR (400 MHz, CDCl$_3$) δ 8.12 (d, *J* = 8.6 Hz, 1H, Ar-H), 7.95 (s, 1H, NH), 7.88 (s, 1H, NH), 7.74 (d, *J* = 15.7 Hz, 1H, CH), 7.70 (d, *J* = 1.9 Hz, 1H, Ar-H), 7.51 (dd, *J* = 8.6, 1.9 Hz, 1H, Ar-H), 7.37 – 7.28 (m, 1H, Ar-H), 7.25 – 7.20 (m, 3H, Ar-H), 7.13 – 7.05 (m, 1H, Ar-H), 6.31 (d, *J* = 15.7 Hz, 1H, CH), 1.49 (s, 9H, OC(CH_3)$_3$) ppm.

^{13}C NMR (101 MHz, CDCl$_3$) δ 166.2 (C(O)OC(CH$_3$)$_3$), 152.9 (C(O)NH), 141.1 (ArC), 137.5 (ArC), 136.6 (CH), 133.9 (ArCH), 131.4 (ArCH), 129.5 (ArCH), 129.3 (ArC), 126.2 (ArCH), 125.0 (CH), 122.8 (ArCH), 121.4 (ArCH), 118.5 (ArCN), 107.2 (ArC), 82.0 (OC(CH$_3$)$_3$), 28.2 (OC(CH$_3$)$_3$) ppm.

HRMS (ESI) m/z calcd. for $C_{21}H_{21}N_3O_3{}^{23}Na$ ([M+Na]$^+$) 386.1475, found 386.1475

FT-IR (thin film) ν_{max} 3649, 3340, 2980, 2360, 2228, 2161, 2033, 1710, 1659, 1607, 1583, 1557, 1500, 1445, 1393, 1369, 1299, 1239, 1153, 979, 851, 754, 694 cm^{-1}.

m.p.: 146-150 ^{O}C

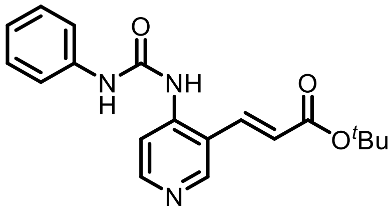

***tert*-Butyl (*E*)-3-(4-(3-phenylureido)pyridin-3-yl)acrylate (1i)** was prepared following **General Procedure A**, using 3-bromopyridin-4-amine (0.76 g, 4.40 mmol, 1.0 eq) and phenyl isocyanate (1.05 g, 8.80 mmol, 2.0 eq). Purification by silica gel chromatography (50% EtOAc in CH_2Cl_2) afforded the title compound as a white powder (0.29 g, 19%).

^{1}H NMR (400 MHz, $CDCl_3$) δ 8.57 (s, 1H, Ar(N)-H), 8.43 (d, *J* = 5.8 Hz, 1H, Ar(N)-H), 8.17 (d, *J* = 5.8 Hz, 1H, Ar(N)-H), 7.84 (s, 1H, NH), 7.78 (s, 1H, NH), 7.66 (d, *J* = 15.8 Hz, 1H, CH), 7.35 – 7.27 (m, 4H, Ar-H), 7.16 – 7.06 (m, 1H, Ar-H), 6.34 (d, *J* = 15.8 Hz, 1H, CH), 1.48 (s, 9H, $OC(CH_3)_3$) ppm.

^{13}C NMR (101 MHz, $CDCl_3$) δ 166.2 ($C(O)OC(CH_3)_3$), 152.4 (C(O)NH), 151.4 (ArCH), 148.9 (ArCH), 144.4 (ArC), 137.6 (ArC), 135.9 (CH), 129.5 (ArCH), 124.9 (ArCH), 124.4 (CH), 121.2 (ArCH), 120.2 (ArC), 114.6 (ArCH), 81.9 ($OC(CH_3)_3$), 28.3 ($OC(CH_3)_3$) ppm.

HRMS (ESI) m/z calcd. for $C_{19}H_{22}N_3O_3$ ($[M+H]^+$) 340.1656, found 340.1655

FT-IR (thin film) ν_{max} 3323, 2979, 2360, 2160, 2030, 1707, 1636, 1601, 1579, 1556, 1516, 1498, 1445, 1419, 1393, 1368, 1314, 1288, 1247, 1199, 1149, 1067, 1025, 979, 871, 841, 752, 692, 646 cm^{-1}.

m.p.: 167-170 ^{O}C

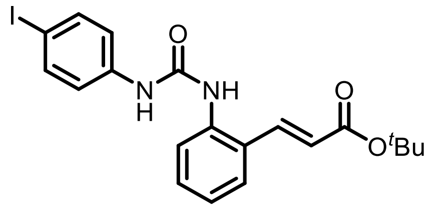

***tert*-Butyl (*E*)-3-(2-(3-(4-iodophenyl)ureido)phenyl)acrylate (1j)** was prepared following **General Procedure A**, using 2-iodoaniline (0.42 g, 1.90 mmol, 1.0 eq) and 1-iodo-4-isocyanatobenzene (0.93 g, 3.80 mmol, 2.0 eq) to afford the title compound as a white powder (0.64 g, 73%).

^{1}H NMR (400 MHz, $CDCl_3$) δ 7.87 (d, *J* = 15.6 Hz, 1H, CH), 7.64 (d, *J* = 7.6 Hz, 1H, Ar-H), 7.58 (brs, 1H, NH), 7.55 (dd, *J* = 8.0, 1.5 Hz, 1H, Ar-H), 7.47 (d, *J* = 7.9 Hz, 2H, Ar-H), 7.46 (brs, 1H, NH), 7.33 (td, *J* = 7.3, 1.5 Hz, 1H, Ar-H), 7.15 (t, *J* = 7.6 Hz, 1H, Ar-H), 6.96 (d, *J* = 7.9 Hz, 2H, Ar-H), 6.33 (d, *J* = 15.6 Hz, 1H, CH), 1.45 (s, 9H, $OC(CH_3)_3$) ppm.

^{13}C NMR (101 MHz, $CDCl_3$) δ 166.8 ($C(O)OC(CH_3)_3$), 153.9 (C(O)NH), 138.7 (CH), 138.06 (ArC), 138.03 (ArCH), 136.5 (ArC), 131.0 (ArCH), 128.5 (ArC), 127.2 (ArCH), 125.8 (ArCH), 125.6 (ArCH), 122.5 (ArCH), 122.1 (CH), 87.1 (ArC), 81.3 ($OC(CH_3)_3$), 28.3 ($OC(CH_3)_3$) ppm.

HRMS (ESI) m/z calcd. for $C_{20}H_{21}{}^{127}IN_2O_3{}^{23}Na$ ($[M+Na]^+$) 487.0489, found 487.0490

FT-IR (thin film) ν_{max} 3853, 3734, 3649, 3295, 2360, 2341, 2161, 2031, 1977, 1705, 1649, 1584, 1542, 1486, 1457, 1392, 1367, 1321, 1235, 1151, 982, 770, 669 cm^{-1}.

m.p.: 216-217 ^{O}C

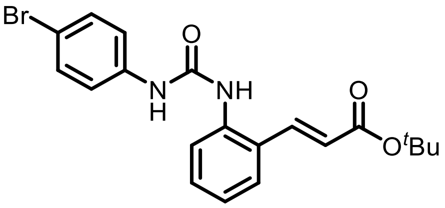

***tert*-Butyl (*E*)-3-(2-(3-(4-bromophenyl)ureido)phenyl)acrylate (1k)** was prepared following **General Procedure A**, using 2-iodoaniline (0.47 g, 2.15 mmol, 1.0 eq) and 1-bromo-4-isocyanatobenzene (0.85 g, 4.30 mmol, 2.0 eq) to afford the title compound as a white powder (0.65 g, 72%).

^{1}H NMR (400 MHz, $CDCl_3$) δ 7.85 (d, *J* = 15.8 Hz, 1H, C<u>H</u>), 7.69 (brs, 1H, N<u>H</u>), 7.63 – 7.57 (m, 2H, N<u>H</u> and Ar-<u>H</u>), 7.53 (dd, *J* = 8.0, 1.5 Hz, 1H, Ar-<u>H</u>), 7.32 – 7.27 (m, 1H, Ar-<u>H</u>), 7.27 – 7.23 (m, 2H, Ar-<u>H</u>), 7.12 (t, *J* = 7.6 Hz, 1H, Ar-<u>H</u>), 7.07 – 6.99 (m, 2H, Ar-<u>H</u>), 6.31 (d, *J* = 15.8 Hz, 1H, C<u>H</u>), 1.45 (s, 9H, $OC(C\underline{H}_3)_3$) ppm.

^{13}C NMR (101 MHz, $CDCl_3$) δ 166.8 ($\underline{C}(O)OC(CH_3)_3$), 154.1 (<u>C</u>(O)NH), 138.7 (<u>C</u>H), 137.3 (Ar<u>C</u>), 136.4 (Ar<u>C</u>), 132.0 (Ar<u>C</u>H), 131.0 (Ar<u>C</u>H), 128.5 (Ar<u>C</u>), 127.2 (Ar<u>C</u>H), 125.8 (Ar<u>C</u>H), 125.7 (Ar<u>C</u>H), 122.3 (Ar<u>C</u>H), 122.0 (<u>C</u>H), 116.6 (Ar<u>C</u>), 81.2 ($O\underline{C}(CH_3)_3$), 28.3 ($OC(\underline{C}H_3)_3$) ppm.

HRMS (ESI) m/z calcd. for $C_{20}H_{21}{}^{80}BrN_2O_3{}^{23}Na$ ($[M+Na]^+$) 439.0628, found 439.0628

FT-IR (thin film) ν_{max} 3323, 2361, 2161, 2031, 1706, 1650, 1591, 1544, 1488, 1456, 1395, 1367, 1321, 1235, 1151, 1073, 1009, 982, 871, 757, 668 cm^{-1}.

m.p.: 199-200 ^{O}C

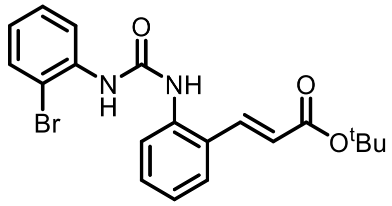

***tert*-Butyl (*E*)-3-(2-(3-(2-bromophenyl)ureido)phenyl)acrylate (1l)** was prepared following **General Procedure A**, using 2-iodoaniline (0.52 g, 2.38 mmol, 1.0 eq) and 1-bromo-2-isocyanatobenzene (0.94 g, 4.76 mmol, 2.0 eq). Purification by silica gel chromatography (10% EtOAc in pentane) afforded the title compound as an off-white powder (0.86 g, 87%).

^{1}H NMR (400 MHz, CDCl$_3$) δ 8.20 (s, 1H, N<u>H</u>), 8.04 (dd, *J* = 8.2, 1.6 Hz, 1H, Ar-<u>H</u>), 7.95 (d, *J* = 15.8 Hz, 1H, C<u>H</u>), 7.66 (dd, *J* = 8.2, 1.2 Hz, 1H, Ar-<u>H</u>), 7.55 – 7.48 (m, 2H, Ar-<u>H</u> and N<u>H</u>), 7.35 (dd, *J* = 8.0, 1.5 Hz, 1H, Ar-<u>H</u>), 7.30 (td, *J* = 7.7, 1.5 Hz, 1H, Ar-<u>H</u>), 7.13 (dddd, *J* = 9.5, 6.4, 3.8, 1.8 Hz, 2H, Ar-<u>H</u>), 6.78 (ddd, *J* = 7.7, 1.6 Hz, 1H, Ar-<u>H</u>), 6.30 (d, *J* = 15.8 Hz, 1H, C<u>H</u>), 1.44 (s, 9H, OC(C<u>H</u>$_3$)$_3$) ppm.

^{13}C NMR (101 MHz, CDCl$_3$) δ 166.7 (<u>C</u>(O)OC(CH$_3$)$_3$), 153.5 (<u>C</u>(O)NH), 138.9 (<u>C</u>H), 136.4 (Ar<u>C</u>), 136.3 (Ar<u>C</u>), 132.4 (Ar<u>C</u>H), 131.0 (Ar<u>C</u>H), 129.3 (Ar<u>C</u>), 128.3 (Ar<u>C</u>H), 127.4 (Ar<u>C</u>H), 126.2 (Ar<u>C</u>H), 126.1 (Ar<u>C</u>H), 124.5 (Ar<u>C</u>H), 122.45 (<u>C</u>H), 122.40 (Ar<u>C</u>H), 114.1 (Ar<u>C</u>), 81.2 (O<u>C</u>(CH$_3$)$_3$), 28.3 (OC(<u>C</u>H$_3$)$_3$) ppm.

HRMS (ESI) m/z calcd. for $C_{20}H_{21}BrN_2O_3{}^{23}Na$ ([M+Na]$^+$) 439.0628, found 439.0628

FT-IR (thin film) ν_{max} 3297, 2977, 2161 2024, 1707, 1650, 1584, 1551, 1477, 1455, 1437, 1391, 1367, 1322, 1297, 1259, 1233, 1151, 1027, 980, 875, 752, 668 cm^{-1}.

m.p.: 87-92 ^{O}C

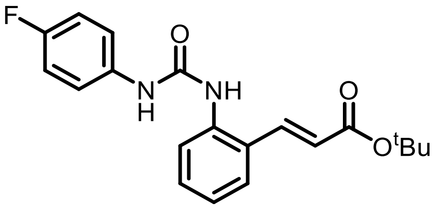

***tert*-Butyl (*E*)-3-(2-(3-(4-fluorophenyl)ureido)phenyl)acrylate (1m)** was prepared following **General Procedure A**, using 2-iodoaniline (0.33 g, 1.50 mmol, 1.0 eq) and 1-fluoro-4-isocyanatobenzene (0.42 g, 3.00 mmol, 2.0 eq) to afford the title compound as a white powder (0.33 g, 61%).

^{1}H NMR (400 MHz, CDCl$_3$) δ 7.86 (d, *J* = 15.8 Hz, 1H, C<u>H</u>), 7.65 (dd, *J* = 8.2, 1.2 Hz, 1H, Ar-<u>H</u>), 7.61 – 7.47 (brs, 2H, N<u>H</u>), 7.53 (dd, *J* = 8.2, 1.2 Hz, 1H, Ar-<u>H</u>), 7.33 – 7.27 (m, 1H, Ar-<u>H</u>), 7.21 – 7.07 (m, 3H, Ar-<u>H</u>), 6.93 – 6.82 (m, 2H, Ar-<u>H</u>), 6.31 (d, *J* = 15.8 Hz, 1H, C<u>H</u>), 1.45 (s, 9H, OC(C<u>H</u>$_3$)$_3$) ppm.

^{13}C NMR (101 MHz, CDCl$_3$) δ 166.7 (<u>C</u>(O)OC(CH$_3$)$_3$), 159.6 (d, *J* = 243.4 Hz, Ar<u>C</u>F), 154.3 (<u>C</u>(O)NH), 138.6 (<u>C</u>H), 138.0 (Ar<u>C</u>), 134.0 (d, *J* = 2.7 Hz, Ar<u>C</u>), 131.0 (Ar<u>C</u>H), 128.3 (Ar<u>C</u>), 127.2 (Ar<u>C</u>H), 125.6 (Ar<u>C</u>H), 125.5 (Ar<u>C</u>H), 123.2 (d, *J* = 8.0 Hz, Ar<u>C</u>H), 122.1 (<u>C</u>H), 115.8 (d, *J* = 22.5 Hz, Ar<u>C</u>H), 81.2 (O<u>C</u>(CH$_3$)$_3$), 28.2 (OC(<u>C</u>H$_3$)$_3$) ppm.

^{19}F NMR (377 MHz, CDCl$_3$) δ -118.58 (s) ppm. (H-F coupling not observed)

HRMS (ESI) m/z calcd. for $C_{20}H_{21}FN_2O_3{}^{23}Na$ ([M+Na]$^+$) 379.1428, found 379.1430

FT-IR (thin film) ν_{max} 3309, 2980, 2551, 2160, 2032, 1977, 1707, 1648, 1558, 1509, 1455, 1408, 1368, 1322, 1213, 1152, 982, 835, 760, 619 cm^{-1}.

m.p.: 116-118 ^{O}C

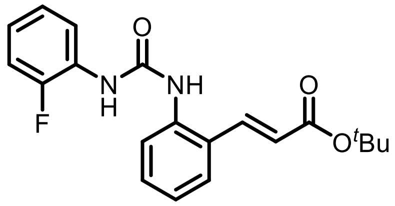

***tert*-Butyl (*E*)-3-(2-(3-(2-fluorophenyl)ureido)phenyl)acrylate (1n)** was prepared following **General Procedure A**, using 2-iodoaniline (0.50 g, 2.25 mmol, 1.0 eq) and 1-fluoro-2-isocyanatobenzene (0.62 g, 4.50 mmol, 2.0 eq) to afford the title compound as an off-white powder (0.65 g, 81%).

^{1}H NMR (400 MHz, $CDCl_3$) δ 8.06 (td, *J* = 8.1, 1.5 Hz, 1H, Ar-H), 7.94 (d, *J* = 15.8 Hz, 1H, CH), 7.79 (s, 1H, NH), 7.75 (dd, *J* = 8.2, 1.2 Hz, 1H, Ar-H), 7.52 (dd, *J* = 7.9, 1.5 Hz, 1H, Ar-H), 7.48 (d, *J* = 2.9 Hz, 1H, NH), 7.34 (ddd, *J* = 8.2, 7.5, 1.5 Hz, 1H, Ar-H), 7.13 (td, *J* = 7.5, 1.2 Hz, 1H, Ar-H), 7.05 – 6.99 (m, 1H, Ar-H), 6.99 – 6.93 (m, 1H, Ar-H), 6.93 – 6.87 (m, 1H, Ar-H), 6.33 (d, *J* = 15.8 Hz, 1H, CH), 1.45 (s, 9H, $OC(CH_3)_3$) ppm.

^{13}C NMR (101 MHz, $CDCl_3$) δ 167.0 ($C(O)OC(CH_3)_3$), 153.5 (C(O)NH), 153.1 (d, *J* = 244.1 Hz, ArCF), 139.1 (CH), 136.6 (ArC), 130.9 (ArCH), 128.3 (ArC), 127.3 (ArCH), 126.9 (d, *J* = 10.3 Hz, ArC), 125.4 (ArCH), 125.2 (ArCH), 124.5 (d, *J* = 3.6 Hz, ArCH), 123.6 (d, *J* = 7.4 Hz, ArCH), 122.2 (CH), 122.1 (ArCH), 114.9 (d, *J* = 19.4 Hz, ArCH), 81.3 ($OC(CH_3)_3$), 28.3 ($OC(CH_3)_3$) ppm.

^{19}F NMR (377 MHz, $CDCl_3$) δ -130.44 (s) ppm. (H-F coupling not observed)

HRMS (ESI) m/z calcd. for $C_{20}H_{21}FN_2O_3{}^{23}Na$ ($[M+Na]^+$) 379.1428, found 379.1428

FT-IR (thin film) ν_{max} 3326, 2979, 2160, 2032, 1707, 1655, 1620, 1583, 1546, 1488, 1456, 1392, 1368, 1322, 1250, 1186, 1150 1098, 1034, 982, 872, 753, 668 cm^{-1}.

m.p.: 163-164 ^{O}C

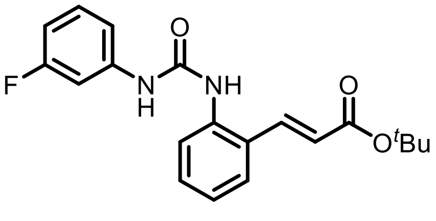

tert**-Butyl (*****E*****)-3-(2-(3-(3-fluorophenyl)ureido)phenyl)acrylate (1o)** was prepared following **General Procedure A**, using 2-iodoaniline (0.47 g, 2.12 mmol, 1.0 eq) and 1-fluoro-3-isocyanatobenzene (0.58 g, 4.24 mmol, 2.0 eq) to afford the title compound as an off-white powder (0.51 g, 67%).

^{1}H NMR (400 MHz, $CDCl_3$) δ 7.86 (d, *J* = 15.8 Hz, 1H, CH), 7.74 (s, 1H, NH), 7.71 (s, 1H, NH), 7.61 (dd, *J* = 8.1, 1.2 Hz, 1H, Ar-H), 7.51 (dd, *J* = 7.8, 1.6 Hz, 1H, Ar-H), 7.32 – 7.27 (m, 1H, Ar-H), 7.14 – 7.07 (m, 2H, Ar-H), 7.06 – 7.00 (m, 1H, Ar-H), 6.87 (ddd, *J* = 8.0, 2.0, 0.9 Hz, 1H, Ar-H), 6.65 (tdd, *J* = 8.3, 2.6, 0.9 Hz, 1H, Ar-H), 6.31 (d, *J* = 15.8 Hz, 1H, CH), 1.44 (s, 9H, $OC(CH_3)_3$) ppm.

^{13}C NMR (101 MHz, $CDCl_3$) δ 166.9 ($C(O)OC(CH_3)_3$), 163.1 (d, *J* = 244.8 Hz, ArCF), 154.1 (C(O)NH), 139.9 (d, *J* = 10.9 Hz, ArC), 138.7 (CH), 136.4 (ArC), 130.9 (ArCH), 130.1 (d, *J* = 9.5 Hz, ArCH), 128.5 (ArC), 127.1 (ArCH), 125.73 (ArCH), 125.71 (ArCH), 121.9 (CH), 115.8 (d, *J* = 2.8 Hz, ArCH), 110.4 (d, *J* = 21.2 Hz, ArCH), 107.8 (d, *J* = 25.5 Hz, ArCH), 81.2 ($OC(CH_3)_3$), 28.2 ($OC(CH_3)_3$) ppm.

^{19}F NMR (377 MHz, $CDCl_3$) δ -111.80 (dt, *J* = 11.0, 7.6 Hz) ppm.

HRMS (ESI) m/z calcd. for $C_{20}H_{21}FN_2O_3{}^{23}Na$ ($[M+Na]^+$) 379.1428, found 379.1429

FT-IR (thin film) ν_{max} 3734, 3649, 2978, 2501, 2359, 2160, 2032, 1977, 1707, 1653, 1609, 1556, 1492, 1456, 1393, 1368, 1322, 1282, 1251, 1220, 1149, 976, 863, 771, 682 cm^{-1}.

m.p.: 167-168 ^{O}C

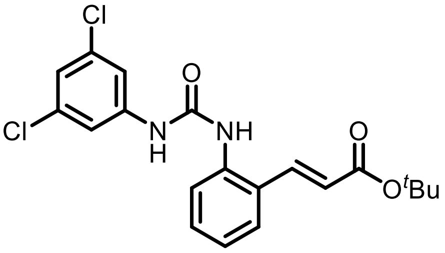

***tert*-Butyl (*E*)-3-(2-(3-(3,5-dichlorophenyl)ureido)phenyl)acrylate (1p)** was prepared following **General Procedure A**, using 2-iodoaniline (0.33 g, 1.50 mmol, 1.0 eq) and 1,3-dichloro-5-isocyanatobenzene (0.57 g, 3.00 mmol, 2.0 eq) to afford the title compound as a white powder (0.59 g, 96%).

^{1}H NMR (400 MHz, $(CD_3)_2SO$) δ 9.28 (s, 1H, N<u>H</u>), 8.65 (s, 1H, N<u>H</u>), 7.78 (d, *J* = 1.6 Hz, 1H, Ar-<u>H</u>), 7.77 – 7.73 (m, 1H, C<u>H</u>), 7.64 (dd, *J* = 8.2, 1.2 Hz, 1H, Ar-<u>H</u>), 7.54 (d, *J* = 1.9 Hz, 2H, Ar-<u>H</u>), 7.44 – 7.35 (m, 1H, Ar-<u>H</u>), 7.23 – 7.13 (m, 2H, Ar-<u>H</u>), 6.48 (d, *J* = 15.8 Hz, 1H, C<u>H</u>), 1.48 (s, 9H, OC(C<u>H</u>$_3$)$_3$) ppm.

^{13}C NMR (101 MHz, $(CD_3)_2SO$) δ 165.6 (<u>C</u>(O)OC(CH$_3$)$_3$), 152.7 (<u>C</u>(O)NH), 142.2 (Ar<u>C</u>), 138.9 (<u>C</u>H), 137.1 (Ar<u>C</u>), 134.1 (Ar<u>C</u>), 130.5 (Ar<u>C</u>H), 127.2 (Ar<u>C</u>H or Ar<u>C</u>), 127.0 (Ar<u>C</u>H or Ar<u>C</u>), 124.7 (Ar<u>C</u>H), 124.6 (Ar<u>C</u>H), 121.0 (Ar<u>C</u>H), 120.8 (<u>C</u>H), 116.4 (Ar<u>C</u>H), 80.1 (O<u>C</u>(CH$_3$)$_3$), 27.8 (OC(<u>C</u>H$_3$)$_3$) ppm.

HRMS (ESI) m/z calcd. for $C_{20}H_{20}Cl_2N_2O_3{}^{23}Na$ ($[M+Na]^+$) 429.0743, found 429.0744

FT-IR (thin film) ν_{max} 3323, 2979, 2360, 2161, 2031, 1706, 1653, 1584, 1544, 1487, 1448, 1410, 1368, 1322, 1249, 1213, 1151, 1113, 981, 845, 758, 670 cm^{-1}.

m.p.: >250 ^{O}C

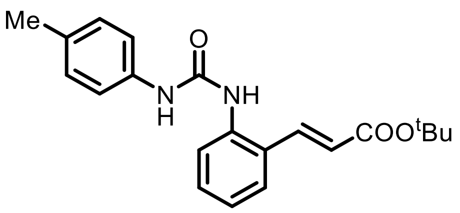

***tert*-Butyl (*E*)-3-(2-(3-(*p*-tolyl)ureido)phenyl)acrylate (1q)** was prepared following **General Procedure A**, using 2-iodoaniline (0.33 g, 1.50 mmol, 1.0 eq) and *p*-tolyl isocyanate (0.40 g, 3.00 mmol, 2.0 eq) to afford the title compound as a white powder (0.48 g, 91%).

^{1}H NMR (400 MHz, CDCl$_3$) δ 7.85 (d, *J* = 15.8 Hz, 1H, C<u>H</u>), 7.72 (dd, *J* = 8.2, 1.2 Hz, 1H, Ar-<u>H</u>), 7.54 (dd, *J* = 7.9, 1.6 Hz, 1H, Ar-<u>H</u>), 7.34 (ddd, *J* = 8.2, 7.3, 1.6 Hz, 1H, Ar-<u>H</u>), 7.31 – 7.16 (m, 2H, N<u>H</u>), 7.15 – 7.10 (m, 3H, Ar-<u>H</u>), 7.05 (d, *J* = 8.2 Hz, 2H, Ar-<u>H</u>), 6.32 (d, *J* = 15.8 Hz, 1H, C<u>H</u>), 2.27 (s, 3H, C<u>H</u>$_3$), 1.47 (s, 9H, OC(C<u>H</u>$_3$)$_3$) ppm.

^{13}C NMR (101 MHz, CDCl$_3$) δ 166.5 (<u>C</u>(O)OC(CH$_3$)$_3$), 154.2 (<u>C</u>(O)NH), 138.7 (<u>C</u>H), 136.7 (Ar<u>C</u>), 135.3 (Ar<u>C</u>), 134.2 (Ar<u>C</u>), 130.9 (Ar<u>C</u>H), 129.9 (Ar<u>C</u>H), 128.2 (Ar<u>C</u>), 127.3 (Ar<u>C</u>H), 125.39 (Ar<u>C</u>H), 125.37 (Ar<u>C</u>H), 122.3 (<u>C</u>H), 121.9 (Ar<u>C</u>H), 81.1 (O<u>C</u>(CH$_3$)$_3$), 28.3 (OC(<u>C</u>H$_3$)$_3$), 21.0 (C<u>H</u>$_3$) ppm.

HRMS (ESI) m/z calcd. for $C_{21}H_{24}N_2O_3{}^{23}Na$ ([M+Na]$^+$) 375.1679, found 375.1680

FT-IR (thin film) ν_{max} 3313, 2979, 2160, 2026, 1707, 1647, 1603, 1581, 1548, 1515, 1481, 1455, 1392, 1367, 1319, 1295, 1236, 1150, 981, 873, 816, 759 cm^{-1}.

m.p.: 219 ^{O}C

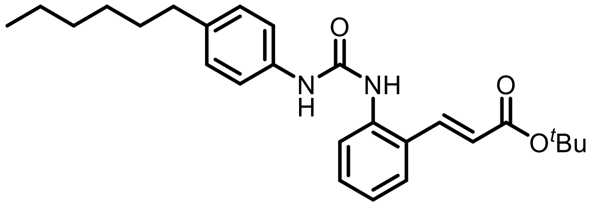

***tert*-Butyl (*E*)-3-(2-(3-(4-hexylphenyl)ureido)phenyl)acrylate (1r)** was prepared following **General Procedure B**, using 2-iodoaniline (0.40 g, 1.84 mmol, 1.0 eq) and 4-hexylaniline (0.66 g, 3.68 mmol, 2.0 eq). Purification by silica gel chromatography (20% Et_2O in pentane) afforded the title compound as a white powder (0.42 g, 54%).

^{1}H NMR (400 MHz, $CDCl_3$) δ 7.87 (d, *J* = 15.9 Hz, 1H, $C\underline{H}$), 7.72 (dd, *J* = 8.2, 1.2 Hz, 1H, Ar-$\underline{H}$), 7.53 (dd, *J* = 7.8, 1.5 Hz, 1H, Ar-$\underline{H}$), 7.39 (brs, 1H, $N\underline{H}$), 7.32 (ddd, *J* = 8.2, 7.3, 1.5 Hz, 1H, Ar-$\underline{H}$), 7.29 (brs, 1H, $N\underline{H}$), 7.19 – 7.08 (m, 3H, Ar-$\underline{H}$), 7.08 – 7.00 (m, 2H, Ar-$\underline{H}$), 6.32 (d, *J* = 15.9 Hz, 1H, $C\underline{H}$), 2.56 – 2.47 (m, 2H, $ArC\underline{H_2}$), 1.64 – 1.47 (m, 2H, $ArCH_2C\underline{H_2}$), 1.46 (s, 9H, $OC(C\underline{H_3})_3$), 1.36 – 1.22 (m, 6H, $ArCH_2CH_2C\underline{H_2}C\underline{H_2}C\underline{H_2}CH_3$), 0.92 – 0.82 (m, 3H, $C\underline{H_3}$) ppm.

^{13}C NMR (101 MHz, $CDCl_3$) δ 166.6 ($\underline{C}(O)OC(CH_3)_3$), 154.2 ($\underline{C}(O)NH$), 139.1 ($Ar\underline{C}$), 138.7 ($\underline{C}H$), 136.9 ($Ar\underline{C}$), 135.6 ($Ar\underline{C}$), 130.9 ($Ar\underline{C}H$), 129.2 ($Ar\underline{C}H$), 128.2 ($Ar\underline{C}$), 127.2 ($Ar\underline{C}H$), 125.4 ($Ar\underline{C}H$), 125.3 ($Ar\underline{C}H$), 122.1 ($\underline{C}H$), 121.7 ($Ar\underline{C}H$), 81.0 ($O\underline{C}(CH_3)_3$), 35.5 ($Ar\underline{C}H_2$), 31.9 ($ArCH_2CH_2CH_2\underline{C}H_2CH_2CH_3$), 31.6 ($ArCH_2\underline{C}H_2$), 29.1 ($ArCH_2CH_2\underline{C}H_2CH_2CH_2CH_3$), 28.3 ($OC(\underline{C}H_3)_3$), 22.8 ($ArCH_2CH_2CH_2CH_2\underline{C}H_2CH_3$), 14.2 ($\underline{C}H_3$) ppm.

HRMS (ESI) m/z calcd. for $C_{26}H_{34}N_2O_3{}^{23}Na$ ($[M+Na]^+$) 445.2462, found 445.2462

FT-IR (thin film) ν_{max} 3734, 3324, 2927, 2360, 2161, 2031, 1707, 1648, 1605, 1550, 1456, 1367, 1320, 1237, 1151, 982, 772 cm_{-1}.

m.p.: 180-183 ^{O}C

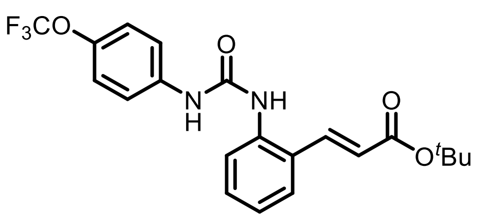

***tert*-Butyl (*E*)-3-(2-(3-(4-(trifluoromethoxy)phenyl)ureido)phenyl)acrylate (1s)** was prepared following **General Procedure A**, using 2-iodoaniline (0.48 g, 2.20 mmol, 1.0 eq) and 1-isocyanato-4-(trifluoromethoxy)benzene (0.89 g, 4.40 mmol, 2.0 eq) to afford the title compound as a white powder (0.58 g, 63%).

^{1}H NMR (400 MHz, $(CD_3)_2SO$) δ 9.15 (s, 1H, NH), 8.51 (s, 1H, NH), 7.80 (d, *J* = 15.7 Hz, 1H, CH), 7.75 (dd, *J* = 8.0, 1.5 Hz, 1H, Ar-H), 7.72 (dd, *J* = 8.2, 1.2 Hz, 1H, Ar-H), 7.61 – 7.52 (m, 2H, Ar-H), 7.38 (ddd, *J* = 8.4, 7.3, 1.6 Hz, 1H, Ar-H), 7.33 – 7.25 (m, 2H, Ar-H), 7.13 (td, *J* = 7.7, 1.1 Hz, 1H, Ar-H), 6.47 (d, *J* = 15.7 Hz, 1H, CH), 1.48 (s, 9H, $OC(CH_3)_3$) ppm.

^{13}C NMR (101 MHz, $(CD_3)_2SO$) δ 165.6 ($C(O)OC(CH_3)_3$), 152.8 (C(O)NH), 142.6 (ArCO), 139.0 (ArC), 138.9 (CH), 137.5 (ArC), 130.5 (ArCH), 127.0 (ArCH), 126.5 (ArC), 124.13 (ArCH), 123.95 (ArCH), 121.7 (ArCH), 121.5 (CF_3), 120.8 (CH), 119.4 (ArCH), 80.0 ($OC(CH_3)_3$), 27.8 ($OC(CH_3)_3$) ppm. (C-F coupling not observed)

^{19}F NMR (377 MHz, $(CD_3)_2SO$) δ -56.89 (s)(assigned to self-cyclized product in $(CD_3)SO$ during nmr measurement), -57.11 (s) ppm.

HRMS (ESI) m/z calcd. for $C_{21}H_{21}F_3N_2O_4{}^{23}Na$ ($[M+Na]^+$) 445.1346, found 445.1347

FT-IR (thin film) ν_{max} 3325, 2980, 2360, 2161, 2032, 1708, 1652, 1610, 1557, 1509, 1457, 1411, 1369, 1323, 1263, 1238, 1202, 1154, 982, 849, 761, 670 cm^{-1}.

m.p.: 180 ^{O}C

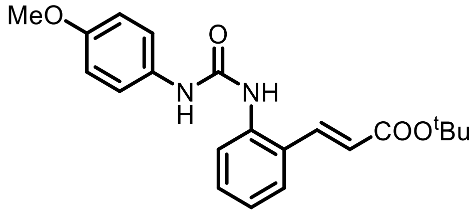

***tert*-Butyl (*E*)-3-(2-(3-(4-methoxyphenyl)ureido)phenyl)acrylate (1t)** was prepared following **General Procedure A**, using 2-iodoaniline (0.32 g, 1.43 mmol, 1.0 eq) and 4-Methoxyphenyl isocyanate (0.34 g, 2.86 mmol, 2.0 eq) to afford the title compound as a white powder (0.43 g, 82%).

^{1}H NMR (400 MHz, CDCl$_3$) δ 7.85 (d, *J* = 15.8 Hz, 1H, CH), 7.70 (dd, *J* = 8.3, 1.2 Hz, 1H, CH=CHAr-H), 7.52 (dd, *J* = 7.9, 1.6 Hz, 1H, CH=CHAr-H), 7.42 (brs, 1H, NH), 7.35 (brs, 1H, NH), 7.30 (ddd, *J* = 8.3, 7.4, 1.6 Hz, 1H, CH=CHAr-H), 7.14 – 7.06 (m, 3H, CH=CHAr-H and Ar-H), 6.85 – 6.67 (m, 2H, Ar-H), 6.30 (d, *J* = 15.8 Hz, 1H, CH), 3.74 (s, 3H, OCH$_3$), 1.45 (s, 9H, OC(CH$_3$)$_3$) ppm.

^{13}C NMR (101 MHz, CDCl$_3$) δ 166.6 (C(O)OC(CH$_3$)$_3$), 156.9 (COCH$_3$ or C(O)NH), 154.7 (COCH$_3$ or C(O)NH), 138.7 (CH), 136.9 (CH=CHArC or ArC), 130.9 (CH=CHArCH), 130.8 (CH=CHArC or ArC), 128.0 (CH=CHArC or ArC), 127.2 (CH=CHArCH), 125.3 (CH=CHArCH), 125.2 (CH=CHArCH), 124.2 (ArCH), 122.0 (CH), 114.5 (ArCH), 81.0 (OC(CH$_3$)$_3$), 55.6 (OCH$_3$), 28.3 (OC(CH$_3$)$_3$) ppm.

HRMS (ESI) m/z calcd. for $C_{21}H_{24}N_2O_4{}^{23}Na$ ($[M+Na]^+$) 391.1628, found 391.1629

FT-IR (thin film) ν_{max} 3628, 3309, 2520, 2364, 2160, 2028, 1978, 1707, 1646, 1555, 1511, 1367, 1322, 1233, 1151, 982, 832, 760, 626 cm^{-1}.

m.p.: 208-210 ^{O}C

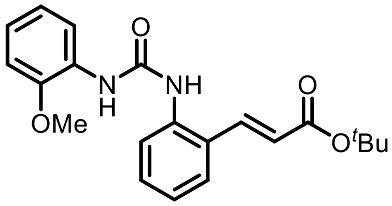

***tert*-Butyl (*E*)-3-(2-(3-(2-methoxyphenyl)ureido)phenyl)acrylate (1u)** was prepared following **General Procedure B**, using 2-iodoaniline (0.29 g, 1.31 mmol, 1.0 eq) and 2-methoxyaniline (0.33 g, 2.62 mmol, 2.0 eq). Purification by silica gel chromatography (15% EtOAc in pentane) afforded the title compound as an off-white powder (0.44 g, 90%).

^{1}H NMR (400 MHz, $CDCl_3$) δ 8.14 (dd, *J* = 7.9, 1.9 Hz, 1H, Ar-H), 7.93 (d, *J* = 15.8 Hz, 1H, CH), 7.77 (dd, *J* = 8.3, 1.2 Hz, 1H, Ar-H), 7.54 (dd, *J* = 7.9, 1.6 Hz, 1H, Ar-H), 7.50 (brs, 1H, NH), 7.48 (brs, 1H, NH), 7.36 (ddd, *J* = 8.3, 7.3, 1.6 Hz, 1H, Ar-H), 7.19 – 7.10 (m, 1H, Ar-H), 6.95 (td, *J* = 7.6, 1.9 Hz, 1H, Ar-H), 6.90 (td, *J* = 7.6, 1.7 Hz, 1H, Ar-H), 6.79 (dd, *J* = 7.9, 1.7 Hz, 1H, Ar-H), 6.34 (d, *J* = 15.8 Hz, 1H, CH), 3.73 (s, 3H, OCH_3), 1.46 (s, 9H, $OC(CH_3)_3$) ppm.

^{13}C NMR (101 MHz, $CDCl_3$) δ 166.7 ($C(O)OC(CH_3)_3$), 153.4 (C(O)NH), 148.5 (ArC), 139.1 (CH), 137.0 (ArC), 130.8 (ArCH), 128.25 (ArC), 128.19 (ArC), 127.3 (ArCH), 125.3 (ArCH), 125.2 (ArCH), 123.0 (ArCH), 122.2 (CH), 121.3 (ArCH), 119.9 (ArCH), 110.2 (ArCH), 81.1 ($OC(CH_3)_3$), 55.7 (OCH_3), 28.3 ($OC(CH_3)_3$) ppm.

HRMS (ESI) m/z calcd. for $C_{21}H_{24}N_2O_4{}^{23}Na$ ($[M+Na]^+$) 391.1628, found 391.1629

FT-IR (thin film) ν_{max} 3649, 3333, 2979, 2916, 2850, 2360, 2341, 2161, 2031, 1707, 1654, 1635, 1602, 1582, 1542, 1489, 1459, 1435, 1367, 1323, 1291, 1248, 1217, 1151, 1122, 1029, 982, 871, 749, 669 cm^{-1}.

m.p.: 191-192 ^{O}C

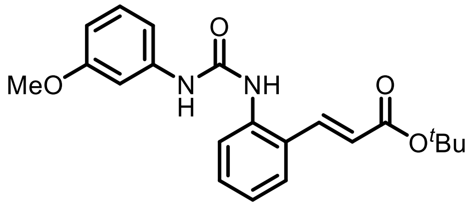

***tert*-Butyl (*E*)-3-(2-(3-(3-methoxyphenyl)ureido)phenyl)acrylate (1v)** was prepared following **General Procedure B**, using 2-iodoaniline (0.29 g, 1.30 mmol, 1.0 eq) and 3-methoxyaniline (0.32 g, 2.60 mmol, 2.0 eq). Purification by silica gel chromatography (20% EtOAc in pentane) afforded the title compound as an off-white powder (0.29 g, 60%).

^{1}H NMR (400 MHz, CDCl$_3$) δ 7.89 (d, *J* = 15.8 Hz, 1H, CH), 7.69 (dd, *J* = 8.2, 1.2 Hz, 1H, Ar-H), 7.57 – 7.50 (m, 2H, Ar-H and NH), 7.42 (brs, 1H, NH), 7.33 (ddd, *J* = 8.2, 7.3, 1.5 Hz, 1H, Ar-H), 7.15 – 7.10 (m, 1H, Ar-H), 7.10 (t, *J* = 8.2 Hz, 1H, Ar-H), 7.01 (t, *J* = 2.2 Hz, 1H, Ar-H), 6.69 (ddd, *J* = 8.0, 2.0, 0.9 Hz, 1H, Ar-H), 6.57 (ddd, *J* = 8.2, 2.5, 0.9 Hz, 1H, Ar-H), 6.33 (d, *J* = 15.8 Hz, 1H, CH), 3.72 (s, 3H, OCH$_3$), 1.45 (s, 9H, OC(CH$_3$)$_3$) ppm.

^{13}C NMR (101 MHz, CDCl$_3$) δ 166.7 (C(O)OC(CH$_3$)$_3$), 160.4 (ArCO), 154.0 (C(O)NH), 139.5 (ArC), 138.7 (CH), 136.7 (ArC), 130.9 (ArCH), 129.8 (ArCH), 128.3 (ArC), 127.2 (ArCH), 125.54 (ArCH), 125.50 (ArCH), 122.2 (CH), 113.2 (ArCH), 110.1 (ArCH), 106.6 (ArCH), 81.1 (OC(CH$_3$)$_3$), 55.4 (OCH$_3$), 28.3 (OC(CH$_3$)$_3$) ppm.

HRMS (ESI) m/z calcd. for $C_{21}H_{24}N_2O_4{}^{23}Na$ ([M+Na]$^+$) 391.1628, found 391.1629

FT-IR (thin film) ν_{max} 3317, 2978, 2553, 2160, 2031, 1706, 1650, 1603, 1582, 1553, 1494, 1455, 1429, 1392, 1367, 1322, 1289, 1252, 1221, 1155, 1042, 981, 858, 760, 689 cm^{-1}.

m.p.: 177-179 ^{O}C

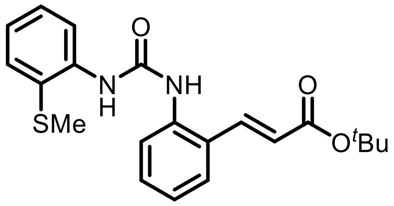

***tert*-Butyl (*E*)-3-(2-(3-(2-(methylthio)phenyl)ureido)phenyl)acrylate (1w)** was prepared following **General Procedure B**, using 2-iodoaniline (0.43 g, 1.93 mmol, 1.0 eq) and 2-(methylthio)aniline (0.54 g, 3.86 mmol, 2.0 eq). Purification by silica gel chromatography (20% EtOAc in pentane) afforded the title compound as an off-white powder (0.51 g, 69%).

^{1}H NMR (400 MHz, $CDCl_3$) δ 8.07 (dd, *J* = 8.2, 1.4 Hz, 1H, Ar-H), 7.92 (d, *J* = 15.9 Hz, 1H, CH), 7.73 (s, 1H, NH), 7.70 (dd, *J* = 8.1, 1.2 Hz, 1H, Ar-H), 7.59 (dd, *J* = 7.9, 1.5 Hz, 1H, Ar-H), 7.39 (ddd, *J* = 8.1, 7.3, 1.5 Hz, 1H, Ar-H), 7.35 (dd, *J* = 7.8, 1.5 Hz, 1H, Ar-H), 7.29 (s, 1H, NH), 7.22 (ddt, *J* = 9.7, 7.5, 1.6 Hz, 2H, Ar-H), 6.99 (td, *J* = 7.6, 1.4 Hz, 1H, Ar-H), 6.35 (d, *J* = 15.9 Hz, 1H, CH), 2.21 (s, 3H, SCH_3), 1.47 (s, 9H, $OC(CH_3)_3$) ppm.

^{13}C NMR (101 MHz, $CDCl_3$) δ 166.4 ($C(O)OC(CH_3)_3$), 153.7 (C(O)NH), 138.7 (CH), 138.4 (ArC), 136.3 (ArC), 132.2 (ArCH), 130.9 (ArCH), 129.5 (ArC), 128.6 (ArCH), 127.5 (ArCH), 126.8 (ArC), 126.4 (ArCH), 126.2 (ArCH), 124.1 (ArCH), 122.6 (CH), 121.4 (ArCH), 81.0 ($OC(CH_3)_3$), 28.3 ($OC(CH_3)_3$), 18.4 (SCH_3) ppm.

HRMS (ESI) m/z calcd. for $C_{21}H_{24}{}^{32}SN_2O_3{}^{23}Na$ ($[M+Na]^+$) 407.1400, found 407.1400

FT-IR (thin film) ν_{max} 3298, 2978, 2360, 2160, 2031, 1707, 1652, 1578, 1534, 1479, 1455, 1437, 1392, 1367, 1322, 1295, 1259, 1228, 1150, 1039, 981, 871, 755, 668 cm^{-1}.

m.p.: 171-172 ^{O}C

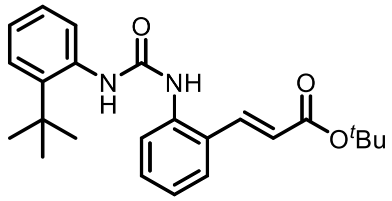

***tert*-Butyl (*E*)-3-(2-(3-(2-(tert-butyl)phenyl)ureido)phenyl)acrylate (1x)** was prepared following **General Procedure B**, using 2-iodoaniline (0.27 g, 1.22 mmol, 1.0 eq) and 2-(*tert*-butyl)aniline (0.37 g, 2.44 mmol, 2.0 eq). Purification by silica gel chromatography (40% Et_2O in pentane) afforded the title compound as an off-white powder (0.37 g, 77%).

^{1}H NMR (400 MHz, $CDCl_3$) δ 7.72 (dd, *J* = 8.1, 1.2 Hz, 1H, Ar-H), 7.67 (d, *J* = 15.9 Hz, 1H, CH), 7.48 (dd, *J* = 7.8, 1.6 Hz, 1H, Ar-H), 7.43 (td, *J* = 7.8, 1.8 Hz, 2H, Ar-H), 7.32 (ddd, *J* = 8.1, 7.4, 1.6 Hz, 1H, Ar-H), 7.30 – 7.20 (m, 2H, Ar-H), 7.20 – 7.09 (m, 1H, Ar-H), 6.67 (s, 1H, NH), 6.56 (s, 1H, NH), 6.22 (d, *J* = 15.9 Hz, 1H, CH), 1.49 (s, 9H, $OC(CH_3)_3$), 1.37 (s, 9H, $ArC(CH_3)_3$) ppm.

^{13}C NMR (101 MHz, $CDCl_3$) δ 165.9 ($C(O)OC(CH_3)_3$), 154.8 (C(O)NH), 146.4 (ArC), 138.5 (CH), 136.4 (ArC), 135.2 (ArC), 130.7 (ArCH), 130.3 (ArCH), 128.7 (ArC), 127.64 (ArCH), 127.62 (ArCH), 127.5 (ArCH), 127.3 (ArCH), 125.5 (ArCH), 125.4 (ArCH), 122.9 (CH), 80.8 ($OC(CH_3)_3$), 35.2 ($ArC(CH_3)_3$), 30.8 ($ArC(CH_3)_3$), 28.3 ($OC(CH_3)_3$) ppm.

HRMS (ESI) m/z calcd. for $C_{24}H_{30}N_2O_3{}^{23}Na$ ($[M+Na]^+$) 417.2149, found 417.2149

FT-IR (thin film) ν_{max} 3271, 2966, 2555, 2160, 2025, 1977, 1708, 1631, 1599, 1550, 1485, 1366, 1320, 1288, 1236, 1151, 983, 866, 755 cm^{-1}.

m.p.: 182-184 ^{O}C

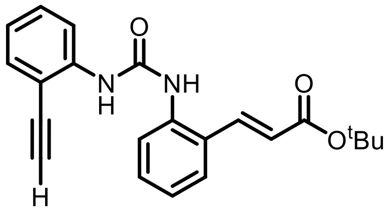

***tert*-Butyl (*E*)-3-(2-(3-(2-ethynylphenyl)ureido)phenyl)acrylate (1y)** was prepared following **General Procedure B**, using 2-iodoaniline (0.32 g, 1.46 mmol, 1.0 eq) and 2-ethynyl aniline (0.30 g, 2.54 mmol, 2.0 eq). Purification by silica gel chromatography (25% Et_2O in pentane) afforded the title compound as an off-white powder (0.42 g, 79%).

^{1}H NMR (400 MHz, $CDCl_3$) δ 8.24 (dd, *J* = 8.5, 1.1 Hz, 1H, Ar-H), 7.92 (d, *J* = 15.9 Hz, 1H, CH), 7.63 (ddd, *J* = 8.0, 6.7, 1.5 Hz, 2H, Ar-H), 7.52 (s, 1H, NH), 7.41 (td, *J* = 7.6, 1.5 Hz, 1H, Ar-H), 7.37 – 7.23 (m, 3H, Ar-H), 7.18 (s, 1H, NH), 6.93 (td, *J* = 7.6, 1.1 Hz, 1H, Ar-H), 6.37 (d, *J* = 15.9 Hz, 1H, CH), 3.16 (s, 1H, C≡CH), 1.47 (s, 9H, $OC(CH_3)_3$) ppm.

^{13}C NMR (101 MHz, $CDCl_3$) δ 166.3 ($C(O)OC(CH_3)_3$), 153.3 (C(O)NH), 140.5 (ArC), 138.4 (CH), 136.0 (ArC), 132.2 (ArCH), 131.0 (ArCH), 130.3 (ArCH), 127.6 (ArCH), 127.1 (ArCH), 126.7 (ArC), 122.9 (CH), 122.5 (ArCH), 119.2 (ArCH), 110.9 (ArC), 84.0 (C≡CH), 81.1 ($OC(CH_3)_3$ or C≡CH), 79.4 ($OC(CH_3)_3$ or C≡CH), 28.3 ($OC(CH_3)_3$) ppm.

HRMS (ESI) m/z calcd. for $C_{22}H_{22}N_2O_3{}^{23}Na$ ($[M+Na]^+$) 385.1523, found 385.1523

FT-IR (thin film) ν_{max} 3278, 2977, 2511, 2160, 2032, 1977, 1707, 1648, 1608, 1580, 1546, 1480, 1448, 1391, 1367, 1322, 1298, 1239, 1152, 1043, 979, 876, 755, 668, 629 cm^{-1}.

m.p.: 160-162 ^{O}C

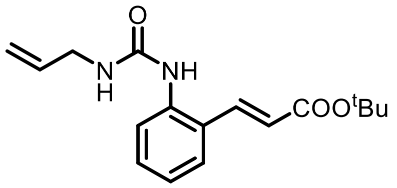

***tert*-Butyl (*E*)-3-(2-(3-allylureido)phenyl)acrylate (1z)** was prepared following **General Procedure A**, using 2-iodoaniline (0.22 g, 1.00 mmol, 1.0 eq) and 3-isocyanatoprop-1-ene (0.17 g, 2.00 mmol, 2.0 eq) to afford the title compound as a white powder (0.25 g, 82%).

^{1}H NMR (400 MHz, CDCl$_3$) δ 7.87 (d, *J* = 15.9 Hz, 1H, C<u>H</u>CH), 7.63 (dd, *J* = 8.1, 1.2 Hz, 1H, Ar-<u>H</u>), 7.55 (dd, *J* = 7.9, 1.6 Hz, 1H， Ar-<u>H</u>), 7.34 (ddd, *J* = 8.1, 7.3, 1.6 Hz, 1H, Ar-<u>H</u>), 7.21 (brs, 1H, N<u>H</u>), 7.18 – 7.10 (m, 1H, Ar-<u>H</u>), 6.33 (d, *J* = 15.9 Hz, 1H, C<u>H</u>CH), 5.83 (ddt, *J* = 17.2, 10.3, 5.5 Hz, 1H, C<u>H</u>=CH$_2$), 5.68 - 4.57 (brs, 1H, N<u>H</u>), 5.17 (dq, *J* = 17.2, 1.6 Hz, 1H, CH=C<u>H$_2$</u>), 5.09 (dq, *J* = 10.3, 1.6 Hz, 1H, CH=C<u>H$_2$</u>), 3.83 (dt, *J* = 5.5, 1.6 Hz, 2H, NHC<u>H$_2$</u>), 1.47 (s, 9H, OC(C<u>H$_3$</u>)$_3$) ppm.

^{13}C NMR (101 MHz, CDCl$_3$) δ 166.5 (<u>C</u>(O)OC(CH$_3$)$_3$), 156.3 (<u>C</u>(O)NH), 138.8 (<u>C</u>HCH), 137.2 (Ar<u>C</u>), 135.1 (<u>C</u>H=CH$_2$), 131.0 (Ar<u>C</u>H), 128.6 (Ar<u>C</u>), 127.3 (Ar<u>C</u>H), 125.7 (Ar<u>C</u>H), 125.4 (Ar<u>C</u>H), 122.2 (CH<u>C</u>H), 116.1 (CH=<u>C</u>H$_2$), 81.0 (O<u>C</u>(CH$_3$)$_3$), 43.0 (NH<u>C</u>H$_2$), 28.3 (OC(<u>C</u>H$_3$)$_3$) ppm.

HRMS (ESI) m/z calcd. for $C_{17}H_{22}N_2O_3{}^{23}Na$ ([M+Na]$^+$) 325.1523, found 325.1523

FT-IR (thin film) ν_{max} 3333, 2978, 2160, 2031, 1707, 1637, 1556, 1476, 1455, 1392, 1367, 1322, 1242, 1150, 985, 918, 872, 760, 668 cm^{-1}.

m.p.: 136-138 ^{O}C

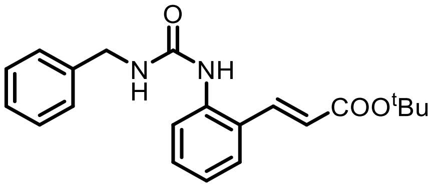

***tert*-Butyl (*E*)-3-(2-(3-benzylureido)phenyl)acrylate (1aa)** was prepared following **General Procedure A**, using 2-iodoaniline (0.31 g, 1.42 mmol, 1.0 eq) and 1-isocyanatobutane (0.38 g, 2.84 mmol, 2.0 eq) to afford the title compound as a white powder (0.38 g, 86%).

^{1}H NMR (400 MHz, $CDCl_3$) δ 7.89 (d, *J* = 15.9 Hz, 1H, C<u>H</u>), 7.63 (dd, *J* = 8.1, 1.2 Hz, 1H, Ar-<u>H</u>), 7.53 (dd, *J* = 7.9, 1.6 Hz, 1H, Ar-<u>H</u>), 7.35 – 7.28 (m, 2H, N<u>H</u> and Ar-<u>H</u>), 7.27 – 7.17 (m, 5H, Ar-<u>H</u>), 7.12 (td, *J* = 7.6, 1.2 Hz, 1H, Ar-<u>H</u>), 6.31 (d, *J* = 15.9 Hz, 1H, C<u>H</u>), 5.57 (t, *J* = 5.7 Hz, 1H, $CH_2N\underline{H}$), 4.29 (d, *J* = 5.7 Hz, 2H, $C\underline{H}_2$), 1.46 (s, 9H, $OC(C\underline{H}_3)_3$) ppm.

^{13}C NMR (101 MHz, $CDCl_3$) δ 166.5 ($\underline{C}(O)OC(CH_3)_3$), 156.4 (<u>C</u>(O)NH), 138.9 (<u>C</u>H), 138.8 (Ar<u>C</u>), 137.2 (Ar<u>C</u>), 131.0 (Ar<u>C</u>H), 128.7 (Ar<u>C</u>H), 128.6 (Ar<u>C</u>), 127.5 (Ar<u>C</u>H), 127.4 (Ar<u>C</u>H), 127.3 (Ar<u>C</u>H), 125.7 (Ar<u>C</u>H), 125.4 (Ar<u>C</u>H), 122.0 (<u>C</u>H), 81.0 ($O\underline{C}(CH_3)_3$), 44.4 ($\underline{C}H_2$), 28.3 ($OC(\underline{C}H_3)_3$) ppm.

HRMS (ESI) m/z calcd. for $C_{21}H_{24}N_2O_3{}^{23}Na$ ($[M+Na]^+$) 375.1679, found 375.1680

FT-IR (thin film) ν_{max} 3333, 2980, 2531, 2359, 2161, 2027, 1977, 1707, 1639, 1558, 1455, 1367, 1322, 1250, 1150, 983, 761, 698 cm^{-1}.

m.p.: 123-124 ^{O}C

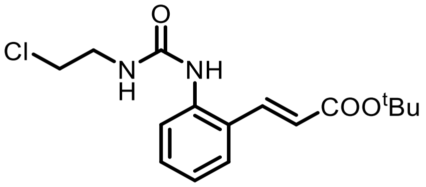

***tert*-Butyl (*E*)-3-(2-(3-(2-chloroethyl)ureido)phenyl)acrylate (1ab)** was prepared following **General Procedure A**, using 2-iodoaniline (0.22 g, 1.00 mmol, 1.0 eq) and 1-chloro-2-isocyanatoethane (0.21 g, 2.00 mmol, 2.0 eq) to afford the title compound as a white powder (0.26 g, 78%).

^{1}H NMR (400 MHz, $CDCl_3$) δ 7.78 (d, *J* = 15.9 Hz, 1H, C<u>H</u>), 7.52 (ddd, *J* = 13.8, 7.9, 1.5 Hz, 2H, Ar-<u>H</u>), 7.29 (td, *J* = 7.9, 1.5 Hz, 1H, Ar-<u>H</u>), 7.14 – 7.08 (m, 1H, Ar-<u>H</u>), 7.06 (brs, 1H, N<u>H</u>), 6.27 (d, *J* = 15.9 Hz, 1H, C<u>H</u>), 5.45 (brs, 1H, N<u>H</u>), 3.60 – 3.53 (m, 2H, C<u>H</u>$_2$), 3.50 (td, *J* = 5.0, 4.5, 1.3 Hz, 2H, C<u>H</u>$_2$), 1.41 (s, 9H, OC(C<u>H</u>$_3$)$_3$) ppm.

^{13}C NMR (101 MHz, $CDCl_3$) δ 166.5 (<u>C</u>(O)OC(CH$_3$)$_3$), 156.1 (<u>C</u>(O)NH), 138.6 (<u>C</u>H), 136.7 (Ar<u>C</u>), 131.0 (Ar<u>C</u>H), 128.9 (Ar<u>C</u>), 127.4 (Ar<u>C</u>H), 125.84 (Ar<u>C</u>H), 125.78 (Ar<u>C</u>H), 122.4 (<u>C</u>H), 81.1 (O<u>C</u>(CH$_3$)$_3$), 44.7 (<u>C</u>H$_2$), 42.3 (<u>C</u>H$_2$), 28.3 (OC(<u>C</u>H$_3$)$_3$) ppm.

HRMS (ESI) m/z calcd. for $C_{16}H_{21}ClN_2O_3{}^{23}Na$ ([M+Na]$^+$) 347.1133, found 347.1133

FT-IR (thin film) ν_{max} 3334, 2978, 2161, 2032, 1705, 1640, 1556, 1476, 1455, 1392, 1368, 1322, 1248, 1150, 983, 871, 759, 658 cm^{-1}.

m.p.: 128-129 ^{O}C

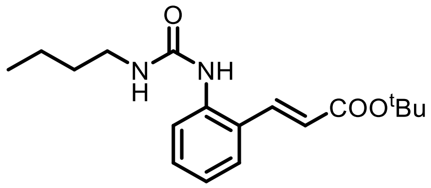

***tert*-Butyl (*E*)-3-(2-(3-butylureido)phenyl)acrylate (1ac)** was prepared following **General Procedure A**, using 2-iodoaniline (0.30 g, 1.35 mmol, 1.0 eq) and 1-isocyanatobutane (0.27 g, 2.70 mmol, 2.0 eq) to afford the title compound as a white powder (0.33 g, 76%).

^{1}H NMR (400 MHz, $CDCl_3$) δ 7.87 (d, *J* = 15.9 Hz, 1H, C<u>H</u>), 7.61 (dd, *J* = 8.1, 1.3 Hz, 1H, Ar-<u>H</u>), 7.54 (dd, *J* = 7.9, 1.5 Hz, 1H, Ar-<u>H</u>), 7.32 (ddd, *J* = 8.1, 7.2, 1.5 Hz, 1H, Ar-<u>H</u>), 7.27 (s, 1H, N<u>H</u>), 7.16 – 7.07 (m, 1H, Ar-<u>H</u>), 6.32 (d, *J* = 15.9 Hz, 1H, C<u>H</u>), 5.24 (s, 1H, N<u>H</u>), 3.18 (t, *J* = 7.3 Hz, 2H, C<u>H</u>$_2$), 1.49 – 1.39 (m, 11H, OC(C<u>H</u>$_3$)$_3$ and C<u>H</u>$_2$), 1.36 – 1.23 (m, 2H, C<u>H</u>$_2$), 0.88 (t, *J* = 7.3 Hz, 3H, CH_2C<u>H</u>$_3$) ppm.

^{13}C NMR (101 MHz, $CDCl_3$) δ 166.6 (<u>C</u>(O)OC(CH_3)$_3$), 156.5 (<u>C</u>(O)NH), 138.9 (<u>C</u>H), 137.5 (Ar<u>C</u>), 130.9 (Ar<u>C</u>H), 128.4 (Ar<u>C</u>), 127.2 (Ar<u>C</u>H), 125.6 (Ar<u>C</u>H), 125.1 (Ar<u>C</u>H), 121.9 (<u>C</u>H), 80.9 (O<u>C</u>(CH_3)$_3$), 40.3 (<u>C</u>H_2), 32.3 (<u>C</u>H_2), 28.3 (OC(<u>C</u>H_3)$_3$), 20.2 (<u>C</u>H_2), 13.9 (CH_2<u>C</u>H_3) ppm.

HRMS (ESI) m/z calcd. for $C_{18}H_{26}N_2O_3{}^{23}Na$ ($[M+Na]^+$) 341.1836, found 341.1836

FT-IR (thin film) ν_{max} 3329, 2961, 2931, 2161, 2025, 1709, 1639, 1560, 1476, 1455, 1392, 1367, 1321, 1244, 1149, 983, 759 cm^{-1}.

m.p.: 127-129 ^{O}C

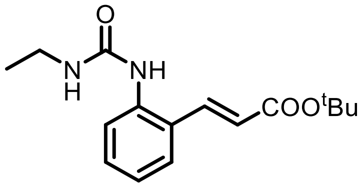

***tert*-Butyl (*E*)-3-(2-(3-ethylureido)phenyl)acrylate (1ad)** was prepared following **General Procedure A**, using 2-iodoaniline (0.23 g, 1.02 mmol, 1.0 eq) and isocyanatoethane (0.15 g, 2.04 mmol, 2.0 eq) to afford the title compound as a white powder (0.23 g, 76%).

^{1}H NMR (400 MHz, $(CD_3)_2SO$) δ 8.18 (s, 1H, N<u>H</u>), 7.75 (d, *J* = 15.7 Hz, 1H, C<u>H</u>), 7.68 (ddd, *J* = 7.8, 5.1, 1.5 Hz, 2H, Ar-<u>H</u>), 7.31 (ddd, *J* = 8.4, 7.2, 1.5 Hz, 1H, Ar-<u>H</u>), 7.03 (td, *J* = 7.8, 1.0 Hz, 1H, Ar-<u>H</u>), 6.47 – 6.35 (m, 2H, C<u>H</u> and N<u>H</u>), 3.11 (qd, *J* = 7.2, 5.5 Hz, 2H, C<u>H</u>$_2$), 1.49 (s, 9H, OC(C<u>H</u>$_3$)$_3$), 1.06 (t, *J* = 7.2 Hz, 3H, C<u>H</u>$_3$) ppm.

^{13}C NMR (101 MHz, $(CD_3)_2SO$) δ 165.7 (<u>C</u>(O)OC(CH$_3$)$_3$), 155.3 (<u>C</u>(O)NH), 139.3 (<u>C</u>H), 138.7 (Ar<u>C</u>), 130.4 (Ar<u>C</u>H), 126.9 (Ar<u>C</u>H), 125.5 (Ar<u>C</u>), 123.3 (Ar<u>C</u>H), 123.0 (Ar<u>C</u>H), 120.1 (<u>C</u>H), 79.9 (O<u>C</u>(CH$_3$)$_3$), 34.1 (<u>C</u>H$_2$), 27.9 (OC(<u>C</u>H$_3$)$_3$), 15.4 (<u>C</u>H$_3$) ppm.

HRMS (ESI) m/z calcd. for $C_{16}H_{22}N_2O_3{}^{23}Na$ ([M+Na]$^+$) 313.1523, found 313.1523

FT-IR (thin film) ν_{max} 3327, 2979, 2161, 2026, 1707, 1640, 1557, 1476, 1455, 1367, 1322, 1243, 1149, 982, 876, 759, 668 cm^{-1}.

m.p.: 111-113 ^{O}C

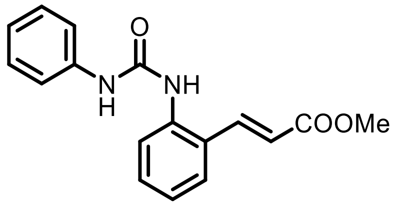

Methyl (*E*)-3-(2-(3-phenylureido)phenyl)acrylate (1ae) was prepared following **General Procedure A**, using 2-iodoaniline (8.18 g, 37.35 mmol, 1.0 eq) and phenyl isocyanate (8.90 g, 74.70 mmol, 2.0 eq) to afford the title compound as a white powder (10.09 g, 91%). Data is consistent with the published literature.[10]

^{1}H NMR (400 MHz, (CD$_3$)$_2$SO) δ 8.94 (s, 1H, NH), 8.49 (s, 1H, NH), 7.89 (d, *J* = 15.8 Hz, 1H, CH), 7.76 (dd, *J* = 8.2, 1.3 Hz, 2H, CH=CHAr-H), 7.49 – 7.44 (m, 2H, Ar-H), 7.40 (ddd, *J* = 8.6, 7.3, 1.3 Hz, 1H, CH=CHAr-H), 7.32 – 7.26 (m, 2H, Ar-H), 7.16 – 7.11 (m, 1H, CH=CHAr-H), 7.02 – 6.95 (m, 1H, Ar-H), 6.59 (d, *J* = 15.8 Hz, 1H, CH), 3.74 (s, 3H, OCH$_3$) ppm.

^{13}C NMR (101 MHz, (CD$_3$)$_2$SO) δ 166.7 (C(O)O), 152.7 (C(O)NH), 139.8 (ArC), 139.6 (CH), 137.8 (CH=CHArC), 130.7 (CH=CHArCH), 128.8 (ArCH), 127.5 (CH=CHArC), 127.1(CH=CHArCH), 123.8 (CH=CHArCH), 123.7 (CH=CHArCH), 121.9 (ArCH), 118.8 (CH), 118.2 (ArCH), 51.5 (OCH$_3$) ppm.

HRMS (ESI) m/z calcd. for $C_{17}H_{17}N_2O_3$ ($[M+H]^+$) 297.12337, found 297.12327

FT-IR (thin film) ν_{max} 3280, 2950, 2555, 2160, 2024, 1977, 1718, 1642, 1599, 1582, 1552, 1498, 1445, 1322, 1275, 1235, 1195, 1171, 976, 760, 749, 693 cm^{-1}.

m.p.: 181-182 ^{O}C

5. Preparation of Racemic Compounds

DBU (0.2 eq) was added to a solution of starting materials (0.20 mmol, 1.0 eq) in THF (0.1M) under N_2 at room temperature. The reaction was stirred at room temperature for 16 hours. before diluting with CH_2Cl_2 (10 mL). The resulting mixture was then washed with 1M HCl aqueous solution (20 mL) and H_2O (20 mL). The combined organic layers were dried over Na_2SO_4, filtered and evaporated to dryness under reduced pressure to afford the pure samples of racemic compounds for HPLC analysis.

6. Preparation of Enantioenriched Products

General Procedure C for the intramolecular aza-Michael reaction

Et_2O (0.0125 M, 1.6 mL) was added to the BIMP catalysts precursors **S9** (9.5 mg, 0.02 mmol, 0.1 eq) and tris(4-methoxyphenyl)phosphine (7.2 mg, 0.02 mmol, 0.1 eq) in a sealed mass spectrometry vial under N_2 at room temperature. The reaction mixture was stirred for 24 hours before evaporating to dryness under a stream of nitrogen gas. The iminophosphorane product was confirmed by HRMS and TLC and used as crude for enantioselective reactions without any purification.

Active BIMP catalyst HRMS (ESI) m/z calcd. for $C_{47}H_{52}N_4O_6P$ ($[M+H]^+$) 799.3619, found 799.3615.

Corrsponding urea (0.2 mmol, 1.0 eq) was added to a solution of the *in situ* generated catalyst (0.02 mmol, 0.1 eq) in toluene (0.025M, 8 mL) unless otherwise indicated under N_2 at room temperature. The reaction mixture was stirred at room temperature for 24 hours unless otherwise indicated before loaded directly onto silica gel. Purification by silica gel chromatography (pentane/EtOAc) afforded the pure intramolecular aza-Michael reaction products, which were taken the isolated yield and analysed by chiral HPLC.

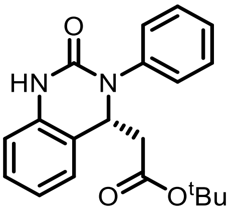

***tert*-Butyl (*R*)-2-(2-oxo-3-phenyl-1,2,3,4-tetrahydroquinazolin-4-yl)acetate (2a)** was prepared following **General Procedure C**, using ***tert*-butyl (*E*)-3-(2-(3-phenylureido)phenyl)acrylate (1a)** (16.9 mg, 0.05 mmol, 1.0 eq). Purification by silica gel chromatography (pentane/EtOAc = 7/3) afforded the title compound as a white solid (9:1 (**A**:**B**) mixture of rotamers) (16.7 mg, 99%, 94.5:5.5 er).

^{1}H NMR (400 MHz, $CDCl_3$) δ 8.12 (s, 0.1H, NH (**B**)), 8.08 (s, 0.9H, NH (**A**)), 7.48 – 7.39 (m, 4H, Ar-H), 7.30 (ddt, *J* = 5.7, 4.6, 3.8 Hz, 1H, Ar-H), 7.19 (ddd, *J* = 15.9, 7.9, 1.4 Hz, 2H, Ar-H), 6.97 (td, *J* = 7.5, 1.1 Hz, 1H, Ar-H), 6.81 (dd, *J* = 7.9, 1.1 Hz, 0.1H, Ar-H, (**B**)), 6.77 (dd, *J* = 7.9, 1.1 Hz, 0.9H, Ar-H, (**A**)), 5.23 (dd, *J* = 8.5, 4.6 Hz, 1H, CH), 2.84 – 2.66 (m, 2H, CH_2), 1.32 (s, 8.1H, $OC(CH_3)_3$ (**A**)), 1.31 (s, 0.9H, (**B**)) ppm.

^{13}C NMR (101 MHz, $CDCl_3$) δ 169.4 ($C(O)OC(CH_3)_3$), 153.9 (C(O)NH), 141.0 (ArC), 136.6 (ArC), 129.3 (ArCH), 128.8 (ArCH), 127.6 (ArCH), 126.9 (ArCH), 126.0 (ArCH), 122.1 (ArCH), 121.3 (ArC), 114.5 (ArCH), 81.3 ($OC(CH_3)_3$), 60.1 (CH), 41.2 (CH_2), 28.0 ($OC(CH_3)_3$) ppm.

HRMS (ESI) m/z calcd. for $C_{20}H_{23}N_2O_3$ ($[M+H]^+$) 339.1703, found 339.1704

FT-IR (thin film) ν_{max} 3020, 2453, 2160, 2030, 1977, 1677, 1422, 1215, 909, 745, 669, 628 cm^{-1}.

m.p.: 200-202 °C

$[\alpha]_D^{25}$ = -2.5 (c=2.00, $CHCl_3$).

Chiral HPLC: Chiralcel OD, hexane/isopropanol = 95/5, 1.0 ml/min, λ = 254 nm, t_R (major) = 36.6 min, t_R (minor) = 57.4 min.

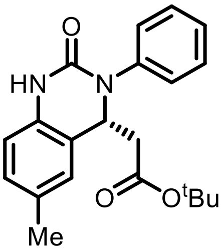

***tert*-Butyl (*R*)-2-(6-methyl-2-oxo-3-phenyl-1,2,3,4-tetrahydroquinazolin-4-yl)acetate (2b)** was prepared following **General Procedure C** (variation from standard conditions: reaction carried out at 80 °C), using ***tert*-butyl (*E*)-3-(5-methyl-2-(3-phenylureido)phenyl)acrylate (1b)** (70.5 mg, 0.2 mmol, 1.0 eq). Purification by silica gel chromatography (pentane/EtOAc = 7/3) afforded the title compound as an off-white solid (69.8 mg, 99%, 91:9 er).

^{1}H NMR (400 MHz, CDCl$_3$) δ 8.60 (s, 1H, NH), 7.44 – 7.36 (m, 4H, Ar-H), 7.28 (ddd, *J* = 5.7, 4.5, 2.6 Hz, 1H, Ar-H), 7.00 – 6.89 (m, 2H, Ar-H), 6.71 – 6.61 (m, 1H, Ar-H), 5.15 (dd, *J* = 8.6, 4.5 Hz, 1H, CH), 2.82 – 2.62 (m, 2H, CH$_2$), 2.24 (s, 3H, CH$_3$), 1.30 (s, 9H, OC(CH$_3$)$_3$) ppm.

^{13}C NMR (101 MHz, CDCl$_3$) δ 169.5 (C(O)OC(CH$_3$)$_3$), 154.0 (C(O)NH), 141.1 (ArC), 134.0 (ArC), 131.5 (ArC), 129.3 (s, 2 non-equivalent C, ArCH), 127.6 (ArCH), 126.9 (ArCH), 126.3 (ArCH), 121.1 (ArC), 114.3 (ArCH), 81.3 (OC(CH$_3$)$_3$), 60.2 (CH), 41.2 (CH$_2$), 28.0 (OC(CH$_3$)$_3$), 20.8 (CH$_3$) ppm.

HRMS (ESI) m/z calcd. for $C_{21}H_{25}N_2O_3$ ([M+H]$^+$) 353.1860, found 353.1861

FT-IR (thin film) ν_{max} 3020, 2402, 2161, 2029, 1721, 1673, 1516, 1445, 1369, 1296, 1215, 1143, 1030, 928, 745, 668, 628 cm^{-1}.

m.p.: 200-201 °C

$[\alpha]_D^{25}$ = -96.1 (c=0.55, CHCl$_3$).

Chiral HPLC: Chiralcel IA, hexane/isopropanol = 90/10, 1.0 ml/min, λ = 240 nm, t_R (major) = 19.0 min, t_R (minor) = 14.8 min.

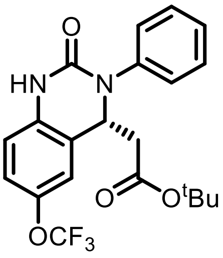

***tert*-Butyl (*R*)-2-(2-oxo-3-phenyl-6-(trifluoromethoxy)-1,2,3,4-tetrahydroquinazolin-4-yl)acetate (2c)** was prepared following **General Procedure C**, using ***tert*-butyl (*E*)-3-(2-(3-phenylureido)-5-(trifluoromethoxy)phenyl)acrylate (1c)** (84.5 mg, 0.2 mmol, 1.0 eq). Purification by silica gel chromatography (pentane/EtOAc = 7/3) afforded the title compound as a white solid (9:1 (**A**:**B**) mixture of rotamers) (82.6 mg, 98%, 93.5:6.5 er).

^{1}H NMR (500 MHz, CDCl$_3$) δ 9.41 (s, 0.1H, NH (**B**)), 9.37 (s, 0.9H, NH (**A**)), 7.49 – 7.39 (m, 4H, NAr-H), 7.33 (tt, *J* = 6.5, 1.7 Hz, 1H, NAr-H), 7.08 (d, *J* = 2.6 Hz, 1H, NHAr-H), 7.06 – 6.99 (m, 1H, NHAr-H), 6.81 (d, *J* = 8.7 Hz, 0.1H, NHAr-H (**B**)), 6.74 (d, *J* = 8.7 Hz, 0.9H, NHAr-H (**B**)), 5.21 (dd, *J* = 8.9, 4.3 Hz, 1H, CH), 2.80 (dd, *J* = 15.2, 4.3 Hz, 1H, CH_2), 2.73 (dd, *J* = 15.2, 8.9 Hz, 1H, CH_2), 1.33 (s, 8.1H, $OC(CH_3)_3$ (**A**)), 1.31 (s, 0.9H, $OC(CH_3)_3$ (**B**)) ppm.

^{13}C NMR (126 MHz, CDCl$_3$) δ 169.2 ($C(O)OC(CH_3)_3$), 153.9 (C(O)NH), 143.8 (q, *J* = 2.0 Hz, $ArCOCF_3$), 140.6 (NArC), 135.6 (NHArC), 129.5 (NArCH), 127.7 (NArCH), 127.3 (NArCH), 122.4 (NHArC), 121.9 (NHArCH), 120.6 (q, *J* = 257.1 Hz, CF_3)119.3 (NHArCH), 115.6 (NHArCH), 81.7 ($OC(CH_3)_3$), 59.7 (CH), 40.7(CH_2), 27.9 ($OC(CH_3)_3$) ppm.

^{19}F NMR (377 MHz, CDCl$_3$) δ -58.27 (s) ppm.

HRMS (ESI) m/z calcd. for $C_{21}H_{22}F_3N_2O_4$ ($[M+H]^+$) 423.1526, found 423.1526

FT-IR (thin film) ν_{max} 3235, 2981, 2160, 2032, 1726, 1680, 1596, 1510, 1480, 1457, 1397, 1369, 1254, 1215, 1162, 1075, 1008, 914, 827, 755, 698, 620 cm^{-1}.

m.p.: 96-99 °C

$[\alpha]_D^{25}$ = -119.5 (c=0.47, $CHCl_3$).

Chiral HPLC: Chiralcel OD, hexane/isopropanol = 95/5, 1.0 ml/min, λ = 240 nm, t_R (major) = 37.4 min, t_R (minor) = 28.6 min.

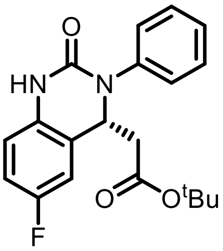

***tert*-Butyl (*R*)-2-(6-fluoro-2-oxo-3-phenyl-1,2,3,4-tetrahydroquinazolin-4-yl)acetate (2d)** was prepared following **General Procedure C** (variation from standard conditions: reaction carried out at 80 °C for 16 hours), using ***tert*-butyl (*E*)-3-(5-fluoro-2-(3-phenylureido)phenyl)acrylate (1d)** (71.3 mg, 0.2 mmol, 1.0 eq). Purification by silica gel chromatography (pentane/EtOAc = 7/3) afforded the title compound as a white solid (60.9 mg, 85%, 92.5:7.5 er).

^{1}H NMR (400 MHz, CDCl$_3$) δ 8.39 (s, 1H, N<u>H</u>), 7.47 – 7.38 (m, 4H, NAr-<u>H</u>), 7.32 (ddt, *J* = 6.9, 4.6, 1.8 Hz, 1H, NAr-<u>H</u>), 6.98 – 6.86 (m, 2H, NHAr-<u>H</u>), 6.80 – 6.67 (m, 1H, NHAr-<u>H</u>), 5.17 (dd, *J* = 8.8, 4.5 Hz, 1H, C<u>H</u>), 2.83 – 2.66 (m, 2H, C<u>H</u>$_2$), 1.33 (s, 9H, OC(C<u>H</u>$_3$)$_3$) ppm.

^{13}C NMR (101 MHz, CDCl$_3$) δ 169.2 (<u>C</u>(O)OC(CH$_3$)$_3$), 158.2 (d, *J* = 240.9 Hz, <u>C</u>F), 153.7 (<u>C</u>(O)NH), 140.7 (NAr<u>C</u>), 132.8 (d, *J* = 2.3 Hz, NHAr<u>C</u>), 129.5 (NAr<u>C</u>H), 127.6 (NAr<u>C</u>H), 127.2 (NAr<u>C</u>H), 122.7 (d, *J* = 7.6 Hz, NHAr<u>C</u>), 115.6 (d, *J* = 23.1 Hz, NHAr<u>C</u>H), 115.5 (d, *J* = 7.9 Hz, NHAr<u>C</u>H), 113.1 (d, *J* = 24.2 Hz, NHAr<u>C</u>H), 81.7 (O<u>C</u>(CH$_3$)$_3$), 59.9 (d, *J* = 1.8 Hz, <u>C</u>H), 40.8 (<u>C</u>H$_2$), 28.0 (OC(<u>C</u>H$_3$)$_3$) ppm.

^{19}F NMR (377 MHz, CDCl$_3$) δ -121.25 (td, *J* = 8.5, 4.6 Hz) ppm.

HRMS (ESI) m/z calcd. for $C_{20}H_{22}FN_2O_3$ ($[M+H]^+$) 357.1609, found 357.1610

FT-IR (thin film) ν_{max} 3020, 2402, 2160, 2028, 1721, 1675, 1509, 1446, 1370, 1298, 1243, 1215, 1138, 1030, 925, 745, 668, 625 cm^{-1}.

m.p.: 131-135 °C

$[\alpha]_D^{25}$ = -129.9 (c=0.83, CHCl$_3$).

Chiral HPLC: Chiralcel IA, hexane/isopropanol = 95/5, 1.0 ml/min, λ = 220 nm, t_R (major) = 91.0 min, t_R (minor) = 75.2 min.

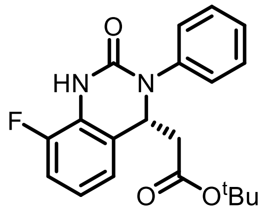

***tert*-Butyl (*R*)-2-(8-fluoro-2-oxo-3-phenyl-1,2,3,4-tetrahydroquinazolin-4-yl)acetate (2e)** was prepared following **General Procedure C**, (variation from standard conditions: reaction carried out at 40 °C for 30h), using ***tert*-butyl (*E*)-3-(3-fluoro-2-(3-phenylureido)phenyl)acrylate (1e)** (71.3 mg, 0.2 mmol, 1.0 eq). Purification by silica gel chromatography (pentane/EtOAc = 4/1) afforded the title compound as a white solid (70.6 mg, 99%, 82:18 er).

^{1}H NMR (400 MHz, CDCl$_3$) δ 7.49 – 7.37 (m, 5H, Ar-H and NH), 7.30 (ddt, *J* = 6.8, 5.7, 2.6 Hz, 1H, Ar-H), 7.03 (ddd, *J* = 10.0, 8.0, 1.4 Hz, 1H, Ar-H), 7.00 – 6.97 (m, 1H, Ar-H), 6.92 (td, *J* = 8.0, 5.0 Hz, 1H, Ar-H), 5.26 (dd, *J* = 8.4, 4.4 Hz, 1H, CH), 2.83 – 2.66 (m, 2H, CH$_2$), 1.33 (s, 9H, OC(CH$_3$)$_3$) ppm.

^{13}C NMR (101 MHz, CDCl$_3$) δ 169.1 (C(O)OC(CH$_3$)$_3$), 152.5 (C(O)NH), 149.2 (d, *J* = 243.9 Hz, CF), 140.6 (ArC), 129.5 (ArCH), 127.6 (ArCH), 127.3 (ArCH), 125.1 (d, *J* = 13.6 Hz, ArC), 123.6 (d, *J* = 2.1 Hz, ArC), 122.2 (d, *J* = 6.8 Hz, ArCH), 121.5 (d, *J* = 3.6 Hz, ArCH), 114.9 (d, *J* = 17.5 Hz, ArCH), 81.6 (OC(CH$_3$)$_3$), 60.0 (d, *J* = 2.1 Hz, CH), 41.0 (CH$_2$), 28.0 (OC(CH$_3$)$_3$) ppm.

^{19}F NMR (377 MHz, CDCl$_3$) δ -135.54 (td, *J* = 12.5, 10.0, 5.0 Hz) ppm.

HRMS (ESI) m/z calcd. for $C_{20}H_{22}FN_2O_3$ ([M+H]$^+$) 357.1609, found 357.1610

FT-IR (thin film) ν_{max} 3220, 2160, 2032, 1723, 1675, 1632, 1595, 1511, 1422, 1367, 1292, 1256, 1145, 1074, 1007, 922, 757, 703 621 cm^{-1}.

m.p.: 56-58 °C

$[\alpha]_D^{25}$ = -42.9 (c=0.42, CHCl$_3$).

Chiral HPLC: Chiralcel OD, hexane/isopropanol = 95/5, 1.0 ml/min, λ = 240 nm, t_R (major) = 29.9 min, t_R (minor) = 35.9 min.

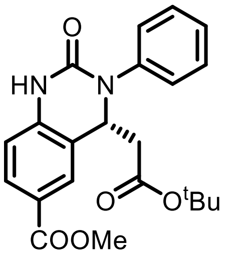

Methyl (*R*)-4-(2-(*tert*-butoxy)-2-oxoethyl)-2-oxo-3-phenyl-1,2,3,4-tetrahydroquinazoline-6-carboxylate (2f) was prepared following **General Procedure C** (variation from standard conditions: reaction carried out for 48 hours), using **methyl (*E*)-3-(3-(*tert*-butoxy)-3-oxoprop-1-en-1-yl)-4-(3-phenylureido)benzoate (1f)** (79.3 mg, 0.2 mmol, 1.0 eq). Purification by silica gel chromatography (pentane/EtOAc = 3/2) afforded the title compound as a white solid (70.6 mg, 89%, 94.5:5.5 er).

^{1}H NMR (400 MHz, CDCl$_3$) δ 8.99 (s, 1H, NH), 7.87 (d, *J* = 8.0 Hz, 2H, NHAr-H), 7.49 – 7.39 (m, 4H, NAr-H)), 7.36 – 7.31 (m, 1H, NAr-H)), 6.87 – 6.73 (m, 1H, NHAr-H), 5.26 (dd, *J* = 8.4, 4.5 Hz, 1H, CH), 3.87 (s, 3H, OCH$_3$), 2.92 – 2.53 (m, 2H, CH$_2$), 1.32 (s, 9H, OC(CH$_3$)$_3$) ppm.

^{13}C NMR (101 MHz, CDCl$_3$) δ 168.9 (C(O)), 166.5 (C(O)), 153.4 (C(O)NH), 140.7 (ArC), 140.5 (ArC), 130.8 (NHArCH), 129.5 (NArCH), 127.9 (NHArCH), 127.8 (NArCH), 127.4 (NArCH), 124.0 (ArC), 120.9 (ArC), 114.3 (NHArCH), 81.8 (OC(CH$_3$)$_3$), 60.0 (CH), 52.1 (OCH$_3$), 41.4 (CH$_2$), 28.0 (OC(CH$_3$)$_3$) ppm.

HRMS (ESI) m/z calcd. for $C_{22}H_{25}N_2O_5$ ($[M+H]^+$) 397.1758, found 397.1758

FT-IR (thin film) ν_{max} 3218, 3066, 2950, 2545, 2161, 2029, 1977, 1719, 1679, 1617, 1606 1494, 1456, 1434, 1399, 1368, 1290, 1262, 1199, 1144, 1104, 1009, 958, 915, 844, 758, 698, 652, 615 cm^{-1}.

m.p.: 174-176 °C

$[\alpha]_D^{25}$ = -79.9 (c=0.85, CHCl$_3$).

Chiral HPLC: Chiralcel AD-H, hexane/isopropanol = 90/10, 1.0 ml/min, λ = 220 nm, t_R (major) = 40.0 min, t_R (minor) = 48.3 min.

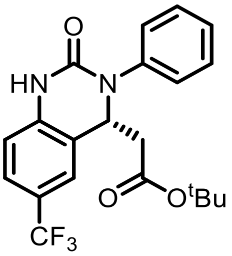

***tert*-Butyl (*R*)-2-(2-oxo-3-phenyl-6-(trifluoromethyl)-1,2,3,4-tetrahydroquinazolin-4-yl)acetate (2g)** was prepared following **General Procedure C**, using ***tert*-butyl (*E*)-3-(2-(3-phenylureido)-5-(trifluoromethyl)phenyl)acrylate (1g)** (81.3 mg, 0.2 mmol, 1.0 eq). Purification by silica gel chromatography (pentane/EtOAc =7/3) afforded the title compound as a white solid (81.2 mg, 99%, 94:6 er).

^{1}H NMR (400 MHz, CDCl$_3$) δ 9.52 (s, 1H, N<u>H</u>), 7.52 – 7.32 (m, 7H, Ar-<u>H</u>), 6.81 (d, *J* = 8.3 Hz, 1H, Ar-<u>H</u>), 5.26 (dd, *J* = 9.1, 4.3 Hz, 1H, C<u>H</u>), 2.82 (dd, *J* = 14.9, 4.3 Hz, 1H, C<u>H</u>$_2$), 2.72 (dd, *J* = 14.9, 9.1 Hz, 1H, C<u>H</u>$_2$), 1.32 (s, 9H, OC(C<u>H</u>$_3$)$_3$) ppm.

^{13}C NMR (101 MHz, CDCl$_3$) δ 169.0 (<u>C</u>(O)OC(CH$_3$)$_3$), 153.8 (<u>C</u>(O)NH), 140.5 (Ar<u>C</u>), 139.7 (Ar<u>C</u>), 129.5 (Ar<u>C</u>H), 127.7 (Ar<u>C</u>H), 127.4 (Ar<u>C</u>H), 126.1 (q, *J* = 3.7 Hz, Ar<u>C</u>H), 124.3 (q, *J* = 32.7 Hz, Ar<u>C</u>CF$_3$), 124.25 (q, *J* = 272.3 Hz, CF$_3$), 123.4 (q, *J* = 4.0 Hz, Ar<u>C</u>H), 121.4 (Ar<u>C</u>), 114.8 (Ar<u>C</u>H), 81.8 (O<u>C</u>(CH$_3$)$_3$), 59.9 (<u>C</u>H), 40.9 (<u>C</u>H$_2$), 27.9 (OC(<u>C</u>H$_3$)$_3$) ppm.

^{19}F NMR (377 MHz, CDCl$_3$) δ -61.74 (s) ppm.

HRMS (ESI) m/z calcd. for $C_{21}H_{22}F_3N_2O_3$ ([M+H]$^+$) 407.1577, found 407.1577

FT-IR (thin film) ν_{max} 3020, 2161, 2035, 1977, 1722, 1681, 1626, 1496, 1455, 1406, 1370, 1330, 1295, 1215, 1165, 1145, 1128, 1074, 924, 837, 746, 697, 668 cm^{-1}.

m.p.: 80-83 °C

$[\alpha]_D^{25}$ = -115.6 (c=0.50, CHCl$_3$).

Chiral HPLC: Chiralcel OD, hexane/isopropanol = 95/5, 1.0 ml/min, λ = 240 nm, t_R (major) = 35.1 min, t_R (minor) = 26.5 min.

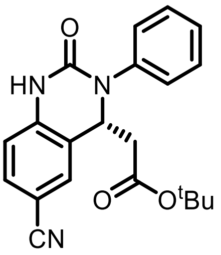

***tert*-Butyl (*R*)-2-(6-cyano-2-oxo-3-phenyl-1,2,3,4-tetrahydroquinazolin-4-yl)acetate (2h)** was prepared following **General Procedure C**, using ***tert*-butyl (*E*)-3-(5-cyano-2-(3-phenylureido)phenyl)acrylate (1h)** (72.7 mg, 0.2 mmol, 1.0 eq). Purification by silica gel chromatography (pentane/EtOAc = 13/7) afforded the title compound as an off-white solid (6:1 (**A**:**B**) mixture of rotamers) (64.0 mg, 88%, 87.5:12.5 er).

^{1}H NMR (400 MHz, CDCl$_3$) δ 9.66 (s, 0.14H, NH (**B**)), 9.62 (s, 0.86H, NH (**A**)), 7.52 – 7.44 (m, 3H, Ar-H), 7.43 – 7.34 (m, 4H, Ar-H), 6.81 (d, *J* = 8.3 Hz, , 0.14H, Ar-H (**B**)), 6.74 (d, *J* = 8.3 Hz, 0.86H, Ar-H (**A**)), 5.21 (dd, J = 8.6, 4.4 Hz, 1H, CH), 2.77 (dd, *J* = 15.0, 4.4 Hz, 1H, CH$_2$), 2.69 (dd, *J* = 15.0, 8.6 Hz, 1H, CH$_2$), 1.33 (s, 7.7H, OC(CH$_3$)$_3$ (**A**)), 1.31 (s, 1.3H, OC(CH$_3$)$_3$ (**B**)) ppm.

^{13}C NMR (101 MHz, CDCl$_3$) δ 168.8 (C(O)OC(CH$_3$)$_3$), 153.3 (C(O)NH), 140.7 (ArC), 140.1 (ArC), 133.1 (ArCH), 130.2 (ArCH), 129.6 (ArCH), 127.8 (ArCH), 127.7 (ArCH), 121.8 (ArC), 118.9 (ArC), 115.3 (ArCH), 105.2 (ArCN), 82.0 (OC(CH$_3$)$_3$), 59.5 (CHCH$_2$), 40.9 (CHCH$_2$), 28.0 (OC(CH$_3$)$_3$) ppm.

HRMS (ESI) m/z calcd. for $C_{21}H_{22}N_3O_3$ ([M+H]$^+$) 364.1656, found 364.1656

FT-IR (thin film) ν_{max} 3242, 2978, 2934, 2533, 2224, 2160, 2032, 1977, 1722, 1680, 1615, 1597, 1498 1456, 1402, 1367, 1295, 1146, 1030, 959, 900, 836, 756, 699, 627 cm^{-1}.
m.p.: 95-97 °C

[α]$_D$^{25} = -88.2 (c=0.69, CHCl$_3$).

Chiral HPLC: Chiralcel IA, hexane/isopropanol = 85/15, 1.0 ml/min, λ = 220 nm, t_R (major) = 19.1 min, t_R (minor) = 25.1 min.

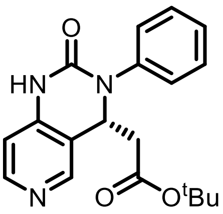

***tert*-Butyl (*R*)-2-(2-oxo-3-phenyl-1,2,3,4-tetrahydropyrido[4,3-d]pyrimidin-4-yl)acetate (2i)** was prepared following **General Procedure C**, using ***tert*-butyl (*E*)-3-(4-(3-phenylureido)pyridin-3-yl)acrylate (1i)** (67.9 mg, 0.2 mmol, 1.0 eq). Purification by silica gel chromatography (pentane/EtOAc = 3/7) afforded the title compound as a white solid (8:1 (**A**:**B**) mixture of rotamers) (66.1 mg, 97%, 89.5:10.5 er).

^{1}H NMR (400 MHz, CDCl$_3$) δ 9.70 (s, 0.11H, NH (**B**)), 9.66 (s, 0.89H, NH (**A**)), 8.33 (s, 1H, Ar-H), 8.24 (d, *J* = 5.4 Hz, 1H, Ar-H), 7.51 – 7.37 (m, 4H, Ar-H), 7.34 (ddt, *J* = 8.5, 6.8, 1.5 Hz, 1H, Ar-H), 6.65 (d, *J* = 5.4 Hz, 0.11H, Ar-H (**B**)), 6.58 (d, *J* = 5.4 Hz, 0.89H, Ar-H (**A**)), 5.24 (dd, *J* = 8.1, 4.4 Hz, 1H, CH), 2.80 – 2.60 (m, 2H, CH$_2$), 1.31 (s, 8H, OC(CH$_3$)$_3$ (**A**)), 1.28 (s, 1H, OC(CH$_3$)$_3$ (**B**)) ppm.

^{13}C NMR (101 MHz, CDCl$_3$) δ 168.8 (C(O)OC(CH$_3$)$_3$), 153.2 (C(O)NH), 149.8 (ArCH), 146.7 (ArCH), 143.8 (ArC), 140.2 (ArC), 129.5 (ArCH), 127.9 (ArCH), 127.6 (ArCH), 116.9 (ArC), 109.2 (ArCH), 81.8 (OC(CH$_3$)$_3$), 57.9 (CHCH$_2$), 41.3 (CHCH$_2$), 27.9 (OC(CH$_3$)$_3$) ppm.

HRMS (ESI) m/z calcd. for $C_{19}H_{22}N_3O_3$ ([M+H]$^+$) 340.1656, found 340.1657

FT-IR (thin film) ν_{max} 3243, 3063, 2977, 2513, 2160, 2029, 1977, 1723, 1682, 1601, 1494, 1446, 1410, 1367, 1319, 1291, 1141, 1057, 955, 840, 755, 699, 642 cm^{-1}.

m.p.: 108-110 °C

$[\alpha]_D^{25}$ = -120.1 (c=0.74, CHCl$_3$).

Chiral HPLC: Chiralcel IA, hexane/isopropanol = 85/15, 1.0 ml/min, λ = 280 nm, t_R (major) = 23.5 min, t_R (minor) = 16.7 min.

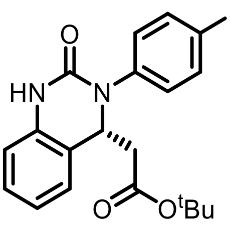

***tert*-Butyl (*R*)-2-(3-(4-iodophenyl)-2-oxo-1,2,3,4-tetrahydroquinazolin-4-yl)acetate (2j)** was prepared following **General Procedure C**, using ***tert*-butyl (*E*)-3-(2-(3-(4-iodophenyl)ureido)phenyl)acrylate (1j)** (92.9 mg, 0.2 mmol, 1.0 eq). Purification by silica gel chromatography (pentane/EtOAc = 7/3) afforded the title compound as a white solid (88.5 mg, 96%, 93:7 er).

^{1}H NMR (400 MHz, $CDCl_3$) δ 8.50 (s, 1H, NH), 7.78 – 7.70 (m, 2H, Ar-H), 7.23 – 7.14 (m, 4H, Ar-H), 6.97 (td, *J* = 7.5, 1.1 Hz, 1H, Ar-H), 6.76 (dd, *J* = 7.9, 1.1 Hz, 1H, Ar-H), 5.21 (dd, *J* = 8.2, 4.7 Hz, 1H, CH), 2.79 – 2.61 (m, 2H, CH_2), 1.32 (s, 9H, $OC(CH_3)_3$) ppm.

^{13}C NMR (101 MHz, $CDCl_3$) δ 169.2 ($C(O)OC(CH_3)_3$), 153.6 (C(O)NH), 140.8 (ArC), 138.4 (ArCH), 136.3 (ArC), 129.4 (ArCH), 129.0 (ArCH), 126.0 (ArCH), 122.4 (ArCH), 121.2 (ArC), 114.5 (ArCH), 91.7 (CI), 81.5 ($OC(CH_3)_3$), 60.0 ($CHCH_2$), 41.2 ($CHCH_2$), 28.0 ($OC(CH_3)_3$) ppm.

HRMS (ESI) m/z calcd. for $C_{20}H_{22}{}^{127}IN_2O_3$ ($[M+H]^+$) 465.0670, found 465.0669

FT-IR (thin film) ν_{max} 3247, 3064, 2978, 2932, 2543, 2361, 2341, 2161, 2025, 1977, 1721, 1675, 1605, 1504, 1485, 1449, 1413, 1394, 1368, 1280, 1207, 1145, 1075, 1009, 948, 828, 804, 756, 724 cm^{-1}.

m.p.: 102-105 °C

$[\alpha]_D^{25}$ = -23.0 (c=1.12, CH_3OH).

Chiral HPLC: Chiralcel AD-H, hexane/isopropanol = 70/30, 1.0 ml/min, λ = 220 nm, t_R (major) = 9.8 min, t_R (minor) = 11.0 min.

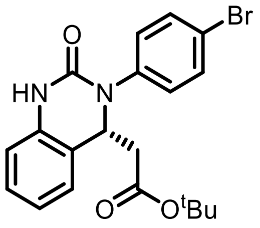

***tert*-Butyl (*R*)-2-(3-(4-bromophenyl)-2-oxo-1,2,3,4-tetrahydroquinazolin-4-yl)acetate (2k)** was prepared following **General Procedure C**, using ***tert*-butyl (*E*)-3-(2-(3-(4-bromophenyl)ureido)phenyl)acrylate (1k)** (83.5 mg, 0.2 mmol, 1.0 eq). Purification by silica gel chromatography (pentane/EtOAc = 7/3) afforded the title compound as a white solid (83.0 mg, 99%, 92:8 er).

^{1}H NMR (400 MHz, $CDCl_3$) δ 8.56 (s, 1H, NH), 7.59 – 7.51 (m, 2H, Ar-H), 7.37 – 7.29 (m, 2H, Ar-H), 7.20 (td, *J* = 7.6, 1.4 Hz, 1H, Ar-H), 7.16 (dd, *J* = 7.6, 1.4 Hz, 1H, Ar-H), 7.01 – 6.92 (m, 1H, Ar-H), 6.76 (dd, *J* = 7.9, 1.1 Hz, 1H, Ar-H), 5.21 (dd, *J* = 8.2, 4.7 Hz, 1H, CH), 2.78 – 2.63 (m, 2H, CH_2), 1.32 (s, 9H, $OC(CH_3)_3$) ppm.

^{13}C NMR (101 MHz, $CDCl_3$) δ 169.3 ($C(O)OC(CH_3)_3$), 153.6 (C(O)NH), 140.0 (ArC), 136.4 (ArC), 132.4 (ArCH), 129.2 (ArCH), 129.0 (ArCH), 126.0 (ArCH), 122.4 (ArCH), 121.2 (ArC), 120.4 (ArC), 114.5 (ArCH), 81.5 ($OC(CH_3)_3$), 60.0 ($CHCH_2$), 41.2 ($CHCH_2$), 28.0 ($OC(CH_3)_3$) ppm.

HRMS (ESI) m/z calcd. for $C_{20}H_{22}{}^{80}BrN_2O_3$ ($[M+H]^+$) 417.0808, found 417.0809

FT-IR (thin film) ν_{max} 3236, 3065, 2979, 2929, 2544, 2360, 2341, 2161, 2031, 1977, 1722, 1674, 1604, 1489, 1452, 1418, 1398, 1367, 1294, 1256, 1142, 1071, 1034, 1013, 956, 831, 804, 756, 726, 624 cm^{-1}.

m.p.: 72-74 °C

$[\alpha]_D^{25}$ = -59.51 (c=0.79, CH_3OH).

Chiral HPLC: Chiralcel AD-H, hexane/isopropanol = 70/30, 1.0 ml/min, λ = 240 nm, t_R (major) = 8.8 min, t_R (minor) = 10.5 min.

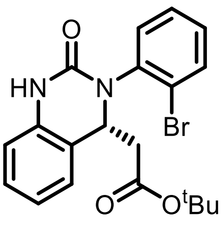

***tert*-Butyl (*R*)-2-(3-(2-bromophenyl)-2-oxo-1,2,3,4-tetrahydroquinazolin-4-yl)acetate (2l)** was prepared following **General Procedure C**, (variation from standard conditions: reaction carried out with 5 mol% **Cat.AH** for 72h), using ***tert*-butyl (*E*)-3-(2-(3-(2-bromophenyl)ureido)phenyl)acrylate (1l)** (167.0 mg, 0.4 mmol, 1.0 eq). Purification by silica gel chromatography (pentane/EtOAc = 1/1) afforded the title compound as an off-white solid (6.8:1 (**A**:**B**) mixture of rotamers) (151.2 mg, 91%, 93:7 er).

^{1}H NMR (400 MHz, CDCl$_3$) δ 7.92 (s, 0.88H, Ar-H (**A**)), 7.86 (s, 0.12H, Ar-H (**B**)), 7.69 (dt, *J* = 8.0, 2.5 Hz, 1H, Ar-H), 7.57 (dd, *J* = 8.0, 1.7 Hz, 1H, Ar-H), 7.46 – 7.31 (m, 1H, Ar-H), 7.25 – 7.13 (m, 2H, Ar-H), 6.98 (td, *J* = 7.5, 1.2 Hz, 1H, Ar-H), 6.83 – 6.66 (m, 1H, Ar-H), 5.26 (dd, *J* = 9.1, 4.6 Hz, 0.12H, CH (**B**)), 5.12 (dd, *J* = 7.3, 5.3 Hz, 0.88H, CH (**A**)), 3.02 (dd, *J* = 15.5, 4.6 Hz, 0.12H, CH_2 (**B**)), 2.79 – 2.65 (m, 1.88H, CH_2 (**A**)), 1.31 (s, 7.8H, $OC(CH_3)_3$ (**A**)), 1.30 (s, 1.2H, $OC(CH_3)_3$ (**B**)) ppm.

^{13}C NMR (101 MHz, CDCl$_3$) δ 169.3 ($C(O)OC(CH_3)_3$), 153.4 (C(O)NH), 139.0 (ArC), 136.3 (ArC), 134.0 (ArCH), 133.0 (ArCH), 129.6 (ArCH), 128.8 (ArCH), 128.2 (ArCH), 126.3 (ArCH), 122.8 (ArC), 122.5 (ArCH), 121.2 (ArC), 114.4 (ArCH), 81.4 ($OC(CH_3)_3$), 58.9 ($CHCH_2$), 41.2 ($CHCH_2$), 28.0 ($OC(CH_3)_3$) ppm.

HRMS (ESI) m/z calcd. for $C_{20}H_{22}BrN_2O_3$ ($[M+H]^+$) 417.0808, found 417.0807

FT-IR (thin film) ν_{max} 3020, 2161, 2027, 1978, 1722, 1678, 1603, 1476, 1450, 1369, 1287, 1215, 1143, 1029, 929, 745, 668, 627 cm^{-1}.

m.p.: 80-82 °C

$[\alpha]_D^{25}$ = -190.9 (c=0.45, $CHCl_3$).

Chiral HPLC: Chiralcel AS-H, hexane/isopropanol = 85/15, 1.0 ml/min, λ = 240 nm, t_R (major) = 21.1 min, t_R (minor) = 35.6 min.

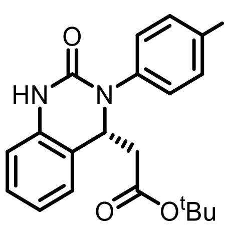

***tert*-Butyl (*R*)-2-(3-(4-fluorophenyl)-2-oxo-1,2,3,4-tetrahydroquinazolin-4-yl)acetate (2m)** was prepared following **General Procedure C**, using ***tert*-butyl (*E*)-3-(2-(3-(4-fluorophenyl)ureido)phenyl)acrylate (1m)** (71.3 mg, 0.2 mmol, 1.0 eq). Purification by silica gel chromatography (pentane/EtOAc = 13/7) afforded the title compound as an off-white solid (67.3 mg, 95%, 92:8 er).

^{1}H NMR (400 MHz, $CDCl_3$) δ 8.88 (s, 1H, N<u>H</u>), 7.45 – 7.36 (m, 2H, Ar-<u>H</u>), 7.24 – 7.06 (m, 4H, Ar-<u>H</u>), 6.95 (td, *J* = 7.5, 1.1 Hz, 1H, Ar-<u>H</u>), 6.83 – 6.72 (m, 1H, Ar-<u>H</u>), 5.18 (dd, *J* = 8.0, 4.7 Hz, 1H, C<u>H</u>), 2.79 – 2.61 (m, 2H, C<u>H</u>$_2$), 1.32 (s, 9H, OC(C<u>H</u>$_3$)$_3$) ppm.

^{13}C NMR (101 MHz, $CDCl_3$) δ 169.3 (<u>C</u>(O)OC(CH$_3$)$_3$), 161.3 (d, *J* = 246.5 Hz, <u>C</u>F), 154.0 (<u>C</u>(O)NH), 136.9 (d, *J* = 3.2 Hz, Ar<u>C</u>), 136.5 (Ar<u>C</u>), 129.6 (d, *J* = 8.5 Hz, Ar<u>C</u>H), 128.8 (Ar<u>C</u>H), 125.9 (Ar<u>C</u>H), 122.2 (Ar<u>C</u>H), 121.0 (Ar<u>C</u>), 116.2 (d, *J* = 22.6 Hz, Ar<u>C</u>H), 114.5 (Ar<u>C</u>H), 81.4 (O<u>C</u>(CH$_3$)$_3$), 60.3 (<u>C</u>H), 41.2 (<u>C</u>H$_2$), 28.0 (OC(<u>C</u>H$_3$)$_3$) ppm.

^{19}F NMR (377 MHz, $CDCl_3$) δ -114.80 (ddd, *J* = 13.1, 8.5, 4.9 Hz) ppm.

HRMS (ESI) m/z calcd. for $C_{20}H_{22}FN_2O_3$ ([M+H]$^+$) 357.1609, found 357.1609

FT-IR (thin film) ν_{max} 3699, 3236, 3071, 2929, 2552, 2160, 2032, 1977, 1722, 1675, 1602, 1509, 1453, 1412, 1368, 1294, 1217, 1143, 1016, 841, 755, 661, 616 cm^{-1}.

m.p.: 68-72 °C

$[\alpha]_D^{25}$ = -80.5 (c=0.55, $CHCl_3$).

Chiral HPLC: Chiralcel OD, hexane/isopropanol = 95/5, 1.0 ml/min, λ = 254 nm, t_R (major) = 19.3 min, t_R (minor) = 24.8 min.

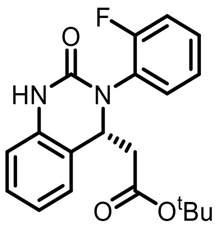

***tert*-Butyl (*R*)-2-(3-(2-fluorophenyl)-2-oxo-1,2,3,4-tetrahydroquinazolin-4-yl)acetate (2n)** was prepared following **General Procedure C**, using ***tert*-butyl (*E*)-3-(2-(3-(2-fluorophenyl)ureido)phenyl)acrylate (1n)** (71.3 mg, 0.2 mmol, 1.0 eq). Purification by silica gel chromatography (pentane/EtOAc = 7/3) afforded the title compound as a white solid (68.8 mg, 96%, 93.5:6.5 er).

^{1}H NMR (400 MHz, $CDCl_3$) δ 8.75 (s, 1H, NH), 7.47 (td, *J* = 7.6, 1.8 Hz, 1H, Ar-H), 7.39 – 7.29 (m, 1H, Ar-H), 7.26 – 7.11 (m, 4H, Ar-H), 6.95 (td, *J* = 7.6, 1.1 Hz, 1H, Ar-H), 6.85 – 6.70 (m, 1H, Ar-H), 5.13 (dd, *J* = 8.2, 4.9 Hz, 1H, CH), 2.82 – 2.75 (m, 1H, CH_2), 2.70 (dd, *J* = 15.0, 8.2 Hz, 1H, CH_2), 1.31 (s, 9H, $OC(CH_3)_3$) ppm.

^{13}C NMR (101 MHz, $CDCl_3$) δ 169.5 ($C(O)OC(CH_3)_3$), 158.7 (d, *J* = 250.4 Hz, CF), 153.5 (C(O)NH), 136.5 (ArC), 131.1 (ArCH), 129.3 (d, *J* = 8.0 Hz, ArCH), 128.8 (ArCH), 128.3 (d, *J* = 12.2 Hz, ArC), 126.0 (ArCH), 124.7 (d, *J* = 3.7 Hz, ArCH), 122.2 (ArCH), 121.1 (ArC), 116.8 (d, *J* = 20.3 Hz, ArCH), 114.6 (ArCH), 81.3 ($OC(CH_3)_3$), 59.8 (d, *J* = 1.8 Hz, CH), 41.5 (CH_2), 28.0 ($OC(CH_3)_3$) ppm.

^{19}F NMR (377 MHz, $CDCl_3$) δ -118.90 (s) ppm (H-F coupling not observed).

HRMS (ESI) m/z calcd. for $C_{20}H_{22}FN_2O_3$ ($[M+H]^+$) 357.1609, found 357.1609

FT-IR (thin film) ν_{max} 3219, 3069, 2978, 2554, 2160, 2029, 1723, 1680, 1603, 1501, 1453, 1420, 1367, 1289, 1257, 1216, 1143, 1106, 1034, 960, 846, 824, 754, 686 cm^{-1}.

m.p.: 168-170 °C

$[\alpha]_D^{25}$ = -112.7 (c=0.75, $CHCl_3$).

Chiral HPLC: Chiralcel OD, hexane/isopropanol = 90/10, 1.0 ml/min, λ = 240 nm, t_R (major) = 10.6 min, t_R (minor) = 14.7 min.

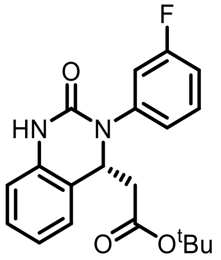

***tert*-Butyl (*R*)-2-(3-(3-fluorophenyl)-2-oxo-1,2,3,4-tetrahydroquinazolin-4-yl)acetate (2o)** was prepared following **General Procedure C**, using ***tert*-butyl (*E*)-3-(2-(3-(3-fluorophenyl)ureido)phenyl)acrylate (1o)** (71.3 mg, 0.2 mmol, 1.0 eq). Purification by silica gel chromatography (pentane/EtOAc = 3/1) afforded the title compound as a white solid (69.8 mg, 97%, 91.5:8.5 er).

^{1}H NMR (400 MHz, $CDCl_3$) δ 9.02 (s, 1H, N<u>H</u>), 7.46 – 7.34 (m, 1H, Ar-<u>H</u>), 7.28 – 7.20 (m, 2H, Ar-<u>H</u>), 7.20 – 7.14 (m, 2H, Ar-<u>H</u>), 7.05 – 6.92 (m, 2H, Ar-<u>H</u>), 6.80 (dd, *J* = 7.9, 1.1 Hz, 1H, Ar-<u>H</u>), 5.26 (dd, *J* = 8.2, 4.6 Hz, 1H, C<u>H</u>), 2.78 (dd, *J* = 14.9, 4.7 Hz, 1H, C<u>H</u>$_2$), 2.70 (dd, *J* = 14.9, 8.2 Hz, 1H, C<u>H</u>$_2$), 1.33 (s, 9H, OC(C<u>H</u>$_3$)$_3$) ppm.

^{13}C NMR (101 MHz, $CDCl_3$) δ 169.3 (<u>C</u>(O)OC(CH$_3$)$_3$), 162.9 (d, *J* = 246.5 Hz, Ar<u>C</u>), 153.8 (<u>C</u>(O)NH), 142.5 (d, *J* = 9.9 Hz, Ar<u>C</u>), 136.3 (Ar<u>C</u>), 130.3 (d, *J* = 9.2 Hz, Ar<u>C</u>H), 128.9 (Ar<u>C</u>H), 125.9 (Ar<u>C</u>H), 122.6 (d, *J* = 3.2 Hz, Ar<u>C</u>H), 122.3 (Ar<u>C</u>H), 121.2 (Ar<u>C</u>), 114.9 (d, *J* = 23.3 Hz, Ar<u>C</u>H), 114.6 (Ar<u>C</u>H), 113.8 (d, *J* = 20.9 Hz, Ar<u>C</u>H), 81.5 (O<u>C</u>(CH$_3$)$_3$), 60.0 (<u>C</u>H), 41.3 (<u>C</u>H$_2$), 28.0 (OC(<u>C</u>H$_3$)$_3$) ppm.

^{19}F NMR (377 MHz, $CDCl_3$) δ -111.62 (td, *J* = 10.4, 9.7, 7.0 Hz) ppm.

HRMS (ESI) m/z calcd. for $C_{20}H_{22}FN_2O_3$ ($[M+H]^+$) 357.1609, found 357.1609

FT-IR (thin film) ν_{max} 3233, 3068, 2978, 2930, 2539, 2160, 2031, 1977, 1723, 1678, 1605, 1487, 1454, 1416, 1368, 1288, 1209, 1141, 1038, 1014, 870, 844, 755, 697, 634 cm^{-1}.

m.p.: 134-139 °C

$[\alpha]_D^{25}$ = -83.2 (c=0.66, $CHCl_3$).

Chiral HPLC: Chiralcel OD, hexane/isopropanol = 95/5, 1.0 ml/min, λ = 240 nm, t_R (major) = 13.4 min, t_R (minor) = 18.8 min.

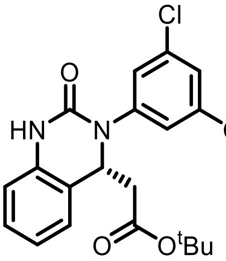

***tert*-Butyl (*R*)-2-(3-(3,5-dichlorophenyl)-2-oxo-1,2,3,4-tetrahydroquinazolin-4-yl)acetate (2p)** was prepared following **General Procedure C**, using ***tert*-butyl (*E*)-3-(2-(3-(3,5-dichlorophenyl)ureido)phenyl)acrylate (1p)** (81.5 mg, 0.2 mmol, 1.0 eq). Purification by silica gel chromatography (pentane/EtOAc = 17/3) afforded the title compound as a white solid (6:1 (**A**:**B**) mixture of rotamers) (79.9 mg, 98%, 87.5:12.5 er).

^{1}H NMR (400 MHz, $CDCl_3$) δ 8.62 (s, 1H, NH), 7.38 (d, *J* = 1.8 Hz, 2H, Ar-H), 7.29 (t, *J* = 1.8 Hz, 1H, Ar-H), 7.25 – 7.20 (m, 1H, Ar-H), 7.17 (dd, *J* = 7.8, 1.4 Hz, 1H, Ar-H), 7.00 (tdd, *J* = 7.5, 2.7, 1.1 Hz, 1H, Ar-H), 6.79 (dd, *J* = 8.0, 1.1 Hz, 1H, Ar-H), 5.23 (dd, *J* = 8.0, 4.8 Hz, 1H, CH), 2.80 – 2.61 (m, 2H, CH_2), 1.34 (s, 7.75H, $OC(CH_3)_3$ (**A**)), 1.33 (s, 1.25H, $OC(CH_3)_3$ (**B**)) ppm.

^{13}C NMR (101 MHz, $CDCl_3$) δ 169.1 ($C(O)OC(CH_3)_3$), 153.4 (C(O)NH), 142.9 (ArC), 136.0 (ArC), 135.3 (ArC), 129.2 (ArCH), 127.1 (ArCH), 126.0 (ArCH), 125.96 (ArCH), 122.7 (ArCH), 121.1 (ArC), 114.6 (ArCH), 81.8 ($OC(CH_3)_3$), 60.0 ($CHCH_2$), 41.4 ($CHCH_2$), 28.0 ($OC(CH_3)_3$) ppm.

HRMS (ESI) m/z calcd. for $C_{20}H_{21}Cl_2N_2O_3$ ($[M+H]^+$) 407.0924, found 407.0924

FT-IR (thin film) ν_{max} 3229, 3074, 2979, 2930, 2361, 2160, 2031, 1977, 1725, 1681, 1605, 1586, 1570, 1504, 1453, 1414, 1368, 1302, 1142, 1036, 847, 804, 756, 684 cm^{-1}.

m.p.: 62-64 °C

$[\alpha]_D^{25}$ = -74.7 (c=0.69, CH_3OH).

Chiral HPLC: Chiralcel IA, hexane/isopropanol = 95/5, 1.0 ml/min, λ = 240 nm, t_R (major) = 25.4 min, t_R (minor) = 14.1 min.

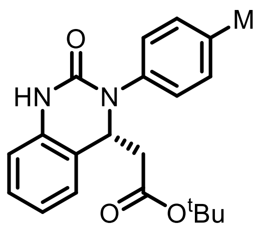

***tert*-Butyl (*R*)-2-(2-oxo-3-(p-tolyl)-1,2,3,4-tetrahydroquinazolin-4-yl)acetate (2q)** was prepared following **General Procedure C** (variation from standard conditions: reaction carried out for 48 hours), using ***tert*-butyl (*E*)-3-(2-(3-(*p*-tolyl)ureido)phenyl)acrylate (1q)** (70.5 mg, 0.2 mmol, 1.0 eq). Purification by silica gel chromatography (pentane/EtOAc = 13/7) afforded the title compound as a white solid (62.3 mg, 88%, 94:6 er).

^{1}H NMR (400 MHz, CDCl$_3$) δ 8.79 (s, 1H, NH), 7.35 – 7.28 (m, 2H, Ar-H), 7.26 – 7.21 (m, 2H, Ar-H), 7.21 – 7.12 (m, 2H, Ar-H), 6.93 (td, *J* = 7.5, 1.1 Hz, 1H, Ar-H), 6.77 (dt, *J* = 7.5, 1.1 Hz, 1H, Ar-H), 5.18 (dd, *J* = 8.5, 4.5 Hz, 1H, CH), 2.82 – 2.64 (m, 2H, CH$_2$), 2.38 (s, 3H, CH$_3$), 1.32 (s, 9H, OC(CH$_3$)$_3$) ppm.

^{13}C NMR (101 MHz, CDCl$_3$) δ 169.5 (C(O)OC(CH$_3$)$_3$), 154.1 (C(O)NH), 138.3 (ArC), 136.8 (ArC), 136.7 (ArC), 129. (ArCH)9, 128.7 (ArCH), 127.5 (ArCH), 125.9 (ArCH), 122.0 (ArCH), 121.2 (ArC), 114.5 (ArCH), 81.2 (OC(CH$_3$)$_3$), 60.2 (CH), 41.1 (CH$_2$), 28.0 (OC(CH$_3$)$_3$), 21.2 (CH$_3$)ppm.

HRMS (ESI) m/z calcd. for $C_{21}H_{25}N_2O_3$ ([M+H]$^+$) 353.1860, found 353.1861

FT-IR (thin film) ν_{max} 3225, 2160, 2031, 1722, 1675, 1603, 1514, 1454, 1367, 1291, 1144, 1076, 1007, 914, 754, 659 cm^{-1}.

m.p.: 88-91 °C

$[\alpha]_D^{25}$ = -89.3 (c=0.35, CHCl$_3$).

Chiral HPLC: Chiralcel OD, hexane/isopropanol = 95/5, 1.0 ml/min, λ = 240 nm, t_R (major) = 21.4 min, t_R (minor) = 30.0 min.

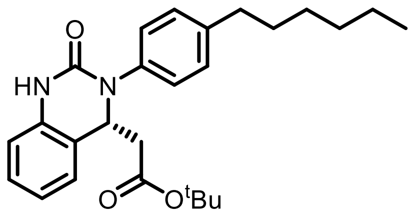

***tert*-Butyl (*R*)-2-(3-(4-hexylphenyl)-2-oxo-1,2,3,4-tetrahydroquinazolin-4-yl)acetate (2r)** was prepared following **General Procedure C**, (variation from standard conditions: reaction carried out for 48h), using ***tert*-butyl (*E*)-3-(2-(3-(4-hexylphenyl)ureido)phenyl)acrylate (1r)** (84.5 mg, 0.2 mmol, 1.0 eq). Purification by silica gel chromatography (pentane/EtOAc = 4/1) afforded the title compound as a white solid (76.7 mg, 91%, 95:5 er).

^{1}H NMR (400 MHz, $CDCl_3$) δ 8.73 (s, 1H, NH), 7.37 – 7.29 (m, 2H, Ar-H), 7.26 – 7.22 (m, 2H, Ar-H), 7.16 (dd, *J* = 8.5, 6.8 Hz, 2H, Ar-H), 6.94 (td, *J* = 7.5, 1.2 Hz, 1H, Ar-H), 6.84 – 6.73 (m, 1H, Ar-H), 5.20 (dd, *J* = 8.5, 4.5 Hz, 1H, C<u>H</u>CH_2), 2.83 – 2.67 (m, 2H, CHC<u>H</u>$_2$), 2.67 – 2.59 (m, 2H, ArC<u>H</u>$_2CH_2CH_2CH_2CH_2CH_3$), 1.70 – 1.58 (m, 2H, $ArCH_2$C<u>H</u>$_2CH_2CH_2CH_2CH_3$), 1.43-1.29 (m, 15H, OC(C<u>H</u>$_3$)$_3$ and $ArCH_2CH_2$C<u>H</u>$_2$C<u>H</u>$_2$C<u>H</u>$_2CH_3$), 0.98 – 0.87 (m, 3H, C<u>H</u>$_3$) ppm.

^{13}C NMR (101 MHz, $CDCl_3$) δ 169.5 (<u>C</u>(O)OC(CH_3)$_3$), 154.0 (<u>C</u>(O)NH), 141.7 (Ar<u>C</u>), 138.5 (Ar<u>C</u>), 136.7(Ar<u>C</u>), 129.3 (Ar<u>C</u>H), 128.7 (Ar<u>C</u>H), 127.4 (Ar<u>C</u>H), 126.0 (Ar<u>C</u>H), 122.0 (Ar<u>C</u>H), 121.4 (Ar<u>C</u>), 114.5 (Ar<u>C</u>H), 81.2 (O<u>C</u>(CH_3)$_3$), 60.2 (<u>C</u>HCH_2), 41.1 (CH<u>C</u>H$_2$), 35.7 (Ar<u>C</u>H$_2CH_2CH_2CH_2CH_2CH_3$), 31.8 ($ArCH_2$<u>C</u>H$_2CH_2CH_2CH_2CH_3$), 31.4 ($ArCH_2CH_2$<u>C</u>H$_2CH_2CH_2CH_3$), 29.1 ($ArCH_2CH_2CH_2$<u>C</u>H$_2CH_2CH_3$), 28.0 (OC(<u>C</u>H$_3$)$_3$), 22.7 ($ArCH_2CH_2CH_2CH_2$<u>C</u>H$_2CH_3$), 14.2 ($ArCH_2CH_2CH_2CH_2CH_2$<u>C</u>H$_3$) ppm.

HRMS (ESI) m/z calcd. for $C_{26}H_{35}N_2O_3$ ($[M+H]^+$) 423.2642, found 423.2642

FT-IR (thin film) ν_{max} 3216, 2928, 2857, 2160, 2031, 1724, 1676, 1604, 1512, 1451, 1413, 1367, 1282, 1143, 846, 754 cm^{-1}.

m.p.: 42-45 °C

$[\alpha]_D^{25}$ = -64.5 (c=1.28, $CHCl_3$).

Chiral HPLC: Chiralcel OD, hexane/isopropanol = 90/10, 1.0 ml/min, λ = 254 nm, t_R (major) = 8.9 min, t_R (minor) = 11.4 min.

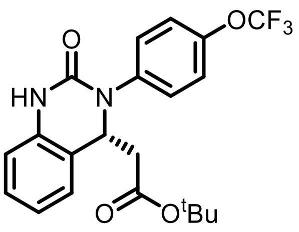

***tert*-Butyl (*R*)-2-(2-oxo-3-(4-(trifluoromethoxy)phenyl)-1,2,3,4-tetrahydroquinazolin-4-yl)acetate (2s)** was prepared following **General Procedure C**, using ***tert*-butyl (*E*)-3-(2-(3-(4-(trifluoromethoxy)phenyl)ureido)phenyl)acrylate (1s)** (84.5 mg, 0.2 mmol, 1.0 eq). Purification by silica gel chromatography (pentane/EtOAc = 3/1) afforded the title compound as a white solid (83.7 mg, 99%, 90:10 er).

^{1}H NMR (400 MHz, $CDCl_3$) δ 8.98 (s, 1H, NH), 7.53 – 7.45 (m, 2H, Ar-H), 7.32 – 7.25 (m, 2H, Ar-H), 7.24 – 7.14 (m, 2H, Ar-H), 7.07 – 6.93 (m, 1H, Ar-H), 6.87 – 6.73 (m, 1H, Ar-H), 5.24 (dd, *J* = 8.0, 4.8 Hz, 1H, CH), 2.76 (dd, *J* = 14.7, 4.8 Hz, 1H, CH_2), 2.69 (dd, *J* = 14.7, 8.0 Hz, 1H, CH_2), 1.32 (s, 9H, $OC(CH_3)_3$) ppm.

^{13}C NMR (101 MHz, $CDCl_3$) δ 169.3 ($C(O)OC(CH_3)_3$), 153.9 (C(O)NH), 147.5 (d, *J* = 2.1 Hz, ArC), 139.5 (ArC), 136.4 (ArC), 128.9 (s, 2 non-equivalent C, ArCH), 125.9 (ArCH), 122.4 (ArCH), 121.8 (ArCH), 121.1 (ArC), 120.6 (q, *J* = 257.5 Hz, CF_3), 114.6 (ArCH), 81.5 ($OC(CH_3)_3$), 60.2 (CH), 41.3 (CH_2), 27.9 ($OC(CH_3)_3$) ppm.

^{19}F NMR (377 MHz, $CDCl_3$) δ -57.87 (s) ppm.

HRMS (ESI) m/z calcd. for $C_{21}H_{22}F_3N_2O_4$ ($[M+H]^+$) 423.1526, found 423.1526

FT-IR (thin film) ν_{max} 3241, 2512, 2160, 2032, 1977, 1724, 1677, 1605, 1509, 1451, 1413, 1369, 1260, 1222, 1206, 1164, 1020, 845, 755 cm^{-1}.

m.p.: 124-126 °C

$[\alpha]_D^{25}$ = -37.4 (c=0.61, $CHCl_3$).

Chiral HPLC: Chiralcel AD-H, hexane/isopropanol = 70/30, 1.0 ml/min, λ = 230 nm, t_R (major) = 6.5 min, t_R (minor) = 8.7 min.

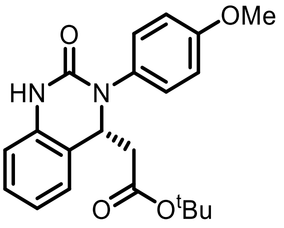

***tert*-Butyl (*R*)-2-(3-(4-methoxyphenyl)-2-oxo-1,2,3,4-tetrahydroquinazolin-4-yl)acetate (2t)** was prepared following **General Procedure C** (variation from standard conditions: reaction carried out at 50 ºC for 29 hours), using ***tert*-butyl (*E*)-3-(2-(3-(4-methoxyphenyl)ureido)phenyl)acrylate (1t)** (73.7 mg, 0.2 mmol, 1.0 eq). Purification by silica gel chromatography (pentane/EtOAc = 1/1) afforded the title compound as a white solid (73.4 mg, 99%, 92:8 er).

^{1}H NMR (400 MHz, $CDCl_3$) δ 8.37 (s, 1H, NH), 7.37 – 7.29 (m, 2H, Ar-H), 7.24 – 7.11 (m, 2H, Ar-H), 7.01 – 6.90 (m, 3H, Ar-H), 6.75 (dd, *J* = 7.9, 1.1 Hz, 1H, Ar-H), 5.14 (dd, *J* = 8.2, 4.6 Hz, 1H, CH), 3.83 (s, 3H, OCH_3), 2.76 (dd, *J* = 14.7, 4.6 Hz, 1H, CH_2), 2.69 (dd, *J* = 14.7, 8.2 Hz, 1H, CH_2), 1.32 (s, 9H, $OC(CH_3)_3$) ppm.

^{13}C NMR (101 MHz, $CDCl_3$) δ 169.5 ($C(O)OC(CH_3)_3$), 158.5 (C(O)NH or ArCO), 154.1 (C(O)NH or ArCO), 136.7 (ArC), 133.7 (ArC), 129.2 (ArCH), 128.8 (ArCH), 126.0 (ArCH), 122.1 (ArC), 121.2 (ArCH), 114.7 (ArCH), 114.4 (ArCH), 81.3 ($OC(CH_3)_3$), 60.4 (CH), 55.6 (OCH_3), 41.1(CH_2), 28.0 ($OC(CH_3)_3$) ppm.

HRMS (ESI) m/z calcd. for $C_{21}H_{25}N_2O_4$ ($[M+H]^+$) 369.1809, found 369.1810

FT-IR (thin film) ν_{max} 2857, 2160, 1675, 1605, 1581, 1495, 1450, 1380, 1305, 1249, 1188, 1117, 1035, 996, 975, 933, 889, 840, 761, 687, 666, 646 cm^{-1}.

m.p.: 66-89 ºC

$[\alpha]_D^{25}$ = -98.8 (c=0.63, $CHCl_3$).

Chiral HPLC: Chiralcel OD, hexane/isopropanol = 95/5, 1.0 ml/min, λ = 280 nm, t_R (major) = 33.3 min, t_R (minor) = 46.6 min.

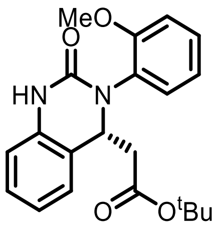

***tert*-Butyl (*R*)-2-(3-(2-methoxyphenyl)-2-oxo-1,2,3,4-tetrahydroquinazolin-4-yl)acetate (2u)** was prepared following **General Procedure C**, (variation from standard conditions: reaction carried out at 50 °C), using ***tert*-butyl (*E*)-3-(2-(3-(2-methoxyphenyl)ureido)phenyl)acrylate (1u)** (73.7 mg, 0.2 mmol, 1.0 eq). Purification by silica gel chromatography (pentane/EtOAc = 1/1) afforded the title compound as a white solid (8:1 (**A**:**B**) mixture of rotamers) (58.9 mg, 80%, 91:9 er).

^{1}H NMR (400 MHz, CDCl$_3$) δ 8.57 (s, 0.11H, NH (**B**)), 8.46 (s, 0.88H, NH (**A**)), 7.41 (brs, 1H, Ar-H), 7.32 (ddd, *J* = 8.2, 7.5, 1.7 Hz, 1H, Ar-H), 7.20 – 7.10 (m, 2H, Ar-H), 7.06 – 6.96 (m, 2H, Ar-H), 6.96 – 6.87 (m, 1H, Ar-H), 6.78 (dd, *J* = 8.0, 1.1 Hz, 0.11H, Ar-H (**B**)), 6.73 (dd, *J* = 8.3, 1.2 Hz, 0.88H, Ar-H (**A**)), 5.10 (dd, *J* = 8.3, 4.7 Hz, 1H, CH), 3.80 (d, *J* = 3.4 Hz, 3H, OCH$_3$), 2.86 – 2.60 (m, 2H, CH$_2$), 1.31 (s, 8H, OC(CH$_3$)$_3$ (**A**)), 1.29 (s, 1H, OC(CH$_3$)$_3$ (**B**)) ppm.

^{13}C NMR (101 MHz, CDCl$_3$) δ 169.8 (C(O)OC(CH$_3$)$_3$), 155.3 (C(O)NH or ArCO), 153.7 (C(O)NH or ArCO), 136.8 (ArC), 131.2 (ArC), 131.1 (ArCH), 129.0 (ArCH), 128.5 (ArCH), 126.0 (ArCH), 121.9 (ArCH), 121.5 (ArCH), 120.9 (ArC), 114.3 (ArCH), 112.4 (ArCH), 81.1 (OC(CH$_3$)$_3$), 58.0 (CHCH$_2$), 55.8 (OCH$_3$), 41.6 (CHCH$_2$), 28.0 (OC(CH$_3$)$_3$) ppm.

HRMS (ESI) m/z calcd. for $C_{21}H_{25}N_2O_4$ ([M+H]$^+$) 369.1809, found 369.1809

FT-IR (thin film) ν_{max} 3791, 3213, 3068, 2932, 2161, 2035, 1978, 1723, 1676, 1601, 1502, 1454, 1423, 1367, 1288, 1259, 1143, 1116, 1027, 847, 752, 692 cm^{-1}.

m.p.: 70-73 °C

$[\alpha]_D^{25}$ = -147.4 (c=0.63, CHCl$_3$).

Chiral HPLC: Chiralcel IA, hexane/isopropanol = 85/15, 1.0 ml/min, λ = 240 nm, t_R (major) = 15.1 min, t_R (minor) = 13.3 min.

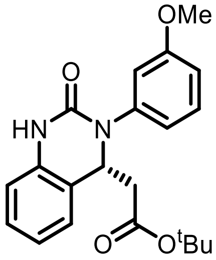

***tert*-Butyl (*R*)-2-(3-(3-methoxyphenyl)-2-oxo-1,2,3,4-tetrahydroquinazolin-4-yl)acetate (2v)** was prepared following **General Procedure C**, (variation from standard conditions: reaction carried out at 50 °C), using ***tert*-butyl (*E*)-3-(2-(3-(3-methoxyphenyl)ureido)phenyl)acrylate (1v)** (73.7 mg, 0.2 mmol, 1.0 eq). Purification by silica gel chromatography (pentane/EtOAc = 3/2) afforded the title compound as a white solid (71.4 mg, 97%, 93:7 er).

^{1}H NMR (400 MHz, $CDCl_3$) δ 8.91 (s, 1H, NH), 7.34 (ddd, *J* = 8.3, 7.4, 0.9 Hz, 1H, Ar-H), 7.17 (dd, *J* = 8.0, 6.6 Hz, 2H, Ar-H), 7.07 – 6.99 (m, 2H, Ar-H), 6.94 (td, *J* = 7.4, 1.2 Hz, 1H, Ar-H), 6.86 (ddd, *J* = 8.3, 2.3, 1.2 Hz, 1H, Ar-H), 6.82 – 6.75 (m, 1H, Ar-H), 5.23 (dd, *J* = 8.4, 4.5 Hz, 1H, CH), 3.82 (s, 3H, OCH_3), 2.84 – 2.65 (m, 2H, CH_2), 1.33 (s, 9H, $OC(CH_3)_3$) ppm.

^{13}C NMR (101 MHz, $CDCl_3$) δ 169.4 ($C(O)OC(CH_3)_3$), 160.3 (ArCO), 153.9 (C(O)NH), 142.1 (ArC), 136.6 (ArC), 129.9 (ArCH), 128.7 (ArCH), 125.9 (ArCH), 122.0 (ArCH), 121.3 (ArC), 119.7 (ArCH), 114.5 (ArCH), 113.5 (ArCH), 112.8 (ArCH), 81.3 ($OC(CH_3)_3$), 60.1 ($CHCH_2$), 55.5 (OCH_3), 41.2 ($CHCH_2$), 28.0 ($OC(CH_3)_3$) ppm.

HRMS (ESI) m/z calcd. for $C_{21}H_{25}N_2O_4$ ($[M+H]^+$) 369.1809, found 369.1810

FT-IR (thin film) ν_{max} 3221, 3068, 2978, 2161, 2027, 1977, 1722, 1674, 1604, 1489, 1453, 1416, 1368, 1289, 1207, 1142, 1045, 844, 755, 697 cm^{-1}.

m.p.: 54-57 °C

$[\alpha]_D^{25}$ = -92.1 (c=0.92, $CHCl_3$).

Chiral HPLC: Chiralcel IA, hexane/isopropanol = 85/15, 1.0 ml/min, λ = 240 nm, t_R (major) = 20.5 min, t_R (minor) = 12.1 min.

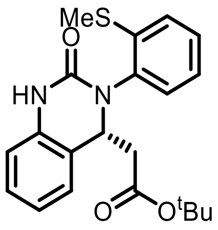

***tert*-Butyl (*R*)-2-(3-(2-(methylthio)phenyl)-2-oxo-1,2,3,4-tetrahydroquinazolin-4-yl)acetate (2w)** was prepared following **General Procedure C**, (variation from standard conditions: reaction carried out at 50 °C), using ***tert*-butyl (*E*)-3-(2-(3-(2-(methylthio)phenyl)ureido)phenyl)acrylate (1w)** (76.9 mg, 0.2 mmol, 1.0 eq). Purification by silica gel chromatography (pentane/EtOAc = 1/1) afforded the title compound as a white solid (74.8 mg, 98%, 96:4 er).

^{1}H NMR (400 MHz, $CDCl_3$) δ 8.75 (s, 1H, NH), 7.45 (dd, *J* = 7.6, 1.5 Hz, 1H, Ar-H), 7.39 – 7.30 (m, 1H, Ar-H), 7.28 – 7.20 (m, 2H, Ar-H), 7.18 – 7.09 (m, 2H, Ar-H), 6.93 (td, *J* = 7.6, 1.2 Hz, 1H, Ar-H), 6.74 (dd, *J* = 7.9, 1.2 Hz, 1H, Ar-H), 5.11 (dd, *J* = 7.3, 5.5 Hz, 1H, CH), 2.83 – 2.67 (m, 2H, CH_2), 2.33 (s, 3H, SCH_3), 1.31 (s, 9H, $OC(CH_3)_3$) ppm.

^{13}C NMR (101 MHz, $CDCl_3$) δ 169.5 ($C(O)OC(CH_3)_3$), 153.8 (C(O)NH), 137.6 (ArC), 137.0 (ArC), 136.5 (ArC), 131.6 (ArCH), 128.6 (ArCH), 128.5 (ArCH), 126.1 (ArCH), 125.8 (ArCH), 125.0 (ArCH), 122.0 (ArCH), 121.2 (ArC), 114.6 (ArCH), 81.1 ($OC(CH_3)_3$), 58.0 (CH), 41.2 (CH_2), 27.9 ($OC(CH_3)_3$), 14.5 (SCH_3) ppm.

HRMS (ESI) m/z calcd. for $C_{21}H_{25}{}^{32}SN_2O_3$ ($[M+H]^+$) 385.1580, found 385.1581

FT-IR (thin film) ν_{max} 3019, 2161, 2026, 1722, 1675, 1603, 1473, 1447, 1369, 1295, 1215, 1143, 929, 745, 668, 628 cm^{-1}.

m.p.: 85-87 °C

$[\alpha]_D^{25}$ = -261.9 (c=0.70, $CHCl_3$).

Chiral HPLC: Chiralcel OD, hexane/isopropanol = 90/10, 1.0 ml/min, λ = 240 nm, t_R (major) = 15.2 min, t_R (minor) = 20.8 min.

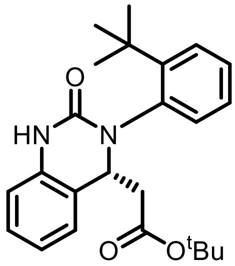

***tert*-Butyl (*R*)-2-(3-(2-(*tert*-butyl)phenyl)-2-oxo-1,2,3,4-tetrahydroquinazolin-4-yl)acetate (2x)** was prepared following **General Procedure C**, (variation from standard conditions: reaction carried out at 50 °C), using ***tert*-butyl (*E*)-3-(2-(3-(2-(tert-butyl)phenyl)ureido)phenyl)acrylate (1x)** (78.9 mg, 0.2 mmol, 1.0 eq). Purification by silica gel chromatography (pentane/EtOAc = 7/3) afforded the title compound as a white solid (71.1 mg, 90%, 97:3 er).

^{1}H NMR (400 MHz, CDCl$_3$) δ 8.97 (s, 1H, N$\underline{H}$), 7.60 – 7.53 (m, 1H, Ar-$\underline{H}$), 7.40 – 7.28 (m, 3H, Ar-$\underline{H}$), 7.14 (td, J = 7.6, 1.1 Hz, 1H, Ar-$\underline{H}$), 7.09 (dd, J = 7.6, 1.4 Hz, 1H, Ar-$\underline{H}$), 6.91 (td, J = 7.6, 1.1 Hz, 1H, Ar-$\underline{H}$), 6.72 (dd, J = 8.1, 1.1 Hz, 1H, Ar-$\underline{H}$), 4.89 (dd, J = 9.5, 4.3 Hz, 1H, C$\underline{H}$), 2.99 (dd, J = 14.2, 4.3 Hz, 1H, C$\underline{H}_2$), 2.80 (dd, J = 14.2, 9.5 Hz, 1H, C$\underline{H}_2$), 1.305 (s, 9H, C(C$\underline{H}_3$)$_3$), 1.296 (s, 9H, C(C$\underline{H}_3$)$_3$) ppm.

^{13}C NMR (101 MHz, CDCl$_3$) δ 169.6 ($\underline{C}$(O)OC(CH$_3$)$_3$), 155.1 ($\underline{C}$(O)NH), 147.5 (Ar$\underline{C}$), 138.8 (Ar$\underline{C}$), 136.6 (Ar$\underline{C}$), 133.7 (Ar$\underline{C}$H), 128.9 (Ar$\underline{C}$H), 128.7 (Ar$\underline{C}$H), 128.5 (Ar$\underline{C}$H), 127.0 (Ar$\underline{C}$H), 126.3 (Ar$\underline{C}$H), 122.1 (Ar$\underline{C}$H), 121.2 (Ar$\underline{C}$), 114.6 (Ar$\underline{C}$H), 81.2 (O$\underline{C}$(CH$_3$)$_3$), 60.6 ($\underline{C}$HCH$_2$), 40.1 (CH$\underline{C}$H$_2$), 35.7 (Ar$\underline{C}$(CH$_3$)$_3$), 31.6 (C($\underline{C}$H$_3$)$_3$), 28.0 (C($\underline{C}$H$_3$)$_3$) ppm.

HRMS (ESI) m/z calcd. for $C_{24}H_{31}N_2O_3$ ([M+H]$^+$) 395.2329, found 395.2329

FT-IR (thin film) ν_{max} 3214, 3065, 2972, 2513, 2160, 2028, 1978, 1724, 1677, 1602, 1503, 1486, 1447, 1418, 1367, 1289, 1140, 1092, 1054, 960, 847, 755, 725, 667, 630 cm^{-1}.

m.p.: 178-180 °C

$[\alpha]_D^{25}$ = -147.7 (c=0.71, CHCl$_3$).

Chiral HPLC: Chiralcel IA, hexane/isopropanol = 95/5, 1.0 ml/min, λ = 240 nm, t_R (major) = 17.3 min, t_R (minor) = 18.9 min.

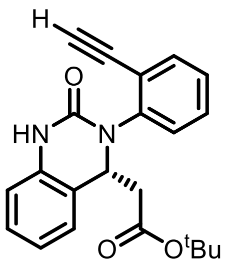

***tert*-Butyl (*R*)-2-(3-(2-ethynylphenyl)-2-oxo-1,2,3,4-tetrahydroquinazolin-4-yl)acetate (2y)** was prepared following **General Procedure C**, (variation from standard conditions: reaction carried out for 48h), using ***tert*-butyl (*E*)-3-(2-(3-(2-ethynylphenyl)ureido)phenyl)acrylate (1y)** (72.5 mg, 0.2 mmol, 1.0 eq). Purification by silica gel chromatography (pentane/EtOAc = 3/2) afforded the title compound as a white solid (65.8 mg, 91%, 96:4 er).

^{1}H NMR (400 MHz, $CDCl_3$) δ 8.57 (s, 1H, NH), 7.60 (dd, *J* = 7.7, 1.6 Hz, 1H, Ar-H), 7.52 (d, *J* = 8.0 Hz, 1H, Ar-H), 7.44 (td, *J* = 7.7, 1.6 Hz, 1H, Ar-H), 7.31 (td, *J* = 7.7, 1.3 Hz, 1H, Ar-H), 7.14 (dtd, *J* = 8.0, 3.9, 1.3 Hz, 2H, Ar-H), 6.92 (td, *J* = 7.7, 1.1 Hz, 1H, Ar-H), 6.74 (dd, *J* = 8.3, 1.1 Hz, 1H, Ar-H), 5.33 (dd, *J* = 8.2, 4.9 Hz, 1H, CH), 3.10 (s, 1H), 2.83-2.74 (m, 1H, CH_2), 2.69 (dd, *J* = 14.8, 8.3 Hz, 1H, CH_2), 1.31 (s, 9H, $OC(CH_3)_3$) ppm.

^{13}C NMR (101 MHz, $CDCl_3$) δ 169.5 ($C(O)OC(CH_3)_3$), 153.6 (C(O)NH), 142.8 (ArC), 136.6 (ArC), 134.0 (ArCH), 130.8 (ArCH), 129.6 (ArCH), 128.6 (ArCH), 127.5 (ArCH), 125.8 (ArCH), 122.1 (ArCH), 121.6 (ArC), 121.4 (ArC), 114.5 (ArCH), 82.9 (C≡CH), 81.2 ($OC(CH_3)_3$), 80.3 (C≡CH), 59.6 ($CHCH_2$), 41.4 ($CHCH_2$), 28.0 ($OC(CH_3)_3$) ppm.

HRMS (ESI) m/z calcd. for $C_{22}H_{23}N_2O_3$ ($[M+H]^+$) 363.1703, found 363.1703

FT-IR (thin film) ν_{max} 3019, 2160, 2031, 1721, 1675, 1604, 1485, 1451, 1422, 1369, 1287, 1215, 1143, 929, 745, 668, 628 cm^{-1}.

m.p.: 76-78 °C

$[\alpha]_D^{25}$ = -306.6 (c=0.68, $CHCl_3$).

Chiral HPLC: Chiralcel OD, hexane/isopropanol = 95/5, 1.0 ml/min, λ = 254 nm, t_R (major) = 24.2 min, t_R (minor) = 36.5 min.

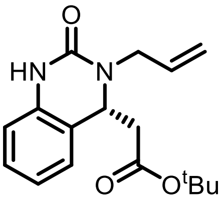

***tert*-Butyl (*R*)-2-(3-allyl-2-oxo-1,2,3,4-tetrahydroquinazolin-4-yl)acetate (2z)** was prepared following **General Procedure C** (variation from standard conditions: reaction carried out at 60 °C), using ***tert*-butyl (*E*)-3-(2-(3-allylureido)phenyl)acrylate (1z)** (60.5 mg, 0.2 mmol, 1.0 eq). Purification by silica gel chromatography (pentane/Et_2O = 7/13) afforded the title compound as a colorless oil (60.4 mg, 99%, 85:15 er).

^{1}H NMR (400 MHz, $CDCl_3$) δ 8.88 (s, 1H, NH), 7.17 (tt, *J* = 7.7, 2.1 Hz, 1H, Ar-H), 7.08 (dd, *J* = 7.7, 1.5 Hz, 1H, Ar-H), 6.99 – 6.86 (m, 1H, Ar-H), 6.86 – 6.78 (m, 1H, Ar-H), 5.83 (dddd, *J* = 17.0, 10.1, 6.7, 4.8 Hz, 1H, CH=CH_2), 5.24 (dd, *J* = 17.3, 1.6 Hz, 1H, CH=CH_2), 5.19 (dd, *J* = 10.3, 1.5 Hz, 1H, CH=CH_2), 4.83 (dd, *J* = 8.4, 4.8 Hz, 1H, CH), 4.66 (ddt, *J* = 15.8, 5.1, 1.7 Hz, 1H, NCH_2), 3.68 (dd, *J* = 15.7, 6.7 Hz, 1H, NCH_2), 2.69 (dd, *J* = 14.8, 4.8 Hz, 1H, CH_2), 2.55 (dd, *J* = 14.8, 8.4 Hz, 1H, CH_2), 1.38 (s, 9H, $OC(CH_3)_3$) ppm.

^{13}C NMR (101 MHz, $CDCl_3$) δ 169.9 ($C(O)OC(CH_3)_3$), 155.0 (C(O)NH), 136.7 (ArC), 133.4 (CH=CH_2), 128.6 (ArCH), 125.7 (ArCH), 122.0 (ArCH), 121.3 (ArC), 117.7 (CH=CH_2), 114.3 (ArCH), 81.3 ($OC(CH_3)_3$), 55.2 (CH), 47.8 (NCH_2), 41.1 (CH_2), 28.1 ($OC(CH_3)_3$) ppm.

HRMS (ESI) m/z calcd. for $C_{17}H_{23}N_2O_3$ ($[M+H]^+$) 303.1703, found 303.1704

FT-IR (thin film) ν_{max} 3211, 3067, 2977, 2930, 2535, 2160, 2028, 1722, 1669, 1605, 1466, 1416, 1368, 1289, 1259, 1147, 995, 925, 846, 755 cm^{-1}.

$[\alpha]_D^{25}$ = +38.1 (c=1.83, $CHCl_3$).

Chiral HPLC: Chiralcel IA, hexane/isopropanol = 95/5, 1.0 ml/min, λ = 254 nm, t_R (major) = 20.1 min, t_R (minor) = 22.2 min.

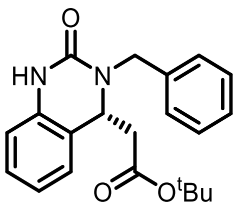

***tert*-Butyl (*R*)-2-(3-benzyl-2-oxo-1,2,3,4-tetrahydroquinazolin-4-yl)acetate (2aa)** was prepared following **General Procedure C** (variation from standard conditions: reaction carried out at 50 ºC for 30 hours), using ***tert*-butyl (*E*)-3-(2-(3-benzylureido)phenyl)acrylate (1aa)** (70.5 mg, 0.2 mmol, 1.0 eq). Purification by silica gel chromatography (pentane/EtOAc = 7/3) afforded the title compound as a white solid (5:1 (**A**:**B**) mixture of rotamers) (67.7 mg, 96%, 85:15 er).

^{1}H NMR (400 MHz, $CDCl_3$) δ 8.92 (s, 0.8H, NH (**A**)), 8.77 (s, 0.2H, NH (**B**)), 7.35 – 7.24 (m, 5H, Ar-H), 7.17 (td, J = 7.7, 1.5 Hz, 0.2H (**B**)), 7.14 (td, J = 7.7, 1.5 Hz, 0.8H (**A**)), 6.99 (dd, *J* = 7.6, 1.5 Hz, 1H, Ar-H), 6.89 (td, J = 7.6, 1.1 Hz, 0.2H (**B**)), 6.87 (td, J = 7.6, 1.1 Hz, 0.8H (**A**)), 6.83 (dd, *J* = 8.0, 1.1 Hz, 0.2H (**B**)), 6.77 (dd, *J* = 8.0, 1.1 Hz, 0.8H, Ar-H (**A**)), 5.31 (d, *J* = 15.4 Hz, 1H, NCH_2), 4.79 (dd, *J* = 8.2, 4.9 Hz, 1H, CH), 4.23 (dd, *J* = 15.4, 1.5 Hz, 1H, NCH_2), 2.72 – 2.51 (m, 2H, CH_2), 1.38 (s, 7.5H, $OC(CH_3)_3$ (**A**)), 1.37 (s, 1.5H, $OC(CH_3)_3$ (**B**)) ppm.

^{13}C NMR (101 MHz, $CDCl_3$) δ 169.8 ($C(O)OC(CH_3)_3$), 155.6 (C(O)NH), 137.1 (ArC), 129.0 (ArC), 128.8 (ArCH), 128.7 (ArC), 128.5 (ArCH), 128.0 (ArCH), 127.7 (ArCH), 125.8 (ArCH), 122.3 (ArCH), 114.4 (ArCH), 81.4 ($OC(CH_3)_3$), 55.4 (CH), 48.7 (NCH_2), 44.2, 41.2 (CH_2), 28.1 ($OC(CH_3)_3$) ppm.

HRMS (ESI) m/z calcd. for $C_{21}H_{24}N_2O_3{}^{23}Na$ ($[M+Na]^+$) 375.1679, found 375.1680

FT-IR (thin film) ν_{max} 3204, 3064, 2977, 2929, 2534, 2160, 2032, 1977, 1715, 1666, 1605, 1493, 1467, 1454, 1367, 1294, 1259, 1151, 1074, 1030, 958, 845, 756, 714 cm^{-1}.

m.p.: 43-46 ºC

$[\alpha]_D^{25}$ = +43.6 (c=0.93, $CHCl_3$).

Chiral HPLC: Chiralcel OD, hexane/isopropanol = 95/5, 1.0 ml/min, λ = 240 nm, t_R (major) = 20.7 min, t_R (minor) = 13.1 min.

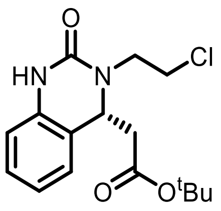

***tert*-Butyl *(R)*-2-(3-(2-chloroethyl)-2-oxo-1,2,3,4-tetrahydroquinazolin-4-yl)acetate (2ab)** was prepared following **General Procedure C** (variation from standard conditions: reaction carried out for 120h), using ***tert*-butyl *(E)*-3-(2-(3-(2-chloroethyl)ureido)phenyl)acrylate (1ab)** (65.0 mg, 0.2 mmol, 1.0 eq). Purification by silica gel chromatography (pentane/EtOAc = 13/7) afforded the title compound as a colorless oil (62.0 mg, 96%, 81:19 er).

^{1}H NMR (400 MHz, $CDCl_3$) δ 8.92 (s, 1H, N<u>H</u>), 7.19 (tt, *J* = 7.6, 1.6 Hz, 1H, Ar-<u>H</u>), 7.10 (dd, *J* = 7.6, 1.3 Hz, 1H, Ar-<u>H</u>), 6.93 (td, *J* = 7.6, 1.3 Hz, 1H, Ar-<u>H</u>), 6.86 – 6.78 (m, 1H, Ar-<u>H</u>), 4.93 (dd, *J* = 7.7, 5.5 Hz, 1H, C<u>H</u>), 4.27 (ddd, *J* = 14.2, 6.3, 5.1 Hz, 1H, $C\underline{H}_2CH_2$), 3.78 – 3.62 (m, 2H, $CH_2C\underline{H}_2$), 3.41 – 3.30 (m, 1H, $C\underline{H}_2CH_2$), 2.74 – 2.63 (m, 1H, $CHC\underline{H}_2$), 2.55 (dd, *J* = 15.0, 7.7 Hz, 1H, $CHC\underline{H}_2$), 1.39 (s, 9H, $OC(C\underline{H}_3)_3$) ppm.

^{13}C NMR (101 MHz, $CDCl_3$) δ 169.7 ($\underline{C}(O)OC(CH_3)_3$), 155.0 (<u>C</u>(O)NH), 136.5 (Ar<u>C</u>), 128.7 (Ar<u>C</u>H), 125.8 (Ar<u>C</u>H), 122.3 (Ar<u>C</u>H), 121.3 (Ar<u>C</u>), 114.3 (Ar<u>C</u>H), 81.5 ($O\underline{C}(CH_3)_3$), 58.0 (<u>C</u>H), 48.5 ($\underline{C}H_2CH_2$), 42.0 ($CH_2\underline{C}H_2$), 41.9 ($CH\underline{C}H_2$), 28.1 ($OC(\underline{C}H_3)_3$) ppm.

HRMS (ESI) m/z calcd. for $C_{16}H_{21}ClN_2O_3{}^{23}Na$ ($[M+Na]^+$) 347.1133, found 347.1134

FT-IR (thin film) ν_{max} 3019, 2160, 2031, 1715, 1667, 1605, 1466, 1369, 1294, 1259, 1215, 1150, 1012, 929, 844, 745, 668, 626 cm^{-1}.

$[\alpha]_D^{25}$ = +44.6 (c=1.89, $CHCl_3$).

Chiral HPLC: Chiralcel OD, hexane/isopropanol = 90/10, 1.0 ml/min, λ = 280 nm, t_R (major) = 16.0 min, t_R (minor) = 11.0 min.

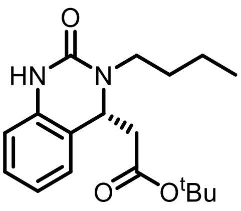

***tert*-Butyl *(R)*-2-(3-butyl-2-oxo-1,2,3,4-tetrahydroquinazolin-4-yl)acetate (2ac)** was prepared following **General Procedure C** (variation from standard conditions: reaction carried out at 80 °C for 72 hours), using ***tert*-butyl (*E*)-3-(2-(3-butylureido)phenyl)acrylate (1ac)** (63.7 mg, 0.2 mmol, 1.0 eq). Purification by silica gel chromatography (pentane/EtOAc = 3/1) afforded the title compound as a colorless oil (45.9 mg, 72%, 86:14 er).

^{1}H NMR (400 MHz, $CDCl_3$) δ 8.44 (s, 1H, NH), 7.17 (tt, *J* = 7.9, 1.4 Hz, 1H, Ar-H), 7.10 (dd, *J* = 7.6, 1.5 Hz, 1H, Ar-H), 6.90 (td, *J* = 7.5, 1.1 Hz, 1H, Ar-H), 6.79 (ddd, *J* = 7.9, 4.1, 1.1 Hz, 1H, Ar-H), 4.80 (dd, *J* = 8.5, 4.8 Hz, 1H, CH), 4.05 – 3.92 (m, 1H, CH_2), 2.97 (ddd, *J* = 14.0, 7.9, 6.2 Hz, 1H, CH_2), 2.71 – 2.61 (m, 1H, CH_2C(O)O), 2.59 – 2.48 (m, 1H, CH_2C(O)O), 1.66 – 1.52 (m, 2H, CH_2), 1.38 (s, 9H, OC(CH_3)$_3$), 1.36 – 1.29 (m, 2H, CH_2), 0.92 (t, *J* = 7.4 Hz, 3H, CH_3) ppm.

^{13}C NMR (101 MHz, $CDCl_3$) 170.0 (C(O)OC(CH_3)$_3$), 162.6, 152.3 (C(O)NH), 138.8 (ArC), 135.0 (ArCH), 128.5 (ArCH), 123.4 (ArCH), 122.3 (ArC), 115.1 (ArCH), 81.5 (OC(CH_3)$_3$), 56.2 (CH), 41.0 (CH_2C(O)O), 30.2 (CH_2), 28.2 (CH_2), 28.1 (OC(CH_3)$_3$), 20.4 (CH_2), 14.0 (CH_3) ppm.

HRMS (ESI) m/z calcd. for $C_{18}H_{26}N_2O_3{}^{23}Na$ ([M+Na]$^+$) 341.1836, found 341.1836

FT-IR (thin film) ν_{max} 3207, 2960, 2932, 2873, 2160, 2028, 1714, 1663, 1607, 1493, 1454, 1409, 1370, 1287, 1149, 1077, 949, 845, 757, 693 cm^{-1}.

$[\alpha]_D^{25}$ = +22.8 (c=1.41, $CHCl_3$).

Chiral HPLC: Chiralcel OD, hexane/isopropanol = 95/5, 1.0 ml/min, λ = 240 nm, t_R (major) = 11.6 min, t_R (minor) = 8.3 min.

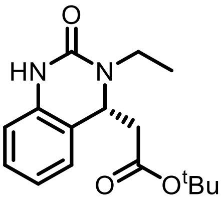

***tert*-Butyl (*R*)-2-(3-ethyl-2-oxo-1,2,3,4-tetrahydroquinazolin-4-yl)acetate (2ad)** was prepared following **General Procedure C** (variation from standard conditions: reaction carried out for 216 hours), using ***tert*-butyl (*E*)-3-(2-(3-ethylureido)phenyl)acrylate (1ad)** (58.1 mg, 0.2 mmol, 1.0 eq). Purification by silica gel chromatography (pentane/Et_2O = 7/13) afforded the title compound as an off-white solid (33.1 mg, 57%, 89:11 er).

^{1}H NMR (400 MHz, $CDCl_3$) δ 8.34 (s, 1H, NH), 7.17 (td, *J* = 7.6, 1.5 Hz, 1H, Ar-H), 7.11 (dd, *J* = 7.6, 1.5 Hz, 1H, Ar-H), 6.91 (td, *J* = 7.6, 1.1 Hz, 1H, Ar-H), 6.82 – 6.75 (m, 1H, Ar-H), 4.84 (dd, *J* = 8.4, 4.9 Hz, 1H, CH), 3.99 (dq, *J* = 14.2, 7.1 Hz, 1H, NCH_2), 3.10 (dq, *J* = 14.2, 7.1 Hz, 1H, NCH_2), 2.72 – 2.62 (m, 1H, CH_2), 2.55 (dd, *J* = 14.9, 8.4 Hz, 1H, CH_2), 1.39 (s, 9H, $OC(CH_3)_3$), 1.22 (t, *J* = 7.1 Hz, 3H, CH_3) ppm.

^{13}C NMR (101 MHz, $CDCl_3$) δ 170.0 ($C(O)OC(CH_3)_3$), 154.8 (C(O)NH), 136.8 (ArC), 128.6 (ArCH), 125.8 (ArCH), 121.9 (ArCH), 121.6 (ArC), 114.1 (ArCH), 81.4 ($OC(CH_3)_3$), 55.7 (CH), 41.6 (CH_2), 40.7 (NCH_2), 28.1 ($OC(CH_3)_3$), 13.8 (CH_3) ppm.

HRMS (ESI) m/z calcd. for $C_{16}H_{22}N_2O_3{}^{23}Na$ ($[M+Na]^+$) 313.1523, found 313.1523

FT-IR (thin film) ν_{max} 3020, 2402, 2161, 2028, 1666, 1605, 1471, 1215, 1148, 929, 745, 668, 627 cm^{-1}.

m.p.: 51-53 °C

$[\alpha]_D^{25}$ = +23.1 (c=0.47, $CHCl_3$).

Chiral HPLC: Chiralcel OD, hexane/isopropanol = 90/10, 1.0 ml/min, λ = 254 nm, t_R (major) = 10.2 min, t_R (minor) = 6.6 min.

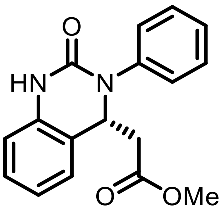

Methyl (*R*)-2-(2-oxo-3-phenyl-1,2,3,4-tetrahydroquinazolin-4-yl)acetate (2ae) was prepared following **General Procedure C** (variation from standard conditions: reaction carried out for 48 hours), using **methyl (*E*)-3-(2-(3-phenylureido)phenyl)acrylate (1ae)** (59.3 mg, 0.2 mmol, 1.0 eq). Purification by silica gel chromatography (pentane/EtOAc = 7/3) afforded the title compound as an off-white solid (10:1 (**A**:**B**) mixture of rotamers) (58.5 mg, 99%, 92:8 er). Data is consistent with the published literature. [11]

^{1}H NMR (400 MHz, CDCl$_3$) δ 8.99 (s, 0.1H, NH (**B**)), 8.94 (s, 1H, NH (**A**)), 7.49 – 7.40 (m, 4H, Ar-H), 7.36 – 7.27 (m, 1H, Ar-H), 7.23 – 7.11 (m, 2H, Ar-H), 6.95 (td, *J* = 7.5, 1.2 Hz, 1H, Ar-H), 6.84 (d, *J* = 8.0, 0.1H, Ar-H (**B**)), 6.78 (d, *J* = 8.0, 1H, Ar-H (**A**)), 5.29 (dd, *J* = 8.4, 4.9 Hz, 1H, CH), 3.52 (s, 3H, CH_3 (**A**)), 3.50 (s, 0.3H, CH_3 (**B**)) 2.90 (dd, *J* = 15.0, 4.9 Hz, 1H, CH_2), 2.79 (dd, *J* = 15.0, 8.4 Hz, 1H, CH_2) ppm.

^{13}C NMR (101 MHz, CDCl$_3$) δ 170.5 (C(O)OCH$_3$), 154.0 (C(O)NH), 140.9 (ArC), 136.6 (ArC), 129.3 (ArCH), 128.9 (ArCH), 127.6 (ArCH), 127.0 (ArCH), 125.6 (ArCH), 122.2 (ArCH), 121.2 (ArC), 114.6 (ArCH), 60.0 (CH), 51.8 (CH_3), 40.0 (CH_2) ppm.

HRMS (ESI) m/z calcd. for $C_{17}H_{17}N_2O_3$ ([M+H]$^+$) 297.1234, found 297.1235

FT-IR (thin film) ν_{max} 3280, 2950, 2555, 2160, 2024, 1977, 1718, 1642, 1599, 1582, 1552, 1498, 1445, 1322, 1275, 1235, 1195, 1171, 976, 760, 749, 693 cm^{-1}.

m.p.: 52-54 °C

$[\alpha]_D^{25}$ = -113.6 (c=0.28, CHCl$_3$).

Chiral HPLC: Chiralcel AD-H, hexane/isopropanol = 70/30, 1.0 ml/min, λ = 240 nm, t_R (major) = 12.5 min, t_R (minor) = 15.8 min.

7. Derivatization and Scale-up

7.1. Scale-up

CPME (0.0125 M, 32 mL) was added to the BIMP catalysts precursors **S9** (189.8 mg, 0.4 mmol, 0.02 eq) and tris(4-methoxyphenyl)phosphine (141.0 mg, 0.4 mmol, 0.02 eq) in a 100 mL RBF under N_2 at room temperature. The reaction mixture was stirred for 24 hours before transferring to a 1000 mL RBF and evaporating to dryness under reduced pressure. The iminophosphorane product was confirmed by HRMS and TLC and used as crude for enantioselective reactions without any purification.

Urea **1j** (9.29 g, 20 mmol, 1.0 eq) was added to a solution of the *in situ* generated catalyst (0.4 mmol, 0.02 eq) in toluene (0.025M, 800 mL) under N_2 at 50 °C. The reaction mixture was stirred at room temperature for 20 hours before evaporating to dryness under reduced pressure. Purification by silica gel chromatography (pentane/EtOAc = 7/3) afforded **2j** as an off-white solid (7.90 g, 85%, 92.5:7.5 er).

Material was dissolved in the minium volume of CH_2Cl_2 before carefully layering with pentane in a RBF. The flask was sealed with a suba-seal. A needle was placed in the seal to allow slow evaporation. The crushed out solid after several days was filtered out. The combined filtrate was concentrated under reduced pressure to afford colourless crystal of enantioenriched **2j** (99.5:0.5 er).

7.2. Derivatization

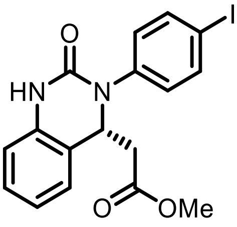

Methyl (*R*)-2-(3-(4-iodophenyl)-2-oxo-1,2,3,4-tetrahydroquinazolin-4-yl)acetate (3) was prepared according to the following procedure. Trifluoroacetic acid (3 mL, 7 mL/mmol) was added dropwise to a solution of the *tert*-butyl (*R*)-2-(3-(4-iodophenyl)-2-oxo-1,2,3,4-tetrahydroquinazolin-4-yl)acetate (**2j**) (200 mg, 0.43 mmol, 1.0 eq, 99.5:0.5 er) in CH_2Cl_2 (6 mL, 2 x Volume of trifluoroacetic acid) under N_2 at 0 °C. The reaction mixture was warmed to room temperature and stirred for 5 hours. Volatiles were removed under a stream of nitrogen gas to afford (*R*)-2-(3-(4-iodophenyl)-2-oxo-1,2,3,4-tetrahydroquinazolin-4-yl)acetic acid which was used as crude for next step without any purification.

Thionyl chloride (112.6 mg, 0.95 mmol, 2.2 eq) was added dropwise to a solution of the (*R*)-2-(3-(4-iodophenyl)-2-oxo-1,2,3,4-tetrahydroquinazolin-4-yl)acetic acid in MeOH (11 mL, 0.04M) under N_2 at 0 °C. The reaction mixture was warmed to room temperature and stirred for 21 hours before quenching with a saturated aqueous solution of $NaHCO_3$ (50 mL). The aqueous layer was extracted with CH_2Cl_2 (3 x 50 mL). The combined organic layers were washed with brine (100 mL), dried over Na_2SO_4, filtered and evaporated to dryness under reduced pressure to afford the title compound as an off-white solid (176.7 mg, 97%, 99.4:0.6 er).

^{1}H NMR (400 MHz, $CDCl_3$) δ 8.38 (s, 1H, NH), 7.78 – 7.70 (m, 2H, Ar-H), 7.24 – 7.13 (m, 4H, Ar-H), 6.98 (td, *J* = 7.5, 1.1 Hz, 1H, Ar-H), 6.78 (dd, *J* = 7.9, 1.1 Hz, 1H, Ar-H), 5.26 (dd, *J* = 8.2, 5.0 Hz, 1H, CH), 3.53 (s, 3H, CH_3), 2.85 (dd, *J* = 15.1, 5.0 Hz, 1H, CH_2), 2.76 (dd, *J* = 15.1, 8.2 Hz, 1H, CH_2) ppm.

^{13}C NMR (101 MHz, $CDCl_3$) δ 170.4 (<u>C</u>(O)OCH_3), 153.5 (<u>C</u>(O)NH), 140.6 (Ar<u>C</u>), 138.5 (Ar<u>C</u>H), 136.3 (Ar<u>C</u>), 129.4 (Ar<u>C</u>H), 129.2 (Ar<u>C</u>H), 125.8 (Ar<u>C</u>H), 122.7 (Ar<u>C</u>H), 121.1 (Ar<u>C</u>), 114.6 (Ar<u>C</u>H), 91.8(<u>C</u>I), 59.9 (<u>C</u>HCH_2), 52.0 (<u>C</u>H_3), 40.0 (CH<u>C</u>H_2) ppm.

HRMS (ESI) m/z calcd. for $C_{17}H_{16}IN_2O_3$ ($[M+H]^+$) 423.0200, found 423.0198

FT-IR (thin film) ν_{max} 3213, 3064, 2950, 2544, 2160, 2032, 1734, 1677, 1603, 1503, 1485, 1449, 1419, 1396, 1358, 1296, 1251, 1207, 1148, 1059, 1037, 1009, 919, 828, 755, 722 cm^{-1}.

m.p.: 57-59 °C

$[\alpha]_D^{25}$ = -92.6 (c=0.94, $CHCl_3$).

Chiral HPLC: Chiralcel OD, hexane/isopropanol = 70/30, 1.0 ml/min, λ = 254 nm, t_R (major) = 10.3 min, t_R (minor) = 8.2 min.

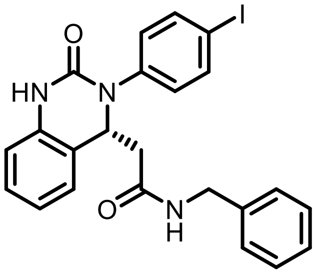

(*R*)-*N*-benzyl-2-(3-(4-iodophenyl)-2-oxo-1,2,3,4-tetrahydroquinazolin-4-yl)acetamide (4) was prepared according to the following procedure. Trifluoroacetic acid (3 mL, 7 mL/mmol) was added dropwise to a solution of the *tert*-butyl (*R*)-2-(3-(4-iodophenyl)-2-oxo-1,2,3,4-tetrahydroquinazolin-4-yl)acetate (**2j**) (200 mg, 0.43 mmol, 1.0 eq, 99.5:0.5 er) in CH_2Cl_2 (6 mL, 2 x Volume of trifluoroacetic acid) under N_2 at 0 °C. The reaction mixture was warmed to room temperature and stirred for 5 hours. Volatiles were removed under a stream of nitrogen gas to afford (*R*)-2-(3-(4-iodophenyl)-2-oxo-1,2,3,4-tetrahydroquinazolin-4-yl)acetic acid which was used as crude for next step without any purification.

Oxalyl chloride (81.9 mg, 0.65 mmol, 1.5 eq) and a few drops of DMF was added dropwise to a solution of the (*R*)-2-(3-(4-iodophenyl)-2-oxo-1,2,3,4-tetrahydroquinazolin-4-yl)acetic acid in CH_2Cl_2 (0.9 mL, 0.5M) under N_2 at room temperature. The reaction mixture was stirred at room temperature for 2 hours. Volatiles were removed under a stream of nitrogen gas to afford (*R*)-2-(3-(4-iodophenyl)-2-oxo-1,2,3,4-tetrahydroquinazolin-4-yl)acetyl chloride which was used as crude for next step without any purification.

Benzylamine (3 mL) and Et_3N (3.8 mL) was added to a solution of the afford (*R*)-2-(3-(4-iodophenyl)-2-oxo-1,2,3,4-tetrahydroquinazolin-4-yl)acetyl chloride in CH_2Cl_2 (5 mL) under N_2 at room temperature. The reaction mixture was stirred at room temperature for 24 hours before quenching with H_2O (50 mL). The aqueous layer was extracted with CH_2Cl_2 (3 x 50 mL). The combined organic layers were washed with 1M HCl aqueous solution (50 mL), saturated aqueous solution of $NaHCO_3$ (50 mL) and H_2O (50 mL). The resulting mixture was dried over Na_2SO_4, filtered and evaporated to dryness under reduced pressure. Purification by silica gel chromatography (pentane/EtOAc = 2/3) afforded the title compound as an off-white solid (77.3 mg, 36%, >99.5:0.5 er).

^{1}H NMR (400 MHz, $CDCl_3$) δ 8.19 (s, 1H, ArNH), 7.68 (d, *J* = 8.1 Hz, 2H, Ar-H), 7.30 – 7.11 (m, 7H, Ar-H), 7.08 – 7.00 (m, 2H, Ar-H), 6.94 (t, *J* = 7.5 Hz, 1H, Ar-H), 6.73 (d, *J* = 7.9 Hz, 1H, Ar-H), 5.78 (t, *J* = 5.7 Hz, 1H, NHCH$_2$), 5.30 (dd, *J* = 9.1, 4.0 Hz, 1H, CH), 4.29 (dd, *J* = 14.7, 4.9 Hz, 1H, NHCH$_2$), 4.17 (dd, *J* = 14.6, 4.3 Hz, 1H, NHCH$_2$), 2.75 (dd, *J* = 13.7, 4.3 Hz, 1H, CHCH$_2$), 2.52 (dd, *J* = 13.6, 9.1 Hz, 1H, CHCH$_2$) ppm.

^{13}C NMR (101 MHz, $CDCl_3$) δ 168.6 (C(O)NH), 153.7 (NC(O)NH), 140.6 (ArC), 138.4 (ArCH), 137.7 (ArC), 136.0 (ArC), 129.0 (ArCH), 128.9 (ArCH), 128.8 (ArCH), 127.9 (ArCH), 127.7 (ArCH), 126.2 (ArCH), 122.7 (ArCH), 121.4 (ArC), 114.4 (ArCH), 91.6 (CI), 60.0 (CH), 43.7 (NHCH$_2$), 41.9 (CHCH$_2$) ppm.

HRMS (ESI) m/z calcd. for $C_{23}H_{21}IN_3O_2$ ($[M+H]^+$) 498.0673, found 498.0673

FT-IR (thin film) ν_{max} 3020, 2542, 2160, 2032, 1978, 1676, 1602, 1485, 1451, 1215, 1009, 929, 745, 669, 629 cm^{-1}.

m.p.: 108-112 °C

$[\alpha]_D^{25}$ = -124.2 (c=0.27, $CHCl_3$).

Chiral HPLC: Chiralcel OD, hexane/isopropanol = 80/20, 1.0 ml/min, λ = 240 nm, t_R = 17.2 min, (minor enantiomer not observed), >99.5:0.5 er.

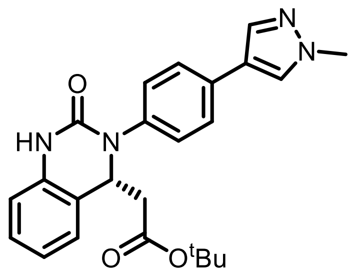

***tert*-Butyl (*R*)-2-(3-(4-(1-methyl-1H-pyrazol-4-yl)phenyl)-2-oxo-1,2,3,4-tetrahydroquinazolin-4-yl)acetate (5)** was prepared according to the following procedure. To an oven-dried screw-cap vial equipped with a magnetic stirrer bar was added *tert*-butyl (*R*)-2-(3-(4-iodophenyl)-2-oxo-1,2,3,4-tetrahydroquinazolin-4-yl)acetate (**2j**) (200 mg, 0.43 mmol, 1.0 eq, 99.5:0.5 er), 1-methyl-1H-pyrazole-3-boronic acid pinacol ester (107.4 mg, 0.52 mmol, 1.2 eq), Pd(dppf)Cl_2·CH_2Cl_2 (17.6 mg, 0.02 mmol, 0.05 eq), Cs_2CO_3 (280.2 mg, 0.86 mmol, 2.0 eq), 1,4-dioxane (3.5 mL, 0.13M) and H_2O (0.6 mL, 0.7M). The reaction mixture was stirred under air at 110 °C for 23 hours. After cooled to room temperature, the mixture was extracted with CH_2Cl_2 (3 x 50 mL). The combined organic layers were washed with brine (50 mL). The resulting mixture was dried over Na_2SO_4, filtered and evaporated to dryness under reduced pressure. Purification by silica gel chromatography (pentane/EtOAc = 3:7 to EtOAc) afforded the title compound as an off-white solid (159.4 mg, 89%, >99.5:0.5 er).

^{1}H NMR (400 MHz, $CDCl_3$) δ 8.03 (s, 1H, N<u>H</u>), 7.76 (s, 1H, NNC<u>H</u>), 7.61 (s, 1H, NNC<u>H</u>), 7.51 (d, *J* = 8.0 Hz, 2H, Ar-<u>H</u>), 7.41 (d, *J* = 8.0 Hz, 2H, Ar-<u>H</u>), 7.24 – 7.15 (m, 2H, Ar-<u>H</u>), 6.97 (td, *J* = 7.5, 0.9 Hz, 1H, Ar-<u>H</u>), 6.77 (d, *J* = 7.8 Hz, 1H, Ar-<u>H</u>), 5.23 (dd, *J* = 8.5, 4.5 Hz, 1H, NC<u>H</u>CH_2), 3.96 (s, 3H, C<u>H</u>$_3$), 2.79 (dd, *J* = 14.8, 4.5 Hz, 1H, NCHC<u>H</u>$_2$), 2.71 (dd, *J* = 14.8, 8.5 Hz, 1H, NCHC<u>H</u>$_2$), 1.32 (s, 9H, OC(C<u>H</u>$_3$)$_3$) ppm.

^{13}C NMR (101 MHz, $CDCl_3$) δ 169.4 (<u>C</u>(O)OC(CH_3)$_3$), 153.6 (<u>C</u>(O)NH), 139.1 (Ar<u>C</u>), 136.7 (N-Ar<u>C</u>H), 136.5 (Ar<u>C</u>), 131.3 (Ar<u>C</u>), 128.9 (Ar<u>C</u>H), 128.0 (Ar<u>C</u>H), 127.4 (N-Ar<u>C</u>H), 126.5 (Ar<u>C</u>H), 126.1 (Ar<u>C</u>H), 122.8 (Ar<u>C</u>), 122.3 (Ar<u>C</u>H), 121.4 (Ar<u>C</u>), 114.3 (Ar<u>C</u>H), 81.4 (O<u>C</u>(CH_3)$_3$), 60.1 (N<u>C</u>HCH_2), 41.2 (NCH<u>C</u>H$_2$), 39.3 (<u>C</u>H$_3$), 28.0 (OC(<u>C</u>H$_3$)$_3$) ppm.

HRMS (ESI) m/z calcd. for $C_{24}H_{27}N_4O_3$ ([M+H]$^+$) 419.2078, found 419.2073

FT-IR (thin film) ν_{max} 3221, 2978, 2528, 2161, 2034, 1977, 1722, 1673, 1604 1572, 1505, 1452, 1416, 1367, 1301, 1213, 1142, 987, 956, 840, 755, 626 cm^{-1}.

m.p.: 87-89 °C

$[\alpha]_D^{25}$ = -89.2 (c=0.58, $CHCl_3$).

Chiral HPLC: Chiralcel OJ, 98/2 to 70/30 gradient hexane/isopropanol, 1.0 ml/min, λ = 280 nm, t_R = 38.7 min, (minor enantiomer not observed), >99.5:0.5 er.

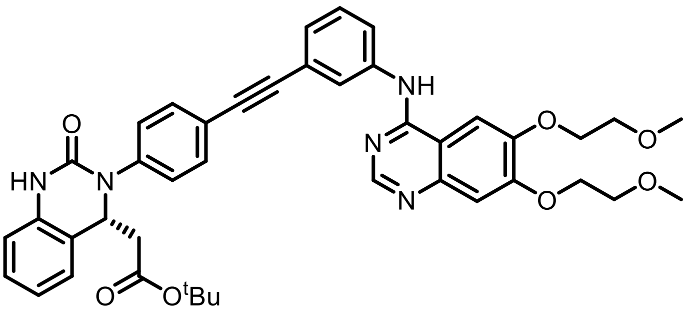

***tert*-Butyl (*R*)-2-(3-(4-((3-((6,7-bis(2-methoxyethoxy)quinazolin-4-yl)amino)phenyl)ethynyl)phenyl)-2-oxo-1,2,3,4-tetrahydroquinazolin-4-yl)acetate (6)** was prepared according to the following procedure. To an oven-dried screw-cap vial equipped with a magnetic stirrer bar was added *tert*-butyl (*R*)-2-(3-(4-iodophenyl)-2-oxo-1,2,3,4-tetrahydroquinazolin-4-yl)acetate (**2j**) (200 mg, 0.43 mmol, 1.0 eq, 99.5:0.5 er), erlotinib (HCl complex) (172.7 mg, 0.43 mmol, 1.0 eq), copper iodide (16.4 mg, 0.09 mmol, 0.2 eq), bis(triphenylphosphine) palladium dichloride (30.2 mg, 0.043 mmol, 0.1 eq), triphenylphosphine (56.4 mg, 0.22 mmol, 0.5 eq) and anhydrous triethylamine (1.5 mL, 0.3M). The vial was closed, evacuated and back-filled with nitrogen three times before heating to 90 ºC for 18 hours. After cooled to room temperature, the reaction mixture was quenched with water (10 mL) and extracted with EtOAc (3 x 50 mL). The combined organic layers were washed with brine (100 mL), dried over Na_2SO_4, filtered and evaporated to dryness under reduced pressure. Purification by silica gel chromatography (CH_2Cl_2/MeOH = 95:5) afforded the title compound as a pale yellow solid (155.1 mg, 50%, 98:2 er).

^{1}H NMR (500 MHz, $CDCl_3$) δ 8.61 (s, 1H, NC<u>H</u>N), 8.39 (s, 1H, N<u>H</u>), 8.18 (s, 1H, N<u>H</u>), 7.90 (s, 1H, Ar-<u>H</u>), 7.72 – 7.61 (m, 2H, Ar-<u>H</u>), 7.57 – 7.49 (m, 1H, Ar-<u>H</u>), 7.44 (ddd, *J* = 8.8, 7.1, 3.0 Hz, 1H, Ar-<u>H</u>), 7.38 (d, *J* = 8.1 Hz, 2H, Ar-<u>H</u>), 7.32 – 7.27 (m, 2H, Ar-<u>H</u>), 7.25 – 7.18 (m, 2H, Ar-<u>H</u>), 7.16 (d, *J* = 7.5 Hz, 1H, Ar-<u>H</u>), 7.00 – 6.94 (m, 1H, Ar-<u>H</u>), 6.81 (d, *J* = 8.0 Hz, 1H, Ar-<u>H</u>), 5.21 (dd, *J* = 8.1, 4.6 Hz, 1H, NC<u>H</u>), 4.28 (t, *J* = 4.8 Hz, 2H, OC<u>H</u>$_2$CH$_2$O), 4.19 (d, *J* = 5.3 Hz, 2H, OCH$_2$C<u>H</u>$_2$O), 3.78 (dq, *J* = 19.7, 6.3, 5.0 Hz, 4H, OC<u>H</u>$_2$C<u>H</u>$_2$O), 3.42 (s, 3H, C<u>H</u>$_3$), 3.41 (s, 3H, C<u>H</u>$_3$), 2.69 (qd, *J* = 14.9, 6.3 Hz, 2H, NCHC<u>H</u>$_2$), 1.30 (s, 9H, OC(C<u>H</u>$_3$)$_3$) ppm.

^{13}C NMR (126 MHz, CDCl$_3$) δ 169.2 ($\underline{C}$(O)OC(CH$_3$)$_3$), 156.7 (NH$\underline{C}$=N or Ar$\underline{C}$), 154.6 (NH$\underline{C}$=N or Ar$\underline{C}$), 153.5 ($\underline{C}$(O)NH), 153.3 (N$\underline{C}$HN), 148.8 (Ar$\underline{C}$), 140.4 (Ar$\underline{C}$), 139.0 (Ar$\underline{C}$), 136.2 (Ar$\underline{C}$), 132.5 (Ar$\underline{C}$H), 132.2 (Ar$\underline{C}$H), 132.1 (Ar$\underline{C}$H), 129.0 (Ar$\underline{C}$H), 128.9 (Ar$\underline{C}$H), 128.7 (Ar$\underline{C}$), 128.6 (Ar$\underline{C}$H), 127.4 (Ar$\underline{C}$H), 127.3 (Ar$\underline{C}$H), 126.1 (Ar$\underline{C}$H), 125.2 (Ar$\underline{C}$H), 123.6 (Ar$\underline{C}$), 122.5 (Ar$\underline{C}$H), 122.4 (Ar$\underline{C}$H), 121.9 (Ar$\underline{C}$), 121.1 (Ar$\underline{C}$), 114.3 (Ar$\underline{C}$H), 109.4 (Ar$\underline{C}$), 103.5 (Ar$\underline{C}$H), 90.1 (C≡$\underline{C}$), 88.8 ($\underline{C}$≡C), 81.5 (O$\underline{C}$(CH$_3$)$_3$), 70.9 (O$\underline{C}$H$_2$CH$_2$O), 70.5 (O$\underline{C}$H$_2$CH$_2$O), 69.2 (OCH$_2\underline{C}$H$_2$O), 68.4 (OCH$_2\underline{C}$H$_2$O), 59.9 (N$\underline{C}$HCH$_2$), 59.344 ($\underline{C}$H$_3$), 59.336 ($\underline{C}$H$_3$), 41.2 (NCH$\underline{C}$H$_2$), 28.0 (OC($\underline{C}$H$_3$)$_3$) ppm.

HRMS (ESI) m/z calcd. for $C_{42}H_{44}N_5O_7$ ([M+H]$^+$) 730.3235, found 730.3227

FT-IR (thin film) ν_{max} 3019, 2452, 2160, 2032, 1977, 1722, 1671, 1622, 1602, 1578, 1510, 1434, 1369, 1291, 1215, 1143, 1033, 931, 843, 748, 668, 630 cm^{-1}.

m.p.: 148-152 °C

$[\alpha]_D^{25}$ = -32.0 (c=0.60, CHCl$_3$).

Chiral HPLC: Chiralcel AD-H, hexane/isopropanol = 90/10, 1.0 ml/min, λ = 220 nm, t_R (major) = 27.1 min, t_R (minor) = 26.0 min.

8. Single Crystal X-Ray Diffraction Data

X-ray diffraction data has been made available in the Cambridge Crystallographic Data Centre as **CCDC 2054508**

8.1 Preparation of single crystal sample: crystal preparation under air led to decomposition of **2j**. Anhydrous CH_2Cl_2 and pentane were degassed under N_2 for 30 minutes before being used for the crystal preparation. Material **2j** (99.5:0.5 er) was dissolved in minimum volume of CH_2Cl_2 under N_2 before carefully layering with pentane (approximately 3 x volume of CH_2Cl_2) in a vial equipped with a suba seal. The vial was closed, wrapped with aluminum foil and left at room temperature for a few hours to afford colourless crystal of enantiopure **2j** for single crystal X-ray diffraction experiment.

8.2 General X-ray Crystallography Methods

Single crystal X-ray diffraction data were collected using an Rigaku Oxford Diffraction SuperNova diffractometer fitted with an Oxford Cryosystems Cryostream 700 plus open flow nitrogen cooling device.[12] The CrysAlisPro software was used for data collection and integration. The structure was solved using charge flipping[13] with SuperFlip method[14] within the CRYSTALS suite.[15] The structures were then modified, improved and optimized by full-matrix least squares on F2 as per the SI (CIF). Full refinement details are given in the Supporting Information (CIF); Crystallographic data have been deposited with the Cambridge Crystallographic Data Centre (CCDC 2054508) and can be obtained via www. ccdc.cam.ac.uk/data_request/cif.

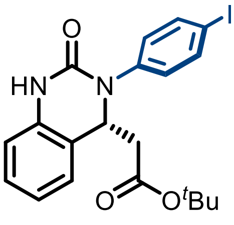

2j

≡

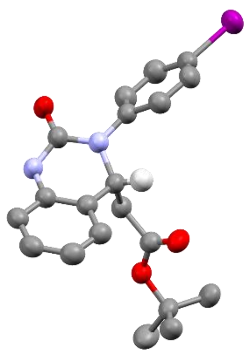

Table S4. Crystal data and structure refinement for 7355.

Identification code	7355	
Empirical formula	C20 H21 I N2 O3	
Formula weight	464.30	
Temperature	150 K	
Wavelength	1.54184 Å	
Crystal system	Monoclinic	
Space group	P 2_1	
Unit cell dimensions	a = 13.46500(10) Å	α = 90°.
	b = 9.55400(10) Å	β = 93.8204(9)°.
	c = 15.72860(10) Å	γ = 90°.
Volume	2018.90(3) $Å^3$	
Z	4	
Density (calculated)	1.527 Mg/m^3	
Absorption coefficient	12.635 mm^{-1}	
F(000)	928	
Crystal size	0.23 x 0.10 x 0.08 mm^3	
Theta range for data collection	2.816 to 76.574°.	
Index ranges	-16<=h<=16, -11<=k<=11, -19<=l<=19	
Reflections collected	22597	
Independent reflections	8251 [R(int) = 0.031]	
Completeness to theta = 75.043°	99.9 %	
Absorption correction	Semi-empirical from equivalents	
Max. and min. transmission	0.36 and 0.03	
Refinement method	Full-matrix least-squares on F^2	
Data / restraints / parameters	8251 / 1 / 470	
Goodness-of-fit on F^2	1.0586	
Final R indices [I>2sigma(I)]	R1 = 0.0748, wR2 = 0.1730	
R indices (all data)	R1 = 0.0752, wR2 = 0.1746	
Absolute structure parameter	0	
Largest diff. peak and hole	1.58 and -0.66 e.$Å^{-3}$	

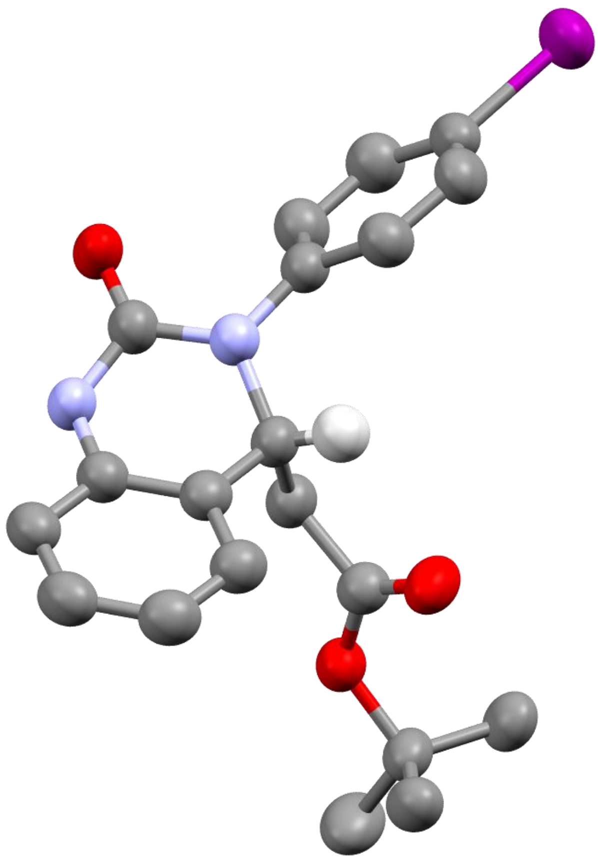

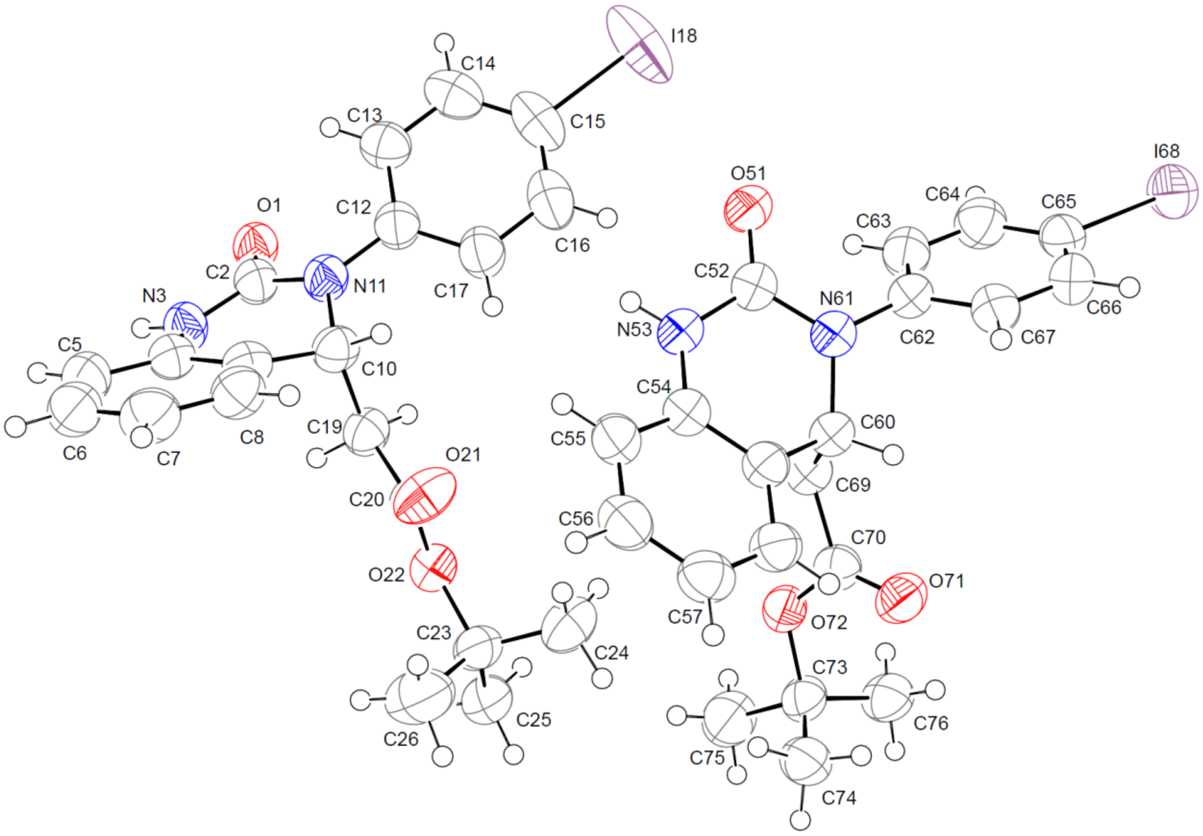
I18
C14
C13
C15
O51
I68
C64
C65
C63
O1
C12
C16
C52
N61
C2
N11
N3
C17
N53
C62
C67
C66
C5
C10
C54
C60
C19
C55
C8
C6
C7
O21
C69
C20
C56
C70
O22
O71
C57
O72
C23
C24
C73
C26
C25
C76
C75
C74

9. Computational Studies

9.1 Details of TS studies

The highly flexible nature of the BIMP squaramide catalyst prompted us to perform a conformational analysis on model substrates of the catalyst (Figure S3). For this purpose, we explored the conformation spaces of the "*left arm* – *LA*" (side containing the amide moiety with the *tert*-butyl group) and "*right arm* – *RA*" (side with the iminophosphorane moiety with phenyl group) of the organocatalyst. Regarding the *left arm*, two conformations are possible which form a hydrogen bonding between the amide and the squaramide, and the model substrate **LA-1** with O(amide)–H(squaramide) interaction was preferred over **LA-2** with O(squaramide)–H(amide) (ΔG = 1.6 kcal mol^{-1}). On the other hand, the *right arm* of the catalyst can have the iminophosphorane located either on the top or bottom side of the squaramide plane. Similar energies between **RA-1** and **RA-2** were observed (ΔG = 0.1 kcal mol^{-1}). Taken altogether, these findings indicate that the various conformations of the catalyst are likely assessable. Below we detail the reactivity for every possible conformation of the BIMP squaramide catalyst.

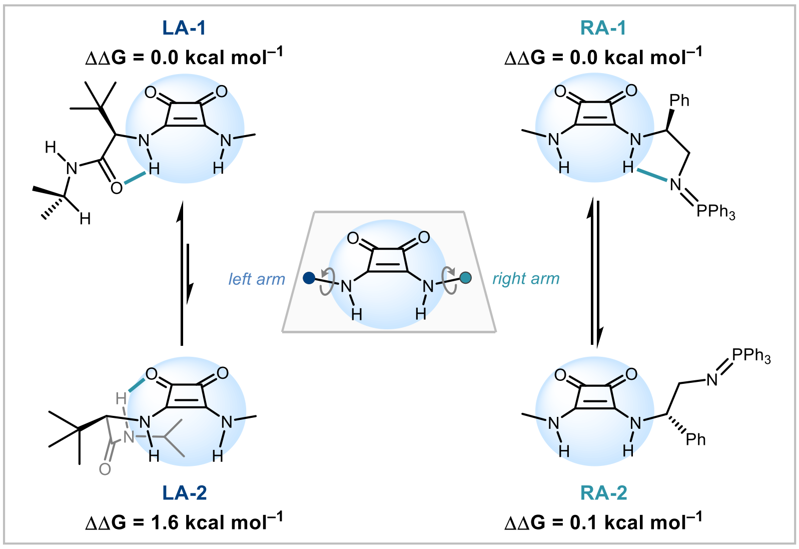

Figure S3. Relative stability of the model squaramide catalyst computed at COSMO(toluene)-ZORA-M06-2X/TZ2P//COSMO(toluene)-ZORA-BLYP-D3(BJ)/DZP. Energies (kcal mol^{-1}) are provided in the insert.

In general, there are two modes for which the substrate may bind to the substrate in the transition structures (TSs, Mode A and Mode B) as originally hypothesized by Pápai (Figure S4).[16] In the mode A, the squaramide coordinates to the electrophile (ester) and the protonated iminophosphorane coordinates

to the nucleophile (urea). In the mode B, the squaramide coordinates to the nucleophile (urea) and the protonated iminophosphorane coordinates to the electrophile (ester). Both activation modes were considered in the calculations of the TSs described below.

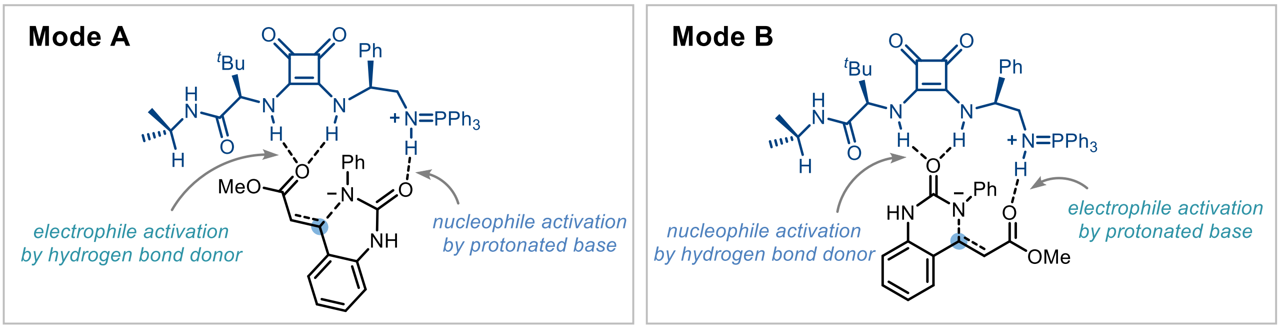

Figure S4. Activation modes of the BIMP catalyst.

Due to the conformational freedom and the existence of two potential activation modes of the BIMP catalyst, we computed and compared all the possible TSs for the stereoenantiochemistry-determining Michael reaction step involving substrate 1ae. (Figure S5).

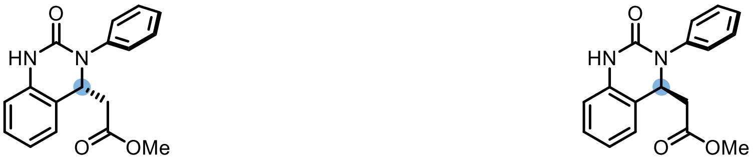

(R)-product ***(S)-product***

TSs for (R)-product	$\Delta G^‡$	$\Delta E^‡$
**TS–ModeA–LA1–RA1–*R***	**15.1**	**–7.7**
**TS–ModeA–LA1–RA2–*R***	18.0	–4.2
**TS–ModeA–LA2–RA1–*R***	16.6	–6.7
**TS–ModeA–LA2–RA2–*R***	29.3	4.8
**TS–ModeB–LA1–RA1–*R***	15.4	–6.5
**TS–ModeB–LA1–RA2–*R***	24.0	–2.2
**TS–ModeB–LA2–RA1–*R***	16.7	–6.2
**TS–ModeB–LA2–RA2–*R***	27.2	2.1

TSs for (S)-product	$\Delta G^‡$	$\Delta E^‡$
**TS–ModeA–LA1–RA1–*S***	27.7	3.3
**TS–ModeA–LA1–RA2–*S***	17.6	–6.5
**TS–ModeA–LA2–RA1–*S***	25.0	–0.8
**TS–ModeA–LA2–RA2–*S***	**16.3**	**–7.3**
**TS–ModeB–LA1–RA1–*S***	18.2	–6.3
**TS–ModeB–LA1–RA2–*S***	19.7	–1.0
**TS–ModeB–LA2–RA1–*S***	20.7	–3.3
**TS–ModeB–LA2–RA2–*S***	21.3	–1.7

Figure S5. Relative stability of the possible intramolecular aza-Michael reaction transition structures computed at COSMO(toluene)-ZORA-M06-2X/TZ2P//COSMO(toluene)-ZORA-BLYP-D3(BJ)/DZP. Energies (kcal mol^{-1}) are provided in the insert.

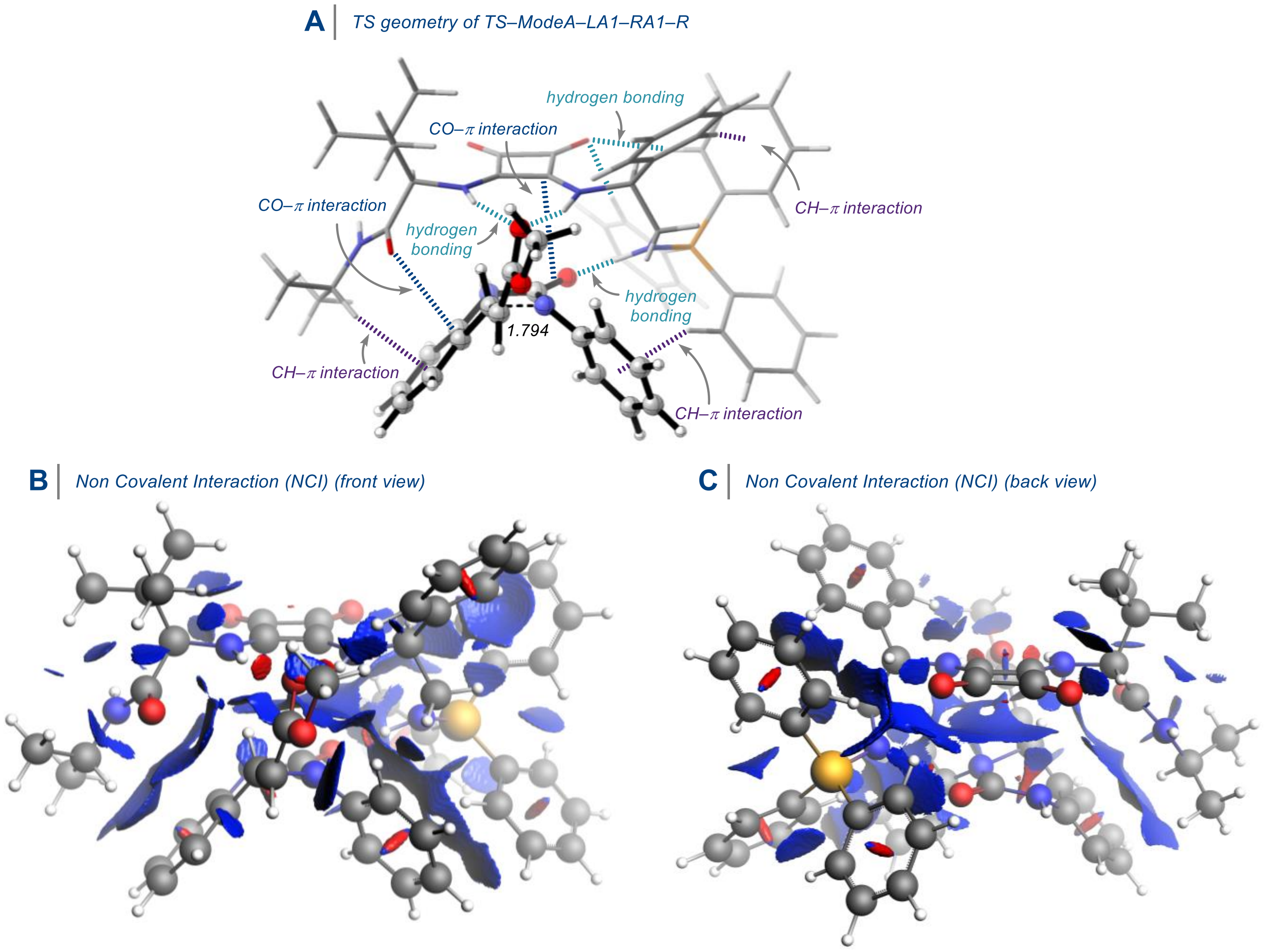

Scheme S5. (A) Transition structure geometry of **TS–ModeA–LA1–RA1–R**, (B) ADFview2020 plot of non-covalent interaction (NCI) regions materialized by reduced density gradient isosurfaces (all default settings were used) colored according to the sign of the signed density *λ2ϱ* (red and blue colors are associated with negatively and positively signed terms) for the transition structure geometry of **TS–ModeA–LA1–RA1–R** from the from view and (C) ADFview2020 plot of noncovalent interaction (NCI) for the transition structure geometry of **TS–ModeA–LA1–RA1–R** from the back view computed at COSMO(toluene)-ZORA-M06-2X/TZ2P//COSMO(toluene)-ZORA-BLYP-D3(BJ)/DZP. A forming bond length (Å) of TS geometry is provided in the insert.

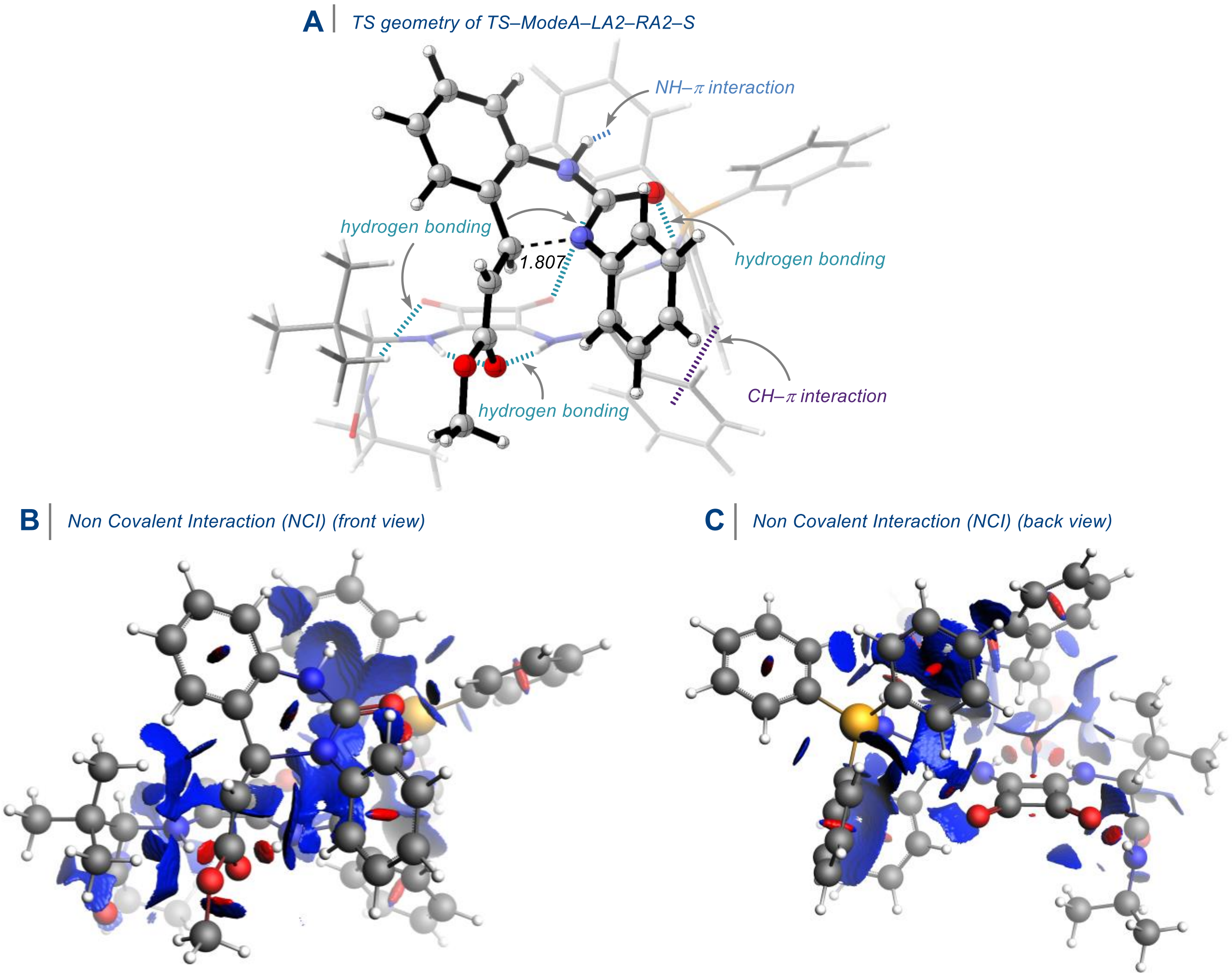

Scheme S6. (A) Transition structure geometry of **TS–ModeA–LA2–RA2–S**, (B) ADFview2020 plot of non-covalent interaction (NCI) regions materialized by reduced density gradient isosurfaces (all default settings were used) colored according to the sign of the signed density *λ2ϱ* (red and blue colors are associated with negatively and positively signed terms) for the transition structure geometry of **TS–ModeA–LA2–RA2–S** from the from view and (C) ADFview2020 plot of noncovalent interaction (NCI) for the transition structure geometry of **TS–ModeA–LA2–RA2–S** from the back view computed at COSMO(toluene)-ZORA-M06-2X/TZ2P//COSMO(toluene)-ZORA-BLYP-D3(BJ)/DZP. A forming bond length (Å) of TS geometry is provided in the insert.

Table S5. Cartesian coordinates (in Å), energies (in kcal mol^{-1}), and number of imaginary frequencies of all stationary points, computed at COSMO(toluene)-ZORA-M06-2X/TZ2P//COSMO(toluene)-ZORA-BLYP-D3(BJ)/DZP.

LA-1

COSMO(toluene)-ZORA-M06-2X/TZ2P//COSMO(toluene)-ZORA-BLYP-D3(BJ)/DZP

E = -8181.81

G = -7993.69

N_{imag} = 0

C	1.02607602	-1.13652637	-3.87229419
C	1.39718648	-2.41751193	-4.64555062
C	2.02945575	-1.53659228	-2.85133557
C	2.36533902	-2.71726597	-3.55134596
O	0.24783170	-0.18895296	-4.00108868
O	1.06328527	-2.98106841	-5.68848355
N	3.22911281	-3.71392999	-3.33394415
N	2.46461092	-0.97453309	-1.71839814
C	1.88655118	0.26517389	-1.18035373
C	1.64678561	0.00087021	0.31850215
H	0.93359059	0.43783135	-1.69745046
C	2.81489056	1.50945330	-1.45487963
H	3.00170107	-1.51114453	-1.03090726
C	3.33221004	-4.88972107	-4.21110093
H	2.69271425	-4.72278369	-5.08273315
N	0.59871935	0.65550588	0.86610582
C	0.22340231	0.53747097	2.28947152
H	3.80536446	-3.69536965	-2.49184665
H	-0.01713655	1.18771940	0.25292513
C	-0.68342719	-0.68388387	2.50910210
C	-0.42810043	1.84740760	2.74469786
H	1.16690251	0.38302114	2.83252270
H	-0.91471489	-0.79967360	3.57749589
H	-0.18334476	-1.59577344	2.15799215
H	-1.62835884	-0.56336613	1.95875400
H	1.98576099	2.79873559	0.12551727
H	1.11780837	2.90046667	-1.43153966
H	2.70729696	3.67130483	-1.24383258
H	4.05686602	1.30233674	0.34742859
H	4.81956830	2.20536830	-0.98701297
H	4.68549215	0.43518750	-1.07383776
H	3.63255604	2.50322331	-3.20950373
H	2.07611352	1.68132796	-3.51466607

H	3.57294777	0.73273914	-3.36393368
H	-0.68858167	1.78728594	3.80989747
H	-1.35478643	2.03790968	2.18112215
H	0.25257451	2.69719139	2.59878377
C	2.10922651	2.79202893	-0.96489317
C	3.03407000	1.60964678	-2.98157324
C	4.17345177	1.34977493	-0.74347047
O	2.40148023	-0.75313359	0.95849775
H	2.99812958	-5.79252481	-3.68240766
H	4.37036914	-5.02378058	-4.53974572

LA-2

COSMO(toluene)-ZORA-M06-2X/TZ2P//COSMO(toluene)-ZORA-BLYP-D3(BJ)/DZP

E = -8181.27

G = -7992.14

N_{imag} = 0

C	-0.36202093	-3.08228059	-6.47323294
C	-0.76346998	-1.60961205	-6.64442330
C	-0.24157402	-2.82526116	-5.01778024
C	-0.61001022	-1.46661403	-5.16392401
O	-0.16285345	-4.05537305	-7.21153535
O	-1.05033054	-0.84853706	-7.56618259
N	-0.73610707	-0.44085470	-4.32271948
N	0.07487736	-3.59751318	-3.96215530
C	0.67442124	-4.93900665	-4.16741756
C	2.16559800	-4.71219423	-4.52710930
H	0.16282085	-5.36286920	-5.04045263
C	0.40110780	-5.88708632	-2.96599264
H	0.33529984	-3.13396732	-3.08994120
C	-1.14139754	0.90727146	-4.75152550
H	-0.36683685	1.63583641	-4.48059702
N	2.39603500	-4.75649864	-5.86306552
C	3.63393161	-4.22528066	-6.46562735
H	-0.56930030	-0.59121760	-3.32684324
H	1.57204333	-4.79443907	-6.47610558
C	3.52431217	-2.69646135	-6.61037500
C	3.88221550	-4.92794383	-7.80412763
H	4.44256376	-4.46374630	-5.76068575
H	4.45589675	-2.27973627	-7.01963733
H	3.33595352	-2.23606045	-5.63115734
H	2.69762753	-2.43609465	-7.28748868
H	2.25719559	-7.03419250	-3.19733453

H	0.90677665	-7.62688045	-4.20178593
H	0.91838968	-7.94005407	-2.44940874
H	1.90306783	-4.99402306	-1.64577288
H	0.68720470	-6.01098445	-0.81581608
H	0.24493632	-4.38903170	-1.35872176
H	-1.35431489	-6.83004698	-2.07575438
H	-1.44411685	-6.68006645	-3.84905727
H	-1.69250658	-5.24660735	-2.82108788
H	4.81249849	-4.56069821	-8.25842628
H	3.05779258	-4.72735475	-8.50528301
H	3.96458350	-6.01441054	-7.66356613
C	1.17220922	-7.20102267	-3.22246262
C	-1.11653116	-6.17660197	-2.92732561
C	0.84458766	-5.27524441	-1.61797648
O	3.02746982	-4.44399501	-3.67369062
H	-2.08955077	1.18625838	-4.27414936
H	-1.26941333	0.89777168	-5.83751105

RA-1

COSMO(toluene)-ZORA-M06-2X/TZ2P//COSMO(toluene)-ZORA-BLYP-D3(BJ)/DZP

E = -13314.81

G = -13038.16

N_{imag} = 0

C	0.60966236	-1.07340398	-3.49640520
C	0.80404882	-2.31481986	-4.39044765
C	1.64563579	-1.64510748	-2.59236819
C	1.81989967	-2.78457399	-3.40917300
O	-0.08787320	-0.05690963	-3.48873922
O	0.33746442	-2.76394626	-5.43862031
N	2.60722107	-3.86002603	-3.34159721
N	2.18760916	-1.24053763	-1.43590581
C	1.87440685	0.05649510	-0.81866019
H	0.06790440	-10.62712603	-1.69383175
H	1.03433742	0.49879816	-1.36208214
H	-0.00040749	-10.86994733	0.78540196
H	2.94353785	-1.79026997	-1.02705798
C	2.49372868	-5.00149945	-4.26005661
H	1.60566421	-4.84166250	-4.88576363
C	2.31271073	-6.26928681	-3.37674939
H	4.97680172	-3.77746941	-4.07083309
H	3.10504963	-4.10912911	-2.47786814
H	1.29307482	-6.24817702	-2.95058847

N	3.32874256	-6.22000684	-2.32204955
H	2.38043441	-7.14987081	-4.03722875
P	3.94119128	-7.54215526	-1.70992554
H	4.40451173	-10.51086843	-1.87191815
H	3.45163628	-8.31821800	1.13462294
H	4.02882207	-5.27397327	0.07378064
H	1.69829949	-9.71472439	2.19358554
C	4.84055635	-8.64062893	-2.87602770
C	5.49385269	-8.03345014	-3.96086643
C	6.21275959	-8.81370600	-4.86879451
C	5.97499541	-5.60114472	-6.77348115
C	6.28917387	-10.20017716	-4.69558798
C	5.64149858	-10.80910697	-3.61381475
C	4.91441370	-10.03349362	-2.70677047
H	5.42836015	-6.95708312	-4.09146286
C	6.04400224	-4.70784431	-5.70071812
C	4.91817185	-4.47744396	-4.90099904
H	2.71831881	-6.55189169	-6.45929869
H	6.70651120	-8.33691798	-5.71349511
H	6.85011026	-10.80791924	-5.40527439
H	5.69856433	-11.88903851	-3.47985297
C	5.13672205	-7.05166061	-0.43001784
C	6.25549155	-7.84724957	-0.14223713
C	7.12880563	-7.47630994	0.88475399
C	4.77010717	-6.25811667	-7.04746197
C	6.88837067	-6.31424025	1.62499388
C	5.77493498	-5.51640884	1.33505545
C	4.89946905	-5.88196875	0.31035564
H	6.45114695	-8.74708707	-0.72176731
H	7.99911055	-8.09412335	1.10359219
H	7.56961224	-6.02821494	2.42591062
H	5.58829430	-4.61024220	1.91086051
C	3.71573662	-5.14413827	-5.15852377
C	3.65141490	-6.03003746	-6.24427344
H	4.70350549	-6.95244939	-7.88464898
H	6.85182644	-5.78078996	-7.39516677
H	6.97723517	-4.18914870	-5.48089414
C	2.75686054	-8.68023878	-0.88371975
C	1.79514175	-9.33714300	-1.67401195
C	0.80752015	-10.12047642	-1.07462840
C	0.76971367	-10.25671636	0.31865243
C	1.72248429	-9.60676446	1.10946646

C	2.71191760	-8.81842617	0.51278924
H	1.82454225	-9.24646132	-2.75887361
H	2.73873457	0.73233620	-0.87818614
H	1.59506307	-0.08562585	0.23300825

RA-2

COSMO(toluene)-ZORA-M06-2X/TZ2P//COSMO(toluene)-ZORA-BLYP-D3(BJ)/DZP

E = -13312.34

G = -13038.05

$\boldsymbol{N}_{\text{imag}}$ = 0

C	1.89201250	-1.89111939	-6.54789592
C	2.31968966	-3.29998463	-6.11599639
C	1.44206741	-1.66012139	-5.15344933
C	1.83345782	-2.95821982	-4.73836699
O	1.88982050	-1.23093237	-7.58904733
O	2.80929618	-4.28454742	-6.66867168
N	1.74839172	-3.53906862	-3.53068297
N	0.86787263	-0.62756066	-4.52886212
C	0.57649048	0.65125475	-5.19438249
H	2.11800998	-7.32952391	-8.39831848
H	0.82816591	0.55035109	-6.25401561
H	0.53413843	-9.24097443	-8.14282410
H	0.67677053	-0.69424061	-3.52849940
C	2.10794029	-4.92919980	-3.15843844
H	2.20162434	-4.90936711	-2.06336966
C	3.50152321	-5.33032037	-3.70539095
H	0.96073965	-6.74315588	-1.51703203
H	1.20641256	-3.04239100	-2.82232972
H	3.45059436	-5.34852111	-4.80158624
N	3.89784575	-6.58691015	-3.07312119
H	4.20351312	-4.52236364	-3.43041428
P	4.16708215	-7.89474436	-3.91865071
H	5.23440268	-9.40886971	-6.30293959
H	2.06246612	-9.97817688	-4.18684961
H	2.74699371	-8.32031529	-1.46149775
H	0.50322788	-10.54827722	-6.02356119
C	5.79784877	-7.97936739	-4.77699980
C	6.83382805	-7.18142362	-4.26744068
C	8.09941421	-7.21076848	-4.85970461
C	-0.89912622	-7.88943053	-4.13001148
C	8.33647436	-8.03761067	-5.96384454
C	7.30507845	-8.83165675	-6.47866728

C	6.03784107	-8.80170955	-5.88999890
H	6.63218262	-6.53635591	-3.41284893
C	-0.38859756	-7.79619008	-2.83298597
C	0.56442924	-6.81936744	-2.52898428
H	0.91028952	-5.41786847	-5.61031584
H	8.89975778	-6.58636469	-4.46337886
H	9.32245941	-8.05827598	-6.42740244
H	7.48634979	-9.46931961	-7.34361084
C	4.14556238	-9.30513445	-2.76213302
C	4.88236094	-10.47268370	-3.01049099
C	4.77020886	-11.56619869	-2.14670424
C	-0.44003585	-7.01365572	-5.11885007
C	3.92369269	-11.49860483	-1.03455151
C	3.19502197	-10.33069519	-0.78020388
C	3.30404368	-9.23560760	-1.64052477
H	5.54806431	-10.52861143	-3.86889497
H	5.34620693	-12.47045733	-2.34153338
H	3.83646578	-12.35313778	-0.36387667
H	2.53977638	-10.27444087	0.08889214
C	1.02909735	-5.93908598	-3.51365275
C	0.52655246	-6.05311220	-4.81756580
H	-0.81232562	-7.09883152	-6.13810646
H	-1.63912122	-8.65111803	-4.37451145
H	-0.72893753	-8.48324677	-2.05825675
C	3.00117965	-8.35261628	-5.26440706
C	3.00467467	-7.60750291	-6.45730501
C	2.11489805	-7.92227109	-7.48410595
C	1.22358550	-8.99050637	-7.33660186
C	1.20801383	-9.72667234	-6.14871129
C	2.08625236	-9.40541464	-5.11114503
H	3.69692481	-6.77972745	-6.58973893
H	1.17775119	1.45741470	-4.75322401
H	-0.48903281	0.89412328	-5.09331999

TS–ModeA–LA1–RA1–*R*

COSMO(toluene)-ZORA-M06-2X/TZ2P//COSMO(toluene)-ZORA-BLYP-D3(BJ)/DZP

E = -25764.79

G = -25188.12

N_{imag} = 1, 192*i* cm^{-1}

C	-5.28606512	0.37238647	1.71949568
C	-6.44543735	-0.20000749	0.89280764
C	-5.24995212	1.51154386	0.76299450

C	-6.28973872	0.95183230	-0.02445461
O	-4.64017010	0.05690606	2.72546697
O	-7.20622404	-1.17944415	0.95225978
N	-6.86465886	1.36578164	-1.15236374
N	-4.56373818	2.64511679	0.67655121
C	-3.56771740	3.02490673	1.68353141
C	-2.26331508	3.37680353	0.94578947
H	-3.41377027	2.13956911	2.31346093
C	-4.08770016	4.18024870	2.60997326
H	-4.75623549	3.27836041	-0.11879789
C	-8.05138752	0.72283845	-1.71502246
H	-8.38133804	-0.02598696	-0.98460794
C	-7.69308691	-0.01537307	-3.02704503
H	-7.99116761	3.50365645	-1.99743303
H	-6.44245830	2.19905061	-1.60648275
H	-8.61800812	-0.29666961	-3.54447525
N	-6.88464745	-1.21561193	-2.77838586
H	-7.14717685	0.66325163	-3.69243797
P	-7.50103613	-2.70793345	-3.00006962
H	-8.61833912	-2.56274726	-0.30838026
H	-6.42916654	-2.70258731	-0.28301476
H	-8.75478151	-4.97122032	-4.46998620
H	-3.64447087	5.81216443	1.21236668
H	-4.85598892	6.20715058	2.46778996
H	-5.30085210	5.18557577	1.08265456
H	-5.05473520	2.74761853	3.96378808
H	-6.12808859	3.37770294	2.69330740
H	-5.68882695	4.41402377	4.07092866
H	-2.64722743	3.66888434	4.18514180
H	-3.33649244	5.30411105	4.31804440
H	-2.09204328	4.96372435	3.09301787
H	-3.44385512	-1.49090027	-6.78014919
H	-4.94662699	0.09695790	-7.99316669
H	-6.04820705	1.94653972	-6.73583668
C	-2.96917040	4.54923610	3.60792914
C	-5.31423210	3.64356263	3.38146845
C	-4.49229463	5.42237211	1.78817347
O	-2.24638062	4.16531445	-0.00772925
N	-1.15713767	2.74971718	1.42193445
C	0.19255459	2.98087912	0.86965233
H	-1.25450540	2.11587860	2.21250224
C	1.12016031	1.86614309	1.35904399

C	0.70998751	4.37746971	1.25048195
H	0.09220188	2.92699924	-0.22214155
H	2.11137497	1.97468458	0.89944054
H	0.72321851	0.88006089	1.08678949
H	1.24212257	1.91387958	2.45289430
H	1.69435516	4.55207956	0.79216505
H	0.81148924	4.46453346	2.34244667
H	0.01628977	5.14786982	0.89248247
C	-1.73507688	1.86829136	-2.38753926
C	-0.66962966	2.55615826	-2.97154005
C	0.64413002	2.09892719	-2.81555850
C	0.89717581	0.94346555	-2.06741162
C	-0.15912549	0.23098288	-1.49298360
C	-1.46784838	0.69131665	-1.65942404
H	-0.87027011	3.47963369	-3.51075124
H	1.46948022	2.65264406	-3.26172680
H	1.91911044	0.59119520	-1.93151616
H	0.02918258	-0.68157870	-0.92751943
N	-2.58101741	-0.01953760	-1.14947597
C	-4.78456336	4.06747913	-2.77554922
C	-3.15423474	2.31129804	-2.39497202
H	-3.53200719	2.31662794	-1.37673642
C	-3.60123494	3.42780233	-3.15405657
H	-3.10731793	3.72906895	-4.07296598
O	-5.55871359	3.76067677	-1.81101272
O	-5.12806190	5.14383278	-3.60020233
C	-6.35681170	5.82774582	-3.24815638
H	-6.39054482	6.71124991	-3.89357280
H	-7.22846007	5.19063868	-3.44354082
H	-6.35633411	6.12262622	-2.19260463
C	-3.70174698	-0.23284783	-1.96033844
H	-2.39380292	-0.80298647	-0.52115707
O	-4.37181916	-1.28945115	-1.81414465
N	-3.98266469	0.77773976	-2.81966443
H	-3.00827575	-1.18663332	-4.35908476
H	-5.88521455	-1.14076717	-2.42776347
C	-4.29527404	0.53805458	-4.16165185
C	-5.15605765	1.41630460	-4.84903715
C	-5.38041859	1.25612373	-6.22025879
C	-4.76804471	0.21672010	-6.92521356
C	-3.92184940	-0.67074221	-6.24421494
C	-3.67966558	-0.50770066	-4.88208304

H	-5.64478677	2.21667102	-4.30678052
C	-9.18991126	1.69959741	-1.96750259
C	-10.48712528	1.18105577	-2.10840581
C	-11.56258660	2.02377386	-2.39569071
C	-11.35232298	3.39988017	-2.55060319
C	-10.06270432	3.92104576	-2.40991025
C	-8.98435124	3.07713840	-2.11796611
H	-10.65690309	0.11283899	-1.98547981
H	-12.56554505	1.60865524	-2.49335629
H	-12.18957468	4.06043108	-2.77385417
H	-9.89236563	4.99146701	-2.52282551
C	-6.43855193	-3.87308947	-2.11759960
C	-5.94649986	-5.02043195	-2.76434871
C	-5.12386203	-5.90588639	-2.06545271
C	-4.78905845	-5.64941904	-0.73152985
C	-5.26843224	-4.49942746	-0.09601044
C	-6.08924532	-3.60517866	-0.78219143
H	-6.18484230	-5.21512972	-3.80608799
H	-4.73891068	-6.79240253	-2.56758064
H	-4.14585595	-6.34269669	-0.19040351
H	-4.99775180	-4.28632960	0.93729732
C	-7.55973265	-3.17933011	-4.75030831
C	-6.88452977	-2.36521135	-5.67147018
C	-6.87890657	-2.71075297	-7.02429957
C	-7.54022660	-3.86317017	-7.45743414
C	-8.21260819	-4.67873987	-6.53692045
C	-8.22784276	-4.33935285	-5.18354926
H	-6.36174007	-1.47629206	-5.33169062
H	-6.35375725	-2.07626073	-7.73470981
H	-7.53274282	-4.13013491	-8.51387880
H	-8.72701649	-5.57715137	-6.87554511
C	-9.22064481	-2.70257885	-2.39497654
C	-10.30394181	-2.65513280	-3.28900447
C	-11.60506590	-2.51687369	-2.79833685
C	-11.82921365	-2.42091859	-1.42202197
C	-10.74972858	-2.45402533	-0.53184735
C	-9.44646627	-2.59127568	-1.01029666
H	-10.13733909	-2.70764258	-4.36188304
H	-12.44089313	-2.47375744	-3.49538822
H	-12.84399415	-2.30714290	-1.04220056
H	-10.91821914	-2.35800682	0.53993926

TS–ModeA–LA1–RA2–*R*

COSMO(toluene)-ZORA-M06-2X/TZ2P//COSMO(toluene)-ZORA-BLYP-D3(BJ)/DZP

E = -25761.27

G = -25185.17

N_{imag} = 1, 142*i* cm^{-1}

C	-5.56800433	0.76642829	1.81987460
C	-6.98616839	0.70466091	1.24406686
C	-5.28826423	1.87728608	0.86953695
C	-6.58339214	1.78232525	0.29418627
O	-4.91459625	0.15975640	2.67549989
O	-8.02004741	0.06141774	1.47171449
N	-7.13131115	2.47215749	-0.70695689
N	-4.24498009	2.67396595	0.64251008
C	-2.99287337	2.51388383	1.39593716
C	-1.83443763	2.55755124	0.38513336
H	-3.03614466	1.52394354	1.86774408
C	-2.84036994	3.57580190	2.55171562
H	-4.28573835	3.36947073	-0.11899592
C	-8.41889543	2.15117797	-1.33695084
H	-8.60626155	2.99668223	-1.99968019
C	-9.56580951	2.17788517	-0.30399455
H	-8.72574845	-0.48776435	-0.55076914
H	-6.51765696	3.16424005	-1.20105872
H	-9.55321894	1.28393704	0.32316677
N	-10.86017897	2.33711792	-0.98447247
H	-9.41753049	3.04798710	0.35145779
P	-12.08434705	1.28426333	-1.24841585
H	-12.01622739	-1.46156659	-2.34846650
H	-10.47746625	0.18553318	-3.40220201
H	-14.48224409	-0.05642873	-0.23220443
H	-2.02047989	5.15974893	1.26850219
H	-2.71313889	5.72851447	2.81247173
H	-3.78207329	5.23031908	1.48458152
H	-4.07664076	2.39222278	3.92837600
H	-4.98462707	3.62230018	3.02090255
H	-3.92452648	4.12075913	4.35720042
H	-1.49479964	2.26604296	3.68995734
H	-1.45877147	3.97276091	4.18420578
H	-0.64548026	3.47186811	2.68554539
H	-10.11505349	9.20854151	-0.99154552
H	-8.54030923	8.88104610	0.91737693
H	-7.15819987	6.80734595	1.03213519

C	-1.52729053	3.30121000	3.31629682
C	-4.03096505	3.41190500	3.52323067
C	-2.83375207	5.00921326	1.98928202
O	-1.82241762	3.36166956	-0.55875301
N	-0.83919395	1.66381195	0.61501461
C	0.40917522	1.64195700	-0.17219775
H	-0.90460587	1.05470437	1.42874685
C	1.02311218	0.24079300	-0.09790450
C	1.37459137	2.73761758	0.30923232
H	0.11269930	1.86277392	-1.20805181
H	1.93455033	0.19690031	-0.70895440
H	0.31711025	-0.51609727	-0.46650690
H	1.29893176	-0.00911701	0.93870424
H	2.27851626	2.75039809	-0.31666611
H	1.67474456	2.55434665	1.35167704
H	0.89345425	3.72209040	0.24534147
C	-7.91896392	4.50412026	-5.11483960
C	-7.05471817	4.70673645	-6.19650035
C	-7.41993749	4.34046591	-7.49651912
C	-8.66920639	3.75309912	-7.72544517
C	-9.55709567	3.56320421	-6.66534980
C	-9.19000302	3.94847092	-5.36826627
H	-6.06968702	5.12260219	-5.99358632
H	-6.72731453	4.49998538	-8.32176832
H	-8.95932869	3.45022775	-8.73120180
H	-10.53928930	3.12612612	-6.83660743
N	-10.08719921	3.77278432	-4.30227987
C	-5.40498196	5.31105813	-2.44727947
C	-7.46125617	4.71599443	-3.69425462
H	-7.29970333	3.74605212	-3.21765436
C	-6.35811132	5.58579370	-3.43472814
H	-6.28341169	6.53340892	-3.96342428
O	-5.34048859	4.31425149	-1.65558825
O	-4.42325822	6.30038394	-2.37764710
C	-3.51533085	6.20439842	-1.25184899
H	-2.79972101	7.02219559	-1.39027294
H	-4.06282662	6.34955734	-0.31235364
H	-2.99432911	5.24346923	-1.22582115
C	-10.09630499	4.58578901	-3.16623570
H	-11.00764219	3.39299474	-4.52023099
O	-11.14873800	4.62412151	-2.46191759
N	-8.92851960	5.19368276	-2.88671035

H	-10.28585997	7.47585073	-2.77297749
H	-10.98238251	3.22615226	-1.53320748
C	-8.85468645	6.15659000	-1.85111365
C	-7.96932551	5.97493375	-0.78133111
C	-7.85574191	6.95587426	0.20808208
C	-8.62927002	8.11838413	0.14421682
C	-9.51252677	8.30238105	-0.92881274
C	-9.61615555	7.33300432	-1.92674896
H	-7.35610176	5.08277881	-0.72525832
C	-8.34352684	0.88221673	-2.18113298
C	-8.05016491	0.97752873	-3.55117358
C	-7.95405439	-0.16731470	-4.34419663
C	-8.15998200	-1.42980321	-3.77670644
C	-8.43855060	-1.53397402	-2.40987308
C	-8.52019345	-0.38740251	-1.61375174
H	-7.91461249	1.94803171	-4.01553171
H	-7.73004351	-0.06952598	-5.40615137
H	-8.09868560	-2.32582358	-4.39411470
H	-8.59961895	-2.51142300	-1.95738590
C	-12.37618157	1.15940505	-3.03588500
C	-13.51878383	1.72933117	-3.62048242
C	-13.64162617	1.75531017	-5.01303015
C	-12.63564136	1.21090207	-5.81811164
C	-11.50190969	0.63310340	-5.23429395
C	-11.36690245	0.61116129	-3.84824484
H	-14.29888966	2.16106389	-2.99831948
H	-14.52216974	2.20757010	-5.46730625
H	-12.73218208	1.24380984	-6.90291026
H	-10.70854762	0.22144501	-5.85516469
C	-13.61527966	1.91083054	-0.50825391
C	-13.79081468	3.29705183	-0.35994821
C	-14.98982333	3.78281651	0.16620255
C	-16.00206618	2.89455996	0.54652706
C	-15.82143690	1.51425124	0.40311642
C	-14.62883351	1.01715102	-0.12699285
H	-13.00321617	3.98808206	-0.65546207
H	-15.13077729	4.85639307	0.28274777
H	-16.93357794	3.27901659	0.96058965
H	-16.60705000	0.82424781	0.70799301
C	-11.70928785	-0.32253770	-0.52861976
C	-11.35670263	-0.37478339	0.83247511
C	-10.97354578	-1.58851019	1.39874569

C	-10.97629067	-2.75363265	0.62432202
C	-11.37075746	-2.70857200	-0.71572686
C	-11.72966142	-1.49418520	-1.30136040
H	-11.36147916	0.52772045	1.44010628
H	-10.65959507	-1.62047369	2.44023106
H	-10.67044959	-3.69976034	1.06935169
H	-11.38288455	-3.61799495	-1.31462288

TS–ModeA–LA2–RA1–*R*
COSMO(toluene)-ZORA-M06-2X/TZ2P//COSMO(toluene)-ZORA-BLYP-D3(BJ)/DZP
E = -25763.76
G = -25186.60
N_{imag} = 1, 193*i* cm^{-1}

C	-5.71978838	0.43427722	1.95762335
C	-6.92503284	0.01272879	1.12946840
C	-5.44170170	1.47827576	0.93541745
C	-6.56180210	1.06687253	0.15530322
O	-5.16961001	0.05780519	3.00727570
O	-7.81685433	-0.84789745	1.20312862
N	-7.04561187	1.49574121	-1.00394571
N	-4.54340381	2.44046475	0.75247113
C	-3.41345890	2.68512220	1.67487231
C	-2.27945782	1.64760234	1.38254874
H	-3.78454928	2.47552389	2.68934472
C	-2.97969285	4.17826164	1.64361664
H	-4.68998563	3.05464356	-0.06917407
C	-8.17302739	0.83669237	-1.66598129
H	-8.57740585	0.11398025	-0.94621330
C	-7.65887198	0.04968740	-2.89729543
H	-8.12916220	3.61236519	-1.88435055
H	-6.53725937	2.28091144	-1.45865651
H	-8.51606215	-0.26688403	-3.50295940
N	-6.87145435	-1.11994186	-2.49589404
H	-7.04343622	0.70433891	-3.52372994
P	-7.39618289	-2.65216551	-2.69799253
H	-8.87044557	-2.42699723	-0.17016622
H	-6.74533389	-2.41443440	0.13165741
H	-8.32722339	-5.10630220	-4.05796779
H	-1.78387860	3.98114773	-0.18435140
H	-2.14233257	5.66326637	0.29926852
H	-3.39938057	4.68058262	-0.46053504
H	-4.49590460	4.73817594	3.13258835

H	-5.03781681	4.93085362	1.44498533
H	-3.88947450	6.09382750	2.14670604
H	-2.08523802	3.99522677	3.63989674
H	-1.54549867	5.41709287	2.71491038
H	-0.92256358	3.79958120	2.29801259
H	-3.21920661	-1.27280555	-6.88184056
H	-4.85094400	0.16595419	-8.11391963
H	-6.12646695	1.91291285	-6.87287581
C	-1.80742570	4.35229611	2.63602980
C	-4.17798611	5.03298011	2.12082623
C	-2.54973030	4.64325958	0.23153554
O	-1.28049451	1.89131309	0.69193364
N	-2.54247295	0.43874988	1.93771918
C	-1.72042464	-0.75674564	1.70837668
H	-3.39477627	0.31937315	2.50553330
C	-2.63987669	-1.94684557	1.38724960
C	-0.80917447	-1.02938014	2.91701375
H	-1.07874175	-0.51379201	0.84959425
H	-2.04657251	-2.85001475	1.18537309
H	-3.28016746	-1.74538937	0.51823924
H	-3.29826599	-2.15417434	2.24338538
H	-0.16924843	-1.90360384	2.72816465
H	-1.41445659	-1.22873716	3.81379026
H	-0.16853477	-0.15897180	3.11250287
C	-1.82830955	2.07584516	-2.78001774
C	-0.90967972	2.88650993	-3.45124163
C	0.44060839	2.52523768	-3.52041340
C	0.88034857	1.34475291	-2.90931340
C	-0.02993822	0.50930282	-2.25704009
C	-1.37677637	0.87318223	-2.20877383
H	-1.24876180	3.82623733	-3.88179237
H	1.15089479	3.17139893	-4.03470781
H	1.93335458	1.06755726	-2.94713984
H	0.30135967	-0.41948045	-1.79367254
N	-2.33836505	0.03611287	-1.60633425
C	-4.97041818	4.15439000	-2.60604563
C	-3.25564722	2.41764314	-2.53860203
H	-3.46837916	2.35767361	-1.47136627
C	-3.85416513	3.54632110	-3.17613234
H	-3.50707650	3.88997069	-4.14584175
O	-5.57646236	3.81824260	-1.53369239
O	-5.43599910	5.25651952	-3.33156953

C	-6.52006691	6.00077015	-2.72240936
H	-6.57965844	6.93846773	-3.28441451
H	-7.46661295	5.45640636	-2.81348793
H	-6.31820284	6.19811349	-1.66376598
C	-3.60456586	-0.14379271	-2.12168679
H	-2.00990776	-0.72163926	-1.01104945
O	-4.25144172	-1.18102604	-1.82872673
N	-4.08917250	0.88410624	-2.90445818
H	-2.86413988	-0.95338904	-4.45445260
H	-5.86737916	-1.02153743	-2.17813514
C	-4.31584269	0.63996034	-4.26795889
C	-5.23013141	1.44994003	-4.97128447
C	-5.41309666	1.27901122	-6.34597796
C	-4.69916522	0.29913140	-7.04330855
C	-3.78624308	-0.50794729	-6.35093535
C	-3.58850476	-0.33511913	-4.98216890
H	-5.78308398	2.21378707	-4.43491640
C	-9.27957609	1.79336703	-2.07235606
C	-10.53179375	1.25312312	-2.40745448
C	-11.57114539	2.08230310	-2.83287111
C	-11.36965157	3.46505443	-2.93083634
C	-10.12674440	4.00750113	-2.59191612
C	-9.08516962	3.17635746	-2.16264632
H	-10.69658428	0.17982369	-2.32645764
H	-12.54045190	1.65151814	-3.08278462
H	-12.17928434	4.11447785	-3.26217913
H	-9.96653042	5.08322236	-2.65712337
C	-6.37245670	-3.66682693	-1.60722021
C	-5.65961409	-4.76712181	-2.11137544
C	-4.81172400	-5.48386131	-1.26376520
C	-4.67316211	-5.10711656	0.07599981
C	-5.38175627	-4.00949126	0.57392581
C	-6.22751345	-3.28297631	-0.26173519
H	-5.74225790	-5.04875745	-3.15745512
H	-4.25098033	-6.33104271	-1.65630616
H	-4.00458065	-5.66537270	0.73050558
H	-5.26837786	-3.70322540	1.61179040
C	-7.23211514	-3.26174516	-4.39679611
C	-6.53684411	-2.47378234	-5.32635809
C	-6.39942546	-2.92409389	-6.64228477
C	-6.94535990	-4.15258974	-7.02461212
C	-7.63394479	-4.94151498	-6.09336917

C	-7.78289892	-4.49852250	-4.77966904
H	-6.10435909	-1.52320369	-5.02561495
H	-5.86496062	-2.30960836	-7.36261763
H	-6.83611081	-4.49904789	-8.05180583
H	-8.05748321	-5.89914017	-6.39347282
C	-9.17573189	-2.67232155	-2.31120451
C	-10.12727654	-2.69280145	-3.34625783
C	-11.48449549	-2.55306756	-3.04523113
C	-11.89619392	-2.39256612	-1.71919048
C	-10.94926703	-2.36428460	-0.68928608
C	-9.59107240	-2.50083853	-0.97808283
H	-9.81454532	-2.79944647	-4.38174626
H	-12.21727509	-2.56071080	-3.85111151
H	-12.95460903	-2.27732213	-1.48796941
H	-11.26382951	-2.22036237	0.34340491

TS–ModeA–LA2–RA2–*R*
COSMO(toluene)-ZORA-M06-2X/TZ2P//COSMO(toluene)-ZORA-BLYP-D3(BJ)/DZP
E = -25752.29
G = -25173.96
$\boldsymbol{N}_{\mathrm{imag}}$ = 1, 165*i* cm^{-1}

C	-6.51228658	1.54644070	2.36444076
C	-7.66147578	1.44494875	1.35932180
C	-5.77177487	2.35963000	1.37194298
C	-6.80841634	2.22804300	0.40744521
O	-6.23844667	1.05280920	3.47187051
O	-8.76614058	0.89700801	1.30298241
N	-6.86068892	2.59753305	-0.86581917
N	-4.57268715	2.95661740	1.36389048
C	-3.60886705	2.64812983	2.45365866
C	-2.85888426	1.38362343	1.97016379
H	-4.22427705	2.36968457	3.31718832
C	-2.75800951	3.86461714	2.89826197
H	-4.20572694	3.27757299	0.45538895
C	-7.95638766	2.28593411	-1.78156959
H	-7.79802091	2.96692832	-2.61618858
C	-9.32344302	2.68037513	-1.17463595
H	-8.86338913	1.43310768	-4.18533728
H	-5.97526992	2.96772299	-1.30583433
H	-9.67204195	1.91630693	-0.47367753
N	-10.31806378	2.90235600	-2.24211113
H	-9.19608767	3.61730994	-0.61367010

P	-11.66009420	1.98617730	-2.51735114
H	-11.48506913	3.32922260	-5.05225666
H	-12.15641367	4.42356543	-1.02862159
H	-13.31966118	1.60495423	-0.16445695
H	-1.42321374	3.76359078	1.17400805
H	-1.28343890	5.26429810	2.13269557
H	-2.67039536	5.03515839	1.05275285
H	-4.24910325	4.48198035	4.38793467
H	-4.47160796	5.22534239	2.78362075
H	-3.15898525	5.78691486	3.85200130
H	-2.29963146	2.84566541	4.78801661
H	-1.22499609	4.21396451	4.40838060
H	-1.03587963	2.66920362	3.54041284
H	-3.97728026	7.47561374	-3.02443231
H	-5.14725473	7.87577425	-0.85668824
H	-7.28008902	6.67247467	-0.37212094
C	-1.76883515	3.36301547	3.97421163
C	-3.72124949	4.90249898	3.51869349
C	-1.98530304	4.51583600	1.73551200
O	-1.88239381	1.41869722	1.20567526
N	-3.46607443	0.23830741	2.38738222
C	-3.27476301	-1.04806617	1.69348903
H	-4.32332518	0.33614736	2.94462102
C	-4.21548065	-1.11597930	0.47463745
C	-3.50723435	-2.19922837	2.67739816
H	-2.23241991	-1.05575691	1.34621038
H	-4.02919092	-2.02630076	-0.11335136
H	-4.06609420	-0.24417812	-0.17636799
H	-5.26268746	-1.12797646	0.81023351
H	-3.36330801	-3.16502679	2.17384886
H	-4.53502616	-2.16938784	3.07104793
H	-2.80884300	-2.13488643	3.52298553
C	-6.10840767	2.05646261	-5.52494903
C	-5.24995636	1.02470075	-5.92563242
C	-5.58768329	0.14645445	-6.95880737
C	-6.80968111	0.29972122	-7.62138441
C	-7.67809751	1.32207040	-7.24489723
C	-7.33744167	2.19280280	-6.19701477
H	-4.30865509	0.91179265	-5.39104512
H	-4.90292137	-0.65222739	-7.24053771
H	-7.08919107	-0.37574825	-8.42960608
H	-8.63740088	1.44324754	-7.74639010

N	-8.28719023	3.13227280	-5.77939625
C	-3.84471226	3.45873482	-2.85680987
C	-5.75375756	2.87357613	-4.30808872
H	-6.10102212	2.35048838	-3.41496852
C	-4.39622684	3.27367007	-4.13300785
H	-3.77700107	3.49203268	-4.99995304
O	-4.39413282	3.31444456	-1.72107322
O	-2.49616424	3.81966910	-2.91798219
C	-1.85987465	4.02543787	-1.63544808
H	-0.81499267	4.26309920	-1.86176445
H	-2.32984908	4.86154618	-1.10647644
H	-1.91661240	3.12487161	-1.01354267
C	-8.15679955	4.05574991	-4.74640620
H	-9.23937719	3.02384586	-6.11735071
O	-9.19930919	4.65431765	-4.35150478
N	-6.89734938	4.21130759	-4.28279189
H	-4.90121539	5.83671328	-4.64990832
H	-10.02140087	3.55625189	-2.99798765
C	-6.54788621	5.25700985	-3.39056137
C	-7.23311248	5.53105104	-2.19567561
C	-6.73710001	6.47508446	-1.29628119
C	-5.54534083	7.15587992	-1.57139374
C	-4.88729010	6.92679064	-2.78291666
C	-5.39250190	5.99795308	-3.69606153
H	-8.16676948	5.02941699	-1.98107766
C	-7.89752543	0.86937992	-2.34581327
C	-7.31183516	-0.19249161	-1.64464542
C	-7.26516843	-1.47114535	-2.20357240
C	-7.78827006	-1.70551688	-3.48092763
C	-8.35961312	-0.64874482	-4.19436783
C	-8.41731166	0.62309749	-3.62367921
H	-6.88193468	-0.03122788	-0.66374203
H	-6.80509510	-2.28460347	-1.64335812
H	-7.73283594	-2.70053387	-3.92188235
H	-8.74676334	-0.80349541	-5.19903859
C	-13.13505072	3.01965308	-2.35057822
C	-14.32652862	2.66267925	-3.00397840
C	-15.47455425	3.43120762	-2.80705754
C	-15.43336540	4.55115283	-1.96773951
C	-14.24327632	4.91083903	-1.32652766
C	-13.08933260	4.14748514	-1.51598154
H	-14.35320626	1.79776050	-3.66417254

H	-16.40027855	3.16035797	-3.31263165
H	-16.33127986	5.15009353	-1.81956660
H	-14.21142726	5.78801094	-0.68196543
C	-11.71124097	0.67277966	-1.28527878
C	-10.78982648	-0.39082156	-1.33411740
C	-10.75656364	-1.31962078	-0.29778422
C	-11.63713673	-1.20167395	0.78151863
C	-12.56393404	-0.15600572	0.82343893
C	-12.60635649	0.78552761	-0.20614033
H	-10.08818731	-0.47745892	-2.15413708
H	-10.02446037	-2.12424306	-0.32734566
H	-11.59477159	-1.92138956	1.59791672
H	-13.24926266	-0.06383130	1.66451870
C	-11.69479555	1.33504289	-4.20894002
C	-11.82098859	-0.03322068	-4.48487185
C	-11.79489995	-0.47461282	-5.81045541
C	-11.65479482	0.44212258	-6.85461868
C	-11.55863916	1.81182111	-6.57957121
C	-11.58062052	2.26410247	-5.26079171
H	-11.93298558	-0.75172902	-3.67770203
H	-11.88037305	-1.53916477	-6.02366358
H	-11.62881498	0.09230239	-7.88581307
H	-11.47082023	2.52900347	-7.39508018

TS–ModeB–LA1–RA1–*R*

COSMO(toluene)-ZORA-M06-2X/TZ2P//COSMO(toluene)-ZORA-BLYP-D3(BJ)/DZP

E = -25763.59

G = -25187.82

N_{imag} = 1, 182*i* cm^{-1}

C	-4.70211551	2.77787646	1.87918228
C	-4.03546889	2.18130916	3.13403761
C	-5.29999248	1.43473015	1.63389618
C	-4.66372941	0.88566964	2.77203608
O	-4.72234149	3.86012682	1.28613560
O	-3.29416588	2.57638339	4.04082438
N	-4.61942388	-0.36716385	3.24115528
N	-6.11314871	0.92946404	0.70573448
C	-6.87649318	1.82036321	-0.18164144
C	-6.82140762	1.26212566	-1.61419885
H	-6.35694702	2.78609824	-0.15000033
C	-8.33898807	2.07022362	0.33089011
H	-6.39686807	-0.05611205	0.79451878

C	-4.17948522	-0.65849492	4.60430964
H	-3.54571375	0.17982095	4.90875187
C	-3.35755684	-1.96424760	4.65478551
H	-4.04962107	-0.18084727	7.25909174
H	-5.27679790	-1.01777108	2.78055382
H	-3.11750606	-2.17075749	5.70836646
N	-2.13630220	-1.94963299	3.82473480
H	-3.97266501	-2.79616045	4.28672623
P	-0.86137976	-0.95116763	4.10898185
H	0.57182644	1.64396502	4.20370834
H	1.62461936	-0.34893240	2.65057323
H	0.06579529	-2.12362887	6.64093678
H	-8.51907373	3.86364771	-0.92131710
H	-10.06978680	3.20764246	-0.34494672
H	-9.19901312	2.39084631	-1.66396589
H	-7.77032189	2.21699947	2.43436674
H	-9.25921178	3.08842415	2.02122834
H	-7.67627843	3.77521118	1.55619436
H	-9.16142746	0.16224418	-0.36305566
H	-10.11992269	0.97336649	0.91160964
H	-8.60995481	0.13190880	1.32642665
H	-1.29301158	-1.25769024	-0.73601851
H	-2.63146007	-0.43161010	-2.66938791
H	-5.10202626	-0.84944048	-2.71776389
C	-9.07226697	2.93406854	-0.71750423
C	-8.24773723	2.84181075	1.66567738
C	-9.10156138	0.74805134	0.56128842
O	-7.35701029	0.19136301	-1.93409704
N	-6.13286180	2.05152462	-2.48126345
C	-5.96050534	1.74314799	-3.90995750
H	-5.71534616	2.91123224	-2.12713797
C	-4.49119978	1.93554273	-4.30622176
C	-6.91981763	2.59235826	-4.76010337
H	-6.24024698	0.68632381	-4.01156862
H	-4.33763940	1.64550453	-5.35471344
H	-3.83730785	1.32506508	-3.67158671
H	-4.19451739	2.99067917	-4.19839621
H	-6.82039312	2.33403617	-5.82401960
H	-6.69485939	3.66305506	-4.64156447
H	-7.95910747	2.41736913	-4.45184918
C	-5.49976652	-5.69016229	1.23773253
C	-5.40401637	-6.93779311	0.61072799

C	-6.52344984	-7.76658156	0.48042617
C	-7.75908101	-7.35162268	0.99070518
C	-7.88176263	-6.10029377	1.59909353
C	-6.75962169	-5.26953057	1.70662386
H	-4.42937983	-7.27309955	0.26297642
H	-6.42718572	-8.73925803	-0.00013616
H	-8.63293261	-7.99706437	0.90795636
H	-8.84587385	-5.76040775	1.97754900
N	-6.86450707	-3.97949485	2.27167188
C	-1.85326427	-4.79757000	1.50766168
C	-4.30926349	-4.83646019	1.54835707
H	-4.19433114	-4.70942956	2.63162793
C	-3.07159524	-5.09438571	0.89084474
H	-3.05982219	-5.44439401	-0.13801471
O	-1.66270487	-4.32352691	2.67157922
O	-0.74899814	-5.06365668	0.69106708
C	0.53888146	-4.76204417	1.27916365
H	1.27477579	-5.01035894	0.50747357
H	0.61411428	-3.70233849	1.54640440
H	0.71216002	-5.36585702	2.17710284
C	-6.15401689	-2.89647119	1.73971134
H	-7.79026468	-3.69936496	2.60037073
O	-6.65644058	-1.73594490	1.84852315
N	-4.94777321	-3.19217049	1.22748700
H	-2.41081448	-2.46977706	1.11481910
H	-1.94948890	-2.85810338	3.30942526
C	-4.36218855	-2.40776551	0.21626461
C	-5.11300725	-1.94049189	-0.87714748
C	-4.49485300	-1.22117594	-1.89783099
C	-3.11501826	-0.98325851	-1.86435383
C	-2.36338780	-1.45523974	-0.78624240
C	-2.98219487	-2.15403420	0.25333206
H	-6.18491986	-2.12092980	-0.91646885
C	-5.32275013	-0.75115845	5.60943708
C	-6.61523389	-1.13698088	5.23232181
C	-7.62028492	-1.25395031	6.19798438
C	-7.34490690	-0.99972039	7.54441281
C	-6.05493899	-0.61314298	7.92461715
C	-5.05104029	-0.48897302	6.96080273
H	-6.85222068	-1.32705520	4.18740390
H	-8.63208103	-1.50851914	5.89453032
H	-8.13292185	-1.09010028	8.29138534

H	-5.83267168	-0.40046386	8.97007936
C	-0.90080814	-0.35619829	5.82886316
C	-1.50374397	0.87830319	6.13032022
C	-1.63318548	1.27459392	7.46356569
C	-1.16780914	0.45183766	8.49317663
C	-0.55896707	-0.77214795	8.19210257
C	-0.42066944	-1.17793651	6.86406204
H	-1.89967990	1.51760905	5.34270112
H	-2.11064362	2.22664925	7.69212937
H	-1.27655893	0.76306371	9.53152916
H	-0.18834663	-1.41196009	8.99184370
C	-0.86396616	0.50338245	3.03658810
C	-1.67726999	0.48440806	1.89691272
C	-1.69440836	1.58201869	1.03640594
C	-0.91169089	2.70219700	1.32099310
C	-0.09097570	2.71906634	2.45675744
C	-0.05725564	1.62205931	3.31553734
H	-2.32029470	-0.36531606	1.70828127
H	-2.34273313	1.56255051	0.16235569
H	-0.94994208	3.57292358	0.66787407
H	0.51346655	3.59712994	2.67964541
C	0.65392177	-1.89718771	3.81661747
C	0.77188001	-3.18572002	4.36815088
C	1.97355506	-3.88033675	4.24452440
C	3.04494114	-3.31295714	3.54685734
C	2.91508237	-2.04772964	2.96586571
C	1.72381573	-1.33180400	3.10450441
H	-0.07925836	-3.65451285	4.85526077
H	2.06471033	-4.87811612	4.67136573
H	3.97795174	-3.86567811	3.44136771
H	3.74058549	-1.61561746	2.40188664

TS–ModeB–LA1–RA2–*R*

COSMO(toluene)-ZORA-M06-2X/TZ2P//COSMO(toluene)-ZORA-BLYP-D3(BJ)/DZP

E = -25759.27

G = -25179.24

$\boldsymbol{N}_{\text{imag}}$ = 1, 157*i* cm^{-1}

C	-4.72709814	1.25802609	1.14109975
C	-5.03666983	1.93496221	2.48526990
C	-5.20965296	-0.01594464	1.74255124
C	-5.40633946	0.58931505	3.01180192
O	-4.27183763	1.59882730	0.04694507

O	-5.02078336	3.08518769	2.93722564
N	-5.83204584	0.04172125	4.15489464
N	-5.45762023	-1.25901561	1.32590082
C	-5.34746096	-1.69599510	-0.07325064
C	-4.97117729	-3.18370778	-0.01581732
H	-4.53834949	-1.11556090	-0.53774387
C	-6.64814258	-1.44383984	-0.93876688
H	-5.66170711	-1.97207786	2.04499393
C	-6.24326200	0.75916933	5.37269931
H	-6.84070962	0.02226807	5.92337753
C	-7.20651601	1.93504579	5.01889584
H	-4.16314082	2.50837554	4.83600672
H	-5.86854462	-0.99386595	4.18423255
H	-6.68486798	2.89701578	5.01252382
N	-8.37878590	1.99235434	5.90955849
H	-7.60313847	1.77088208	4.00873605
P	-8.43507059	2.74672089	7.37397707
H	-7.01546559	3.45326089	9.89612108
H	-10.01326890	3.05455456	9.81615995
H	-9.20000174	5.49290324	6.78189000
H	-5.44552947	-1.49733390	-2.77815855
H	-7.19586704	-1.67951633	-3.03242150
H	-6.24767787	-3.04379080	-2.39734980
H	-7.25861465	0.44477053	-0.00490310
H	-7.74375024	0.26606753	-1.70271343
H	-6.04750862	0.64436803	-1.29016528
H	-7.72023360	-3.24379915	-0.29027597
H	-8.73956523	-2.00036607	-1.04147054
H	-8.15007073	-1.77968108	0.62196170
H	-4.84978165	-2.27865337	8.33778605
H	-6.22968717	-3.52826282	10.00170374
H	-8.40271513	-4.53195051	9.27318306
C	-6.36186446	-1.95110639	-2.37037215
C	-6.93269582	0.07251536	-0.98570260
C	-7.88397592	-2.16506268	-0.37020924
O	-5.61762139	-3.99070151	0.68136980
N	-3.91368048	-3.54997340	-0.76978811
C	-3.39959024	-4.93469816	-0.81229774
H	-3.39590128	-2.82737047	-1.26768923
C	-2.50776190	-5.21198024	0.40814366
C	-2.67248021	-5.15538877	-2.14203584
H	-4.28540792	-5.58457573	-0.75977915

H	-2.18006242	-6.26116993	0.40795431
H	-3.06228945	-5.01799755	1.33514367
H	-1.61677978	-4.56720905	0.38511857
H	-2.31645953	-6.19208660	-2.20688993
H	-1.79691446	-4.49224194	-2.22125225
H	-3.33988125	-4.96115440	-2.99294865
C	-9.96394327	-3.70158532	3.60281560
C	-11.22914924	-4.29241839	3.52988635
C	-11.54858957	-5.18908276	2.50293677
C	-10.59240739	-5.49942917	1.52917562
C	-9.32010491	-4.92727031	1.58840586
C	-9.00762909	-4.03410321	2.62204519
H	-11.97987042	-4.02048740	4.26846737
H	-12.54188237	-5.63414992	2.45950150
H	-10.83409309	-6.19141065	0.72287222
H	-8.56482208	-5.17374789	0.84434573
N	-7.74396024	-3.43481362	2.69102147
C	-10.53529953	-1.26324732	6.43026673
C	-9.58907181	-2.65980656	4.61877178
H	-9.33085351	-1.70560061	4.13901634
C	-10.44521048	-2.47698361	5.74460382
H	-11.00711112	-3.31659690	6.14311681
O	-9.92423867	-0.18013806	6.20389460
O	-11.45053369	-1.31689461	7.49705946
C	-11.67513262	-0.06530820	8.19107926
H	-12.49052476	-0.26714363	8.89347848
H	-10.78431364	0.25076445	8.74445078
H	-11.96108405	0.73021312	7.49492247
C	-7.13026388	-3.06758925	3.88216307
H	-7.07500721	-3.61745705	1.92828088
O	-5.90948528	-2.72338374	3.83058660
N	-7.93117481	-3.11458514	4.97476947
H	-5.60192144	-2.04453420	6.01556880
H	-8.95184068	1.11223223	5.89968257
C	-7.45151768	-3.13712997	6.29565802
C	-8.22136218	-3.84533664	7.24512949
C	-7.79281451	-3.96909402	8.56614939
C	-6.57529946	-3.41185997	8.97503337
C	-5.80505488	-2.71226792	8.04333590
C	-6.23772408	-2.55862679	6.72336673
H	-9.13129783	-4.33695728	6.92154769
C	-5.08387826	1.17465725	6.27120279

C	-5.02069260	0.68433714	7.58122843
C	-4.02392661	1.11615576	8.46081690
C	-3.05869111	2.02960615	8.02631333
C	-3.10162638	2.50597330	6.70988848
C	-4.11133621	2.09057799	5.83911923
H	-5.76979027	-0.01821857	7.92691309
H	-4.00995946	0.74305639	9.48409799
H	-2.27834640	2.36931850	8.70721750
H	-2.35236863	3.21727776	6.36207013
C	-7.47701488	4.27222585	7.27644825
C	-6.08494707	4.24372168	7.46984714
C	-5.34144396	5.40965317	7.29034866
C	-5.97983284	6.60117434	6.93134616
C	-7.36750508	6.63227879	6.75167620
C	-8.12122764	5.46968247	6.92043675
H	-5.58534291	3.32319326	7.74891879
H	-4.26181142	5.38096170	7.42527305
H	-5.39486296	7.50940319	6.79029753
H	-7.86391343	7.56201557	6.47762709
C	-7.86670511	1.78314357	8.80224236
C	-8.14491516	0.40445770	8.81262848
C	-7.77580116	-0.36396224	9.91488963
C	-7.11793004	0.23059383	10.99689917
C	-6.84332607	1.60192999	10.98684595
C	-7.22552502	2.38611036	9.89614004
H	-8.63508447	-0.06123829	7.95973804
H	-7.98502903	-1.43016954	9.91267870
H	-6.81709625	-0.37680411	11.85030252
H	-6.33035276	2.06467519	11.82921709
C	-10.18478475	3.11307622	7.65572191
C	-11.04311944	3.29578890	6.55974783
C	-12.38893173	3.59196384	6.78168714
C	-12.87699445	3.70365082	8.08772116
C	-12.02002126	3.51939258	9.17841173
C	-10.67294112	3.22264262	8.96749118
H	-10.65888917	3.18720560	5.54817562
H	-13.05930183	3.72278393	5.93325616
H	-13.93062066	3.92363886	8.25686886
H	-12.40361241	3.59497845	10.19499574

TS–ModeB–LA2–RA1–*R*

COSMO(toluene)-ZORA-M06-2X/TZ2P//COSMO(toluene)-ZORA-BLYP-D3(BJ)/DZP

E = -25763.31
G = -25186.50
$\boldsymbol{N}_{imag}$ = 1, 159*i* cm^{-1}

C	-4.86516574	2.77886326	1.97242848
C	-4.23235466	2.19576134	3.24087508
C	-5.40989005	1.42715689	1.68015786
C	-4.78831484	0.88025400	2.83310610
O	-4.85840677	3.85964489	1.36447055
O	-3.53068937	2.60315364	4.17234626
N	-4.67010519	-0.37244793	3.28582747
N	-6.15622346	0.91330149	0.70740248
C	-6.91559858	1.76310576	-0.23551363
C	-6.10462284	1.92153323	-1.56186613
H	-6.96529027	2.75983196	0.22382276
C	-8.38315744	1.26335695	-0.37471113
H	-6.33133966	-0.10109458	0.73922019
C	-4.25312057	-0.63012994	4.66545185
H	-3.64666638	0.22930205	4.96663193
C	-3.39628405	-1.91275455	4.75302917
H	-4.18533862	-0.19541260	7.33160755
H	-5.27754391	-1.06391644	2.81460312
H	-3.17739548	-2.10017630	5.81404889
N	-2.15093797	-1.88225933	3.96791653
H	-3.97915852	-2.76694033	4.38233410
P	-0.92860108	-0.80365501	4.17259033
H	0.26983236	1.92742524	4.26675808
H	1.53927625	-0.00459155	2.79635727
H	0.12961187	-1.95175483	6.63891317
H	-9.05337534	3.25907119	-0.98768012
H	-10.19267199	1.94794604	-1.37412594
H	-8.71320676	2.15573276	-2.34952419
H	-8.53940746	0.67636425	1.74017478
H	-10.09562796	1.03869522	0.95628563
H	-9.00315509	2.36737151	1.42154110
H	-7.95818184	-0.28566649	-1.85899094
H	-9.52414216	-0.46945004	-1.01685234
H	-8.02458283	-0.89807290	-0.18040914
H	-1.39794808	-0.89725670	-0.92614340
H	-2.79032791	-0.09389319	-2.83819671
H	-5.21009935	-0.69560502	-2.93223482
C	-9.12721741	2.21729350	-1.33629270
C	-9.04075123	1.34149639	1.02446609

C	-8.46679682	-0.19035096	-0.89445133
O	-6.38486175	1.32530191	-2.60917537
N	-5.05898993	2.77373143	-1.39471916
C	-3.94996262	3.02525114	-2.34084163
H	-4.96428609	3.22006247	-0.47259506
C	-3.17525449	4.24761660	-1.82430350
C	-4.42256217	3.23782792	-3.78690554
H	-3.28023546	2.14725151	-2.31182373
H	-2.26198712	4.39165146	-2.41726727
H	-2.89285348	4.12349815	-0.77046988
H	-3.79533679	5.15414234	-1.90352292
H	-3.55406230	3.48797147	-4.41356505
H	-5.13773447	4.07435201	-3.82985008
H	-4.91334752	2.34266668	-4.18041653
C	-5.29996900	-5.67367182	1.11734866
C	-5.17314648	-6.93878819	0.53514753
C	-6.25541510	-7.82537972	0.50010623
C	-7.48068970	-7.45123301	1.06377961
C	-7.63419064	-6.18414835	1.63212260
C	-6.55143150	-5.29691239	1.64428519
H	-4.20412494	-7.23822183	0.14175636
H	-6.13755199	-8.80984651	0.04921725
H	-8.32414127	-8.14065575	1.05221181
H	-8.59232566	-5.87694558	2.05145632
N	-6.68586767	-3.99153530	2.16623886
C	-1.70643164	-4.55211142	1.16862316
C	-4.15177814	-4.72557033	1.30587086
H	-3.99030770	-4.52881391	2.37425322
C	-2.92324770	-4.96269078	0.61615926
H	-2.92505525	-5.38408363	-0.38557796
O	-1.50645221	-3.93209004	2.25533029
O	-0.60002267	-4.86657183	0.36670596
C	0.67666198	-4.44568772	0.90663512
H	1.40739712	-4.64912159	0.11701555
H	0.67202729	-3.37943371	1.16060383
H	0.92615790	-5.01733276	1.80847949
C	-6.05654598	-2.89644235	1.56249755
H	-7.58826537	-3.74313814	2.57399125
O	-6.56196445	-1.74255957	1.73663027
N	-4.92296758	-3.18204827	0.91042465
H	-2.42736471	-2.27424115	0.86125433
H	-2.00284178	-2.65512674	3.27418232

C	-4.37117123	-2.31772650	-0.05538406
C	-5.14543036	-1.88097047	-1.14385366
C	-4.58478614	-1.06809596	-2.12642979
C	-3.23058326	-0.71588091	-2.06008431
C	-2.45159353	-1.16690183	-0.99243826
C	-3.01954986	-1.95467692	0.01330201
H	-6.18880059	-2.18118462	-1.20809149
C	-5.41978565	-0.73291518	5.64129195
C	-6.70549120	-1.10153738	5.22497710
C	-7.73763629	-1.22894325	6.16112661
C	-7.49475330	-0.99081881	7.51749869
C	-6.21195152	-0.61923394	7.93675390
C	-5.18089564	-0.49205700	7.00270455
H	-6.91405482	-1.27787326	4.17151347
H	-8.73603110	-1.51065901	5.82715965
H	-8.30128977	-1.08721975	8.24354795
H	-6.01652984	-0.42235818	8.99057307
C	-0.95377437	-0.22419488	5.89287169
C	-1.61329621	0.97042791	6.23183989
C	-1.72195110	1.33808757	7.57502466
C	-1.17880233	0.52633534	8.57498328
C	-0.51428446	-0.65819620	8.23536886
C	-0.39682964	-1.03591387	6.89719426
H	-2.06565141	1.59937090	5.46698183
H	-2.24180423	2.25926024	7.83520870
H	-1.27027017	0.81589954	9.62129145
H	-0.08429796	-1.28791531	9.01297259
C	-1.03649058	0.64505815	3.09501965
C	-1.81801406	0.53177334	1.93825238
C	-1.91315246	1.60595129	1.05334826
C	-1.23149656	2.79322484	1.32955703
C	-0.44153279	2.90406343	2.48117653
C	-0.33517451	1.83296672	3.36688684
H	-2.37208813	-0.37976541	1.75563131
H	-2.54076710	1.51270875	0.16958592
H	-1.31901392	3.64233645	0.65574659
H	0.08174697	3.83490984	2.69480545
C	0.61037582	-1.68157375	3.81090271
C	0.75846844	-3.01383421	4.23414930
C	1.96792210	-3.67276161	4.01980476
C	3.01980651	-3.01843543	3.37090906
C	2.86420273	-1.70034296	2.92989952

C	1.66194789	-1.02534382	3.15032027
H	-0.07537096	-3.53728722	4.69460780
H	2.08142794	-4.70709534	4.34072941
H	3.95847072	-3.54252999	3.19387619
H	3.67644141	-1.19729620	2.40683402

TS–ModeB–LA2–RA2–*R*

COSMO(toluene)-ZORA-M06-2X/TZ2P//COSMO(toluene)-ZORA-BLYP-D3(BJ)/DZP

E = -25755.00

G = -25176.00

N_{imag} = 1, 180*i* cm^{-1}

C	-5.34526207	2.94987146	1.87595971
C	-5.24532850	2.83667746	3.40772240
C	-6.25846612	1.78547229	1.89192973
C	-6.12381497	1.62740544	3.29670474
O	-4.83611586	3.65059147	0.98657715
O	-4.69428929	3.46113617	4.30976085
N	-6.65560982	0.68195215	4.08986119
N	-6.93643109	1.08894848	0.97418789
C	-6.74138216	1.28891804	-0.47531121
C	-5.44053023	0.52732785	-0.86704703
H	-6.55761408	2.36296708	-0.61683613
C	-8.01909912	0.92768314	-1.28292656
H	-7.40198119	0.23014275	1.29462434
C	-5.99847745	0.08091596	5.27172605
H	-5.95255302	-0.99238319	5.03759328
C	-6.90763774	0.17945375	6.51835085
H	-4.84059538	1.76712149	7.17153621
H	-7.28909861	0.03002694	3.58205969
H	-6.79468986	1.13291774	7.04707164
N	-6.70150773	-0.94177221	7.45972794
H	-7.95041746	0.12146656	6.17902853
P	-5.74836672	-0.97125925	8.79547969
H	-3.28250287	-0.55589487	10.42489163
H	-4.51008934	-2.63663870	10.86524005
H	-7.97341803	0.07832583	10.25415714
H	-7.31504767	2.11526048	-2.98728453
H	-8.60850863	0.95759060	-3.38092031
H	-6.94612229	0.37313602	-3.11267483
H	-9.34447144	1.85612743	0.19956111
H	-10.05435412	1.70042093	-1.42853828
H	-8.83423764	2.95842768	-1.10394439

H	-7.71323478	-1.23912701	-1.24166700
H	-9.37672575	-0.72848546	-1.64981192
H	-8.81982684	-0.64983210	0.02994606
H	-3.16457739	-3.38138723	4.21446007
H	-2.55453345	-2.81739222	1.86405703
H	-4.37207962	-2.15075750	0.25434588
C	-7.69678580	1.10294953	-2.78379208
C	-9.13013857	1.92322162	-0.87588093
C	-8.50420680	-0.51671612	-1.01363517
O	-5.42544750	-0.65551513	-1.24400594
N	-4.33088377	1.27975395	-0.66342517
C	-2.97561954	0.71276578	-0.57141582
H	-4.45590814	2.22551293	-0.27683358
C	-2.56177981	0.63034387	0.90862792
C	-1.99810643	1.54702898	-1.41073740
H	-3.04873282	-0.29992909	-0.99014982
H	-1.56695194	0.17217095	1.00738758
H	-3.28244131	0.02505419	1.46890958
H	-2.52977031	1.63314073	1.35924225
H	-0.98804626	1.11627818	-1.36106296
H	-1.94939792	2.57922811	-1.03197440
H	-2.31859035	1.57554909	-2.46109861
C	-9.22685594	-4.79646053	3.33391627
C	-9.37306418	-6.17165138	3.12249608
C	-10.36389309	-6.66366528	2.26574516
C	-11.22952650	-5.77509461	1.61654035
C	-11.08598002	-4.39713885	1.79619053
C	-10.07903627	-3.91463705	2.64023106
H	-8.73110222	-6.85934606	3.66812238
H	-10.47151256	-7.73755807	2.11959020
H	-12.01065299	-6.15253631	0.95746897
H	-11.73839655	-3.69805165	1.27326787
N	-9.86458269	-2.53048891	2.80427808
C	-6.83856531	-4.63871866	6.24326638
C	-8.29239810	-4.19089099	4.33300256
H	-8.84298579	-3.56722599	5.04816888
C	-7.31707367	-5.00492724	4.97794255
H	-6.87392949	-5.86004327	4.47589052
O	-7.12104779	-3.59687155	6.89961733
O	-5.92239953	-5.55633744	6.77767139
C	-5.37427744	-5.18789256	8.06826482
H	-4.73591647	-6.02748612	8.36192754

H	-4.77500285	-4.27224435	7.99656199
H	-6.16876900	-5.03642904	8.80461624
C	-8.58187117	-1.98965056	2.90021979
H	-10.53169730	-1.88959390	2.37212180
O	-8.41474040	-0.78644415	2.53699344
N	-7.64105228	-2.81492245	3.39934799
H	-5.51859845	-3.23657498	4.95810068
H	-6.99425574	-1.89303182	7.13595407
C	-6.29517293	-2.74615718	3.00534782
C	-5.93932884	-2.43478229	1.67636449
C	-4.60491505	-2.43522329	1.27821679
C	-3.59669122	-2.80094906	2.18131215
C	-3.94005773	-3.11572304	3.49745125
C	-5.27139184	-3.05751052	3.92183234
H	-6.71087582	-2.19024439	0.95186237
C	-4.56346282	0.54535623	5.42031324
C	-3.63613394	0.11854300	4.45942525
C	-2.29253922	0.46807180	4.56567386
C	-1.85441180	1.24223408	5.64581283
C	-2.77854576	1.69692367	6.58806743
C	-4.12886298	1.36660802	6.45923113
H	-3.97082178	-0.50021415	3.63269213
H	-1.58449356	0.12867328	3.81097591
H	-0.80015738	1.49872480	5.74481631
H	-2.44940866	2.32476353	7.41544101
C	-5.94789888	0.60230270	9.67785851
C	-4.94278490	1.58060653	9.73708780
C	-5.18988334	2.79018705	10.38998319
C	-6.43218004	3.02643298	10.98536452
C	-7.43382547	2.04962875	10.93366928
C	-7.19540164	0.83839911	10.28547392
H	-3.97869565	1.40846656	9.27176291
H	-4.41044581	3.54986969	10.42764526
H	-6.62165671	3.97249122	11.49097827
H	-8.40069973	2.23062659	11.40091472
C	-3.98610150	-1.27213979	8.49767422
C	-3.61506597	-1.81895893	7.25875782
C	-2.26893656	-2.02997402	6.97057008
C	-1.29124073	-1.71369284	7.91812891
C	-1.65945937	-1.19739729	9.16447597
C	-3.00530874	-0.97372264	9.45936384
H	-4.37300966	-2.05569809	6.52042189

H	-1.98233057	-2.42168052	5.99788848
H	-0.23845850	-1.86491234	7.68273644
H	-0.89790989	-0.95582259	9.90479657
C	-6.38914323	-2.31645624	9.82708177
C	-7.72385169	-2.72840631	9.69030613
C	-8.22055547	-3.73885721	10.51444254
C	-7.39446491	-4.33783631	11.47019607
C	-6.06206231	-3.93123377	11.60086653
C	-5.55467007	-2.92396916	10.77919304
H	-8.36223893	-2.28246375	8.93414465
H	-9.25180427	-4.06864144	10.39717198
H	-7.78502429	-5.13195525	12.10561657
H	-5.41238091	-4.40714923	12.33404292

TS–ModeA–LA1–RA1–*S*

COSMO(toluene)-ZORA-M06-2X/TZ2P//COSMO(toluene)-ZORA-BLYP-D3(BJ)/DZP

E = -25753.73

G = -25175.52

$\boldsymbol{N}_{\text{imag}}$ = 1, 245*i* cm^{-1}

C	0.91830663	0.86669541	-1.19143806
C	0.20032329	-0.48067464	-1.34606921
C	2.17856304	0.10118397	-0.95648277
C	1.51613929	-1.13985840	-1.13051694
O	0.63645341	2.06591258	-1.27573306
O	-0.94740527	-0.88276816	-1.56928672
N	1.90119899	-2.42610018	-1.20541986
N	3.43672101	0.46928853	-0.73927371
C	3.84735455	1.86776258	-0.89684729
C	4.63592176	2.28355325	0.35526107
H	2.92085030	2.45216417	-0.96202171
C	4.63976982	2.10017647	-2.24377112
H	4.12122825	-0.25923769	-0.48951225
C	0.95626571	-3.39217422	-1.79506870
H	-0.04041259	-2.97354249	-1.57480378
C	0.95318458	-4.74204706	-1.07357161
H	1.80915787	-1.45658465	-3.56005891
H	2.90332525	-2.63418108	-1.06634680
H	0.87317541	-4.53495339	0.00108639
N	2.14212749	-5.59934815	-1.31092251
H	0.02997798	-5.26499503	-1.35851905
P	2.45344500	-6.87167843	-0.30875927
H	5.28152015	-7.48199610	-0.73169274

H	-0.05793260	-7.55724919	-1.60645513
H	4.07010451	-8.40740467	1.60672979
H	5.30880346	4.12704886	-1.71602404
H	3.72760172	4.07446993	-2.54291957
H	5.22799918	3.80617232	-3.46246992
H	6.66557335	1.96130707	-1.40362749
H	6.55439484	1.65163642	-3.15623620
H	6.02315369	0.41501678	-2.00065002
H	4.31380251	1.67517941	-4.35424064
H	2.80213936	1.79517022	-3.41316908
H	3.83672776	0.34417520	-3.28978200
H	6.83069427	-5.41616507	1.09202157
H	8.46955186	-7.00820199	0.07963868
H	8.40112279	-7.44454739	-2.37605647
C	4.72988345	3.62077187	-2.49975332
C	3.84180430	1.43629352	-3.39070097
C	6.05582954	1.49458112	-2.18802791
O	5.43893628	1.51776039	0.90626509
N	4.40876595	3.55588177	0.77596752
C	5.07373937	4.13452294	1.95751125
H	3.65749674	4.08872570	0.34037855
C	4.35941947	3.70449850	3.24920341
C	5.14412427	5.65693971	1.79873506
H	6.09035490	3.71427796	1.96093797
H	4.89464298	4.08978447	4.12907592
H	4.32307590	2.60917593	3.31129561
H	3.33102843	4.09546338	3.26648640
H	5.66365307	6.10266695	2.65764601
H	4.13285878	6.09081927	1.75360760
H	5.68436619	5.93155525	0.88217579
C	5.34151887	-2.34237460	-4.44369235
C	6.13995688	-1.29553233	-4.91880722
C	6.14790974	-0.94853218	-6.27337579
C	5.34130425	-1.65604407	-7.17233356
C	4.56042628	-2.72304363	-6.72423125
C	4.56983032	-3.08094534	-5.36821026
H	6.71826016	-0.71861524	-4.20182665
H	6.76282950	-0.11921033	-6.62068561
H	5.32435744	-1.38356821	-8.22747633
H	3.94513003	-3.28934571	-7.42252503
N	3.80074353	-4.16186627	-4.92382648
C	5.75231704	-1.98548320	-0.69706928

C	5.15753342	-2.59843572	-2.98626069
H	4.12247278	-2.42158678	-2.68669888
C	6.11347322	-2.16311207	-2.04071634
H	7.16863152	-2.12952836	-2.30195395
O	4.58720439	-2.07328258	-0.19725793
O	6.82964968	-1.68352147	0.12441439
C	6.51054188	-1.52008821	1.53212742
H	7.47397353	-1.34597227	2.02233740
H	6.04617632	-2.43123924	1.92409116
H	5.84924357	-0.66111946	1.67900835
C	4.09126705	-4.96685657	-3.82833119
H	3.06566980	-4.51798429	-5.53194852
O	3.46086782	-6.05869768	-3.68229710
N	4.98455928	-4.44458968	-2.97540560
H	5.16102351	-4.27995757	-0.35087741
H	2.55506116	-5.71093816	-2.27772990
C	5.83736437	-5.19496848	-2.16767284
C	6.75841051	-6.09986772	-2.73178488
C	7.69348622	-6.74703118	-1.92674258
C	7.73409776	-6.50202882	-0.54524258
C	6.82321894	-5.60385332	0.01964975
C	5.88461393	-4.95503429	-0.78522598
H	6.72761188	-6.28164224	-3.80530451
C	1.02098766	-3.45754807	-3.32573442
C	0.54312103	-4.57752996	-4.02028578
C	0.45434654	-4.57138445	-5.41585900
C	0.83593167	-3.43783864	-6.14060532
C	1.32678897	-2.32059048	-5.45831238
C	1.42178941	-2.33499017	-4.06661184
H	0.23947622	-5.47267588	-3.48426554
H	0.08677189	-5.45623750	-5.93505320
H	0.76017407	-3.42979021	-7.22771576
H	1.65097787	-1.44050663	-6.01114377
C	0.87557521	-7.49038766	0.35197102
C	0.68838093	-7.68795644	1.72895809
C	-0.54474537	-8.14293772	2.20378556
C	-1.59227230	-8.39498859	1.31355603
C	-1.41216495	-8.19020952	-0.05955995
C	-0.18555814	-7.73553367	-0.54101794
H	1.49179948	-7.47454914	2.42883736
H	-0.68762880	-8.29054623	3.27324187
H	-2.55388509	-8.74258843	1.68900034

H	-2.23107764	-8.37230600	-0.75383222
C	3.29394906	-8.18532327	-1.22290998
C	2.54073278	-9.14409434	-1.92490287
C	3.19239905	-10.10584583	-2.69444460
C	4.59065189	-10.12179938	-2.76061609
C	5.33662176	-9.17981996	-2.04896543
C	4.69520753	-8.20854661	-1.27988746
H	1.45727461	-9.15570946	-1.86592091
H	2.60789377	-10.84629261	-3.23861498
H	5.09699465	-10.87406328	-3.36456509
H	6.42241227	-9.18884118	-2.09573611
C	3.46214586	-6.38474205	1.11061883
C	3.53707578	-5.02834396	1.46126780
C	4.26447553	-4.64671216	2.58858572
C	4.92242543	-5.61044693	3.35837552
C	4.85497323	-6.96262510	3.00318821
C	4.12105699	-7.35562617	1.88337857
H	3.06889444	-4.26700876	0.84443228
H	4.32796027	-3.59252046	2.84877516
H	5.49966879	-5.30701802	4.23137343
H	5.37862637	-7.71155305	3.59576233

TS–ModeA–LA1–RA2–*S*

COSMO(toluene)-ZORA-M06-2X/TZ2P//COSMO(toluene)-ZORA-BLYP-D3(BJ)/DZP

E = -25763.58

G = -25185.65

$\boldsymbol{N}_{\text{imag}}$ = 1, 201*i* cm^{-1}

C	0.87724262	1.03533219	-1.89128395
C	0.26437012	-0.34176357	-2.13110841
C	2.09873650	0.34966786	-1.39329557
C	1.53475289	-0.94003297	-1.61728522
O	0.53818825	2.21355836	-2.04891234
O	-0.81131921	-0.77422654	-2.57333982
N	2.06867658	-2.15288452	-1.41144698
N	3.27660189	0.76310199	-0.94578057
C	3.64077585	2.18448095	-0.93822615
C	4.28177738	2.47927866	0.43033207
H	2.70459479	2.74750984	-1.04663001
C	4.56449532	2.57056077	-2.15660135
H	3.97963130	0.05838955	-0.66398821
C	1.54831338	-3.41672744	-1.95461165
H	2.42651048	-4.05772571	-2.10013498

C	0.90977703	-3.14813795	-3.32782652
H	-0.59972699	-2.39761278	-0.55411415
H	3.06463361	-2.15510160	-1.09620731
H	-0.06036253	-2.66788243	-3.16566820
N	0.72144840	-4.41179342	-4.08869580
H	1.54351514	-2.44951414	-3.89017986
P	-0.44848230	-4.27986590	-5.27463003
H	0.43614092	-4.00577037	-8.07968541
H	-1.40385356	-5.31739578	-2.74969952
H	1.69914102	-5.79740211	-6.59439498
H	5.28837287	4.45130187	-1.27273943
H	3.77789265	4.61999779	-2.20802172
H	5.32961534	4.40050847	-3.05037172
H	6.46314587	2.15913658	-1.13586844
H	6.55465387	2.17854681	-2.91751024
H	5.84481382	0.78922382	-2.08246005
H	4.47556501	2.40382520	-4.32696360
H	2.87714095	2.64432818	-3.55730521
H	3.69033889	1.06146893	-3.49807623
H	5.56675601	-6.01534660	-0.70179742
H	6.46790525	-7.77858300	-2.22237956
H	6.38272238	-7.43085041	-4.69346023
C	4.74960526	4.10396396	-2.16441251
C	3.85147660	2.14422083	-3.46168415
C	5.93807916	1.88056387	-2.05855027
O	5.06048703	1.68031102	0.97054563
N	3.94677315	3.67983739	0.97042143
C	4.44736983	4.13149115	2.28176080
H	3.22695989	4.23817869	0.51411588
C	3.59462902	3.54562237	3.41882563
C	4.49172275	5.66302665	2.30394304
H	5.46773811	3.72932482	2.36287852
H	4.00779150	3.83559204	4.39561676
H	3.58199818	2.44988261	3.35388377
H	2.56092091	3.91700679	3.35287207
H	4.89205534	6.01480849	3.26438576
H	3.48083038	6.08374982	2.18546288
H	5.12895290	6.04908232	1.49646564
C	5.22052244	-0.92869828	-4.76900005
C	6.26677437	-0.00608129	-4.87672313
C	6.33463570	0.88757804	-5.94867379
C	5.34035222	0.86621859	-6.93266897

C	4.31635512	-0.08093241	-6.87300772
C	4.27168932	-0.99048757	-5.80841423
H	6.99795404	0.04634485	-4.07608872
H	7.14403067	1.61463859	-6.00158381
H	5.36738468	1.57676232	-7.75854937
H	3.55743070	-0.12762951	-7.65287980
N	3.28652797	-1.99366987	-5.76403616
C	5.77422599	-1.78080375	-1.19172959
C	4.97357810	-1.72248123	-3.52266552
H	4.00930420	-1.44771692	-3.08445035
C	6.01930111	-1.84116165	-2.56665775
H	7.05019454	-1.92984194	-2.90028729
O	4.64799048	-1.71244739	-0.60080058
O	6.93094848	-1.76443882	-0.42284640
C	6.71723781	-1.52359442	0.99777419
H	7.71894874	-1.53368788	1.43853926
H	6.09844412	-2.31469624	1.43779544
H	6.23552263	-0.55133619	1.15129889
C	3.55381440	-3.27725164	-5.24382874
H	2.67264065	-2.05080219	-6.57744097
O	2.92974038	-4.25446029	-5.74806170
N	4.40135067	-3.30575536	-4.20501748
H	4.58006357	-3.95579061	-1.63420159
H	1.63482827	-4.68327571	-4.51720474
C	4.91995026	-4.50953633	-3.69702382
C	5.44538603	-5.49893070	-4.54928832
C	5.98718083	-6.66970171	-4.02059009
C	6.03622349	-6.86613067	-2.63265894
C	5.53152325	-5.87894045	-1.78253110
C	4.97453033	-4.70818787	-2.30665848
H	5.41398926	-5.34215732	-5.62506699
C	0.57674980	-4.14514387	-1.04211391
C	0.68565686	-5.53374518	-0.89711715
C	-0.26044106	-6.24592812	-0.15215658
C	-1.31928380	-5.56830613	0.46134291
C	-1.42553762	-4.17949286	0.32625131
C	-0.48697692	-3.47121944	-0.42728763
H	1.50840481	-6.06082544	-1.38038999
H	-0.16853731	-7.32691448	-0.04955914
H	-2.05626263	-6.11995382	1.04478879
H	-2.25197346	-3.64627457	0.79565601
C	-2.01953343	-4.07916291	-4.41884424

C	-3.04953452	-3.31248885	-4.98800530
C	-4.27063587	-3.20227664	-4.32167725
C	-4.46112849	-3.85153766	-3.09693160
C	-3.43325188	-4.61530690	-2.53385748
C	-2.20961827	-4.73667579	-3.19045133
H	-2.89644151	-2.80124280	-5.93593477
H	-5.06998156	-2.60324997	-4.75504536
H	-5.41348233	-3.75629351	-2.57649368
H	-3.57653665	-5.11010145	-1.57597170
C	-0.18479525	-2.84473341	-6.35606971
C	-0.32549992	-1.55176036	-5.81178668
C	0.06904259	-0.44221652	-6.56033730
C	0.58615108	-0.60948088	-7.84899226
C	0.70469123	-1.89115641	-8.39841230
C	0.32519390	-3.01151241	-7.65475048
H	-0.72231293	-1.39808931	-4.81036173
H	-0.02371943	0.55306594	-6.12813566
H	0.89474071	0.26057118	-8.42743732
H	1.10088370	-2.02215341	-9.40460009
C	-0.44117821	-5.81555164	-6.22315833
C	-1.65415167	-6.49628430	-6.42544546
C	-1.65970353	-7.69588478	-7.14030056
C	-0.46418548	-8.21789341	-7.64397819
C	0.74238759	-7.53932658	-7.43867309
C	0.76412499	-6.33556226	-6.73253271
H	-2.58224625	-6.09909153	-6.02105430
H	-2.59879250	-8.22469604	-7.29701632
H	-0.47188735	-9.15737646	-8.19547066
H	1.67305639	-7.94586782	-7.83240174

TS–ModeA–LA2–RA1–*S*

COSMO(toluene)-ZORA-M06-2X/TZ2P//COSMO(toluene)-ZORA-BLYP-D3(BJ)/DZP

E = -25757.84

G = -25178.21

$\boldsymbol{N}_{\text{imag}}$ = 1, 286*i* cm^{-1}

C	1.06033987	0.19822919	-2.76828955
C	0.96446695	-1.15037975	-3.48869373
C	2.24495609	-0.32826193	-2.03960365
C	2.08387927	-1.61668854	-2.61699699
O	0.39803209	1.24701581	-2.70898271
O	0.28652069	-1.65186371	-4.38726309
N	2.64266095	-2.80465722	-2.38882219

N	3.12270857	0.21144375	-1.19581763
C	3.00519922	1.63729097	-0.82616370
C	2.19348199	1.69710168	0.49077625
H	2.38535512	2.09372332	-1.60654307
C	4.36201015	2.39508587	-0.86472645
H	3.79177704	-0.42380150	-0.72043608
C	2.28028779	-3.94938887	-3.25204606
H	1.28559319	-3.70712845	-3.63927120
C	2.19973765	-5.27712811	-2.45550963
H	4.80471422	-5.11811288	-3.42554136
H	3.45889703	-2.83955241	-1.75121617
H	1.46085644	-5.92566670	-2.94736081
N	1.94973087	-5.12219784	-0.99694478
H	3.15211332	-5.80380327	-2.50667961
P	0.49148002	-4.73042332	-0.31510804
H	-0.89310380	-5.65177488	-2.69683701
H	-0.57904106	-4.76136730	2.43279359
H	1.32254725	-7.50866262	0.24050336
H	3.75124398	3.91697420	0.59122717
H	3.30920766	4.30819680	-1.09405334
H	5.00015579	4.45660405	-0.55807964
H	6.36299647	2.36772772	-0.03198930
H	5.66875019	0.74954120	-0.20714507
H	5.10284792	1.81484088	1.10450368
H	5.81604597	2.89986771	-2.41084220
H	4.13856001	2.82075543	-3.00847159
H	5.03174265	1.31988427	-2.65767081
H	2.89740198	-3.48812278	3.31398525
H	3.59235170	-5.06910649	5.10423297
H	5.06140245	-7.00728650	4.53510701
C	4.08353843	3.85857468	-0.45390701
C	4.86549930	2.35415967	-2.32654321
C	5.43282612	1.78749713	0.06184761
O	2.70689816	1.62115980	1.62107005
N	0.85705631	1.73980674	0.26045064
C	-0.15888565	1.67228965	1.32270123
H	0.53677191	1.75731458	-0.71733467
C	-1.21243935	0.61718455	0.95825498
C	-0.77432787	3.06064087	1.56233736
H	0.38095579	1.36141455	2.22722935
H	-1.99059002	0.56733499	1.73261644
H	-0.74875739	-0.37103488	0.87450217

H	-1.69520392	0.86531113	0.00124069
H	-1.50138215	3.02133990	2.38611622
H	-1.29275454	3.41333391	0.65822936
H	0.01053133	3.78451134	1.81971648
C	6.97964783	-5.30847436	-1.66547088
C	8.25907700	-4.85250468	-1.99962041
C	9.07682339	-5.57347716	-2.87643110
C	8.60739342	-6.76604632	-3.43725426
C	7.34134659	-7.25023024	-3.10047273
C	6.53269656	-6.53393818	-2.20875730
H	8.59095778	-3.89787876	-1.59528631
H	10.06626945	-5.19717935	-3.13250046
H	9.22901640	-7.32747836	-4.13411533
H	6.97653640	-8.18535982	-3.52557564
N	5.24233956	-6.98895749	-1.88987350
C	5.58000798	-2.34479303	0.23340103
C	6.02035935	-4.47565313	-0.88392526
H	5.12924176	-4.23055777	-1.46700867
C	6.43587612	-3.41855709	-0.04773923
H	7.34437694	-3.51460919	0.54234707
O	4.45822199	-2.08589598	-0.30570220
O	6.08345919	-1.49544249	1.21946998
C	5.08374868	-0.90257794	2.09215705
H	5.63412006	-0.22313181	2.74976705
H	4.60219451	-1.69330257	2.68223578
H	4.33124322	-0.33871093	1.54195545
C	4.61055608	-6.75867467	-0.65242468
H	4.89162060	-7.81240655	-2.37962578
O	3.52240679	-7.36141659	-0.41111552
N	5.21368166	-5.84335389	0.10985310
H	3.65188838	-3.83991205	0.97517958
H	2.46877073	-5.86961567	-0.48248216
C	4.76720679	-5.61909900	1.42722671
C	5.17467500	-6.50226320	2.44338604
C	4.74835170	-6.31054532	3.75740997
C	3.92405936	-5.22215714	4.07751814
C	3.53642180	-4.33274357	3.07285953
C	3.95703874	-4.52528814	1.75424317
H	5.81871708	-7.34022533	2.18017511
C	3.23415098	-4.03226746	-4.44246815
C	2.87377328	-3.40477826	-5.64431803
C	3.76598325	-3.37774778	-6.72124480

C	5.03066505	-3.96686318	-6.60589741
C	5.39782657	-4.58934218	-5.40771988
C	4.50095979	-4.62264296	-4.33743508
H	1.90122535	-2.91937622	-5.72019273
H	3.47479993	-2.88848649	-7.65057721
H	5.72659375	-3.93782382	-7.44389762
H	6.38022545	-5.04708713	-5.29538243
C	0.71172908	-3.63053526	1.10634693
C	1.49022873	-2.46859077	0.95394722
C	1.52988684	-1.52247488	1.97566523
C	0.80492022	-1.73407982	3.15323994
C	0.05591630	-2.90218272	3.31958584
C	0.00363184	-3.85386366	2.29767592
H	2.07120676	-2.31538528	0.05341584
H	2.11777904	-0.61632205	1.85807715
H	0.83572299	-0.98858545	3.94758351
H	-0.48853628	-3.07909941	4.24632488
C	-0.58306696	-3.87318870	-1.48831101
C	-0.94740105	-2.53109595	-1.28692298
C	-1.84889794	-1.91877160	-2.15605414
C	-2.38276668	-2.63507162	-3.22837202
C	-2.02838528	-3.97402898	-3.42778973
C	-1.13877805	-4.60073850	-2.55740778
H	-0.53393309	-1.96852767	-0.45896186
H	-2.10828847	-0.87202082	-2.01110731
H	-3.07005396	-2.14746308	-3.91782296
H	-2.44730331	-4.53235228	-4.26359762
C	-0.41958363	-6.20093932	0.23389824
C	-1.80393454	-6.10766669	0.46607896
C	-2.50029698	-7.21793142	0.94346961
C	-1.82184564	-8.41858540	1.18596061
C	-0.44912349	-8.51403887	0.93882018
C	0.26123561	-7.40865918	0.46048774
H	-2.33175721	-5.17631067	0.26778952
H	-3.57250409	-7.14806846	1.12171654
H	-2.36812602	-9.28430009	1.55875861
H	0.07511184	-9.45307835	1.11134543

TS–ModeA–LA2–RA2–*S*

COSMO(toluene)-ZORA-M06-2X/TZ2P//COSMO(toluene)-ZORA-BLYP-D3(BJ)/DZP

E = -25764.36

G = -25186.88

N_{imag} = 1, 199*i* cm^{-1}

C	0.92748103	1.24572494	-2.08221080
C	0.28032966	-0.11091810	-2.31340263
C	2.12768689	0.54308476	-1.56775454
C	1.53062704	-0.73816719	-1.77798029
O	0.59183704	2.43401547	-2.21379022
O	-0.80928468	-0.51547951	-2.74282008
N	2.01583732	-1.95485136	-1.51172393
N	3.31865715	0.92145930	-1.11420606
C	3.63320661	2.34697792	-0.86861831
C	3.02074057	2.70050905	0.51342249
H	3.08912117	2.91425614	-1.63551064
C	5.14084192	2.66278062	-1.06313064
H	3.93813671	0.17723763	-0.74513371
C	1.46087070	-3.22522190	-2.00247867
H	2.32143242	-3.89291860	-2.13078071
C	0.82292930	-2.99256886	-3.38232264
H	-0.62777409	-2.07553302	-0.61787076
H	3.00018078	-1.97120088	-1.15524738
H	-0.12802844	-2.46995340	-3.23755302
N	0.58206108	-4.28054337	-4.08100291
H	1.47917705	-2.34400072	-3.97830370
P	-0.55933328	-4.15821650	-5.29234036
H	0.42245275	-4.06254081	-8.07272170
H	-1.57999405	-5.01505203	-2.73193072
H	1.57519956	-5.83616655	-6.41243195
H	5.12653414	4.38851206	0.29081427
H	4.67848152	4.78352765	-1.39182586
H	6.37785713	4.45435605	-0.97494948
H	5.83613645	2.01492325	0.91158109
H	7.11192562	2.08276835	-0.35807456
H	5.94937115	0.74704573	-0.34586600
H	6.54781062	2.60042219	-2.73040455
H	4.87665704	2.98074870	-3.22241237
H	5.32418075	1.31578209	-2.78135124
H	5.47435456	-5.76943808	-0.30596894
H	6.63389702	-7.50267583	-1.67750757
H	6.71551646	-7.25481737	-4.16038119
C	5.33824169	4.16560528	-0.76364868
C	5.48933508	2.37180287	-2.54015471
C	6.05838117	1.82325023	-0.14807809
O	3.59871201	2.48924603	1.59025865

N	1.75314286	3.18067362	0.40577948
C	0.81900446	3.17587263	1.54661099
H	1.32335939	3.17203951	-0.52917086
C	0.19519963	1.77508405	1.69319150
C	-0.23475292	4.26995141	1.34414106
H	1.42289077	3.39633688	2.43785158
H	-0.47241797	1.73558254	2.56615144
H	0.98321010	1.02053385	1.82046282
H	-0.38859084	1.52462033	0.79492470
H	-0.92927745	4.29075198	2.19519690
H	-0.81896081	4.07914684	0.43089136
H	0.24153512	5.25587461	1.25356770
C	5.18307189	-1.00435764	-4.82360344
C	6.22133982	-0.08188708	-4.99701688
C	6.35384781	0.64437215	-6.18415986
C	5.43401478	0.45188321	-7.22091062
C	4.41105813	-0.48861223	-7.08300786
C	4.29555579	-1.22415161	-5.89603938
H	6.90238899	0.09391581	-4.16869700
H	7.15900255	1.36975292	-6.29107458
H	5.51803727	1.02380945	-8.14469571
H	3.70916859	-0.66634825	-7.89593153
N	3.30523662	-2.21827844	-5.76638747
C	5.68408214	-1.68119971	-1.15612339
C	4.90805242	-1.65841784	-3.50291162
H	3.94311815	-1.32935542	-3.10816322
C	5.94445168	-1.70470334	-2.52537362
H	6.97319900	-1.85605758	-2.84394877
O	4.55554189	-1.57019932	-0.56692430
O	6.82999788	-1.77057592	-0.37149004
C	6.59781044	-1.81542747	1.06139467
H	7.59318863	-1.90308499	1.50706846
H	5.98138023	-2.68286632	1.32752253
H	6.10390932	-0.90191853	1.41144724
C	3.55611472	-3.41918610	-5.07678220
H	2.72117353	-2.37672301	-6.58847154
O	2.96239460	-4.46812439	-5.46095109
N	4.36738916	-3.30522658	-4.01399791
H	4.39973004	-3.83671428	-1.39706127
H	1.48863776	-4.63762148	-4.46244388
C	4.93533661	-4.44069708	-3.40290298
C	5.60818314	-5.40884578	-4.16979433

C	6.20430814	-6.50782509	-3.55313650
C	6.15996371	-6.64738477	-2.15825010
C	5.51023793	-5.67730458	-1.39122118
C	4.89941317	-4.58029810	-2.00683031
H	5.64924753	-5.29181152	-5.25078822
C	0.47167846	-3.88504585	-1.05752696
C	0.52495666	-5.27145407	-0.86939612
C	-0.44500276	-5.91959106	-0.09713703
C	-1.47179931	-5.17997512	0.49893421
C	-1.52281439	-3.79304041	0.31975158
C	-0.55929087	-3.14868089	-0.45895232
H	1.32284602	-5.84691551	-1.33853650
H	-0.39648164	-6.99959878	0.04028998
H	-2.22700436	-5.68194801	1.10342170
H	-2.32281382	-3.21162583	0.77730740
C	-2.13263191	-3.84979731	-4.47449832
C	-3.12089894	-3.06529705	-5.09107827
C	-4.33471297	-2.85333388	-4.43566268
C	-4.55765668	-3.41736435	-3.17467087
C	-3.57128632	-4.19971631	-2.56451547
C	-2.35641041	-4.42392363	-3.21010026
H	-2.93997756	-2.61634793	-6.06538338
H	-5.10157425	-2.23902888	-4.90483996
H	-5.50246147	-3.23952772	-2.66210642
H	-3.73906955	-4.62707174	-1.57843607
C	-0.20247151	-2.78897742	-6.43083007
C	-0.29888810	-1.46468569	-5.95640167
C	0.18111589	-0.41516913	-6.74036371
C	0.74187539	-0.67161786	-7.99569031
C	0.81727720	-1.98308752	-8.47728968
C	0.35030197	-3.04464805	-7.69811698
H	-0.72566257	-1.24321685	-4.98041874
H	0.12199798	0.60408489	-6.36152049
H	1.11958474	0.15236773	-8.59955712
H	1.24453431	-2.18394392	-9.45898596
C	-0.58437750	-5.72220527	-6.18805367
C	-1.81983072	-6.33334370	-6.46126345
C	-1.84983491	-7.53608033	-7.17028700
C	-0.65735524	-8.12644961	-7.60056388
C	0.57152811	-7.51668812	-7.32362735
C	0.61891409	-6.31255209	-6.61939604
H	-2.74612848	-5.88032632	-6.11496813

H	-2.80571948	-8.01355391	-7.38063549
H	-0.68507644	-9.06636549	-8.15065550
H	1.49976940	-7.97925049	-7.65623172

TS–ModeB–LA1–RA1–*S*

COSMO(toluene)-ZORA-M06-2X/TZ2P//COSMO(toluene)-ZORA-BLYP-D3(BJ)/DZP

E = -25763.33

G = -25185.03

$\boldsymbol{N}_{\mathbf{imag}}$ = 1, 166*i* cm^{-1}

C	3.22167447	-0.25309816	1.88681090
C	1.97746800	-0.56721679	1.02921899
C	3.75185865	0.51049916	0.72026869
C	2.61627638	0.20171114	-0.06720085
O	3.65454119	-0.54997394	3.00460057
O	0.94121674	-1.23052537	1.13715647
N	2.28771039	0.41741876	-1.35403491
N	4.86244942	1.20913902	0.48974636
C	6.02458005	1.04949681	1.37469113
C	6.66165818	-0.32635658	1.08225467
H	5.65017938	1.04273361	2.40721758
C	7.02443048	2.24136990	1.26034864
H	4.99893884	1.54007396	-0.49428234
C	0.87133986	0.31458216	-1.74839896
H	0.49192083	-0.61988248	-1.31425578
C	0.68271922	0.27497949	-3.27414681
H	-1.56148614	0.09597497	-0.58162613
H	2.90842578	1.04873057	-1.87285714
H	-0.40424967	0.23940522	-3.43626988
N	1.36729188	-0.81049775	-4.00280350
H	1.03843793	1.20782004	-3.72908724
P	1.28255069	-2.41666133	-3.66488297
H	-0.25249784	-4.67806267	-4.65618807
H	0.48959962	-4.24231476	-1.42569470
H	1.72098267	-2.36355670	-0.74194663
H	8.28330140	3.27003047	-0.17272697
H	6.78803086	2.64535397	-0.89481207
H	8.11139341	1.52470160	-0.50576441
H	6.89669858	4.40683885	1.48535197
H	6.05542930	3.50309761	2.77151702
H	5.32795731	3.63293138	1.15074941
H	8.74261136	2.89723873	2.43656634
H	8.89856623	1.23651103	1.81736790

H	7.82803594	1.57633081	3.19993584
H	1.51873637	4.28183449	-4.56359205
H	2.20894953	4.94218363	-6.87221641
H	4.24005794	3.90713767	-7.88818758
C	8.19270561	1.96647320	2.23827403
C	6.27629875	3.52394640	1.69355969
C	7.58173101	2.42273116	-0.16964771
O	7.13006366	-0.61464212	-0.03754004
N	6.63765373	-1.18023942	2.12818239
C	7.11242136	-2.57408716	2.05750091
H	6.06652304	-0.91497904	2.93359062
C	6.02614282	-3.51847200	2.58640372
C	8.43715613	-2.72130283	2.82329371
H	7.29663976	-2.76138552	0.99116238
H	6.33651473	-4.56438916	2.45817542
H	5.07934797	-3.36301781	2.05955168
H	5.84819962	-3.34306784	3.65838007
H	8.83176610	-3.74106684	2.70953709
H	8.28619513	-2.52544270	3.89519751
H	9.18202338	-2.01157757	2.43915295
C	5.86622383	-1.18210618	-4.76282677
C	6.43921354	-2.13925294	-5.60615939
C	7.51483186	-2.92784284	-5.18079403
C	8.03064222	-2.75439817	-3.89149012
C	7.47399844	-1.80594515	-3.03273206
C	6.39061023	-1.02625599	-3.46447674
H	6.01499689	-2.28028464	-6.59806375
H	7.94449624	-3.67081359	-5.85178629
H	8.86626893	-3.36448210	-3.54809271
H	7.85268661	-1.67271607	-2.02202786
N	5.80198185	-0.10302076	-2.58850971
C	3.06175167	0.11240795	-6.98079118
C	4.65051117	-0.38908431	-5.14105825
H	3.79450347	-0.67436503	-4.51910687
C	4.33632358	-0.25762890	-6.53048378
H	5.14362630	-0.25265747	-7.25905988
O	1.99980871	0.26920653	-6.30899781
O	3.01190610	0.32331139	-8.35981562
C	1.72648366	0.77956000	-8.85265555
H	1.87011486	0.93723763	-9.92616371
H	1.43854852	1.71670636	-8.36162547
H	0.94925574	0.02505402	-8.67761696

C	5.13823969	1.04447914	-2.99969409
H	6.15477913	-0.10941399	-1.61973758
O	4.78275641	1.89786707	-2.12985838
N	4.92829519	1.15217442	-4.33064873
H	2.80332086	2.57539833	-3.33105989
H	1.66031439	-0.49451442	-4.98877336
C	4.23928082	2.24498233	-4.90362184
C	4.65078405	2.65856469	-6.18246234
C	3.91808721	3.61591672	-6.88863225
C	2.78037718	4.19596569	-6.32086779
C	2.39094991	3.82150049	-5.02830132
C	3.10758378	2.85116406	-4.33066460
H	5.54481067	2.21640082	-6.61281890
C	0.01649395	1.43849642	-1.17772470
C	0.44613052	2.77081098	-1.21702253
C	-0.37366000	3.79059608	-0.72612572
C	-1.63057672	3.48416871	-0.19185990
C	-2.06065420	2.15368461	-0.14466943
C	-1.23766923	1.13529034	-0.63466127
H	1.42761059	3.01978731	-1.61582465
H	-0.02891237	4.82377643	-0.75636019
H	-2.26784892	4.27883905	0.19463352
H	-3.03189061	1.90787825	0.28379383
C	-0.28874748	-2.80989251	-2.85552550
C	-1.44530827	-2.18725249	-3.36263061
C	-2.68525515	-2.44913855	-2.78243622
C	-2.78037477	-3.33682609	-1.70429172
C	-1.63782221	-3.98065670	-1.22089065
C	-0.39116247	-3.72596913	-1.79647205
H	-1.37269974	-1.50963444	-4.21028799
H	-3.57740384	-1.96069955	-3.17162873
H	-3.74890751	-3.53177023	-1.24524095
H	-1.71333208	-4.68226646	-0.39161095
C	1.36347712	-3.34256816	-5.22158531
C	2.29698738	-3.00690339	-6.21590514
C	2.32780290	-3.73216764	-7.40668136
C	1.44212418	-4.79562485	-7.60946556
C	0.51968687	-5.13691887	-6.61473215
C	0.47369646	-4.41325489	-5.42087741
H	3.00379861	-2.19138779	-6.09302108
H	3.04771965	-3.45512271	-8.17581510
H	1.46832792	-5.35767480	-8.54242826

H	-0.17325165	-5.96298995	-6.76870102
C	2.66357527	-2.94399396	-2.61361216
C	3.83236434	-3.45595899	-3.20583103
C	4.93888434	-3.75326624	-2.41103309
C	4.88527505	-3.55584318	-1.02863082
C	3.71662236	-3.06882847	-0.43684348
C	2.60787473	-2.76096169	-1.22255354
H	3.89288310	-3.61354564	-4.27797725
H	5.84553574	-4.12564733	-2.87986755
H	5.75230476	-3.78811705	-0.41489832
H	3.65262054	-2.93283061	0.63932811

TS–ModeB–LA1–RA2–*S*

COSMO(toluene)-ZORA-M06-2X/TZ2P//COSMO(toluene)-ZORA-BLYP-D3(BJ)/DZP

E = -25758.09

G = -25183.49

$\boldsymbol{N}_{\mathbf{imag}}$ = 1, 236*i* cm^{-1}

C	4.04079035	0.43295258	2.32144831
C	2.74329820	0.68697904	1.52875779
C	4.50345868	-0.39605945	1.17824776
C	3.32912616	-0.13412486	0.43278707
O	4.51588856	0.74806260	3.41744840
O	1.66664906	1.26761743	1.70875197
N	3.03074467	-0.53650164	-0.80211470
N	5.58548992	-1.12441447	0.91546774
C	6.72957251	-1.15712091	1.83351352
C	6.96366977	-2.61811096	2.25726553
H	6.42966336	-0.56374242	2.70685216
C	7.99402681	-0.47165709	1.20624974
H	5.62943025	-1.67888138	0.05251160
C	1.79276754	-0.29340619	-1.55120455
H	2.07957576	-0.41047620	-2.60227700
C	1.29275471	1.17086121	-1.38430029
H	0.52068346	-0.72154161	0.83637688
H	3.67382140	-1.24465294	-1.20025756
H	0.55161493	1.25807432	-0.58440072
N	0.77537540	1.74089235	-2.63820390
H	2.14241498	1.80348479	-1.09823994
P	-0.71435592	1.52599364	-3.31152404
H	0.94322342	0.33037869	-5.29162153
H	-2.03136234	1.78191096	-5.91279683
H	-2.39451886	3.69395311	-2.33234466

H	9.29295872	-0.65078709	-0.52321544
H	7.63871154	-1.19006905	-0.83680303
H	8.73257538	-2.22483826	0.11206868
H	8.48596106	1.51925017	0.47044526
H	7.29168048	1.52115348	1.79816086
H	6.81014626	1.04466393	0.14793755
H	10.01216743	0.03299517	1.84527710
H	9.44095065	-1.52719944	2.48183459
H	8.83437531	-0.00275084	3.17887885
H	5.49640669	3.23510346	-2.21594723
H	7.96917388	2.91807947	-2.22995273
H	8.92945779	0.73188870	-2.94504142
C	9.13635801	-0.49905326	2.24390661
C	7.61668626	0.99312835	0.88905627
C	8.43835328	-1.18671070	-0.08623965
O	6.98419835	-3.54300688	1.43198399
N	7.16017791	-2.79137716	3.59057311
C	7.38617128	-4.11174969	4.20473853
H	7.02700636	-1.99343578	4.21043808
C	6.04751172	-4.78351612	4.55219021
C	8.30221719	-3.95190788	5.42338946
H	7.89299963	-4.71048188	3.43434251
H	6.21824542	-5.79081853	4.95850045
H	5.42305072	-4.86828498	3.65315146
H	5.50517702	-4.19134327	5.30447985
H	8.50679577	-4.93262729	5.87390165
H	7.82416424	-3.32152774	6.18954458
H	9.25746605	-3.49099345	5.13708432
C	3.54636725	-1.92173451	-5.85418329
C	3.41824972	-2.02569970	-7.24423469
C	3.13521291	-3.25083421	-7.85703972
C	2.96627804	-4.39378034	-7.06665722
C	3.12441331	-4.31907872	-5.68163091
C	3.43281963	-3.09308567	-5.07428228
H	3.48737176	-1.11569826	-7.83772684
H	3.02608787	-3.30826772	-8.93920679
H	2.72306024	-5.35051305	-7.52818164
H	3.01960543	-5.21227814	-5.06518297
N	3.60442882	-3.01057276	-3.68458593
C	3.30514683	1.79267508	-5.58639155
C	3.60269319	-0.60020548	-5.16484601
H	2.73448761	-0.46360997	-4.51601524

C	3.95979691	0.58032903	-5.85592485
H	4.74592951	0.56539082	-6.60725049
O	2.34047204	1.98526520	-4.79413243
O	3.76745868	2.86383624	-6.35001658
C	3.03508766	4.10157719	-6.14520091
H	3.46963568	4.81297738	-6.85433301
H	3.16069858	4.46201466	-5.11656514
H	1.96596643	3.96161123	-6.34099783
C	4.48725542	-2.10218280	-3.06122387
H	3.51446486	-3.87817416	-3.15371867
O	4.86033207	-2.36507700	-1.87715987
N	4.76599858	-1.01444853	-3.77890737
H	4.00482688	1.38055403	-2.91903372
H	1.50934390	1.88504650	-3.37369452
C	5.61646736	0.00641334	-3.31248544
C	7.00914730	-0.16212496	-3.33508117
C	7.85007169	0.87995923	-2.94280437
C	7.31142968	2.10624745	-2.53824280
C	5.92436629	2.28242428	-2.52766797
C	5.08008772	1.24015850	-2.91510875
H	7.41698027	-1.11988088	-3.65506512
C	0.74525943	-1.34611462	-1.22502802
C	0.33673739	-2.26176769	-2.20067217
C	-0.62897917	-3.22832685	-1.90234037
C	-1.17441852	-3.29932387	-0.61709869
C	-0.74423728	-2.40445669	0.37214423
C	0.20891099	-1.43108013	0.07071602
H	0.76871395	-2.21767835	-3.19744974
H	-0.95629914	-3.92097014	-2.67623873
H	-1.92657201	-4.05245736	-0.38265981
H	-1.15854088	-2.46161643	1.37852768
C	-0.88079180	2.90982066	-4.46397575
C	-0.28232268	4.14287500	-4.15466002
C	-0.40198678	5.20813445	-5.04654281
C	-1.10860116	5.04623193	-6.24313083
C	-1.70157191	3.81707595	-6.55089818
C	-1.59020824	2.74454230	-5.66370645
H	0.28741672	4.25282853	-3.23495578
H	0.06926979	6.16205949	-4.81371486
H	-1.18987996	5.87773278	-6.94243213
H	-2.24194247	3.68851138	-7.48771727
C	-0.93973122	0.00135264	-4.27607174

C	-2.06715682	-0.82500070	-4.15253613
C	-2.12988777	-2.01724809	-4.87748414
C	-1.08011177	-2.38184165	-5.72510934
C	0.02483609	-1.53642597	-5.88308650
C	0.09369938	-0.33944343	-5.17101489
H	-2.88453778	-0.55426455	-3.48988391
H	-2.99651166	-2.66782782	-4.76681892
H	-1.12238196	-3.32435274	-6.27025423
H	0.83702828	-1.81574024	-6.55038255
C	-1.94862303	1.59776719	-1.99538157
C	-2.20311242	0.46800607	-1.19835022
C	-3.07351740	0.56925779	-0.11354303
C	-3.69992360	1.78651387	0.17264996
C	-3.45739542	2.90830525	-0.62807440
C	-2.58067813	2.82105389	-1.71078718
H	-1.72087110	-0.47818021	-1.41090514
H	-3.25406023	-0.30475960	0.50976954
H	-4.37873016	1.86227711	1.02126328
H	-3.94810052	3.85515912	-0.40749152

TS–ModeB–LA2–RA1–*S*

COSMO(toluene)-ZORA-M06-2X/TZ2P//COSMO(toluene)-ZORA-BLYP-D3(BJ)/DZP

E = -25760.33

G = -25182.47

$\boldsymbol{N}_{imag}$ = 1, 59*i* cm^{-1}

C	3.01097843	-0.15029601	2.37038138
C	1.71259336	-0.15696659	1.53859642
C	3.76613556	-0.14796331	1.09118092
C	2.57639352	-0.15975239	0.32668310
O	3.32088279	-0.24499751	3.56684634
O	0.49843473	-0.23165985	1.73943590
N	2.35408454	-0.25996016	-0.98070465
N	5.05925903	-0.18338656	0.73589030
C	6.08614375	-0.53355930	1.74527584
C	5.98542316	-2.06793017	1.88735368
H	5.76799219	-0.07047816	2.68829429
C	7.48485657	0.03840655	1.38995018
H	5.24537555	-0.47308002	-0.24556139
C	0.98465222	-0.36308873	-1.53454030
H	0.36147909	-0.79846810	-0.74668263
C	1.07446388	-1.32931188	-2.72434783
H	-1.12296262	0.97324515	-0.42246413

H	3.17020677	-0.41957896	-1.58776761
H	1.44677108	-0.80074635	-3.60859087
N	-0.16498341	-2.08590714	-3.04974862
H	1.80717857	-2.10528290	-2.48653380
P	-1.48619493	-1.34015099	-3.75136758
H	-1.53131828	-1.85675408	-0.90311696
H	-2.30258580	-1.45771077	-6.58899806
H	0.48313873	-1.23800224	-5.77140104
H	8.94717114	0.07244843	-0.21237361
H	7.27510150	0.03183441	-0.80279938
H	8.00526116	-1.44524020	-0.13737022
H	8.36606030	2.02891038	1.23986017
H	7.12089183	1.89250155	2.50807056
H	6.64111755	1.97164391	0.79330654
H	9.47388692	-0.04084638	2.27947955
H	8.59087900	-1.58355132	2.36929562
H	8.15665996	-0.23930339	3.46061645
H	3.40341914	1.17217860	-5.77631894
H	4.09097072	0.13231862	-7.95043081
H	5.00220745	-2.19106697	-7.95367770
C	8.48483158	-0.49247760	2.44091862
C	7.39476571	1.57837068	1.48953603
C	7.95123408	-0.35709268	-0.02937792
O	6.48735111	-2.84387207	1.04549094
N	5.19244038	-2.46764355	2.90391191
C	4.60412062	-3.82178085	2.96571201
H	4.69884508	-1.73296625	3.42911526
C	3.32000544	-3.86910063	2.11588893
C	4.35320821	-4.19827392	4.42955060
H	5.35241636	-4.49781747	2.53195564
H	2.90880287	-4.88846771	2.10251465
H	3.52226354	-3.56898161	1.08064745
H	2.56220877	-3.19058099	2.53438418
H	3.93306275	-5.21121272	4.49166103
H	3.63561320	-3.50213249	4.88954800
H	5.28859619	-4.16837224	5.00464114
C	4.23285205	-5.13225670	-2.66306904
C	4.30404355	-6.50818114	-2.90667005
C	4.93877495	-7.36831655	-2.00376565
C	5.49720861	-6.85067327	-0.82916550
C	5.44553527	-5.48047066	-0.57119379
C	4.83101206	-4.62376596	-1.49660892

H	3.82508929	-6.90543911	-3.79869980
H	4.97980534	-8.43751124	-2.20836909
H	5.97552224	-7.51462697	-0.10927736
H	5.87065944	-5.05780352	0.33534617
N	4.78228478	-3.23790973	-1.24384496
C	1.96239602	-4.14009880	-5.51349460
C	3.43473323	-4.18962151	-3.52317818
H	2.53114130	-3.89012671	-2.97247103
C	3.07512132	-4.64206485	-4.84590395
H	3.74134275	-5.30808279	-5.38675540
O	1.09675666	-3.31419511	-5.09759504
O	1.81908187	-4.63936489	-6.82216631
C	0.63326835	-4.16988755	-7.50700660
H	0.62476872	-4.69671301	-8.46724340
H	0.67754249	-3.08546790	-7.67473707
H	-0.27439177	-4.39852246	-6.93640698
C	4.72946637	-2.28028761	-2.24970762
H	5.29708926	-2.93767859	-0.40348581
O	5.02399823	-1.07839135	-1.97453333
N	4.32979183	-2.72884234	-3.46829818
H	3.57877106	-0.11028246	-3.68693591
H	0.15489604	-2.80200311	-3.75931263
C	4.33646893	-1.89756138	-4.61718054
C	4.77275195	-2.45795790	-5.83069852
C	4.67111722	-1.73392601	-7.02161979
C	4.16338694	-0.43099659	-7.02021615
C	3.77797167	0.15034997	-5.80738772
C	3.85389202	-0.57813522	-4.62091911
H	5.19868864	-3.45474001	-5.83058448
C	0.45028195	1.01775116	-1.89191809
C	1.09333220	1.79430765	-2.86816962
C	0.63929069	3.07802946	-3.16254113
C	-0.47543580	3.59835489	-2.49365132
C	-1.11955828	2.83162076	-1.51959852
C	-0.64804156	1.55129297	-1.21014583
H	1.95249325	1.39865495	-3.40406171
H	1.14370865	3.66849000	-3.92589739
H	-0.83700949	4.59860863	-2.73103538
H	-1.98157839	3.23210413	-0.98679375
C	-2.25653577	-2.62692855	-4.76575015
C	-2.55400026	-3.86215718	-4.16351812
C	-3.10372669	-4.88647592	-4.93337895

C	-3.37436436	-4.67712578	-6.29075447
C	-3.09668955	-3.44131145	-6.88116692
C	-2.53491558	-2.41168543	-6.12313283
H	-2.34442916	-4.02493405	-3.10805366
H	-3.31984570	-5.84985103	-4.47383028
H	-3.80161778	-5.48138647	-6.88852793
H	-3.30591869	-3.27892197	-7.93757478
C	-2.65059809	-0.85818481	-2.45807824
C	-3.82057460	-0.14755068	-2.77826632
C	-4.70923454	0.20806376	-1.76388221
C	-4.44995892	-0.16398058	-0.43923896
C	-3.31272550	-0.91517707	-0.13000628
C	-2.41415441	-1.26928648	-1.13716882
H	-4.04354985	0.11919579	-3.80889522
H	-5.60918110	0.77056251	-2.00821499
H	-5.14442067	0.12120346	0.35010205
H	-3.11508439	-1.21903431	0.89687101
C	-1.02422712	0.02853081	-4.83737201
C	-1.61062589	1.30059087	-4.79219324
C	-1.18880022	2.28148480	-5.69032789
C	-0.17527831	2.00402176	-6.61163325
C	0.43027967	0.74241638	-6.63528882
C	0.01055155	-0.25381595	-5.75573660
H	-2.35556465	1.54441292	-4.04186428
H	-1.63640177	3.27320290	-5.64990729
H	0.15579923	2.77812889	-7.30351127
H	1.23584499	0.52811889	-7.33488172

TS–ModeB–LA2–RA2–*S*

COSMO(toluene)-ZORA-M06-2X/TZ2P//COSMO(toluene)-ZORA-BLYP-D3(BJ)/DZP

E = -25758.72

G = -25181.93

$\boldsymbol{N}_{\mathrm{imag}}$ = 1, 227*i* cm^{-1}

C	4.49017228	1.21906898	1.54995455
C	3.14350482	1.48699443	0.85238389
C	4.76310108	0.19440620	0.51385166
C	3.51694840	0.42100578	-0.12073406
O	5.04085439	1.55985907	2.60689206
O	2.13728655	2.17123130	1.05821241
N	2.97961002	-0.26299514	-1.12575262
N	5.73013833	-0.69900573	0.27449379
C	6.65633819	-1.09396278	1.36599376

C	5.83793170	-2.08904168	2.23099664
H	6.83075411	-0.18645060	1.95926424
C	8.02553708	-1.58035246	0.82758741
H	5.53598116	-1.40637182	-0.45025431
C	1.64700934	-0.10410478	-1.71713258
H	1.75097794	-0.50000988	-2.73326650
C	1.23955533	1.39052395	-1.86497231
H	0.72708125	0.16807422	0.85022211
H	3.46856112	-1.14611731	-1.35504911
H	0.55202569	1.69657938	-1.07082362
N	0.65881147	1.67143397	-3.18619078
H	2.12913702	2.02667202	-1.78390893
P	-0.89046335	1.42136427	-3.66320790
H	0.54878057	-0.31902323	-5.42019103
H	-2.57611390	1.21366870	-6.04316932
H	-2.46521924	3.81551251	-3.15225318
H	8.87100481	-3.01842565	-0.56320176
H	7.34346380	-2.32403791	-1.12735457
H	7.32547670	-3.54756300	0.16969421
H	9.69977350	-0.66378969	-0.23175796
H	8.92890161	0.41387733	0.96209551
H	8.12540985	0.08061096	-0.59429348
H	9.86460869	-2.35879367	1.69898447
H	8.38554883	-3.00255413	2.45738430
H	8.92422650	-1.33829700	2.81366882
H	6.06366619	2.65748054	-2.68494600
H	8.42629166	1.86782583	-2.77123224
H	8.91873442	-0.49678370	-3.38885239
C	8.84633689	-2.10391560	2.02635543
C	8.73617880	-0.35916849	0.20223235
C	7.87265620	-2.69009045	-0.23744534
O	5.74405835	-3.29814864	1.97431054
N	5.13665001	-1.46026805	3.21642115
C	3.92562042	-2.05555446	3.81384528
H	5.21337911	-0.43855431	3.27203139
C	2.69981067	-1.72255656	2.93983409
C	3.76918157	-1.56196506	5.25592264
H	4.09021864	-3.14164771	3.80370295
H	1.79686620	-2.21156658	3.33275772
H	2.85405081	-2.06401913	1.90770752
H	2.52442753	-0.63727726	2.92507791
H	2.88221338	-2.01555622	5.71958111

H	3.64398160	-0.46839150	5.27878739
H	4.65275082	-1.82388633	5.85387784
C	2.92640669	-2.39121284	-5.71637546
C	2.68859646	-2.64124940	-7.07227504
C	2.19315174	-3.87682510	-7.50438143
C	1.92247810	-4.87901626	-6.56496923
C	2.18429235	-4.66131875	-5.21065603
C	2.70277727	-3.43000644	-4.78604294
H	2.84862266	-1.83184966	-7.78244479
H	2.00104287	-4.04794636	-8.56298502
H	1.51992148	-5.83935212	-6.88655957
H	2.00011726	-5.44869476	-4.47921705
N	2.99229178	-3.20639902	-3.43031247
C	3.33672840	1.32028034	-5.90295041
C	3.26967355	-1.02925627	-5.20673061
H	2.49794902	-0.65405243	-4.52841633
C	3.74494404	-0.01264405	-6.07148329
H	4.44506380	-0.24760571	-6.86983548
O	2.53839866	1.79829568	-5.04611586
O	3.89415875	2.18954791	-6.84334367
C	3.39975498	3.55315262	-6.76946437
H	3.93416776	4.09482222	-7.55618163
H	3.61277210	3.99381889	-5.78799263
H	2.31692232	3.58435668	-6.94679287
C	4.06146703	-2.39654995	-2.99419960
H	2.80375712	-3.97134896	-2.78052899
O	4.47681153	-2.55525679	-1.80521345
N	4.46354783	-1.48012721	-3.87510993
H	4.21027987	1.09016652	-3.21129991
H	1.37055652	1.62402917	-3.96986700
C	5.51454275	-0.58496024	-3.57902765
C	6.84550634	-1.02220283	-3.64935147
C	7.88837829	-0.14394930	-3.35426663
C	7.61163142	1.18339764	-3.00541535
C	6.28604990	1.62565324	-2.95624359
C	5.23909169	0.74776528	-3.24500114
H	7.04416561	-2.05836759	-3.91978007
C	0.61901600	-0.95810526	-0.99428329
C	0.02287654	-2.04107001	-1.65015896
C	-0.93804622	-2.81946521	-0.99641498
C	-1.28949082	-2.53116452	0.32596860
C	-0.67540906	-1.46269370	0.99340149

C	0.27020658	-0.67638306	0.33615004
H	0.29844868	-2.26586232	-2.67824572
H	-1.41290102	-3.64693417	-1.52234049
H	-2.03684281	-3.13784555	0.83734140
H	-0.94150750	-1.23789262	2.02597350
C	-1.10992894	2.50168130	-5.09486736
C	-0.37186698	3.69486414	-5.18310393
C	-0.55824506	4.53631341	-6.28020049
C	-1.47355697	4.19252799	-7.28217420
C	-2.20487065	3.00163142	-7.19421892
C	-2.02423635	2.15013554	-6.10294548
H	0.35189013	3.94350103	-4.40990872
H	0.01847404	5.45762257	-6.35680290
H	-1.61198502	4.85020588	-8.13970251
H	-2.90910949	2.73079805	-7.98070135
C	-1.26396166	-0.26754029	-4.22104124
C	-2.40556629	-0.97475112	-3.81529720
C	-2.58361090	-2.29645931	-4.23188056
C	-1.63632638	-2.90531752	-5.05910204
C	-0.51577971	-2.18863631	-5.49711745
C	-0.32707302	-0.87127293	-5.08274162
H	-3.14408674	-0.50889025	-3.16832792
H	-3.46085309	-2.85115853	-3.90075130
H	-1.76900739	-3.94071445	-5.37137594
H	0.20956132	-2.65816216	-6.15415374
C	-2.00663229	1.86629105	-2.31629389
C	-2.21158335	0.98254173	-1.24101213
C	-3.00779186	1.37696931	-0.16605778
C	-3.61030443	2.64026176	-0.16410140
C	-3.41715086	3.51508244	-1.23932652
C	-2.61471222	3.13479258	-2.31687795
H	-1.75118912	0.00118553	-1.23541973
H	-3.15141356	0.69467098	0.66999670
H	-4.23222525	2.94477524	0.67715862
H	-3.88943784	4.49710297	-1.23896706

methyl acrylate

COSMO(toluene)-ZORA-M06-2X/TZ2P//COSMO(toluene)-ZORA-BLYP-D3(BJ)/DZP

$\boldsymbol{E}$ = -2315.25

$\boldsymbol{G}$ = -2276.98

$\boldsymbol{N}_{imag}$ = 0

C	-1.68675780	-1.52956762	-0.72924969

H	-2.42263683	-0.73326906	-0.83765877
C	-0.44975131	-1.25859780	-0.30110715
H	0.30310469	-2.03834585	-0.18371502
O	1.27183302	0.13826572	0.44820056
H	1.73861637	2.12732644	-0.05896330
C	-0.03496535	0.12605865	0.04206534
O	-0.73964068	1.12520083	-0.02092880
C	1.78910291	1.45998877	0.80782783
H	1.20089688	1.87893360	1.63092568
H	2.82356433	1.28611574	1.11113113
H	-1.99080570	-2.54435770	-0.97956684

dimethylurea
COSMO(toluene)-ZORA-M06-2X/TZ2P//COSMO(toluene)-ZORA-BLYP-D3(BJ)/DZP
$\boldsymbol{E}$ = -2611.87
$\boldsymbol{G}$ = -2560.35
$\boldsymbol{N}_{imag}$ = 0

H	-2.39494922	4.07472871	1.46055424
H	-3.53028941	4.10369821	2.83595810
H	-8.18693387	2.64151680	0.40100115
O	-6.07456986	3.50564238	2.41039408
H	-8.64399288	4.20144142	-0.35407480
H	-8.40874579	4.09286492	1.41096509
N	-6.65153089	4.13004659	0.26847060
N	-4.41344163	4.10055345	0.93048157
C	-3.34471452	3.68326694	1.84150181
C	-5.73409113	3.86267650	1.27051371
H	-3.27665161	2.58800075	1.93412520
H	-6.30694474	4.18604347	-0.68914602
H	-4.17681822	4.15009869	-0.05959171
C	-8.05109603	3.73401249	0.44006351

dimethylthiourea
COSMO(toluene)-ZORA-M06-2X/TZ2P//COSMO(toluene)-ZORA-BLYP-D3(BJ)/DZP
$\boldsymbol{E}$ = -2490.26
$\boldsymbol{G}$ = -2440.24
$\boldsymbol{N}_{imag}$ = 0

H	-3.24717177	2.72216835	2.20891024
H	-2.40106957	4.04174258	1.33491922
H	-8.34538542	2.76594387	0.85994935
S	-6.21492079	3.27294068	2.84881364
H	-8.53696022	3.81553297	-0.57627668

H	-8.45327358	4.53120525	1.06775542
N	-6.62728437	3.81647752	0.24073928
N	-4.43710574	3.94426701	0.93931198
C	-3.31920055	3.76344246	1.86420476
C	-5.73820026	3.69762475	1.27199541
H	-3.44478864	4.40981216	2.74101716
H	-6.29886308	4.26413766	-0.61578294
H	-4.20415148	4.00996765	-0.05212681
C	-8.07690490	3.73062044	0.41458232

dimethylsquaramide

COSMO(toluene)-ZORA-M06-2X/TZ2P//COSMO(toluene)-ZORA-BLYP-D3(BJ)/DZP

E = -3662.31

G = -3600.64

$\boldsymbol{N}_{imag}$ = 0

C	-6.21359213	2.22551465	1.77085581
C	-7.56216265	2.61096089	1.13426919
C	-5.57210941	3.20812307	0.85343125
C	-6.80953323	3.56010768	0.26794292
O	-5.80759578	1.45748048	2.64451497
O	-8.74767879	2.29997284	1.25992843
N	-7.18065978	4.42710372	-0.68299212
N	-4.30634620	3.60319652	0.67059086
C	-3.17288807	3.03130696	1.41326670
H	-8.88130136	5.58229190	-1.17118730
H	-3.56665890	2.31639872	2.14152158
H	-2.48949017	2.51245417	0.72779487
H	-4.09959428	4.29651469	-0.04894201
C	-8.56496845	4.53176279	-1.17005826
H	-9.20896835	3.95764266	-0.49769023
H	-2.62713837	3.82479429	1.94025058
H	-8.64947800	4.12411925	-2.18694295
H	-6.46264513	4.94849925	-1.18698445

N-phenylurea

COSMO(toluene)-ZORA-M06-2X/TZ2P//COSMO(toluene)-ZORA-BLYP-D3(BJ)/DZP

E = -3719.16

G = -3654.03

$\boldsymbol{N}_{imag}$ = 0

C	-4.10877813	1.53737612	-1.75311124
C	-5.43368525	1.32338923	-1.37601735
H	-6.98173776	2.62850637	-4.10986810

H	-7.51667271	1.54536402	-1.92623145
H	-5.64612557	0.84994648	-0.41786957
C	-6.48130562	1.71198390	-2.21966847
C	-6.17785813	2.31836796	-3.44239346
C	-4.85470554	2.54138871	-3.83523169
H	-3.29903018	1.23002194	-1.09038633
C	-1.87910188	2.92699410	-4.41195012
H	0.00483603	2.21753792	-3.87108382
O	-2.52449928	3.43658606	-5.33299872
N	-2.44111621	2.32410851	-3.29480574
H	-4.62481133	3.01589659	-4.78171642
N	-0.48625705	2.96107308	-4.36833442
C	-3.80525624	2.14893105	-2.98555619
H	-0.06991014	3.19559493	-5.26999987
H	-1.79719562	2.02356481	-2.56425438

deprotonated N-phenylurea

COSMO(toluene)-ZORA-M06-2X/TZ2P//COSMO(toluene)-ZORA-BLYP-D3(BJ)/DZP

E = -3655.42

G = -3597.72

N_{imag} = 0

C	-4.09192229	1.52534688	-1.77300011
C	-5.41426111	1.30396939	-1.39798249
H	-6.96679275	2.67986517	-4.09481950
H	-7.51048917	1.54273574	-1.93389835
H	-5.62584145	0.80652358	-0.44895627
C	-6.47408082	1.71415787	-2.22537604
C	-6.15998105	2.35083516	-3.43516379
C	-4.83731821	2.57687870	-3.82495943
H	-3.27250487	1.20848232	-1.12584460
C	-1.93956169	2.88295117	-4.39158333
H	-0.05472841	2.40029121	-3.72504838
O	-2.54527787	3.28167214	-5.42803455
N	-2.39406278	2.33477155	-3.24144781
H	-4.60940359	3.06601089	-4.76661529
N	-0.51328402	3.05465126	-4.36317373
C	-3.74181976	2.17022181	-2.99959375
H	-0.11433650	3.03050834	-5.30408917

U–MA

COSMO(toluene)-ZORA-M06-2X/TZ2P//COSMO(toluene)-ZORA-BLYP-D3(BJ)/DZP

E = -4933.27

G = -4830.72
N$_{imag}$ = 0

H	-3.05210047	5.87650815	1.75104718
H	-3.71921997	4.77779827	2.98875390
C	-5.44338408	3.57424823	1.32655691
O	-5.40957201	2.90002699	2.37206355
H	-7.13541810	1.50889820	1.00814917
H	-7.90431665	2.16805180	-0.46118027
N	-6.31194037	3.29475894	0.28231448
N	-4.60889827	4.64902138	1.09487800
C	-3.43305082	4.86520733	1.93492560
O	-5.27994060	5.37715995	-1.77069268
H	-2.63113132	4.13604436	1.73275730
O	-4.29008699	6.04223893	-3.71363895
H	-4.59990748	5.02991811	0.14664162
C	-7.46651875	2.42635421	0.50999049
H	-8.23658354	2.90307256	1.13831898
C	-4.48746701	5.16834224	-2.68961912
H	-2.91236626	4.00972956	-3.67691498
H	-6.40124320	4.01118328	-0.44003941
C	-3.70911934	2.91657431	-2.03933660
H	-4.43428835	2.87256953	-1.22807070
C	-3.62358207	3.97723119	-2.85109183
C	-5.09727906	7.26475810	-3.65273680
H	-4.80108389	7.84065989	-4.53171369
H	-6.16087163	7.00802548	-3.68775178
H	-4.87538663	7.80882244	-2.72913623
H	-3.05895443	2.05466722	-2.18210702

TU–MA

COSMO(toluene)-ZORA-M06-2X/TZ2P//COSMO(toluene)-ZORA-BLYP-D3(BJ)/DZP

E = -4812.66

G = -4711.00

N$_{imag}$ = 0

H	-2.83414956	5.66831451	1.71782333
H	-3.79042291	5.04945513	3.09985037
C	-5.42940228	3.58359366	1.32381163
S	-5.43533646	2.52803001	2.66402122
H	-7.03480183	1.49531296	0.33778551
H	-7.95757109	2.65092774	-0.67326859
N	-6.33994254	3.49223375	0.31337522
N	-4.50569408	4.57211507	1.16784037

C	-3.40853631	4.81562158	2.09732451
O	-5.08728812	5.48956848	-1.62230315
H	-2.75201655	3.93787605	2.17837423
O	-4.49769665	5.90594603	-3.78187516
H	-4.53566521	5.11344346	0.30018626
C	-7.42511094	2.51967850	0.27554953
H	-8.12403361	2.67010668	1.11013020
C	-4.50784959	5.14759022	-2.65546793
H	-3.30081192	3.76909538	-3.85232548
H	-6.26157405	4.16090486	-0.45546415
C	-3.60682037	2.96789245	-1.91158359
H	-4.04883010	3.08597591	-0.92640644
C	-3.74627890	3.89636411	-2.86545324
C	-5.22887146	7.17362137	-3.68847820
H	-5.11290681	7.63737844	-4.66987166
H	-6.28237961	6.97679981	-3.46563336
H	-4.79082854	7.79557234	-2.90159560
H	-3.04058231	2.05774959	-2.10218799

SQ–MA

COSMO(toluene)-ZORA-M06-2X/TZ2P//COSMO(toluene)-ZORA-BLYP-D3(BJ)/DZP

E = -5985.24

G = -5872.93

$\boldsymbol{N}_{\mathrm{imag}}$ = 0

C	-6.24251953	2.01194292	1.16696903
C	-7.57537390	2.68344253	0.78342691
C	-5.55154407	3.20158224	0.59968979
C	-6.77702882	3.81915724	0.24667323
O	-5.87319534	0.96059687	1.69718220
O	-8.77951869	2.42520894	0.86102803
N	-7.08581542	4.97453536	-0.35114108
N	-4.26998639	3.55766343	0.46112889
C	-3.15292861	2.70868732	0.89118800
O	-4.43157573	6.01863974	-1.20168467
H	-3.56004895	1.81023766	1.36421148
O	-3.39924256	7.02992694	-2.95960853
H	-4.06957404	4.43949541	-0.01995361
C	-8.46624699	5.38770663	-0.63032885
H	-9.14418697	4.62613011	-0.23386182
C	-4.07777977	6.00785241	-2.38514374
H	-8.67987104	6.35091536	-0.14767062
H	-6.31425593	5.57448486	-0.65741920

C	-4.97495987	3.80109941	-3.05933034
H	-5.37923970	3.61440383	-2.06930474
C	-4.31534017	4.92496394	-3.36328427
C	-3.10812652	8.16735280	-2.07710028
H	-2.55396840	8.86964550	-2.70217425
H	-4.04616963	8.60578650	-1.72269376
H	-2.50710743	7.82963166	-1.22738626
H	-3.91928634	5.09408362	-4.36475395
H	-2.54020809	2.41560267	0.02715768
H	-2.52262193	3.24538398	1.61295972
H	-8.62467002	5.48474846	-1.71330263
H	-5.13045965	3.02746754	-3.80942558

TS$_{\mathbf{uncatalyzed}}$

COSMO(toluene)-ZORA-M06-2X/TZ2P//COSMO(toluene)-ZORA-BLYP-D3(BJ)/DZP

$\boldsymbol{E}$ = -5967.72

$\boldsymbol{G}$ = -5858.40

$\boldsymbol{N}_{\text{imag}}$ = 1, 253i cm^{-1}

H	-0.00670841	2.46707357	-3.02674704
H	-2.51676693	5.87447976	-4.15730365
C	-1.95297309	3.11061328	-2.33334748
N	-0.20719095	2.80765902	-0.10094907
C	-3.34326968	2.91985896	-2.48259302
C	-3.83646793	1.92465656	-3.33007394
H	-0.88778312	0.65418634	-4.47518274
H	-3.35416094	0.31368953	-4.69744280
H	-4.91471167	1.79600896	-3.43132938
O	-4.96915002	5.75997028	-1.91257686
C	-2.96430152	1.09295767	-4.04248906
O	-4.99437111	5.89779508	-4.22289183
C	-1.58010604	1.28268903	-3.91309255
C	-1.08121507	2.29300313	-3.08911115
H	-4.02222944	3.55436642	-1.91755318
C	-4.30953319	5.85008920	-2.96949509
H	-1.01216589	6.24633653	-2.18401860
H	-0.43977264	2.00322229	-0.68342389
C	-2.02358376	5.86807694	-2.03605579
H	-2.44505542	6.15267488	-1.07101344
C	-2.90781094	5.91318997	-3.14143431
C	-6.42636020	5.76987140	-4.11232460
H	-6.80765061	5.81378281	-5.13916928
H	-6.70594352	4.81357305	-3.64820862

H	-6.85915160	6.58432771	-3.51470593
C	-0.49939521	4.09033278	-0.63270243
H	0.73234694	2.76452232	0.29559093
O	0.08339375	5.09051997	-0.15248043
N	-1.49278687	4.16798386	-1.55375965

TS$_{U–MA}$
COSMO(toluene)-ZORA-M06-2X/TZ2P//COSMO(toluene)-ZORA-BLYP-D3(BJ)/DZP
E = -8593.17
G = -8416.41
N_{imag} = 1, 292*i* cm^{-1}

H	-2.57864623	3.15167138	1.29449440
H	-3.88702599	2.14447598	1.98485773
C	-5.63686649	2.92697210	-0.04108698
N	-0.64585685	1.10105860	-0.78645701
H	-7.07821727	2.63062004	-2.46782661
H	-7.98946022	4.12841045	-2.10406680
N	-6.37376611	3.80924840	-0.82178457
N	-4.44831265	3.44707260	0.42118498
C	-3.47175293	2.56380769	1.05854479
O	-4.33184873	5.76010801	-1.40765735
H	-3.17354871	1.73582604	0.40024520
O	-4.27830152	6.36994674	-3.62913645
H	-4.09038620	4.26669850	-0.08298303
C	-7.44872391	3.28420996	-1.65876162
H	-8.14611866	2.69992076	-1.04637191
C	-3.65672746	5.88230554	-2.46391711
O	-6.04197080	1.78171152	0.24776440
H	-5.84622331	4.61398317	-1.18604295
C	-1.50036707	5.04043139	-1.63495034
H	-1.83853424	5.16739825	-0.60787246
C	-2.28996697	5.59643151	-2.65846357
C	-5.70425121	6.57856923	-3.51428212
H	-6.00747477	7.05655634	-4.45183255
H	-6.23087887	5.62114239	-3.40184689
H	-5.94764251	7.22195936	-2.65995863
C	-0.66122981	2.51564423	-0.80605576
H	0.22809474	0.72654129	-0.41624328
O	0.05835563	3.14103955	0.00886995
N	-1.56717504	3.11538745	-1.61715079
H	-0.29014563	1.81825770	-3.62103675
H	-1.89866702	5.67870425	-3.67111107

C	-2.13431179	2.54386728	-2.75020565
C	-3.51495816	2.71814880	-2.98242607
C	-4.11043384	2.25234628	-4.15577244
H	-1.36014210	0.93772074	-5.67935456
H	-3.81499532	1.22858528	-6.04249571
H	-5.18012204	2.39672785	-4.30786446
C	-3.34770232	1.59753778	-5.12958368
C	-1.97016731	1.43060658	-4.92089800
C	-1.36738217	1.91365534	-3.75845699
H	-4.10932602	3.20451916	-2.21965853
H	-0.41691819	5.07503716	-1.73939650
H	-0.98424612	0.61819589	-1.61841052

TS$_{TU\text{–}MA}$

COSMO(toluene)-ZORA-M06-2X/TZ2P//COSMO(toluene)-ZORA-BLYP-D3(BJ)/DZP

E = -8473.82

G = -8297.56

$\boldsymbol{N}_{imag}$ = 1, 278*i* cm^{-1}

H	-2.55370399	3.10352255	1.16749165
H	-3.87466633	2.21538107	1.99906769
C	-5.65573685	2.89553385	-0.07814446
N	-0.60910399	1.09705758	-0.79697005
H	-7.65871795	2.54130745	-2.03929006
H	-7.92197364	4.31275344	-2.09740636
N	-6.32473584	3.73972289	-0.91169318
N	-4.42061048	3.32372357	0.28876375
C	-3.46117389	2.50822789	1.02506689
O	-4.35746789	5.70704621	-1.41013811
H	-3.19821569	1.59938216	0.46720598
O	-4.30972100	6.31546445	-3.63236407
H	-4.07266774	4.16981768	-0.18193931
C	-7.64744927	3.47055154	-1.45088697
H	-8.39463859	3.37107373	-0.65024795
C	-3.68425777	5.83916411	-2.46895480
S	-6.33018249	1.41018761	0.45665427
H	-5.82118821	4.58660790	-1.21553772
C	-1.51888952	5.03829640	-1.63065838
H	-1.85651598	5.16145432	-0.60331773
C	-2.31243929	5.57632130	-2.65750069
C	-5.74113100	6.49187603	-3.53035555
H	-6.04448489	6.97789116	-4.46349279
H	-6.24562055	5.52032969	-3.44103174

H	-6.00755769	7.11609846	-2.66891072
C	-0.67705549	2.50913652	-0.78215212
H	0.26323504	0.74284952	-0.40408259
O	-0.00617191	3.13992650	0.07034141
N	-1.58148412	3.09645544	-1.60326047
H	-0.18990934	1.92642877	-3.60752444
H	-1.92058855	5.65929595	-3.66970284
C	-2.09048821	2.53416813	-2.76882978
C	-3.47095196	2.64971358	-3.03520107
C	-4.00940587	2.20060441	-4.24227896
H	-1.15697327	1.07600439	-5.72918344
H	-3.61112823	1.26580762	-6.15605962
H	-5.08115181	2.29726096	-4.41850699
C	-3.18854153	1.62150478	-5.21644111
C	-1.81120222	1.51110357	-4.97220129
C	-1.26610221	1.97725365	-3.77496937
H	-4.11453420	3.07113726	-2.27336740
H	-0.43587113	5.07058654	-1.73845598
H	-0.90550987	0.62229640	-1.64934887

TS$_{\mathbf{SQ-MA}}$

COSMO(toluene)-ZORA-M06-2X/TZ2P//COSMO(toluene)-ZORA-BLYP-D3(BJ)/DZP

E = -9649.45

G = -9461.74

$\boldsymbol{N}_{imag}$ = 1, 225*i* cm^{-1}

H	-0.03335930	2.51928968	-2.96272124
H	-2.51066918	5.78422768	-4.23885191
C	-2.00649658	3.09012106	-2.27678276
N	-0.21432063	2.79487538	-0.06261326
C	-3.38838482	2.87070238	-2.46619148
C	-3.84427824	1.90731659	-3.36849417
H	-0.84586159	0.76822685	-4.51898792
H	-3.29738481	0.37884726	-4.80406114
H	-4.91707967	1.75705323	-3.49496723
O	-4.87684994	5.78231881	-1.91324166
C	-2.93915883	1.13349017	-4.10413568
O	-5.00827199	5.87880575	-4.20413228
C	-1.56293915	1.35087439	-3.93887480
C	-1.10244149	2.32710628	-3.05492421
H	-4.09344772	3.46243705	-1.88872081
C	-4.27091567	5.85375472	-3.01780104
H	-0.93737702	6.14973575	-2.33144133

H	-0.43484421	1.98283674	-0.63875010
C	-1.98419384	5.92297843	-2.13854970
H	-2.34044191	6.29701889	-1.18136381
C	-2.87709210	5.89778293	-3.22040325
C	-6.44143686	5.75591573	-4.03537325
H	-6.84821788	5.67651900	-5.04851196
H	-6.69224663	4.86235105	-3.45088063
H	-6.85682657	6.64153722	-3.53622204
C	-0.55969661	4.06677034	-0.57460431
H	0.72423682	2.77643543	0.33739111
O	0.00153229	5.08337417	-0.10203788
N	-1.60026461	4.12412797	-1.44443742
C	-5.86217340	5.52963339	2.80162803
C	-5.37197858	5.54911086	1.39441120
O	-5.39397991	5.16405824	3.88891846
N	-4.24854427	5.18647043	0.78087140
C	-3.10829650	4.58390411	1.48382441
H	-3.33132558	4.55905046	2.55502878
H	-4.20381836	5.32195205	-0.24367387
H	-2.93348551	3.56476521	1.11602149
H	-2.19911635	5.17036406	1.30296601
C	-7.16879758	6.15019013	2.29578388
C	-6.59099812	6.12466857	0.93218198
O	-8.24968052	6.52108807	2.78321856
N	-7.02035425	6.49165885	-0.27831116
C	-8.35009331	7.05856344	-0.51063903
H	-8.86404661	7.16142174	0.45038826
H	-6.38226232	6.32995092	-1.07612325
H	-8.94140426	6.40154285	-1.16568636
H	-8.26687820	8.04781515	-0.98343770

10. NMR Spectra

^{1}H NMR: (400 MHz, $CDCl_3$, 298K) of **S6**

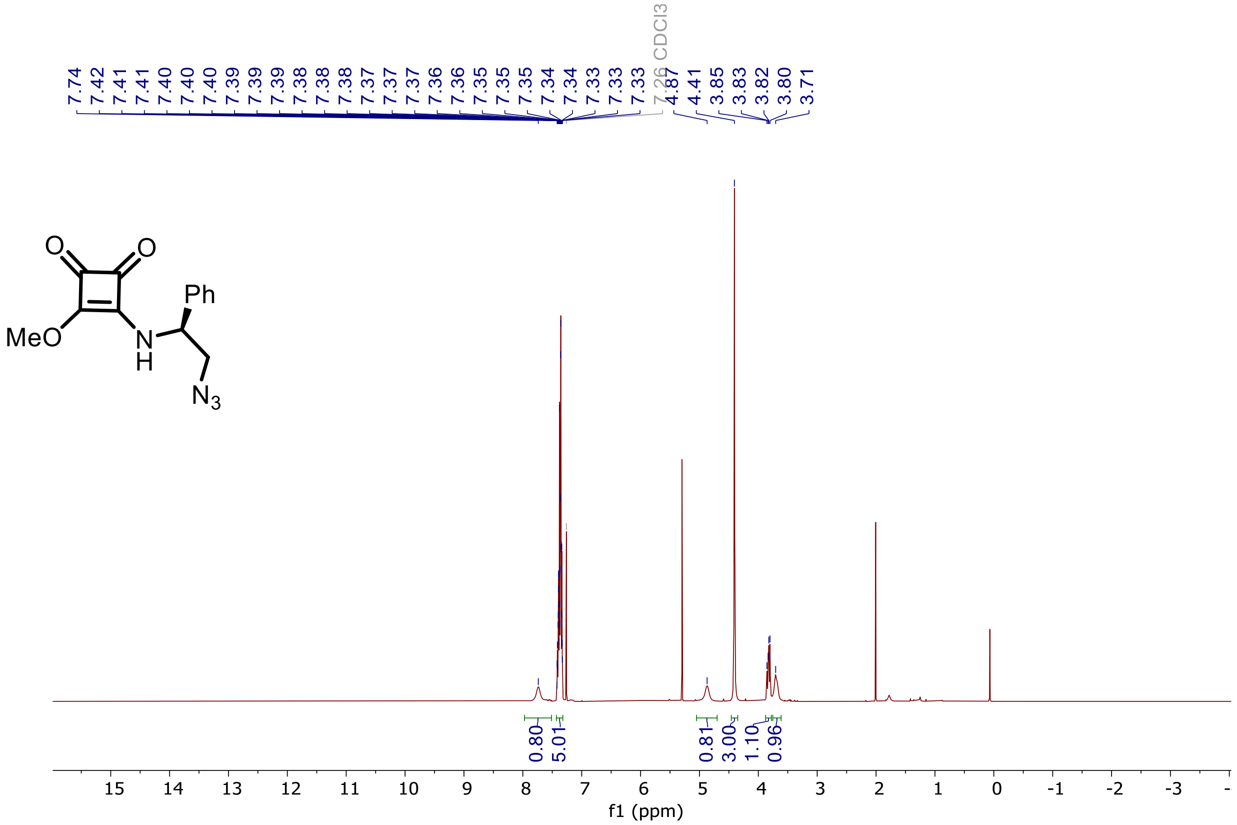

^{13}C NMR: (101 MHz, $CDCl_3$, 298K) of **S6**

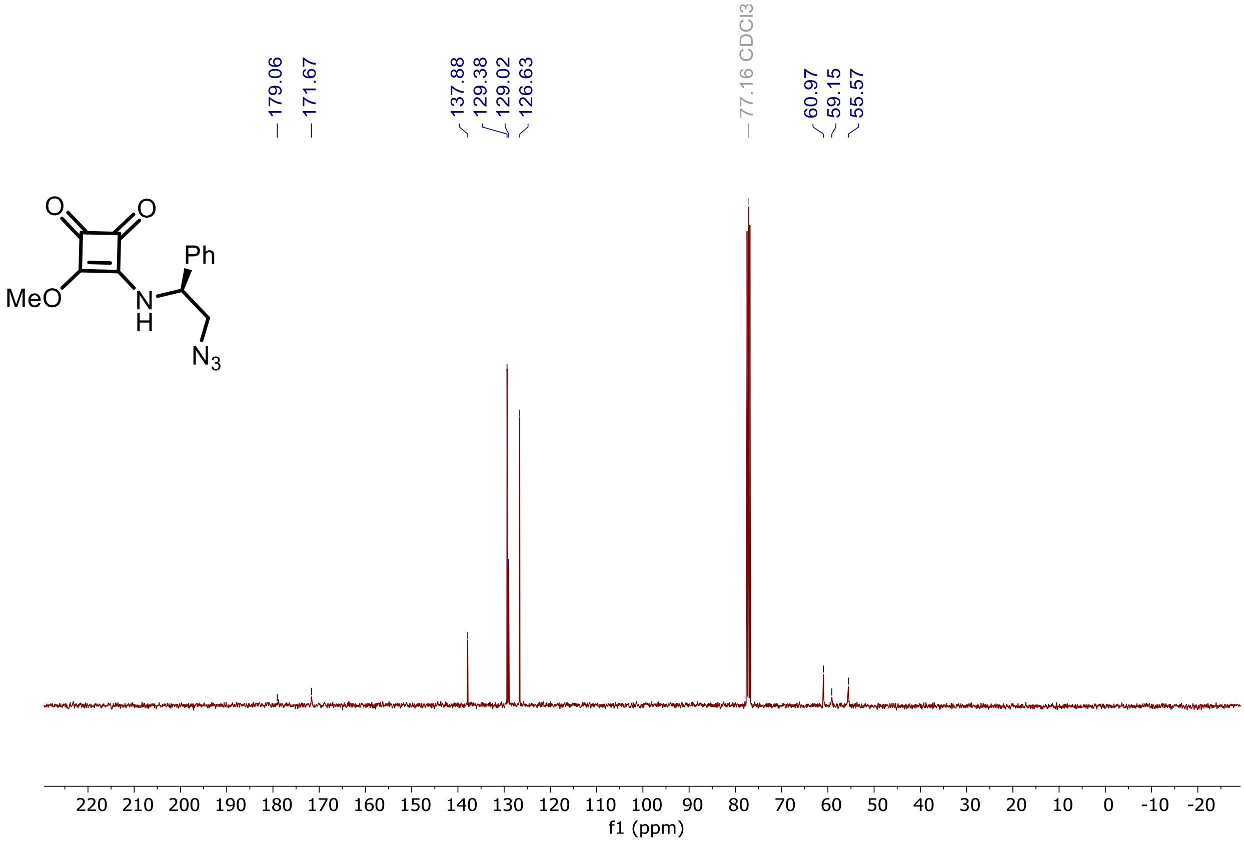

^{1}H NMR: (400 MHz, $CDCl_3$, 298K) of **S8**

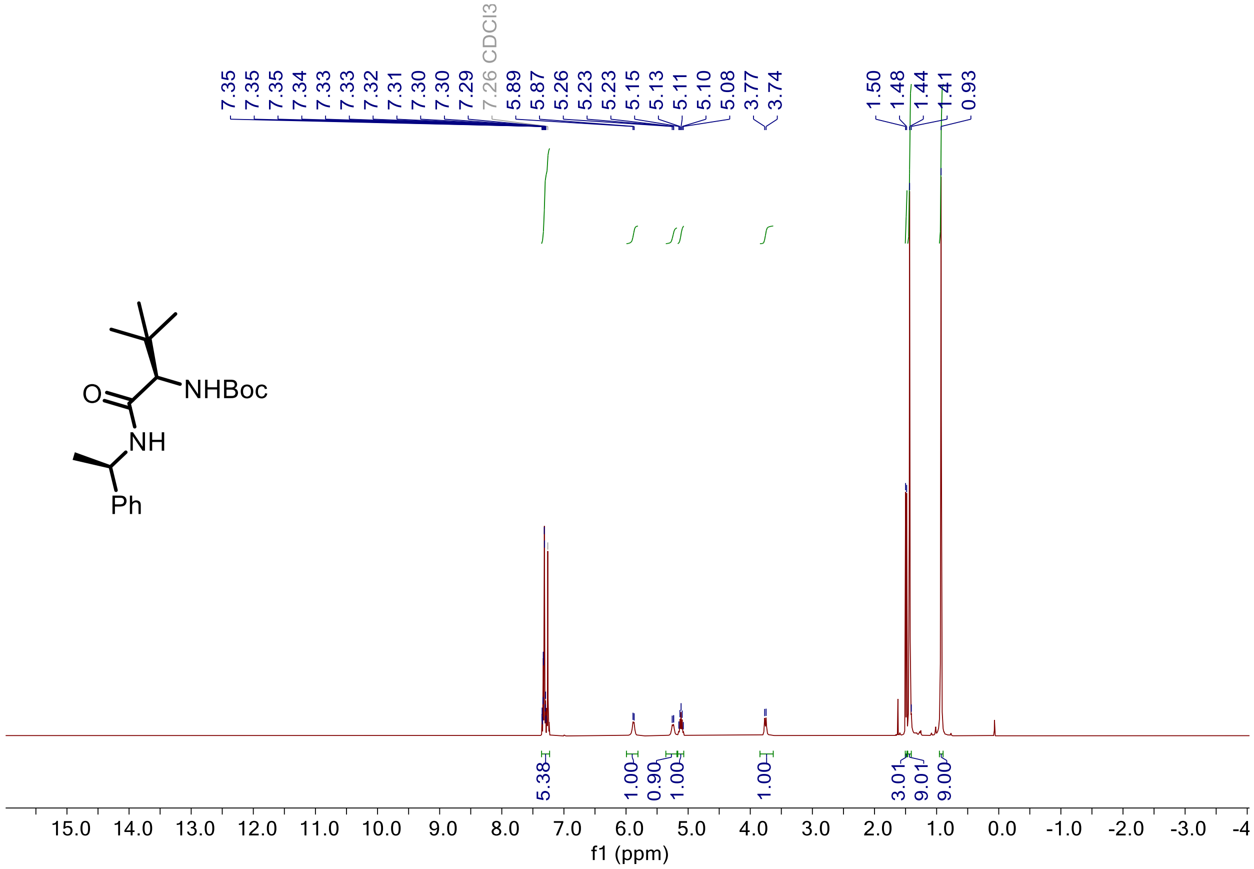

^{13}C NMR: (101 MHz, $CDCl_3$, 298K) **S8**

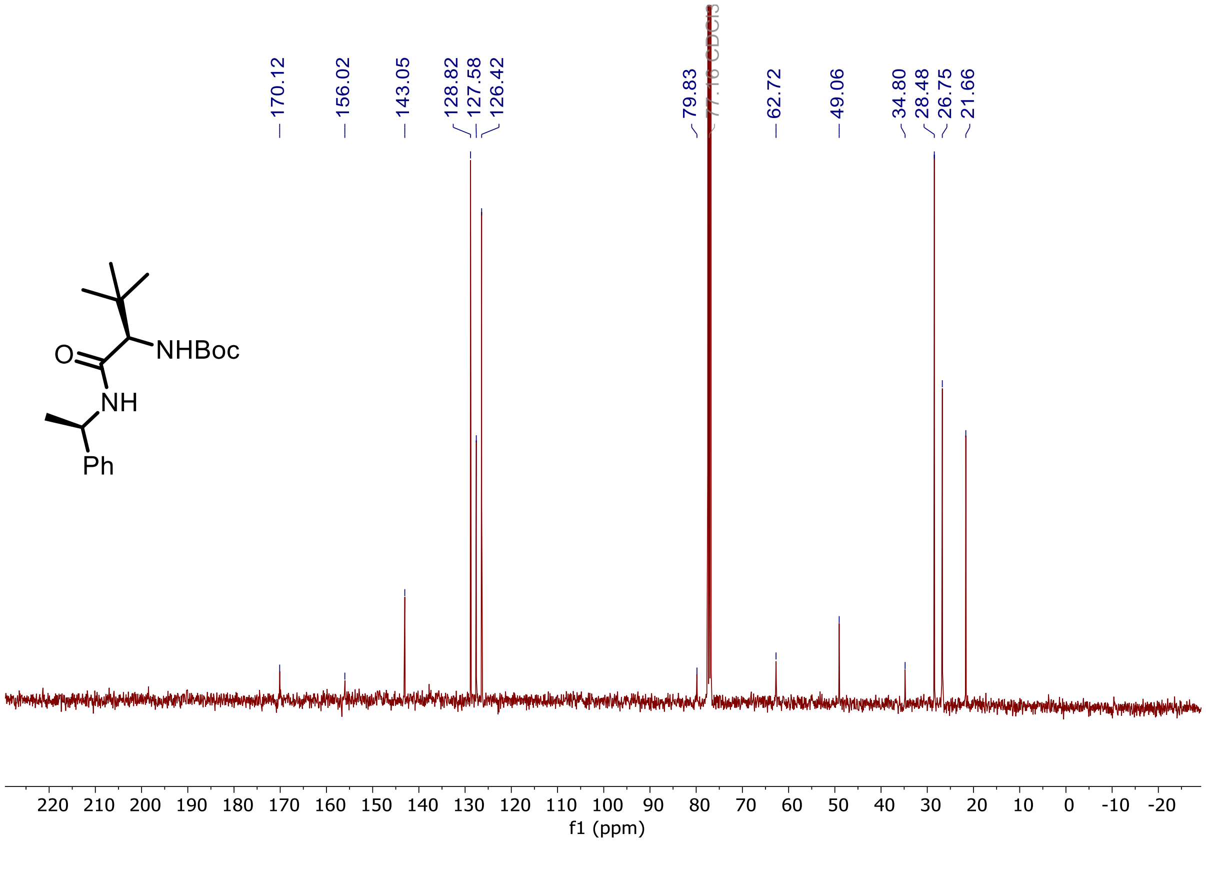

^{1}H NMR: (400 MHz, $(CD_3)_2SO$, 298K) of **Catalyst Precursor S9**

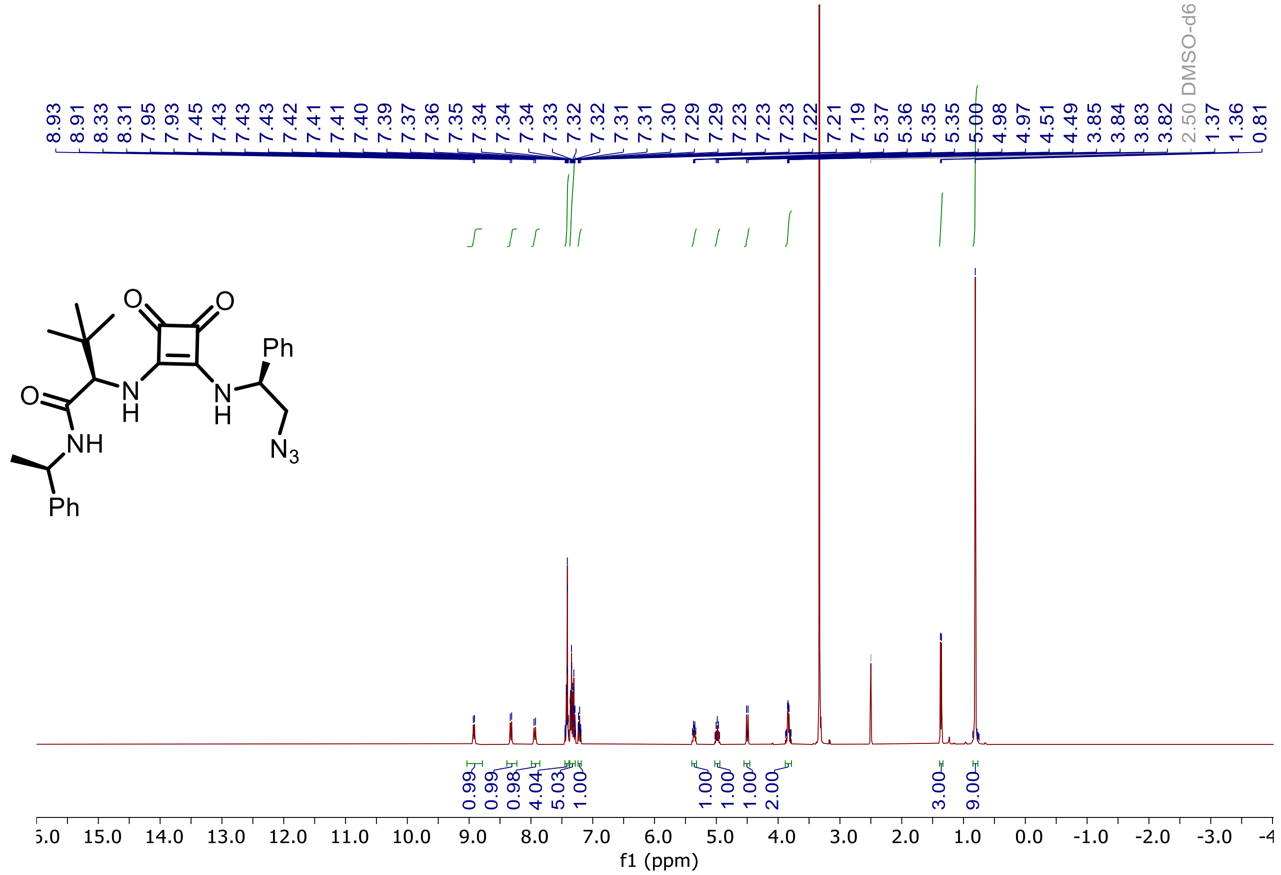

^{13}C NMR: (101 MHz, $(CD_3)_2SO$, 298K) of **Catalyst Precursor S9**

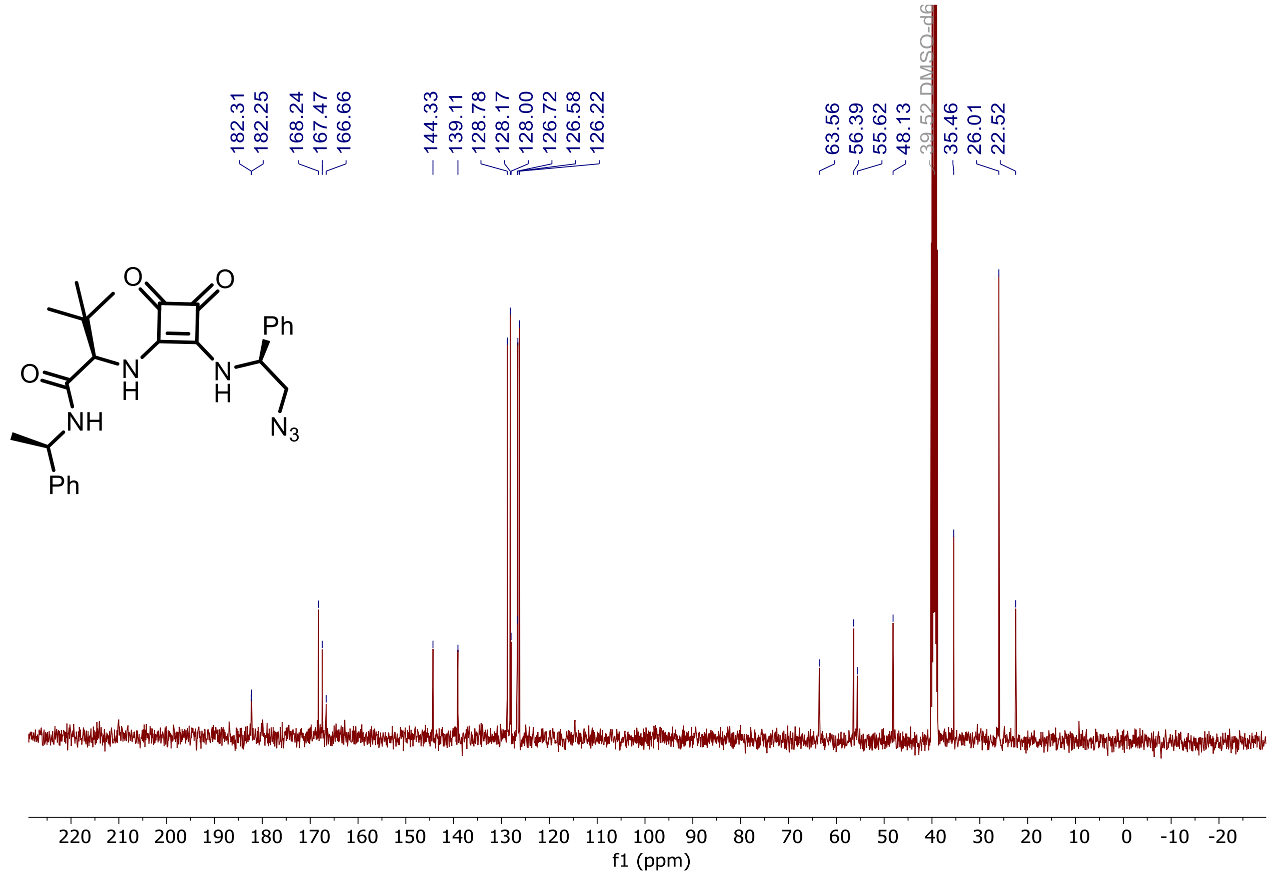

^{1}H NMR: (400 MHz, $(CD_3)_2SO$, 298K) of **1a**

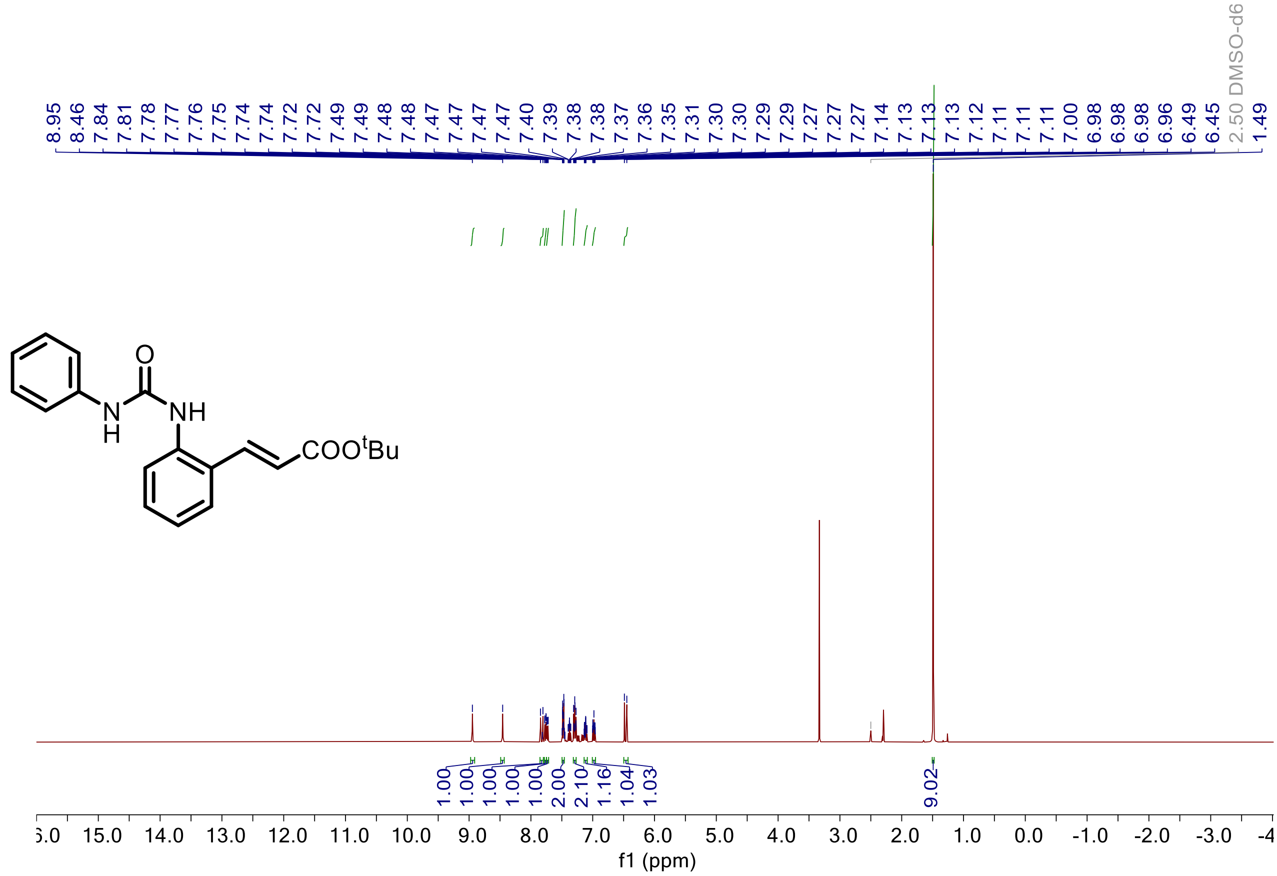

^{13}C NMR: (101 MHz, $(CD_3)_2SO$, 298K) of **1a**

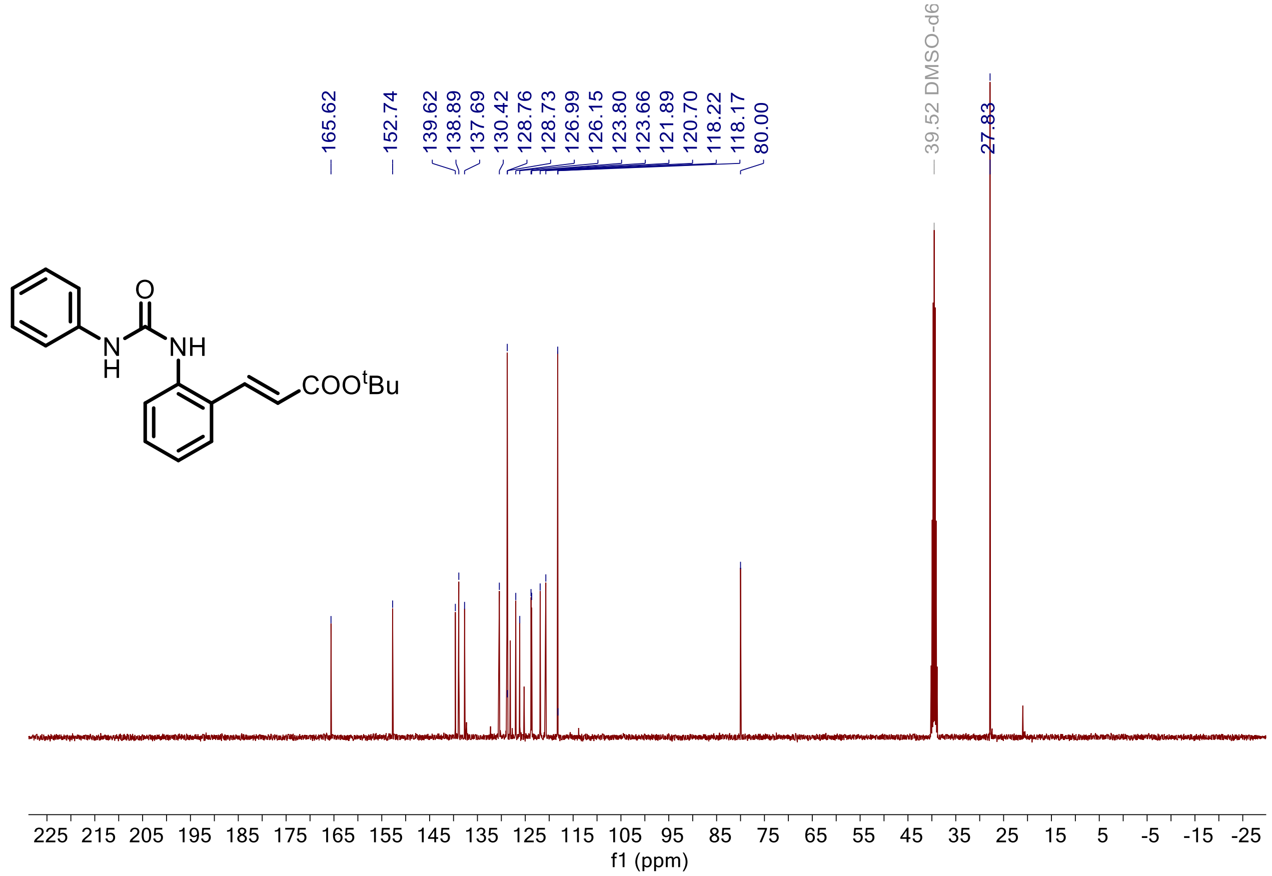

^{1}H NMR: (400 MHz, $CDCl_3$, 298K) of **1b**

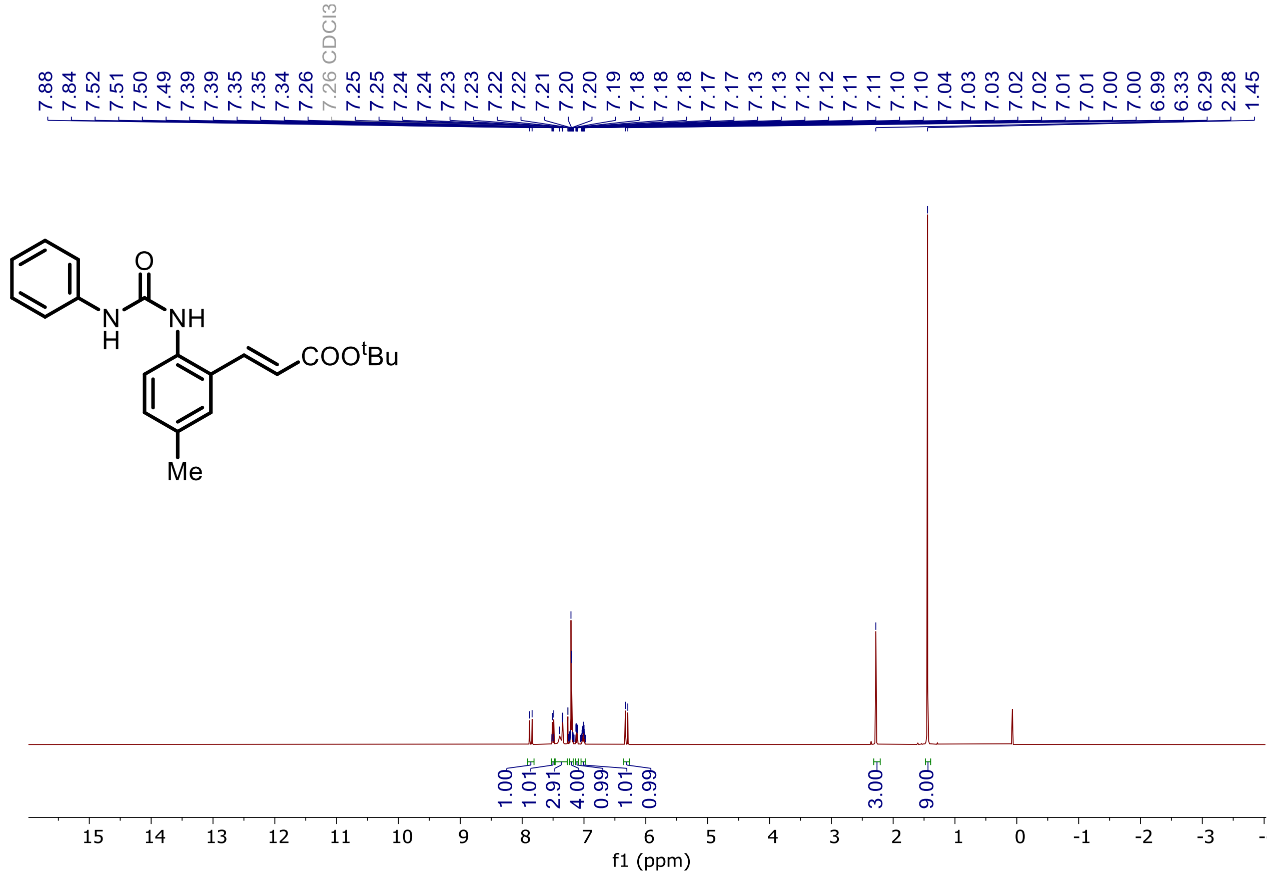

^{13}C NMR: (101 MHz, $CDCl_3$, 298K) of **1b**

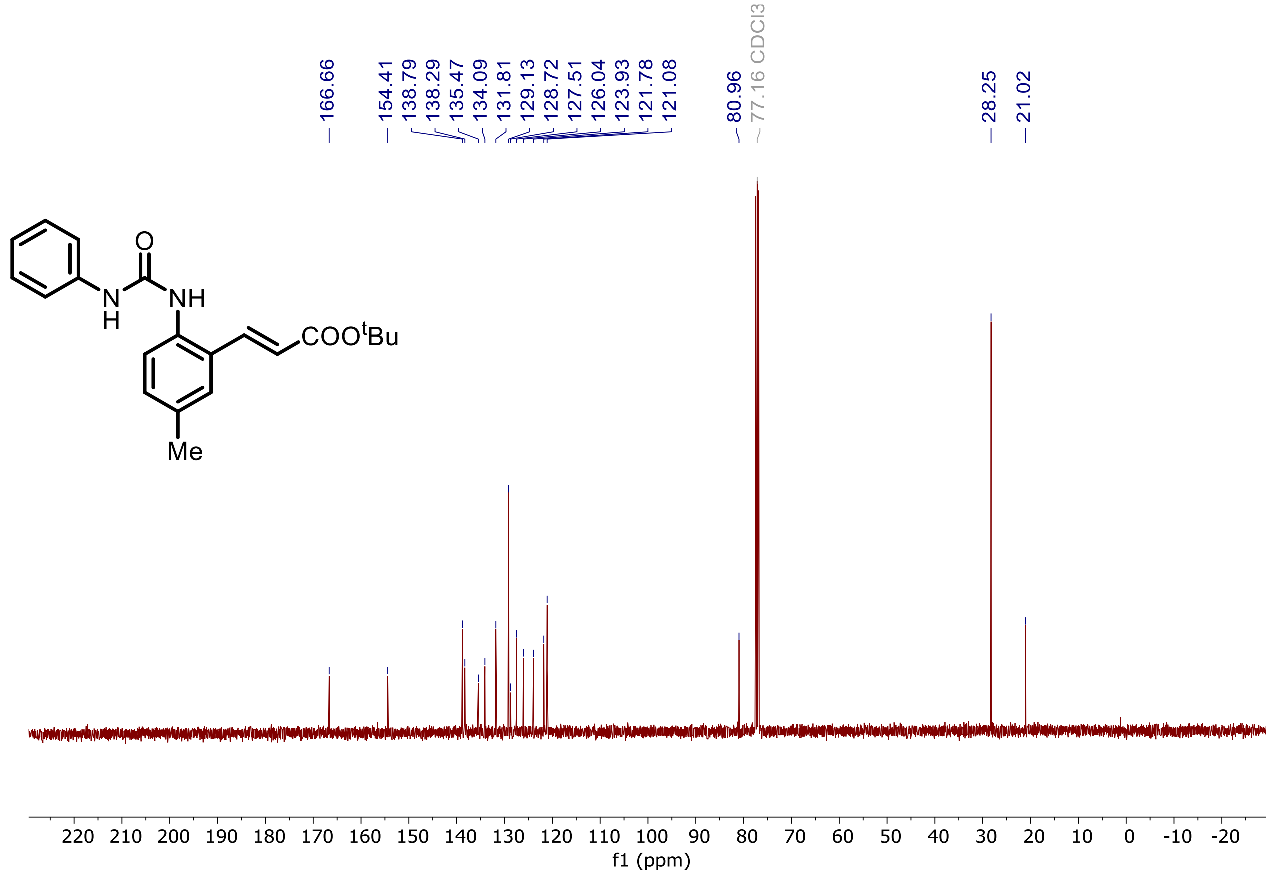

^{1}H NMR: (400 MHz, $CDCl_3$, 298K) of **1c**

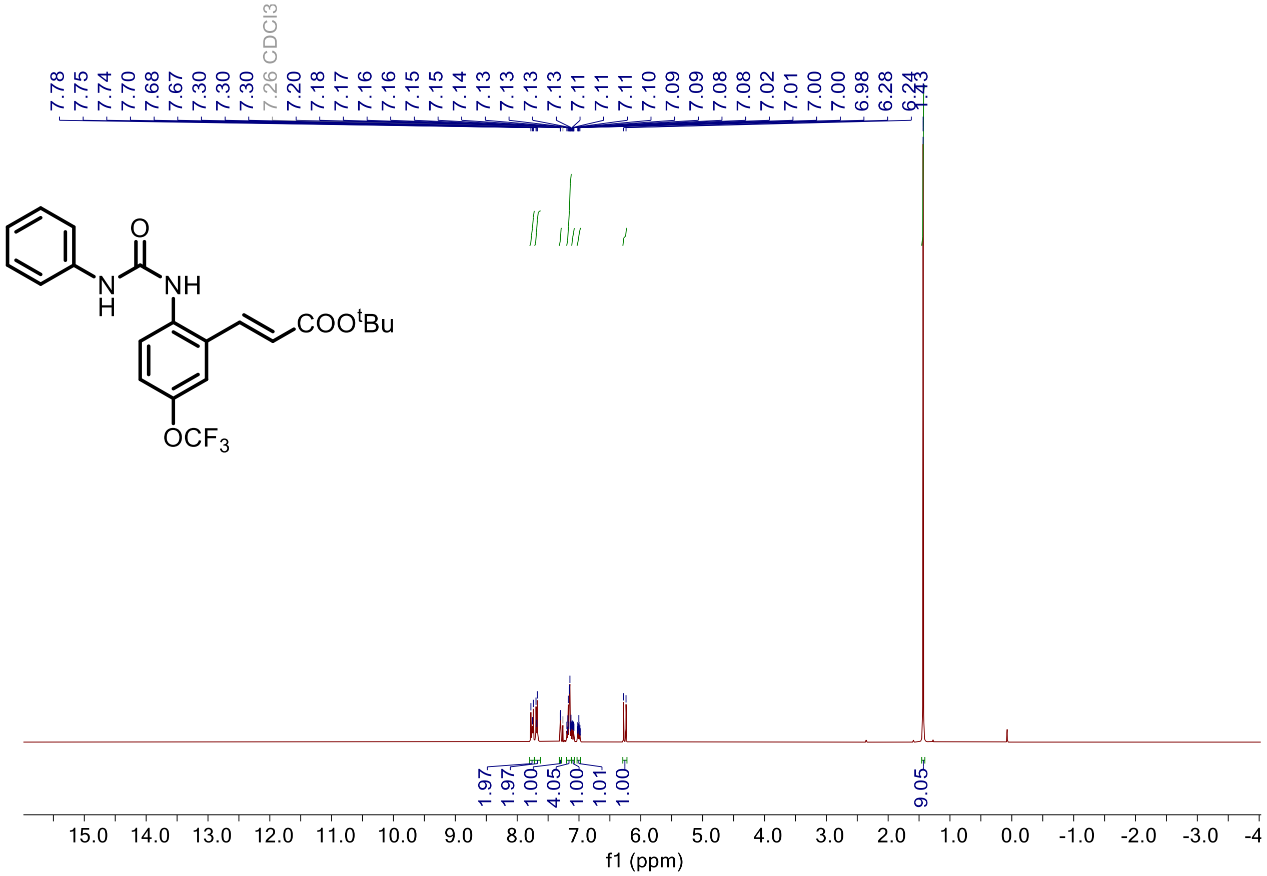

^{13}C NMR: (101 MHz, $CDCl_3$, 298K) of **1c**

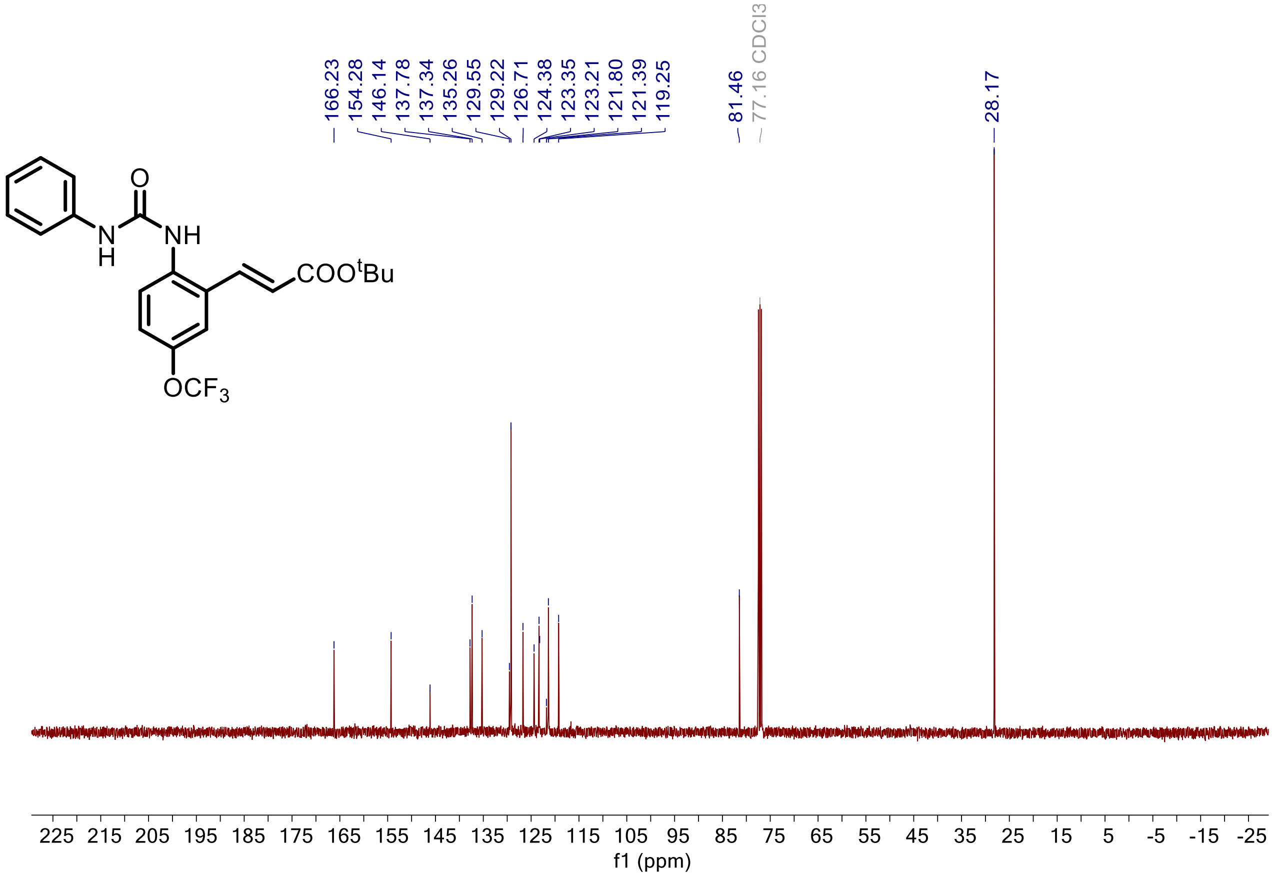

^{19}F NMR: (377 MHz, $CDCl_3$, 298K) of **1c**

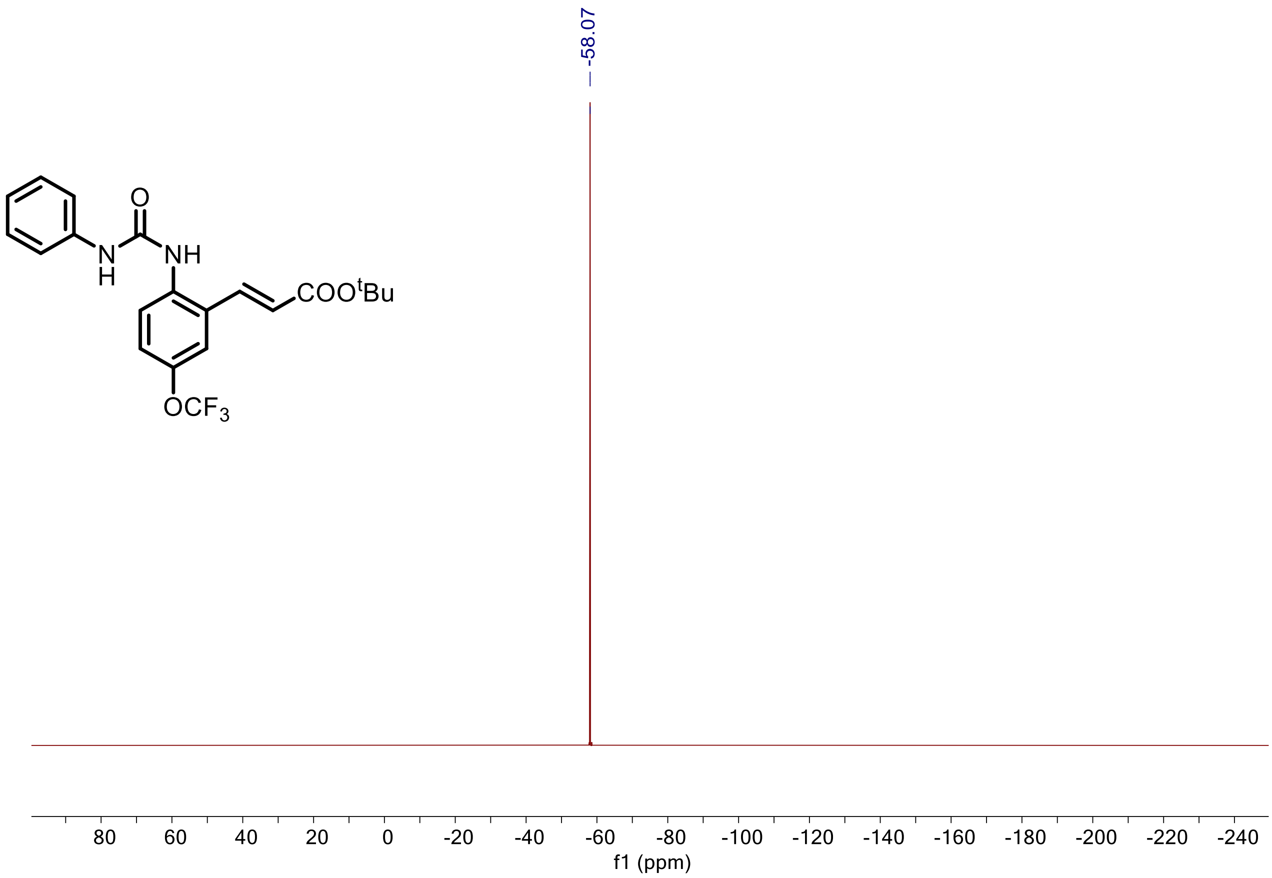

^{1}H NMR: (400 MHz, $CDCl_3$, 298K) of **1d**

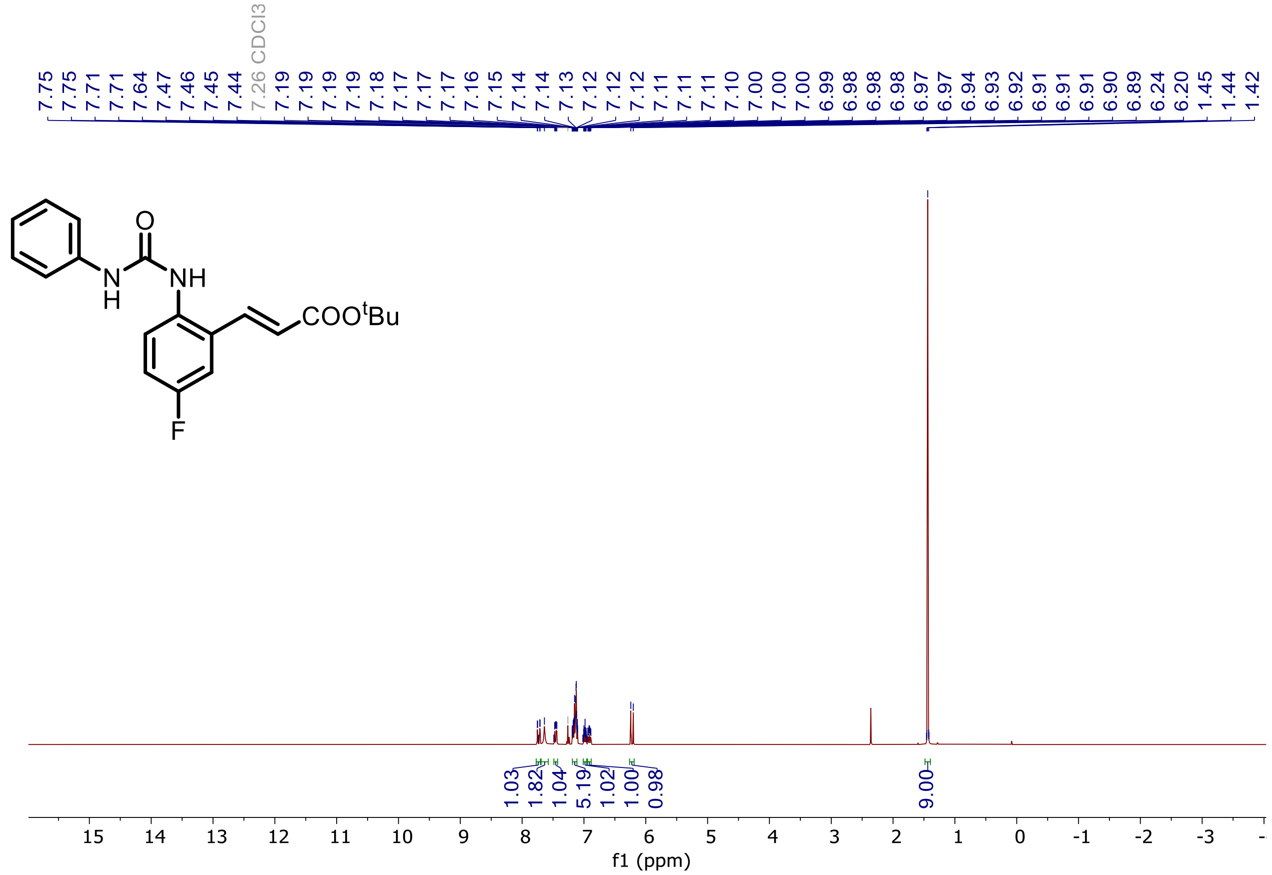

^{13}C NMR: (101 MHz, $CDCl_3$, 298K) of **1d**

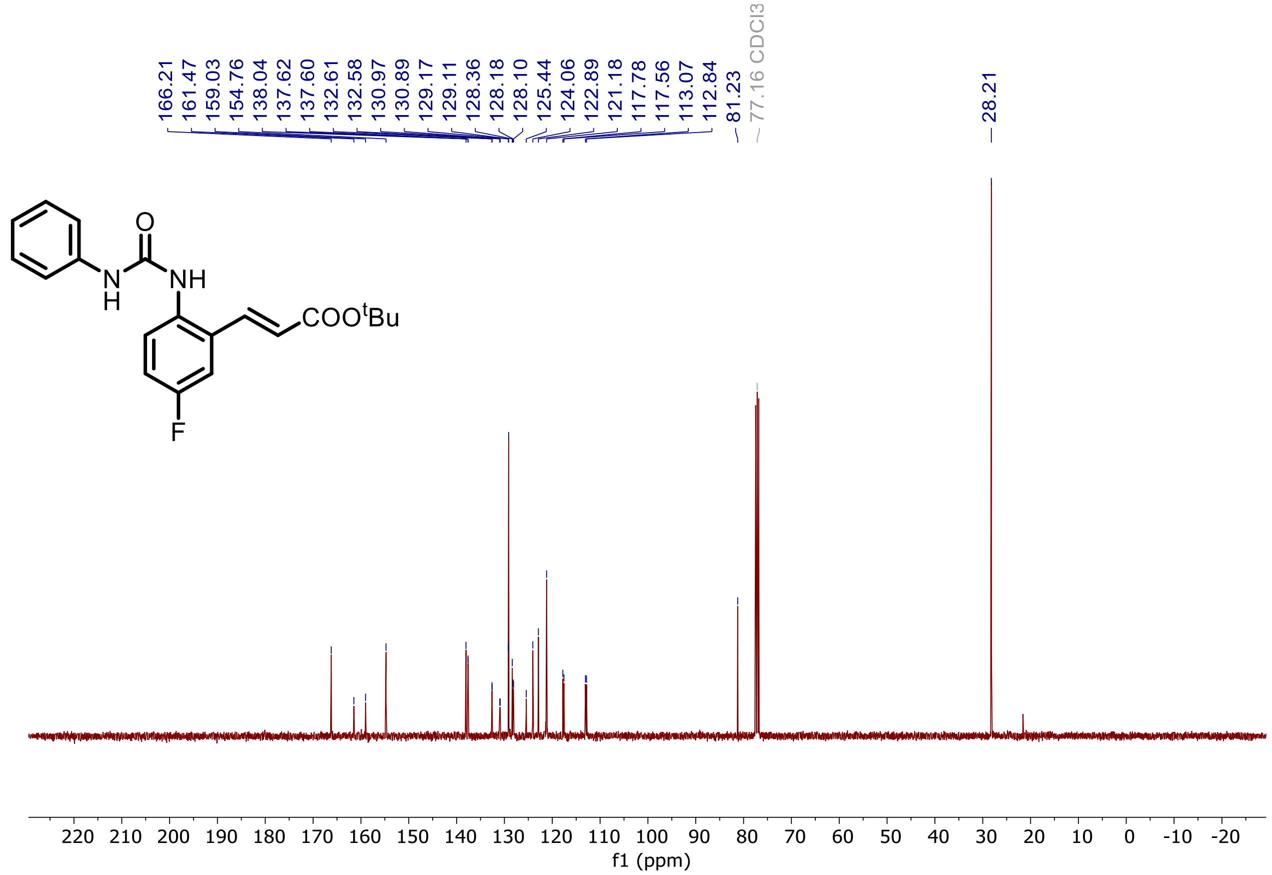

^{19}F NMR: (377 MHz, $CDCl_3$, 298K) of **1d**

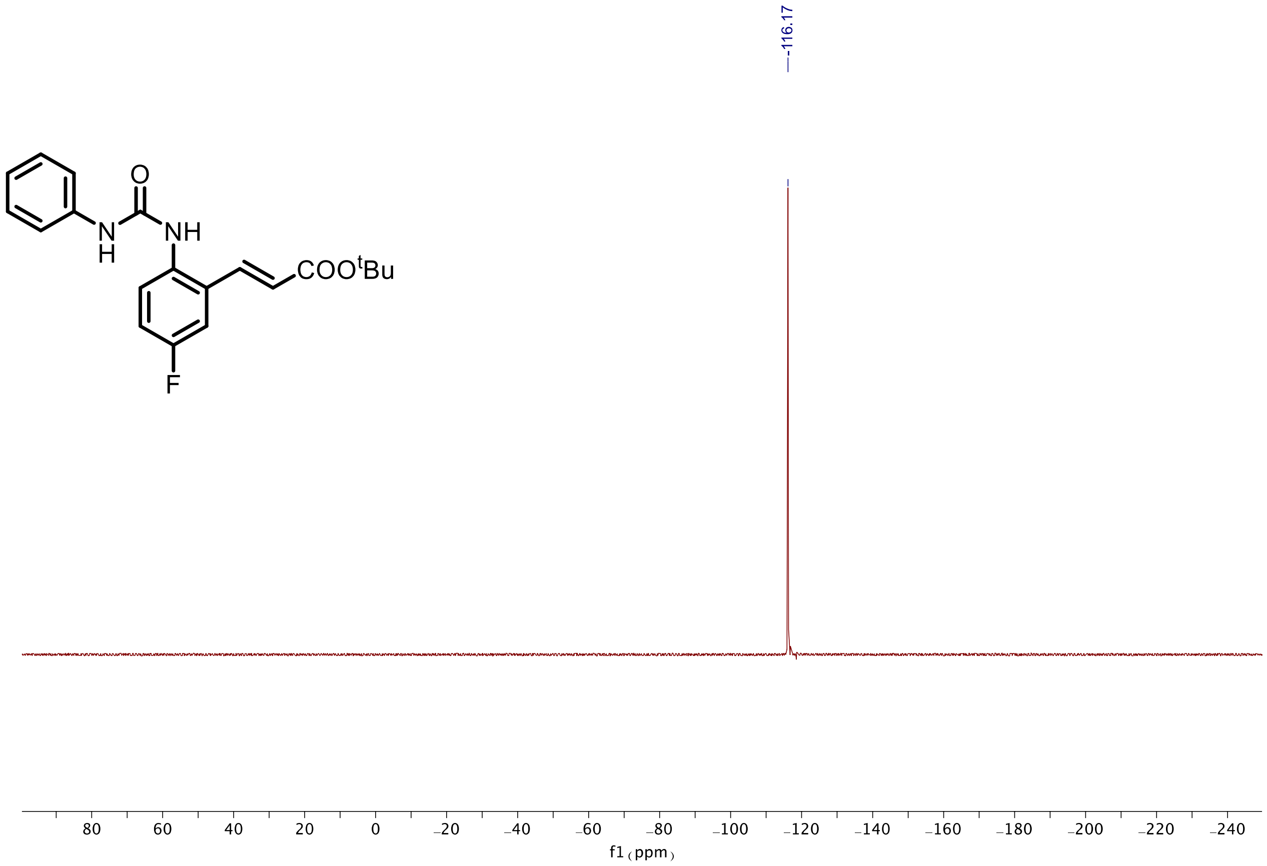

^{1}H NMR: (400 MHz, $CDCl_3$, 298K) of **1e**

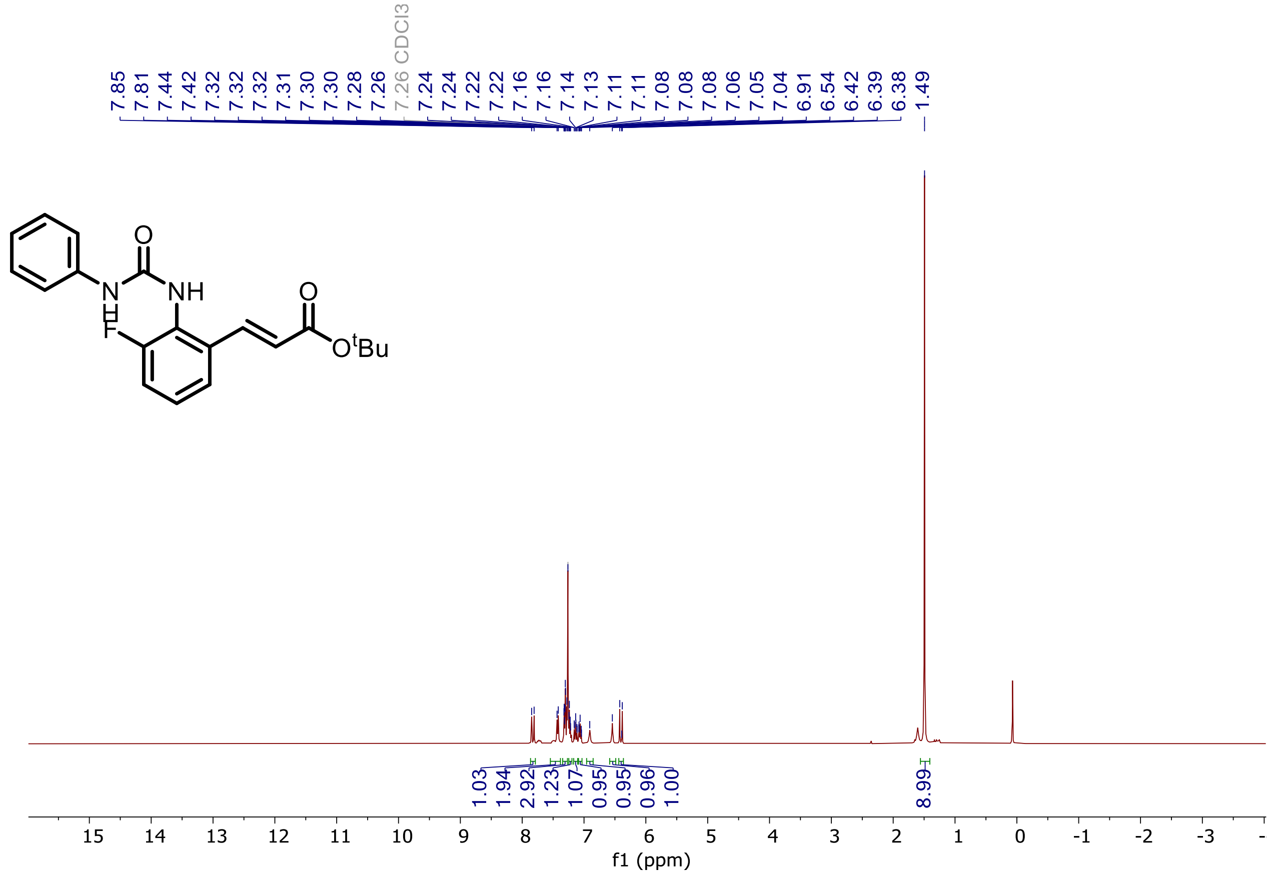

^{19}F NMR: (377 MHz, $CDCl_3$, 298K) of **1e**

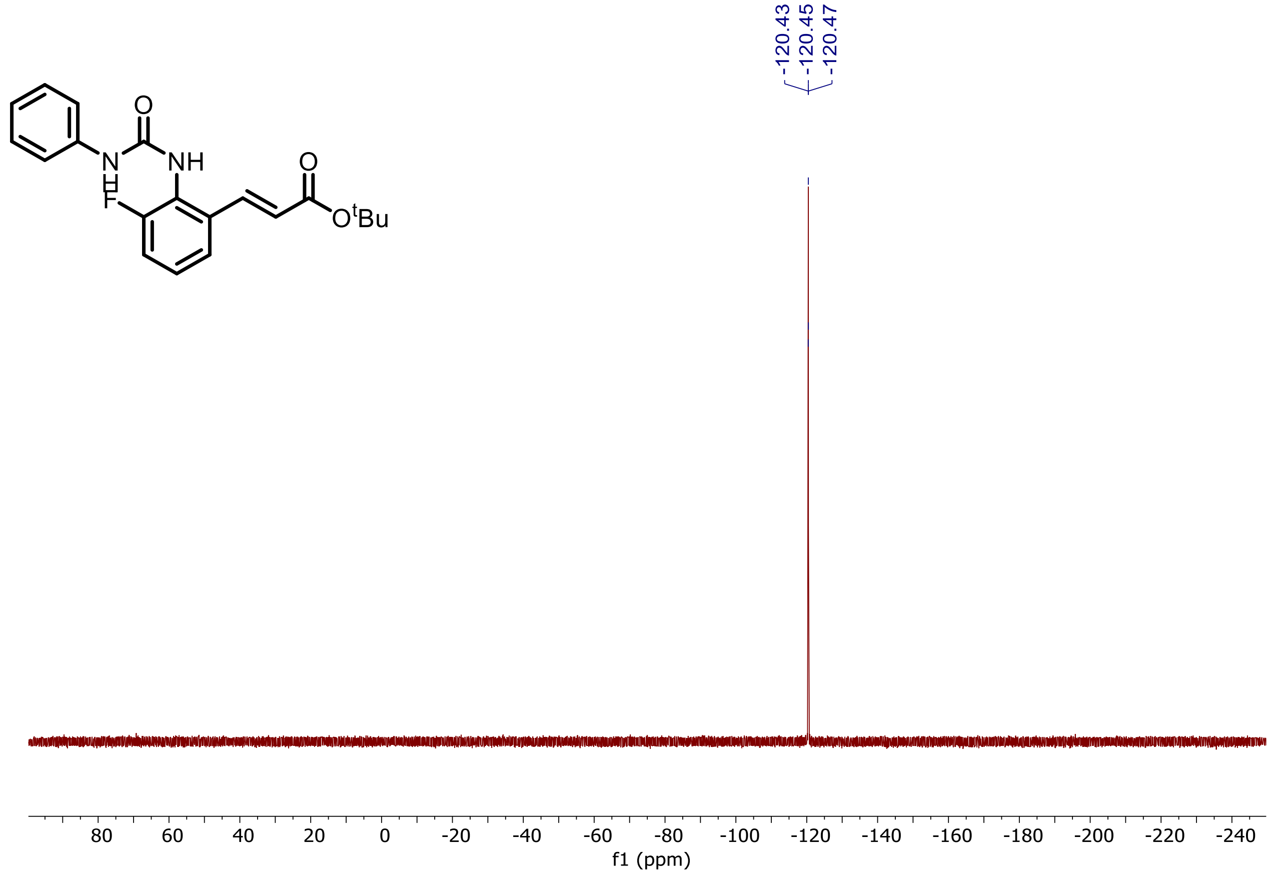

^{1}H NMR: (400 MHz, $CDCl_3$, 298K) of **1f**

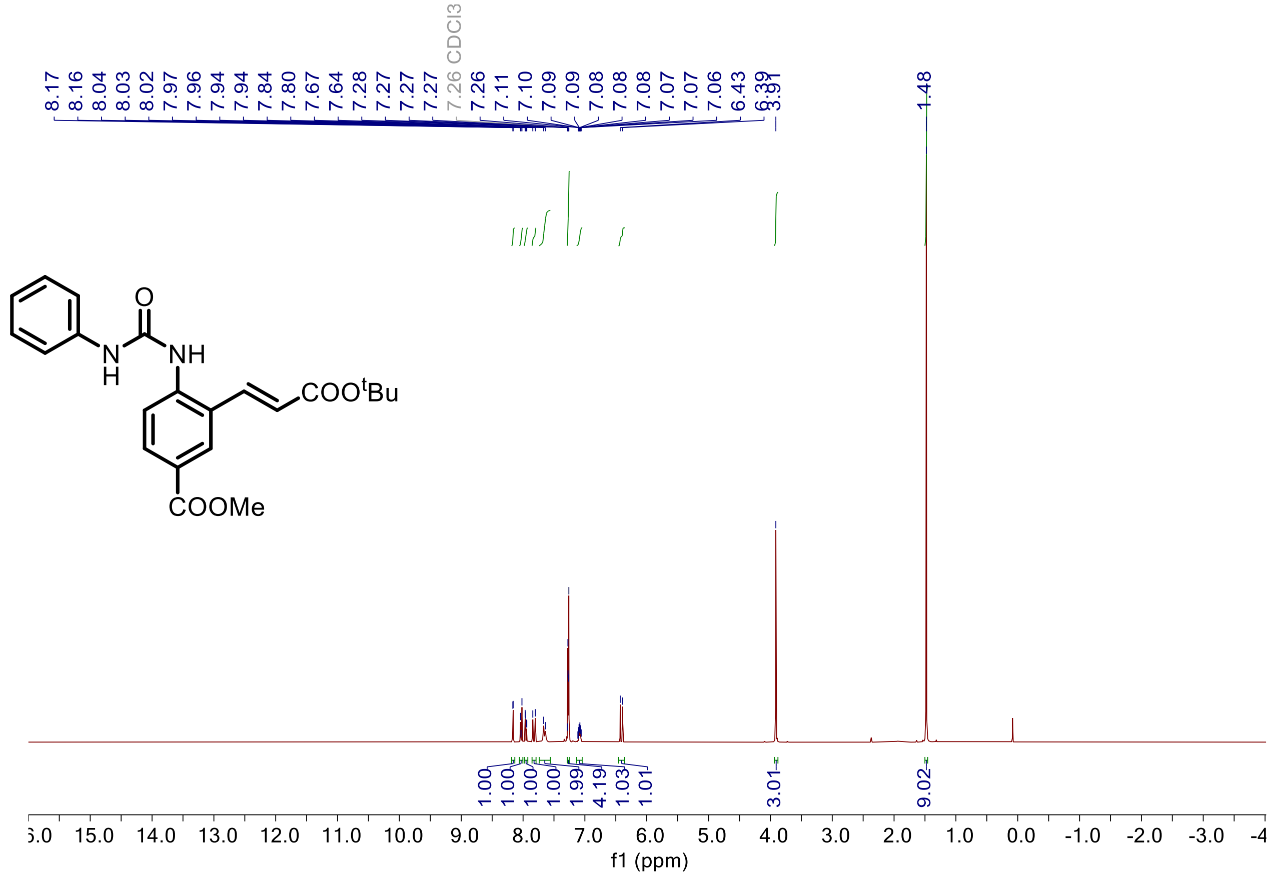

^{13}C NMR: (101 MHz, $CDCl_3$, 298K) of **1f**

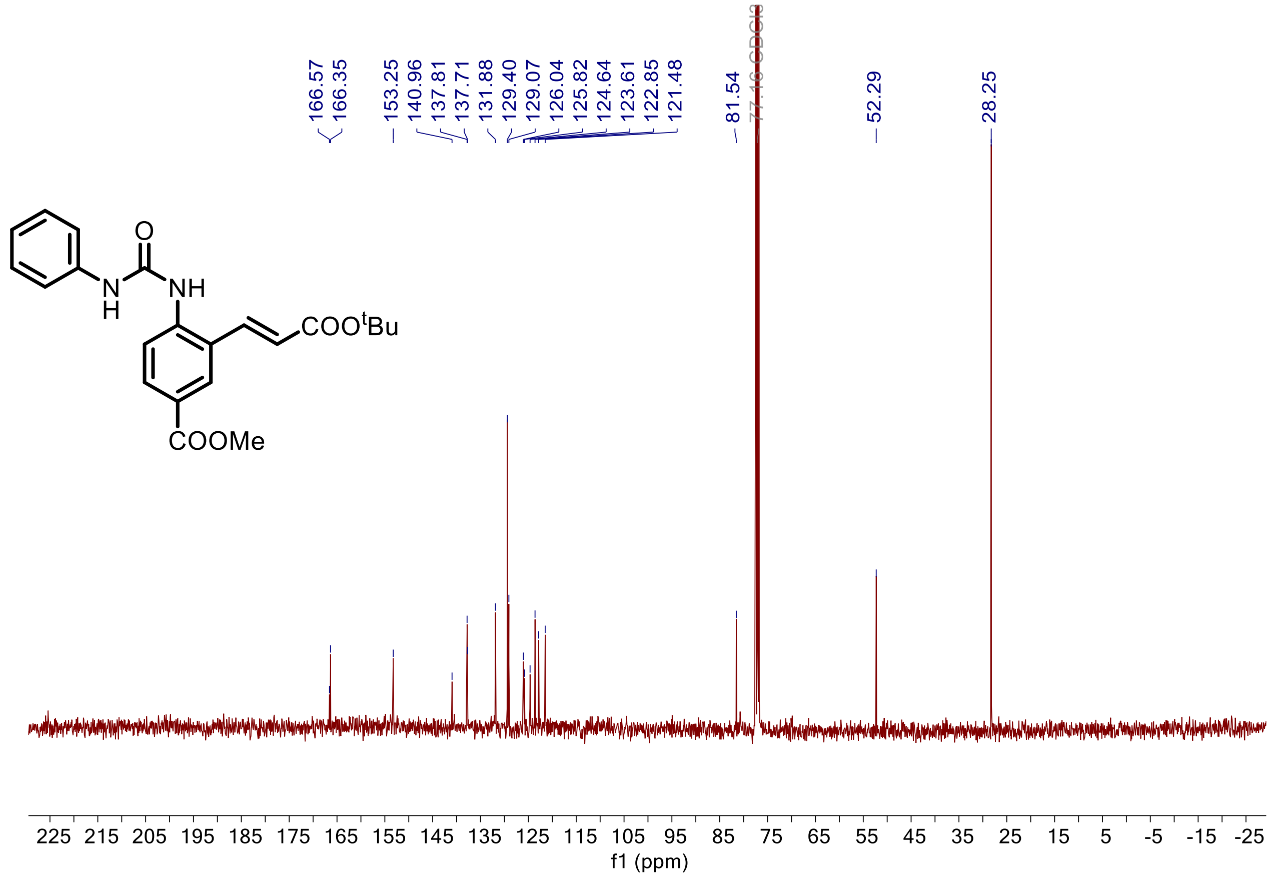

^{1}H NMR: (400 MHz, $CDCl_3$, 298K) of **1g**

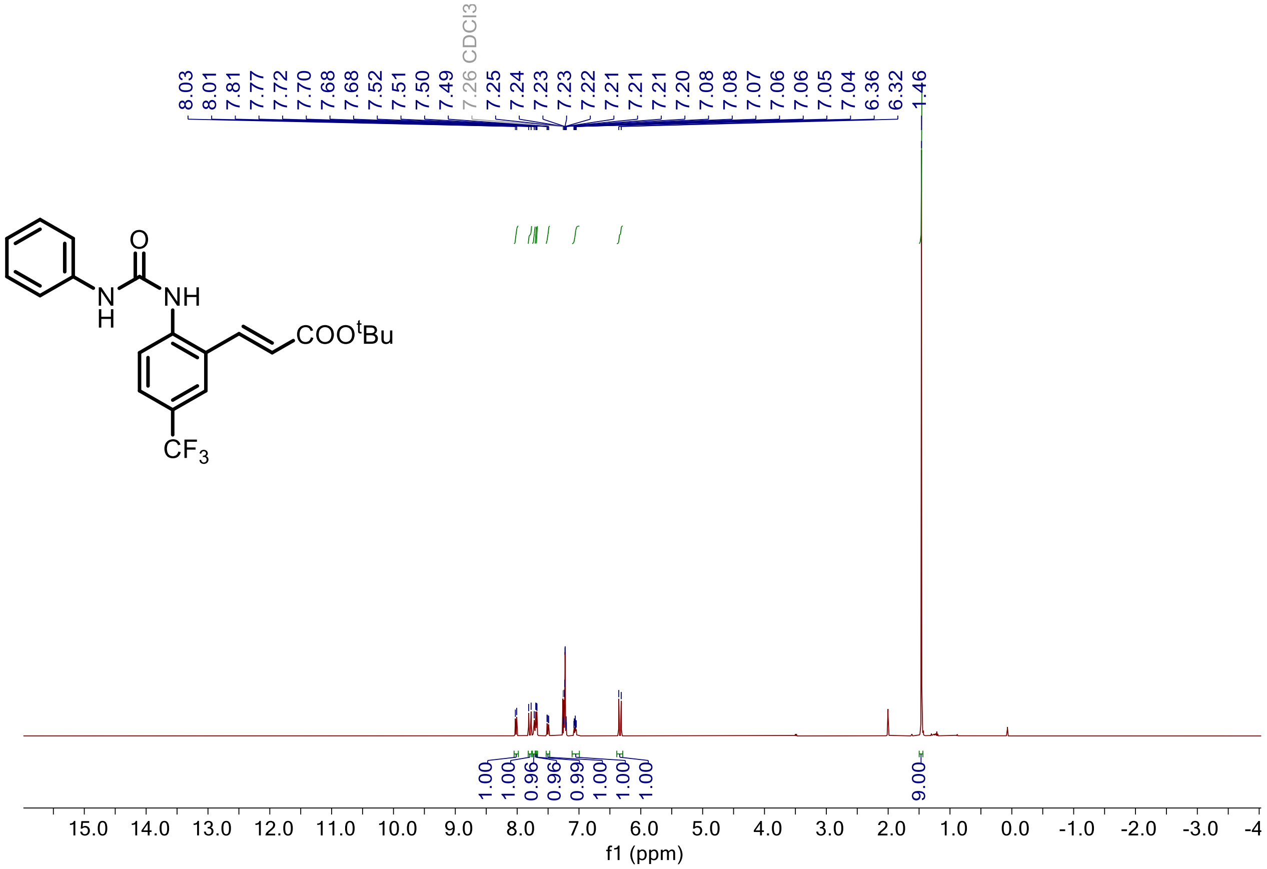

^{13}C NMR: (101 MHz, $CDCl_3$, 298K) of **1g**

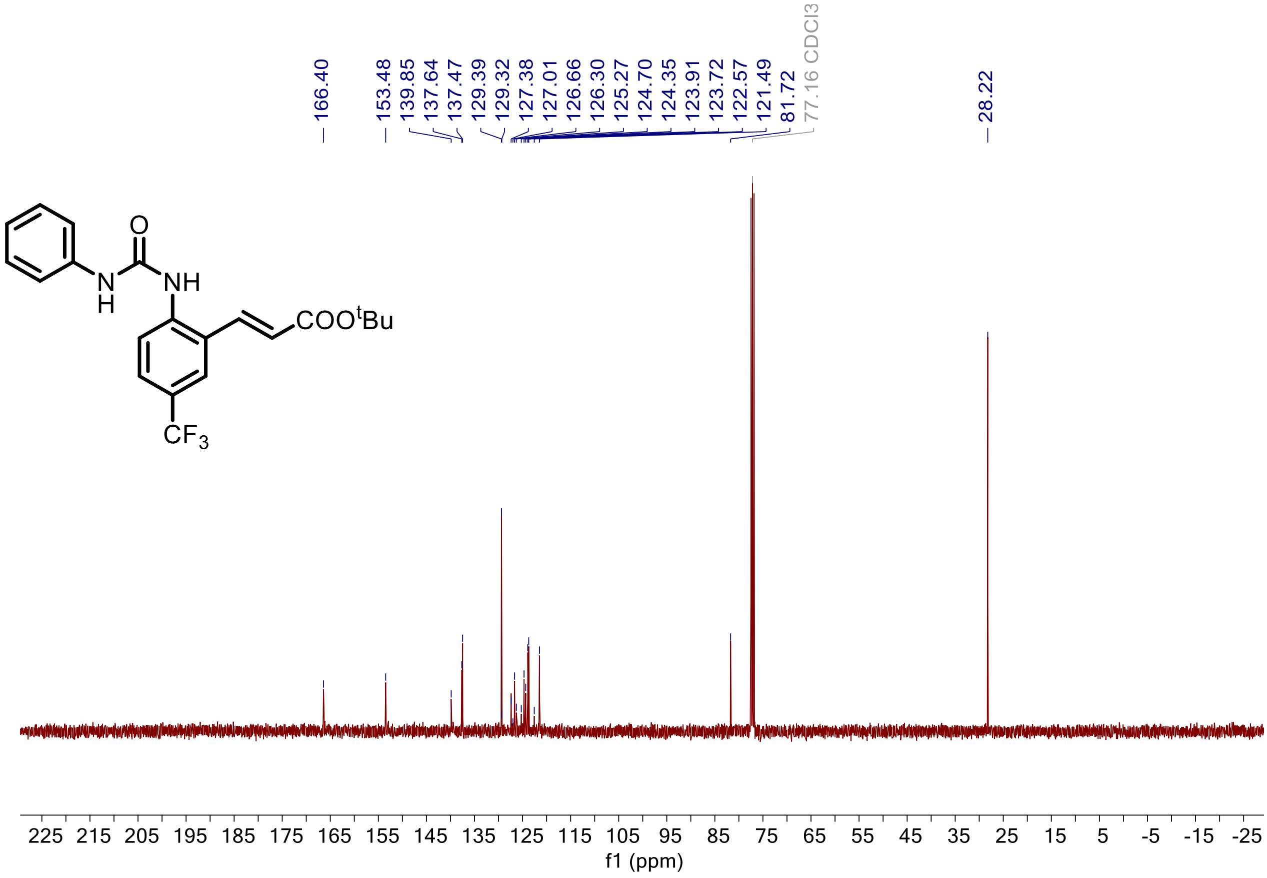

^{19}F NMR: (377 MHz, $CDCl_3$, 298K) of **1g**

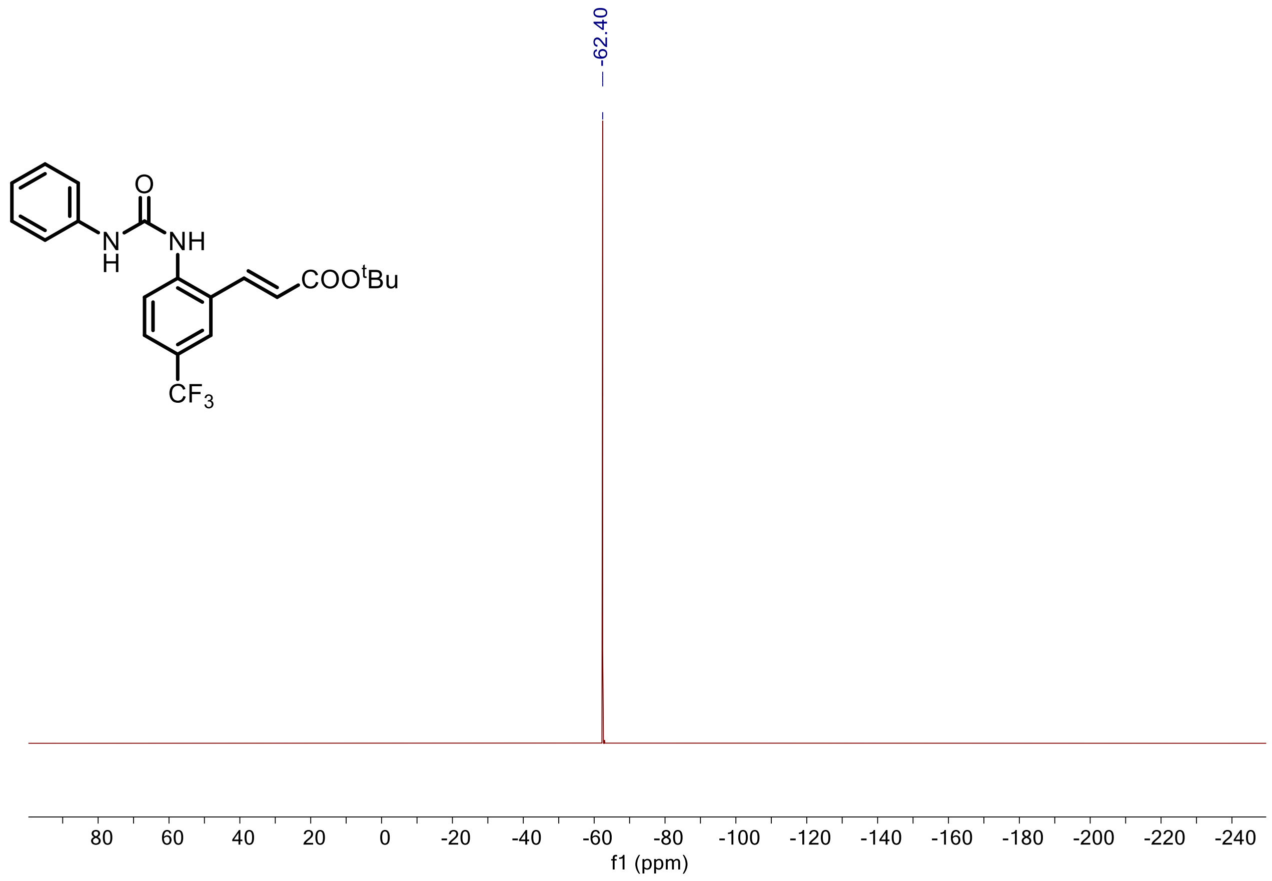

^{1}H NMR: (400 MHz, $CDCl_3$, 298K) of **1h**

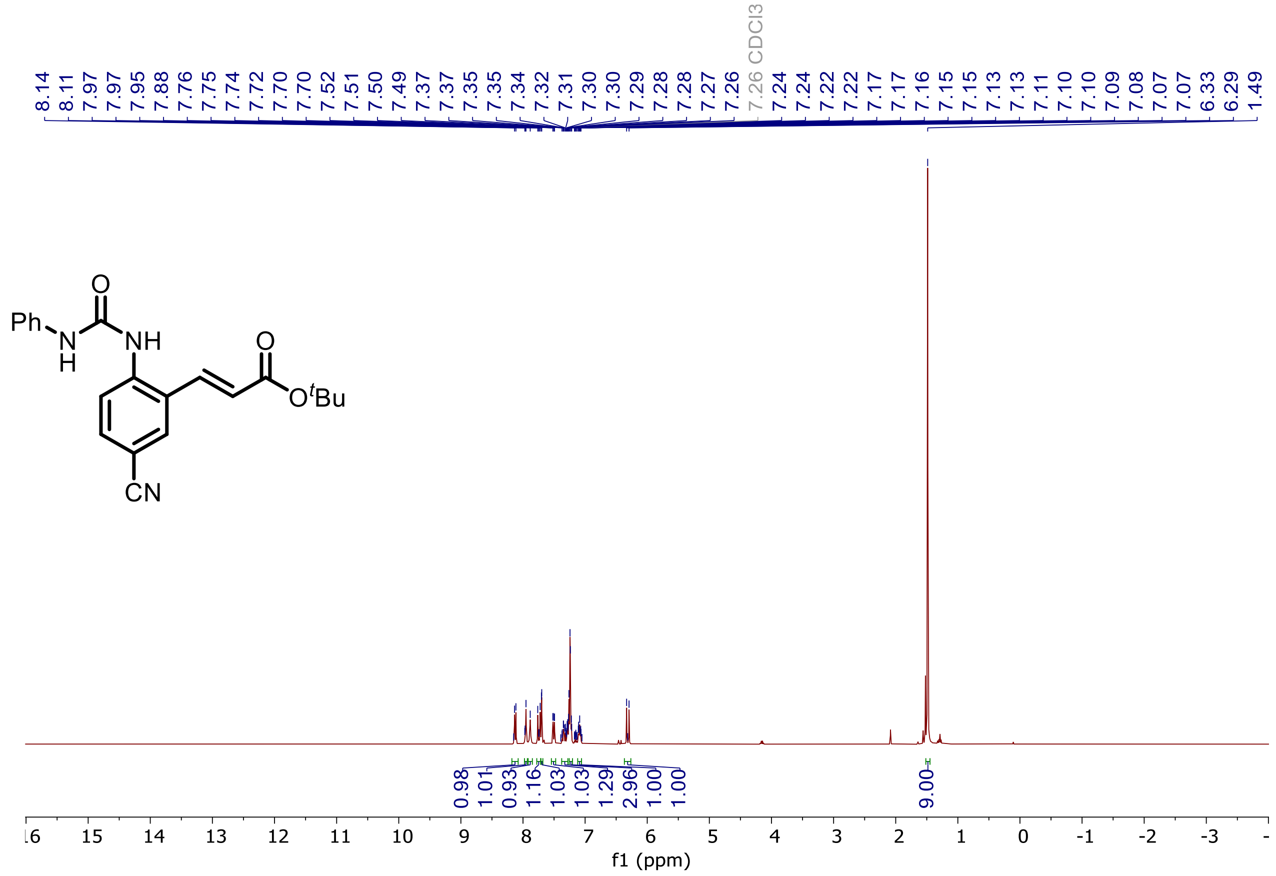

^{13}C NMR: (101 MHz, $CDCl_3$, 298K) of **1h**

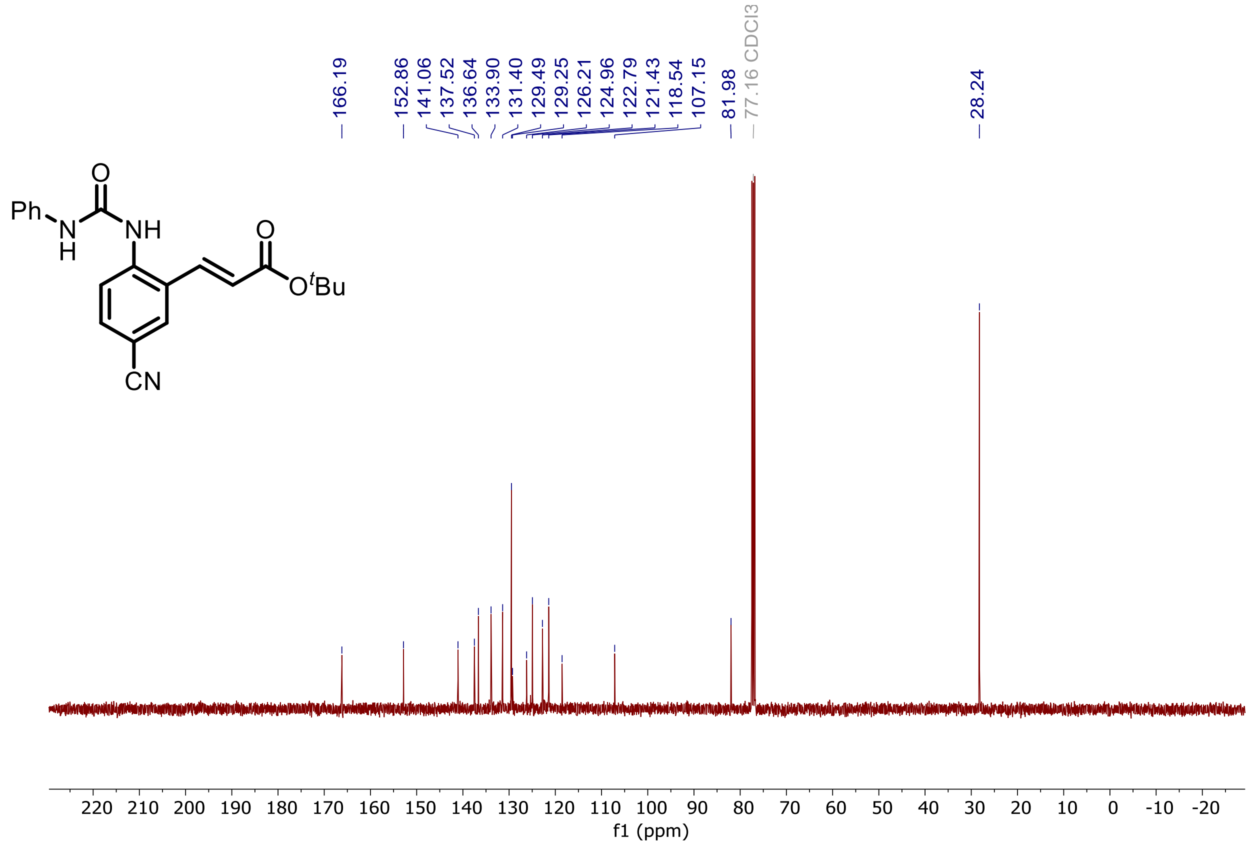

^{1}H NMR: (400 MHz, $CDCl_3$, 298K) of **1i**

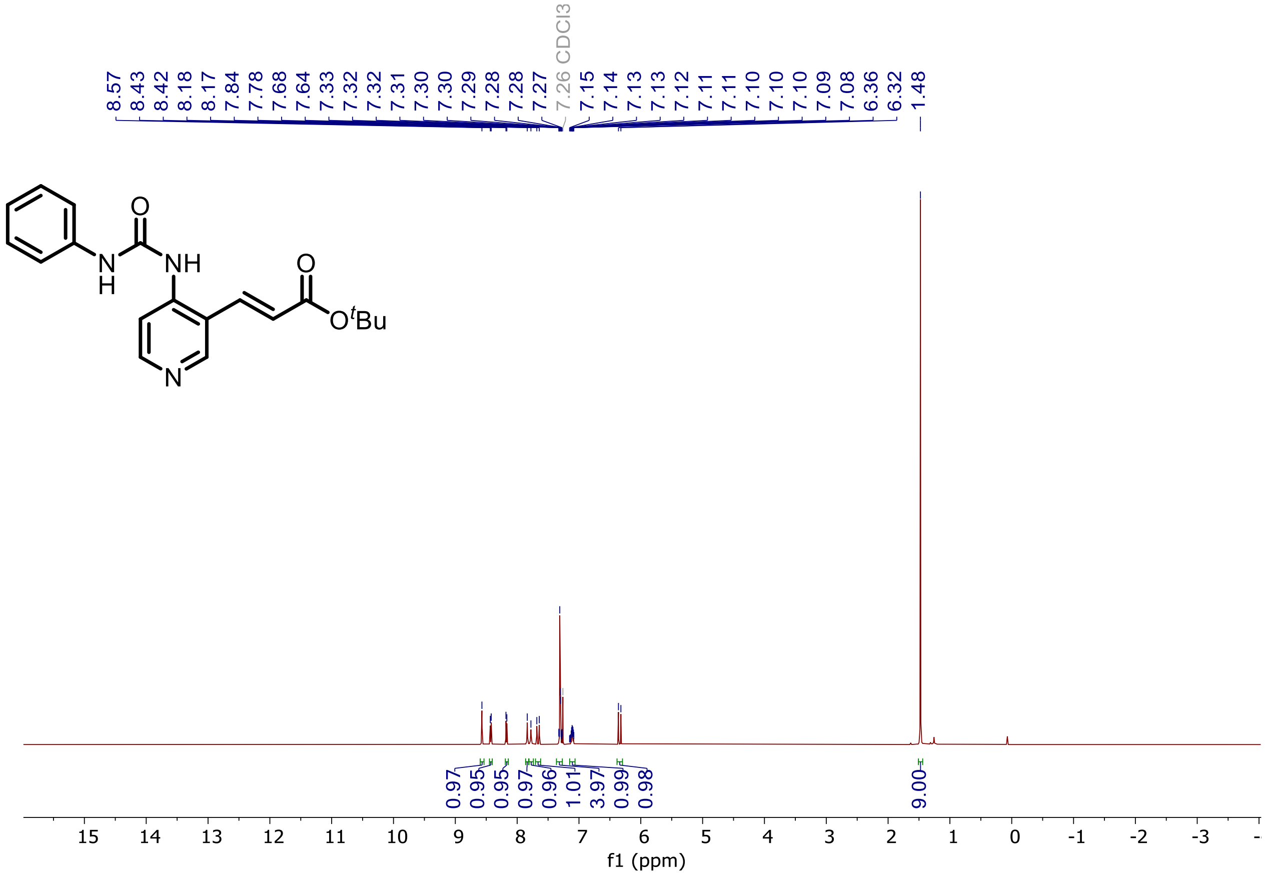

^{13}C NMR: (101 MHz, $CDCl_3$, 298K) of **1i**

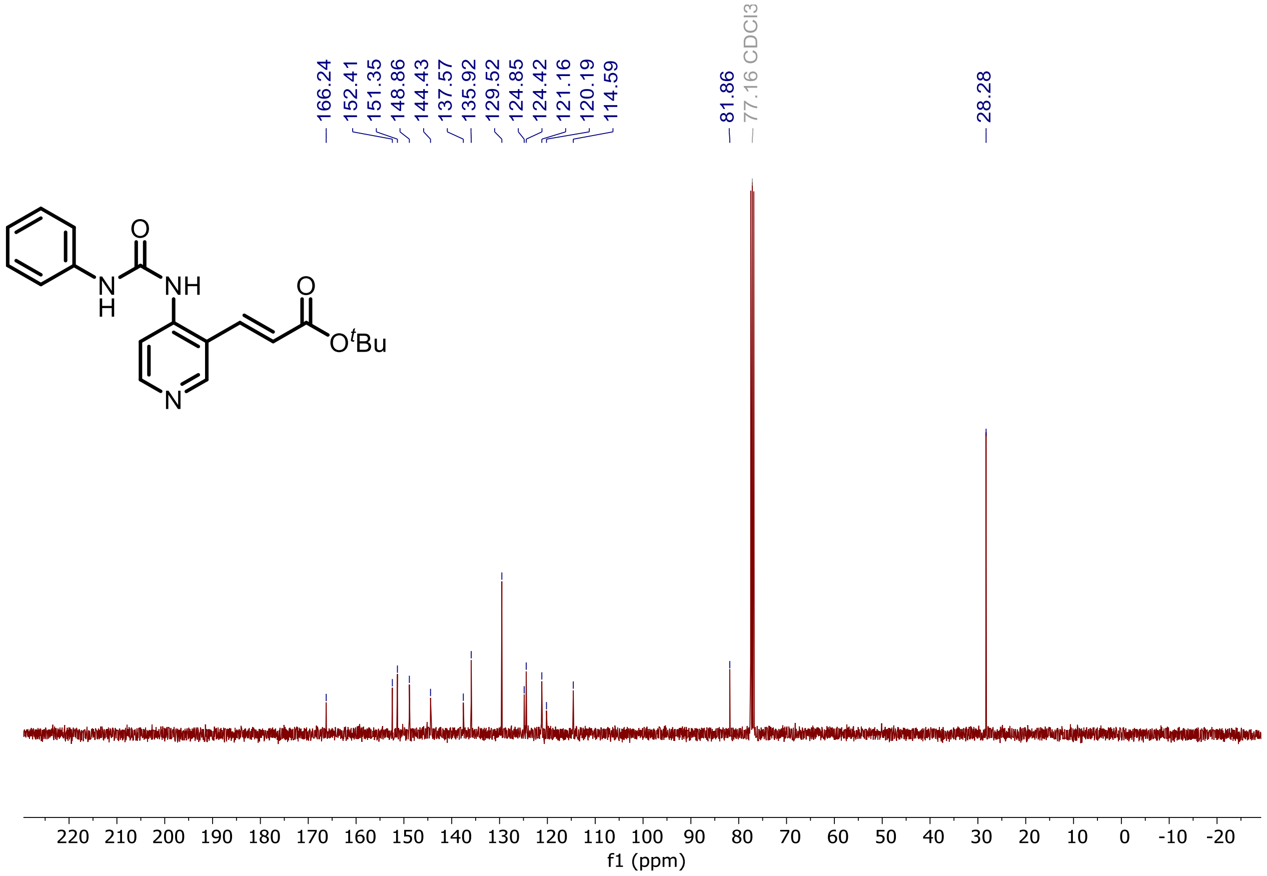

^{1}H NMR: (400 MHz, $CDCl_3$, 298K) of **1j**

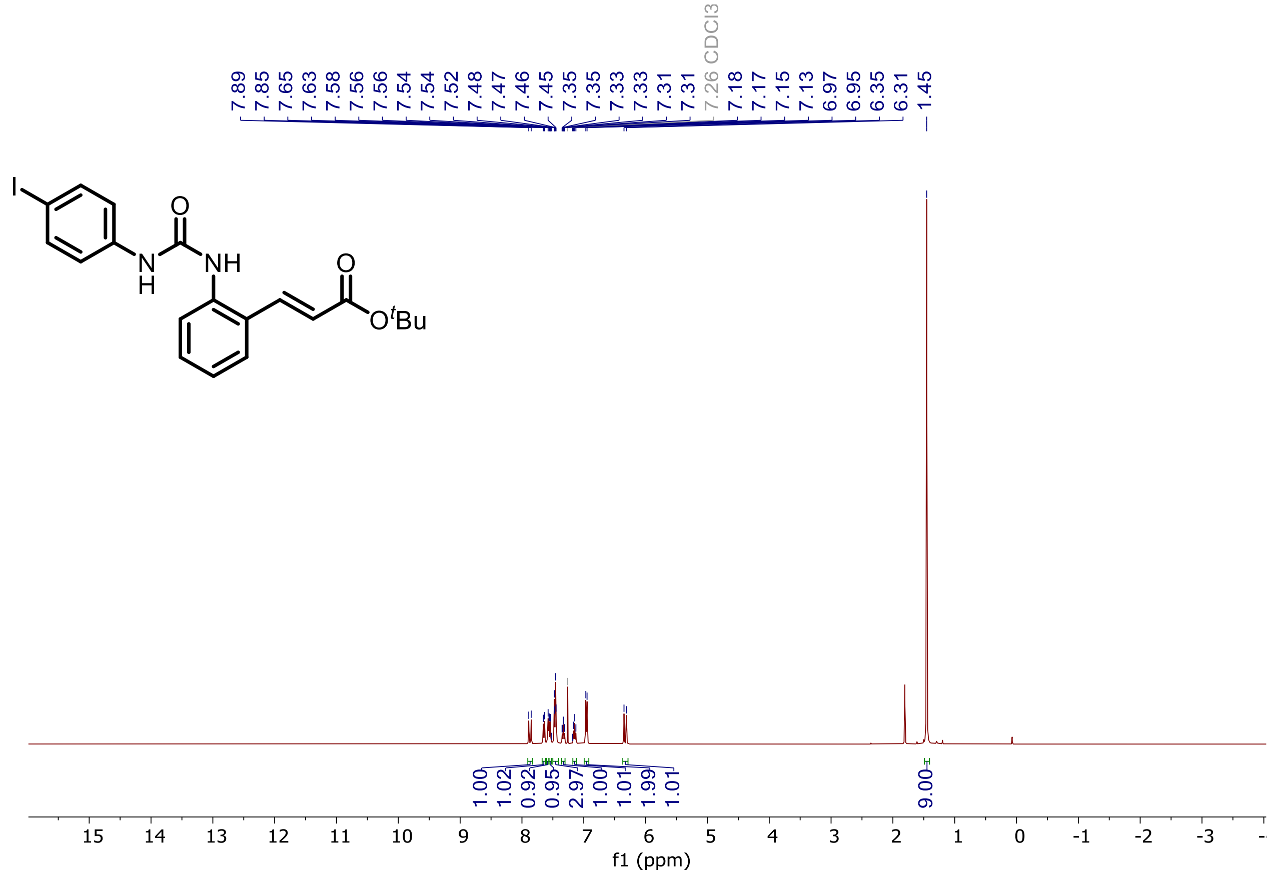

^{13}C NMR: (101 MHz, $CDCl_3$, 298K) of **1j**

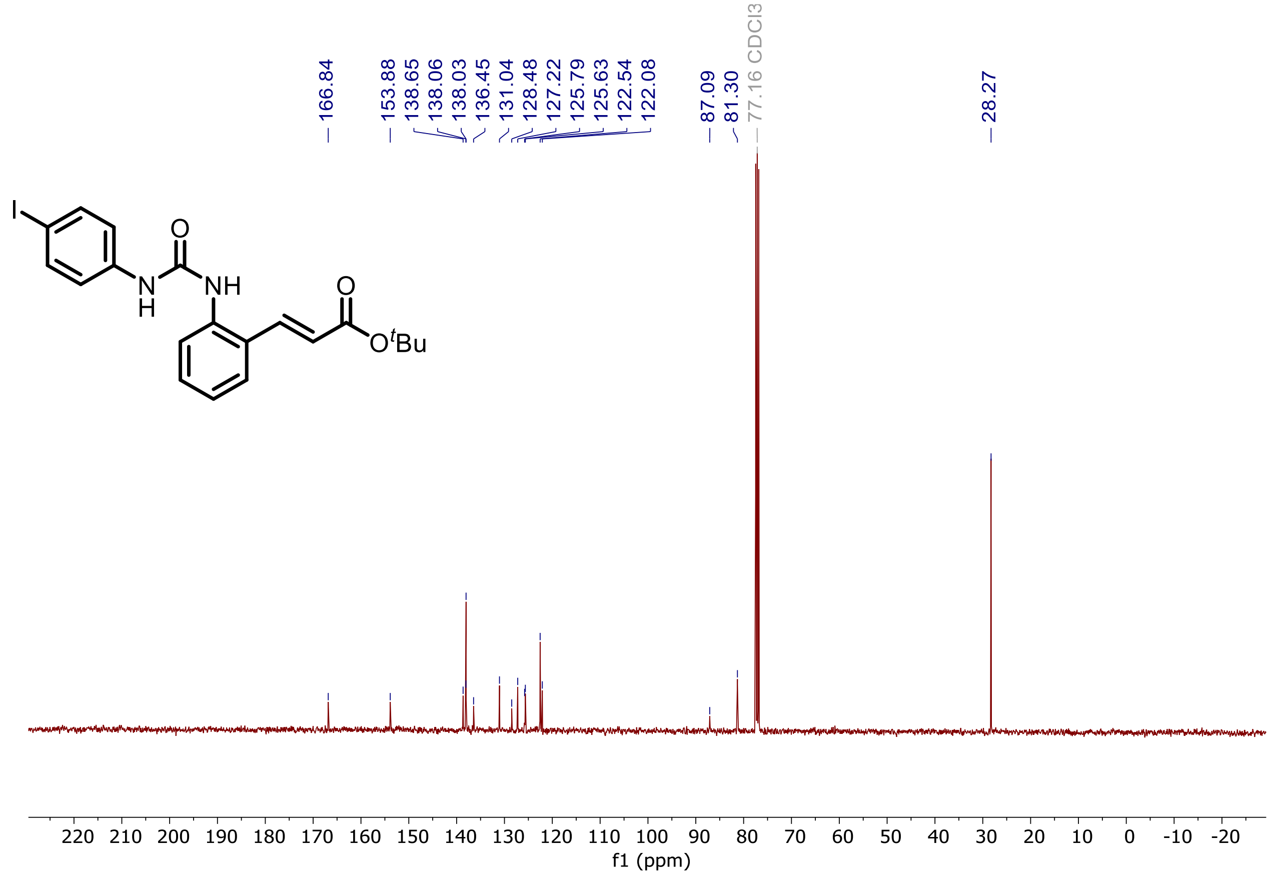

^{1}H NMR: (400 MHz, $CDCl_3$, 298K) of **1k**

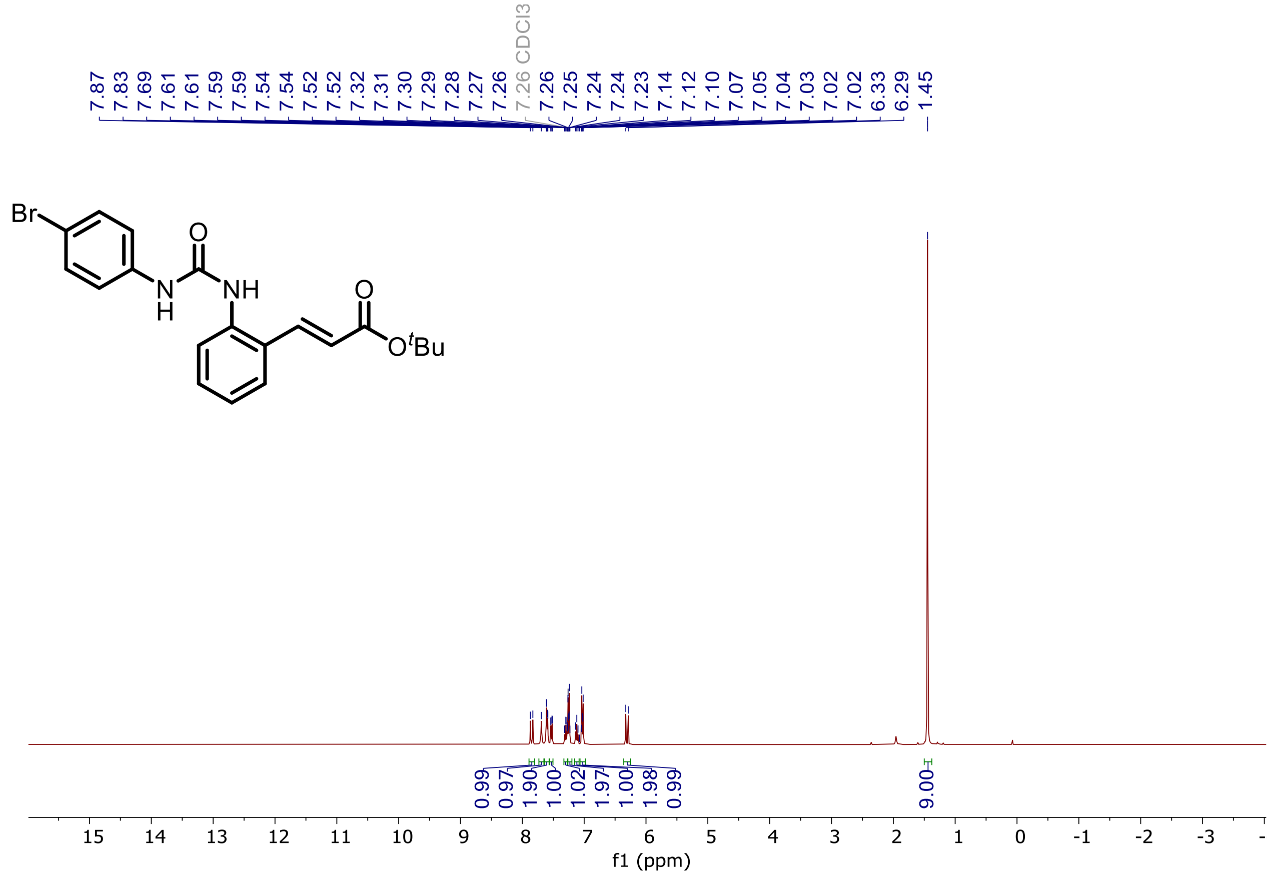

^{13}C NMR: (101 MHz, $CDCl_3$, 298K) of **1k**

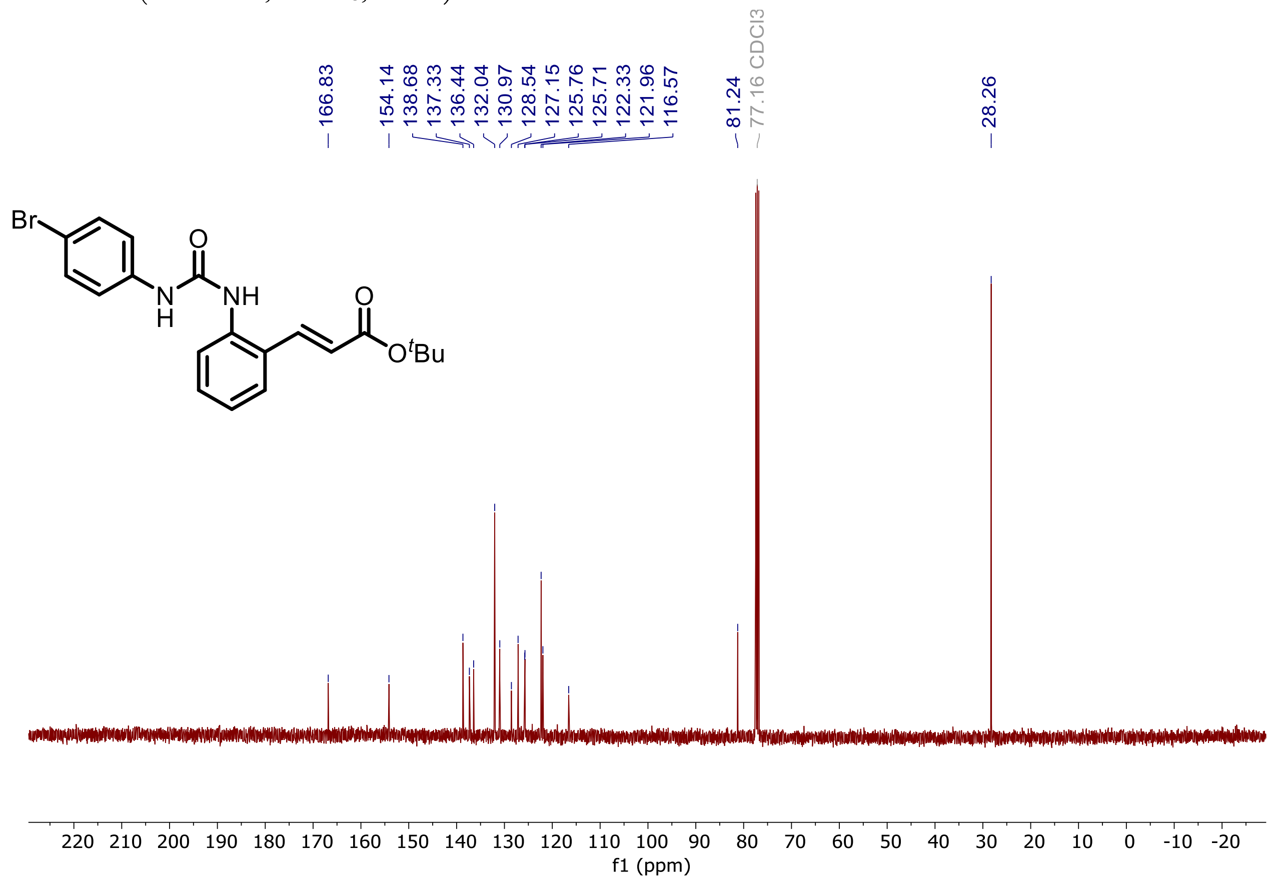

^{1}H NMR: (400 MHz, $CDCl_3$, 298K) of **11**

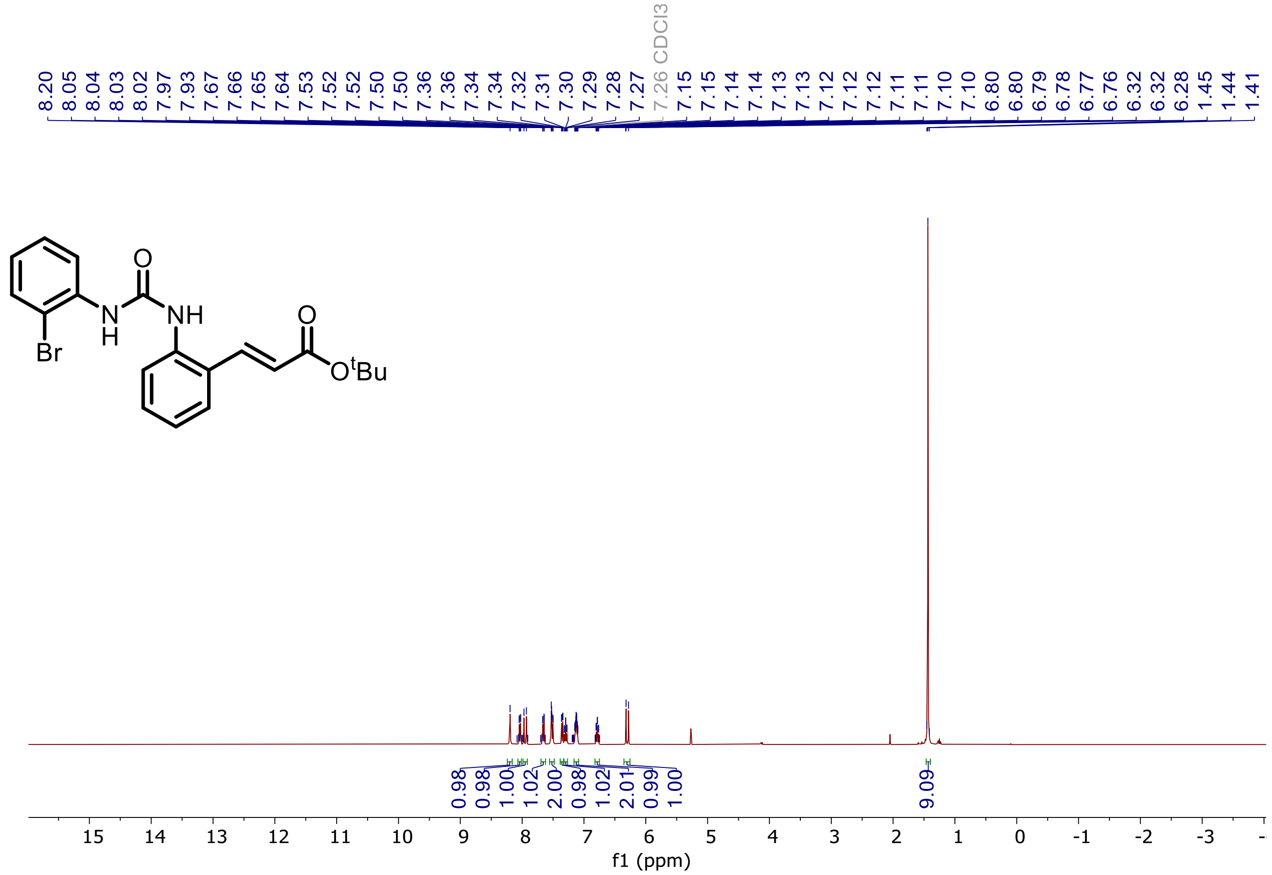

^{13}C NMR: (101 MHz, $CDCl_3$, 298K) of **11**

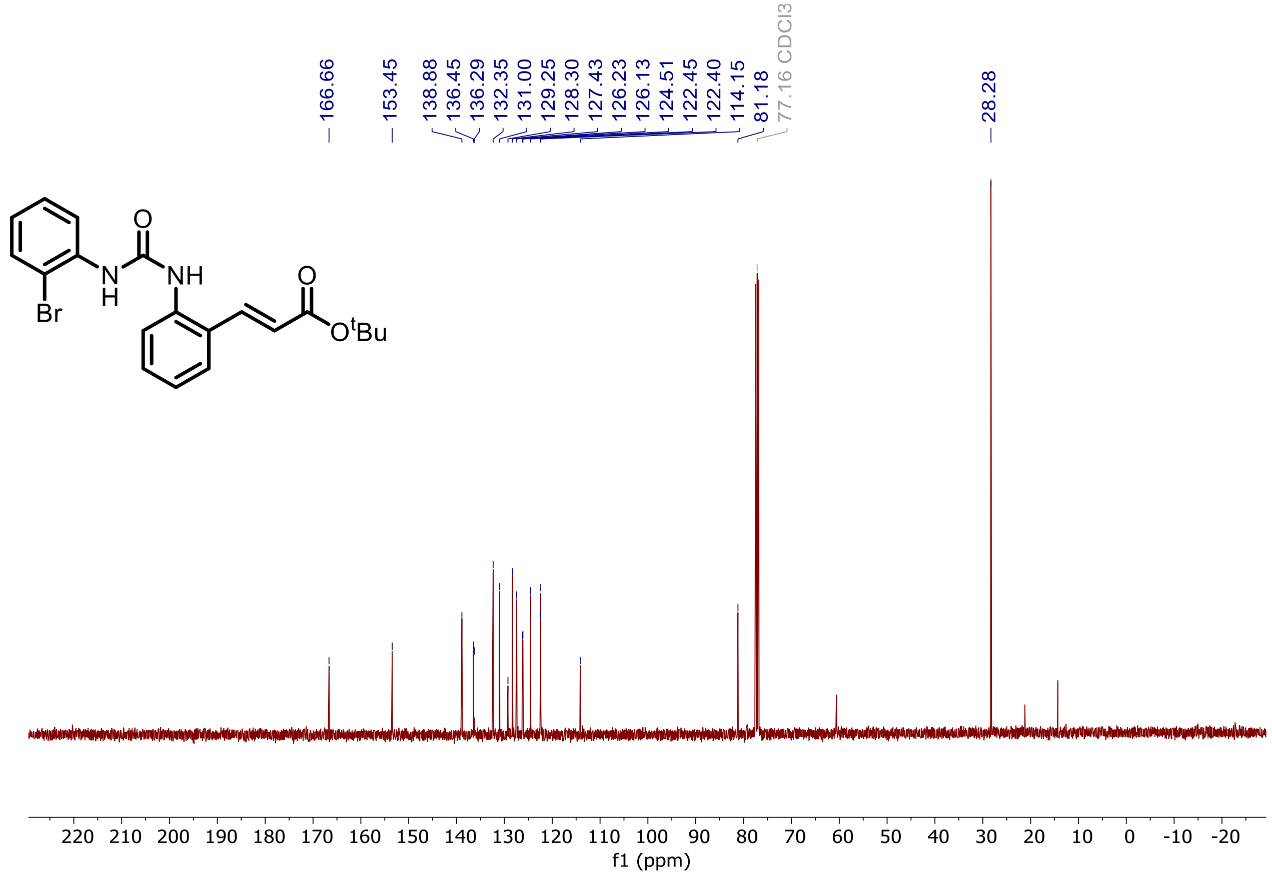

^{1}H NMR: (400 MHz, $CDCl_3$, 298K) of **1m**

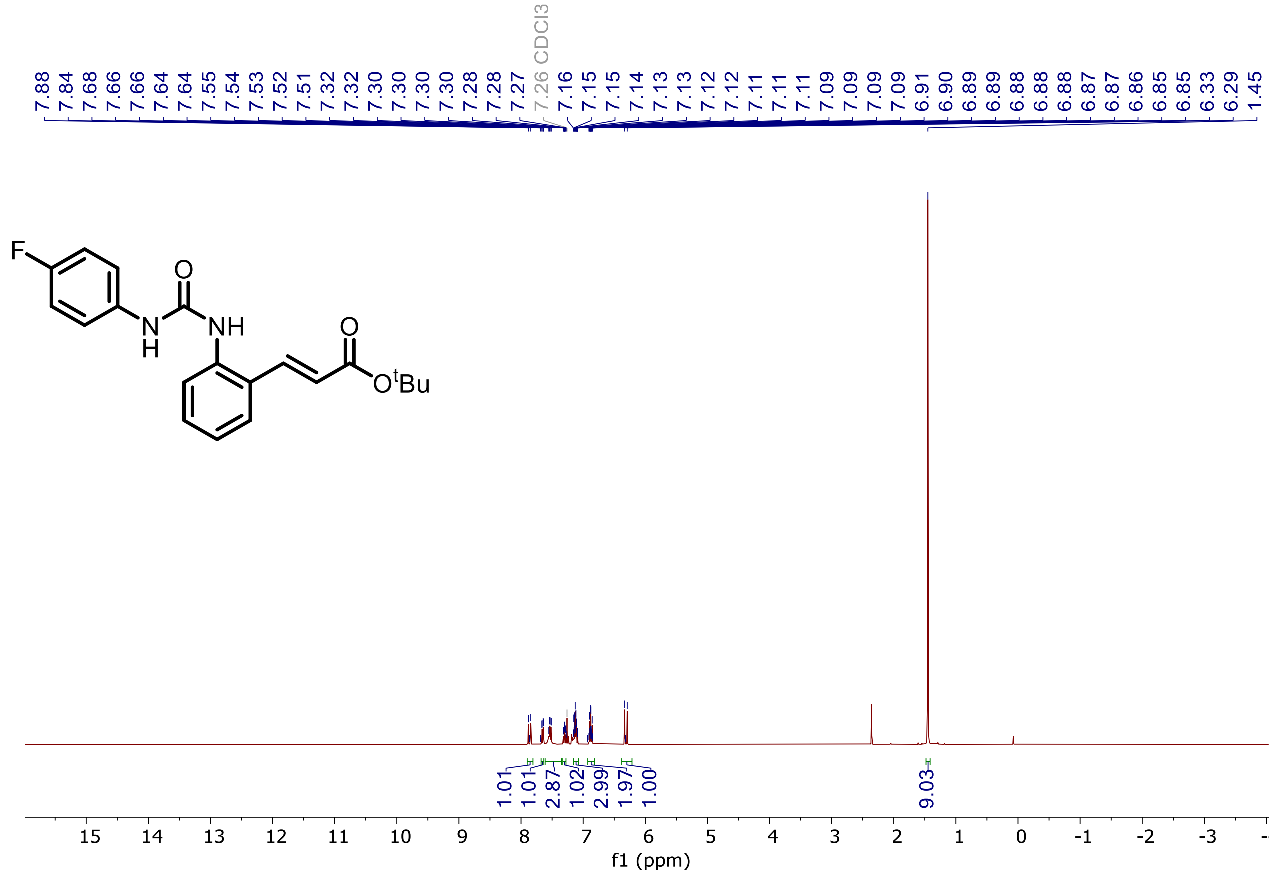

^{13}C NMR: (101 MHz, $CDCl_3$, 298K) of **1m**

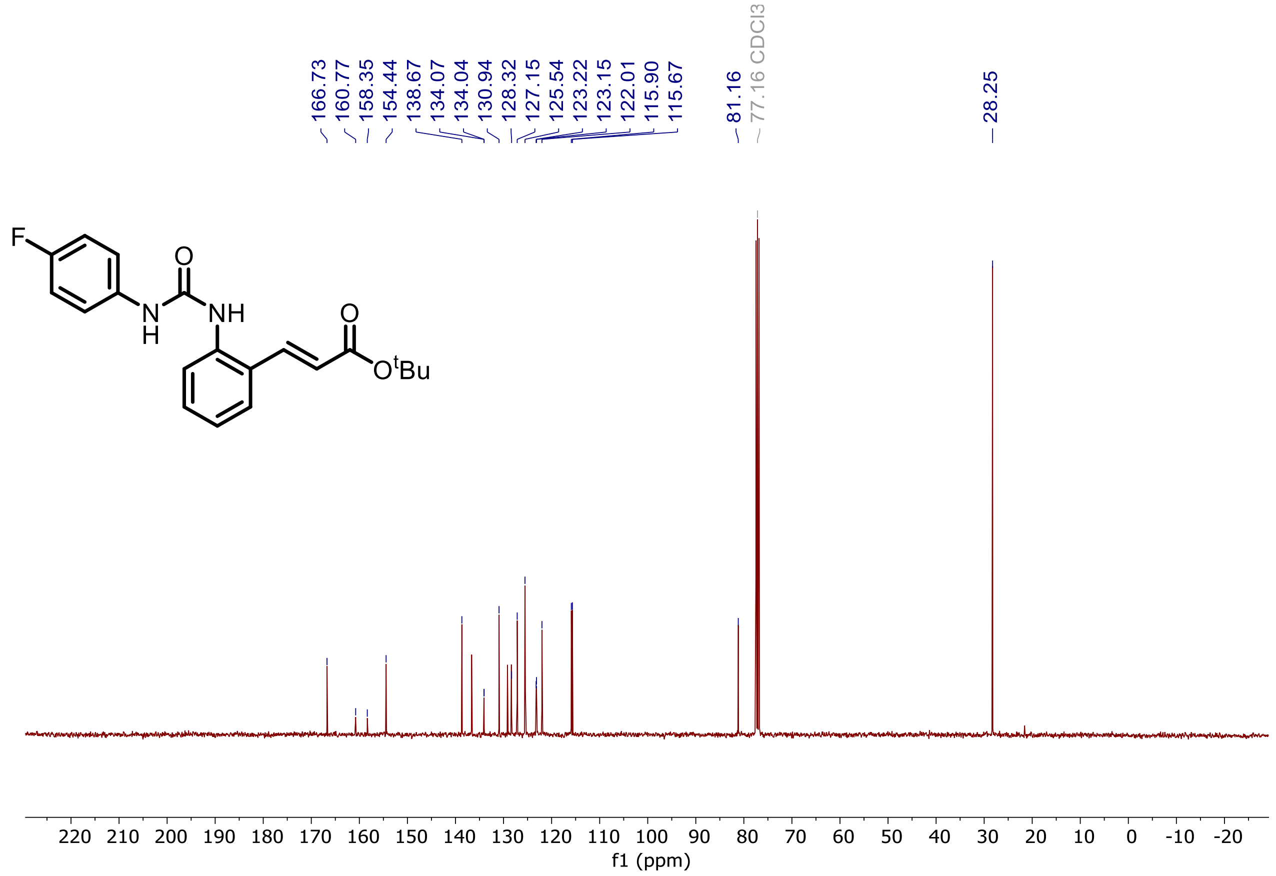

^{19}F NMR: (377 MHz, $CDCl_3$, 298K) of **1m**

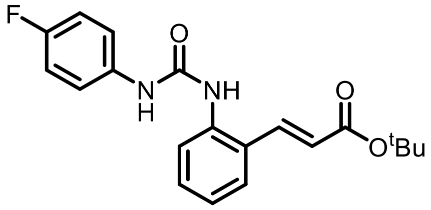

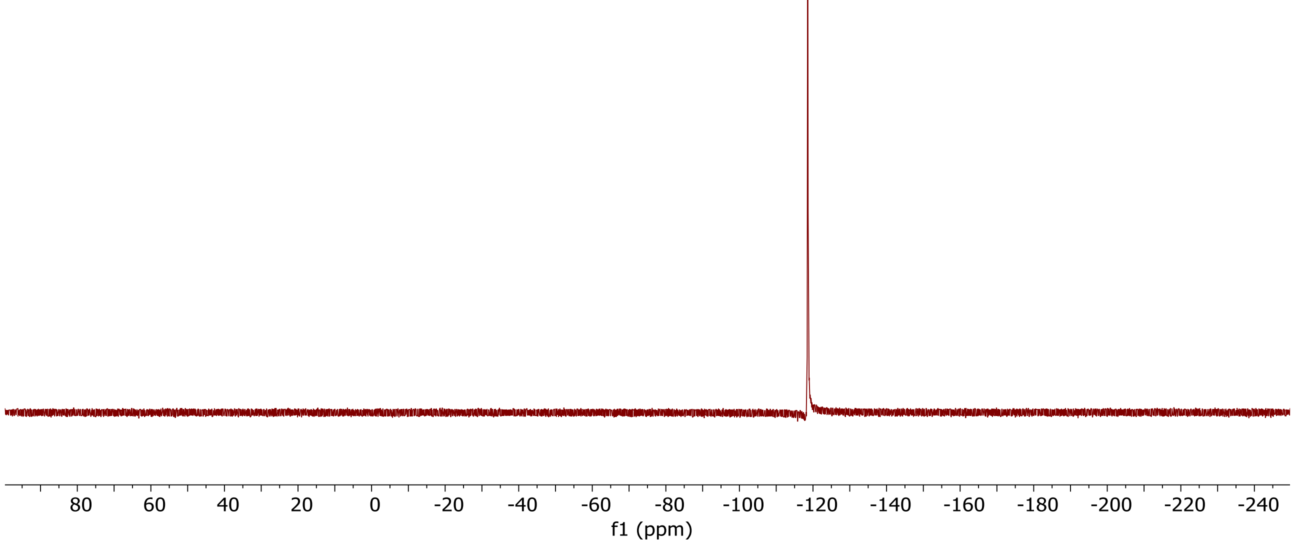

^{1}H NMR: (400 MHz, $CDCl_3$, 298K) of **1n**

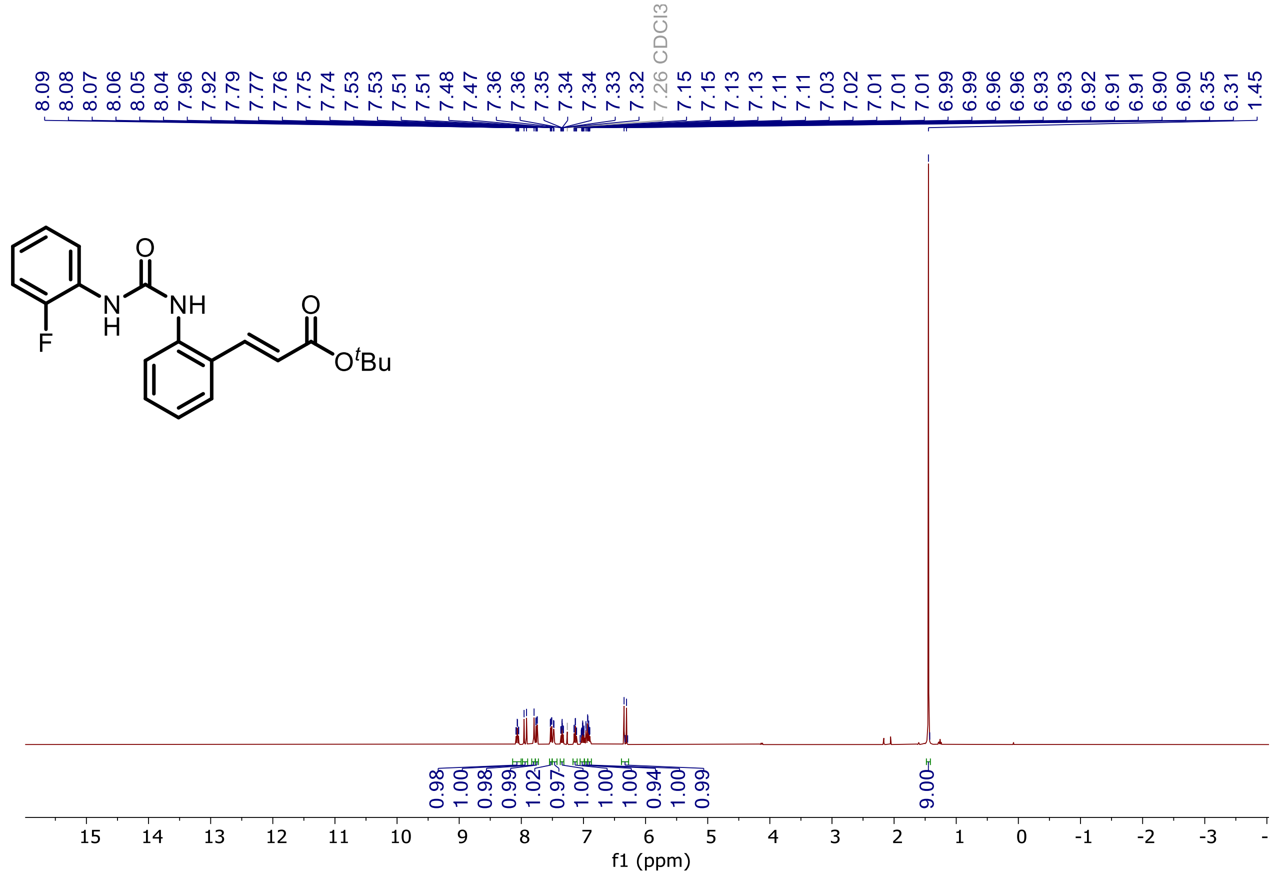

^{13}C NMR: (101 MHz, $CDCl_3$, 298K) of **1n**

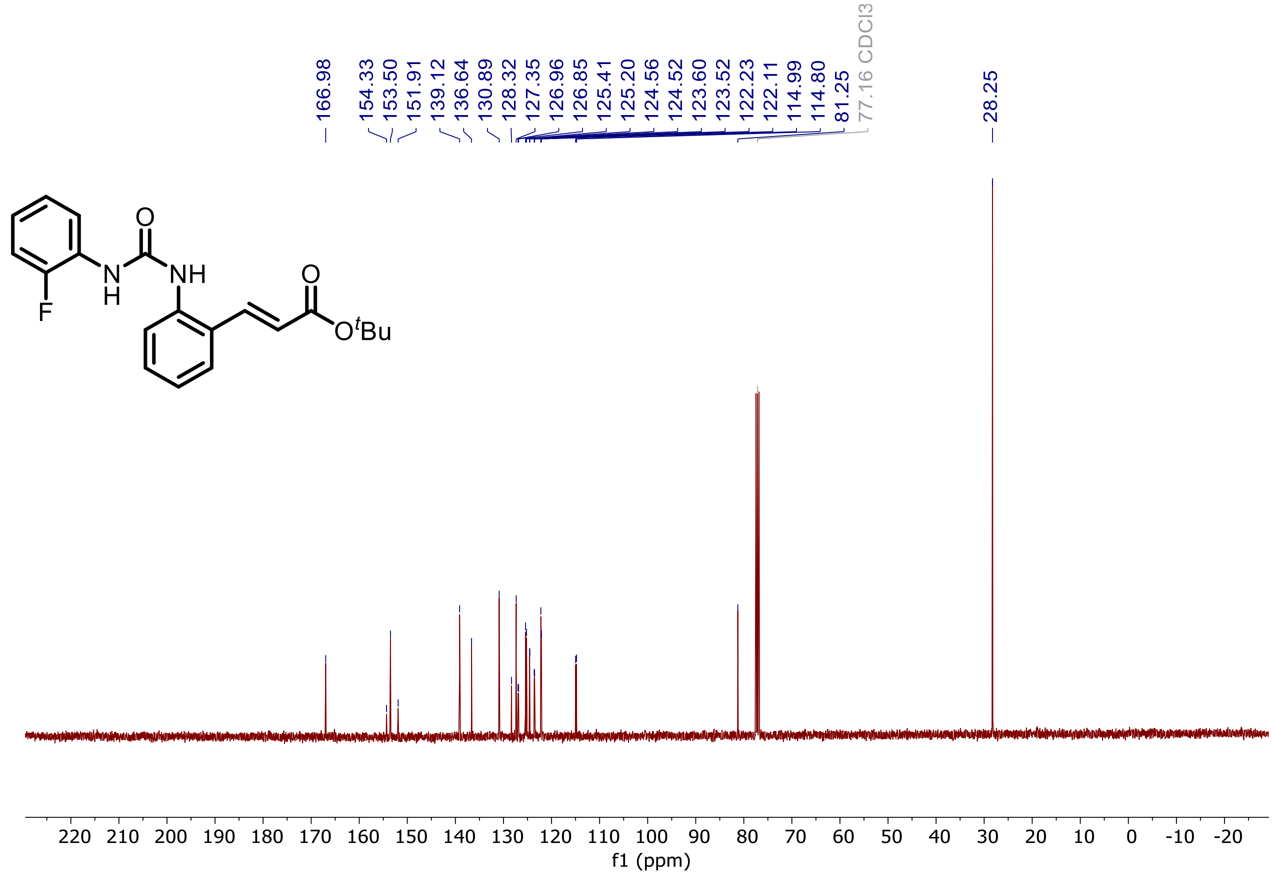

^{19}F NMR: (377 MHz, $CDCl_3$, 298K) of **1n**

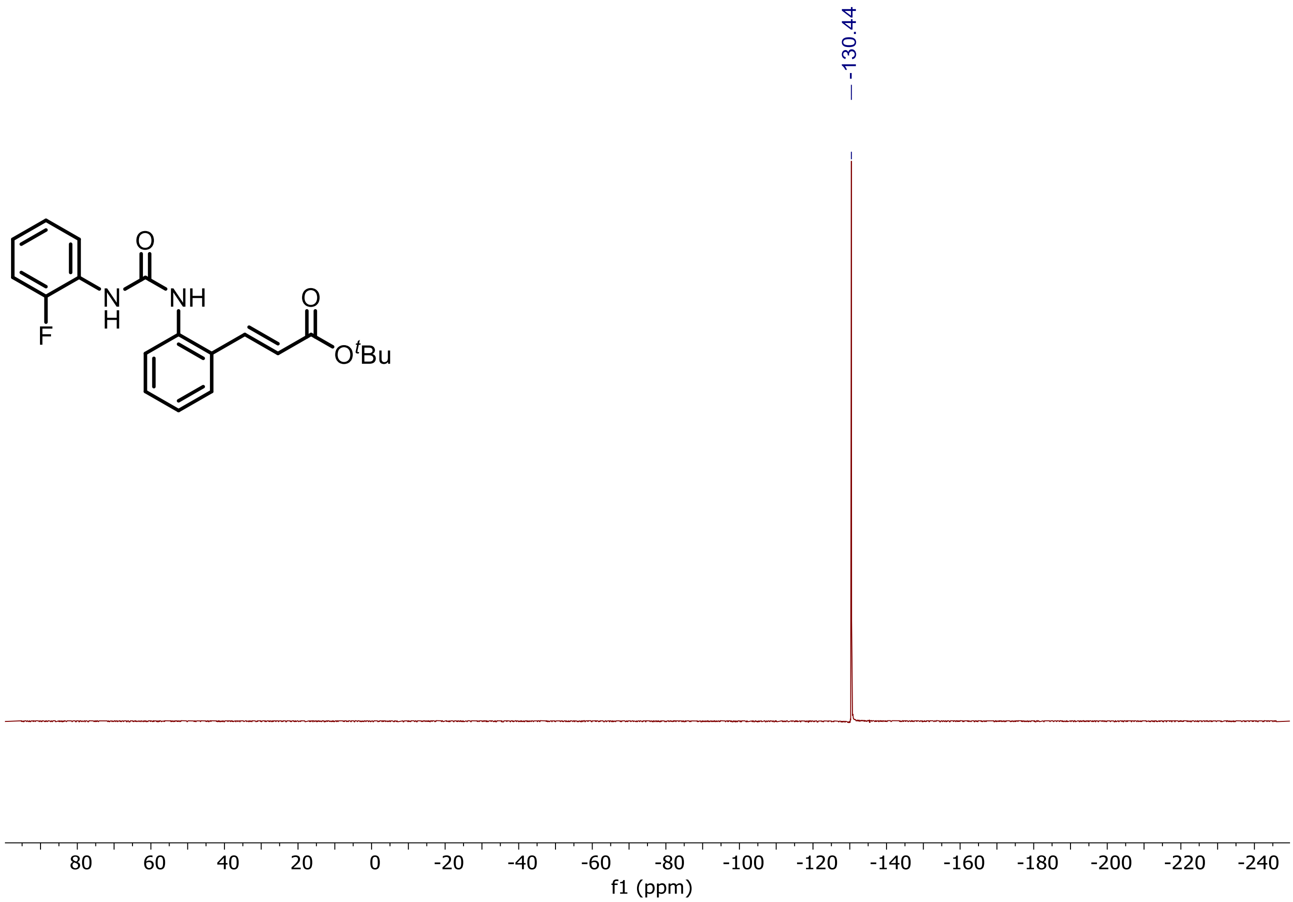

^{1}H NMR: (400 MHz, $CDCl_3$, 298K) of **1o**

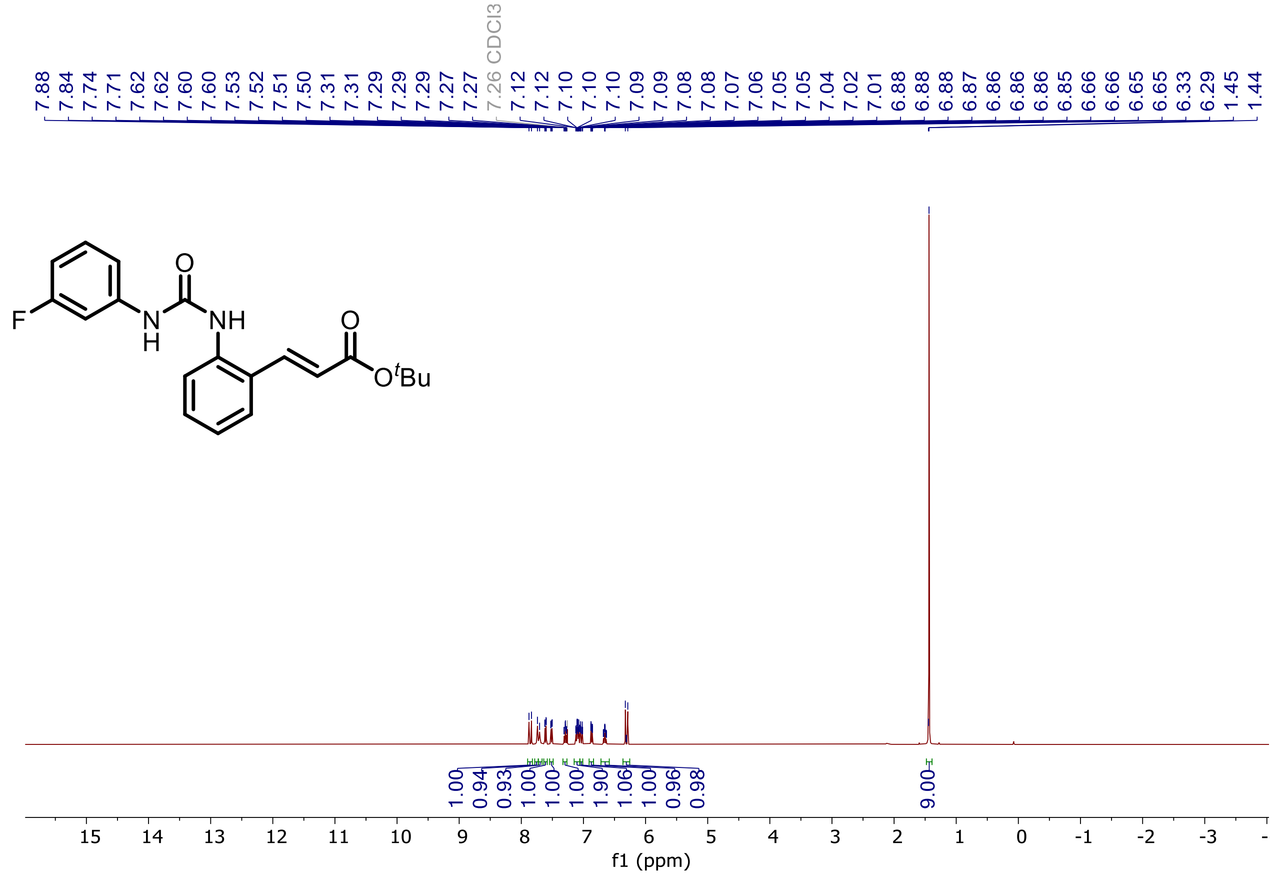

^{13}C NMR: (101 MHz, $CDCl_3$, 298K) of **1o**

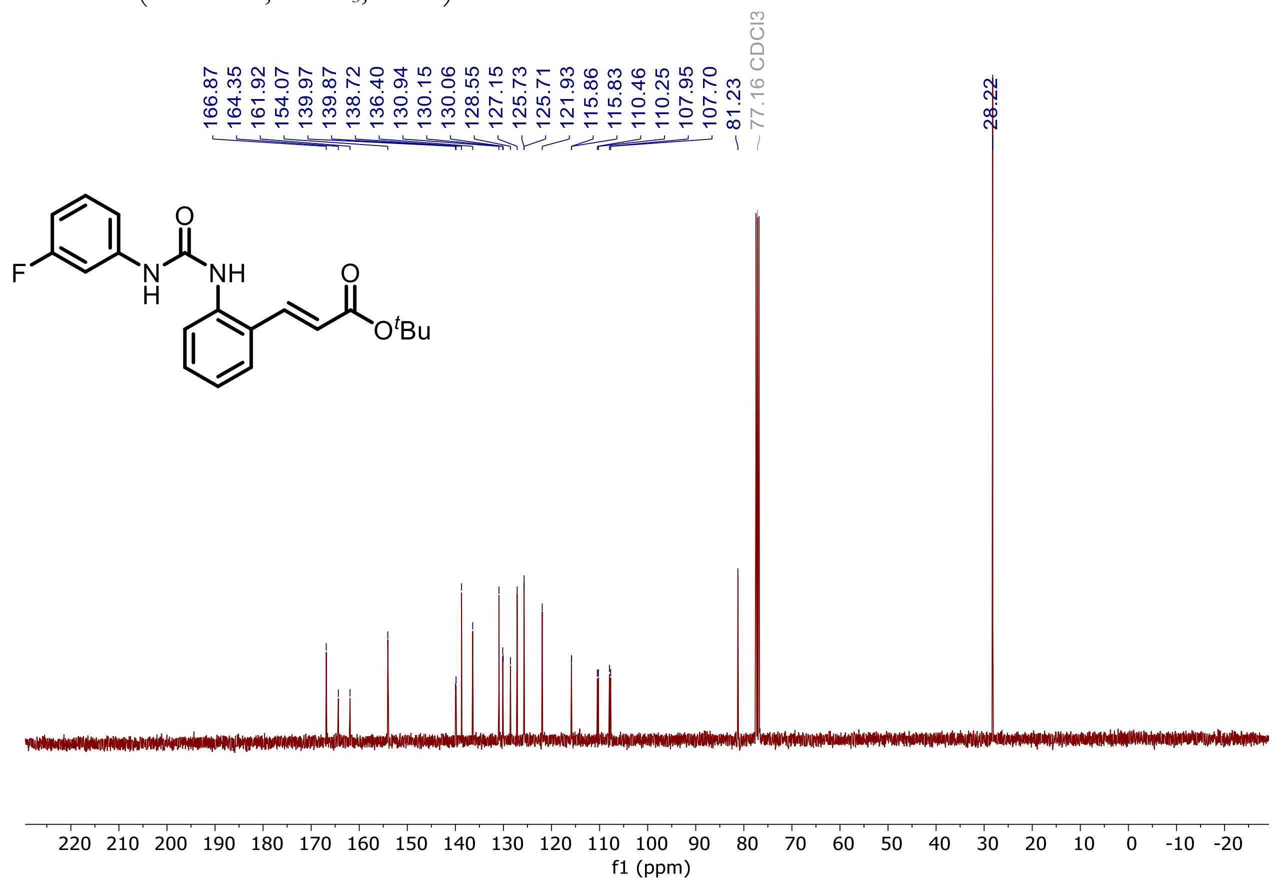

^{19}F NMR: (377 MHz, $CDCl_3$, 298K) of **1o**

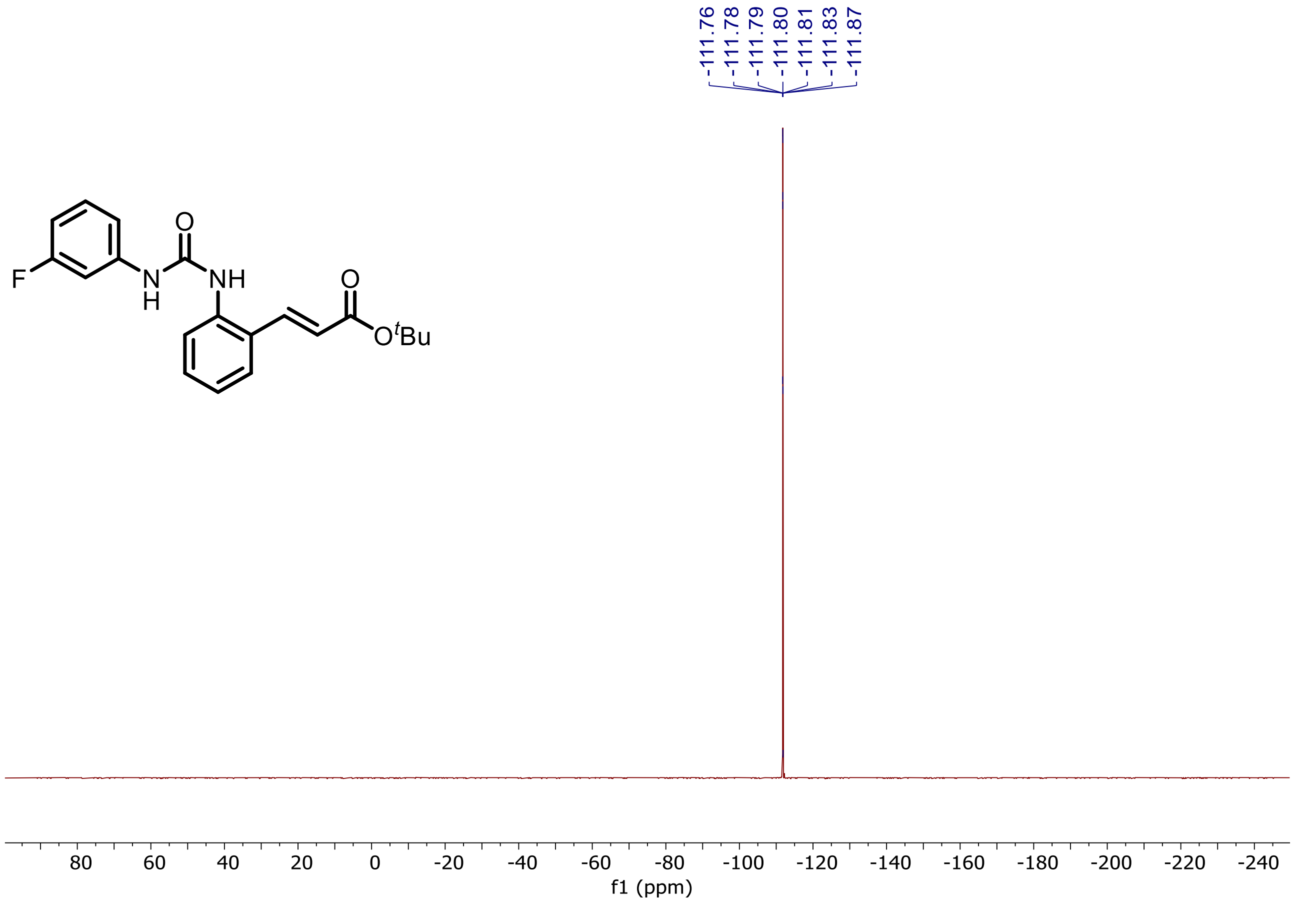

^{1}H NMR: (400 MHz, $(CD_3)_2SO$, 298K) of **1p**

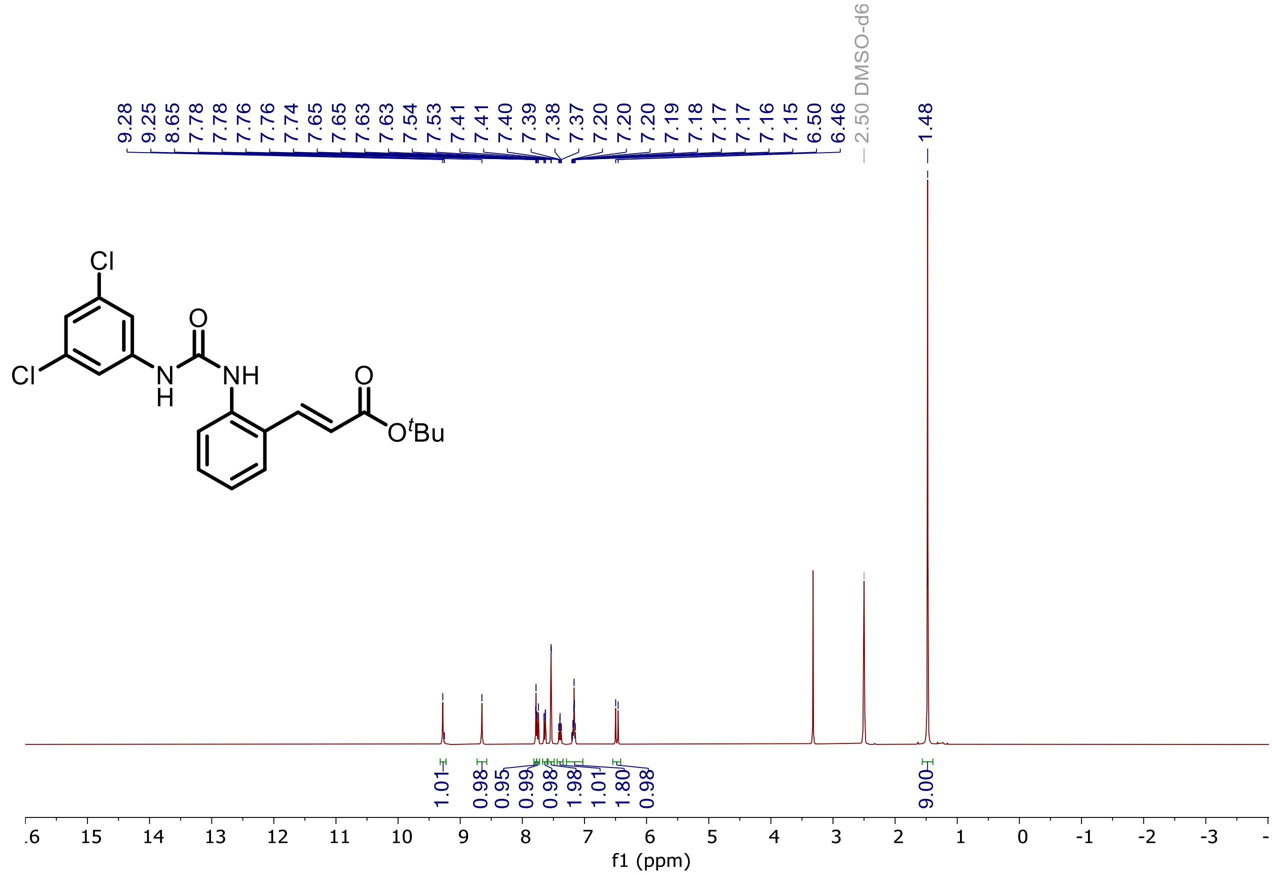

^{13}C NMR: (101 MHz, $(CD_3)_2SO$, 298K) of **1p**

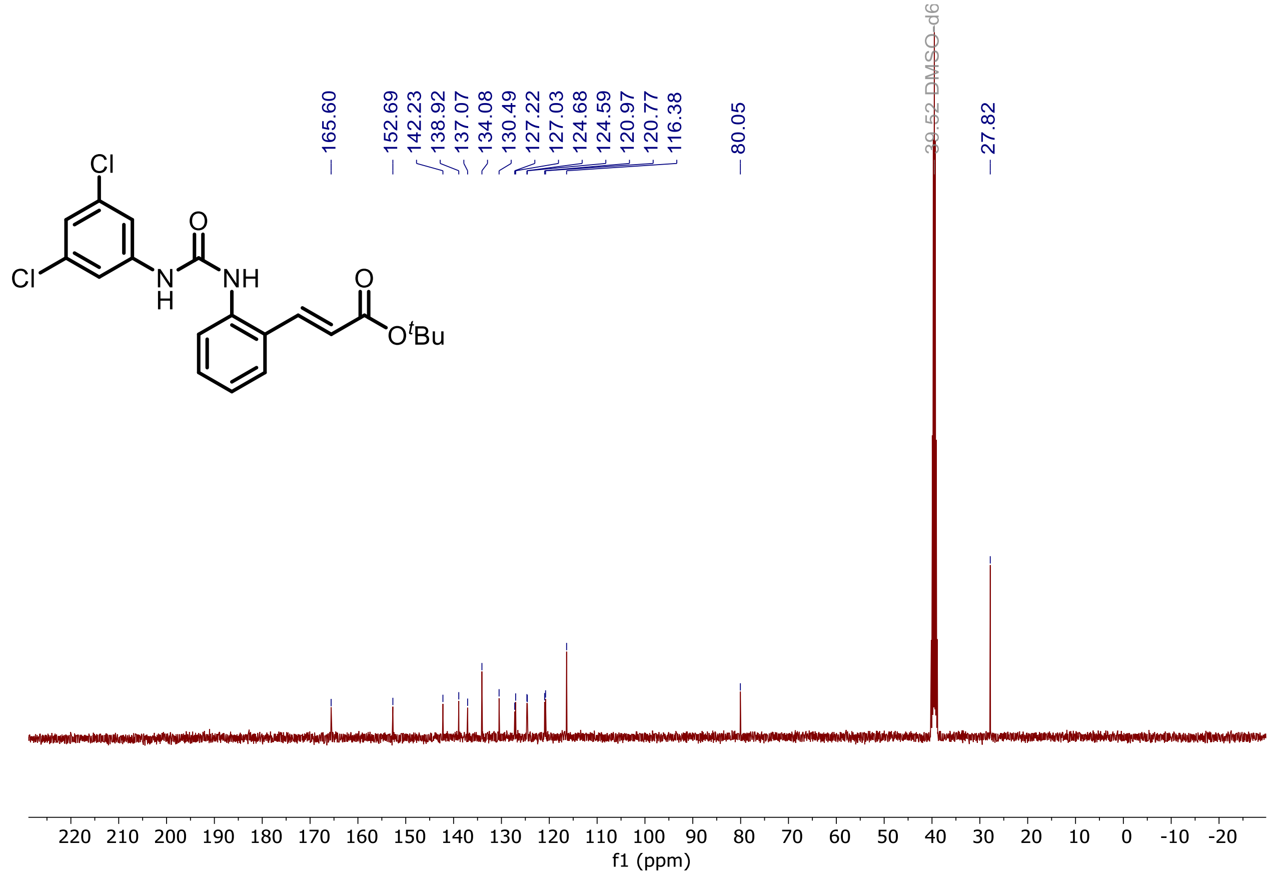

^{1}H NMR: (400 MHz, $CDCl_3$, 298K) of **1q**

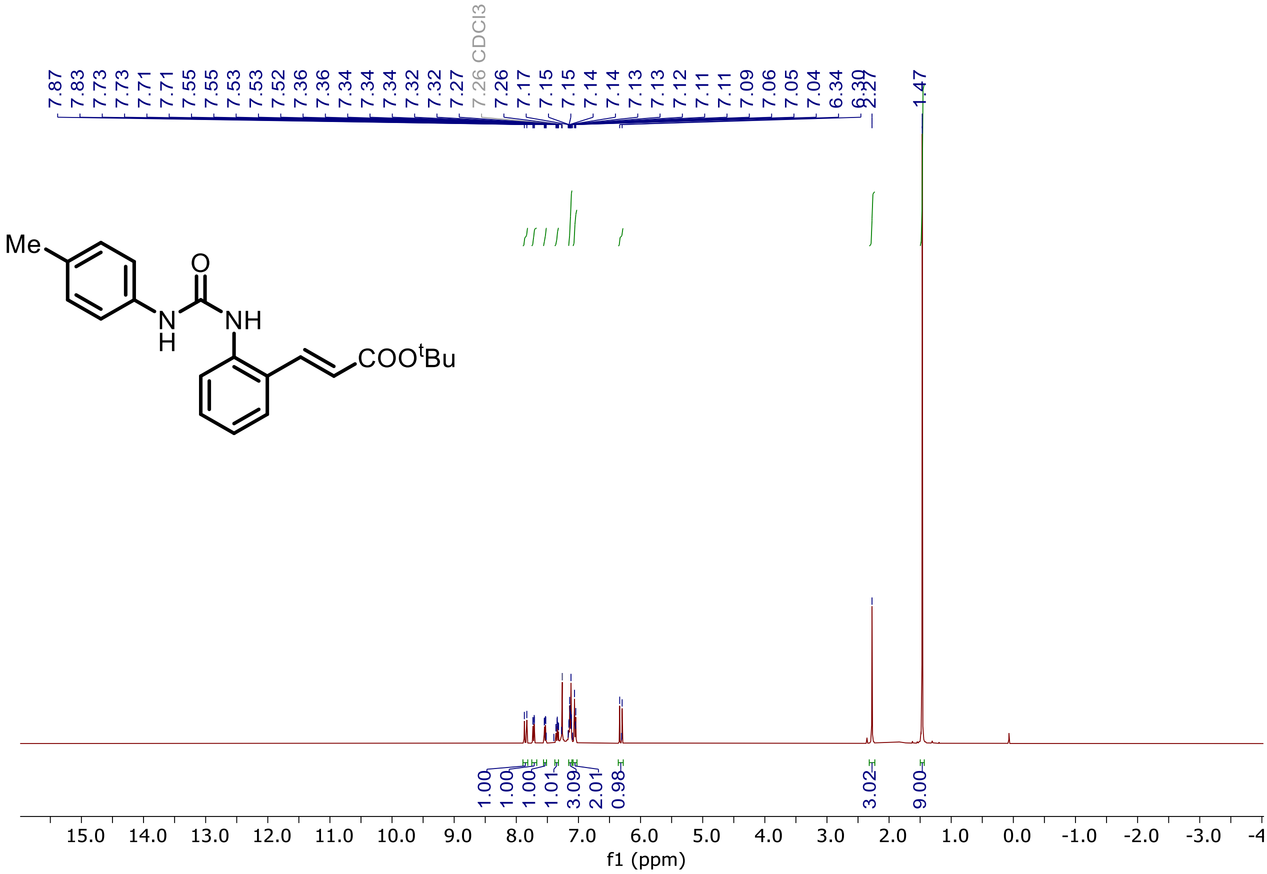

^{13}C NMR: (101 MHz, $CDCl_3$, 298K) of **1q**

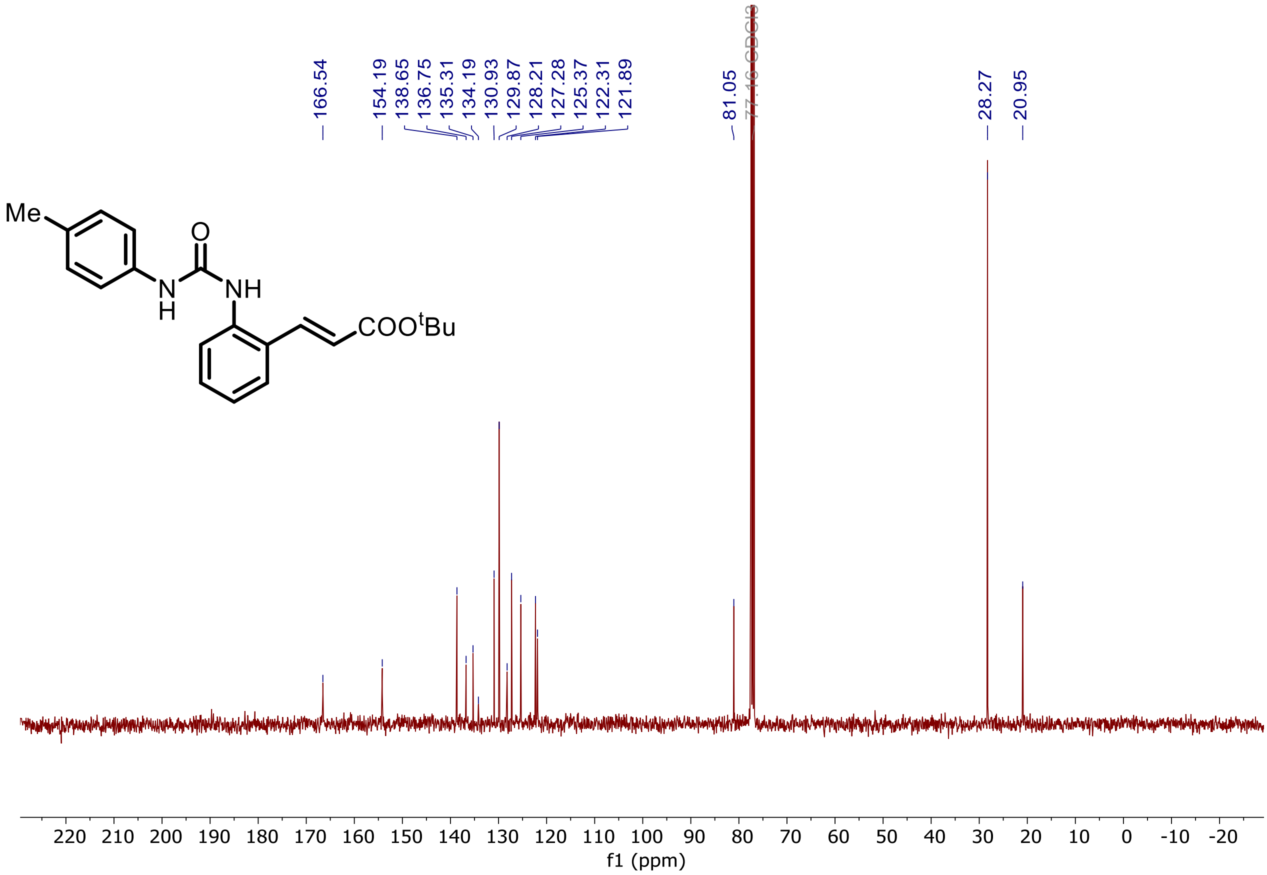

^{1}H NMR: (400 MHz, $CDCl_3$, 298K) of **1r**

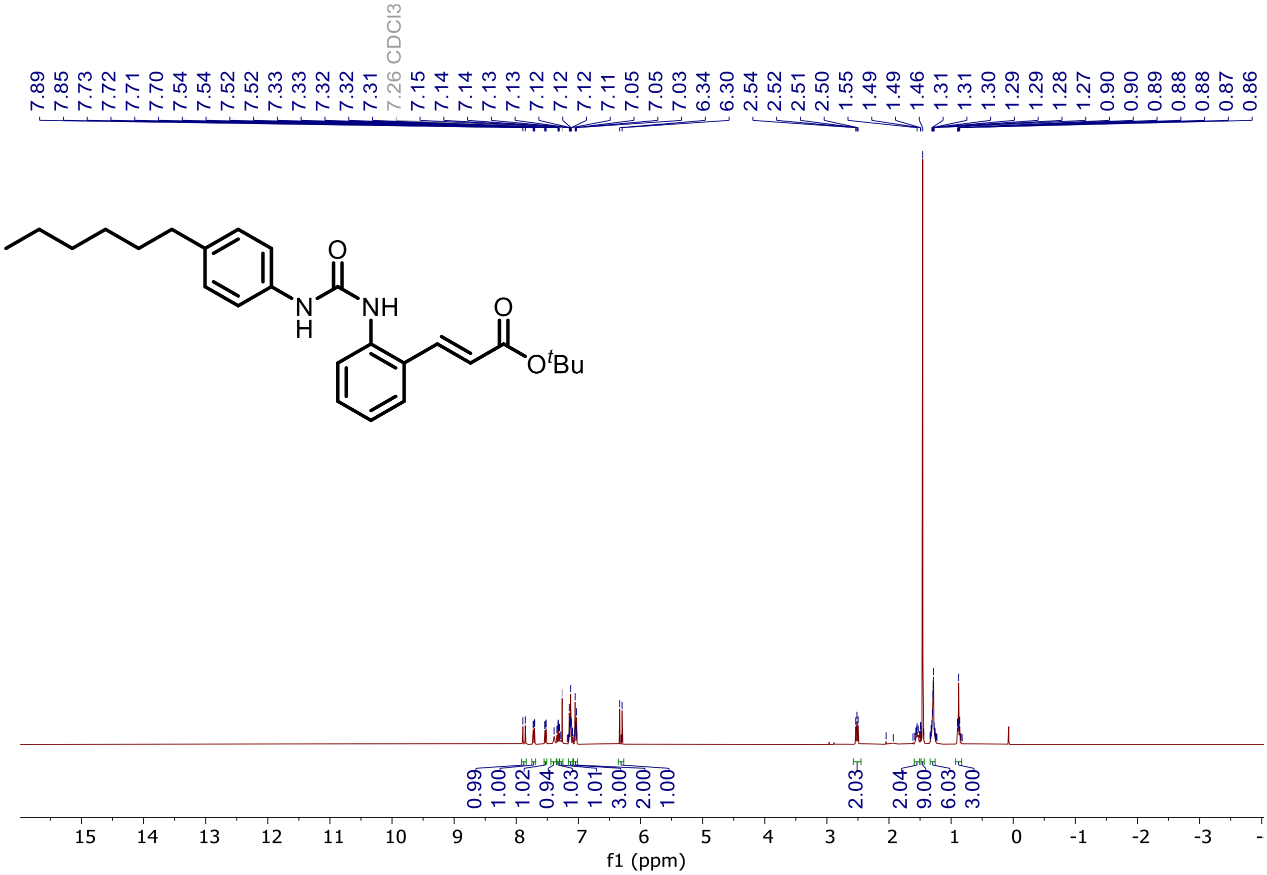

^{13}C NMR: (101 MHz, $CDCl_3$, 298K) of **1r**

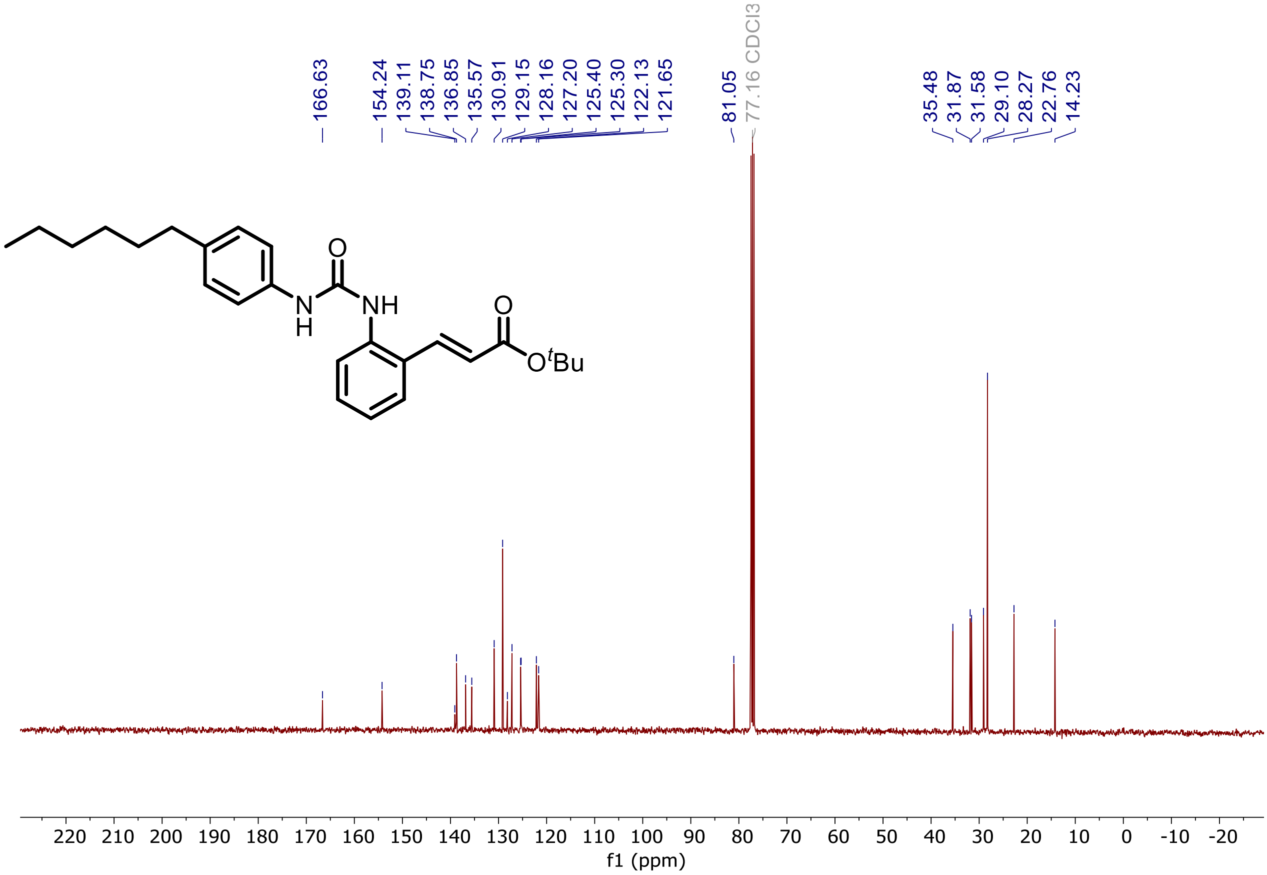

^{1}H NMR: (400 MHz, $(CD_3)_2SO$, 298K) of **1s**

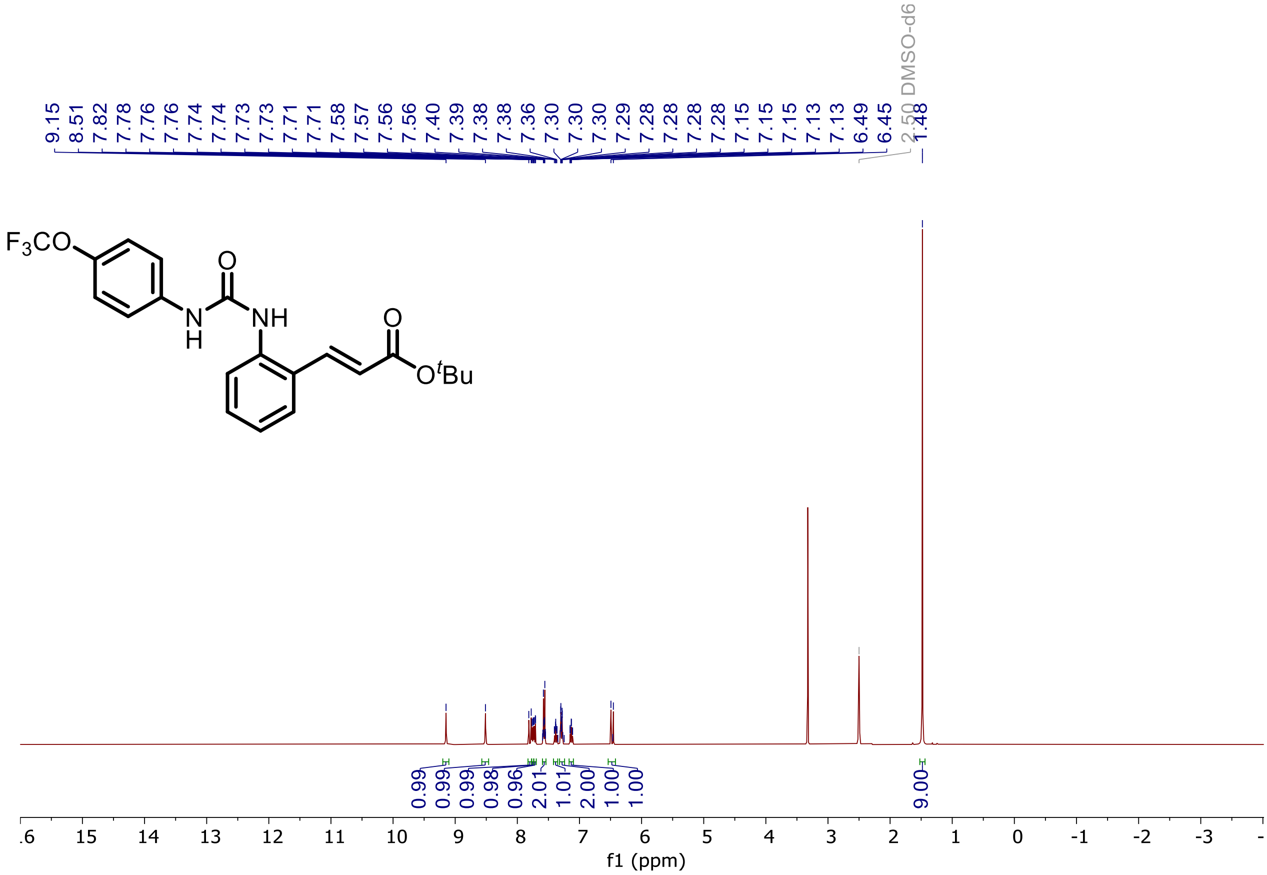

^{13}C NMR: (101 MHz, $(CD_3)_2SO$, 298K) of **1s**

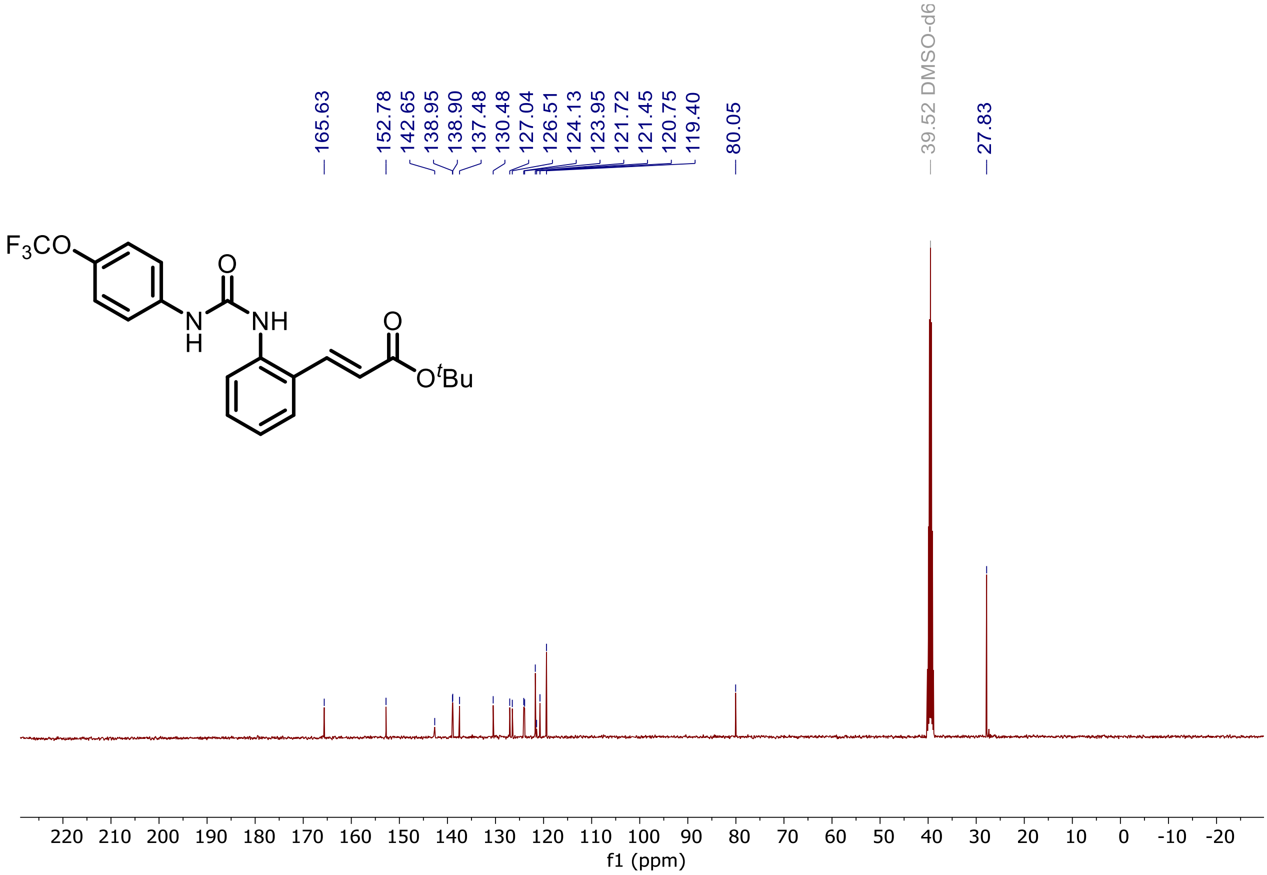

^{19}F NMR: (377 MHz, $(CD_3)_2SO$, 298K) of **1s**

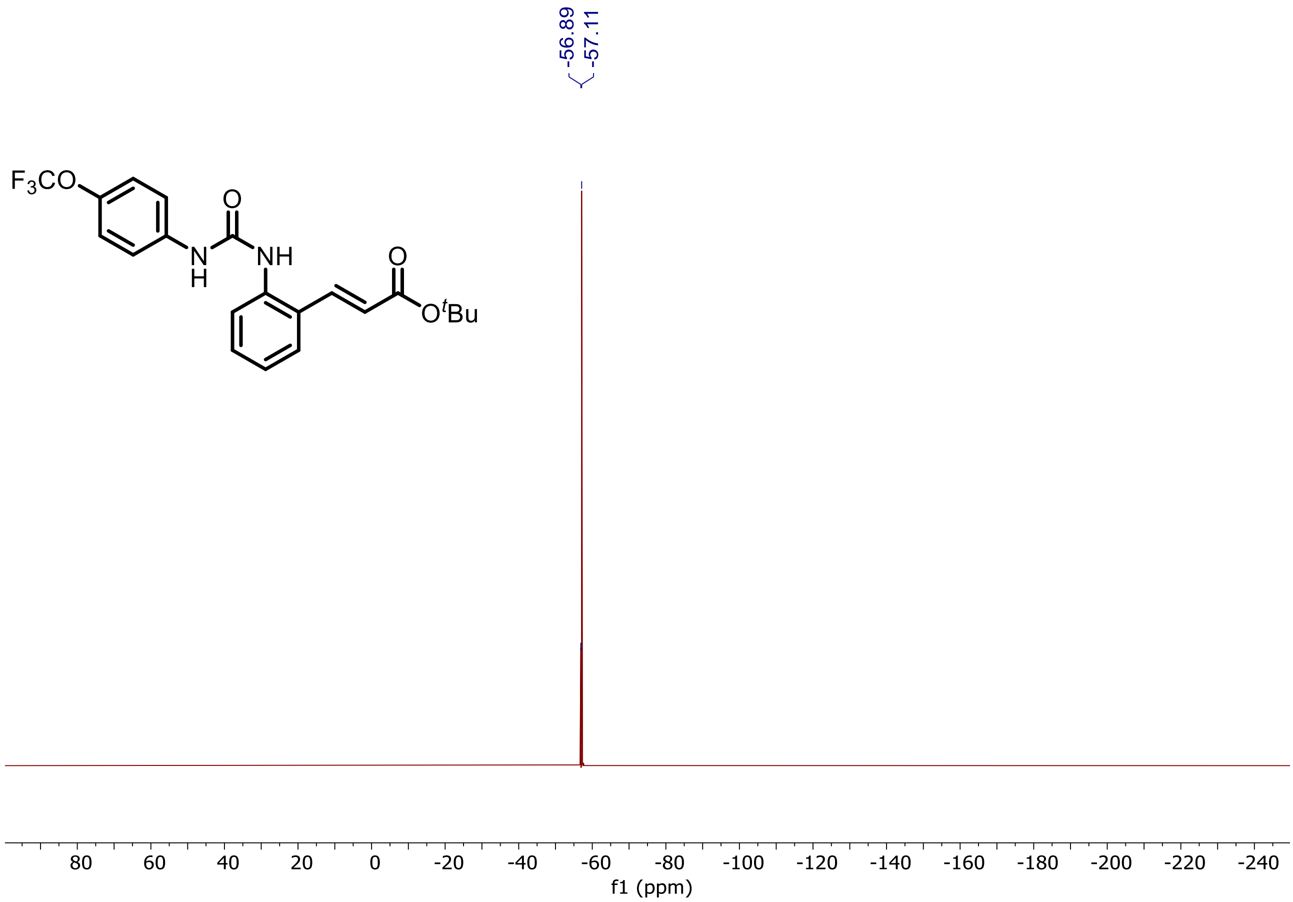

^{1}H NMR: (400 MHz, $CDCl_3$, 298K) of **1t**

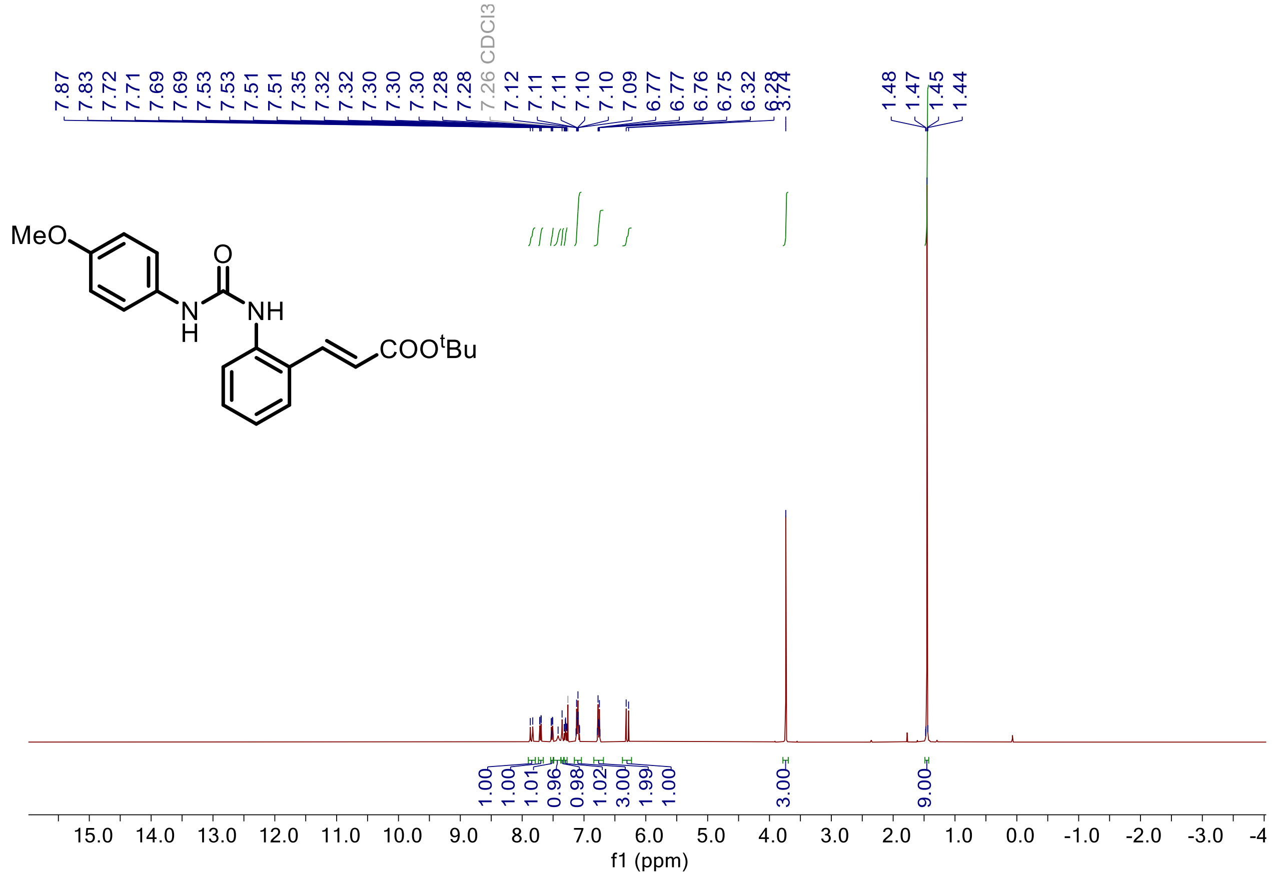

^{13}C NMR: (101 MHz, $CDCl_3$, 298K) of **1t**

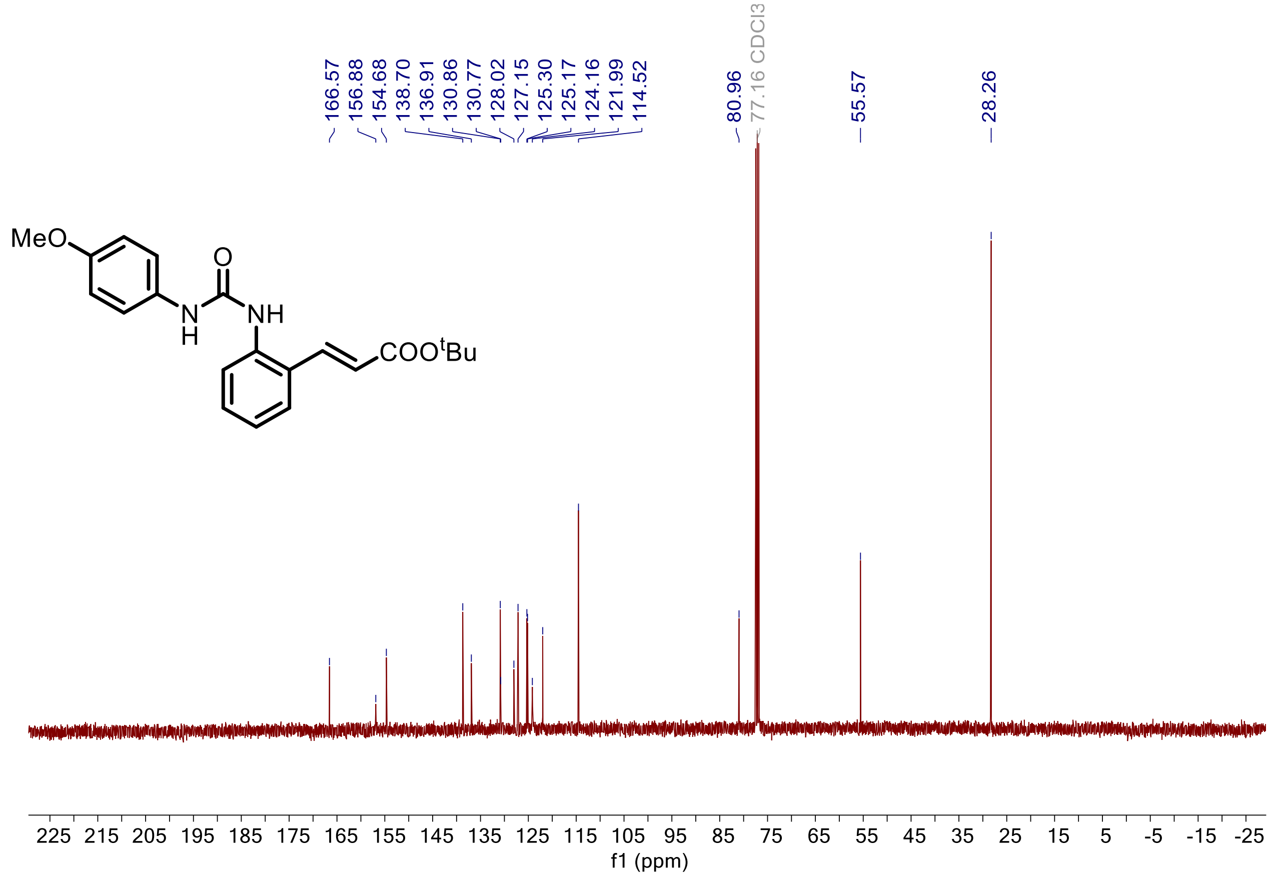

^{1}H NMR: (400 MHz, $CDCl_3$, 298K) of **1u**

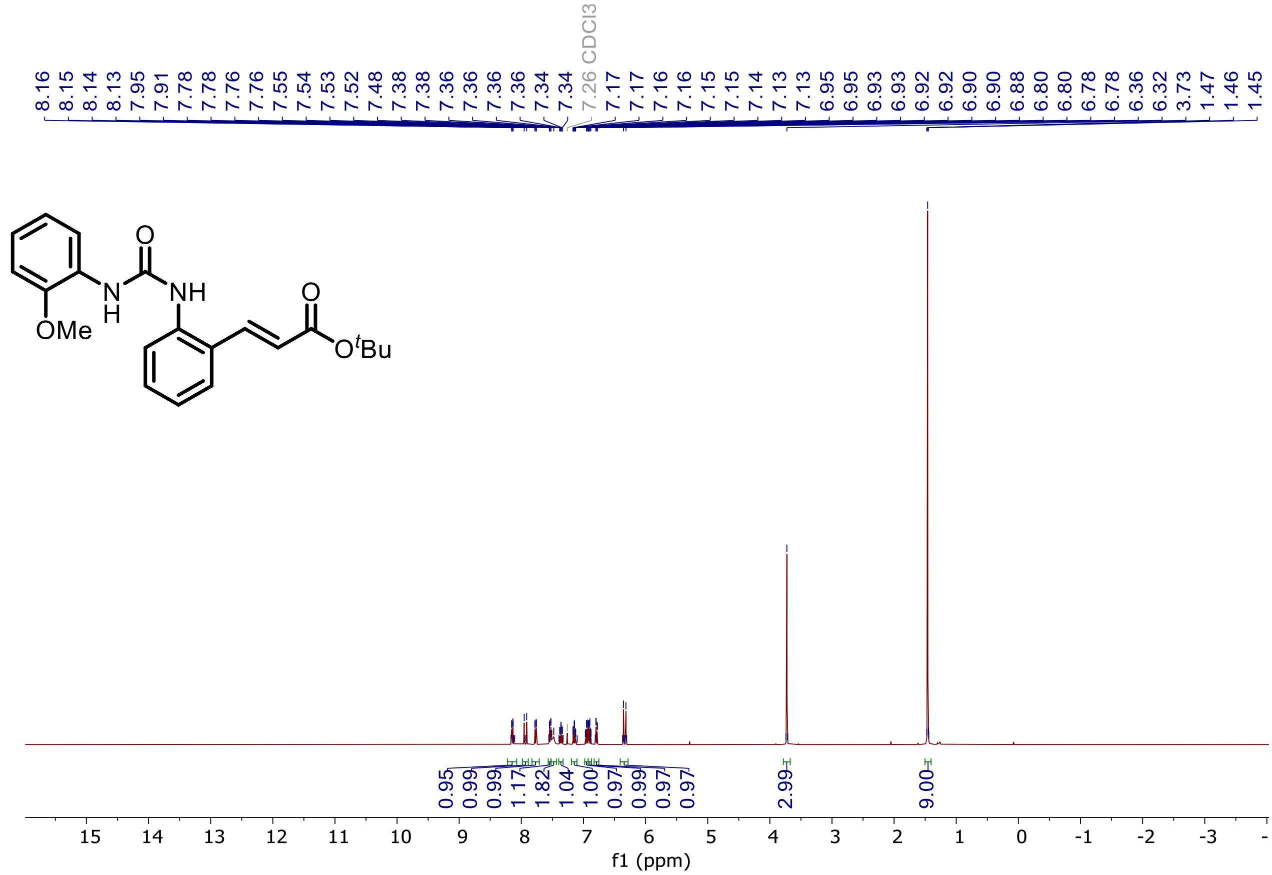

^{13}C NMR: (101 MHz, $CDCl_3$, 298K) of **1u**

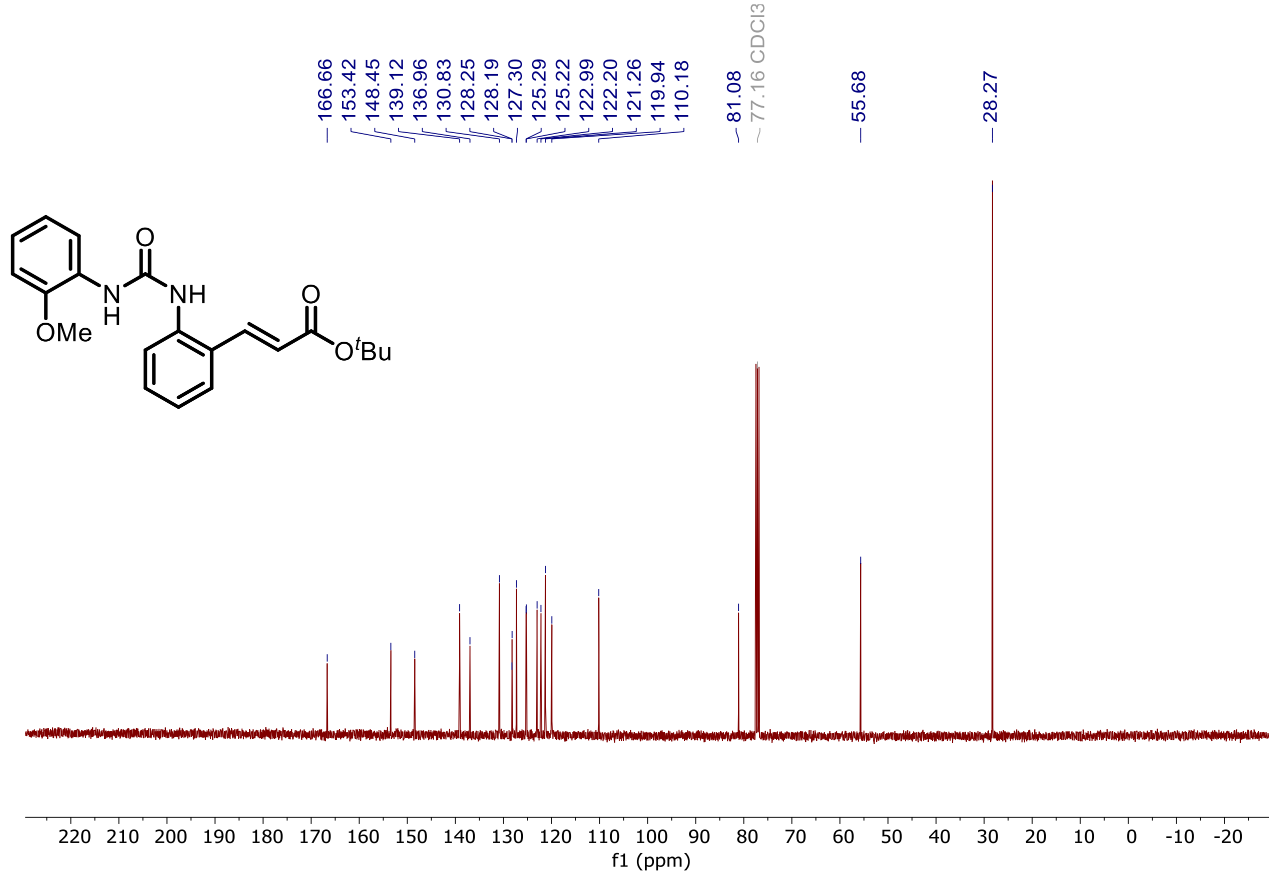

^{1}H NMR: (400 MHz, $CDCl_3$, 298K) of **1v**

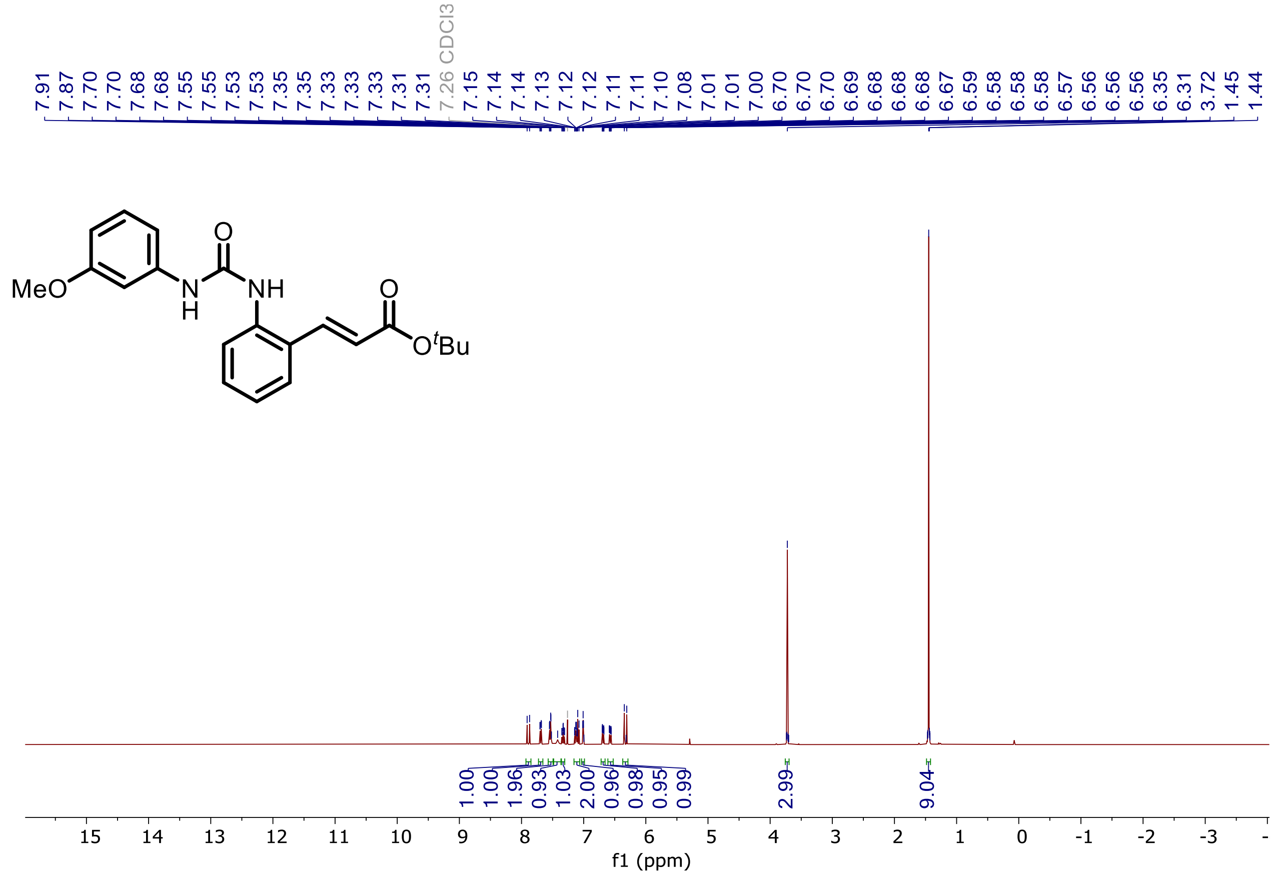

^{13}C NMR: (101 MHz, $CDCl_3$, 298K) of **1v**

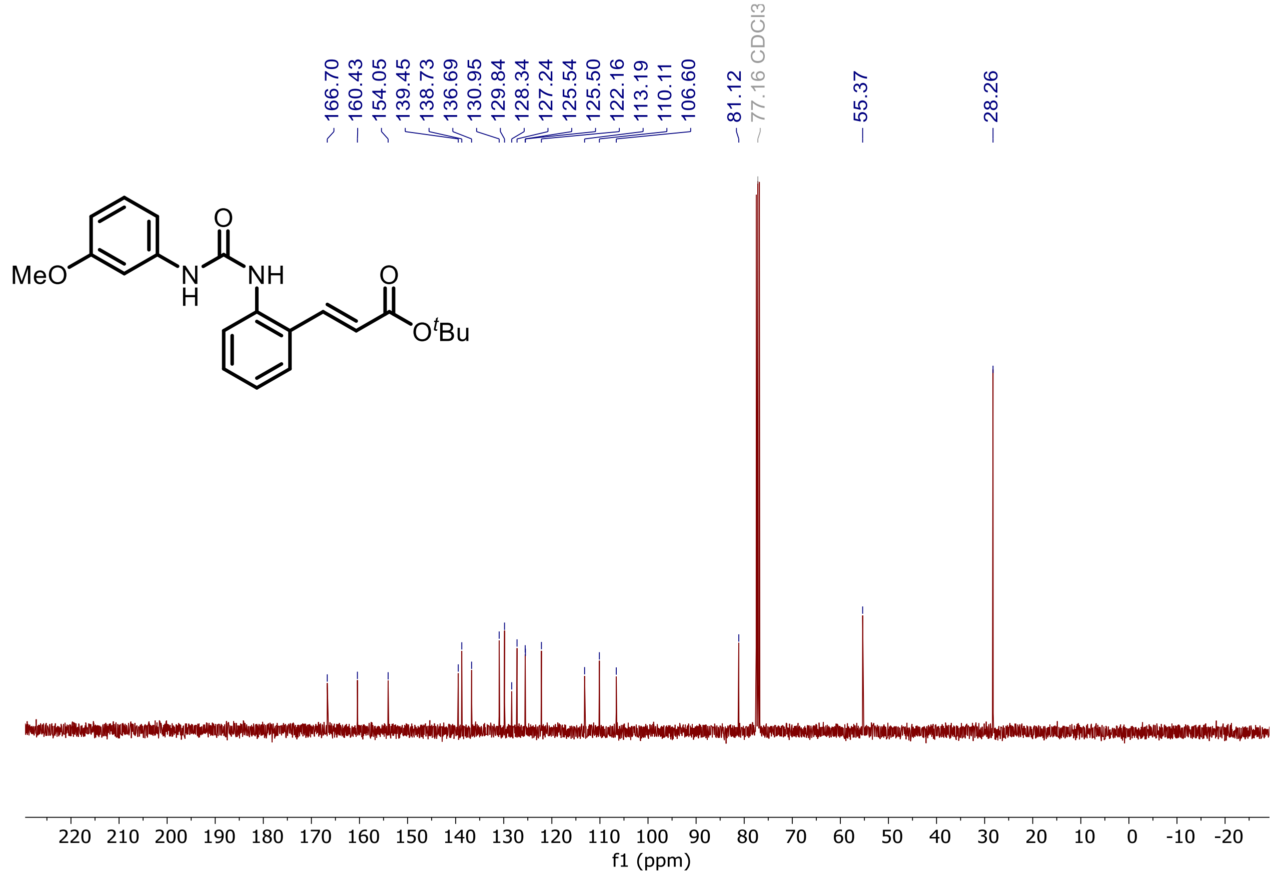

^{1}H NMR: (400 MHz, $CDCl_3$, 298K) of **1w**

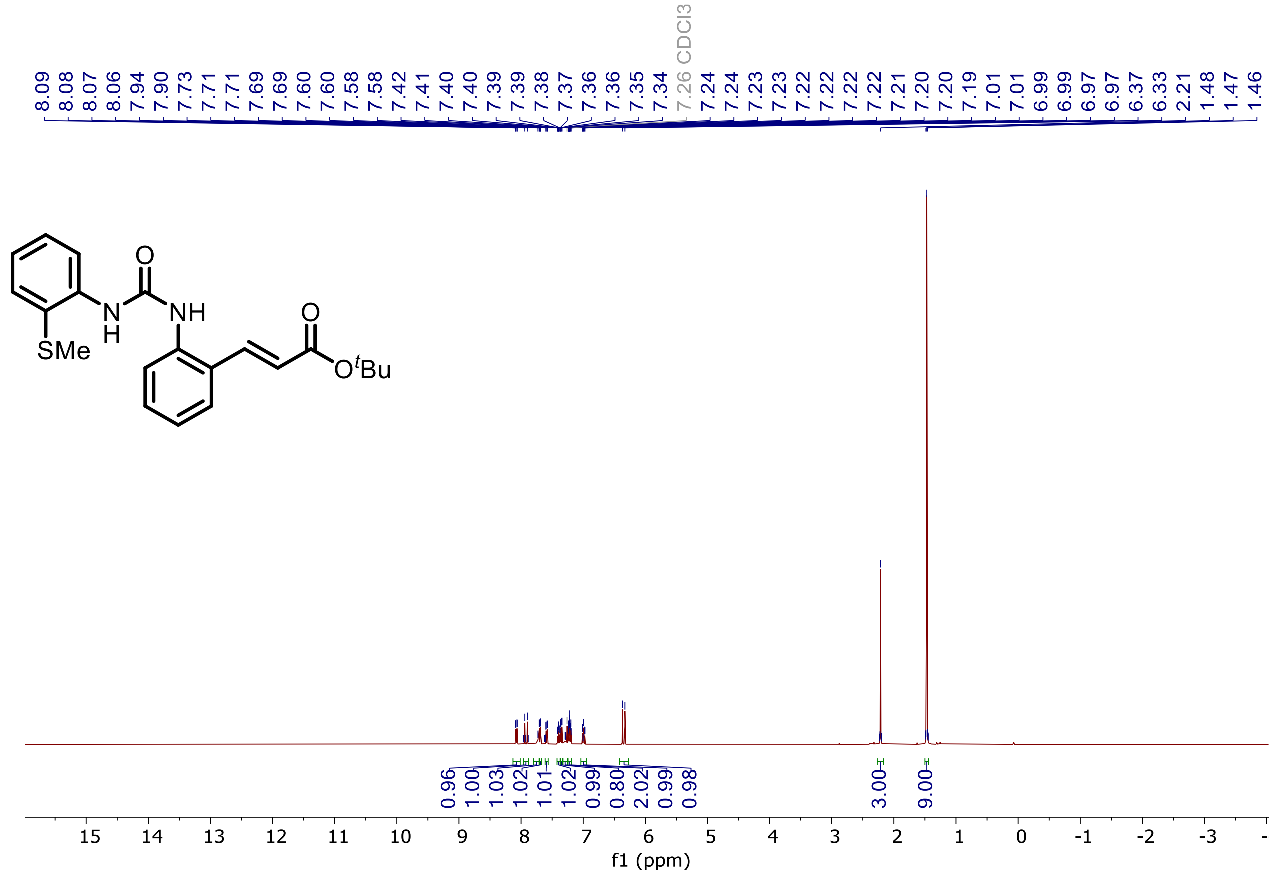

^{13}C NMR: (101 MHz, $CDCl_3$, 298K) of **1w**

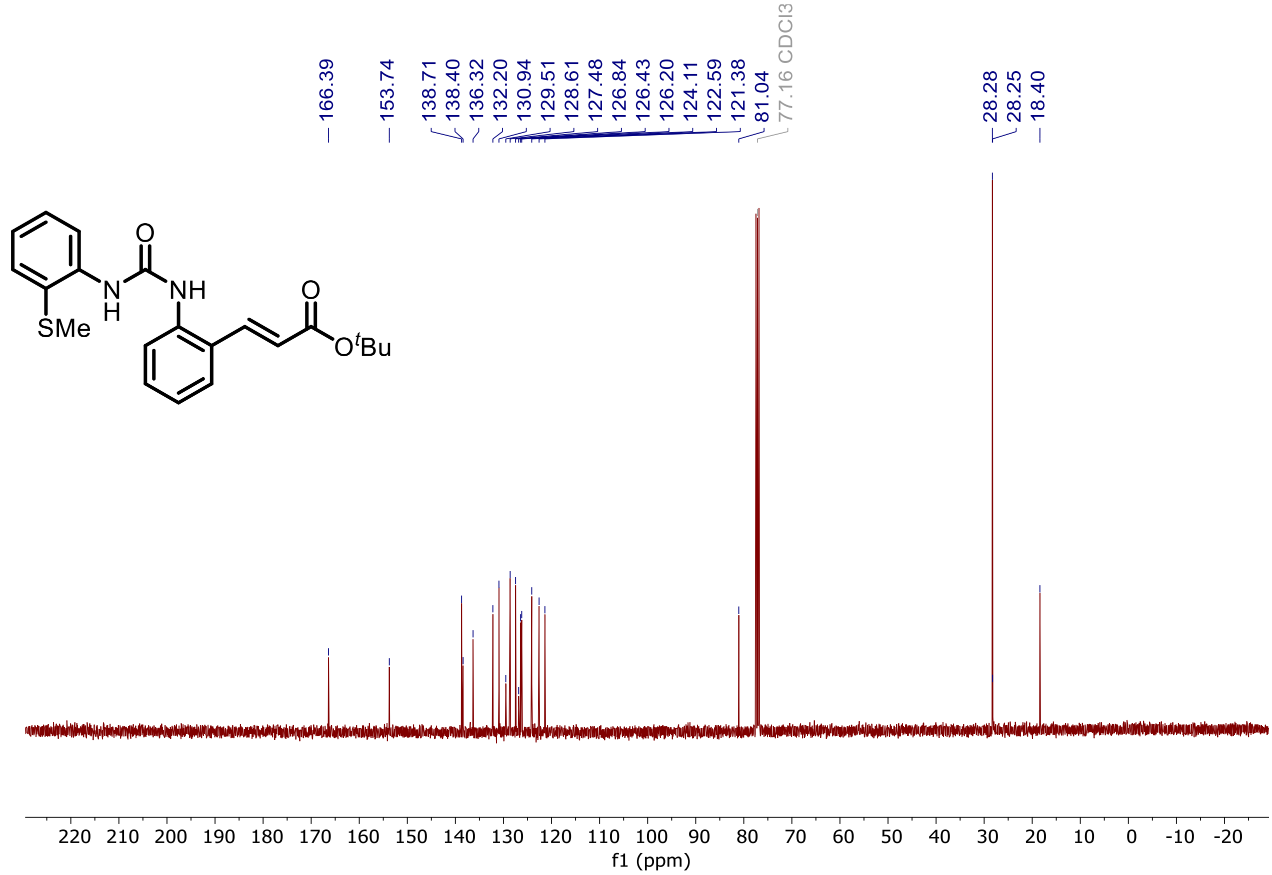

^{1}H NMR: (400 MHz, $CDCl_3$, 298K) of **1x**

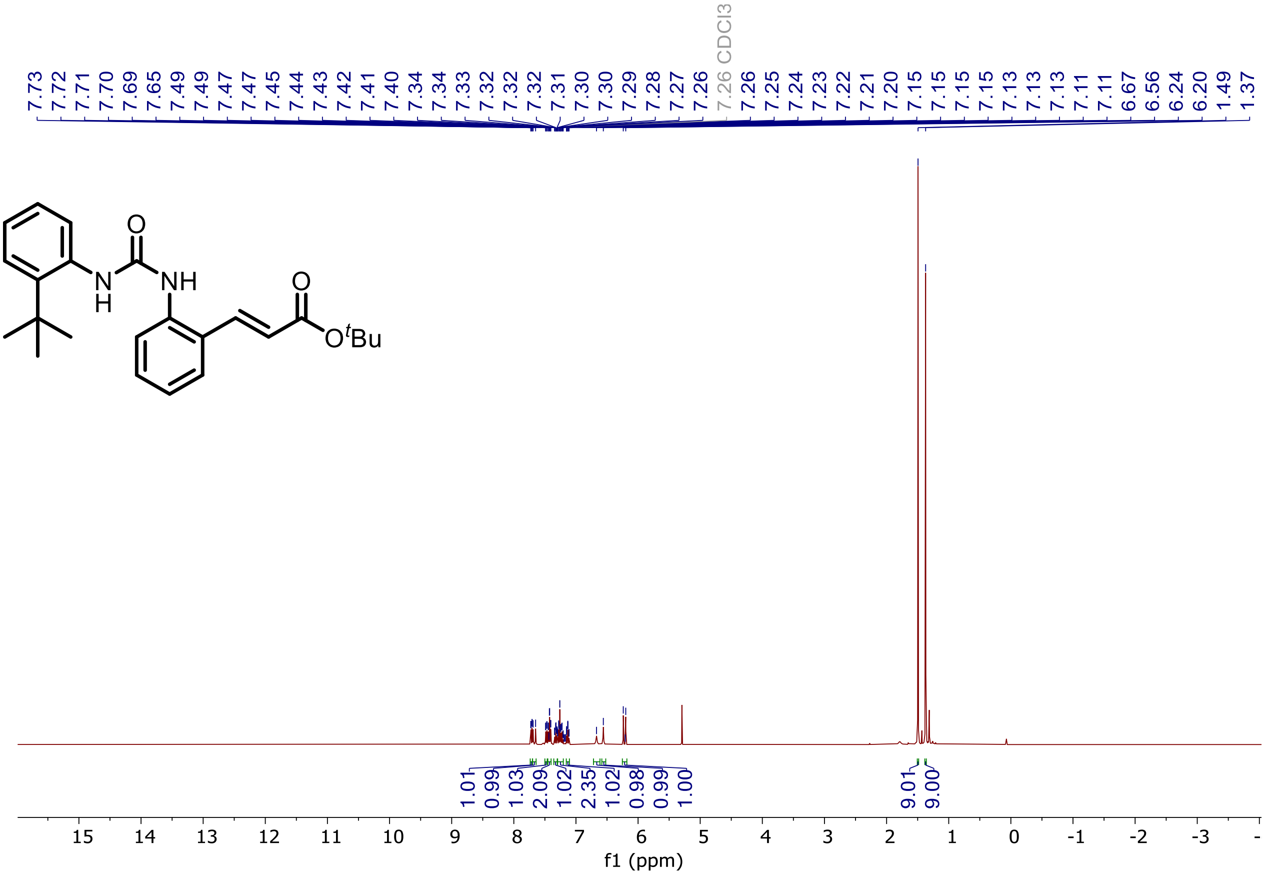

^{13}C NMR: (101 MHz, $CDCl_3$, 298K) of **1x**

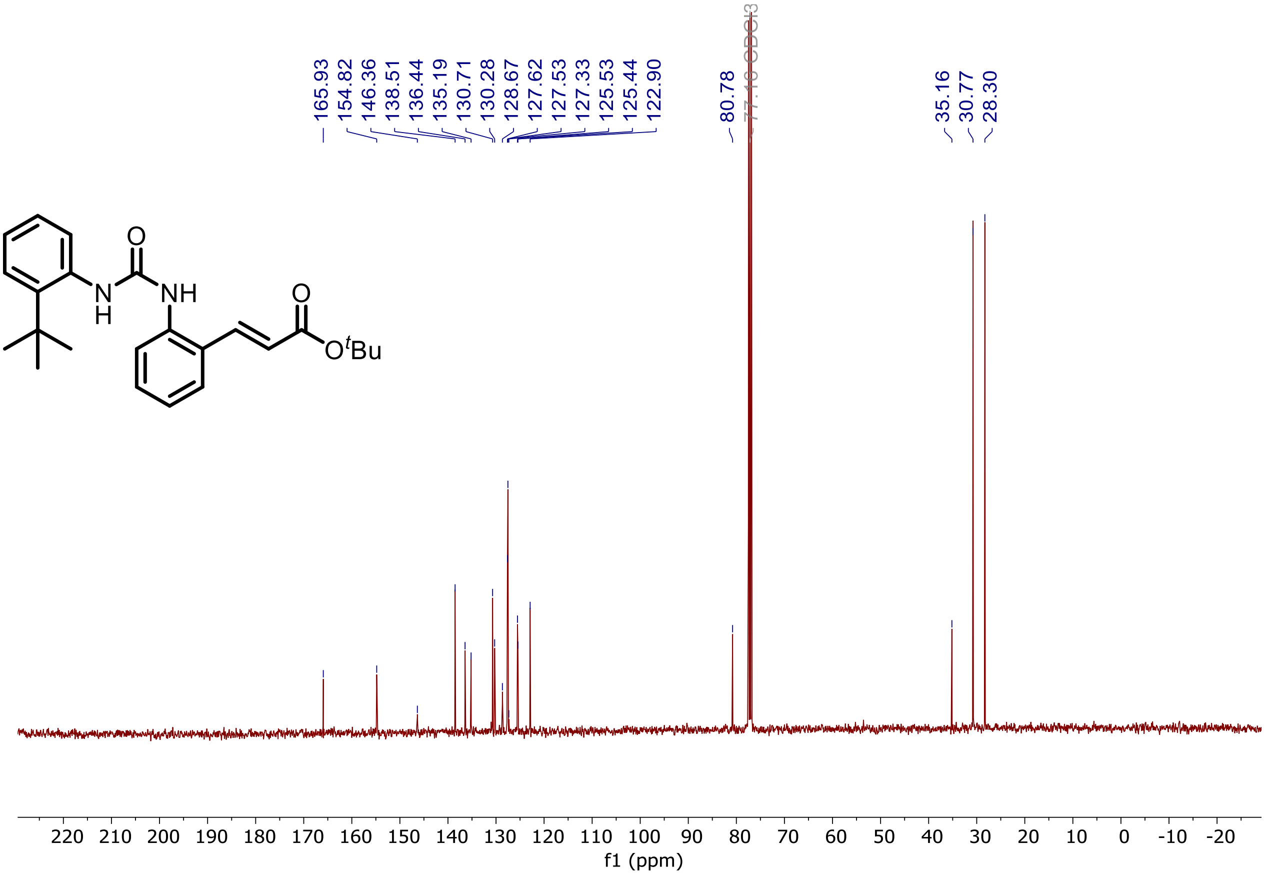

^{1}H NMR: (400 MHz, $CDCl_3$, 298K) of **1y**

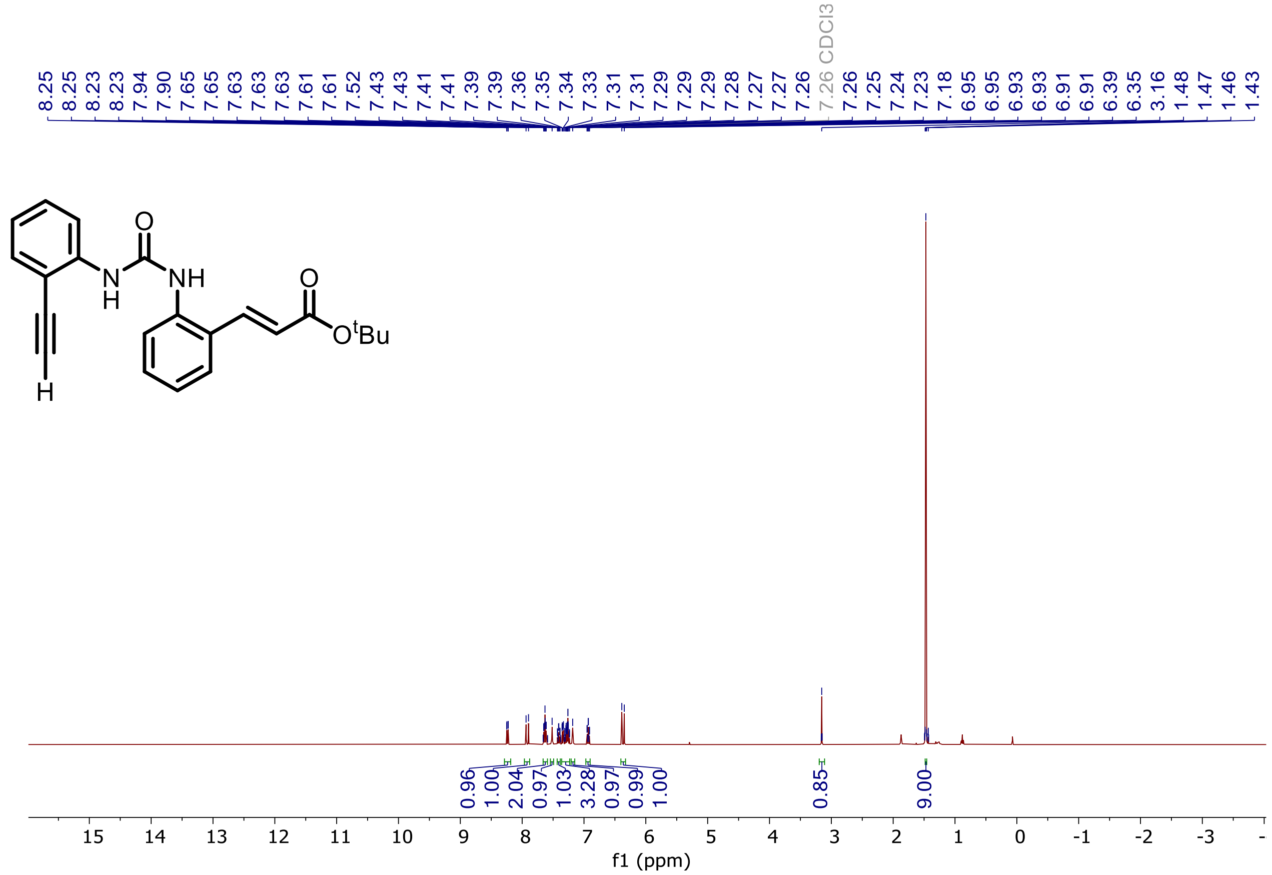

^{13}C NMR: (101 MHz, $CDCl_3$, 298K) of **1y**

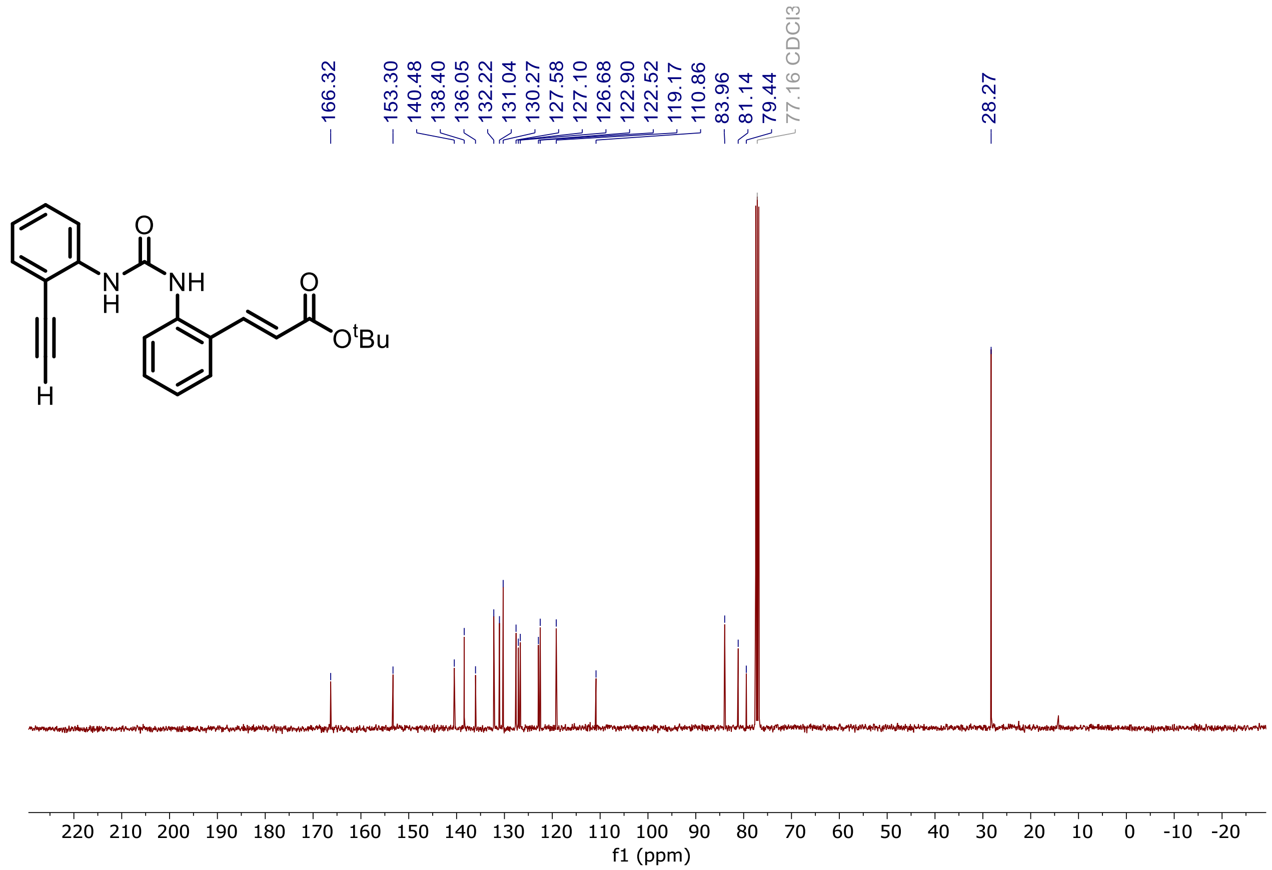

^{1}H NMR: (400 MHz, $CDCl_3$, 298K) of **1z**

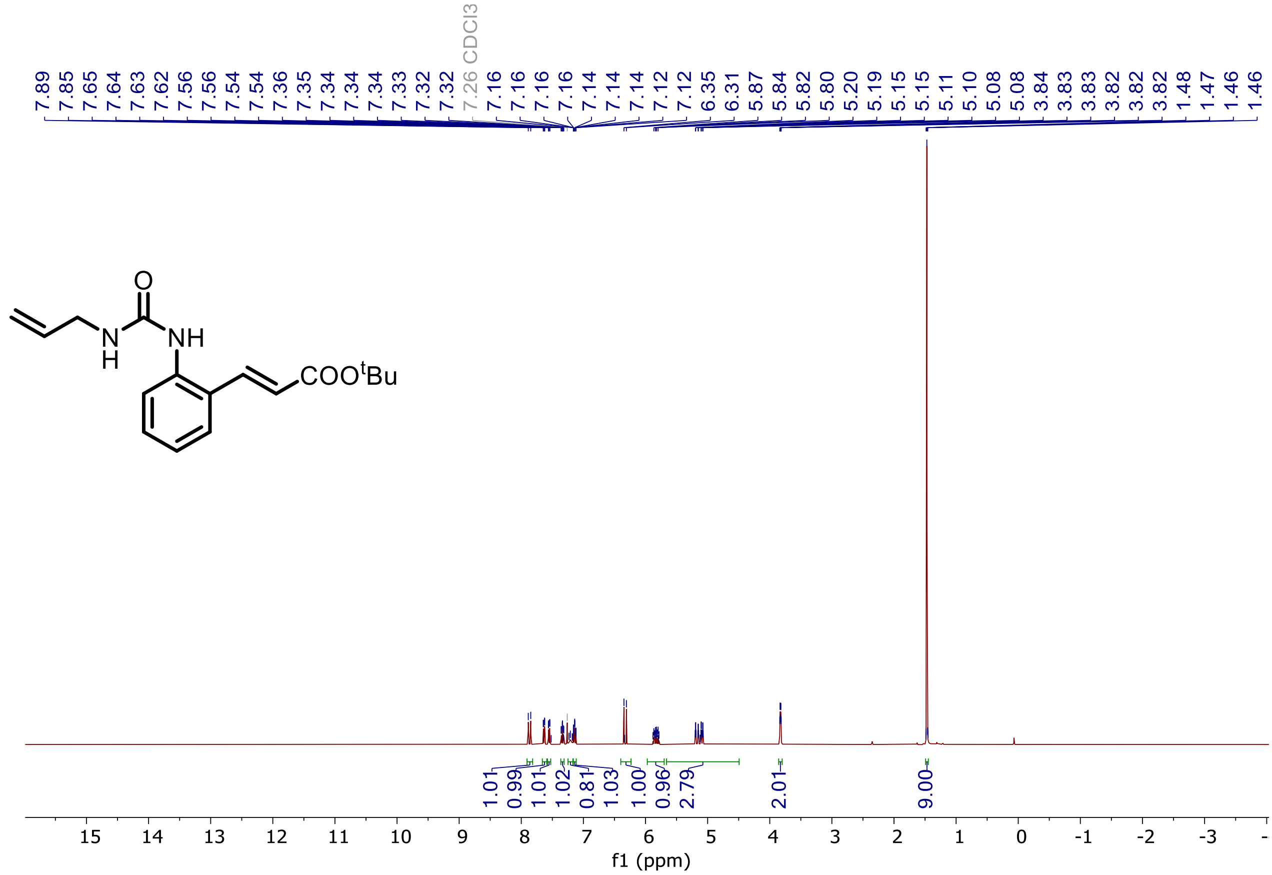

^{13}C NMR: (101 MHz, $CDCl_3$, 298K) of **1z**

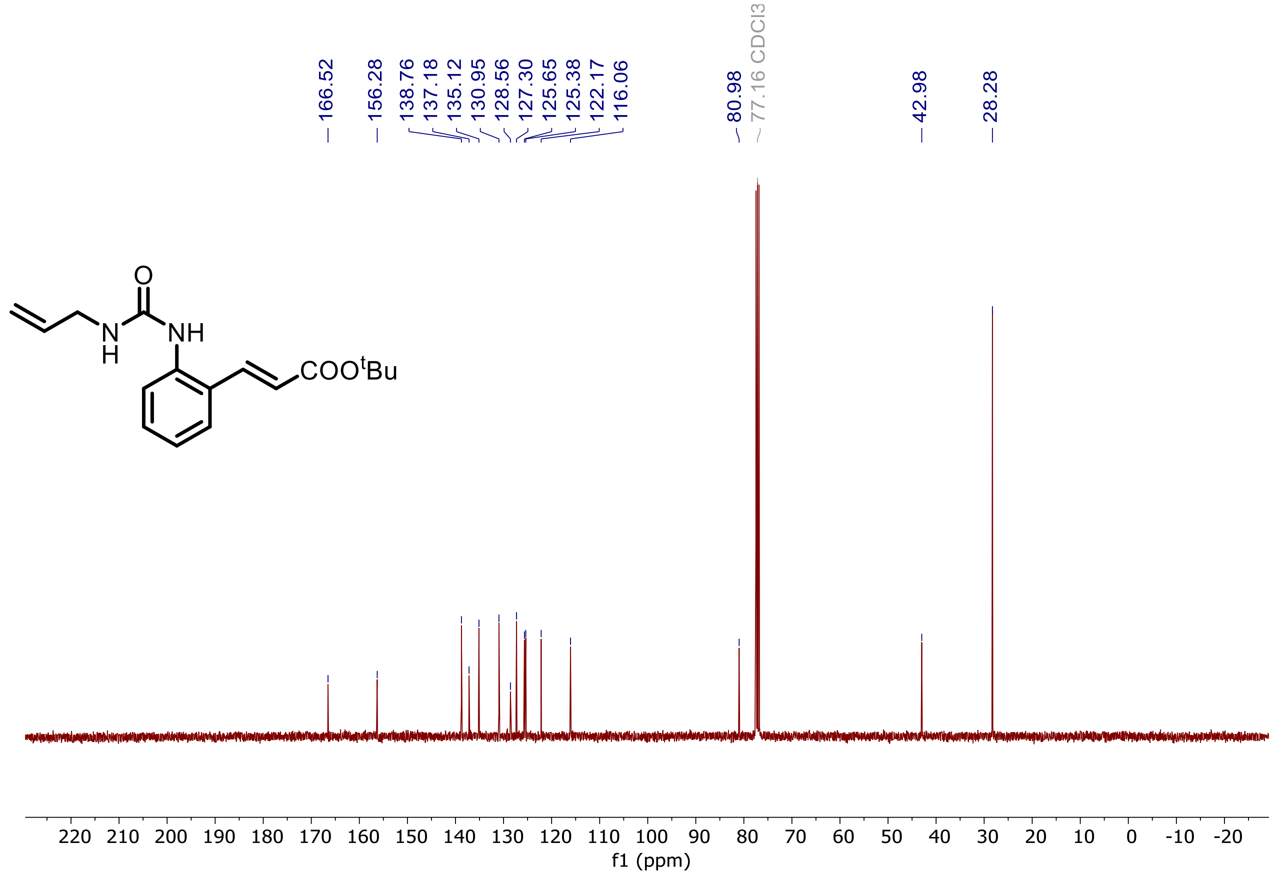

^{1}H NMR: (400 MHz, $CDCl_3$, 298K) of **1aa**

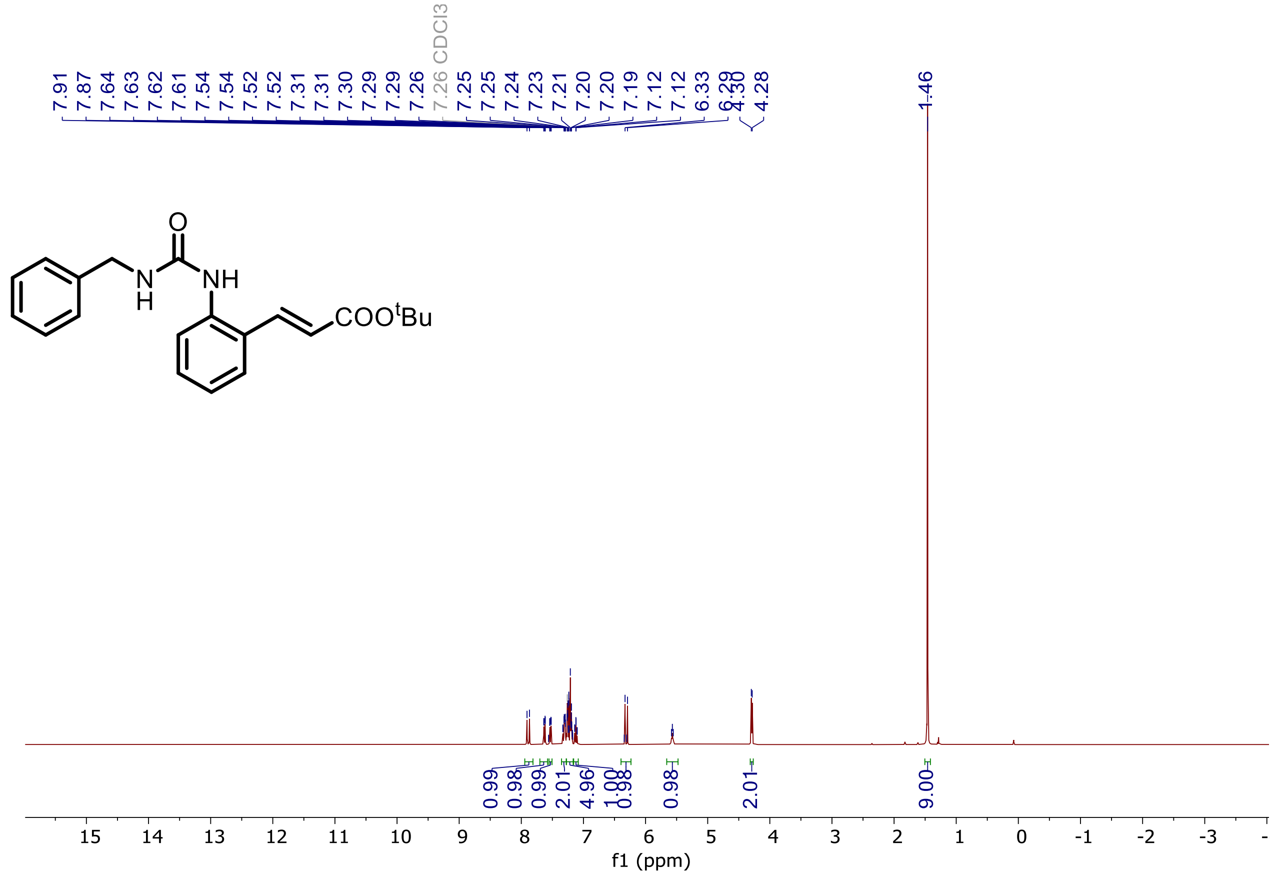

^{13}C NMR: (101 MHz, $CDCl_3$, 298K) of **1aa**

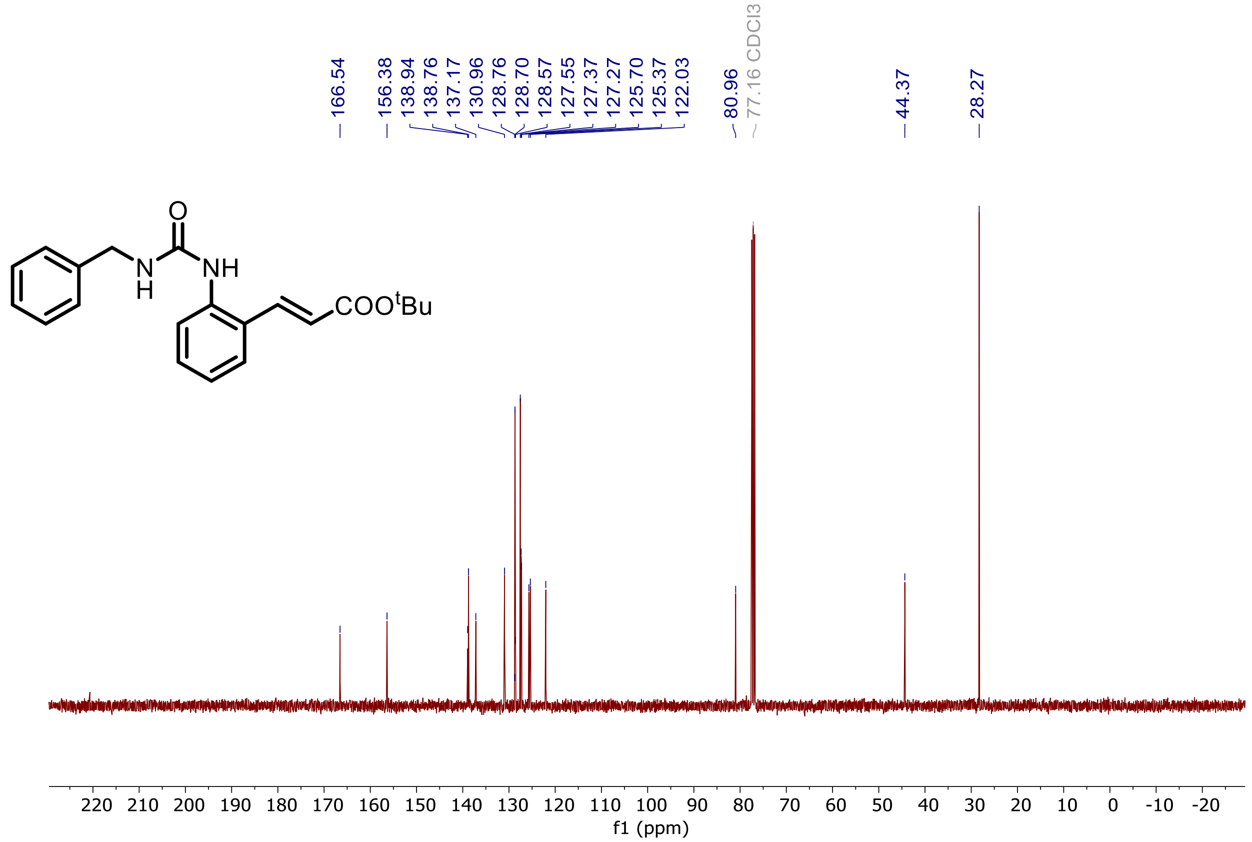

^{1}H NMR: (400 MHz, $CDCl_3$, 298K) of **1ab**

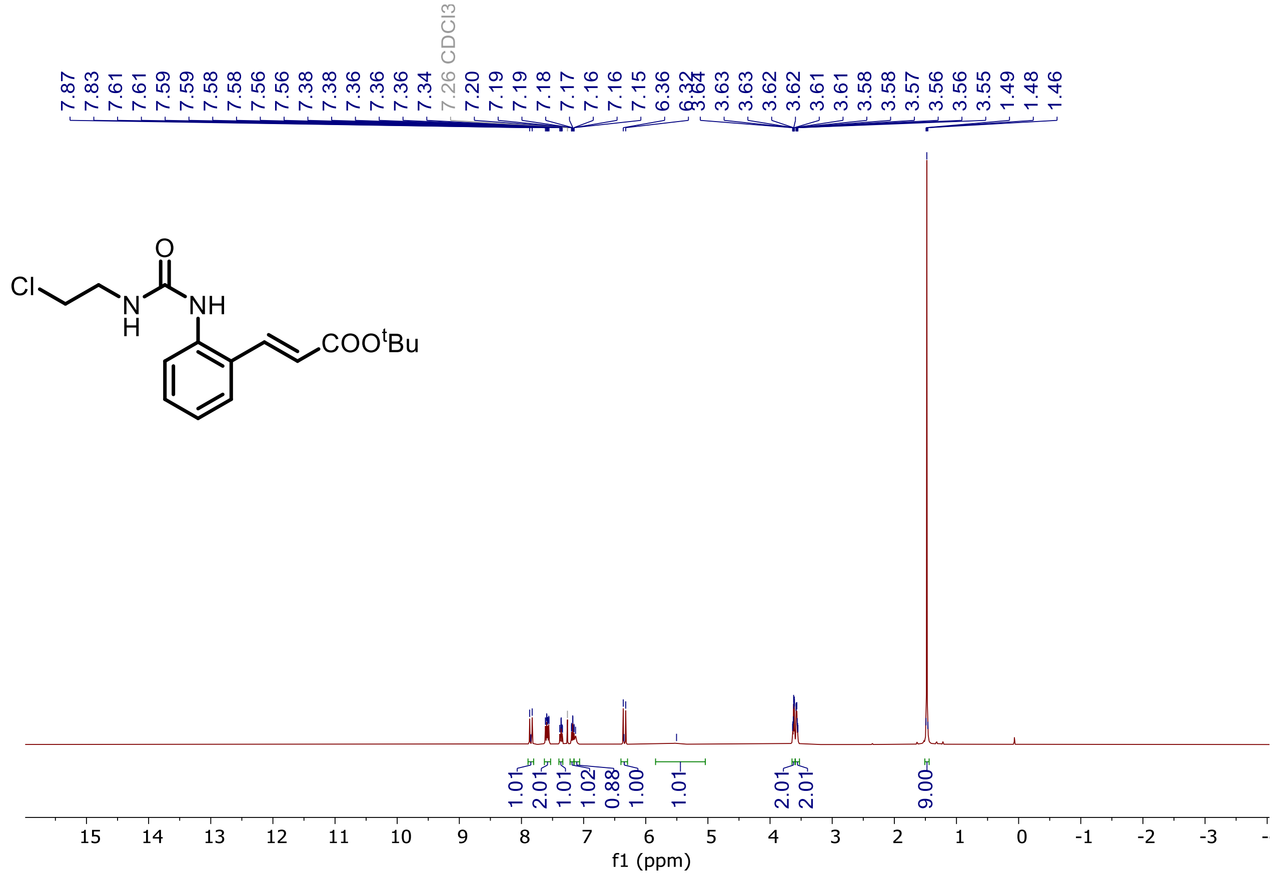

^{13}C NMR: (101 MHz, $CDCl_3$, 298K) of **1ab**

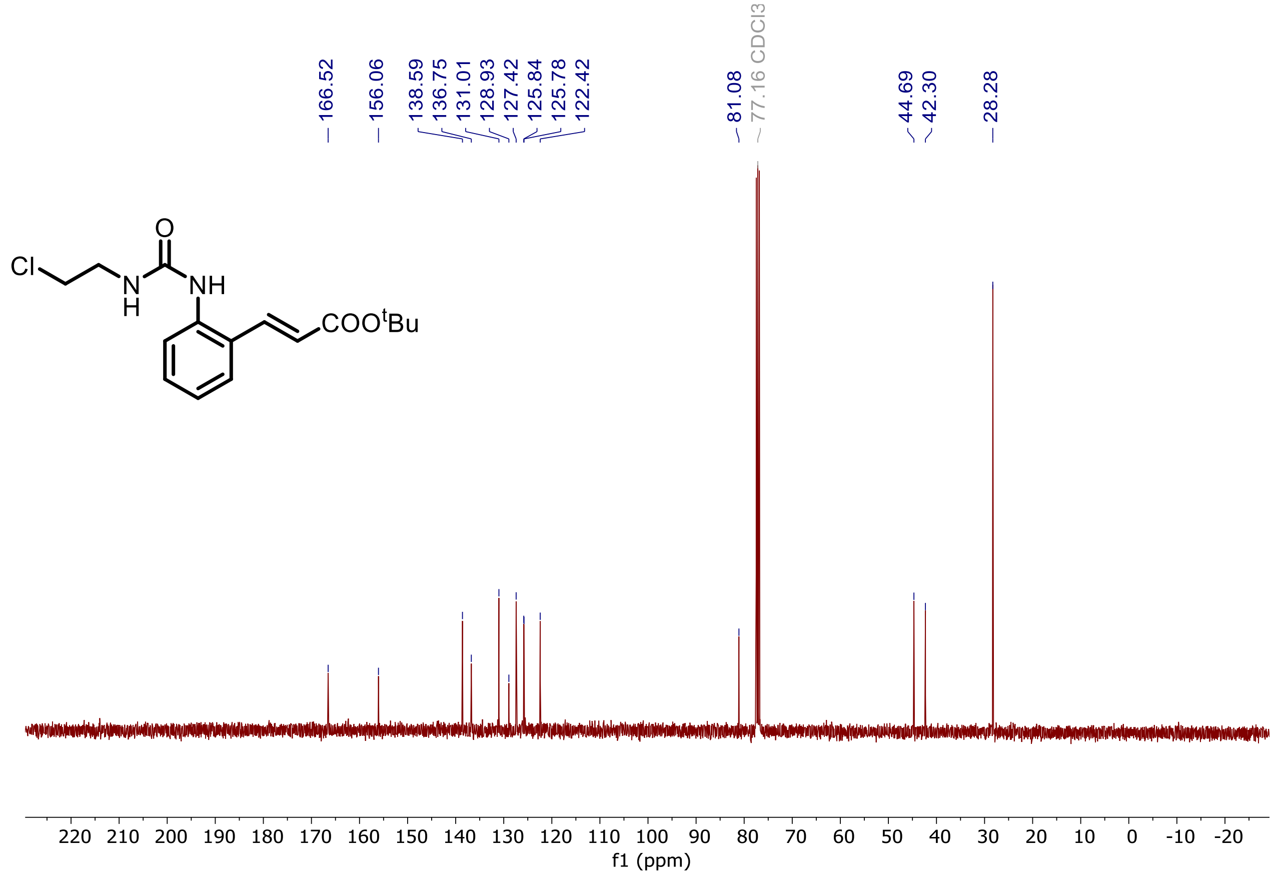

^{1}H NMR: (400 MHz, $CDCl_3$, 298K) of **1ac**

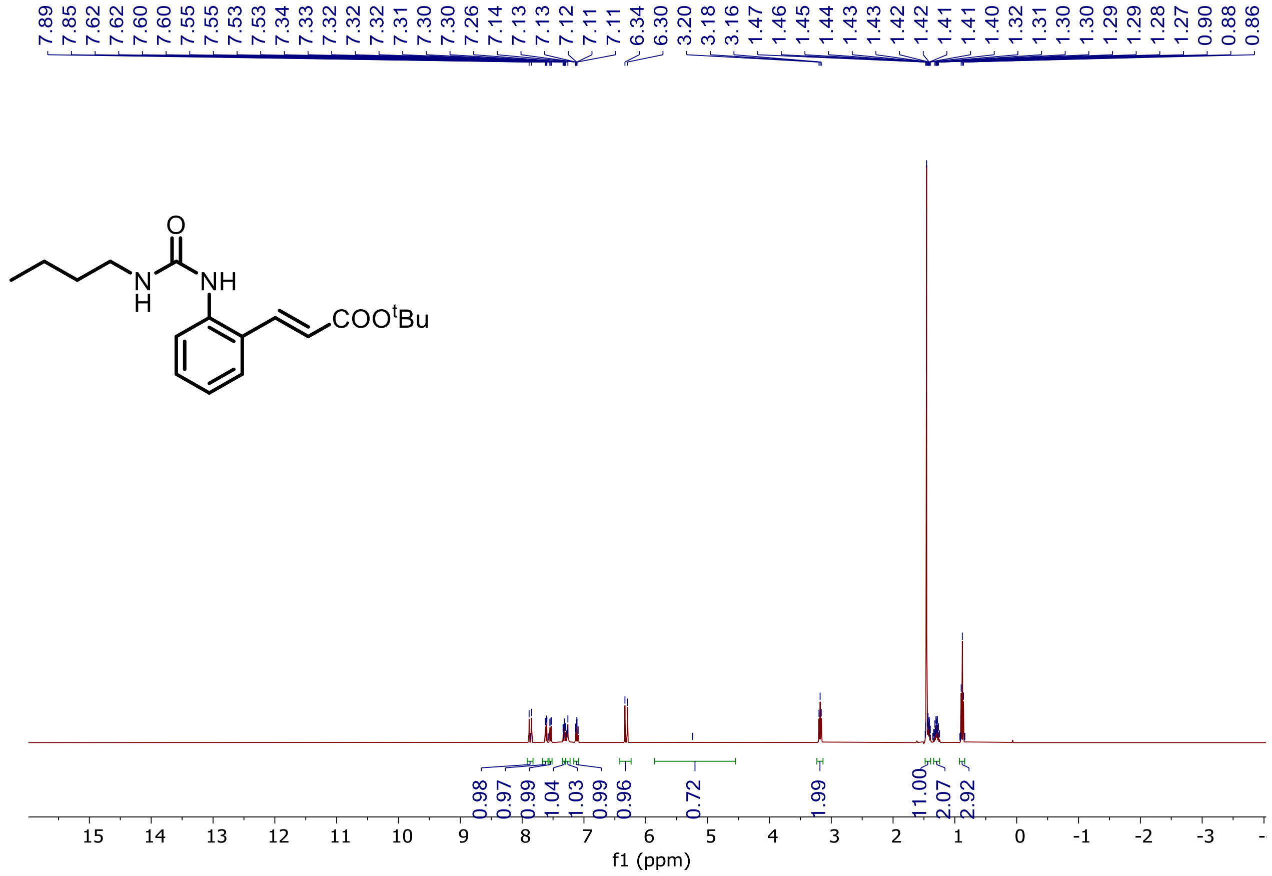

^{13}C NMR: (101 MHz, $CDCl_3$, 298K) of **1ac**

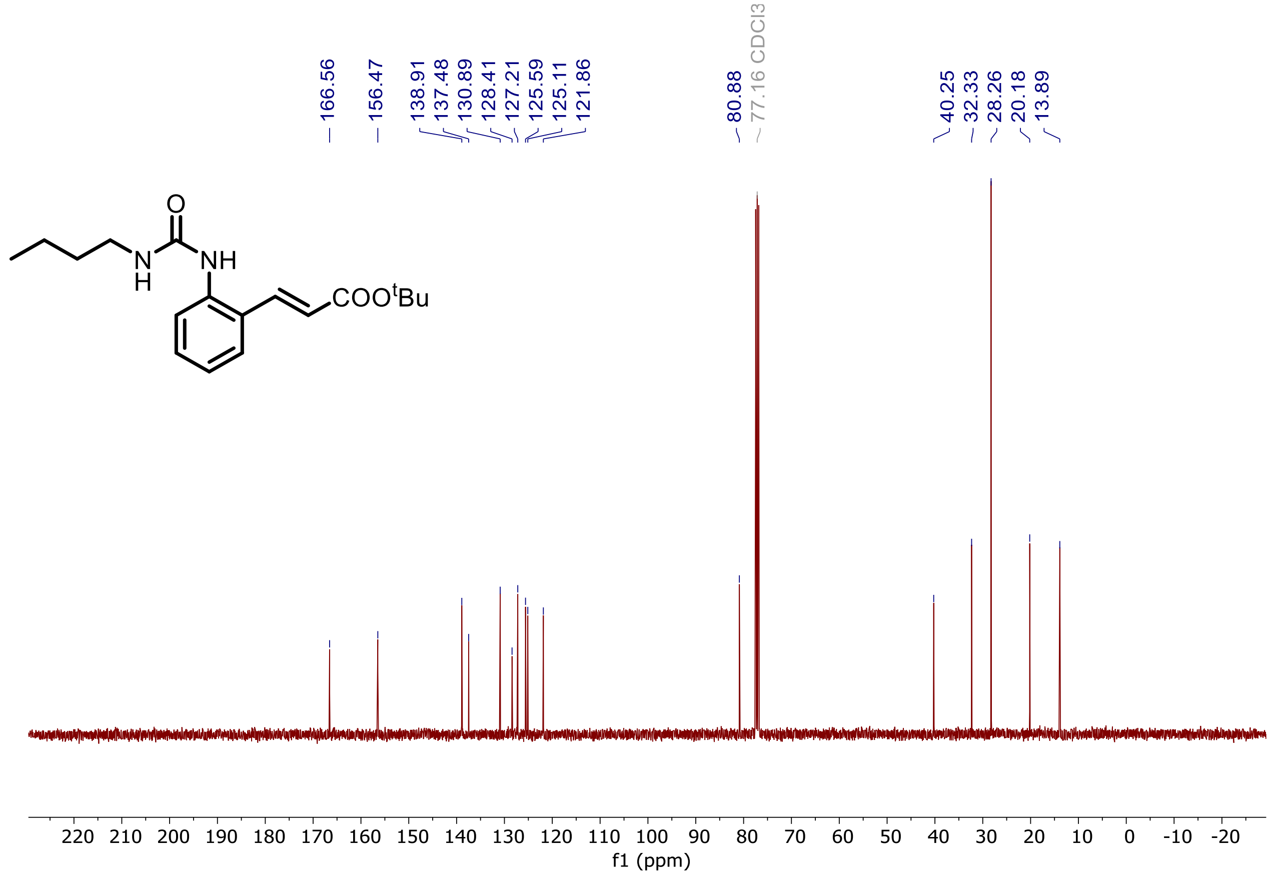

^{1}H NMR: (400 MHz, $(CD_3)_2SO$, 298K) of **1ad**

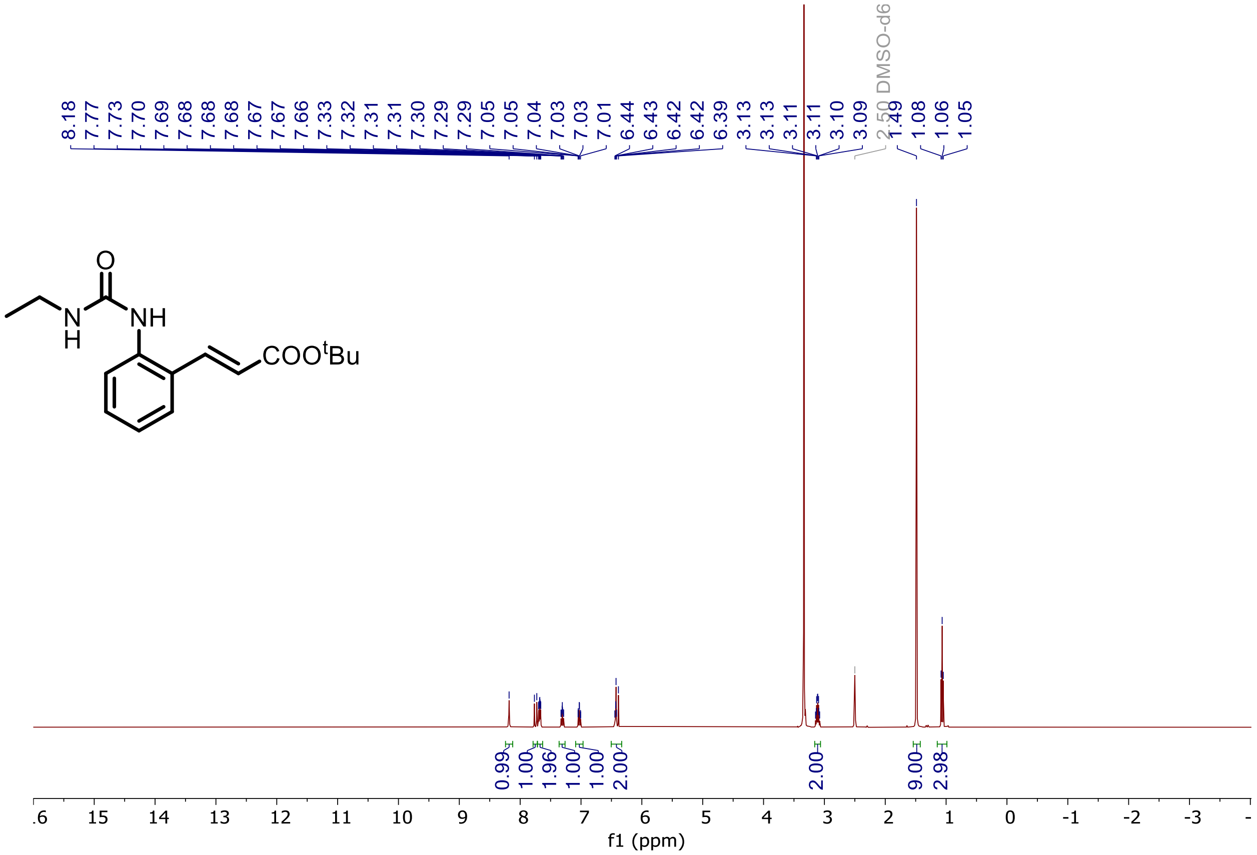

^{13}C NMR: (101 MHz, $(CD_3)_2SO$, 298K) of **1ad**

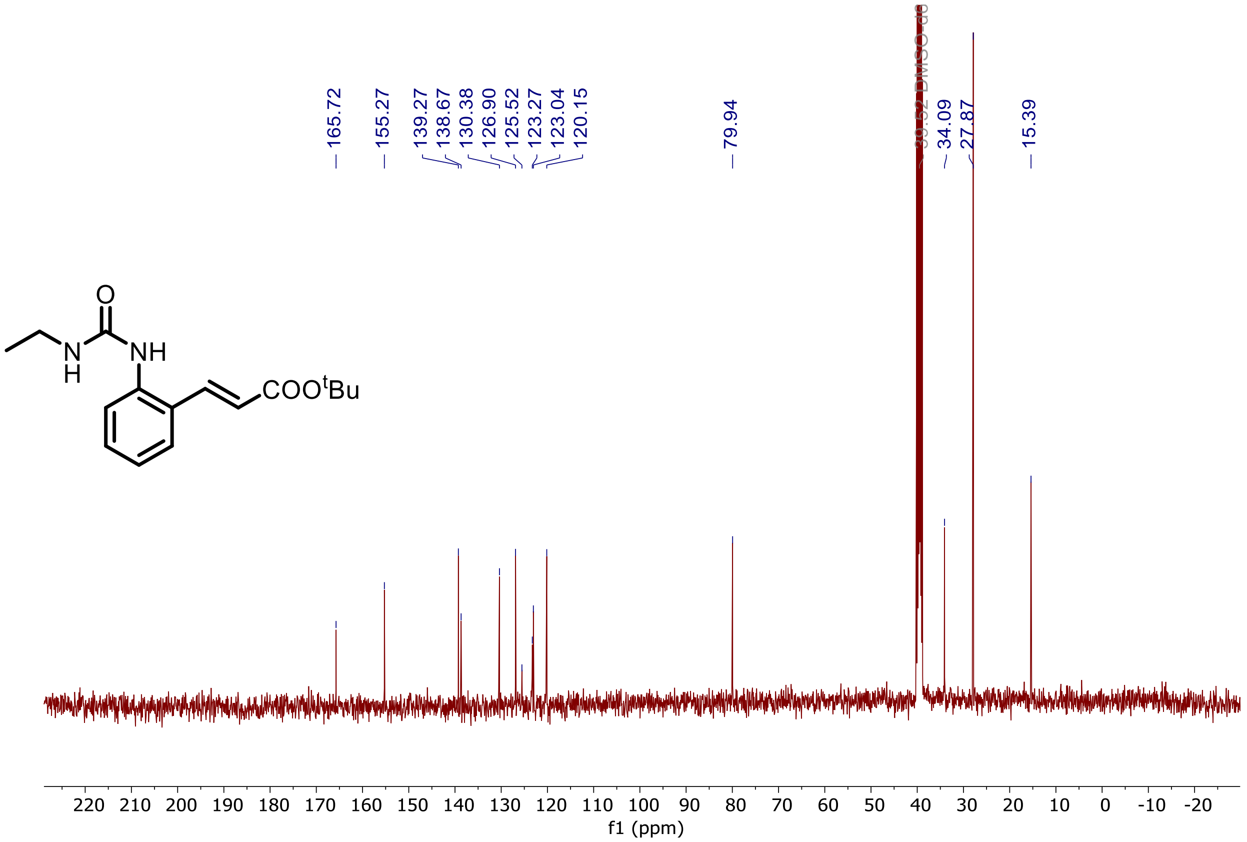

^{1}H NMR: (400 MHz, $CDCl_3$, 298K) of **2a**

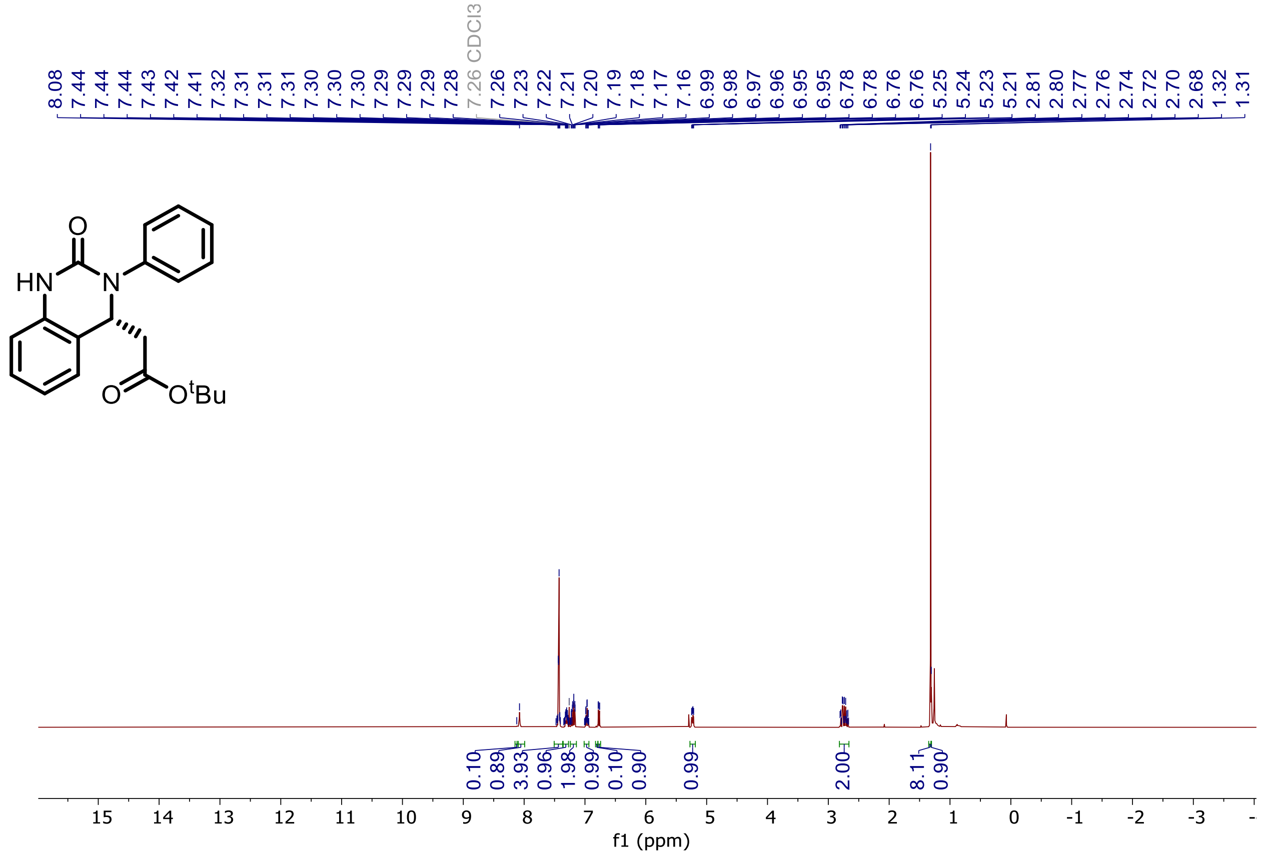

^{13}C NMR: (101 MHz, $CDCl_3$, 298K) of **2a**

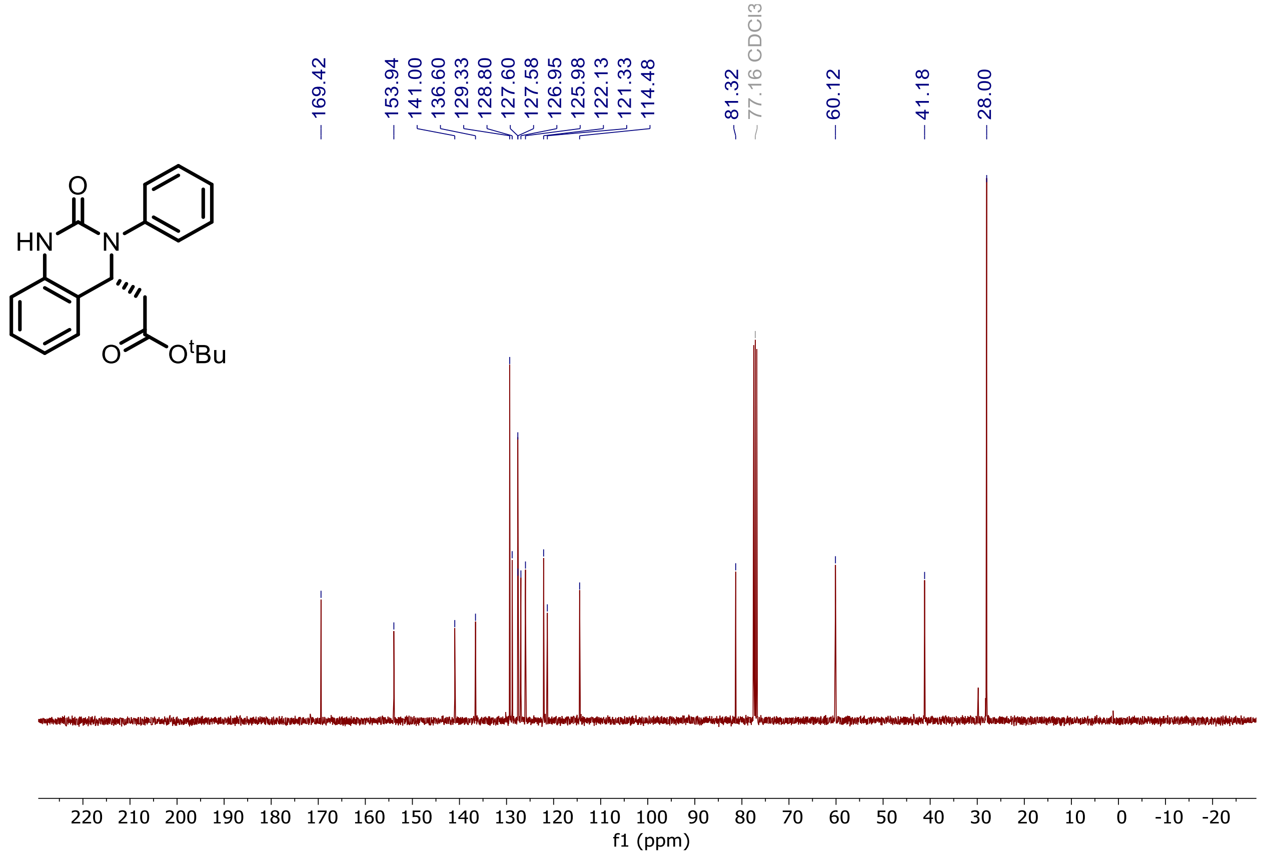

^{1}H NMR: (400 MHz, $CDCl_3$, 298K) of **2b**

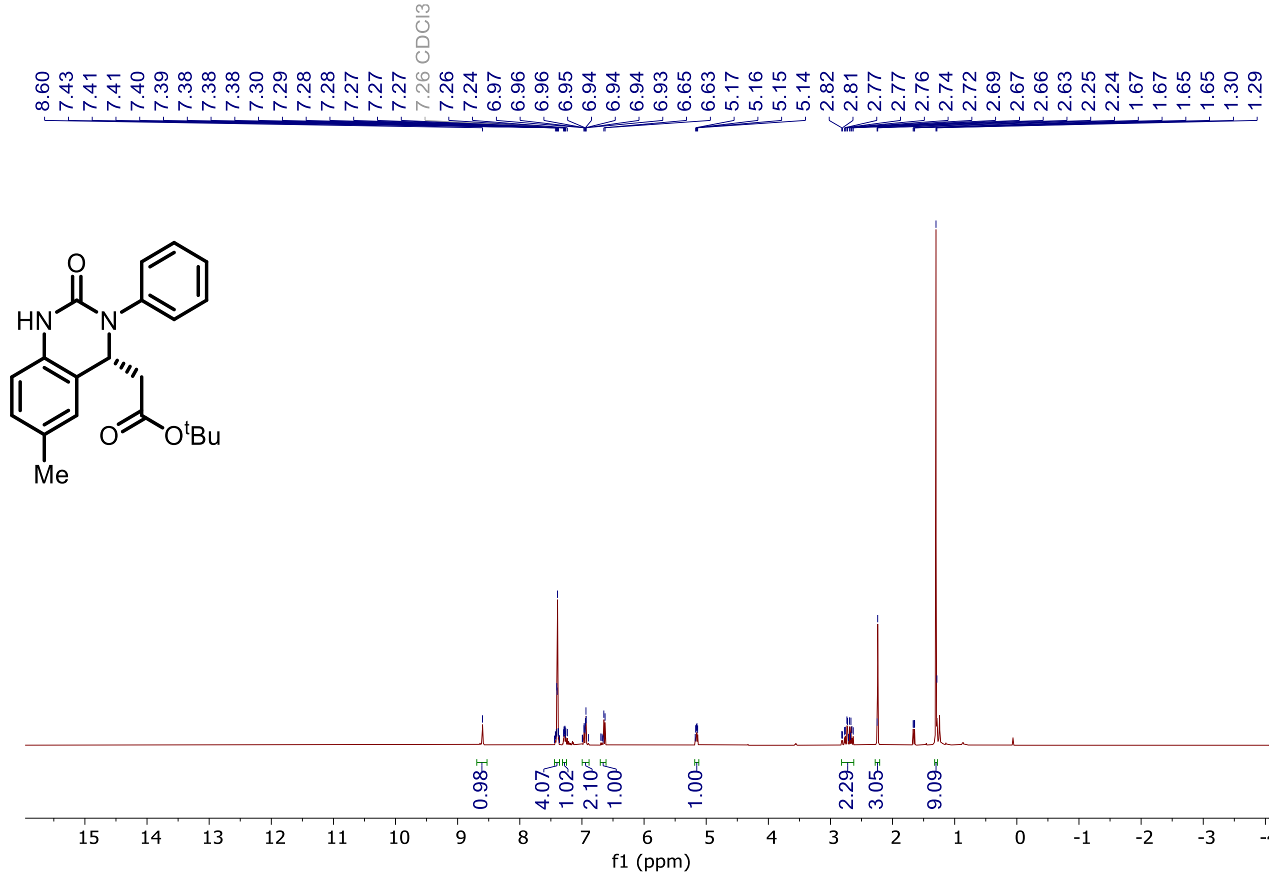

^{13}C NMR: (101 MHz, $CDCl_3$, 298K) of **2b**

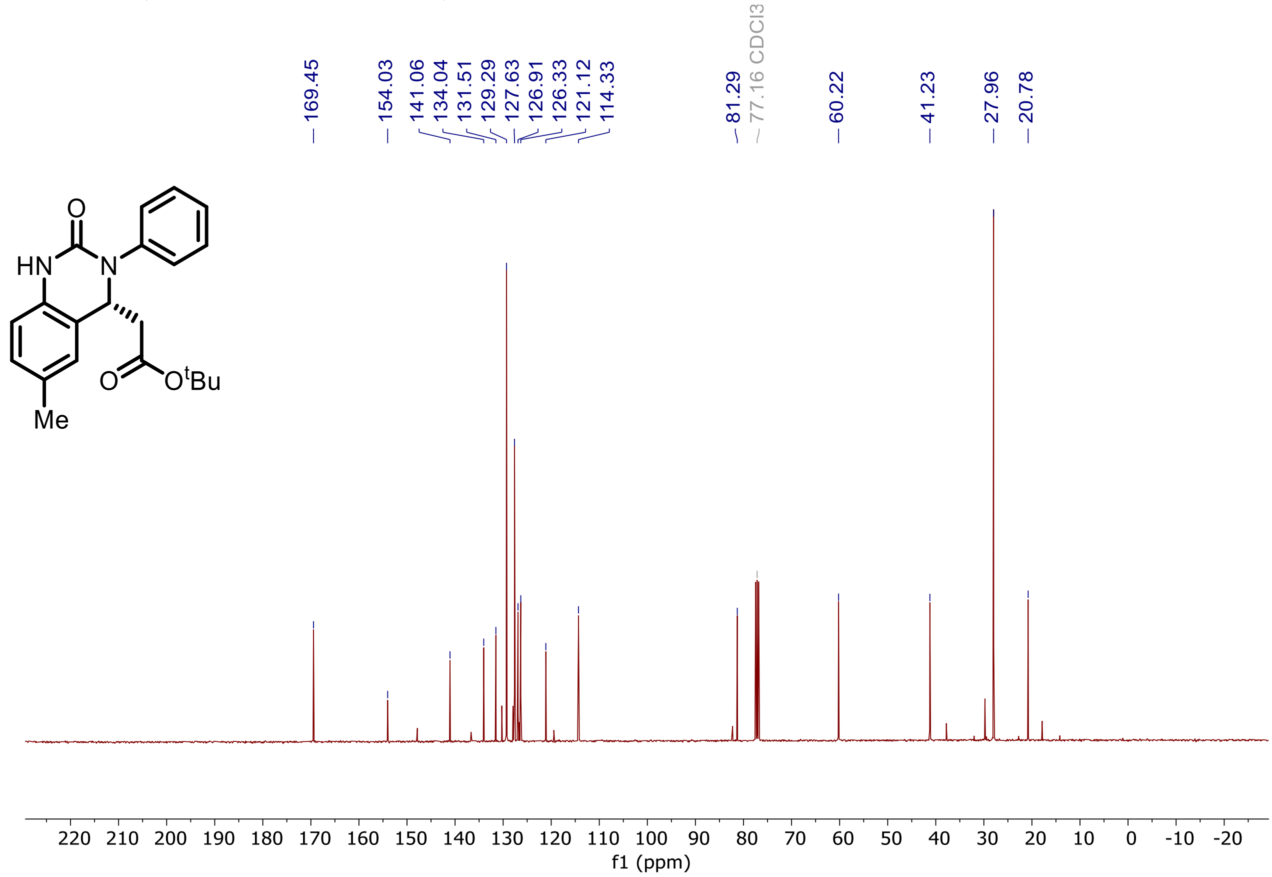

^{1}H NMR: (500 MHz, $CDCl_3$, 298K) of **2c**

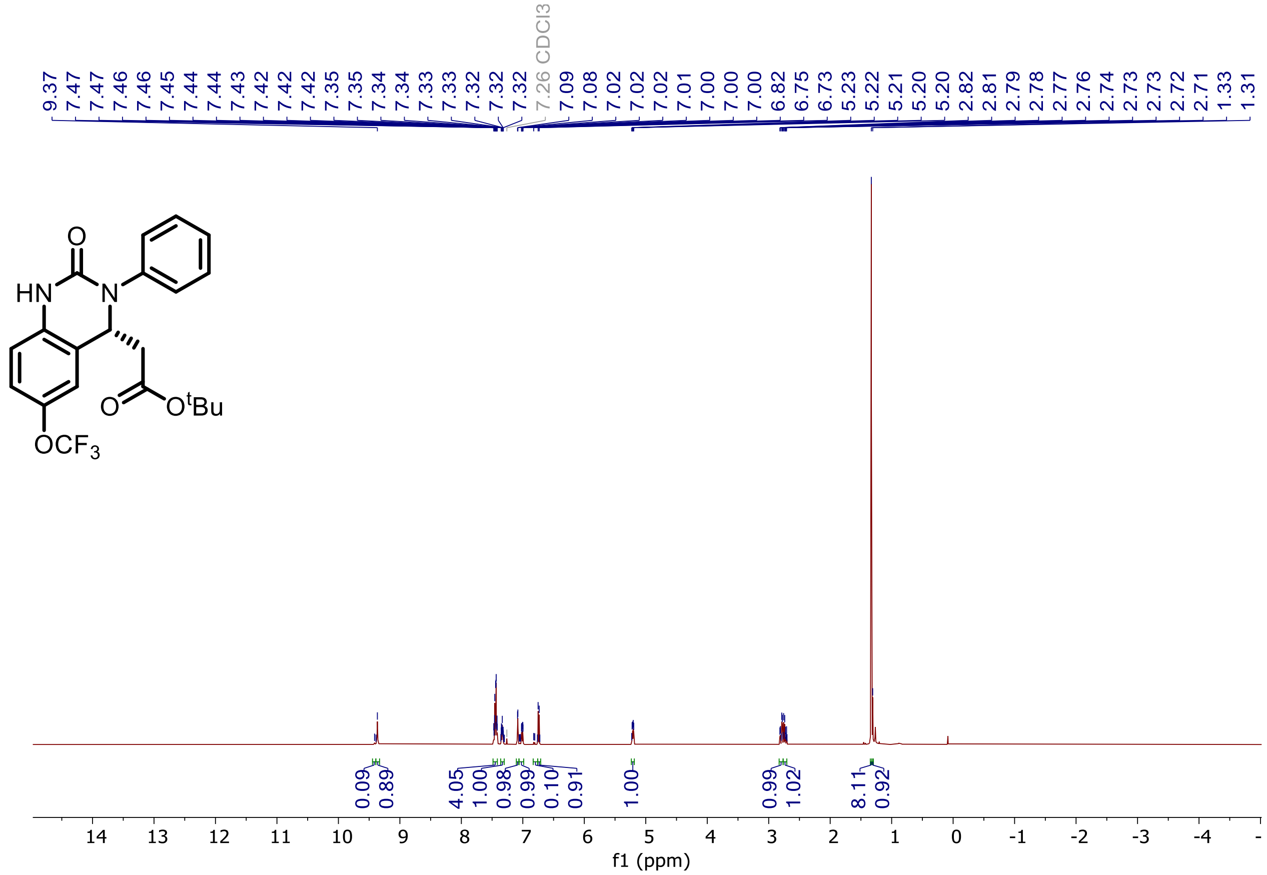

^{13}C NMR: (126 MHz, $CDCl_3$, 298K) of **2c**

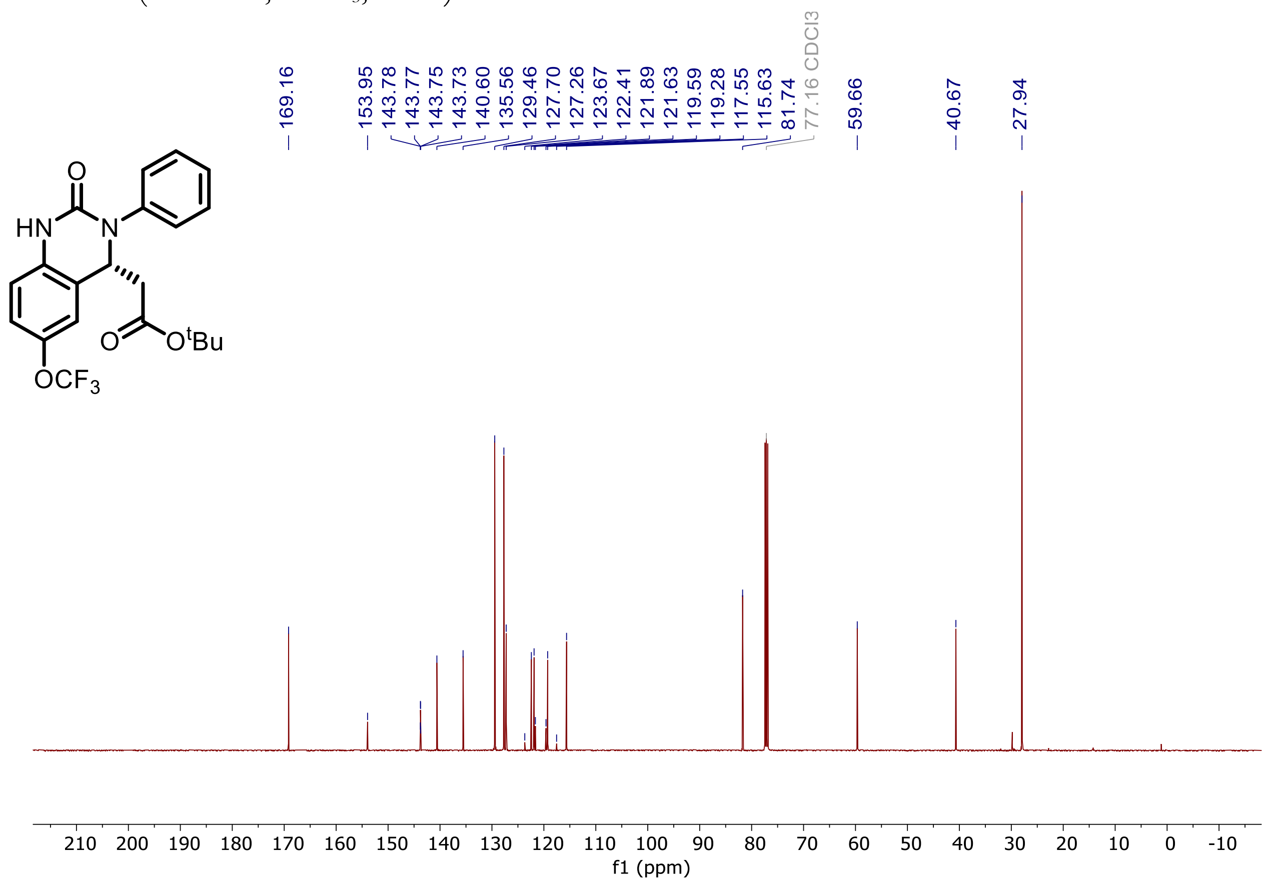

^{19}F NMR: (377 MHz, $CDCl_3$, 298K) of **2c**

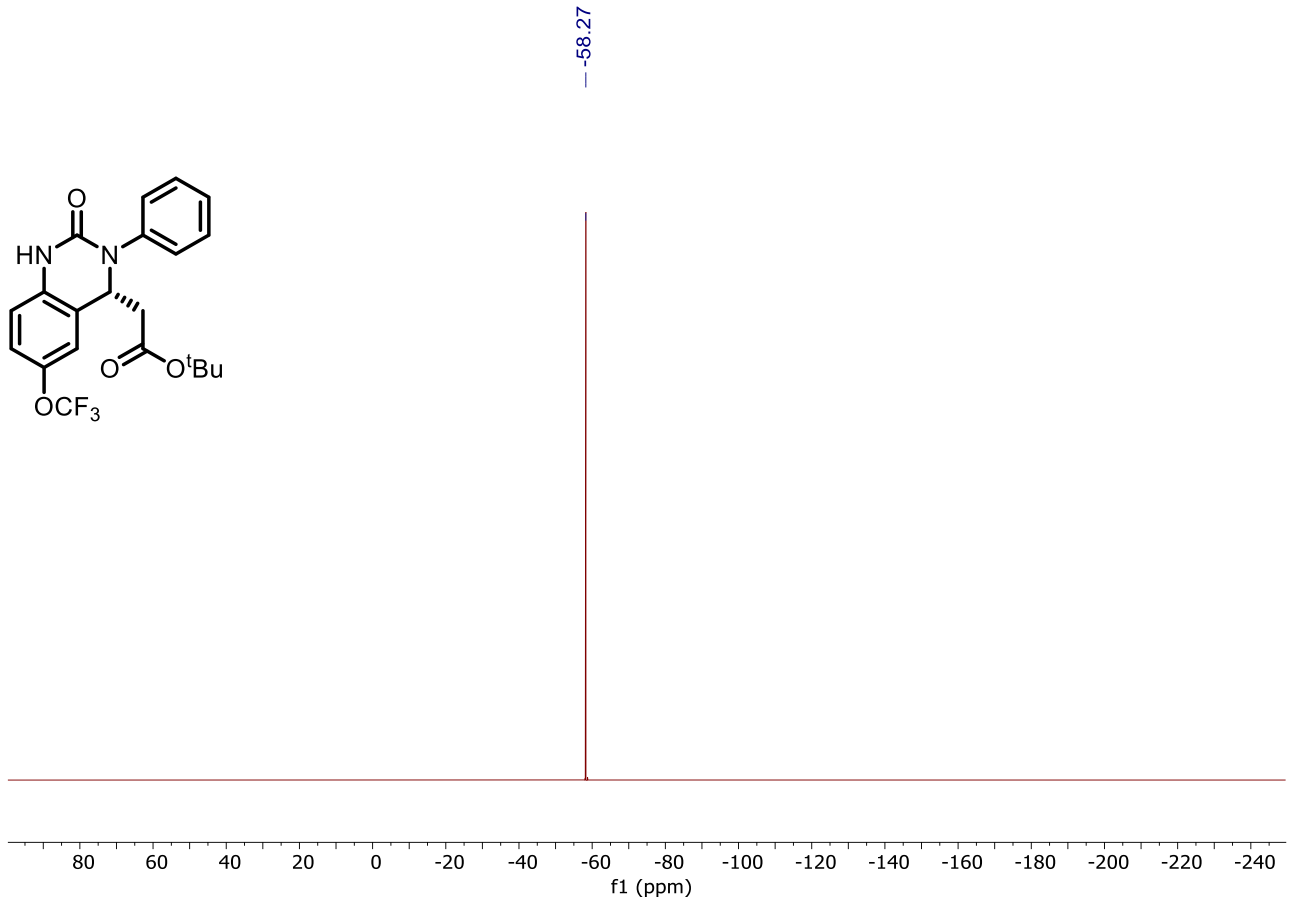

^{1}H NMR: (400 MHz, $CDCl_3$, 298K) of **2d**

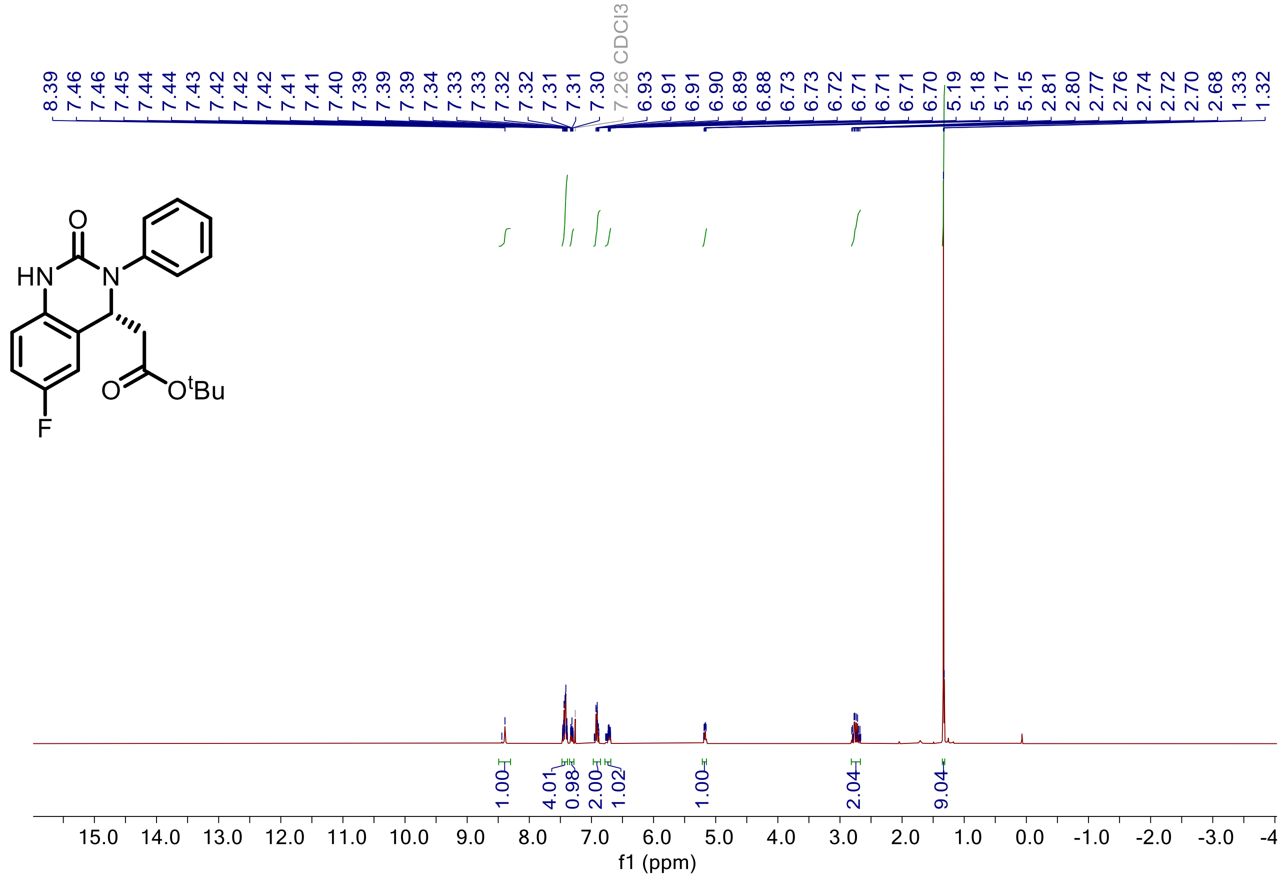

^{13}C NMR: (101 MHz, $CDCl_3$, 298K) of **2d**

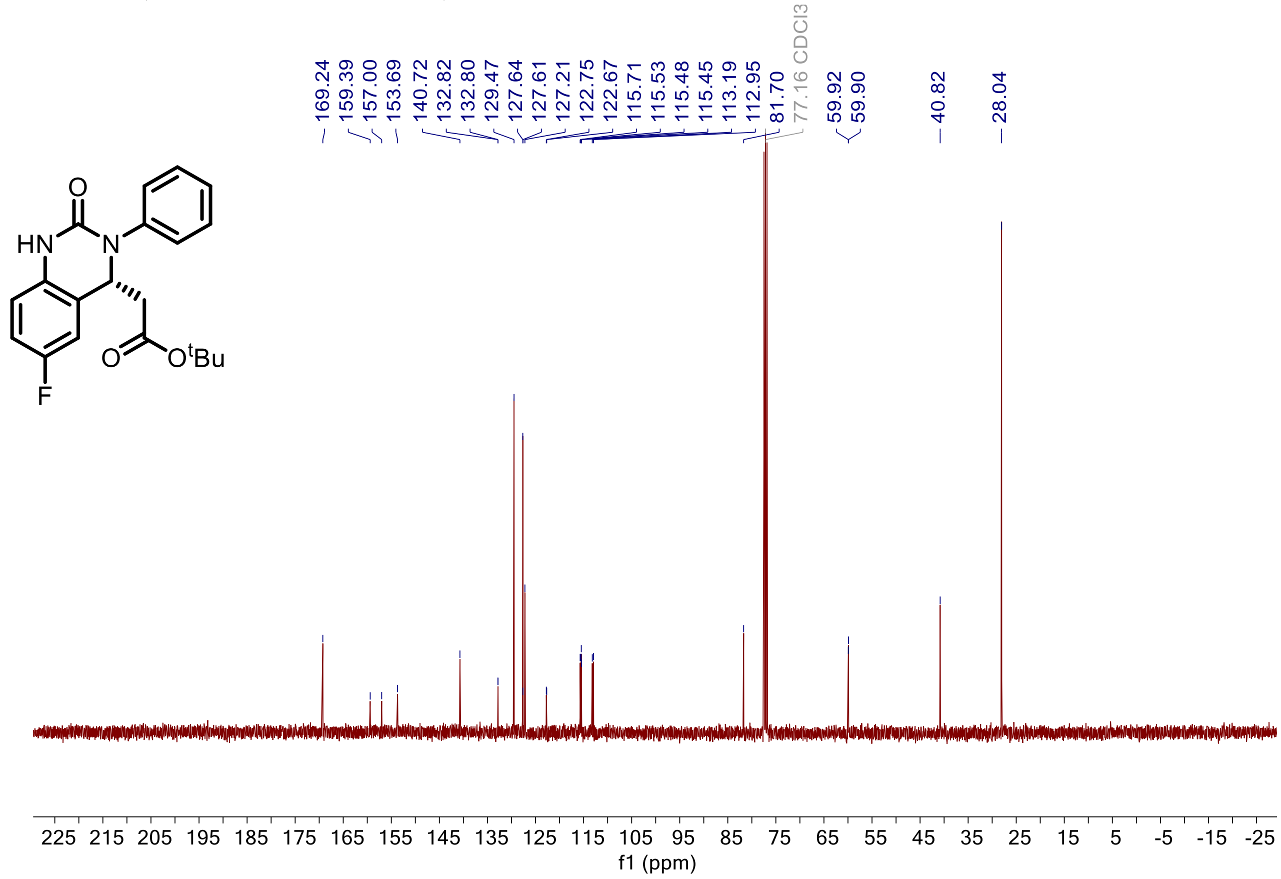

^{19}F NMR: (377 MHz, $CDCl_3$, 298K) of **2d**

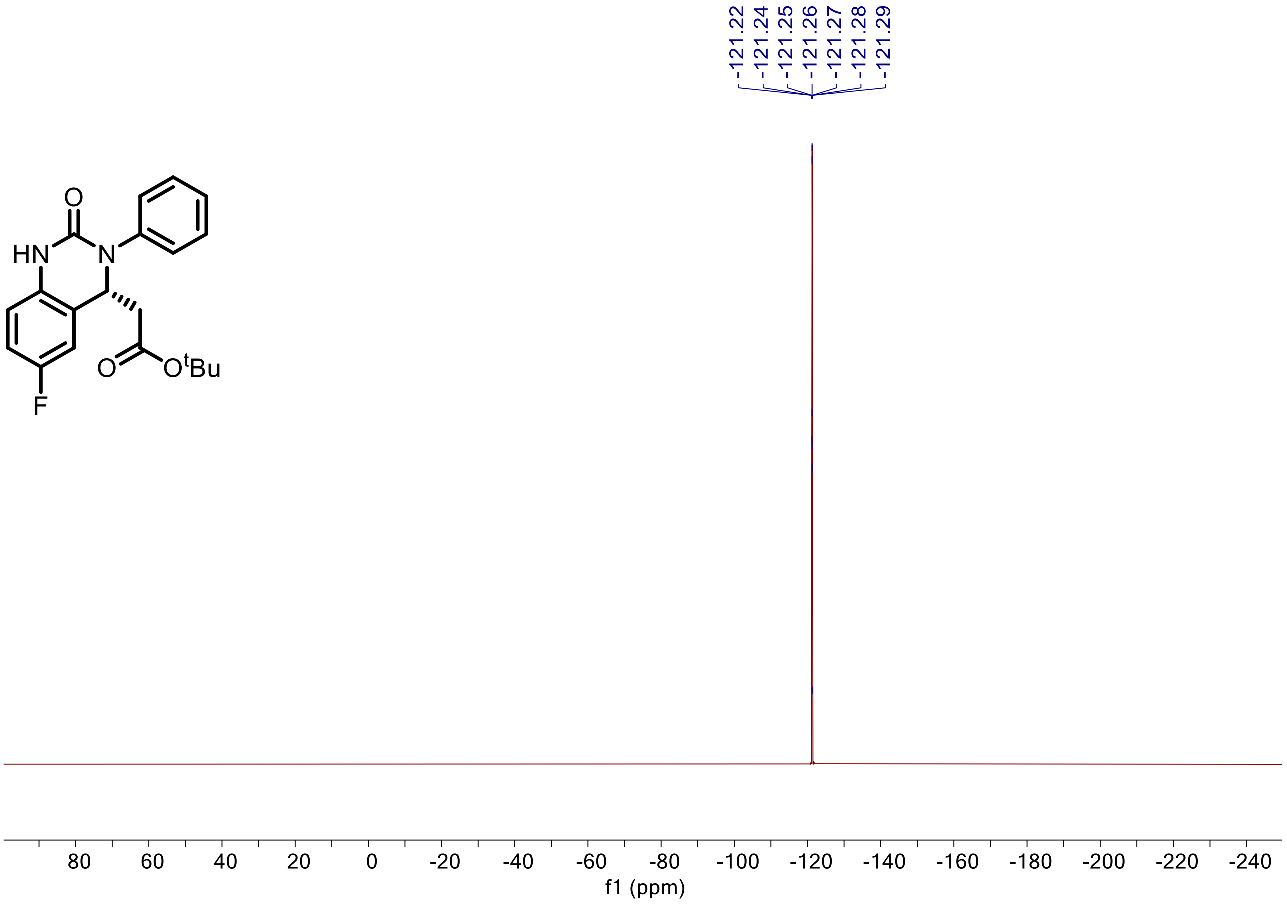

^{1}H NMR: (400 MHz, $CDCl_3$, 298K) of **2e**

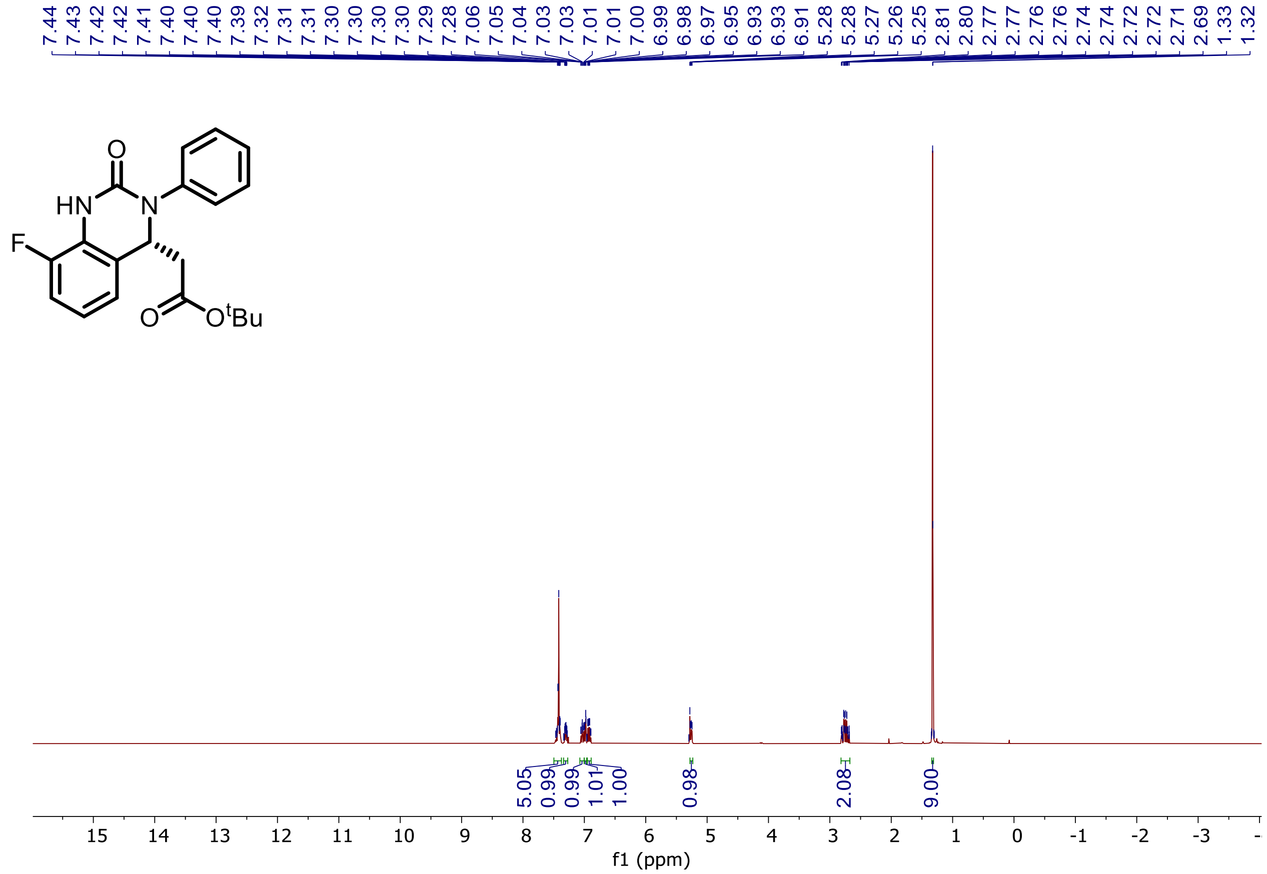

^{13}C NMR: (101 MHz, $CDCl_3$, 298K) of **2e**

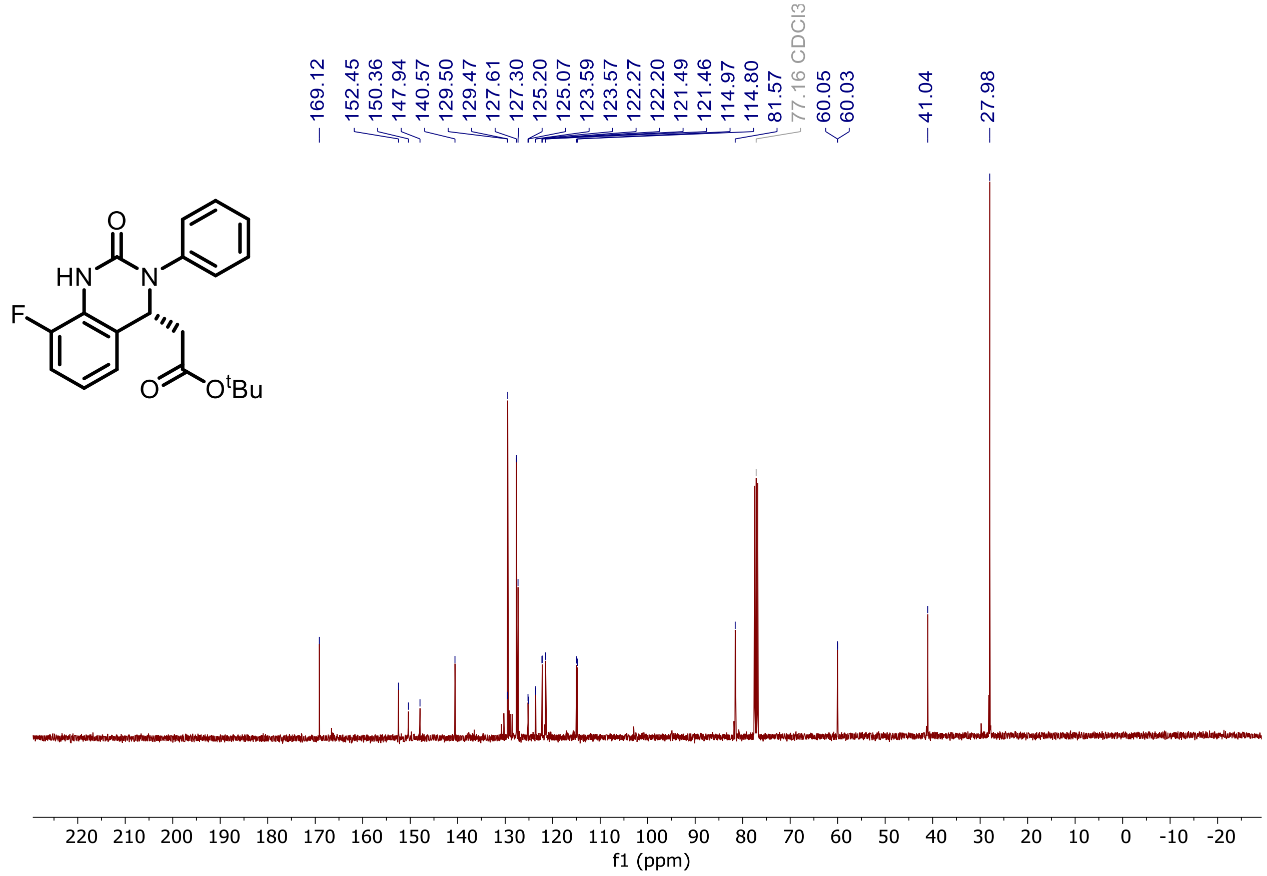

^{1}H NMR: (400 MHz, $CDCl_3$, 298K) of **2f**

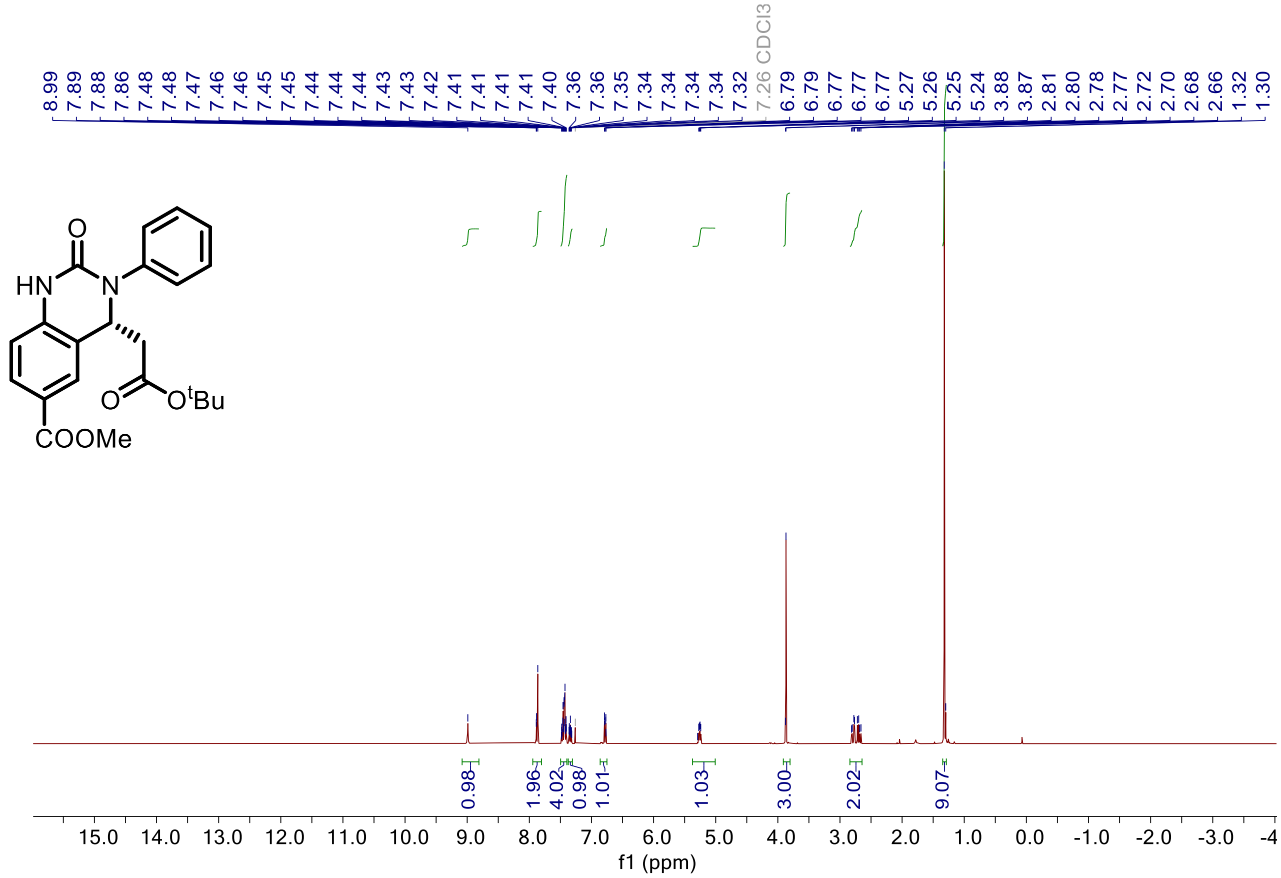

^{13}C NMR: (101 MHz, $CDCl_3$, 298K) **2f**

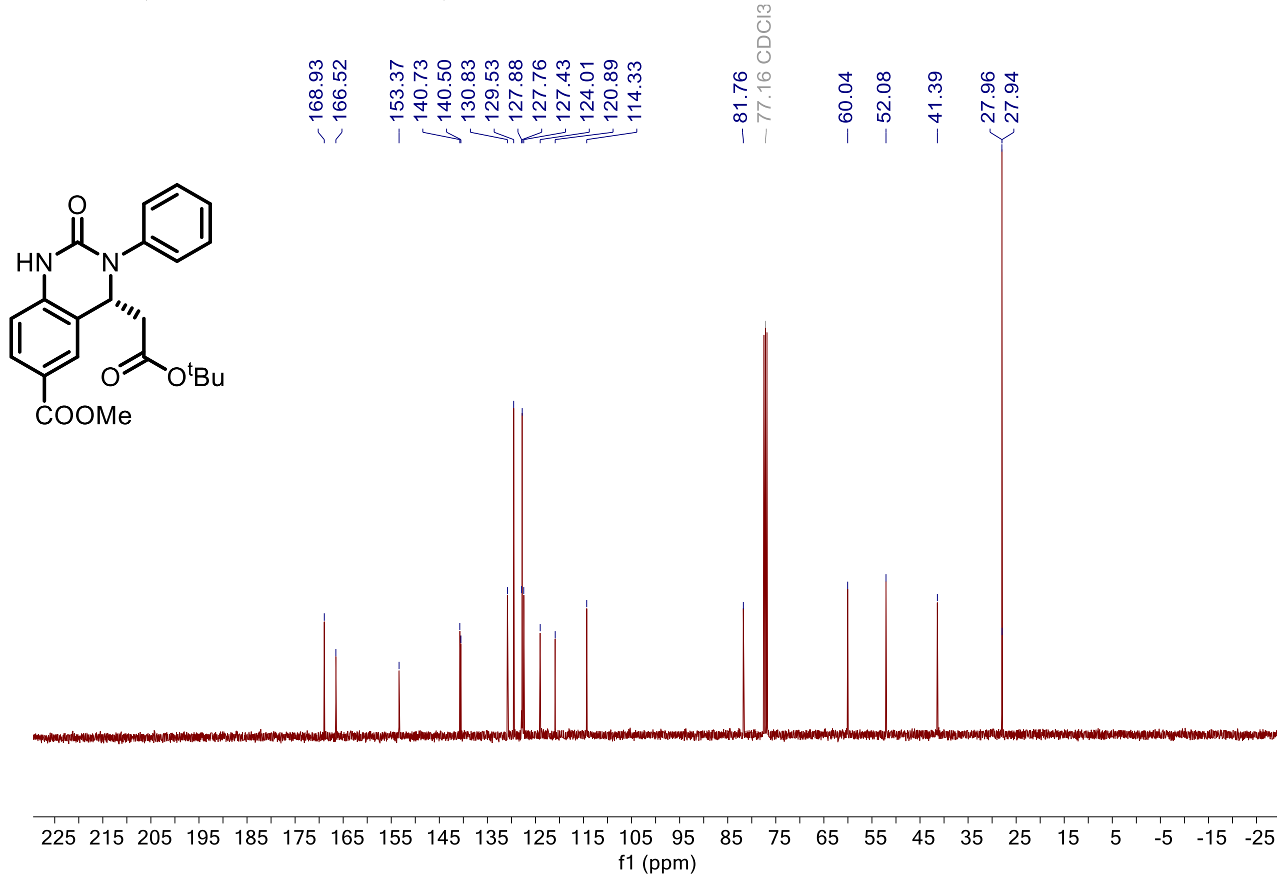

^{1}H NMR: (400 MHz, $CDCl_3$, 298K) of **2g**

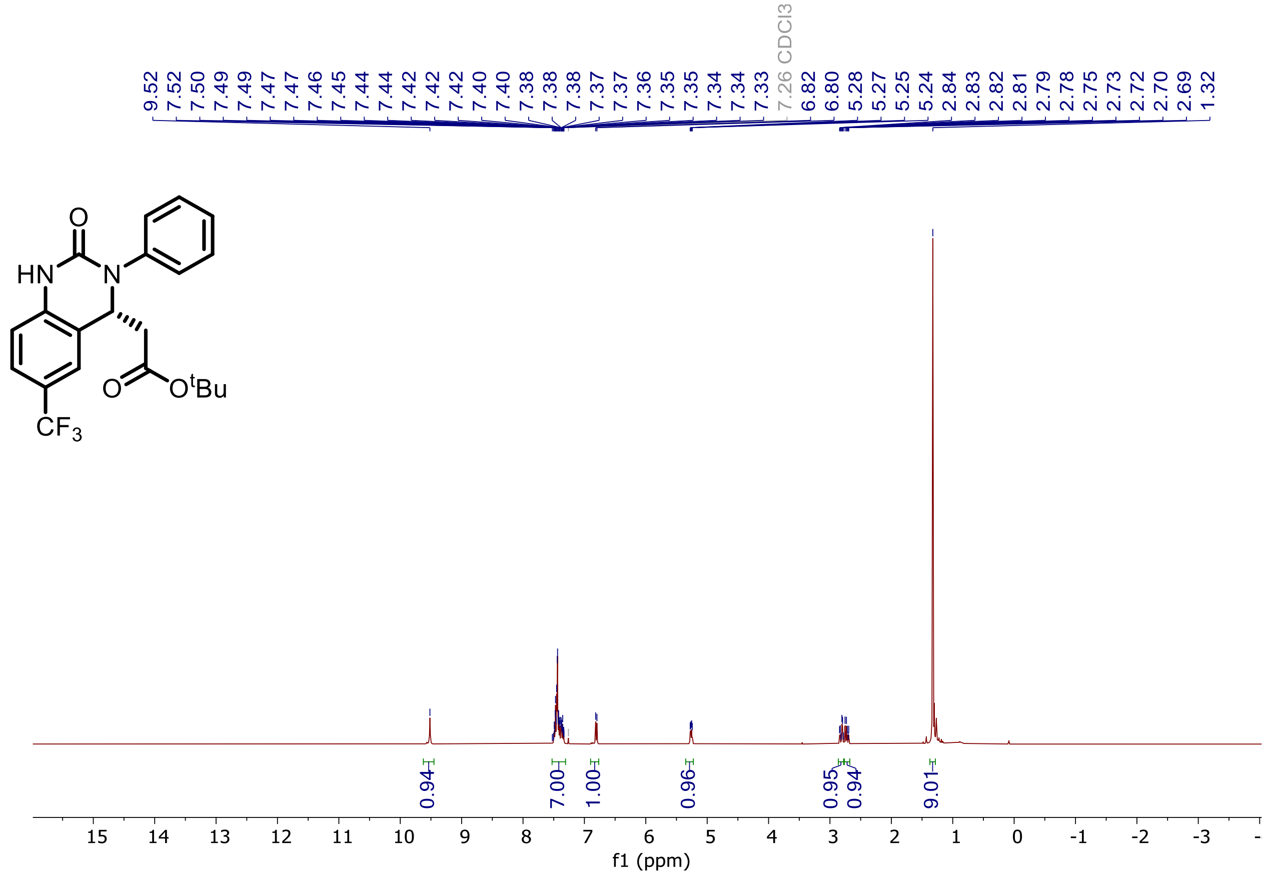

^{13}C NMR: (101 MHz, $CDCl_3$, 298K) **2g**

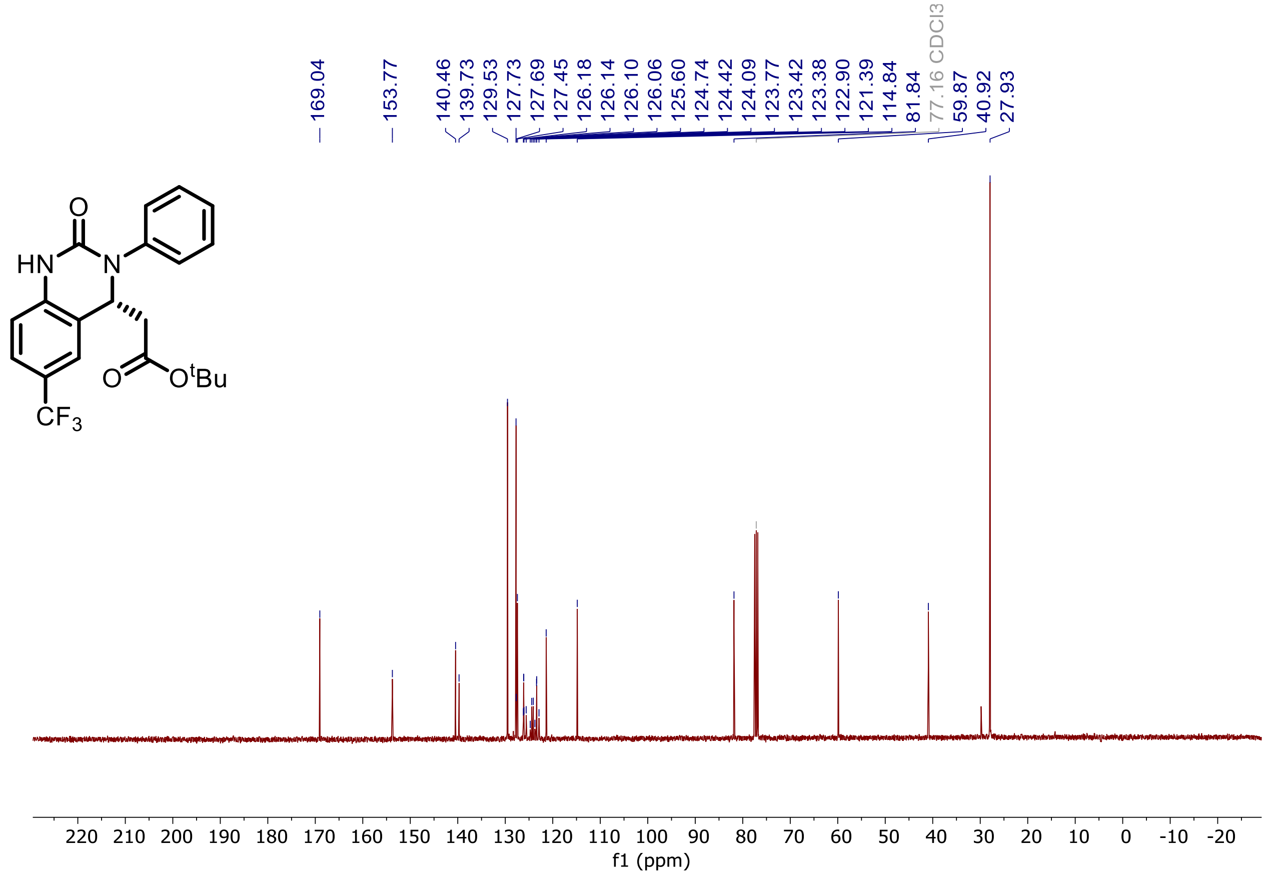

^{1}H NMR: (400 MHz, $CDCl_3$, 298K) of **2h**

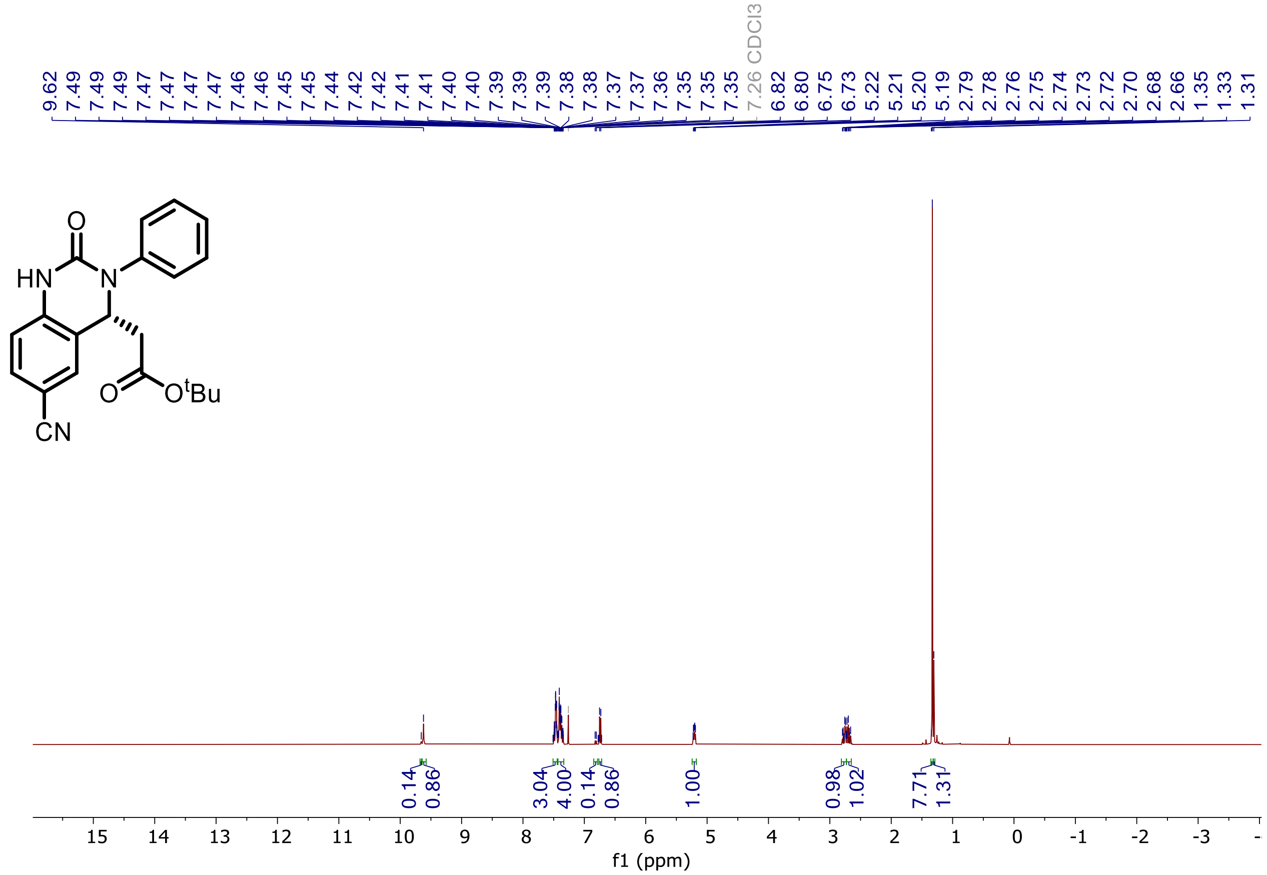

^{13}C NMR: (101 MHz, $CDCl_3$, 298K) of **2h**

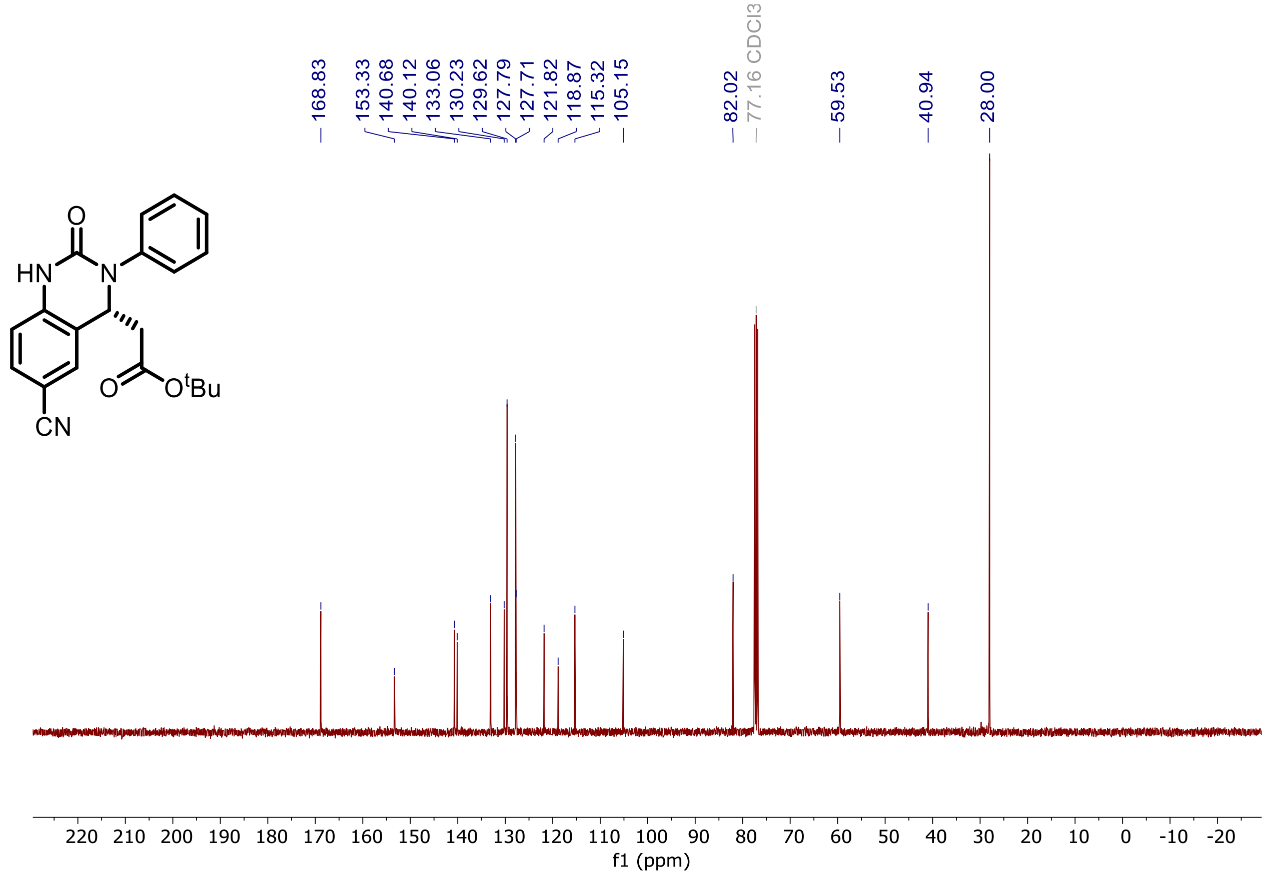

^{1}H NMR: (400 MHz, $CDCl_3$, 298K) of **2i**

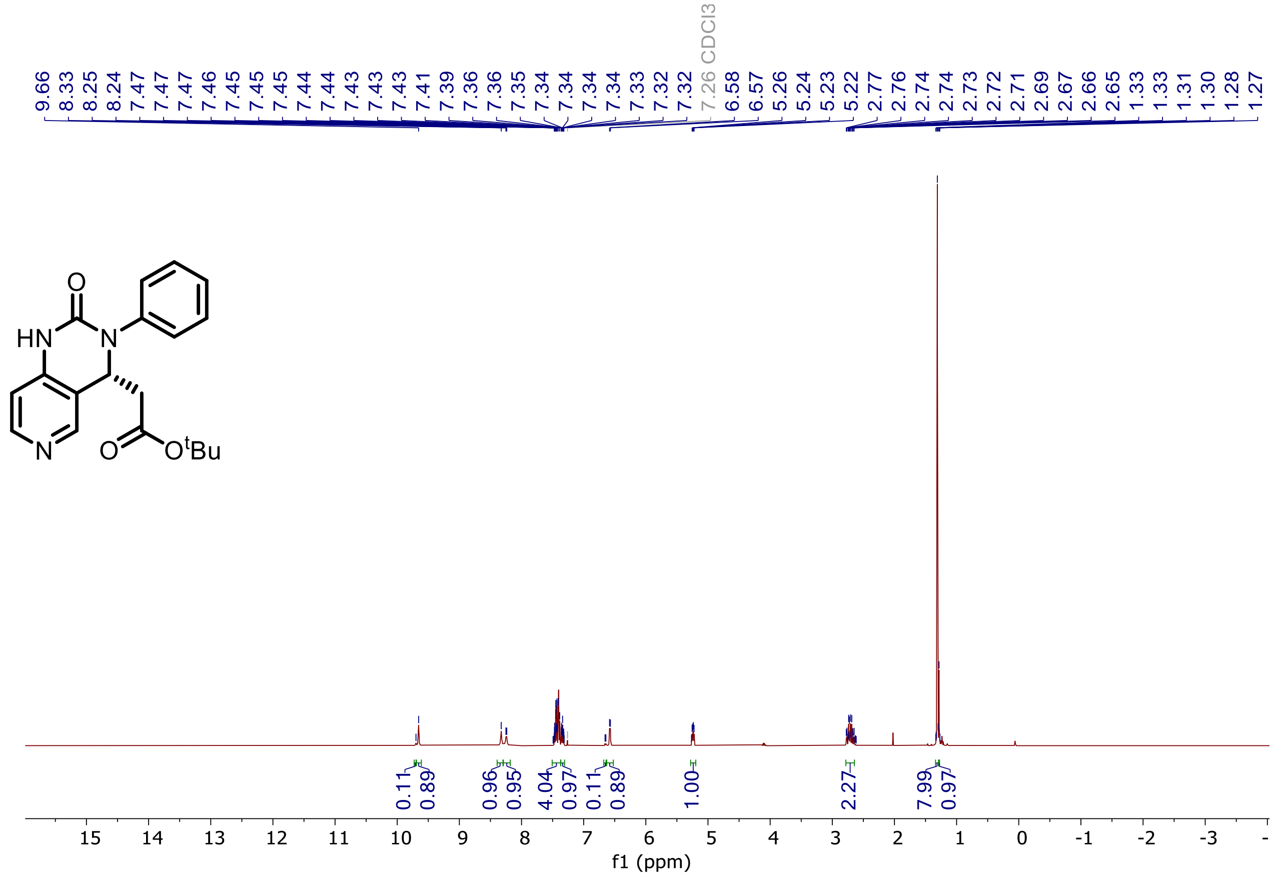

^{13}C NMR: (101 MHz, $CDCl_3$, 298K) of **2i**

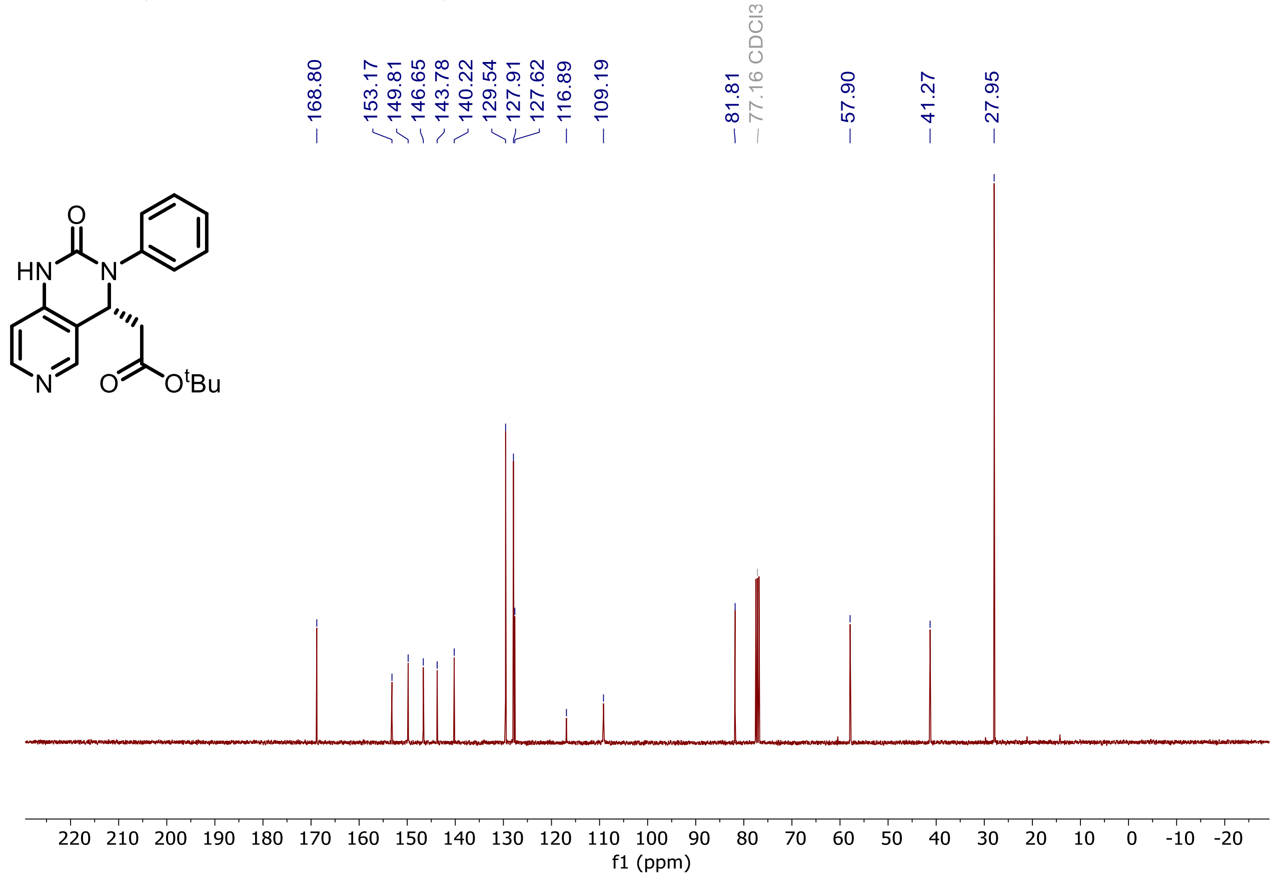

^{1}H NMR: (400 MHz, $CDCl_3$, 298K) of **2j**

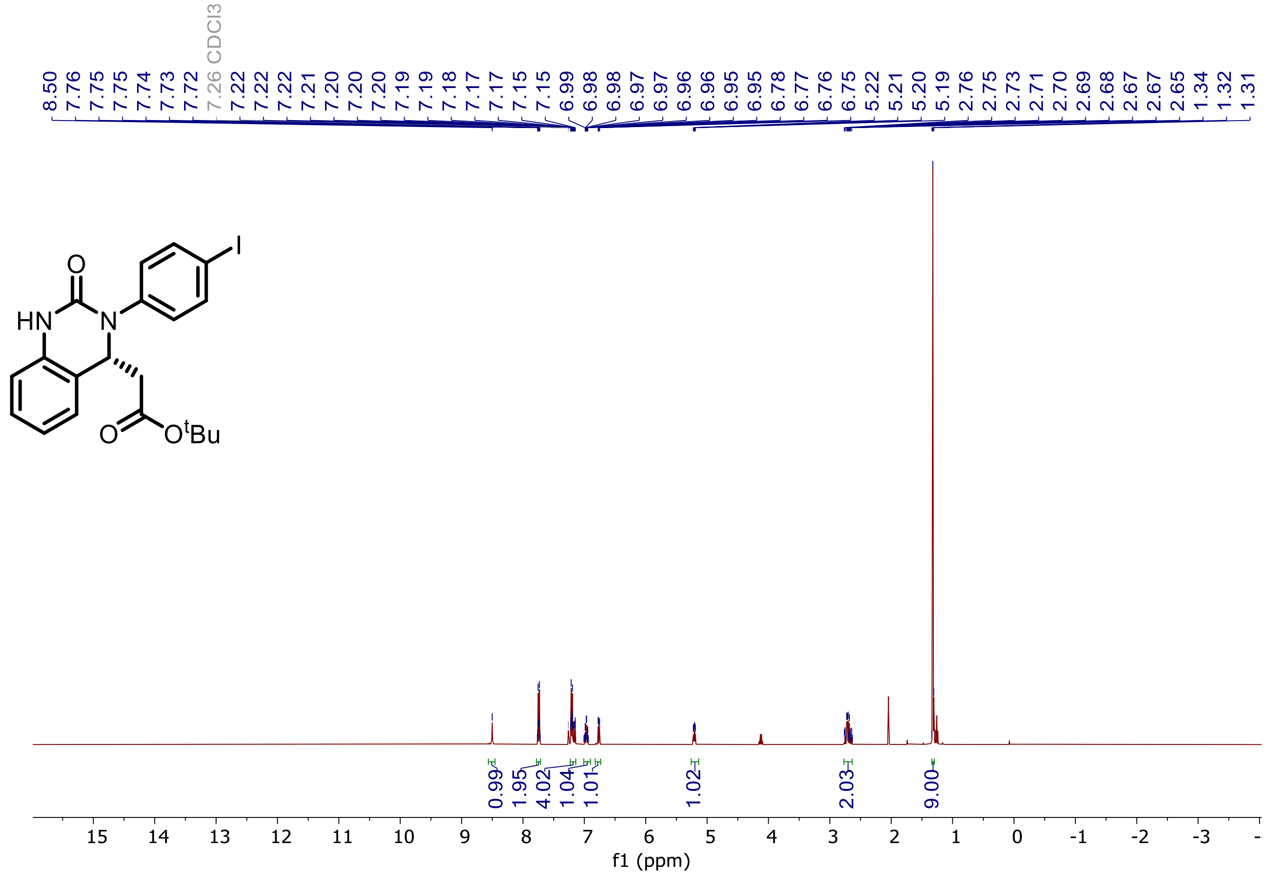

^{13}C NMR: (101 MHz, $CDCl_3$, 298K) of **2j**

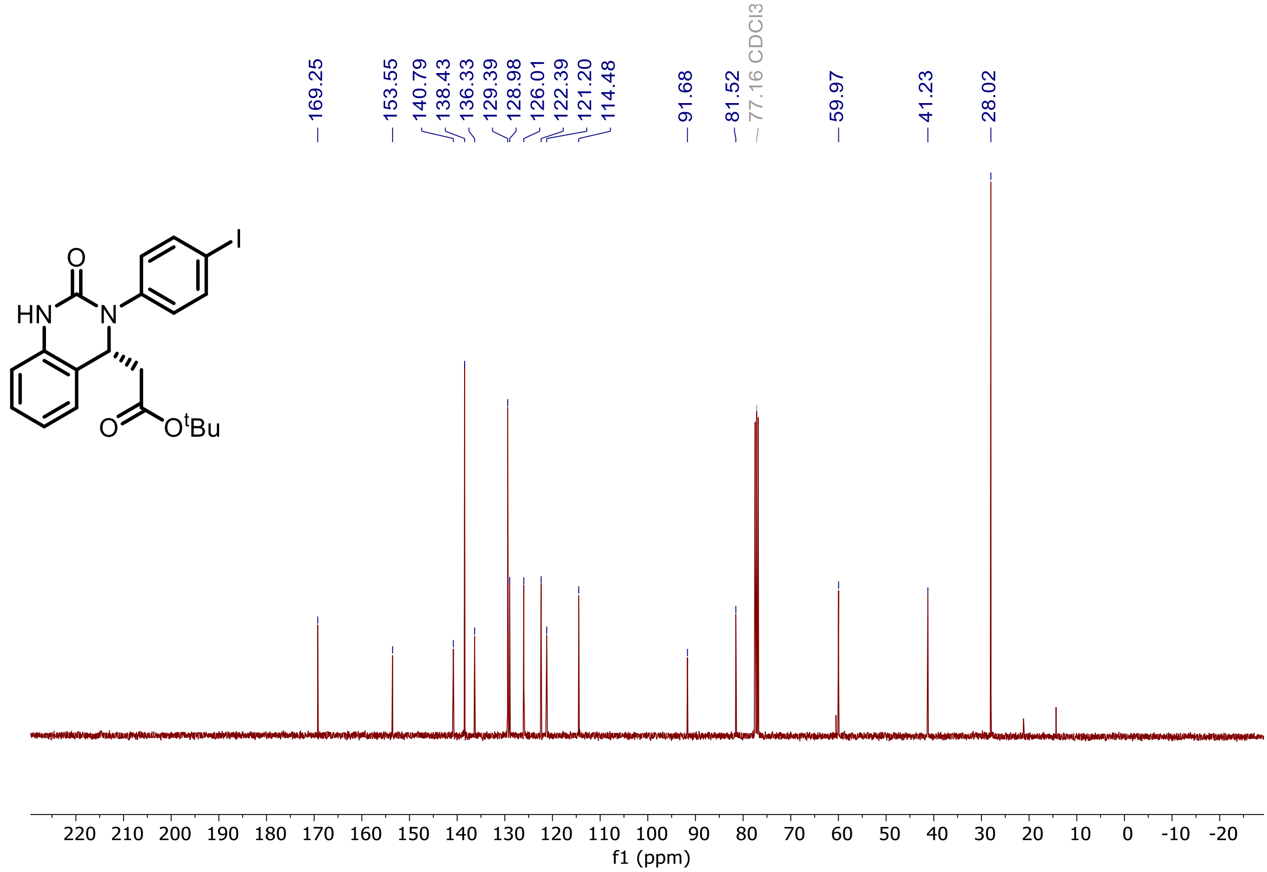

^{1}H NMR: (400 MHz, $CDCl_3$, 298K) of **2k**

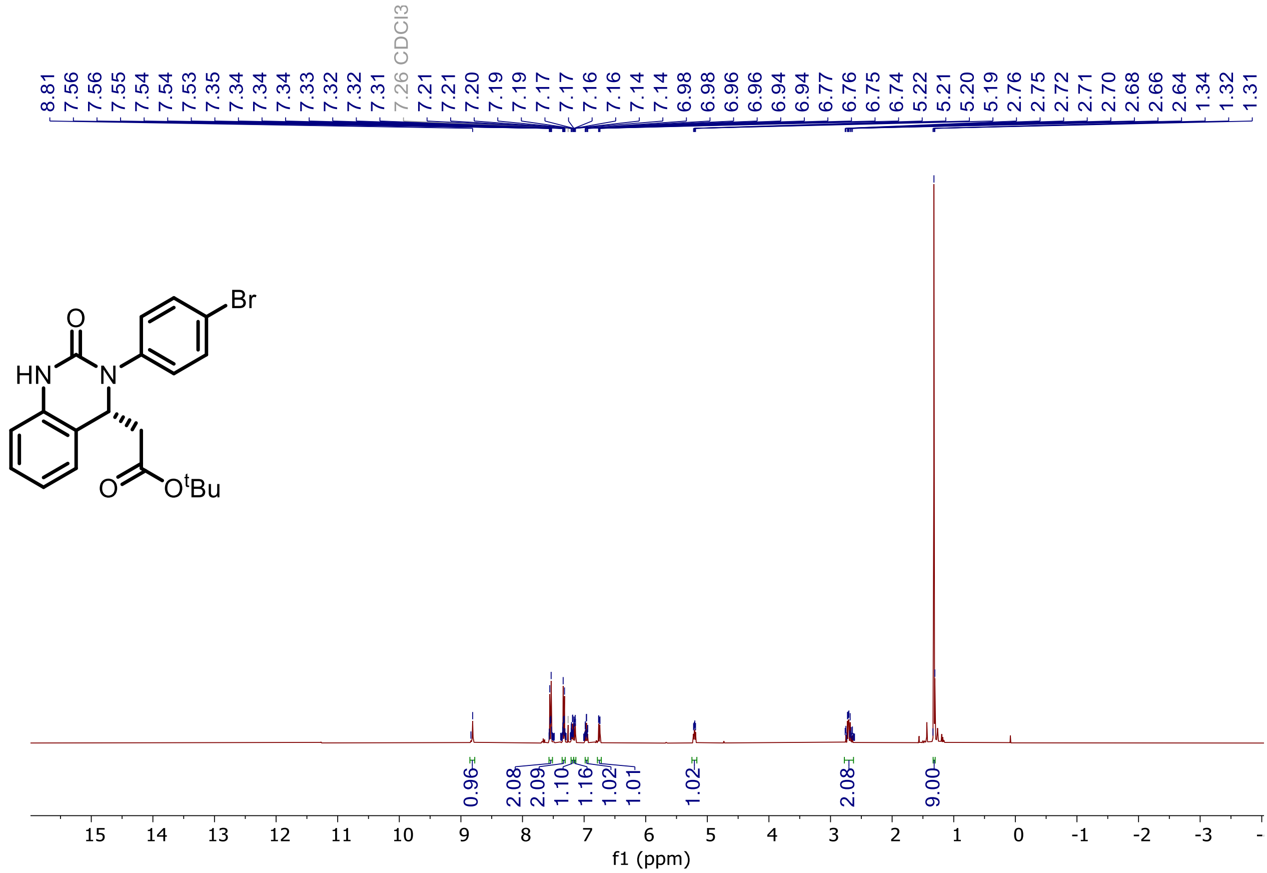

^{13}C NMR: (101 MHz, $CDCl_3$, 298K) of **2k**

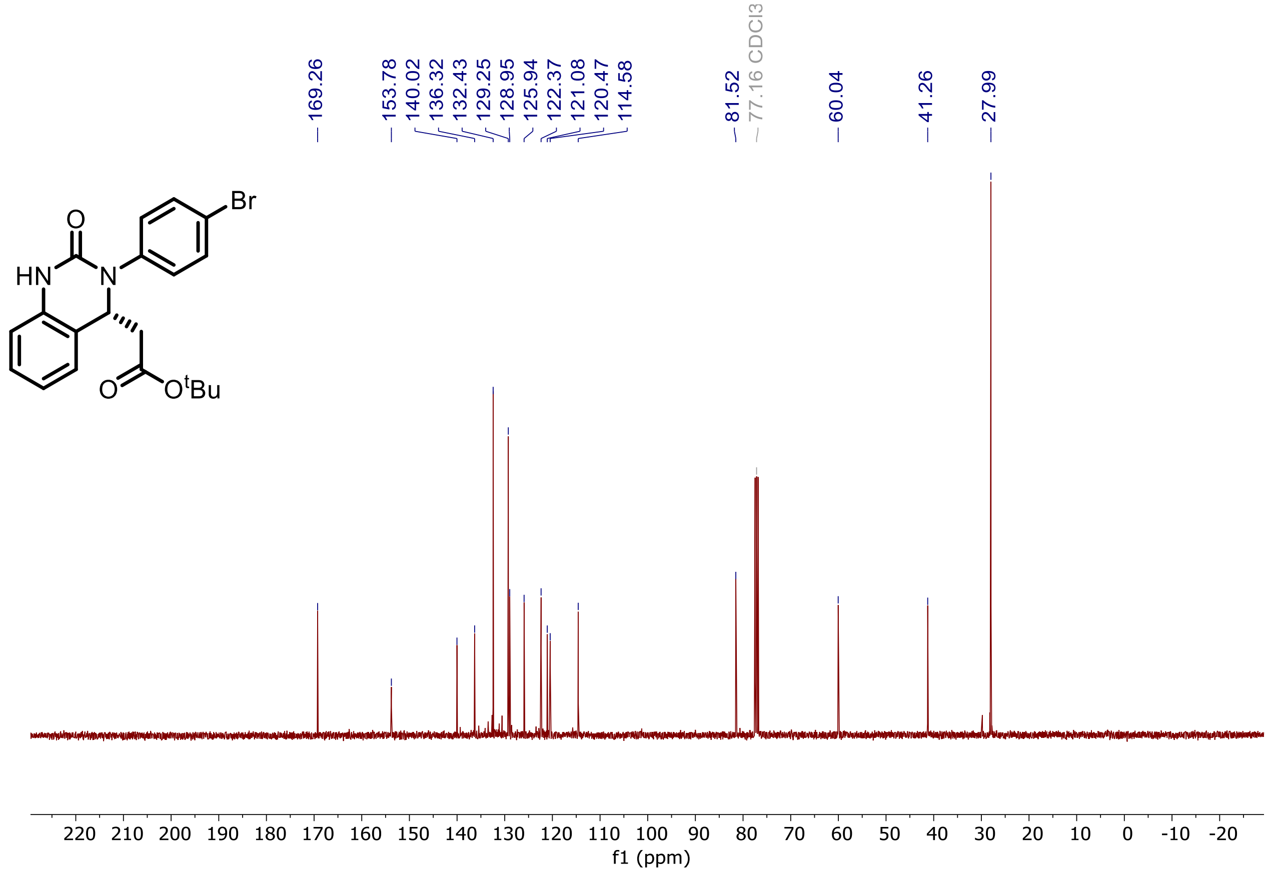

^{1}H NMR: (400 MHz, $CDCl_3$, 298K) of **21**

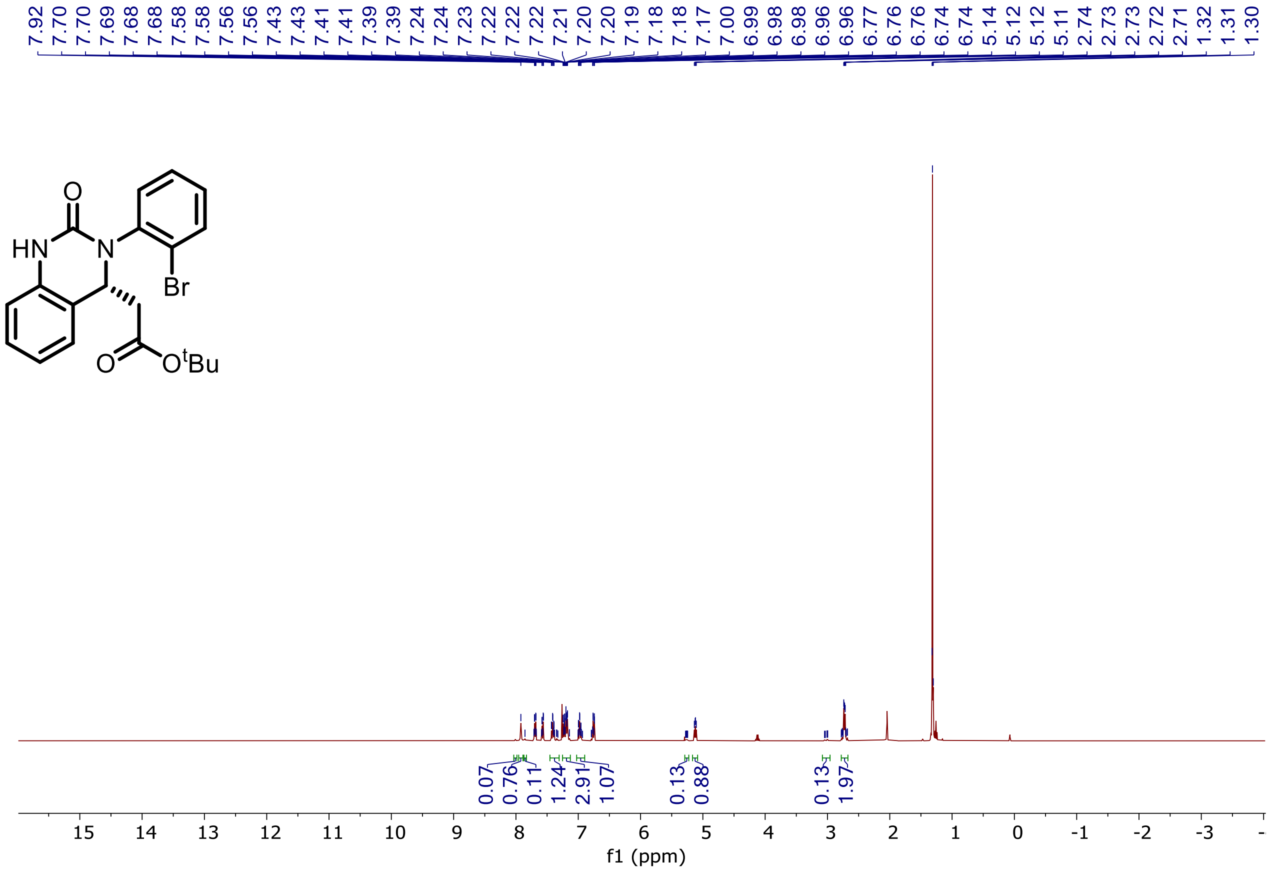

^{13}C NMR: (101 MHz, $CDCl_3$, 298K) of **21**

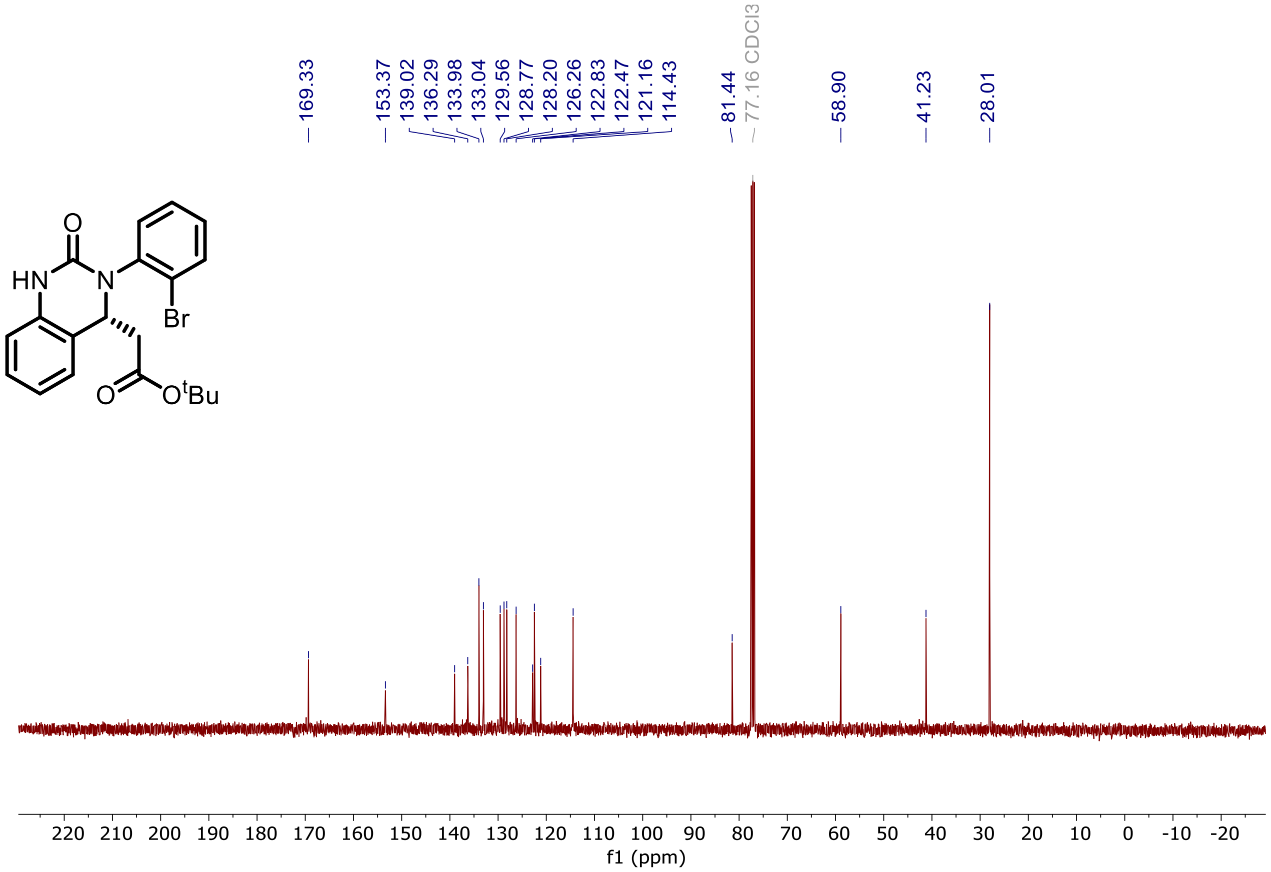

^{1}H NMR: (400 MHz, $CDCl_3$, 298K) of **2m**

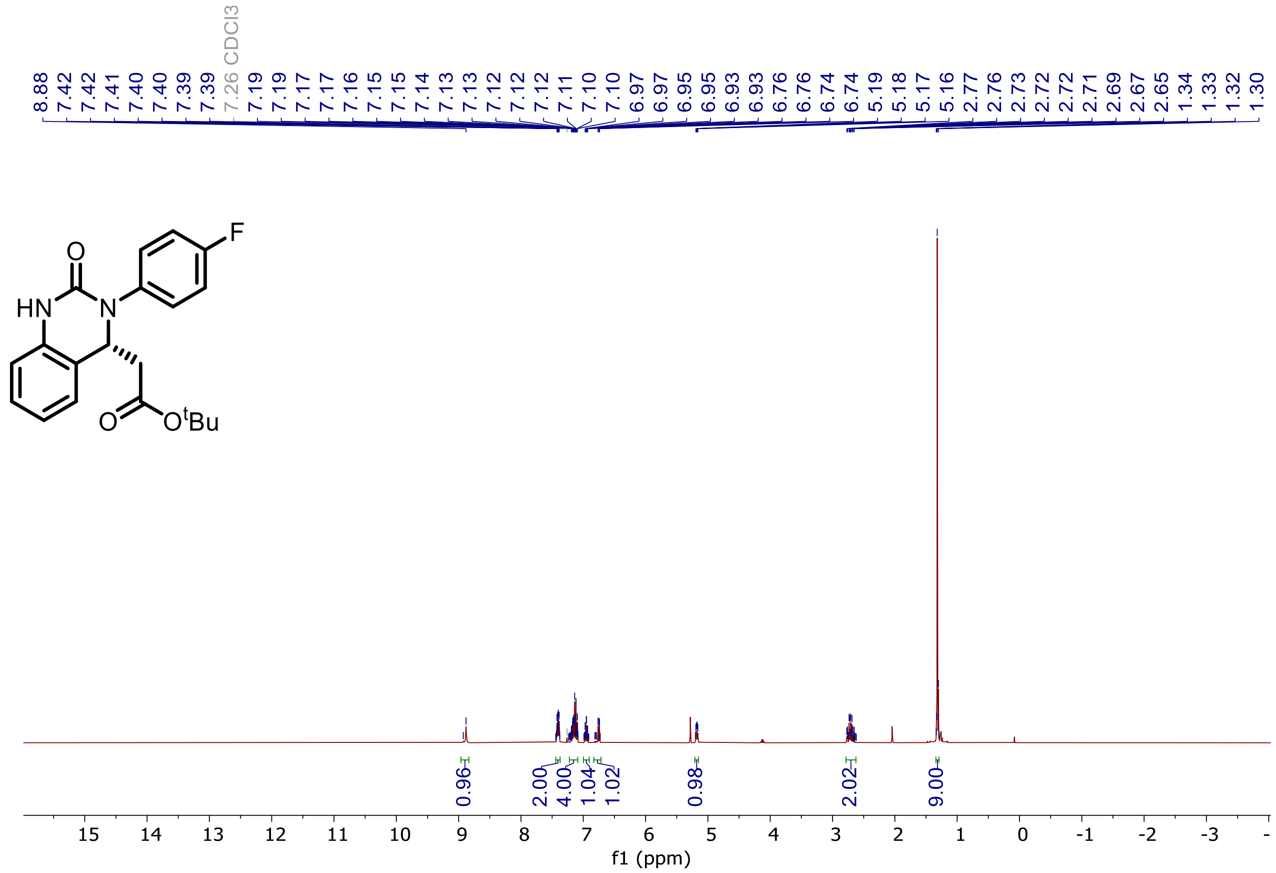

^{13}C NMR: (101 MHz, $CDCl_3$, 298K) of **2m**

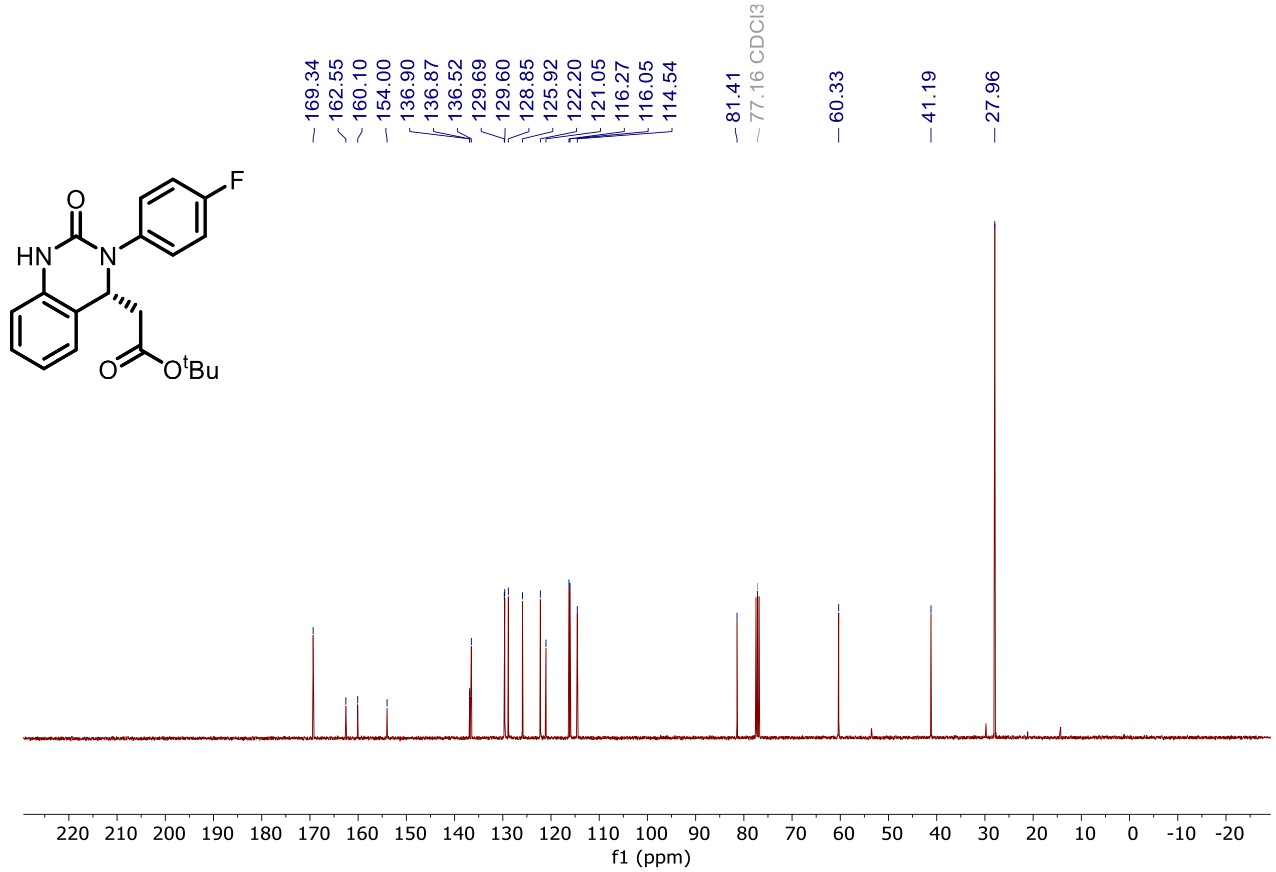

^{19}F NMR: (377 MHz, $CDCl_3$, 298K) of **2m**

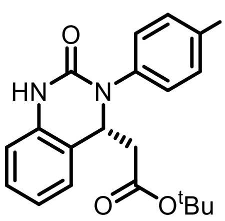

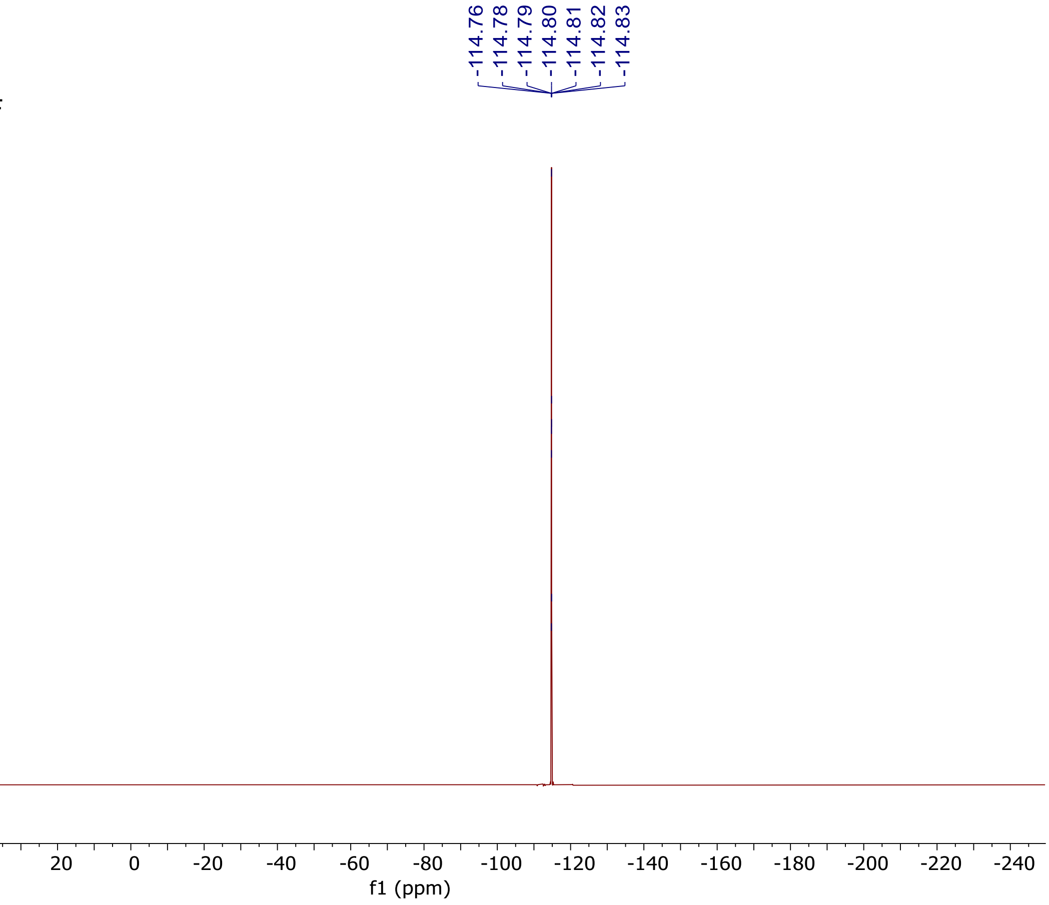

^{1}H NMR: (400 MHz, $CDCl_3$, 298K) of **2n**

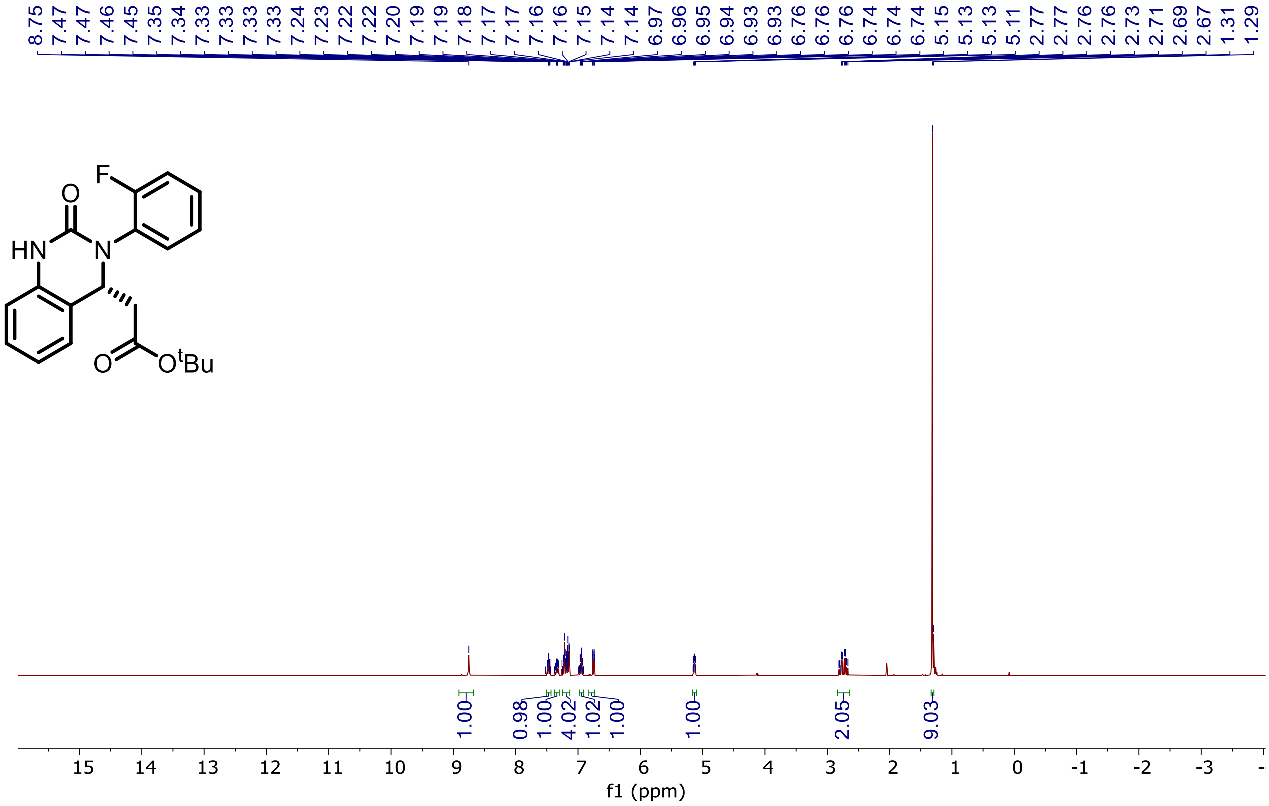

^{13}C NMR: (101 MHz, $CDCl_3$, 298K) of **2n**

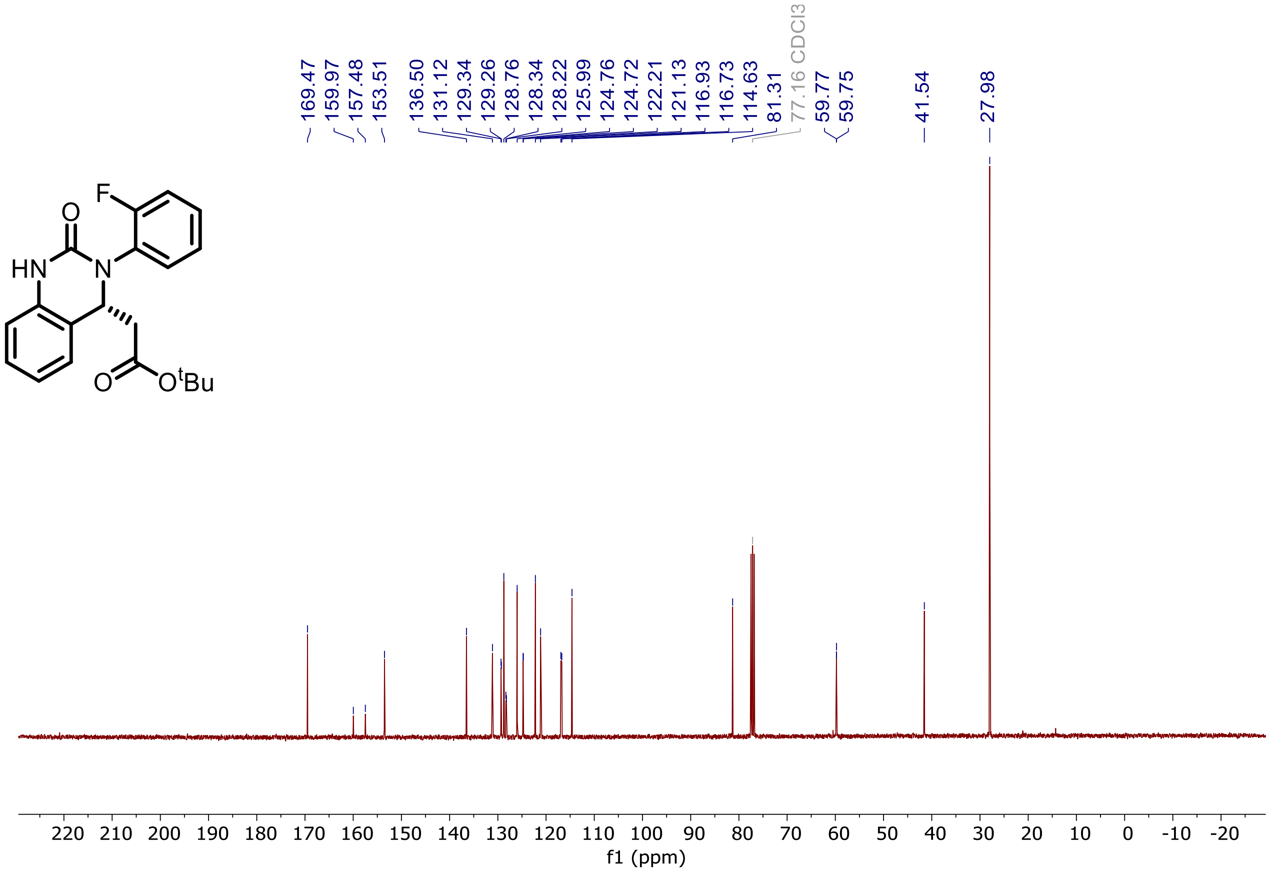

^{19}F NMR: (377 MHz, $CDCl_3$, 298K) of **2n**

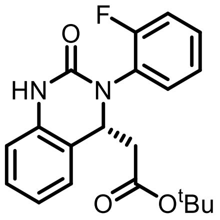

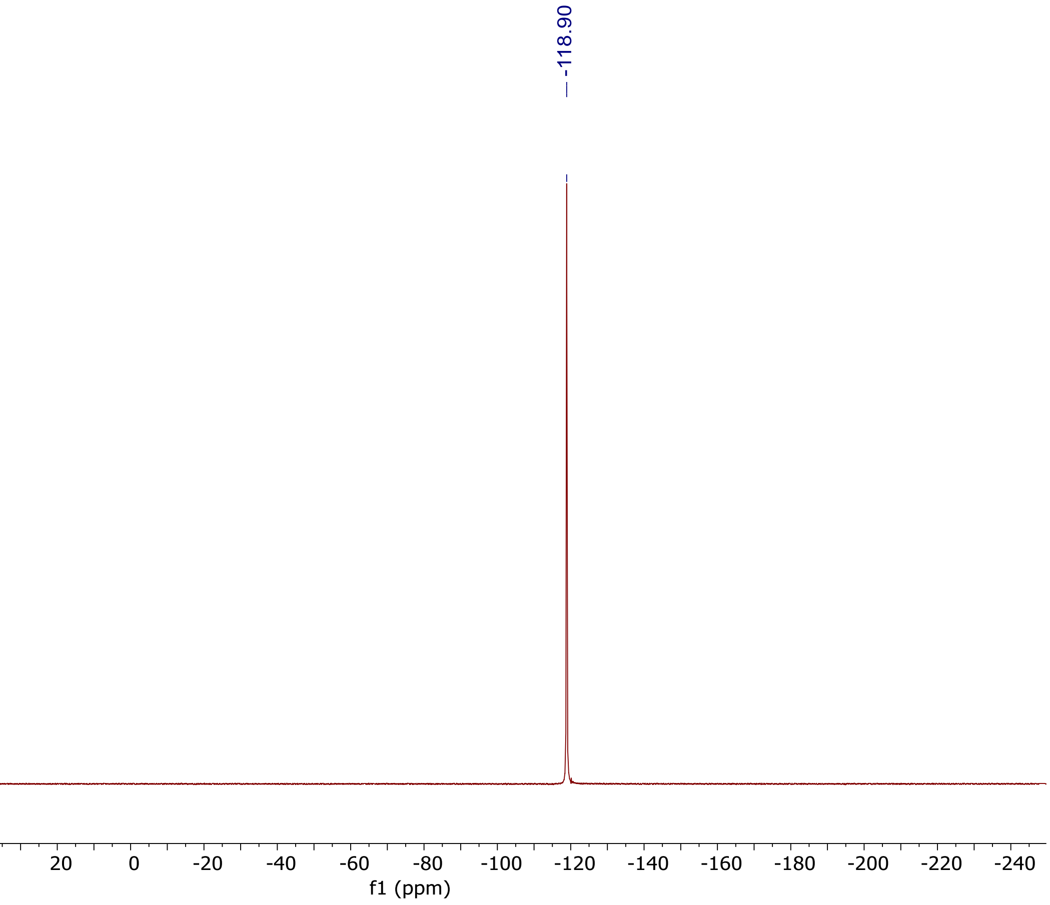

^{1}H NMR: (400 MHz, $CDCl_3$, 298K) of **2o**

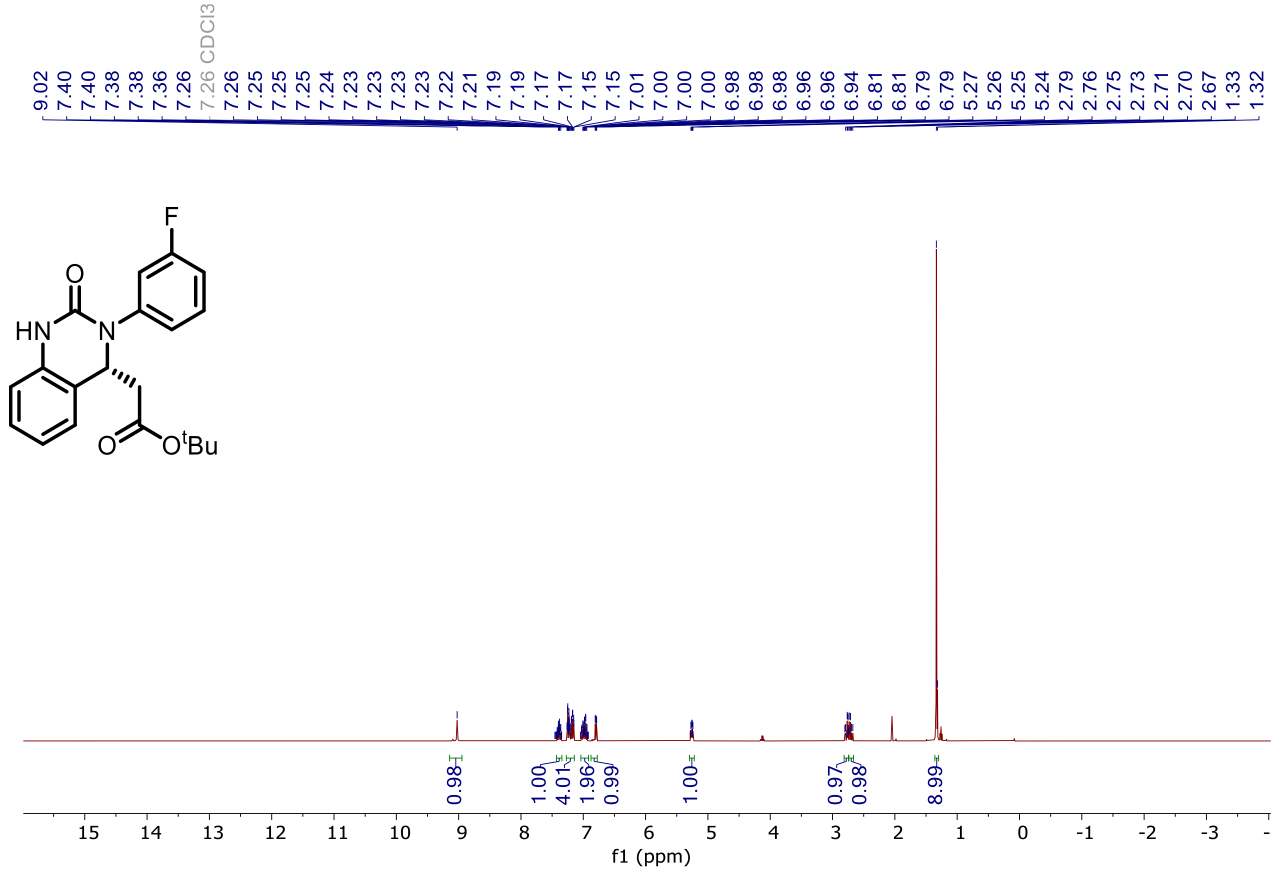

^{13}C NMR: (101 MHz, $CDCl_3$, 298K) of **2o**

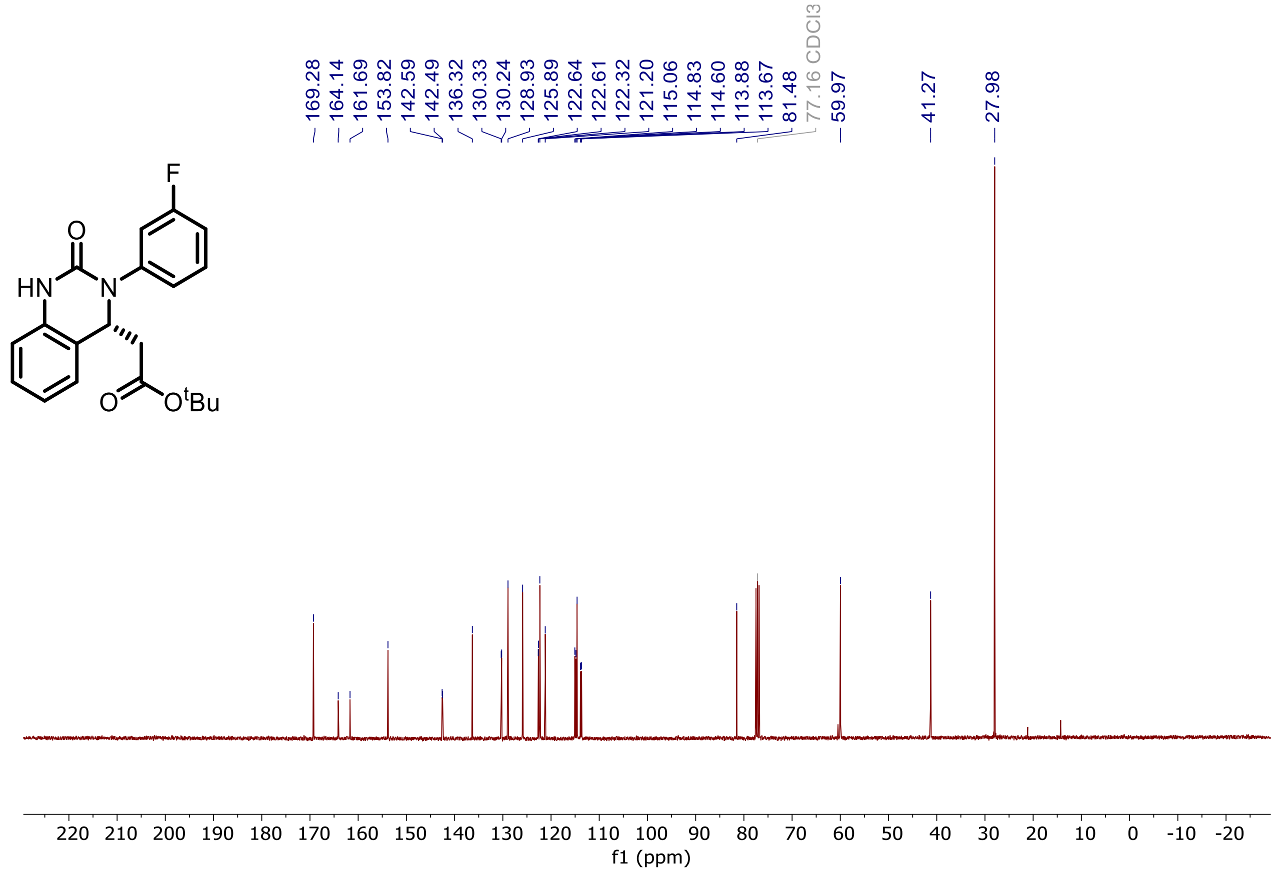

^{19}F NMR: (377 MHz, $CDCl_3$, 298K) of **2o**

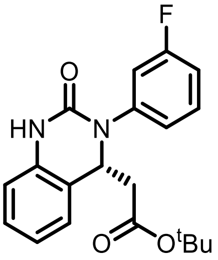

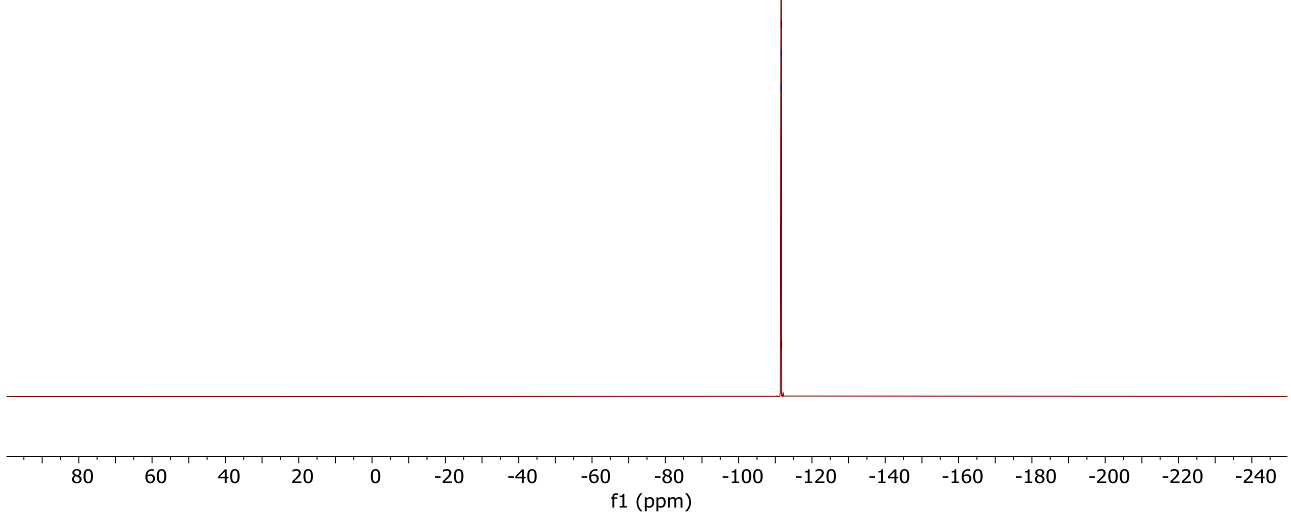

^{1}H NMR: (400 MHz, $CDCl_3$, 298K) of **2p**

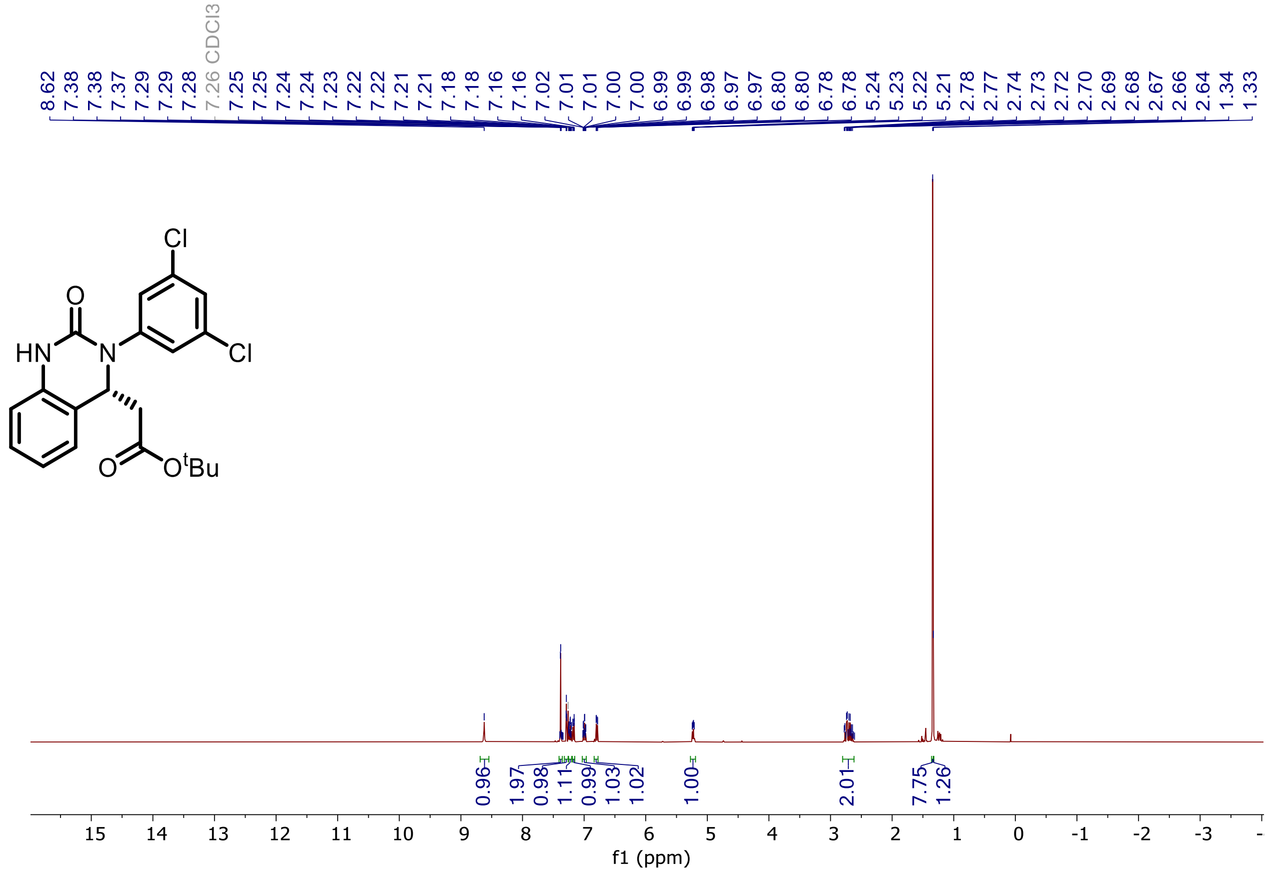

^{13}C NMR: (101 MHz, $CDCl_3$, 298K) of **2p**

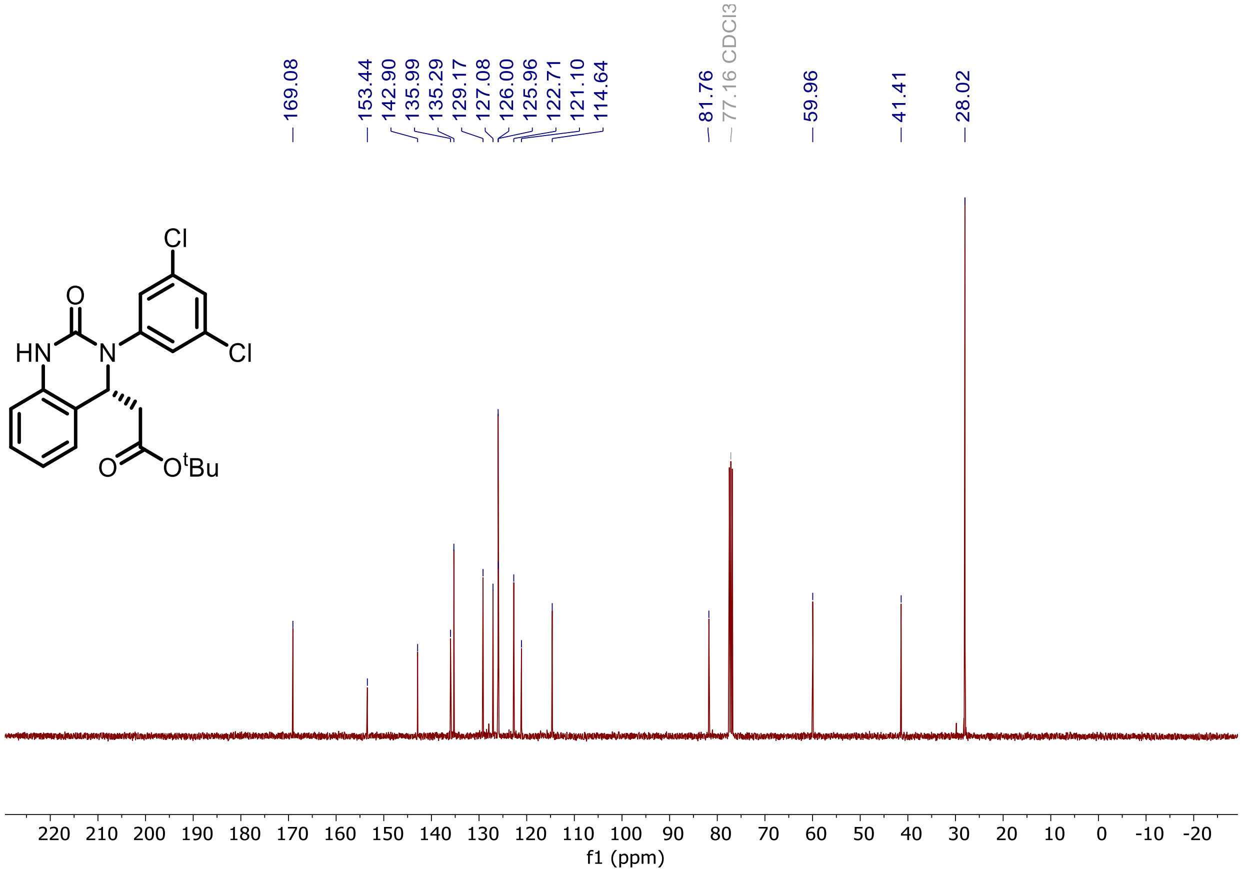

^{1}H NMR: (400 MHz, $CDCl_3$, 298K) of **2q**

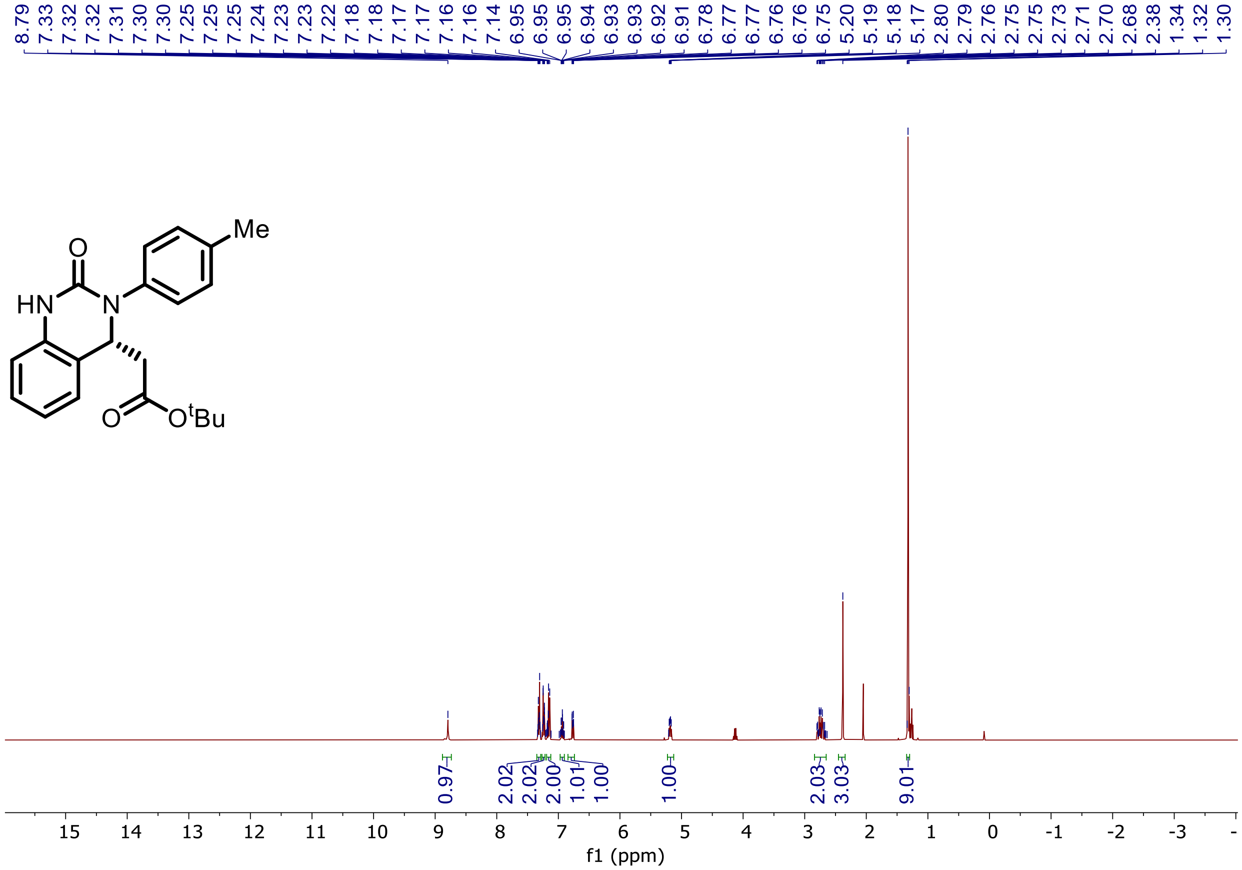

^{13}C NMR: (101 MHz, $CDCl_3$, 298K) of **2q**

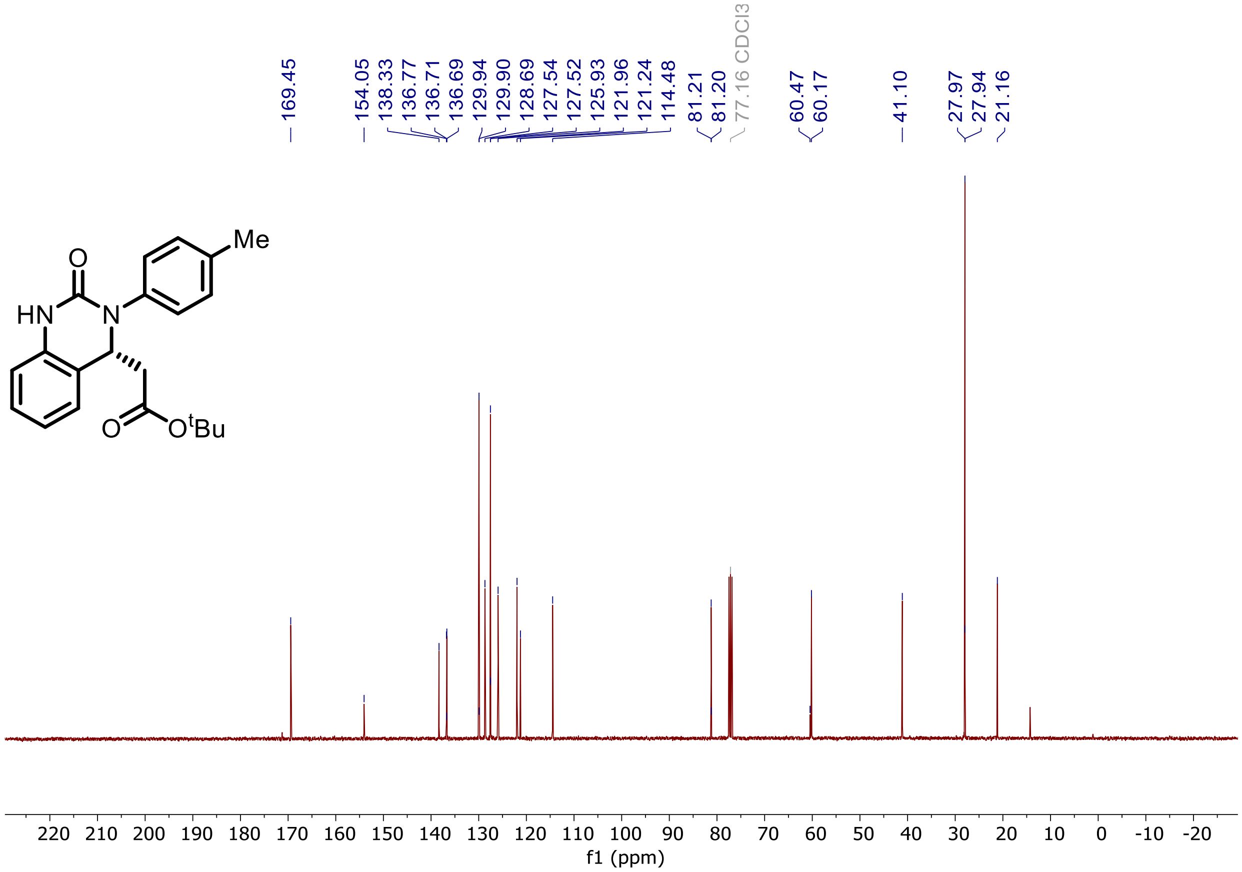

^{1}H NMR: (400 MHz, $CDCl_3$, 298K) of **2r**

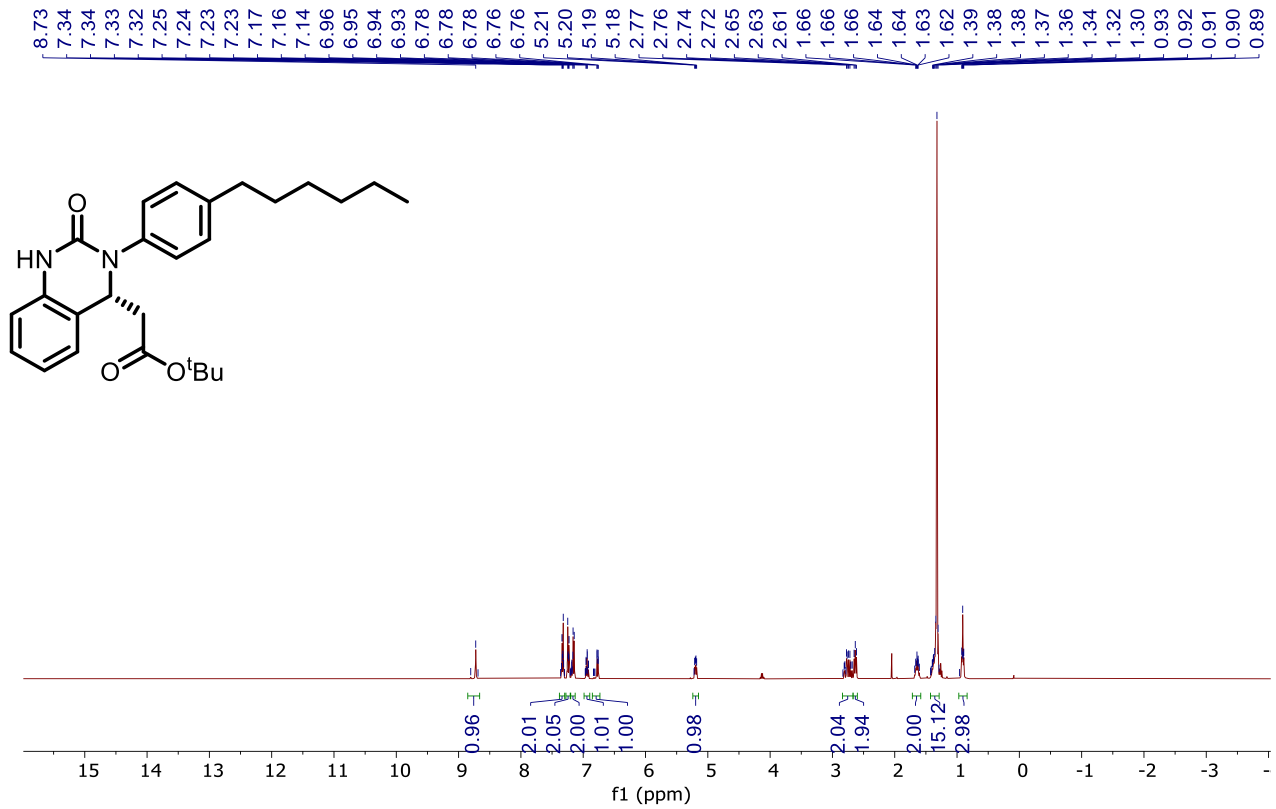

^{13}C NMR: (101 MHz, $CDCl_3$, 298K) of **2r**

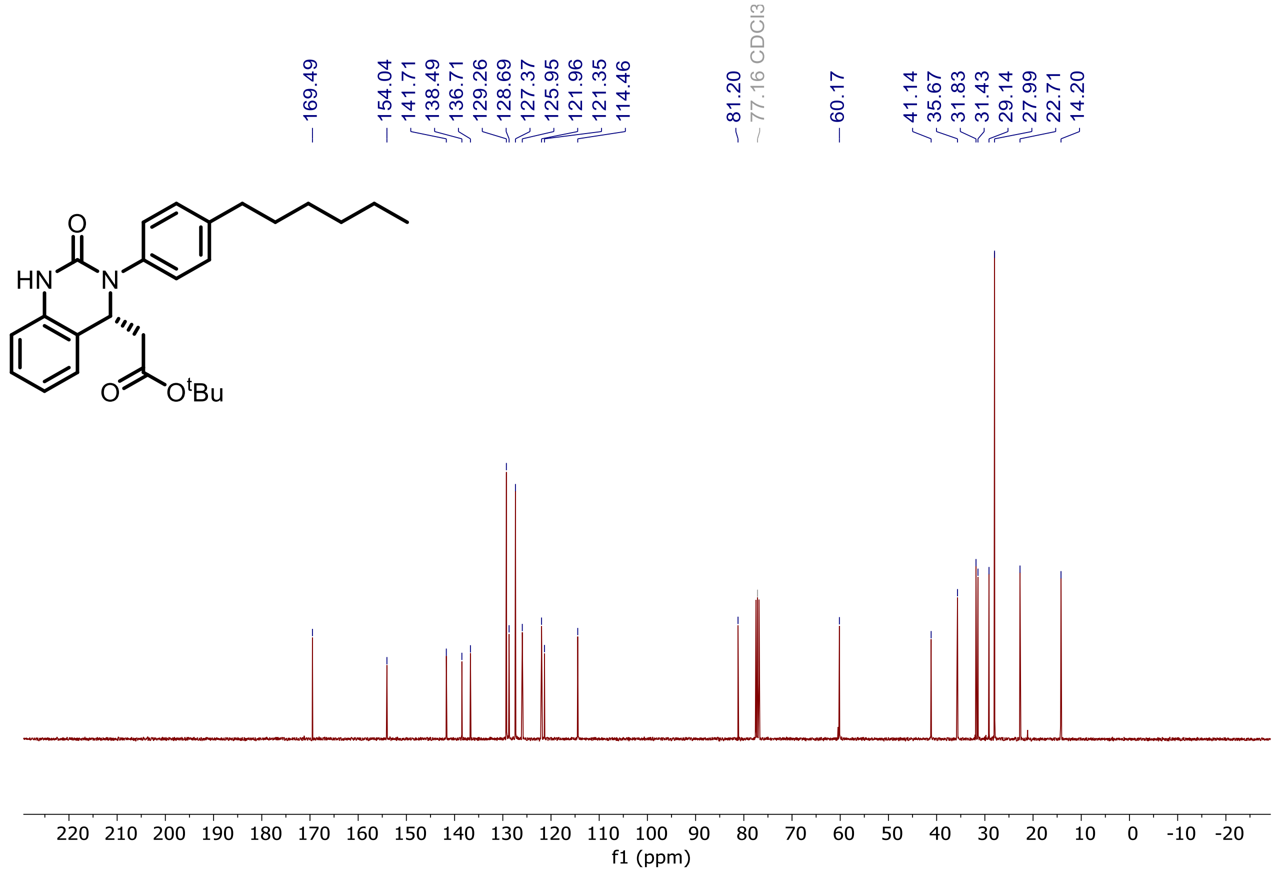

^{1}H NMR: (400 MHz, $CDCl_3$, 298K) of **2s**

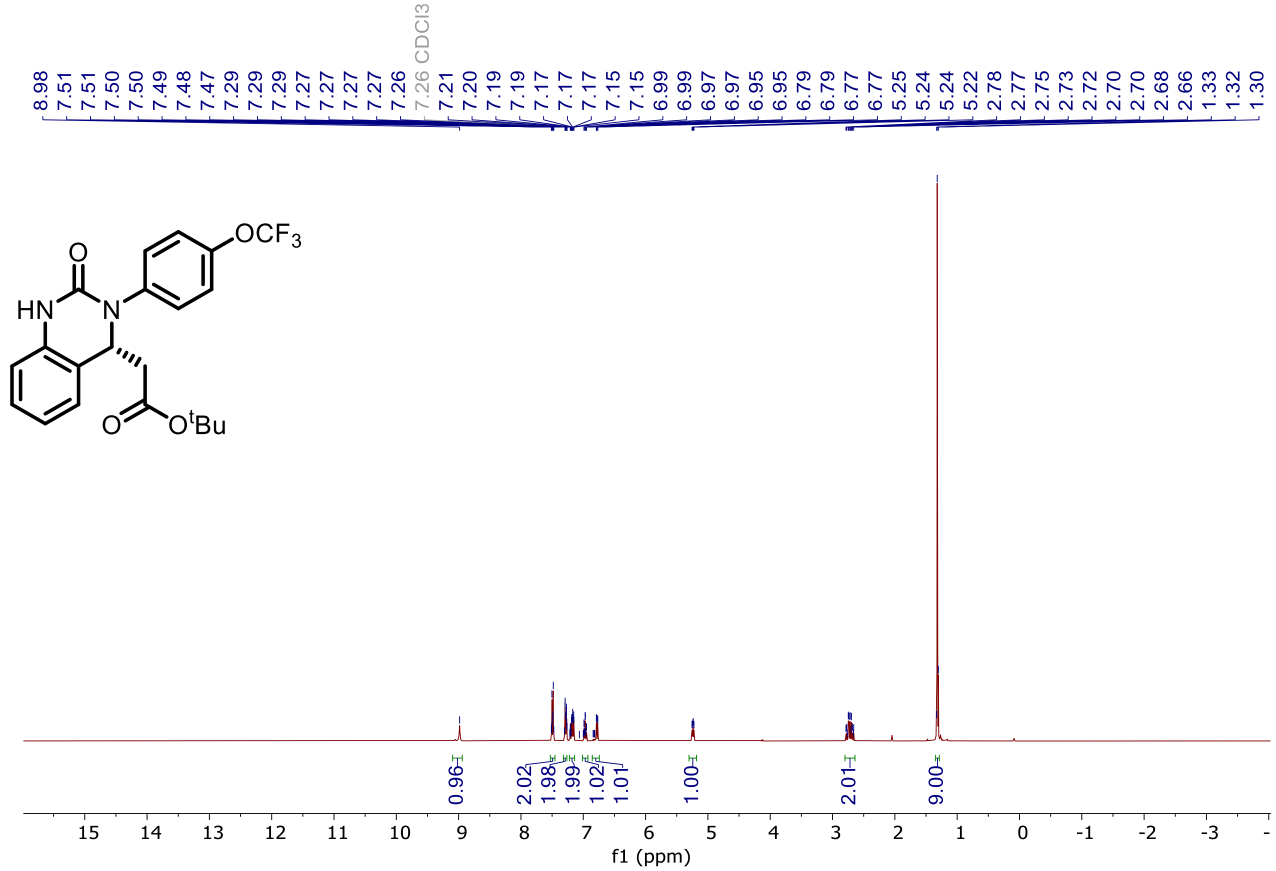

^{13}C NMR: (101 MHz, $CDCl_3$, 298K) of **2s**

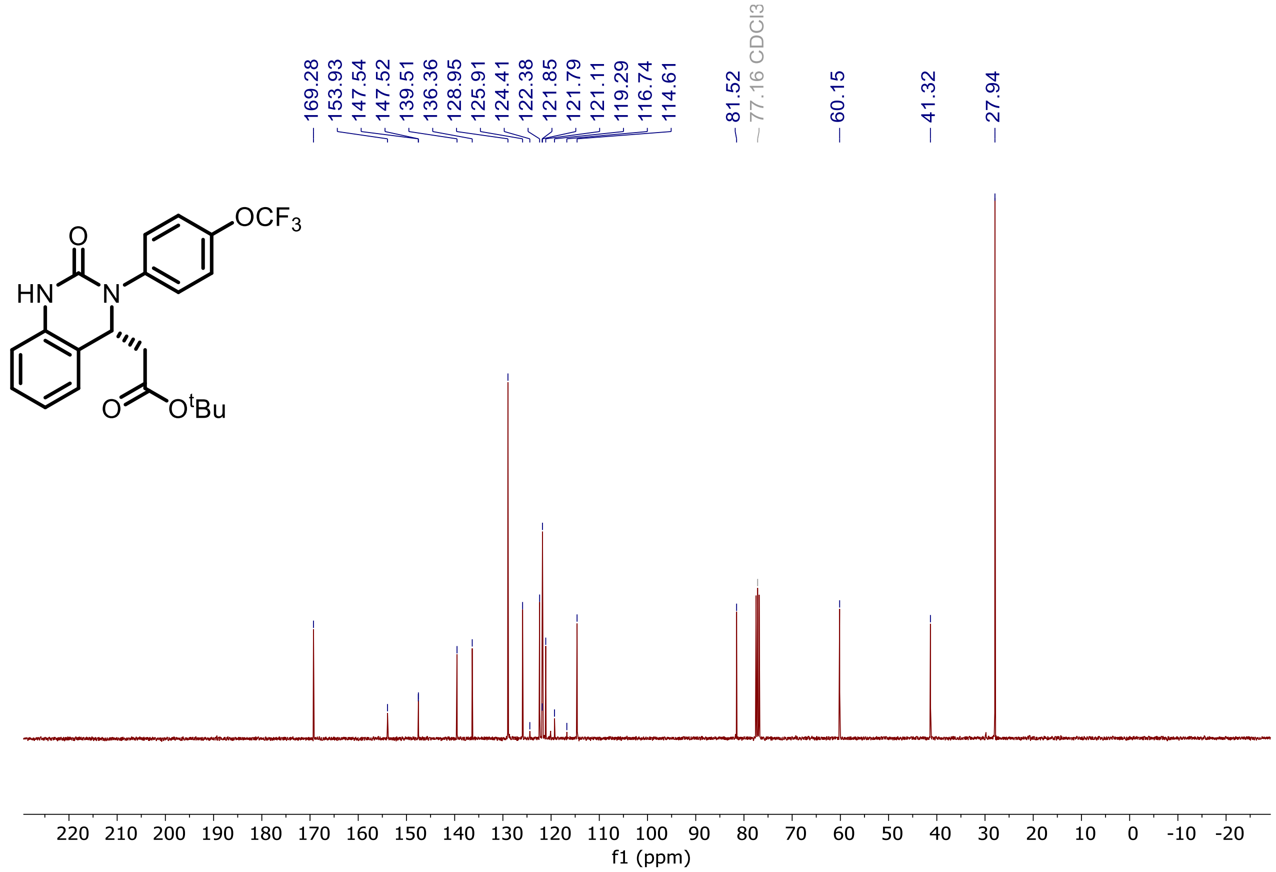

^{19}F NMR: (377 MHz, $CDCl_3$, 298K) of **2s**

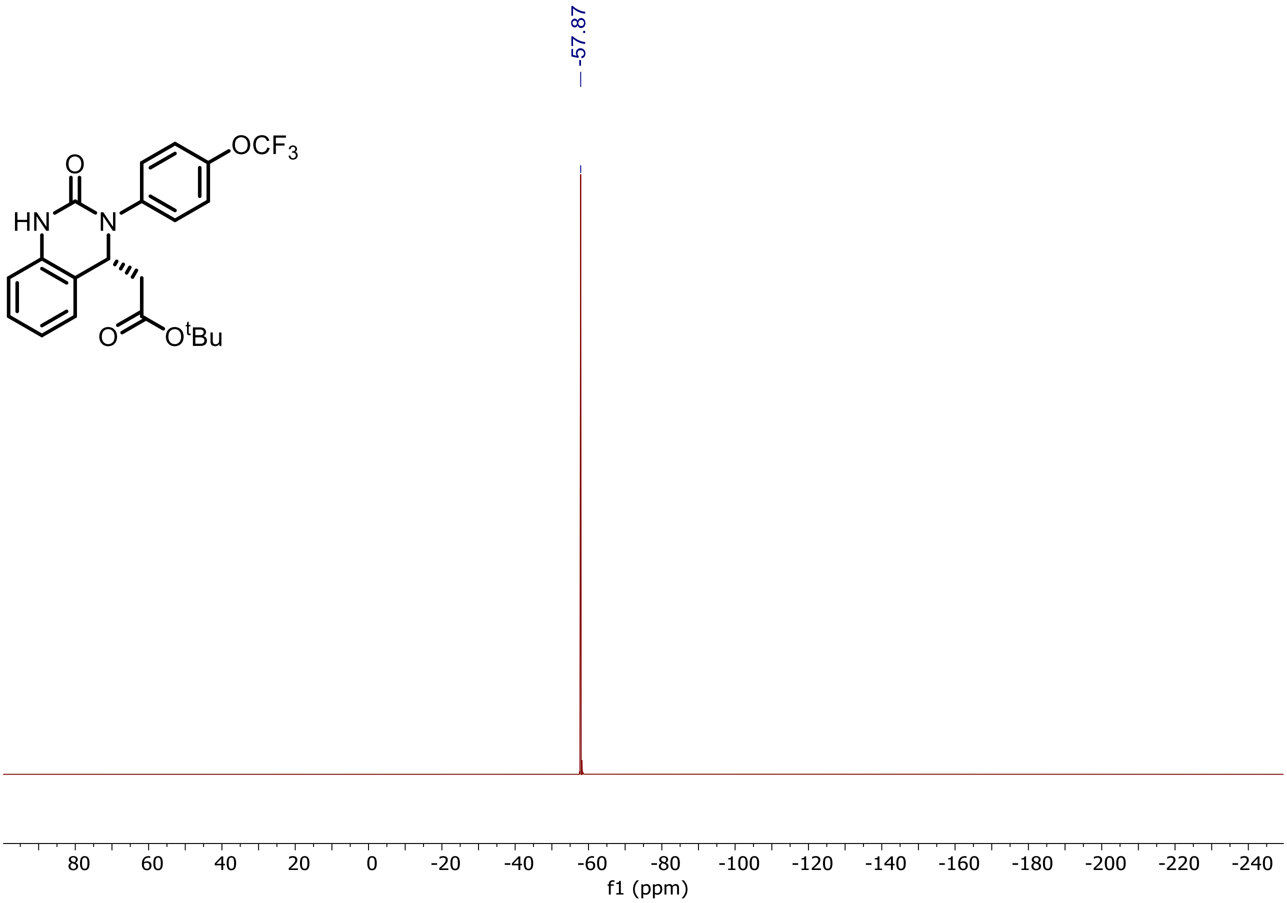

^{1}H NMR: (400 MHz, $CDCl_3$, 298K) of **2t**

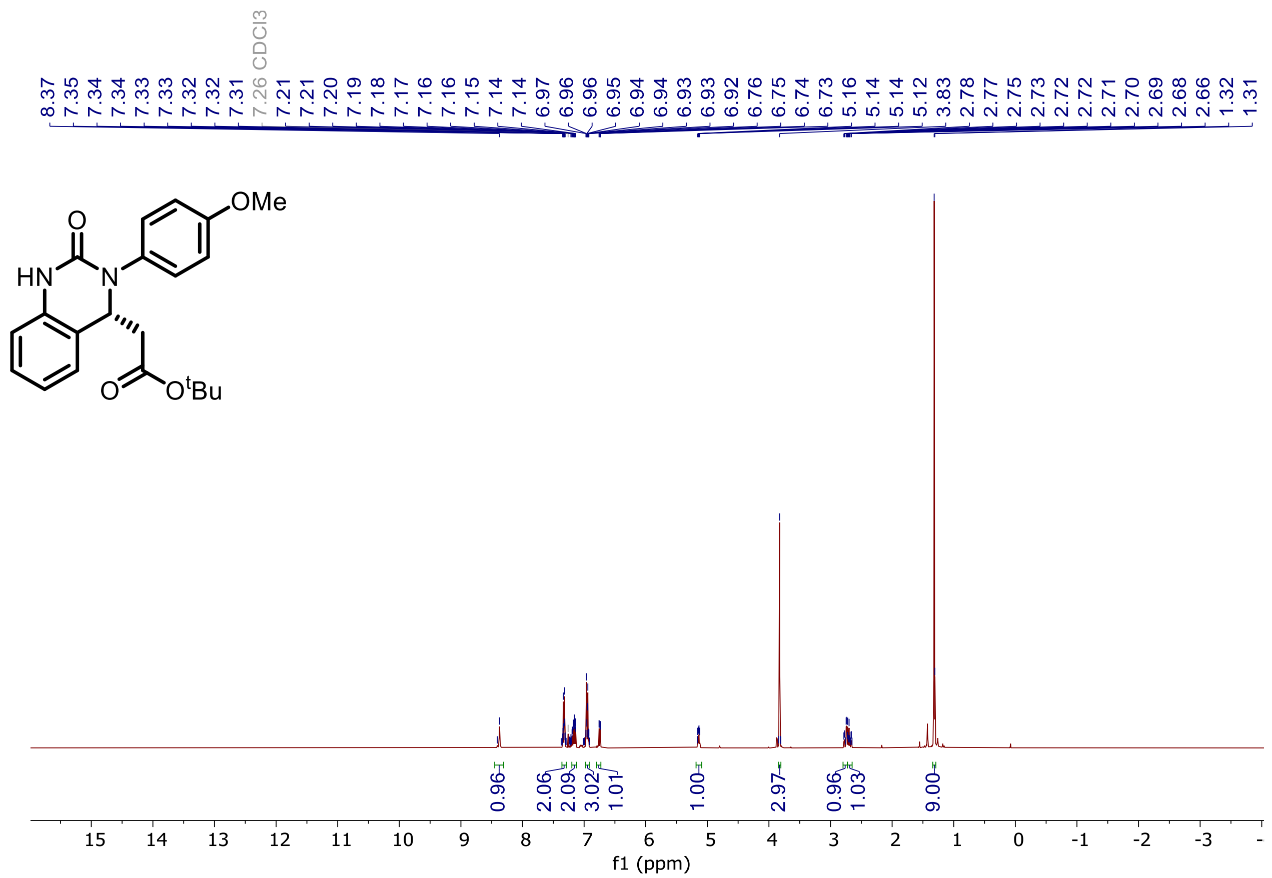

^{13}C NMR: (101 MHz, $CDCl_3$, 298K) of **2t**

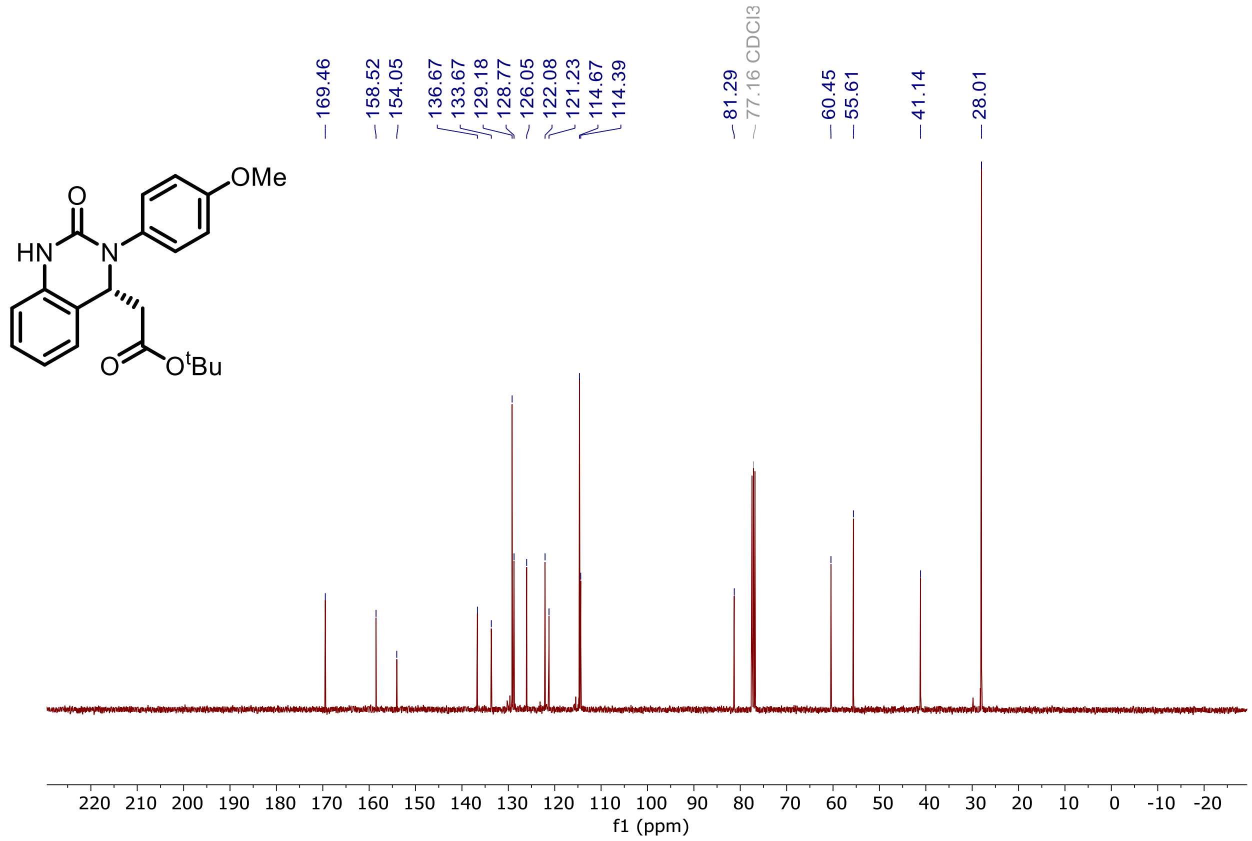

^{1}H NMR: (400 MHz, $CDCl_3$, 298K) of **2u**

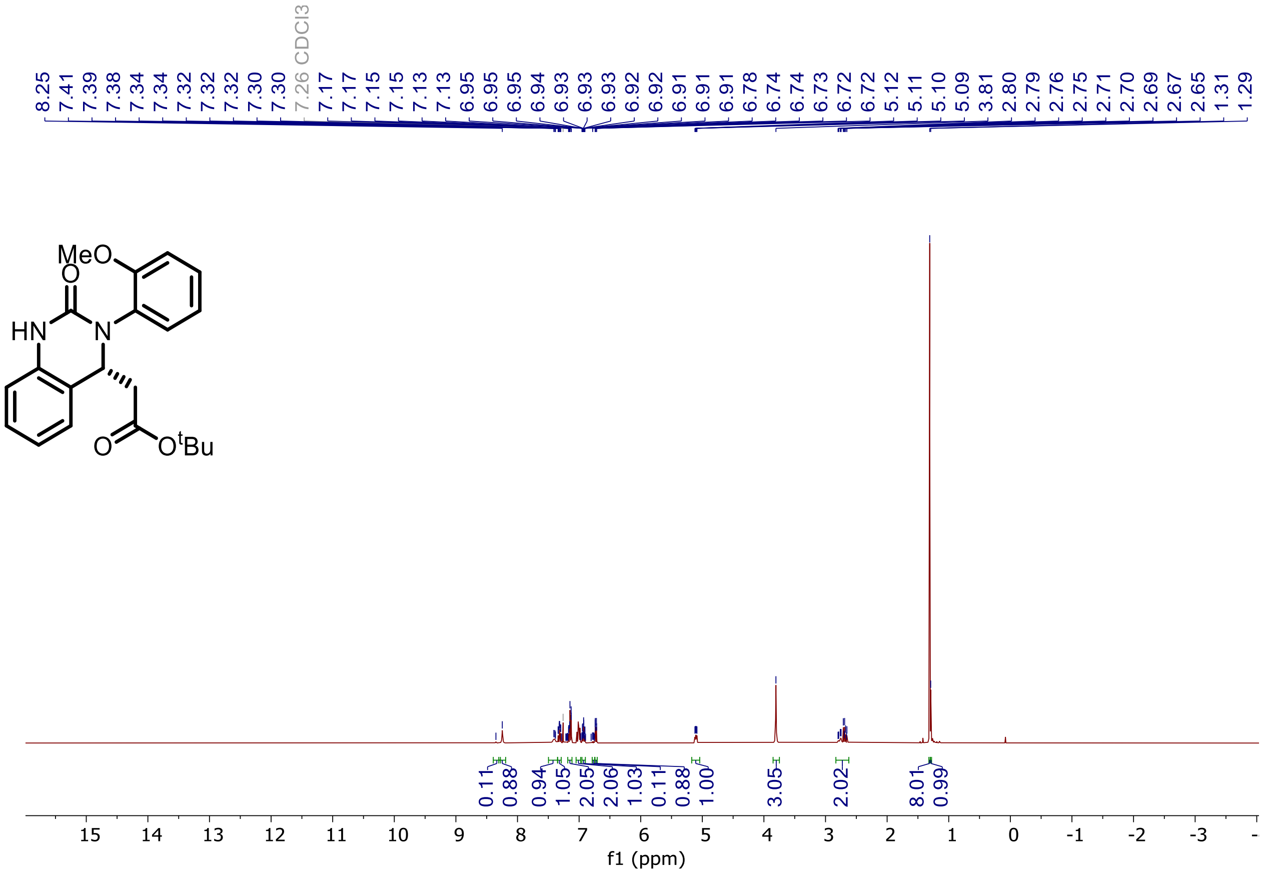

^{13}C NMR: (101 MHz, $CDCl_3$, 298K) of **2u**

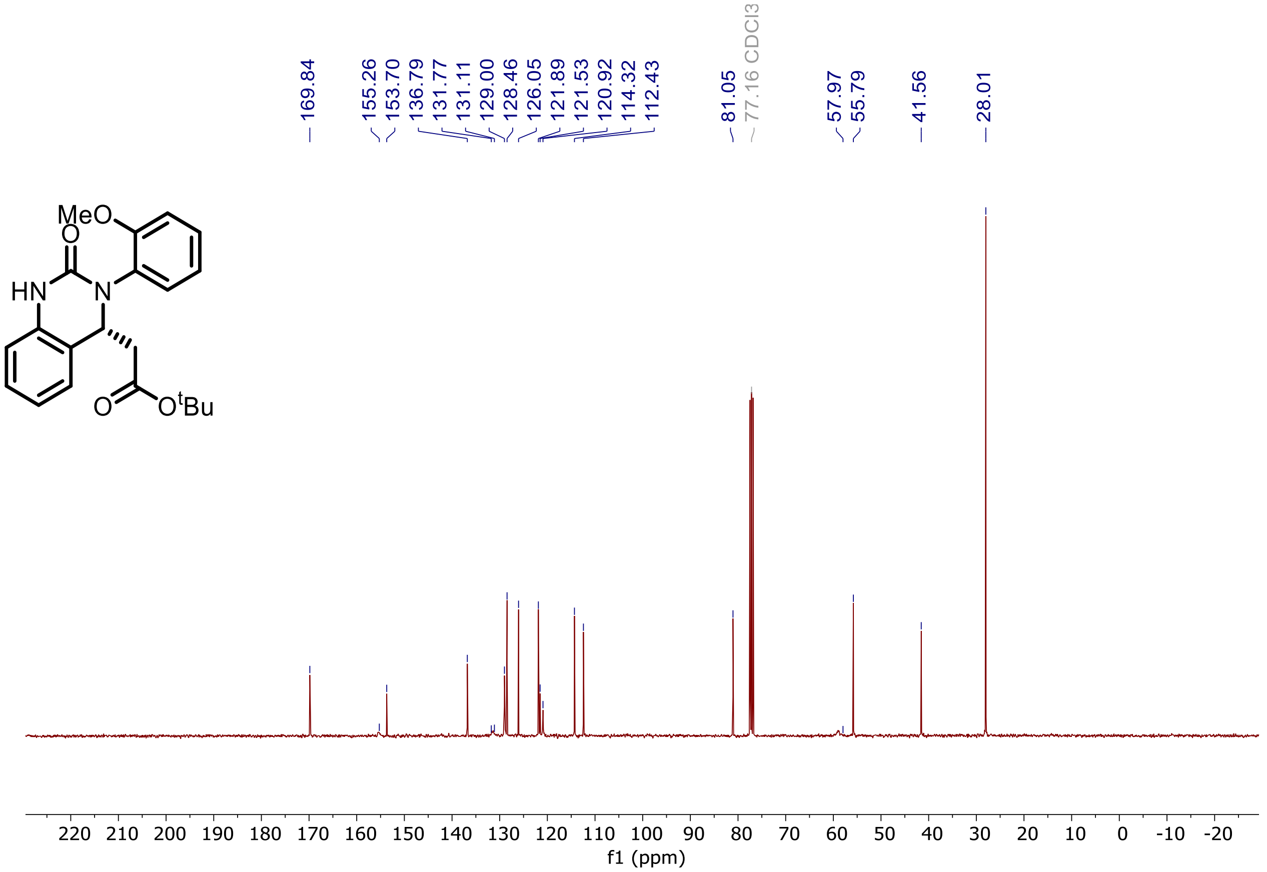

^{1}H NMR: (400 MHz, $CDCl_3$, 298K) of **2v**

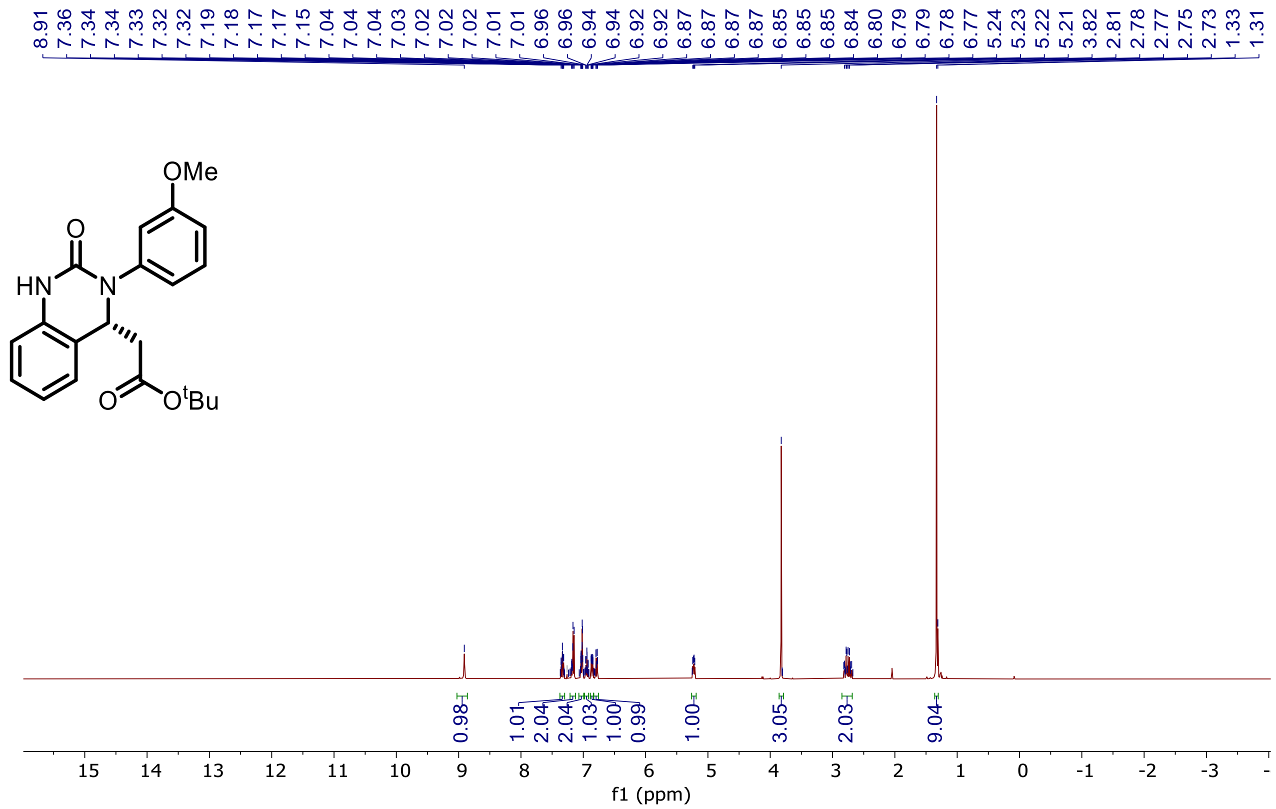

^{13}C NMR: (101 MHz, $CDCl_3$, 298K) of **2v**

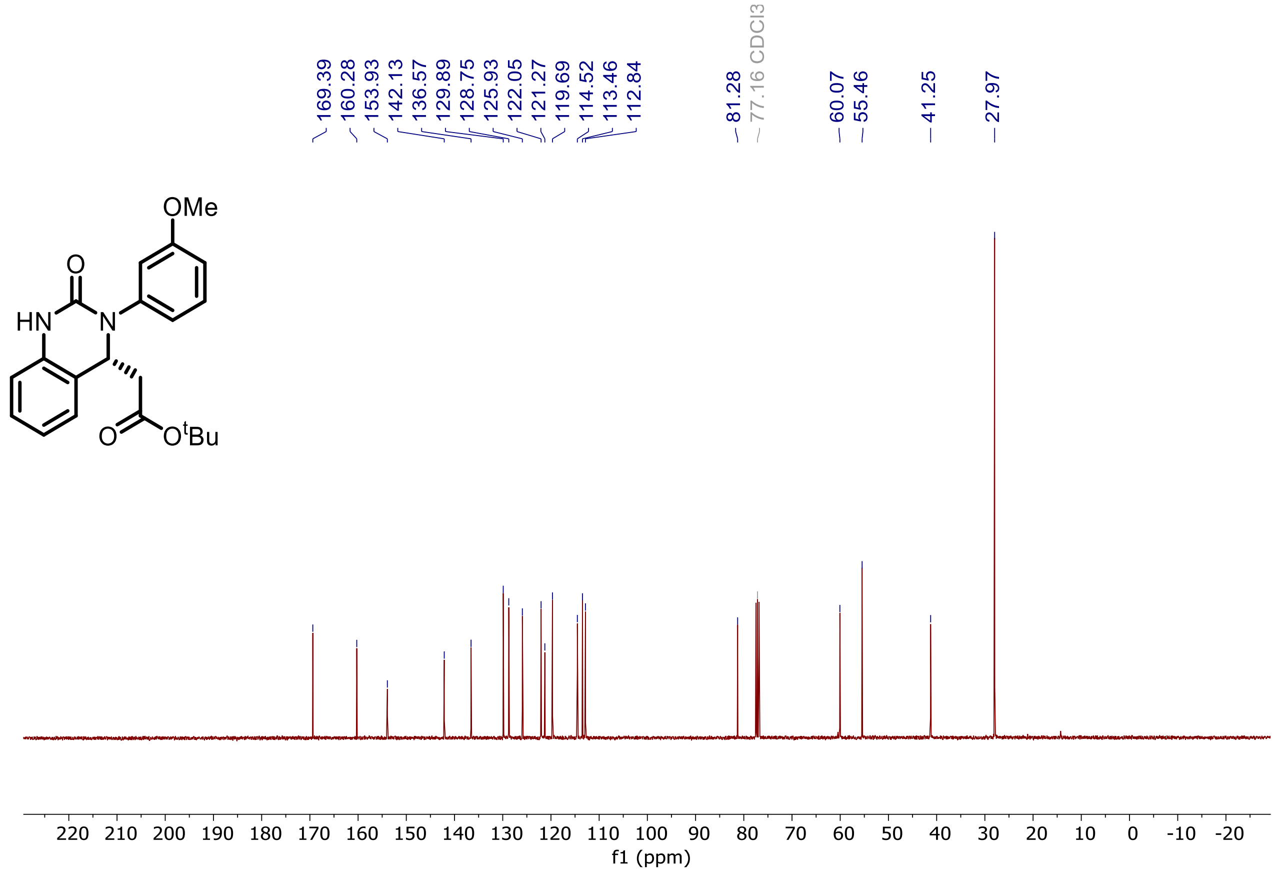

^{1}H NMR: (400 MHz, $CDCl_3$, 298K) of **2w**

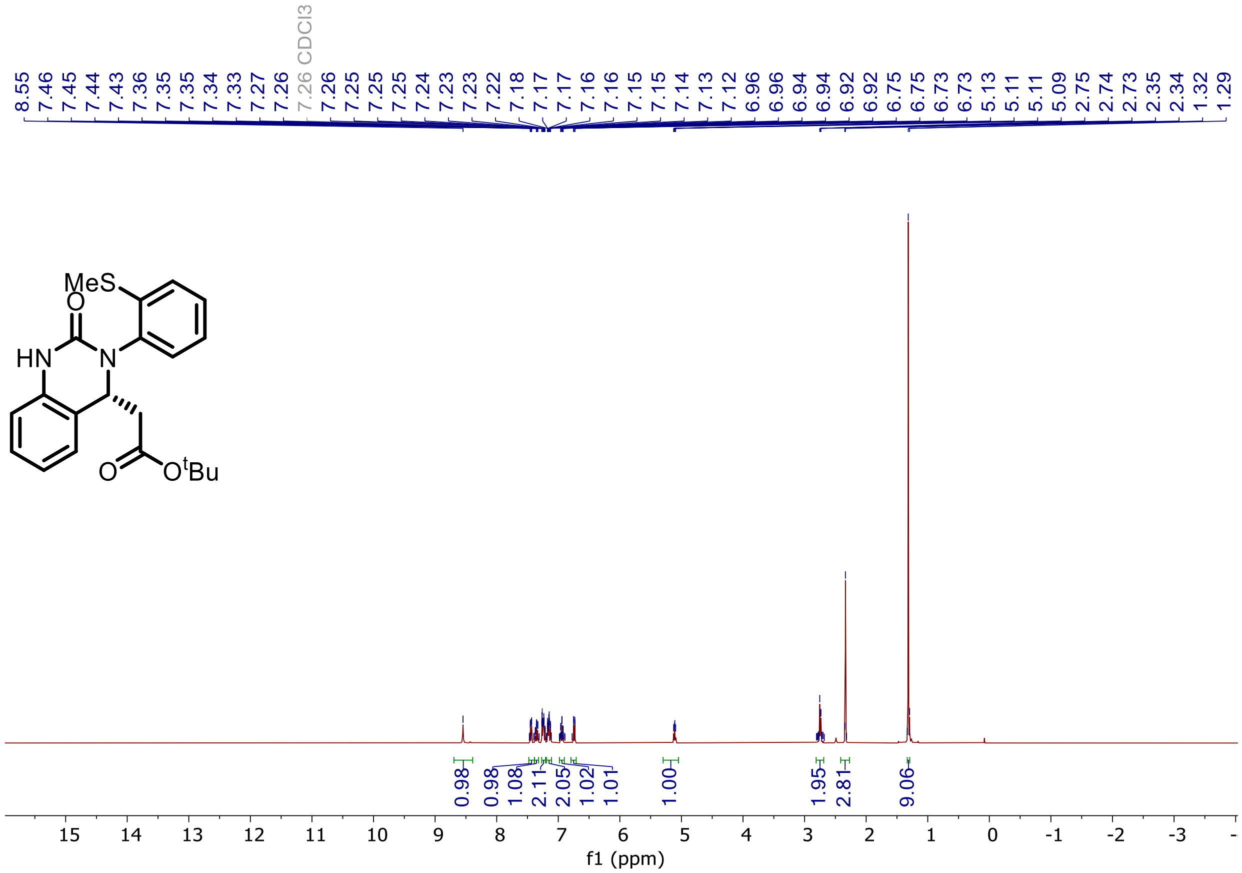

^{13}C NMR: (101 MHz, $CDCl_3$, 298K) of **2w**

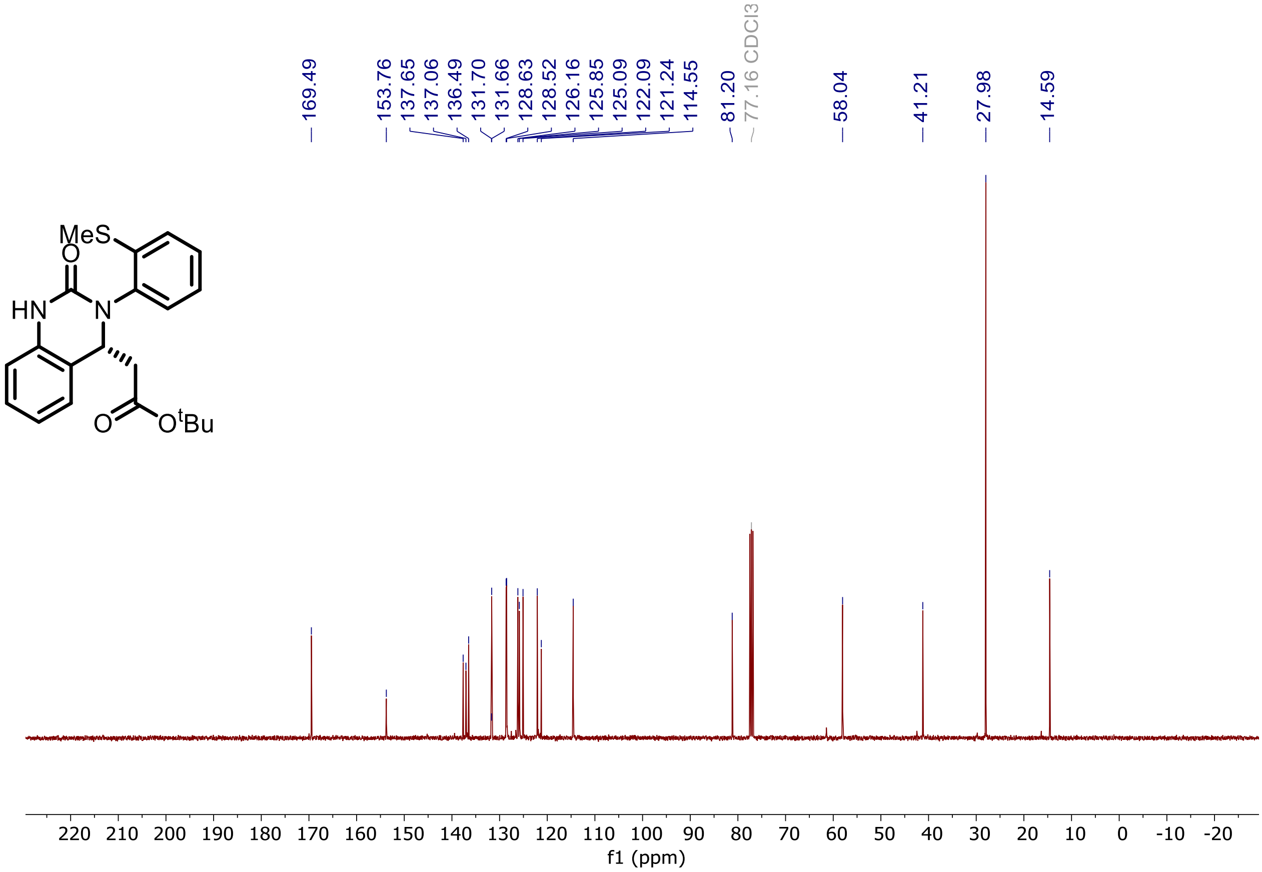

^{1}H NMR: (400 MHz, $CDCl_3$, 298K) of **2x**

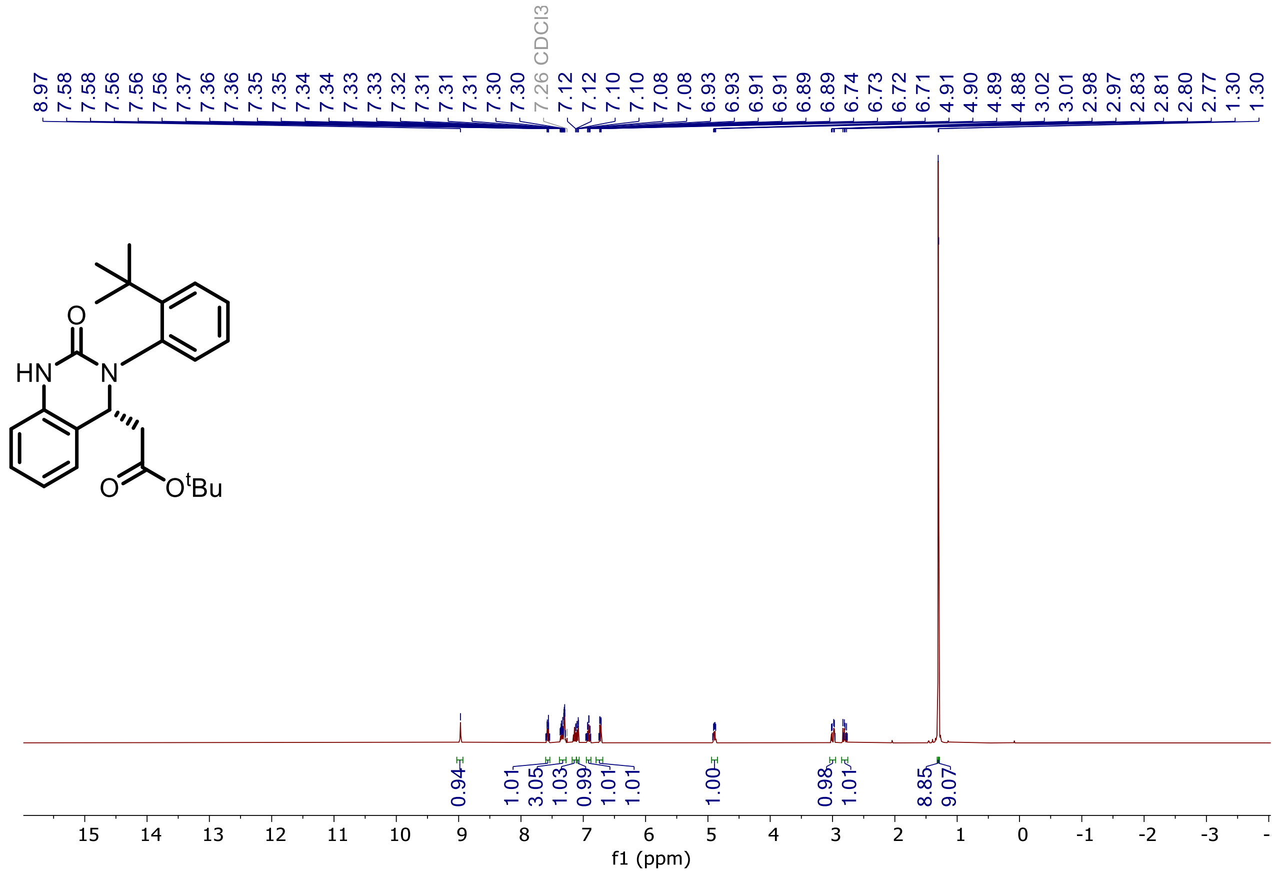

^{13}C NMR: (101 MHz, $CDCl_3$, 298K) of **2x**

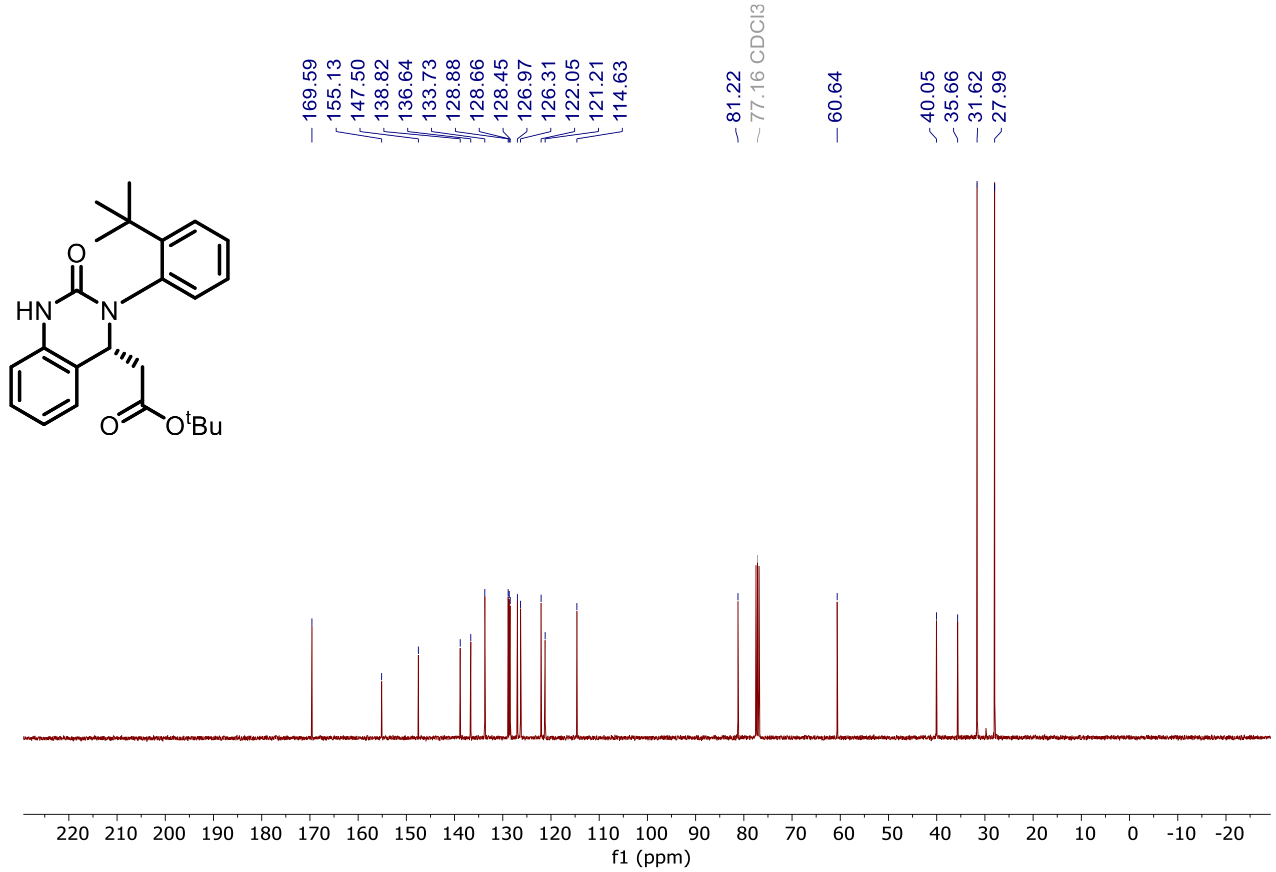

^{1}H NMR: (400 MHz, $CDCl_3$, 298K) of **2y**

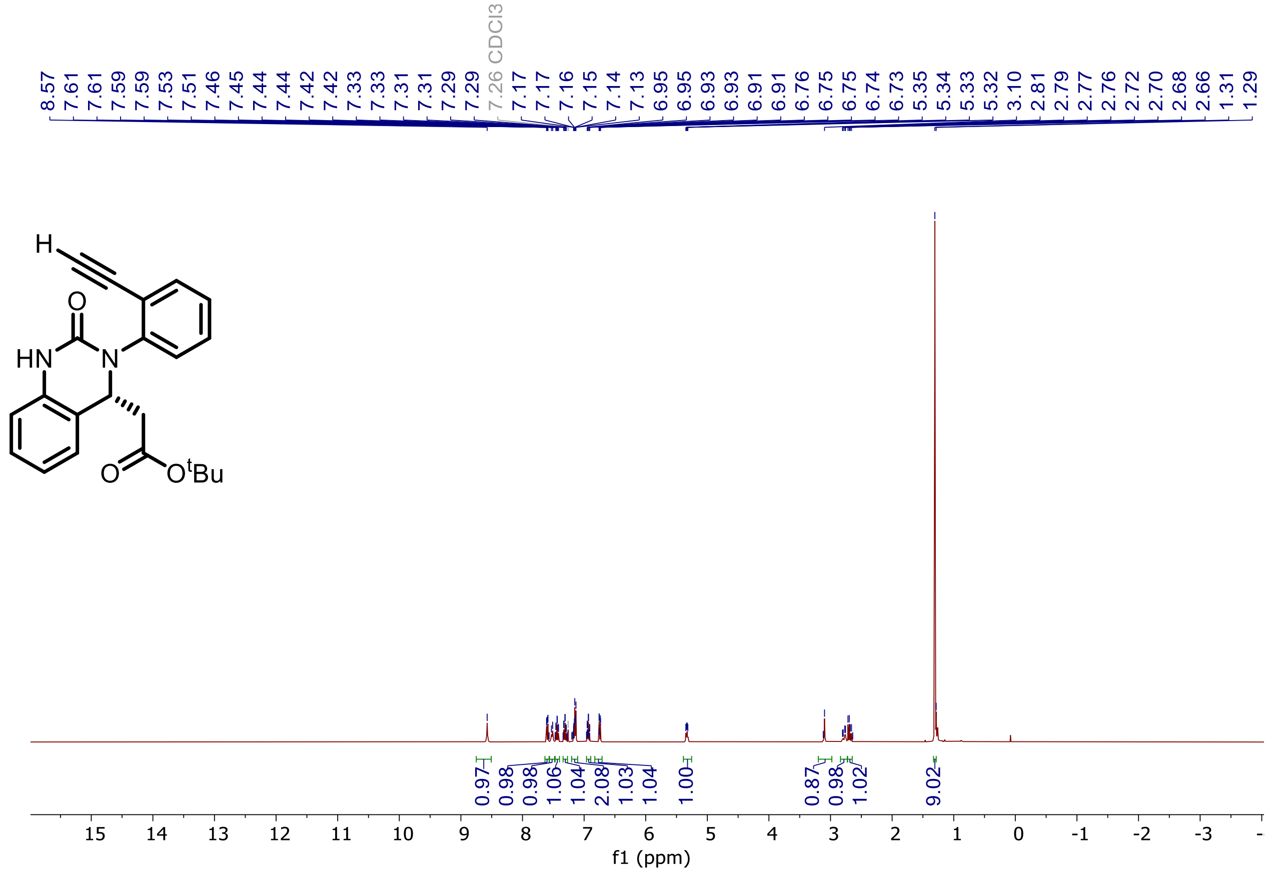

^{13}C NMR: (101 MHz, $CDCl_3$, 298K) of **2y**

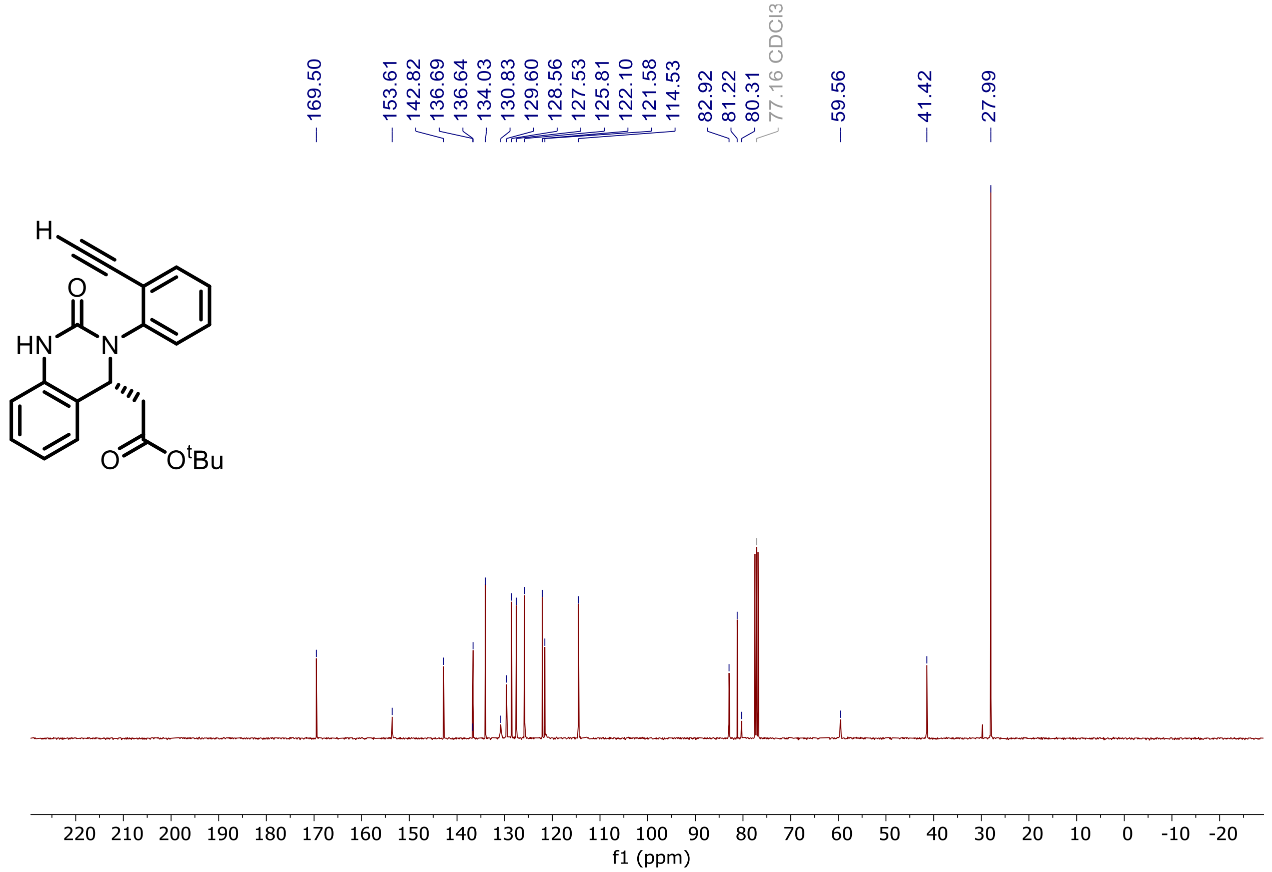

^{1}H NMR: (400 MHz, $CDCl_3$, 298K) of **2z**

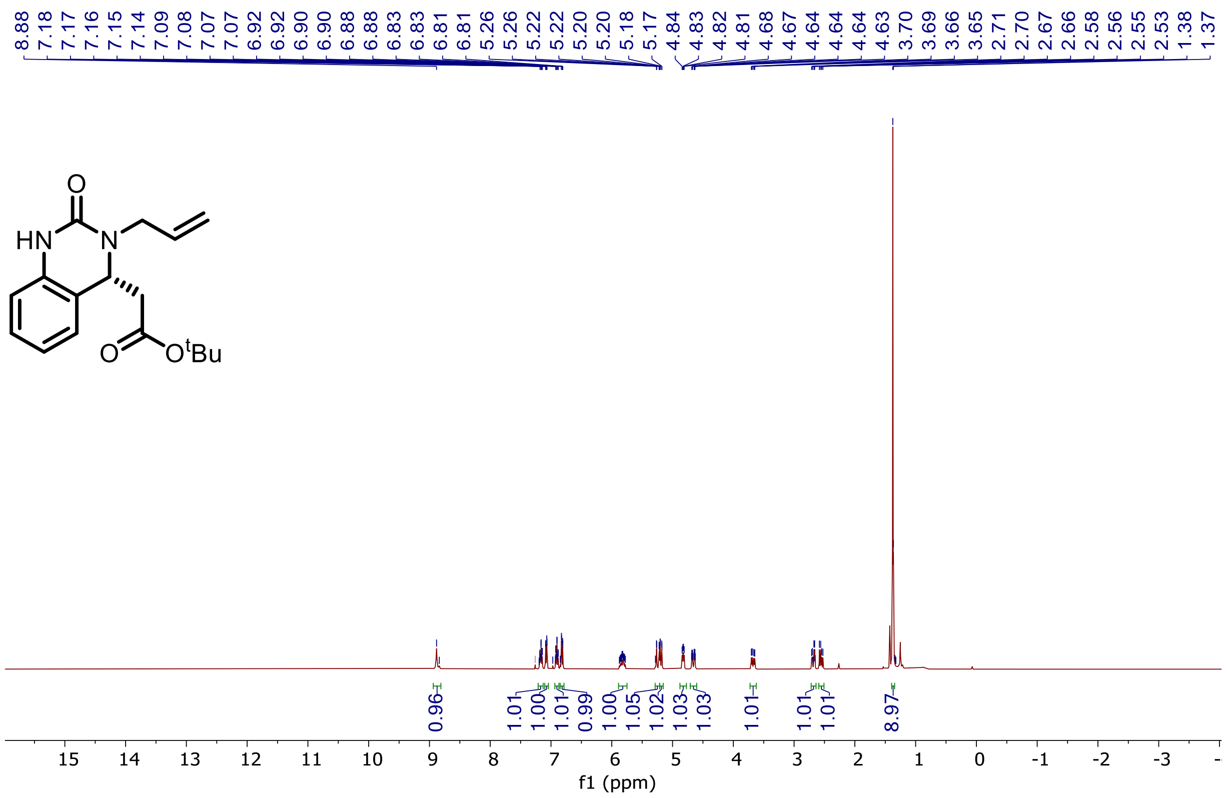

^{13}C NMR: (101 MHz, $CDCl_3$, 298K) of **2z**

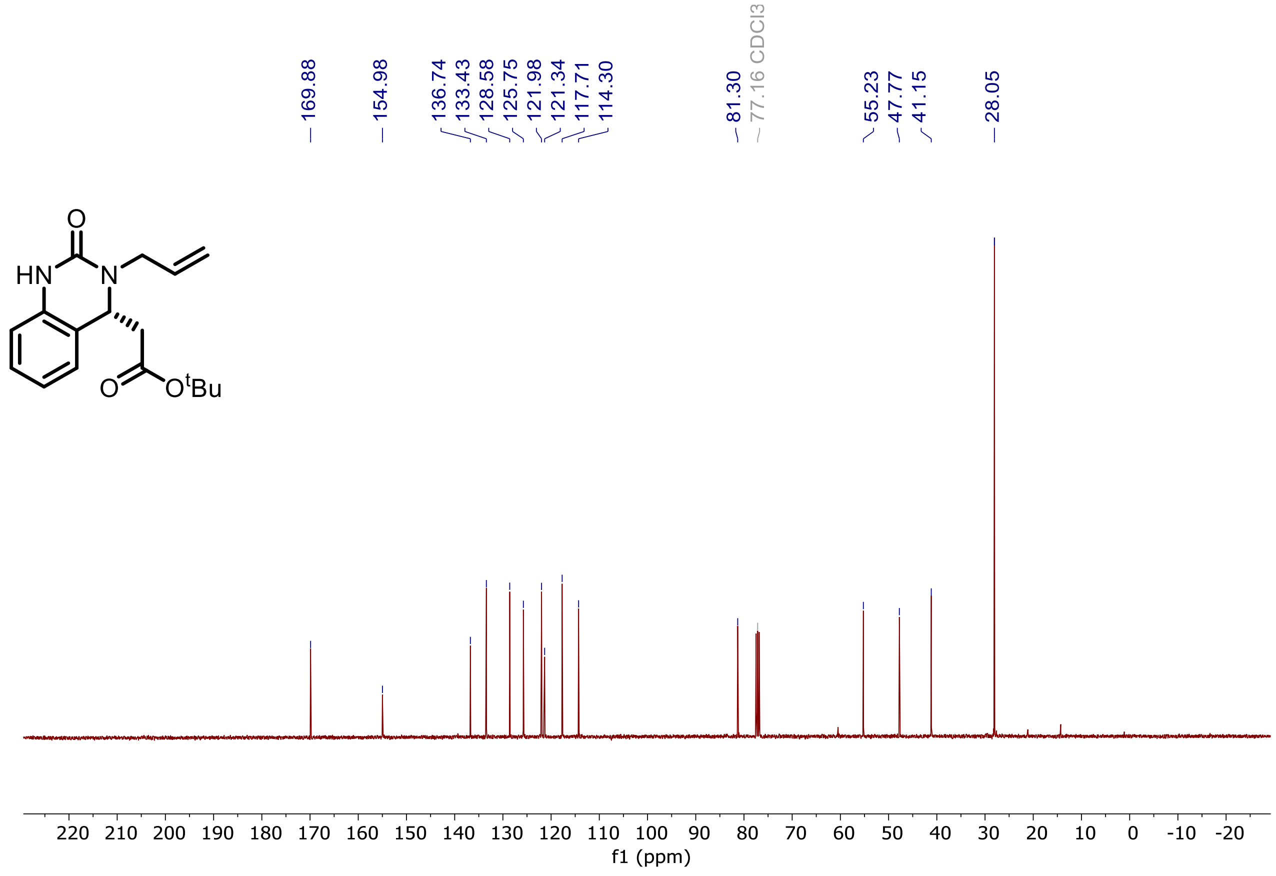

^{1}H NMR: (400 MHz, $CDCl_3$, 298K) of **2aa**

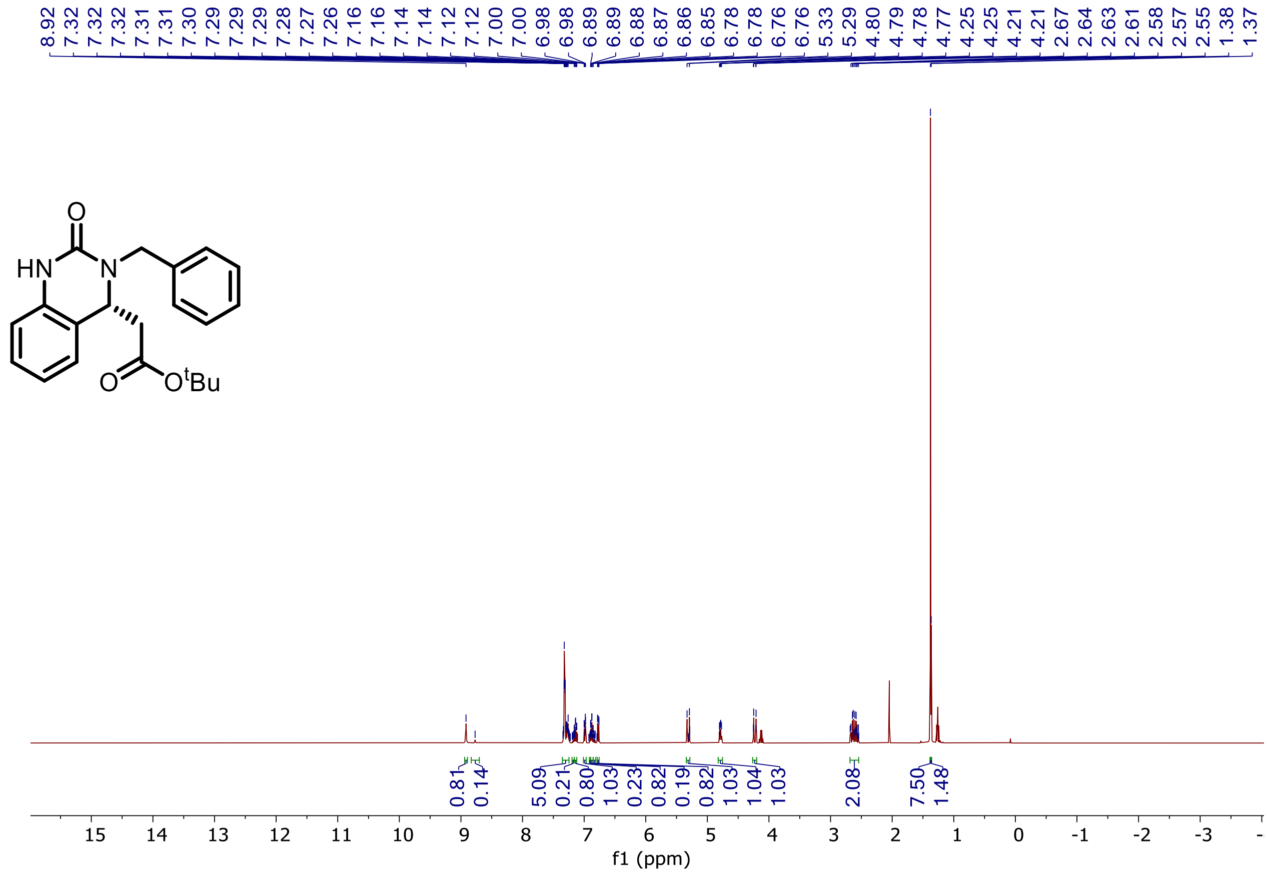

^{13}C NMR: (101 MHz, $CDCl_3$, 298K) of **2aa**

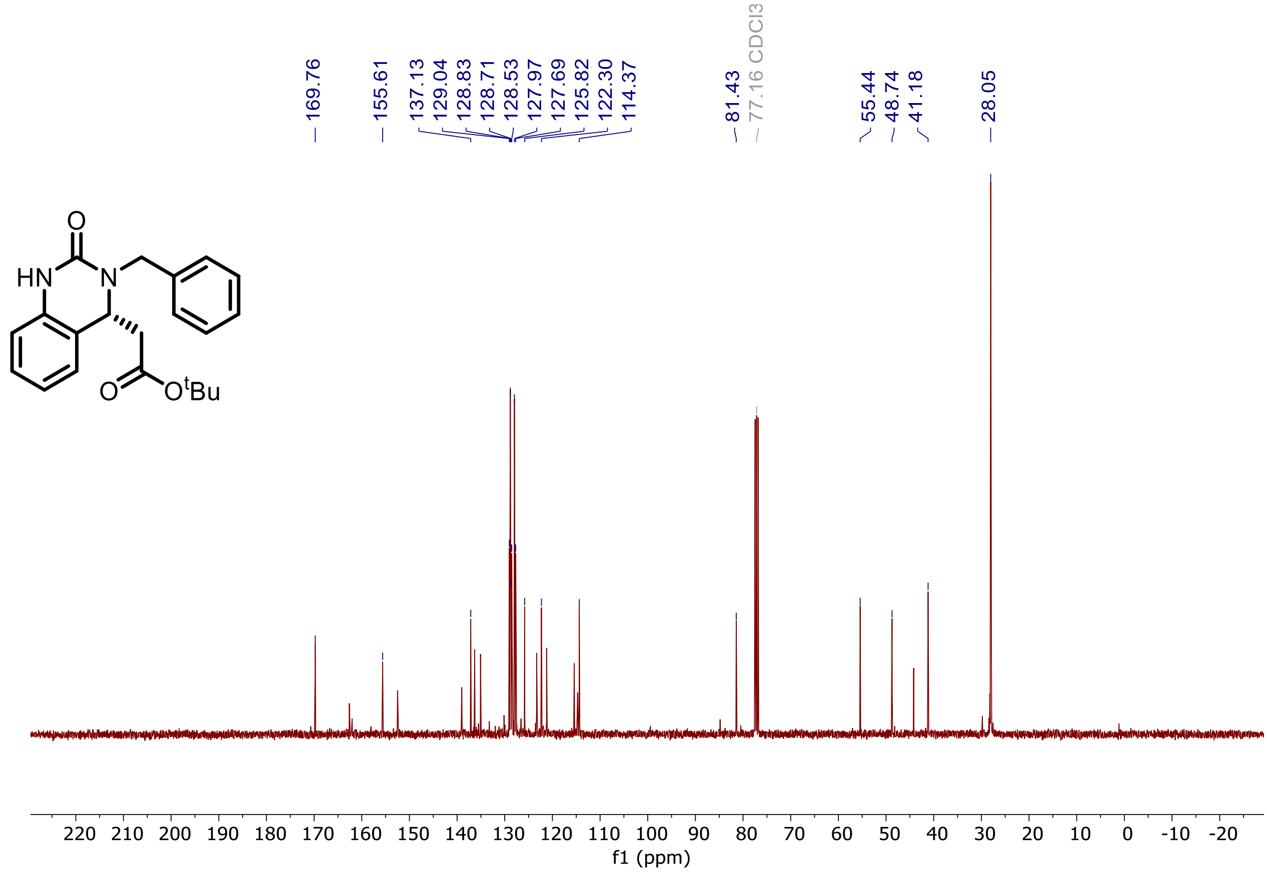

^{1}H NMR: (400 MHz, $CDCl_3$, 298K) of **2ab**

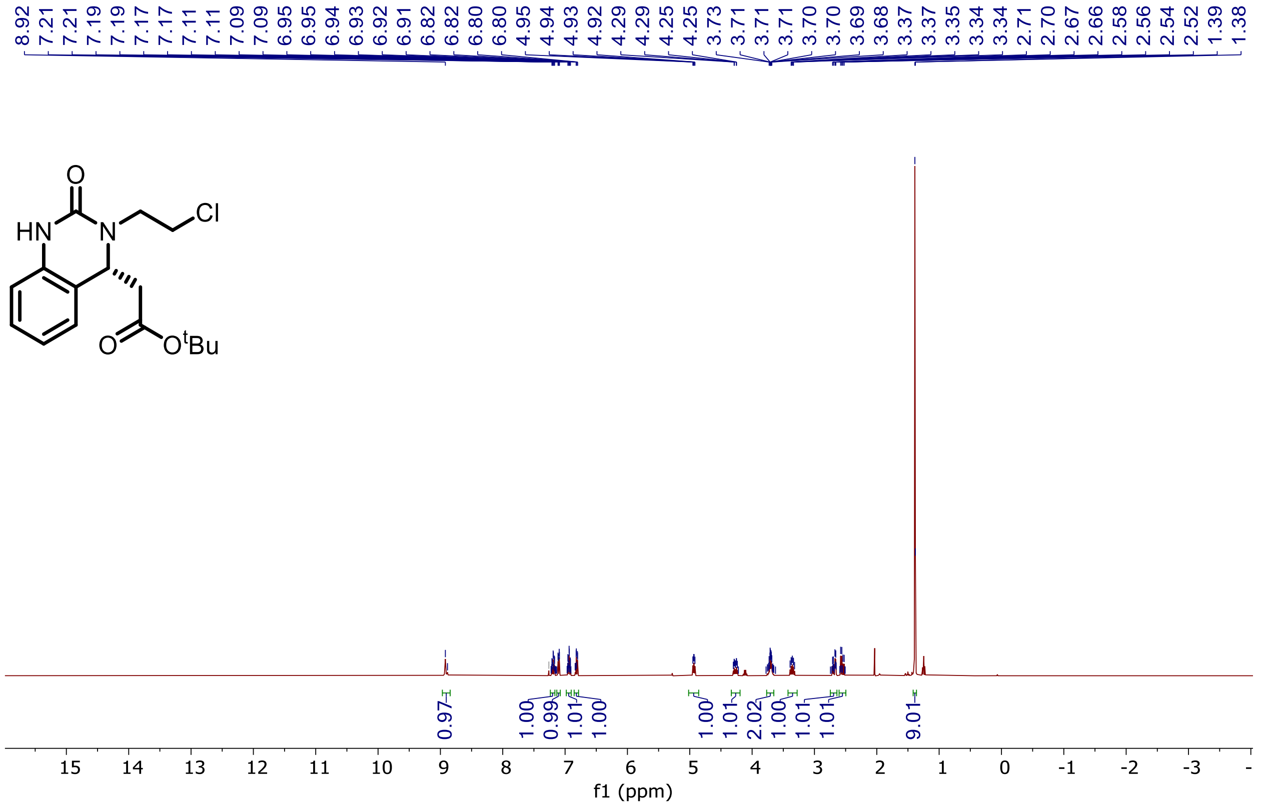

^{13}C NMR: (101 MHz, $CDCl_3$, 298K) of **2ab**

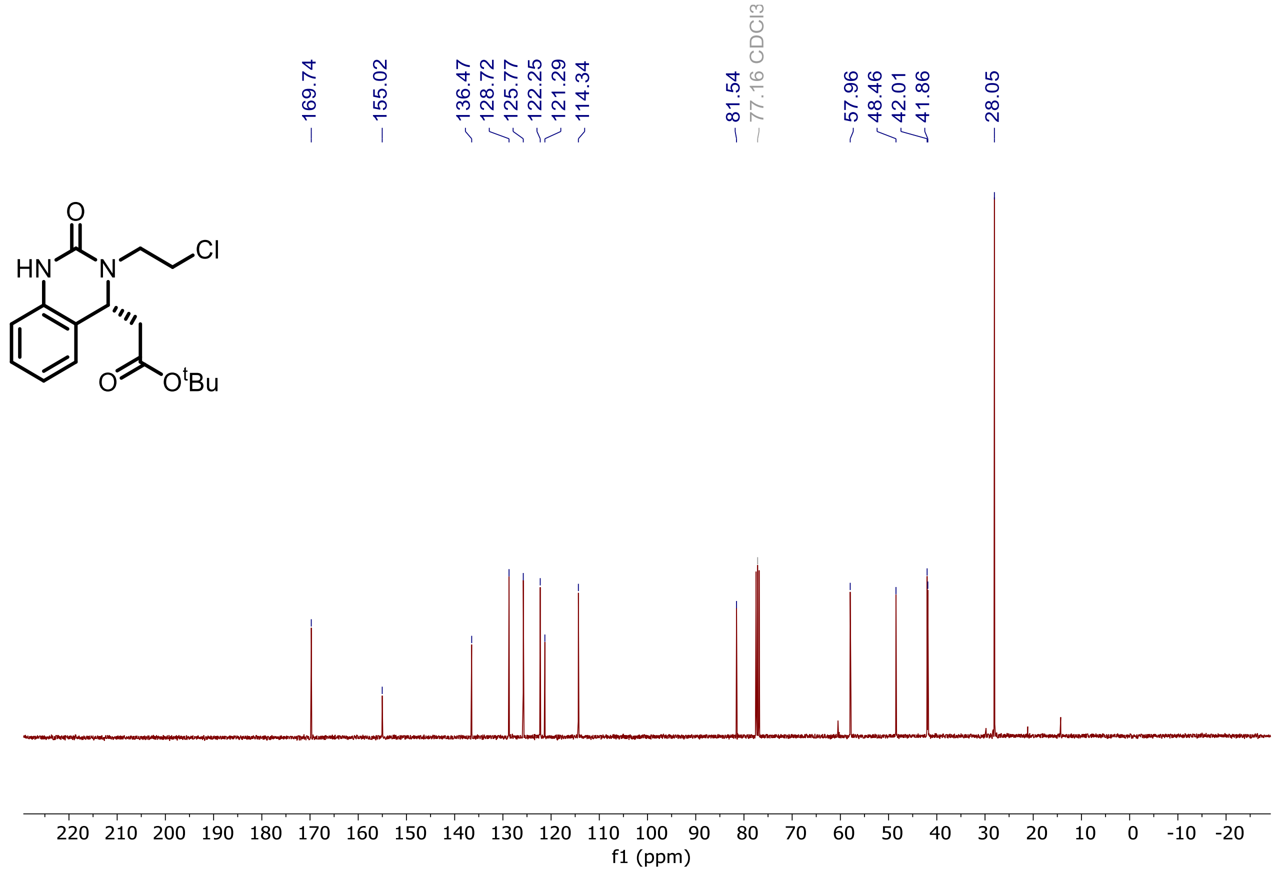

^{1}H NMR: (400 MHz, $CDCl_3$, 298K) of **2ac**

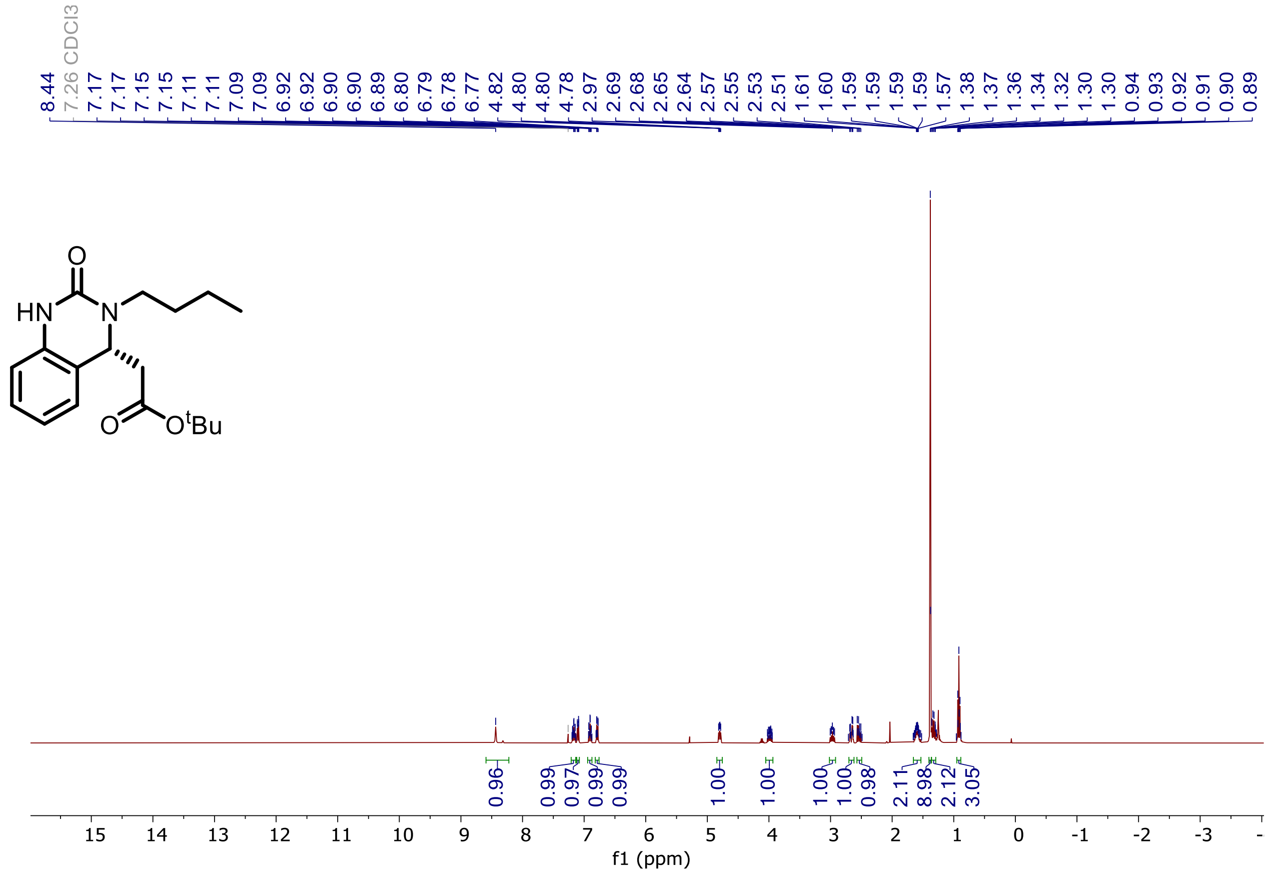

^{13}C NMR: (101 MHz, $CDCl_3$, 298K) of **2ac**

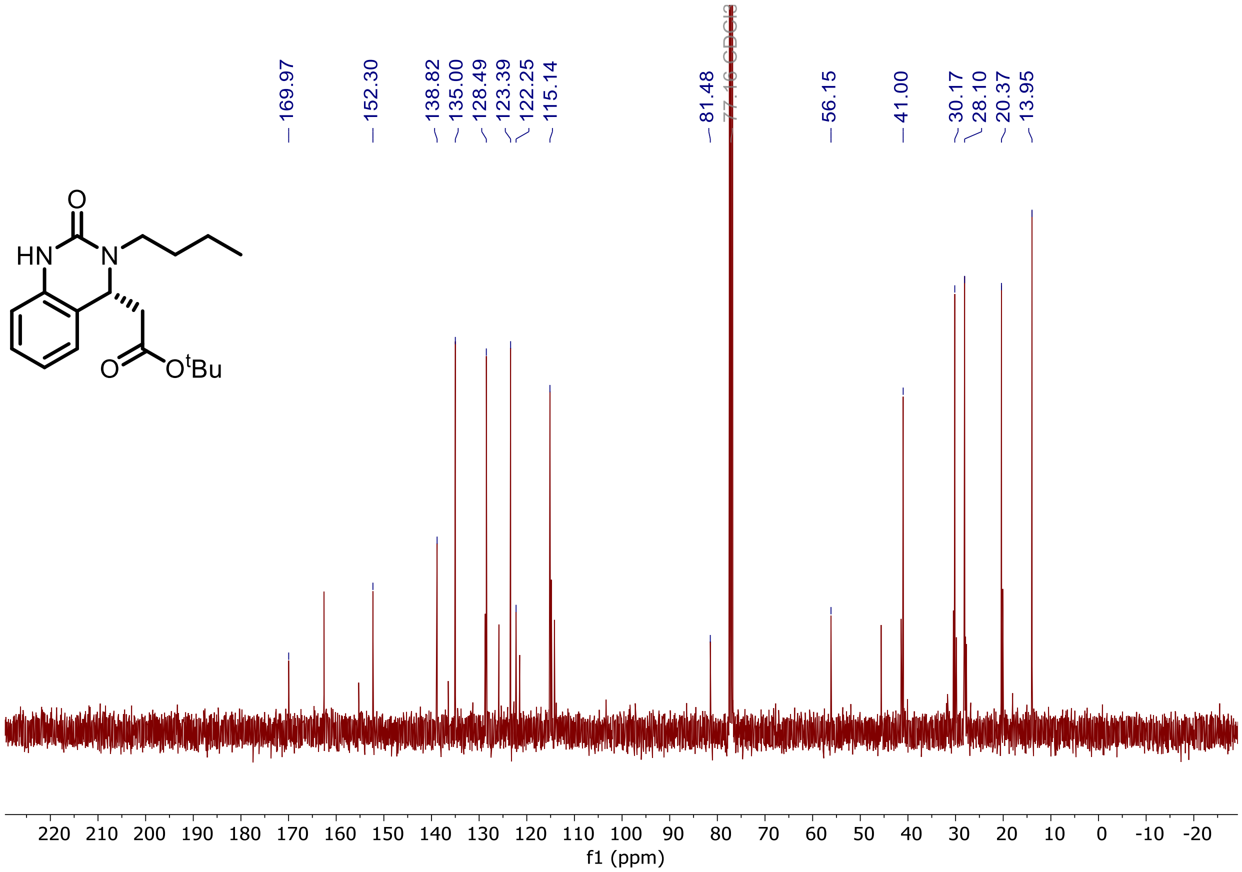

^{1}H NMR: (400 MHz, $CDCl_3$, 298K) of **2ad**

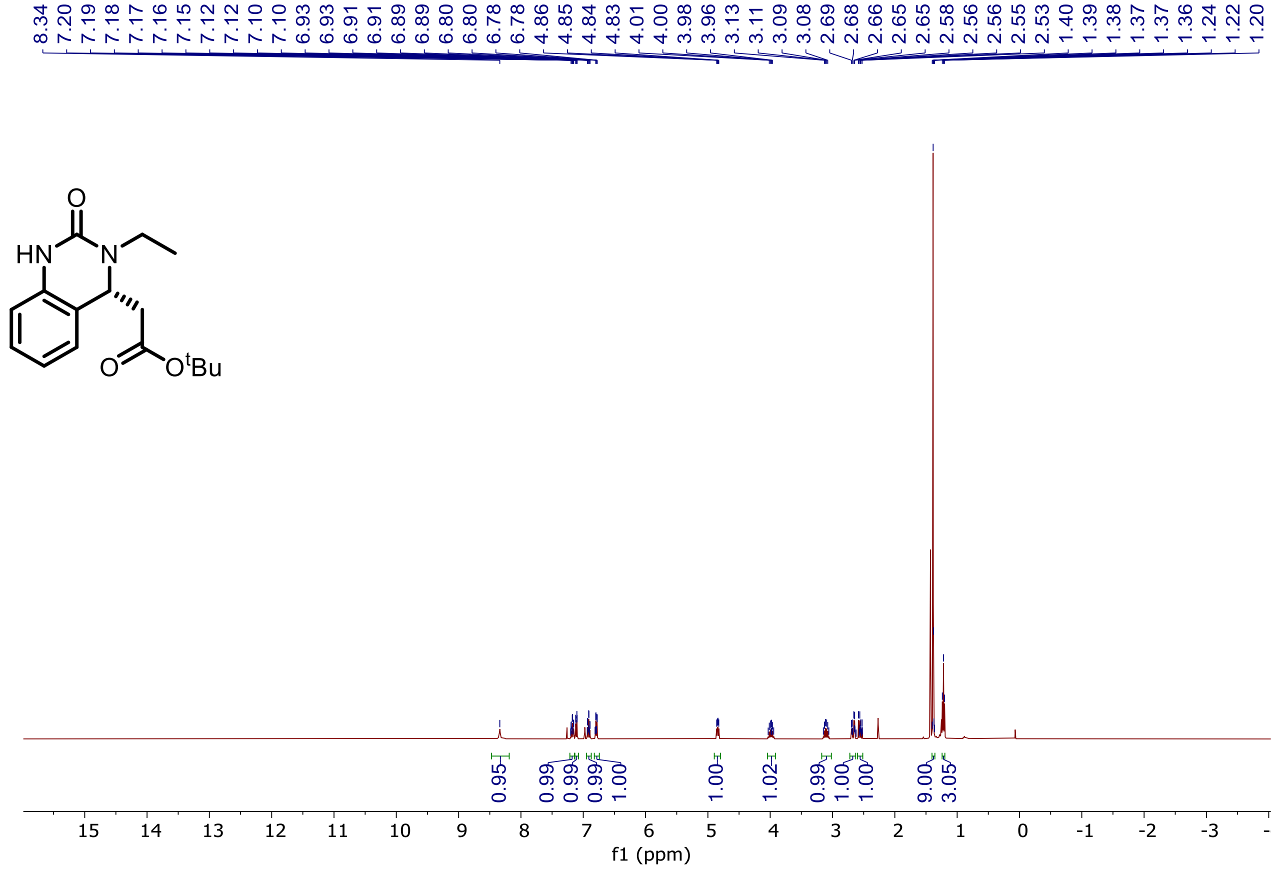

^{13}C NMR: (101 MHz, $CDCl_3$, 298K) of **2ad**

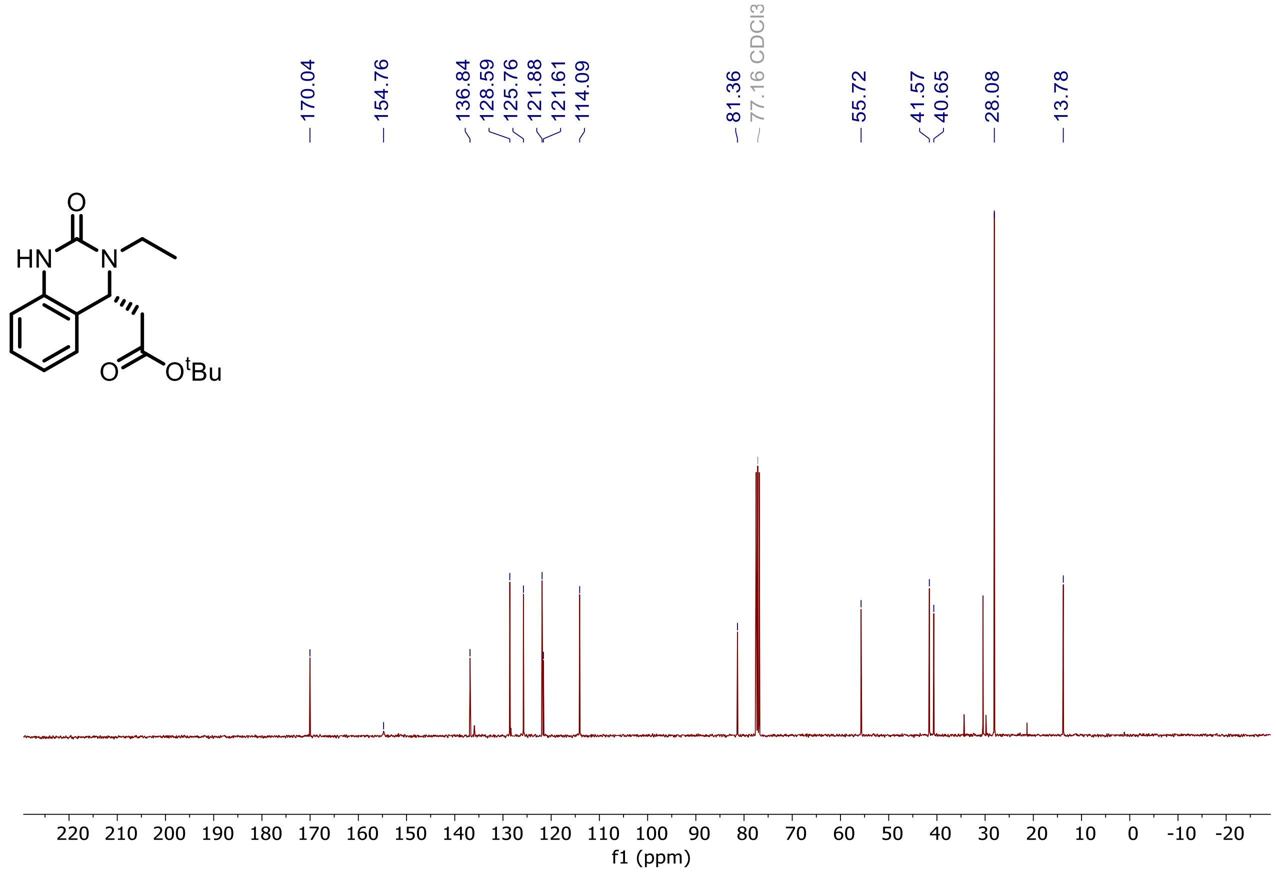

^{1}H NMR: (400 MHz, $CDCl_3$, 298K) of **2ae**

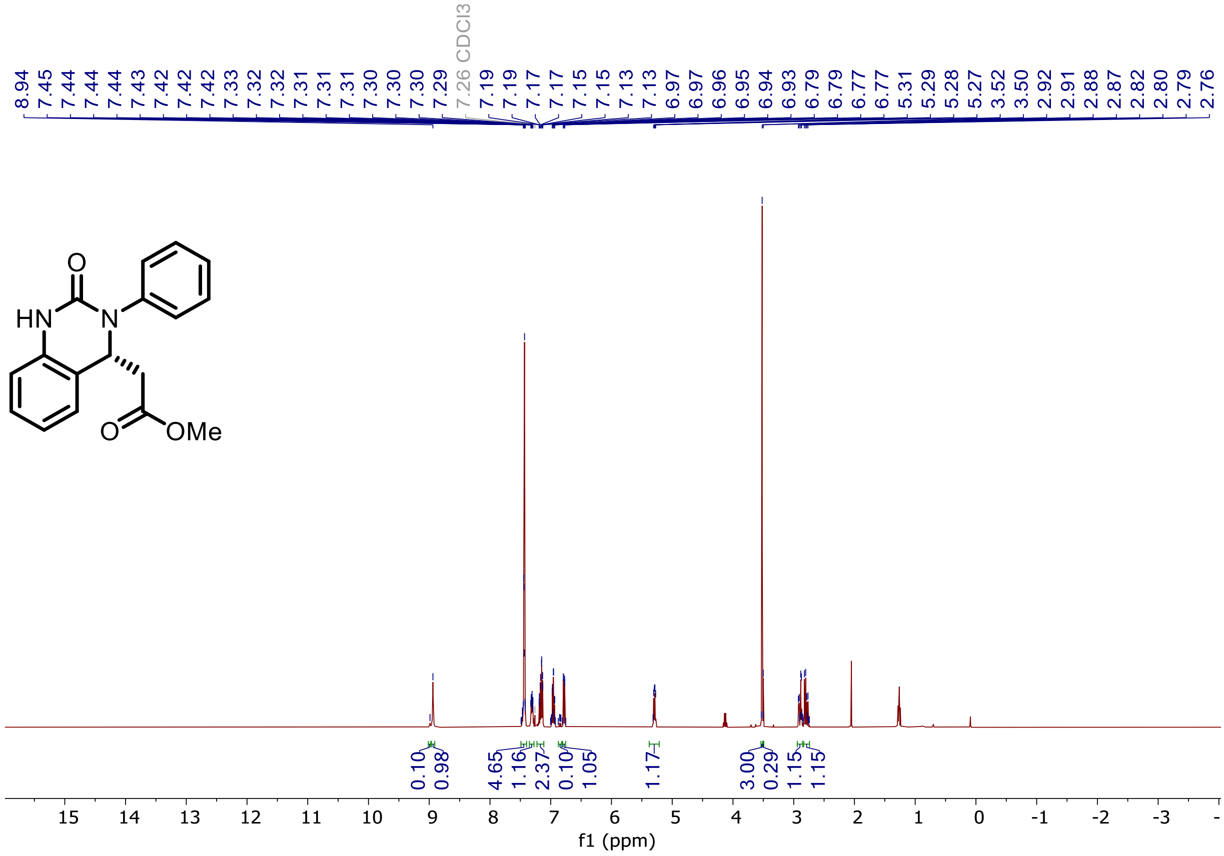

^{13}C NMR: (101 MHz, $CDCl_3$, 298K) of **2ae**

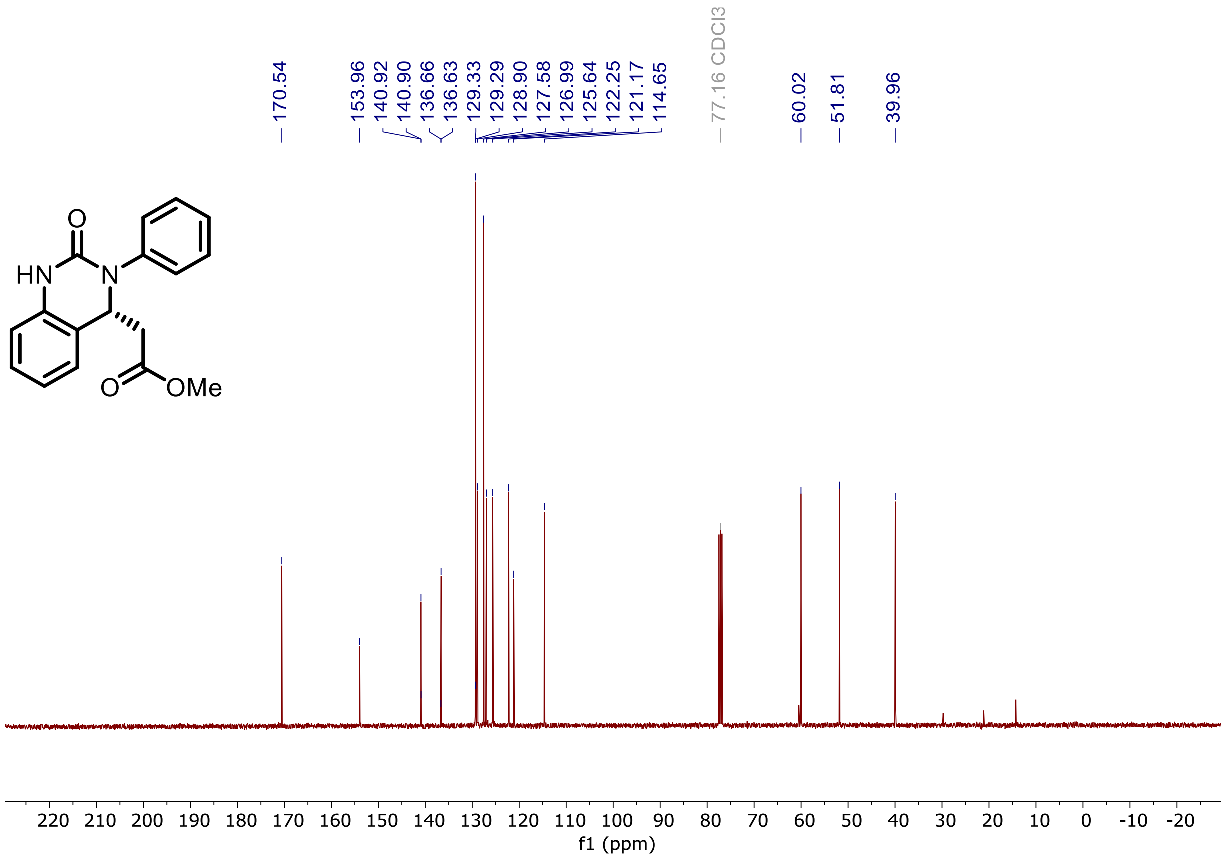

^{1}H NMR: (400 MHz, $CDCl_3$, 298K) of **3**

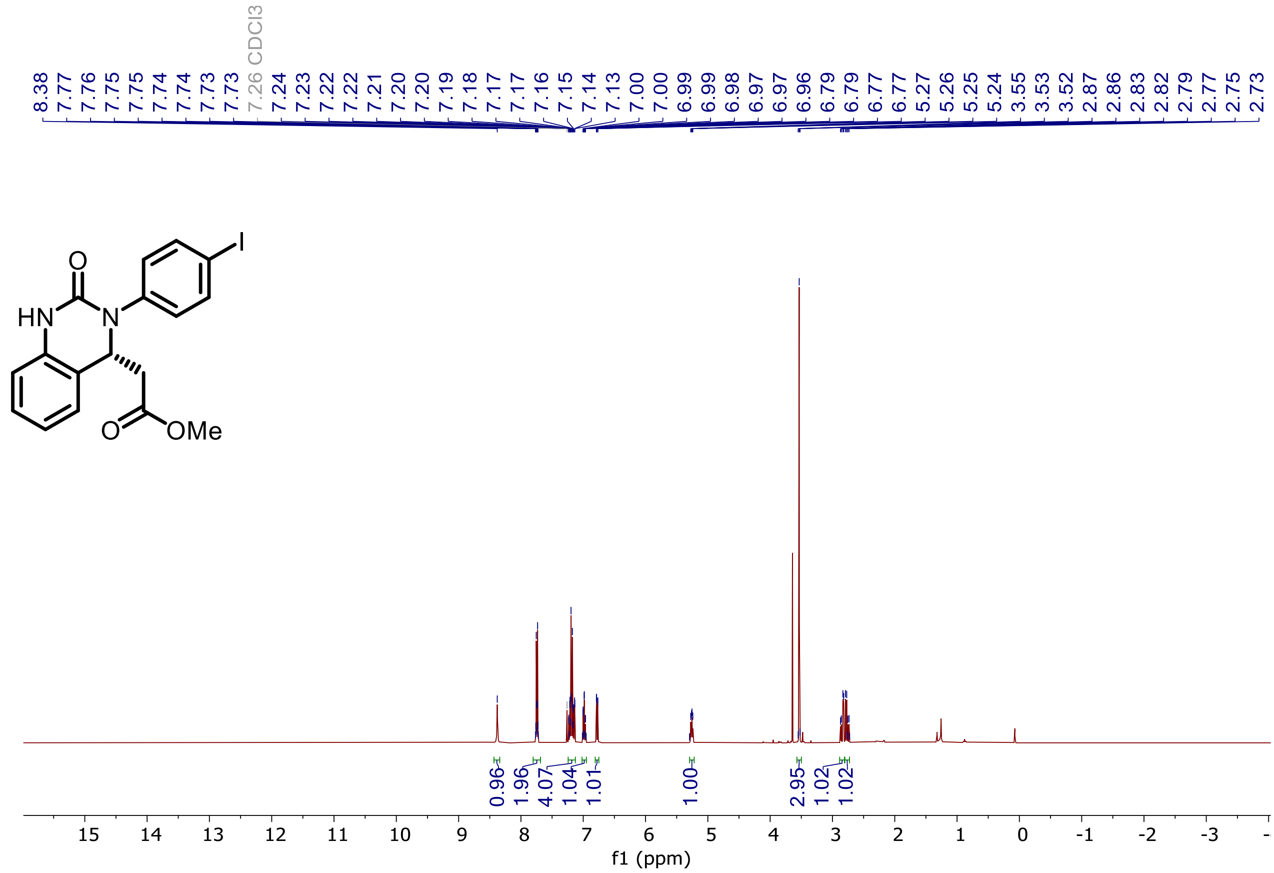

^{13}C NMR: (101 MHz, $CDCl_3$, 298K) of **3**

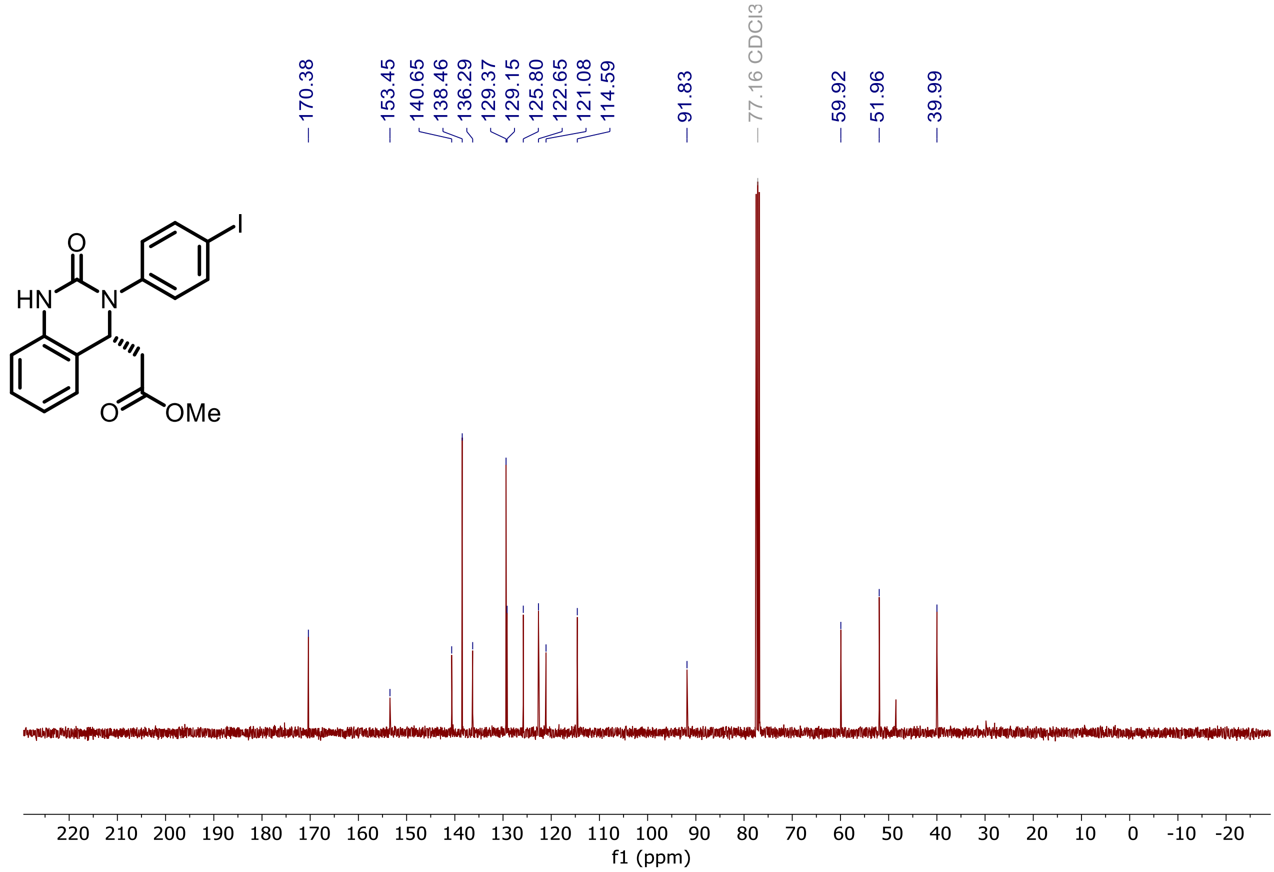

^{1}H NMR: (400 MHz, $CDCl_3$, 298K) of **4**

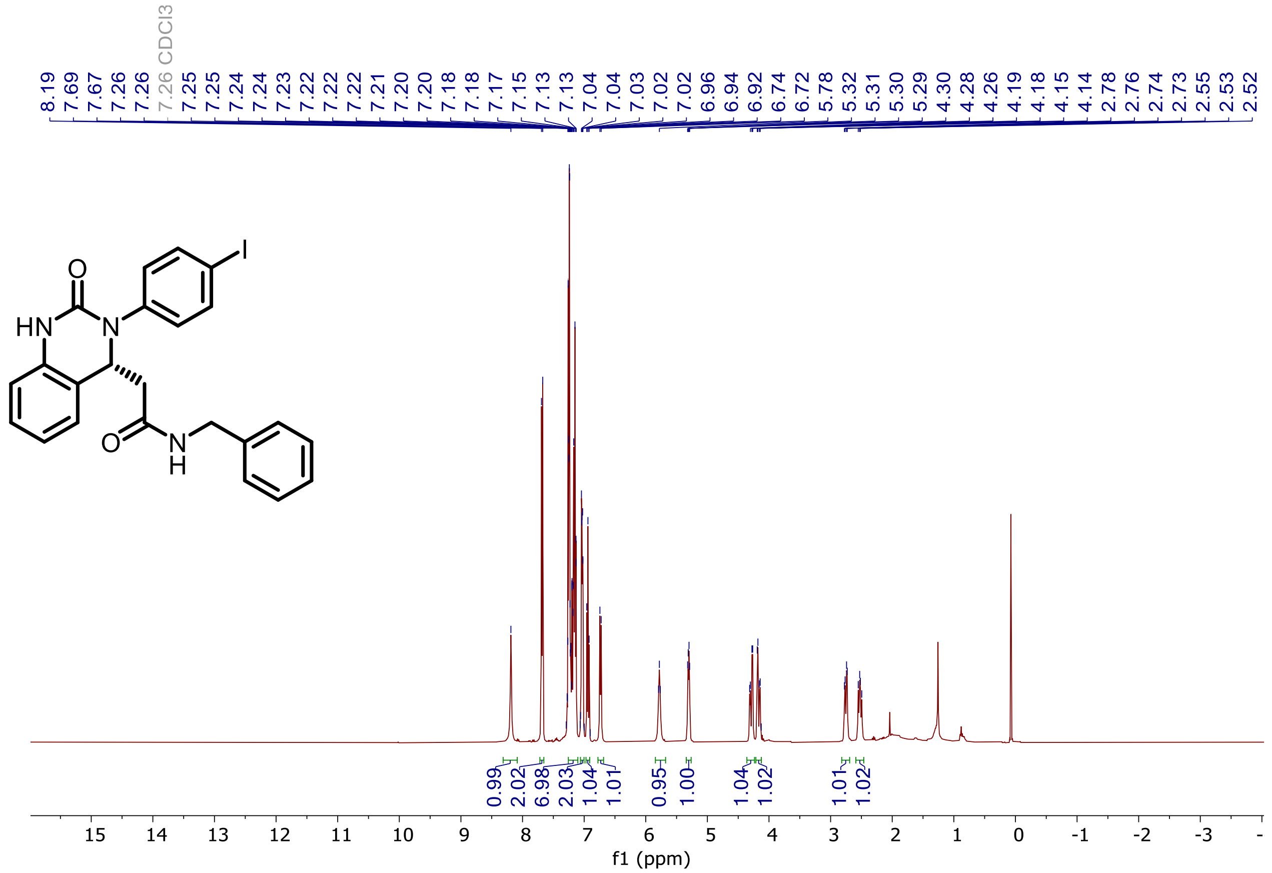

^{13}C NMR: (101 MHz, $CDCl_3$, 298K) of **4**

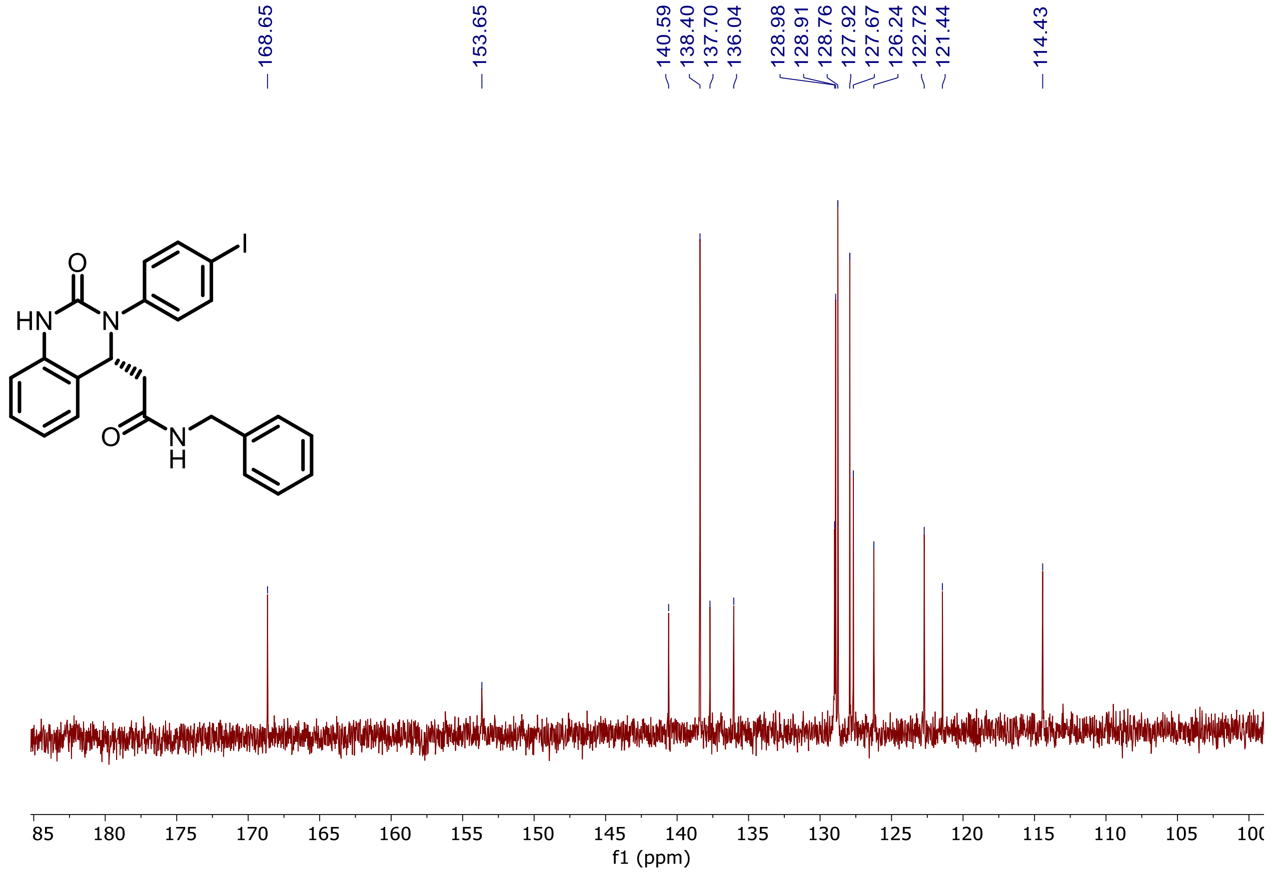

^{1}H NMR: (400 MHz, $CDCl_3$, 298K) of **5**

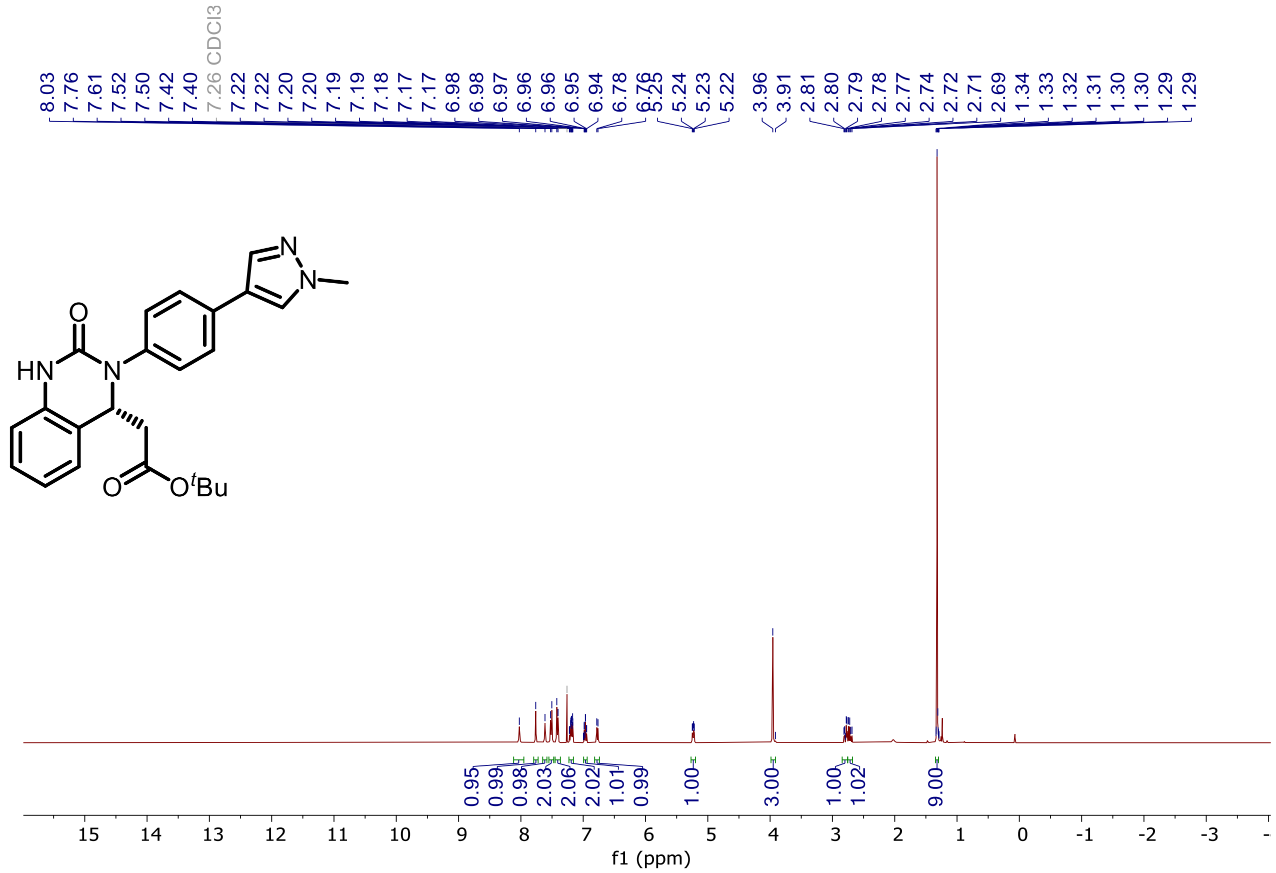

^{13}C NMR: (101 MHz, $CDCl_3$, 298K) of **5**

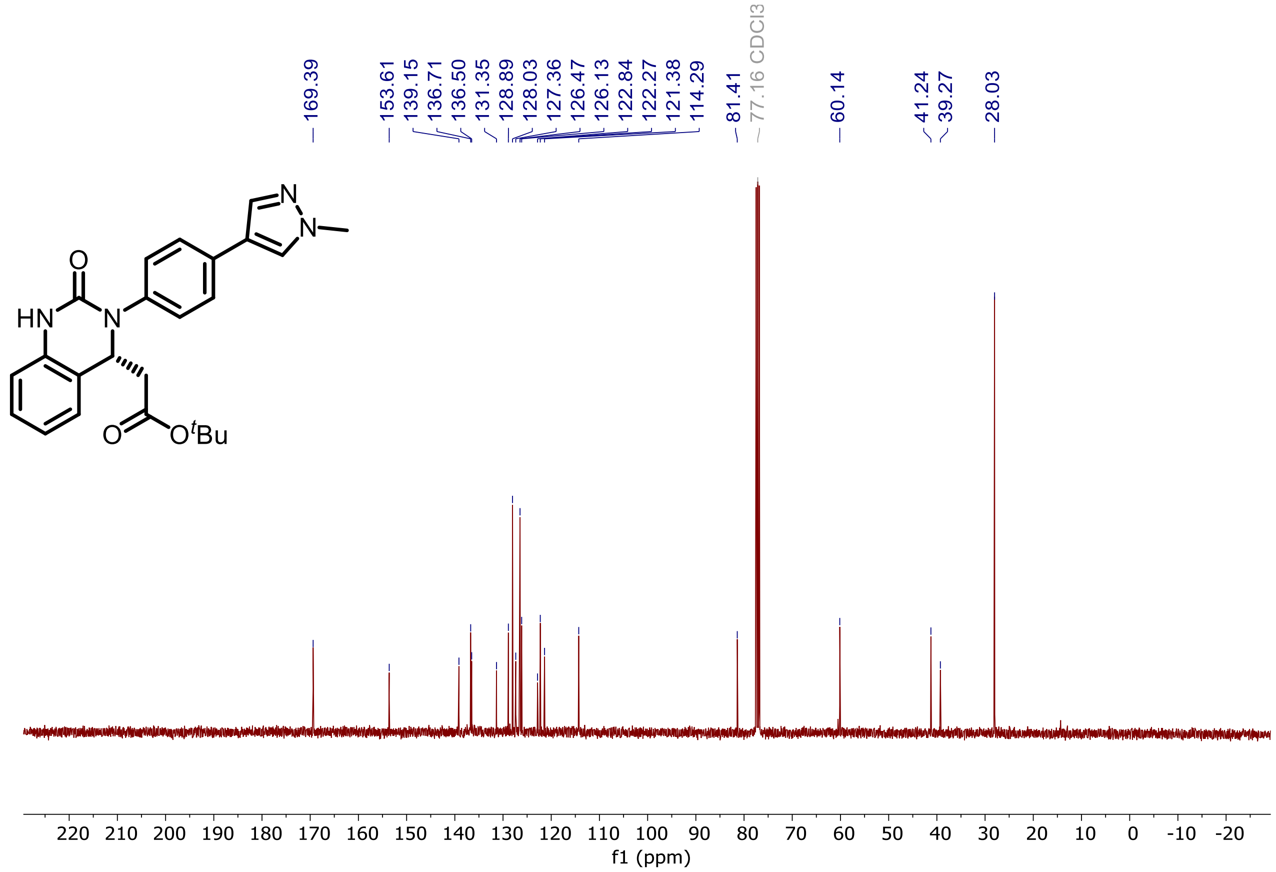

^{1}H NMR: (500 MHz, $CDCl_3$, 298K) of **6**

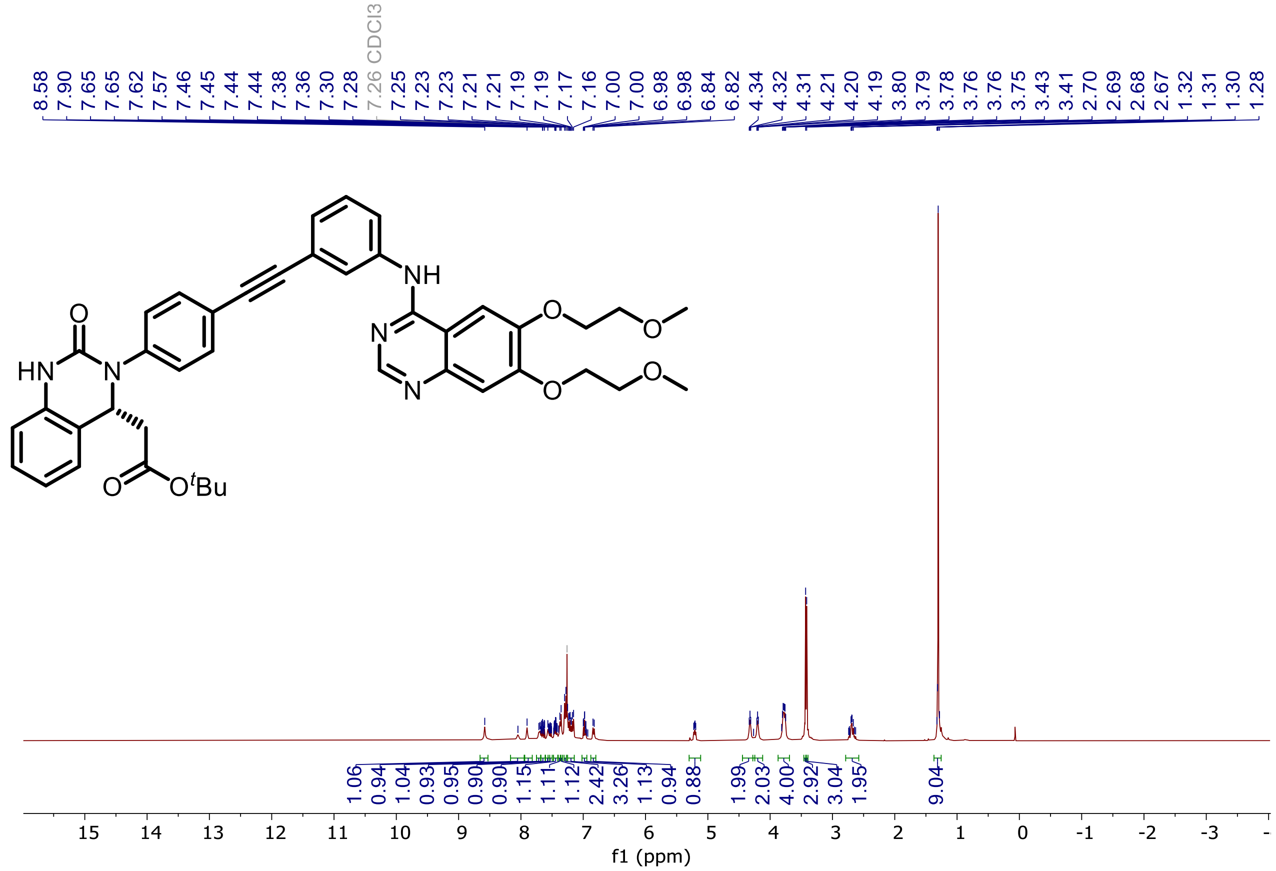

^{13}C NMR: (126 MHz, $CDCl_3$, 298K) of **6**

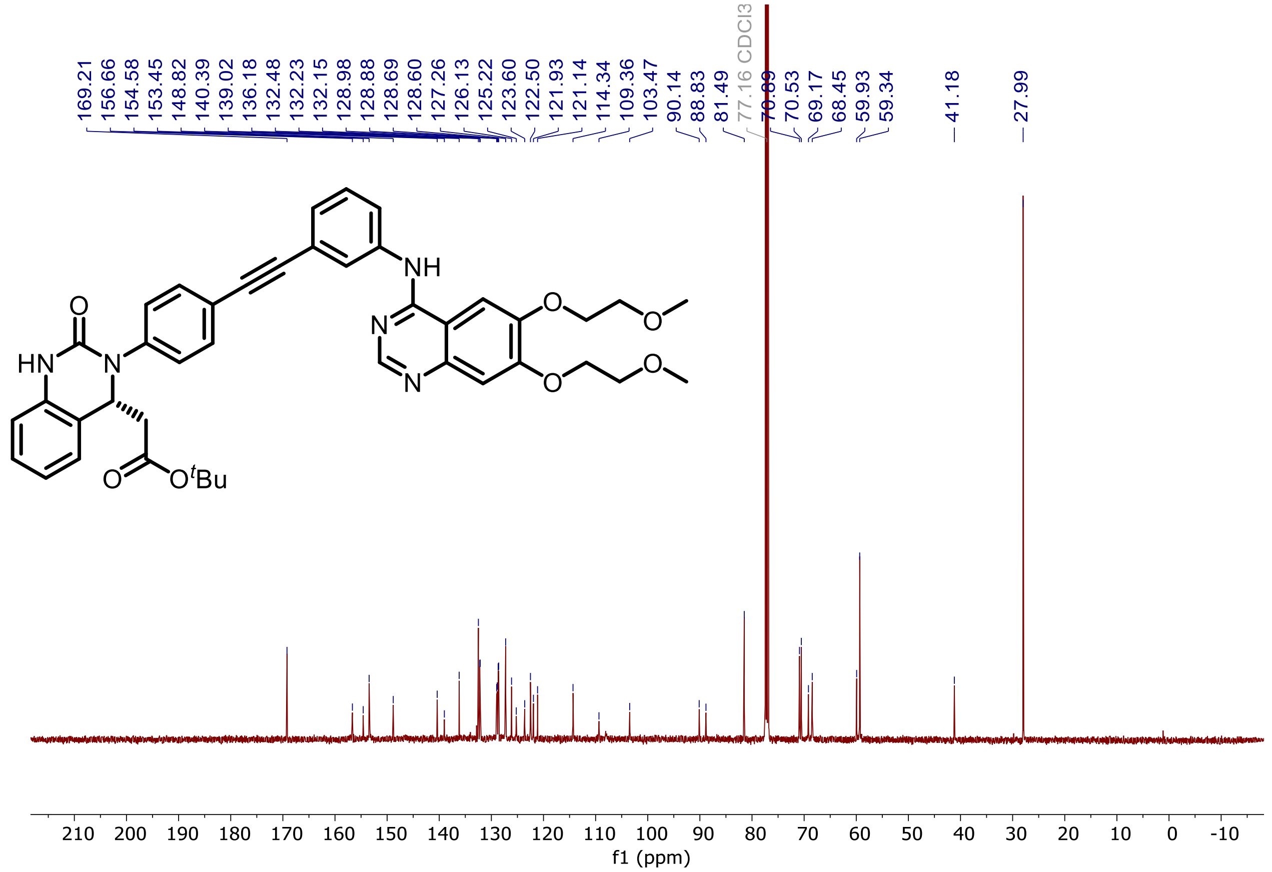

11. HPLC Traces

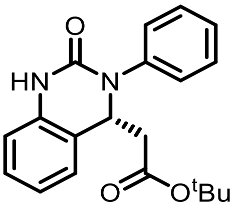

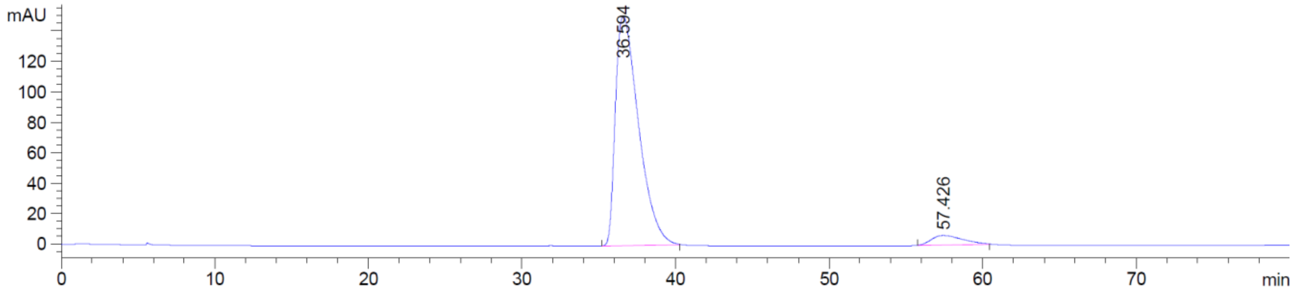

Peak #	RetTime [min]	Type	Width [min]	Area [mAU*s]	Height [mAU]	Area %
1	36.594	BB	1.6045	1.58348e4	150.87428	94.4764
2	57.426	BB	1.8581	925.77991	6.43639	5.5236

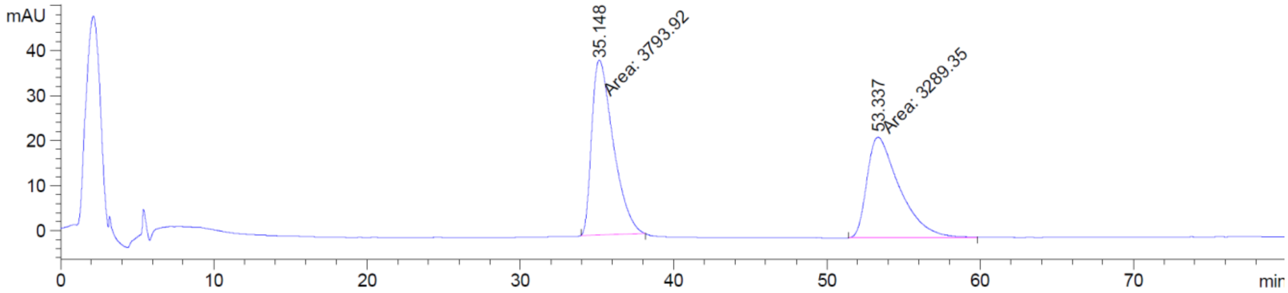

Peak #	RetTime [min]	Type	Width [min]	Area [mAU*s]	Height [mAU]	Area %
1	35.148	MM	1.6292	3793.91895	38.81215	53.5617
2	53.337	MM	2.4601	3289.34888	22.28424	46.4383

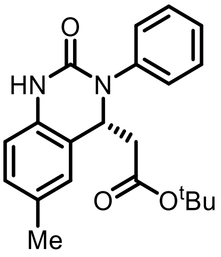

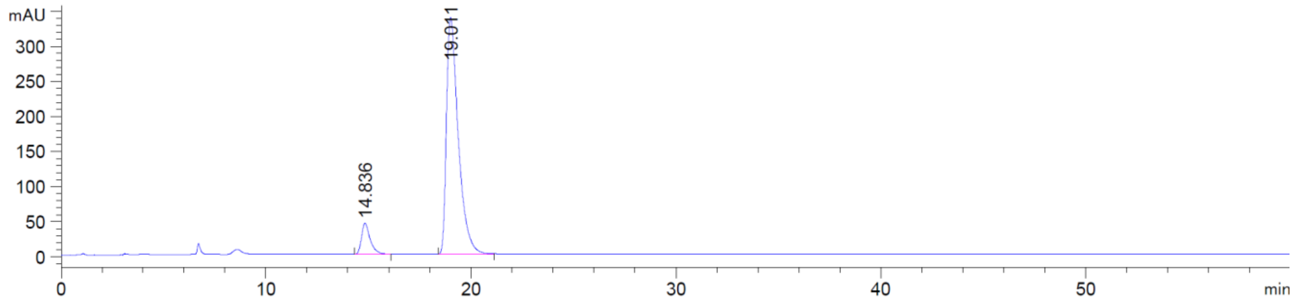

Peak #	RetTime [min]	Type	Width [min]	Area [mAU*s]	Height [mAU]	Area %
1	14.836	BB	0.4470	1315.30457	44.22499	8.8907
2	19.011	BB	0.5973	1.34788e4	337.99069	91.1093

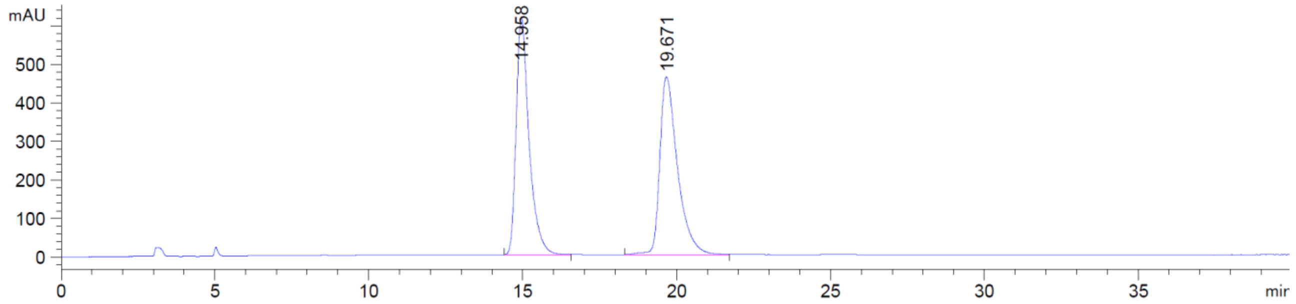

Peak #	RetTime [min]	Type	Width [min]	Area [mAU*s]	Height [mAU]	Area %
1	14.958	BB	0.4348	1.79203e4	617.08472	49.2532
2	19.671	BB	0.5906	1.84638e4	461.64569	50.7468

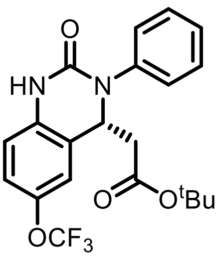

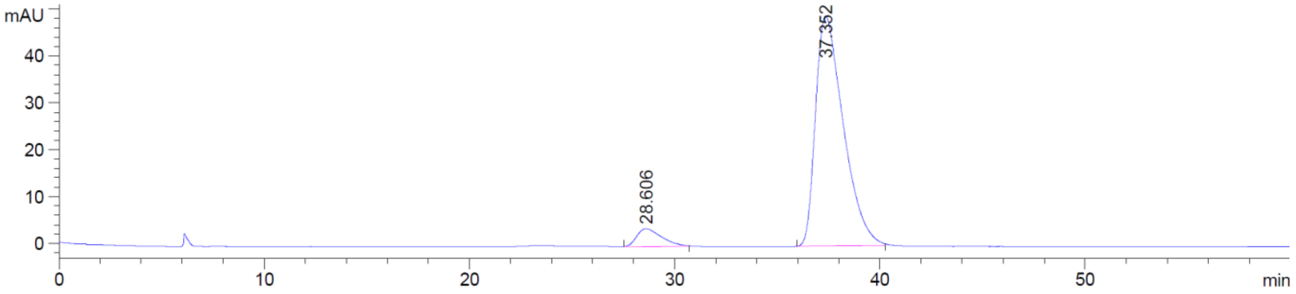

Peak #	RetTime [min]	Type	Width [min]	Area [mAU*s]	Height [mAU]	Area %
1	28.606	BB	1.1828	314.05084	3.75874	6.3706
2	37.352	BB	1.4179	4615.65234	49.04628	93.6294

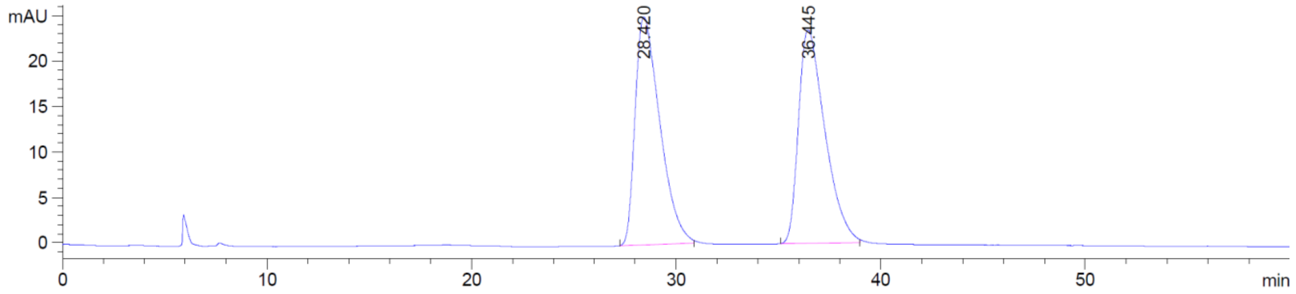

Peak #	RetTime [min]	Type	Width [min]	Area [mAU*s]	Height [mAU]	Area %
1	28.420	BB	1.2490	2112.56909	25.12407	50.0307
2	36.445	BB	1.3271	2109.97681	23.50944	49.9693

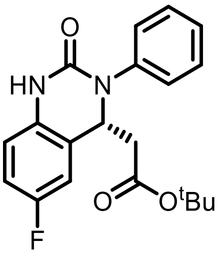

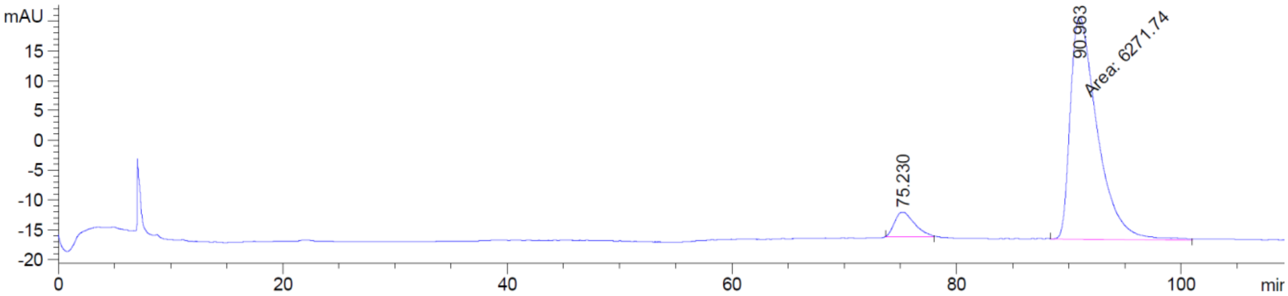

Peak #	RetTime [min]	Type	Width [min]	Area [mAU*s]	Height [mAU]	Area %
1	75.230	BB	1.4570	507.47949	4.12595	7.4858
2	90.963	MM	2.8015	6271.74316	37.31121	92.5142

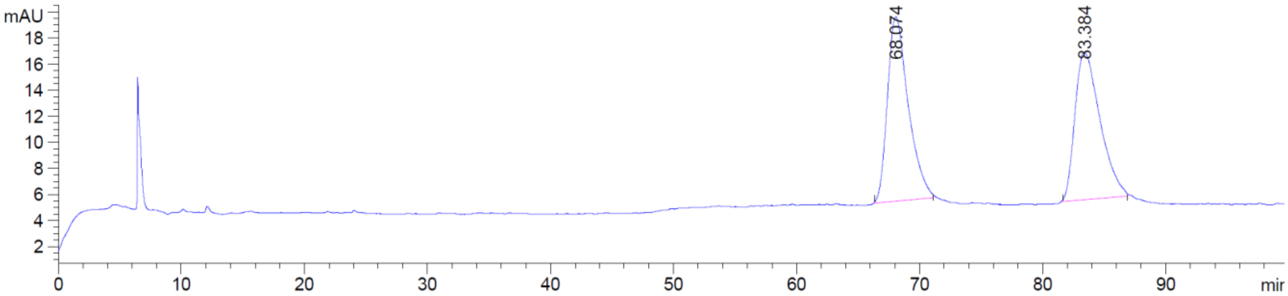

Peak #	RetTime [min]	Type	Width [min]	Area [mAU*s]	Height [mAU]	Area %
1	68.074	BB	1.7295	1667.94092	14.14712	50.9580
2	83.384	BB	1.9079	1605.22437	11.33691	49.0420

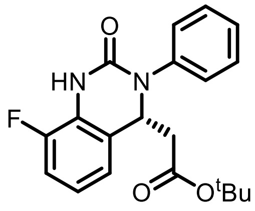

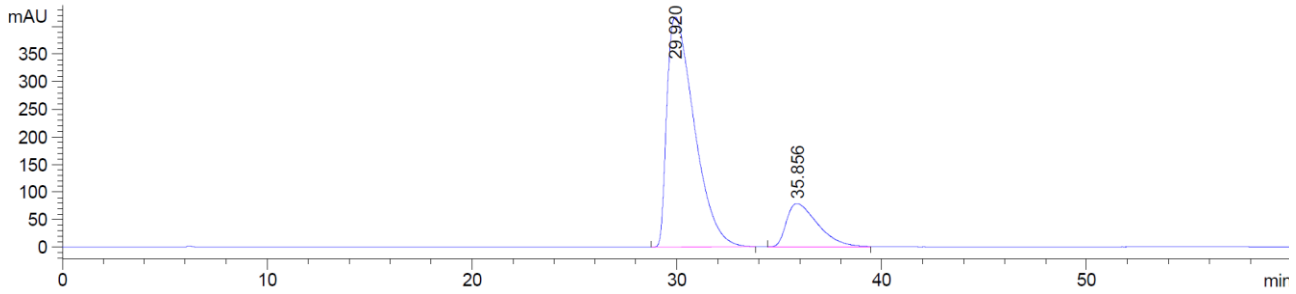

Peak #	RetTime [min]	Type	Width [min]	Area [mAU*s]	Height [mAU]	Area %
1	29.920	BB	1.4579	3.87014e4	417.32169	82.3546
2	35.856	BB	1.6107	8292.24512	78.35205	17.6454

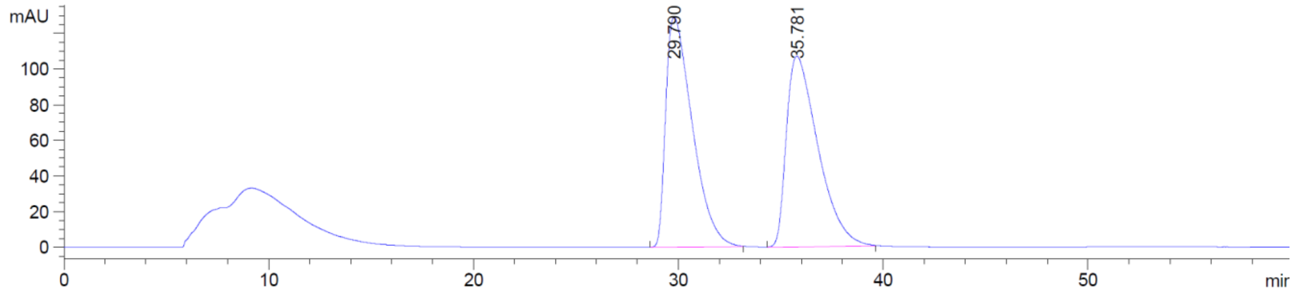

Peak #	RetTime [min]	Type	Width [min]	Area [mAU*s]	Height [mAU]	Area %
1	29.790	BB	1.3804	1.16564e4	129.22141	50.3846
2	35.781	BB	1.6443	1.14785e4	106.93541	49.6154

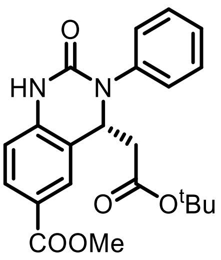

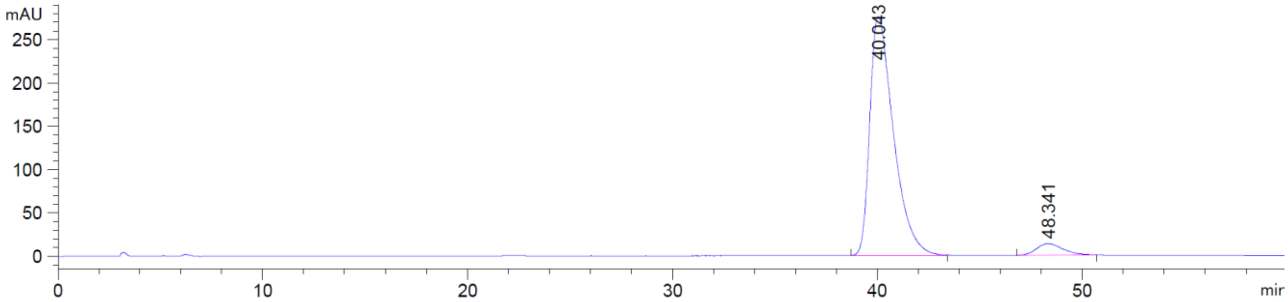

Peak #	RetTime [min]	Type	Width [min]	Area [mAU*s]	Height [mAU]	Area %
1	40.043	BB	1.2111	2.21648e4	275.93494	94.7487
2	48.341	BB	1.1898	1228.45886	13.34732	5.2513

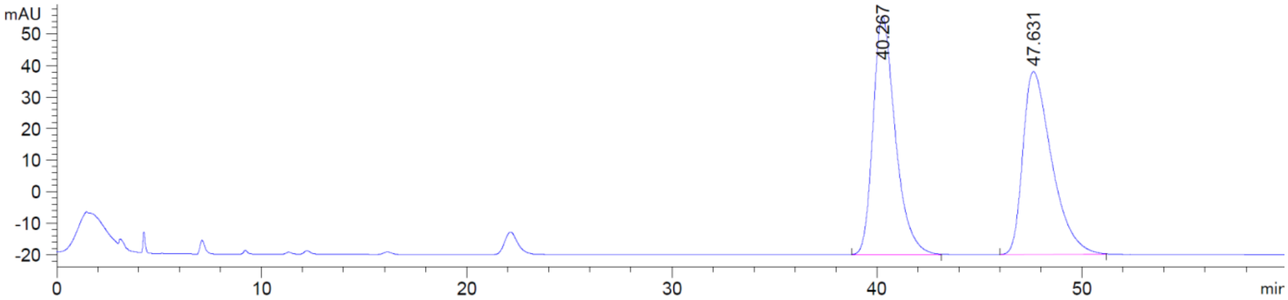

Peak #	RetTime [min]	Type	Width [min]	Area [mAU*s]	Height [mAU]	Area %
1	40.267	BB	1.1521	5702.44727	75.59467	50.1000
2	47.631	BB	1.4824	5679.67871	58.00195	49.9000

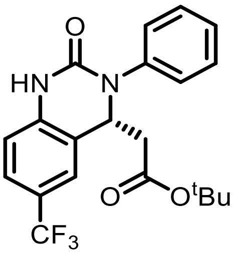

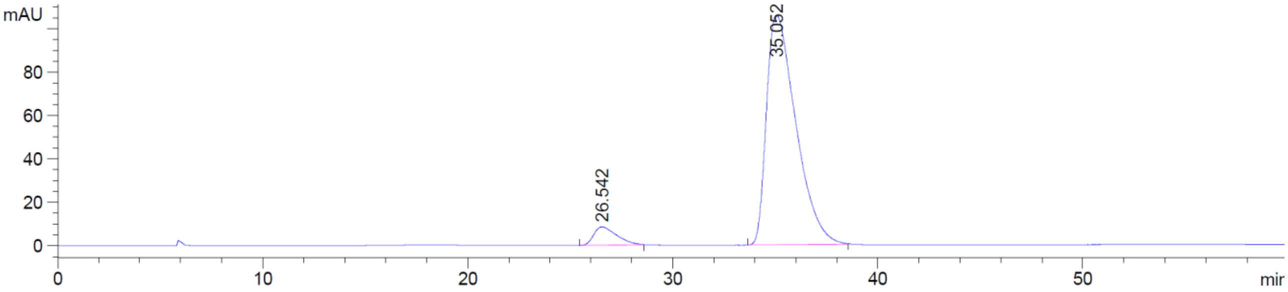

Peak #	RetTime [min]	Type	Width [min]	Area [mAU*s]	Height [mAU]	Area %
1	26.542	BB	1.1373	658.87781	8.41619	5.8726
2	35.052	BB	1.4953	1.05607e4	105.91005	94.1274

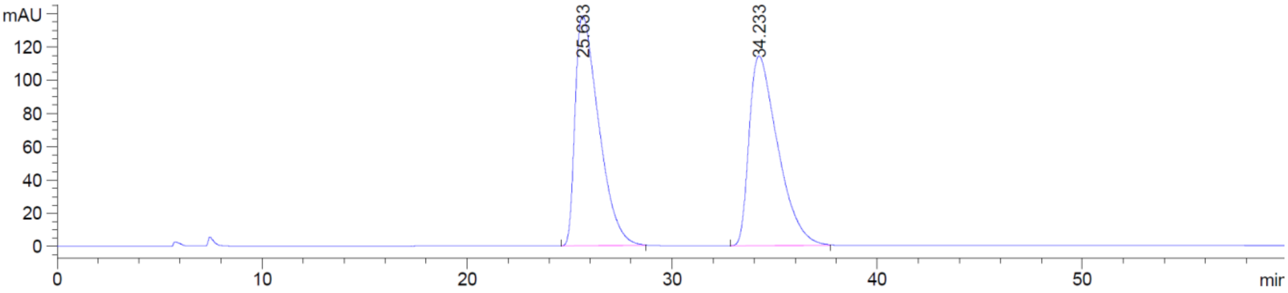

Peak #	RetTime [min]	Type	Width [min]	Area [mAU*s]	Height [mAU]	Area %
1	25.633	BB	1.2205	1.11071e4	138.37029	50.0671
2	34.233	BB	1.4704	1.10773e4	113.92096	49.9329

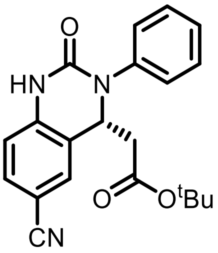

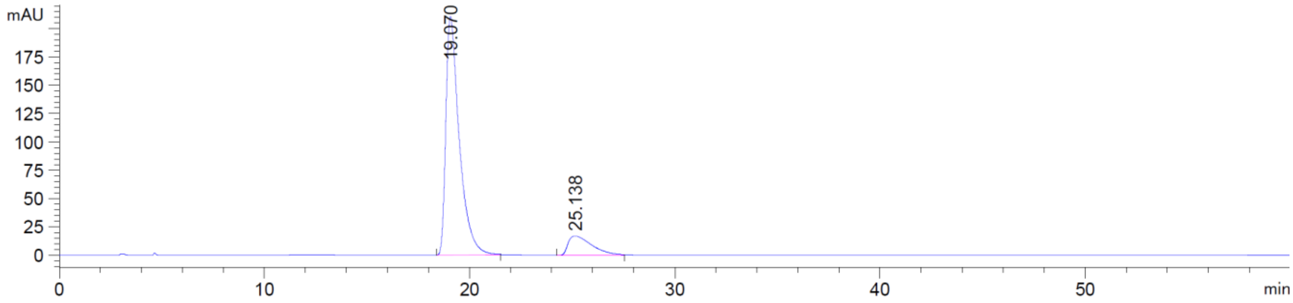

Peak #	RetTime [min]	Type	Width [min]	Area [mAU*s]	Height [mAU]	Area %
1	19.070	BB	0.6744	9580.83105	210.72720	87.7011
2	25.138	BB	1.1126	1343.58606	16.95407	12.2989

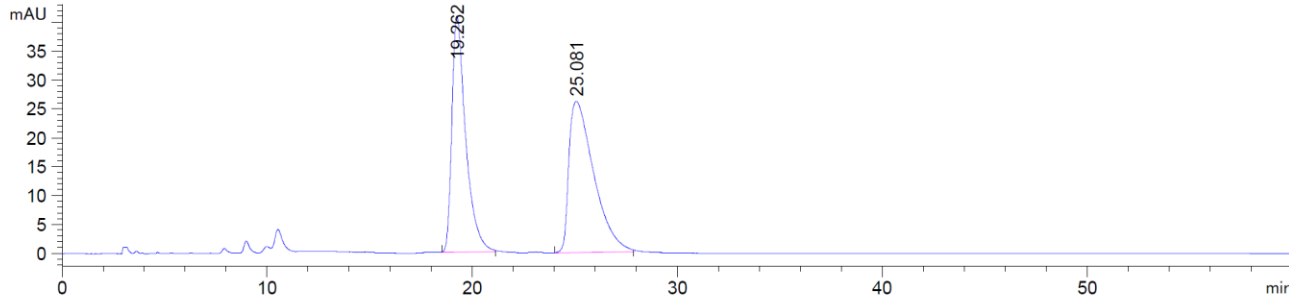

Peak #	RetTime [min]	Type	Width [min]	Area [mAU*s]	Height [mAU]	Area %
1	19.262	BB	0.6906	1914.94189	40.87558	46.4549
2	25.081	BB	1.2721	2207.20728	26.16794	53.5451

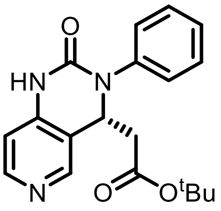

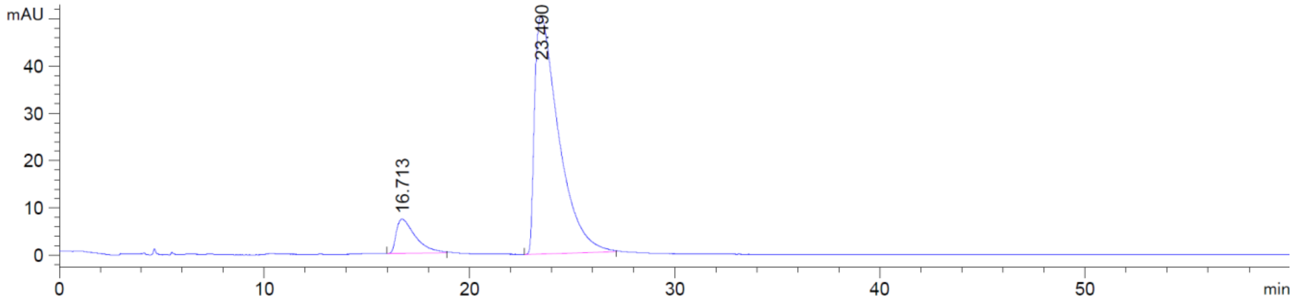

Peak #	RetTime [min]	Type	Width [min]	Area [mAU*s]	Height [mAU]	Area %
1	16.713	BB	0.8805	469.12994	7.23517	10.2373
2	23.490	BB	1.2037	4113.43213	50.21319	89.7627

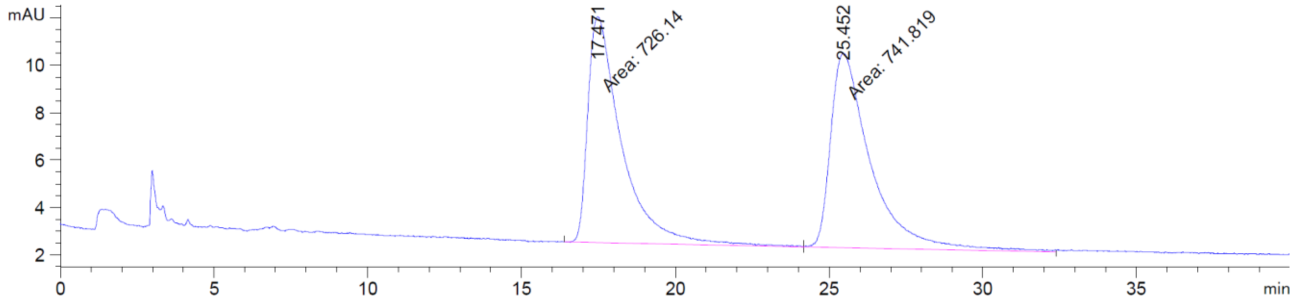

Peak #	RetTime [min]	Type	Width [min]	Area [mAU*s]	Height [mAU]	Area %
1	17.471	MF	1.2689	726.13971	9.53755	49.4659
2	25.452	FM	1.5092	741.81903	8.19221	50.5341

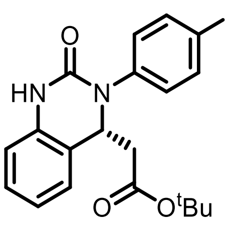

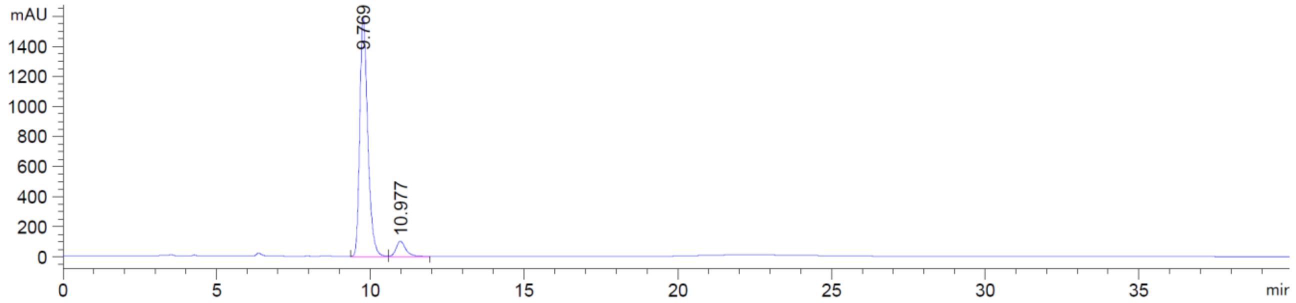

Peak #	RetTime [min]	Type	Width [min]	Area [mAU*s]	Height [mAU]	Area %
1	9.769	BV	0.2766	2.87444e4	1592.18542	93.0975
2	10.977	VB	0.3202	2131.19897	100.18283	6.9025

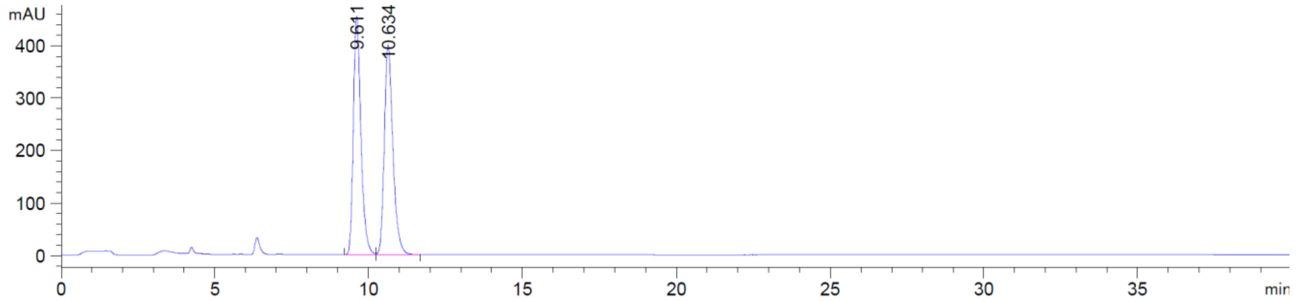

Peak #	RetTime [min]	Type	Width [min]	Area [mAU*s]	Height [mAU]	Area %
1	9.611	BV	0.2589	7750.69580	454.54404	49.9213
2	10.634	VB	0.2951	7775.12598	399.34534	50.0787

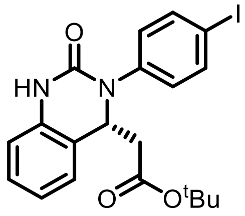

after recrystallization

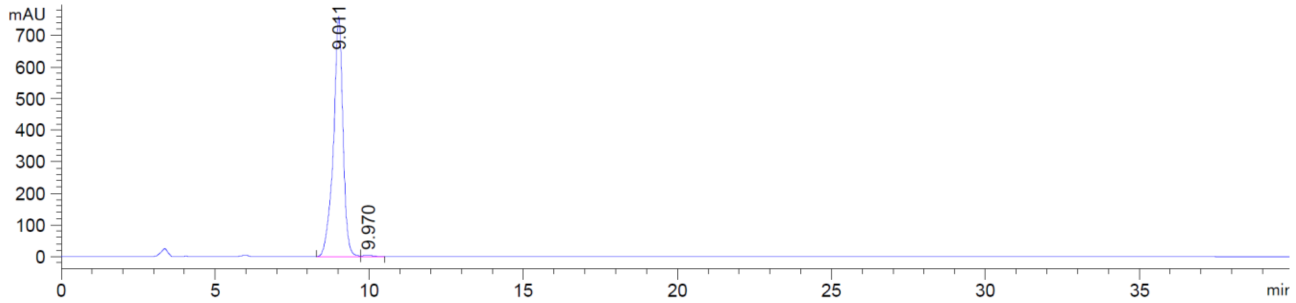

Peak #	RetTime [min]	Type	Width [min]	Area [mAU*s]	Height [mAU]	Area %
1	9.011	BV	0.3158	1.63940e4	759.62506	99.4602
2	9.970	VB	0.3496	88.96754	3.73962	0.5398

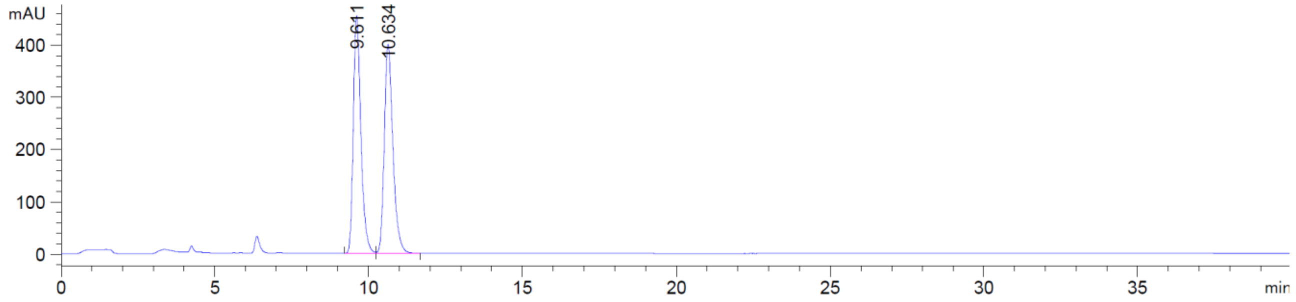

Peak #	RetTime [min]	Type	Width [min]	Area [mAU*s]	Height [mAU]	Area %
1	9.611	BV	0.2589	7750.69580	454.54404	49.9213
2	10.634	VB	0.2951	7775.12598	399.34534	50.0787

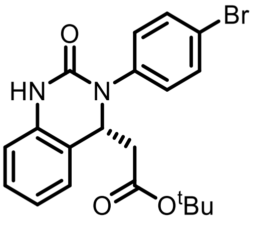

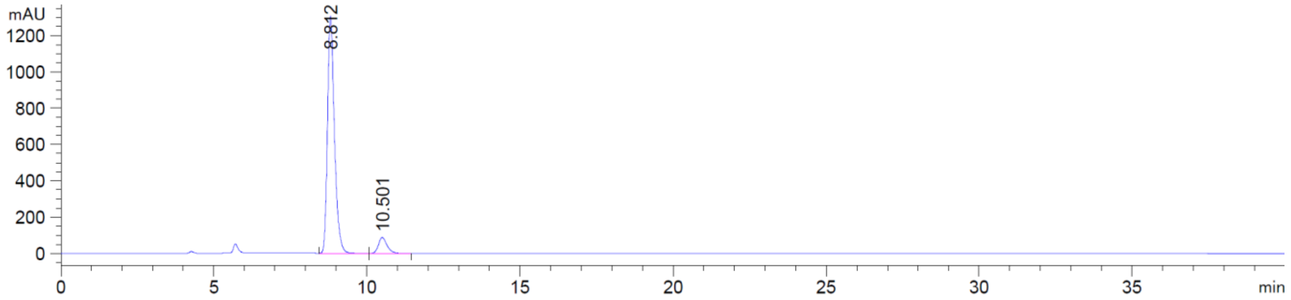

Peak #	RetTime [min]	Type	Width [min]	Area [mAU*s]	Height [mAU]	Area %
1	8.812	VV	0.2427	2.08980e4	1305.75854	92.2020
2	10.501	VB	0.3013	1767.44800	88.40229	7.7980

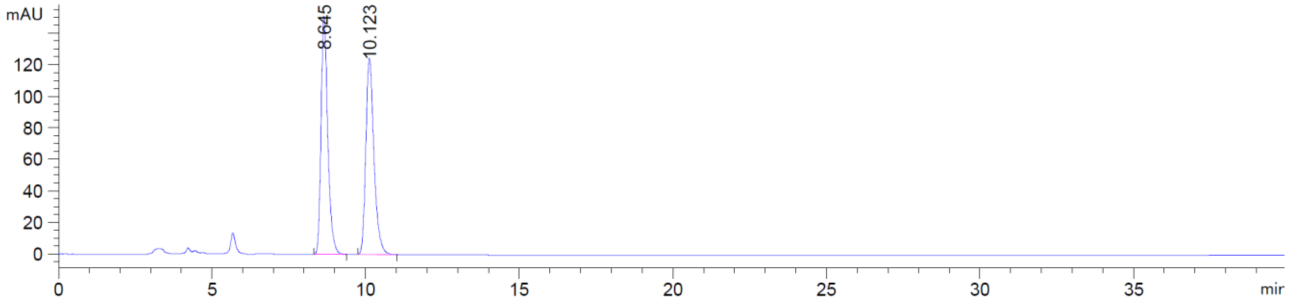

Peak #	RetTime [min]	Type	Width [min]	Area [mAU*s]	Height [mAU]	Area %
1	8.645	BB	0.2301	2307.94312	151.17084	50.0346
2	10.123	BB	0.2818	2304.74707	124.58217	49.9654

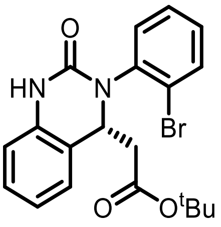

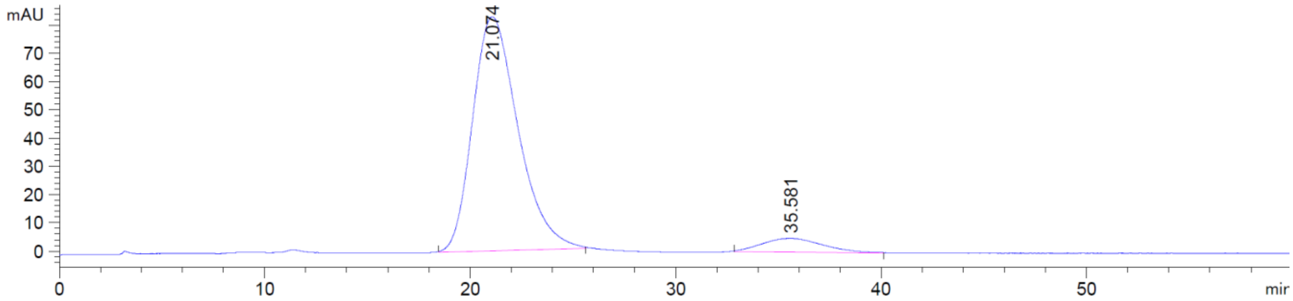

Peak #	RetTime [min]	Type	Width [min]	Area [mAU*s]	Height [mAU]	Area %
1	21.074	BB	2.2473	1.27697e4	82.98274	92.9111
2	35.581	BB	2.3866	974.29553	4.81106	7.0889

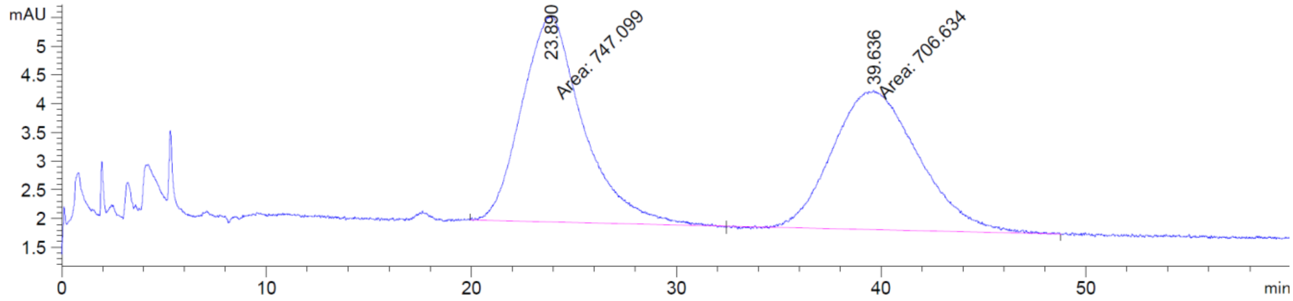

Peak #	RetTime [min]	Type	Width [min]	Area [mAU*s]	Height [mAU]	Area %
1	23.890	MF	3.4726	747.09888	3.58564	51.3918
2	39.636	FM	4.8528	706.63373	2.42691	48.6082

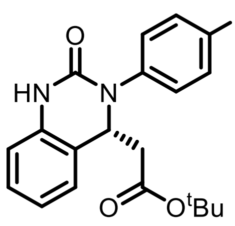

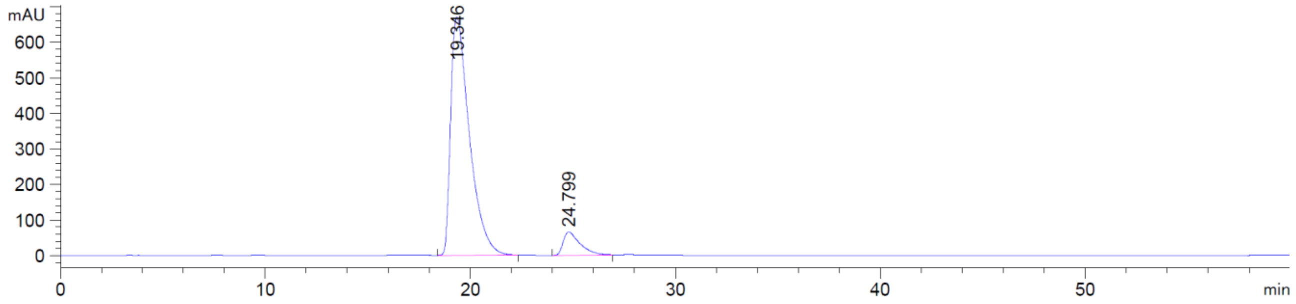

Peak #	RetTime [min]	Type	Width [min]	Area [mAU*s]	Height [mAU]	Area %
1	19.346	BB	0.9577	4.23203e4	669.26642	91.9220
2	24.799	BB	0.8215	3719.04590	65.58806	8.0780

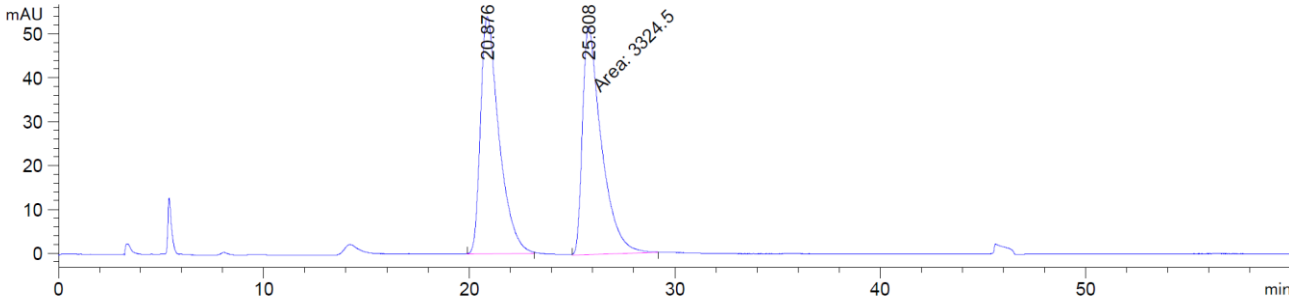

Peak #	RetTime [min]	Type	Width [min]	Area [mAU*s]	Height [mAU]	Area %
1	20.876	BB	0.9267	3388.51294	54.10376	50.4768
2	25.808	MM	1.0668	3324.50122	51.93906	49.5232

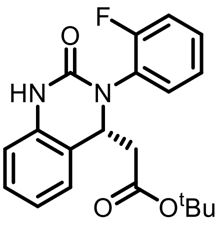

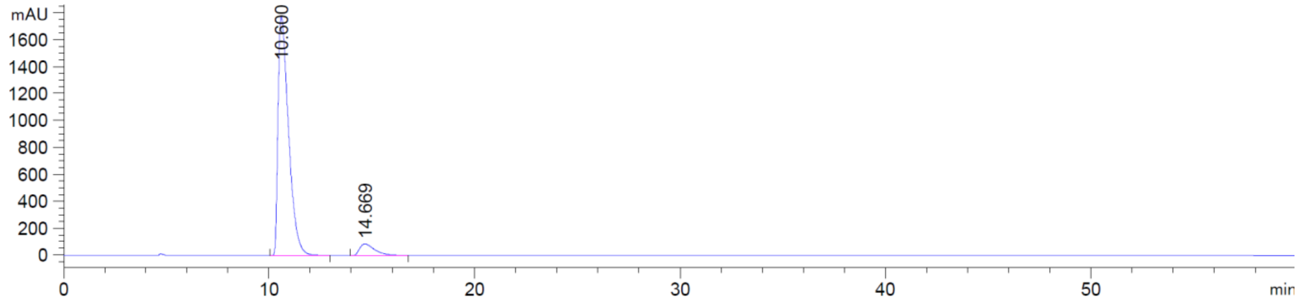

Peak #	RetTime [min]	Type	Width [min]	Area [mAU*s]	Height [mAU]	Area %
1	10.600	BB	0.5469	6.31180e4	1774.65161	93.6058
2	14.669	BB	0.7817	4311.56348	84.71931	6.3942

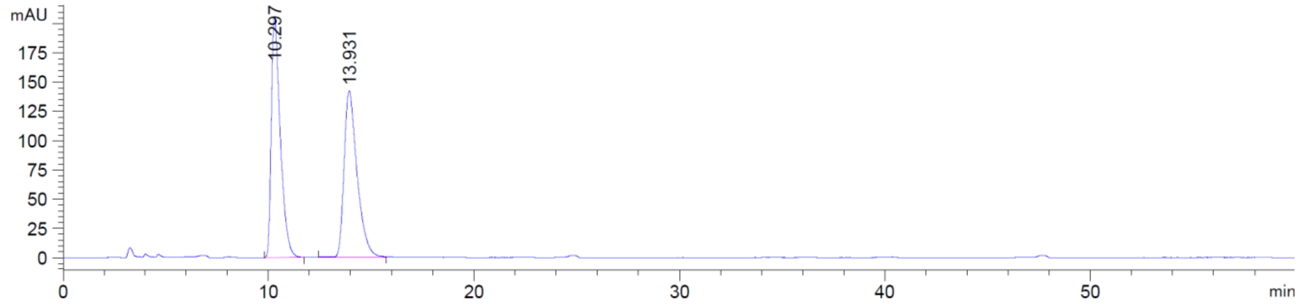

Peak #	RetTime [min]	Type	Width [min]	Area [mAU*s]	Height [mAU]	Area %
1	10.297	BB	0.4520	6238.77588	205.58563	49.9348
2	13.931	BB	0.6566	6255.06934	142.36607	50.0652

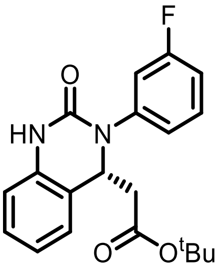

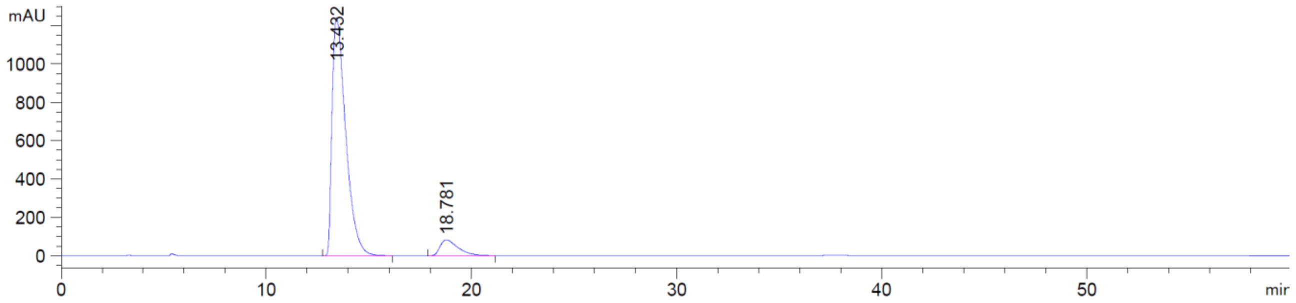

Peak #	RetTime [min]	Type	Width [min]	Area [mAU*s]	Height [mAU]	Area %
1	13.432	BB	0.7070	5.74316e4	1243.06140	91.8390
2	18.781	BB	0.9069	5103.48633	83.28439	8.1610

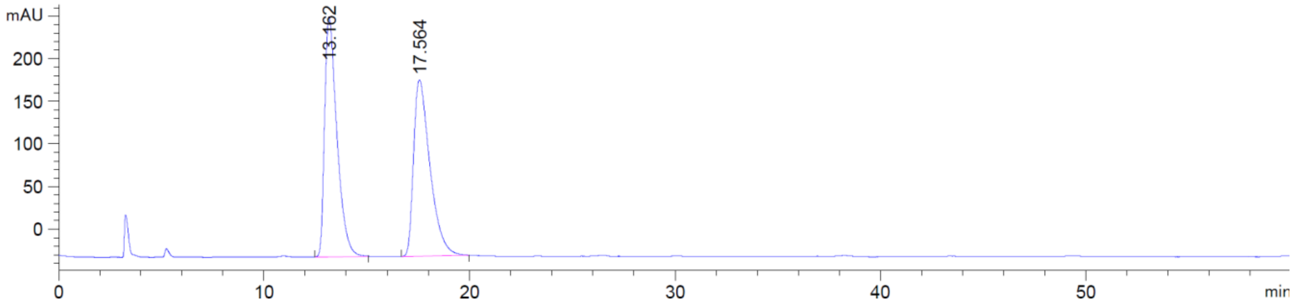

Peak #	RetTime [min]	Type	Width [min]	Area [mAU*s]	Height [mAU]	Area %
1	13.162	BB	0.6328	1.17105e4	281.78696	50.4272
2	17.564	BB	0.8243	1.15121e4	207.17584	49.5728

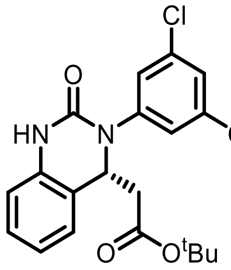

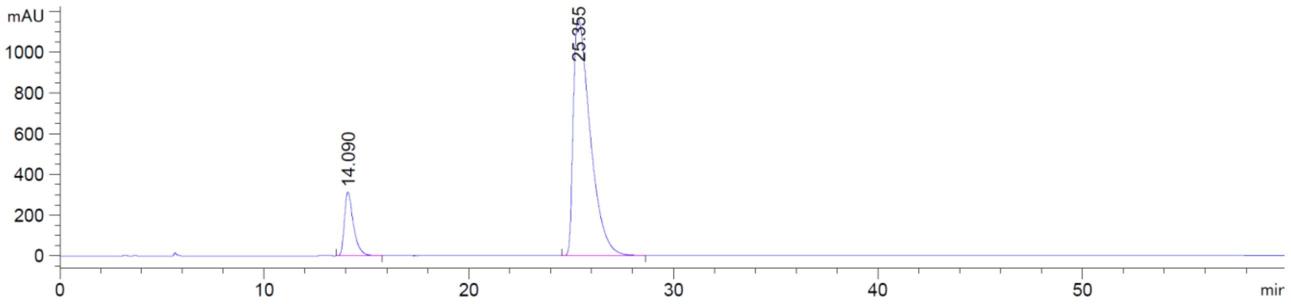

Peak #	RetTime [min]	Type	Width [min]	Area [mAU*s]	Height [mAU]	Area %
1	14.090	BB	0.4602	9547.57031	312.67651	12.4475
2	25.355	BB	0.8677	6.71549e4	1166.46252	87.5525

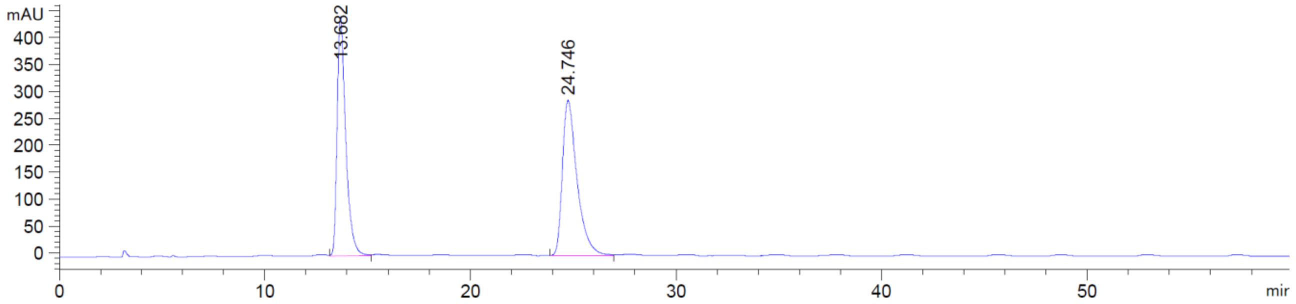

Peak #	RetTime [min]	Type	Width [min]	Area [mAU*s]	Height [mAU]	Area %
1	13.682	VB	0.4378	1.29356e4	444.17398	47.4970
2	24.746	BB	0.7421	1.42989e4	288.57858	52.5030

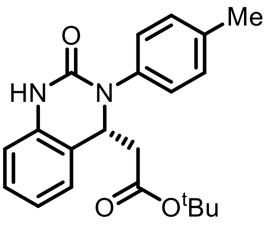

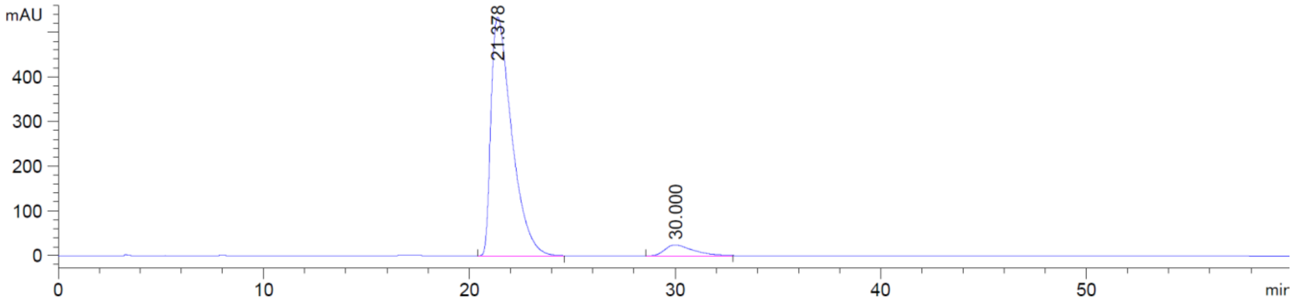

Peak #	RetTime [min]	Type	Width [min]	Area [mAU*s]	Height [mAU]	Area %
1	21.378	BB	1.0581	3.74870e4	534.59631	94.1499
2	30.000	BB	1.3730	2329.30273	24.32932	5.8501

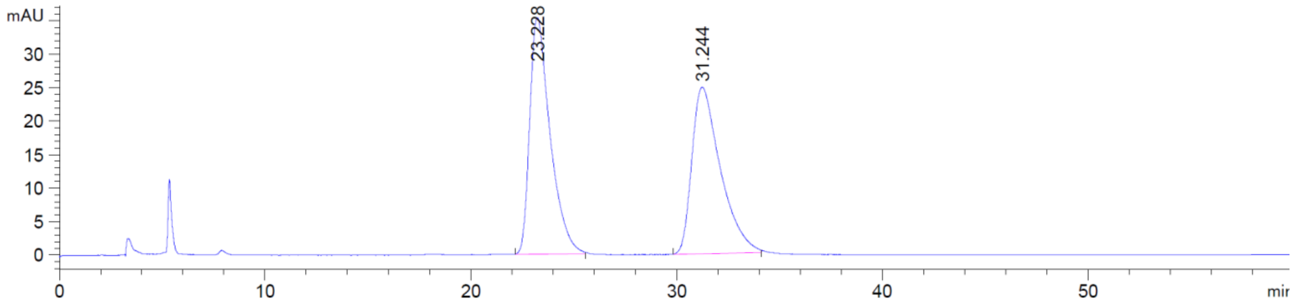

Peak #	RetTime [min]	Type	Width [min]	Area [mAU*s]	Height [mAU]	Area %
1	23.228	BB	1.0025	2443.31738	35.34394	50.4834
2	31.244	BB	1.3724	2396.52856	24.86551	49.5166

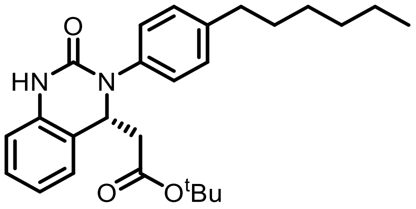

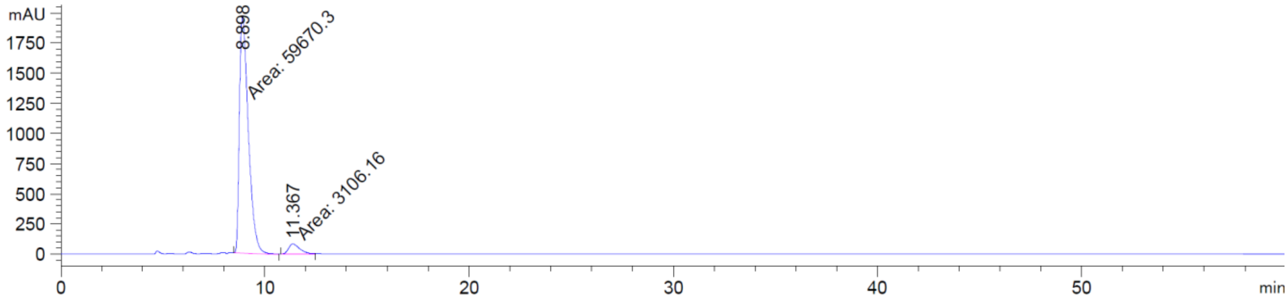

Peak #	RetTime [min]	Type	Width [min]	Area [mAU*s]	Height [mAU]	Area %
1	8.898	MM	0.5061	5.96703e4	1964.96448	95.0520
2	11.367	MM	0.6271	3106.16455	82.55730	4.9480

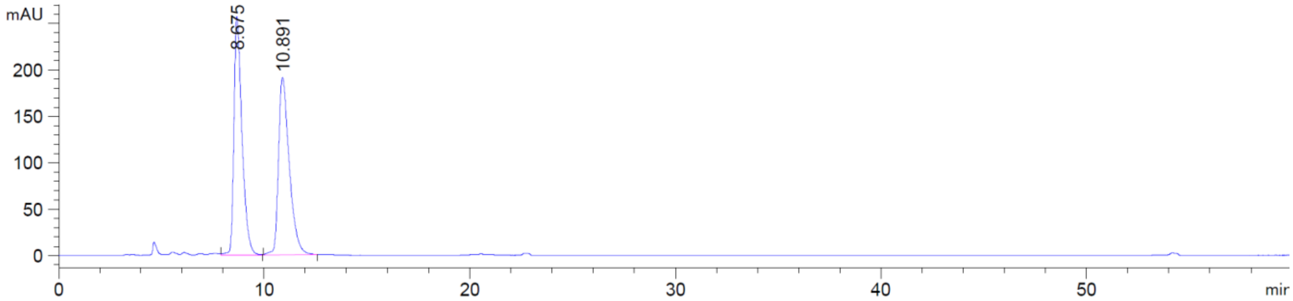

Peak #	RetTime [min]	Type	Width [min]	Area [mAU*s]	Height [mAU]	Area %
1	8.675	VB	0.4063	7012.70947	257.05014	50.1746
2	10.891	BB	0.5495	6963.89600	190.92657	49.8254

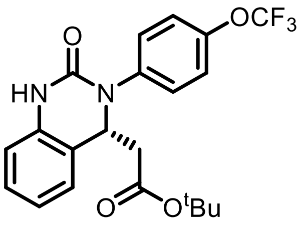

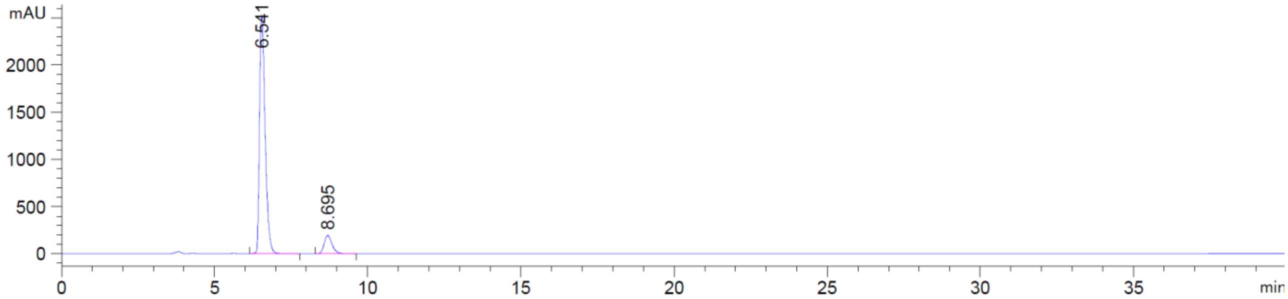

Peak #	RetTime [min]	Type	Width [min]	Area [mAU*s]	Height [mAU]	Area %
1	6.541	BB	0.1972	3.23493e4	2529.05420	90.2075
2	8.695	VB	0.2774	3511.70020	193.82027	9.7925

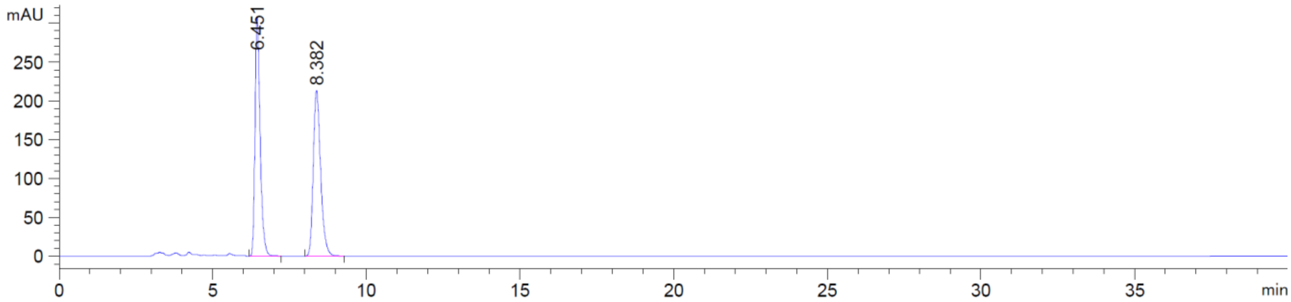

Peak #	RetTime [min]	Type	Width [min]	Area [mAU*s]	Height [mAU]	Area %
1	6.451	VB	0.1747	3559.28516	308.34982	50.0880
2	8.382	BB	0.2545	3546.77881	212.73648	49.9120

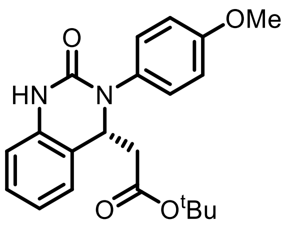

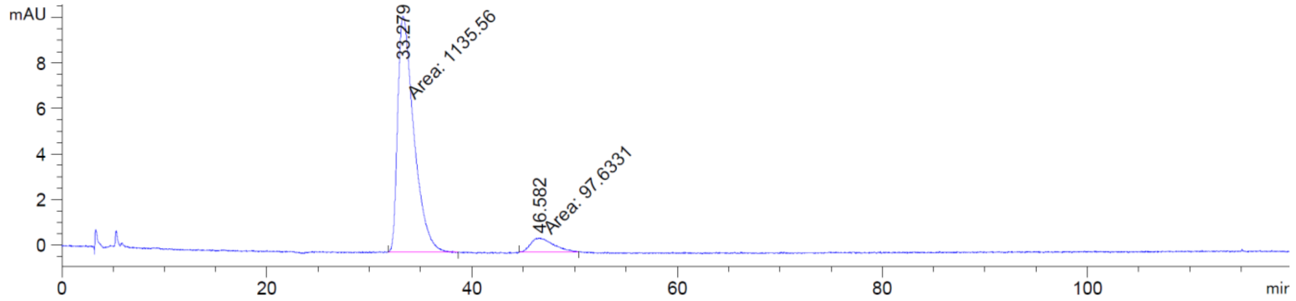

Peak #	RetTime [min]	Type	Width [min]	Area [mAU*s]	Height [mAU]	Area %
1	33.279	MM	1.8264	1135.56433	10.36228	92.0829
2	46.582	MM	2.5994	97.63313	6.26000e-1	7.9171

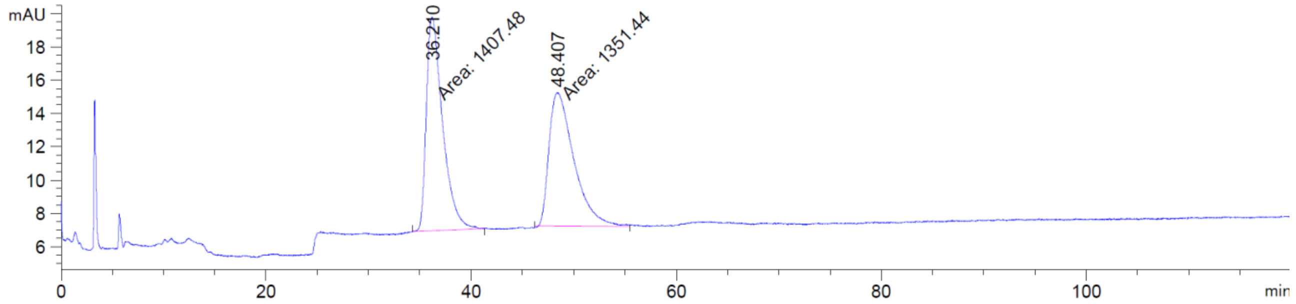

Peak #	RetTime [min]	Type	Width [min]	Area [mAU*s]	Height [mAU]	Area %
1	36.210	MM	1.8298	1407.47949	12.82015	51.0155
2	48.407	MM	2.8081	1351.44409	8.02120	48.9845

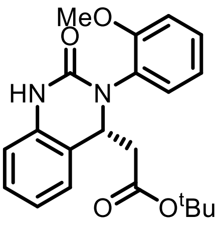

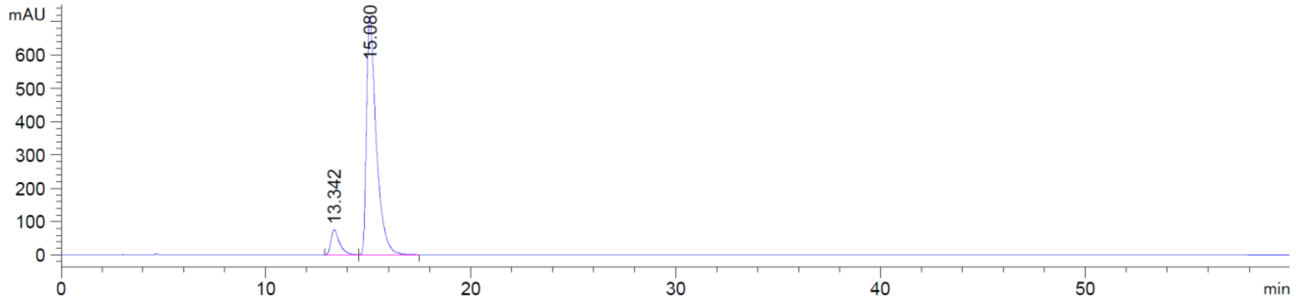

Peak #	RetTime [min]	Type	Width [min]	Area [mAU*s]	Height [mAU]	Area %
1	13.342	BV	0.4342	2228.58691	75.97629	8.5972
2	15.080	VB	0.4904	2.36936e4	715.29602	91.4028

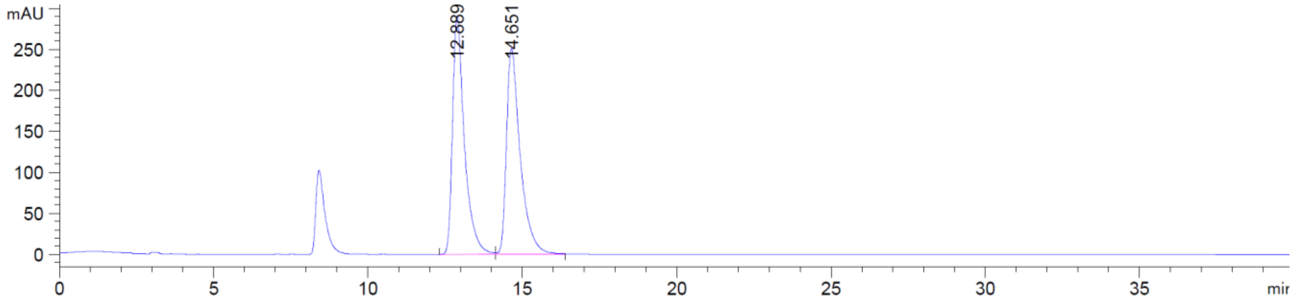

Peak #	RetTime [min]	Type	Width [min]	Area [mAU*s]	Height [mAU]	Area %
1	12.889	BV	0.3967	7801.11719	291.02603	50.0142
2	14.651	VB	0.4585	7796.69141	250.90901	49.9858

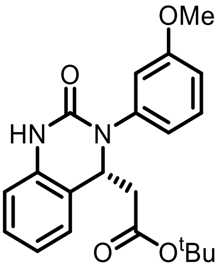

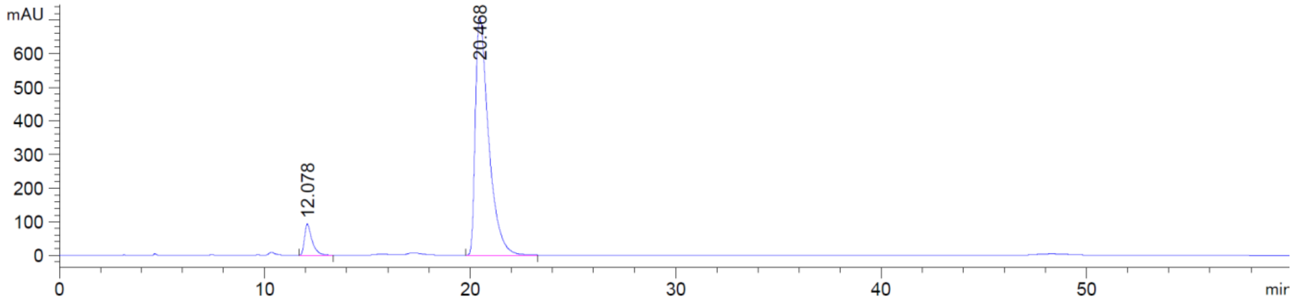

Peak #	RetTime [min]	Type	Width [min]	Area [mAU*s]	Height [mAU]	Area %
1	12.078	BB	0.3774	2376.81055	93.23150	6.9425
2	20.468	BB	0.6670	3.18590e4	710.67029	93.0575

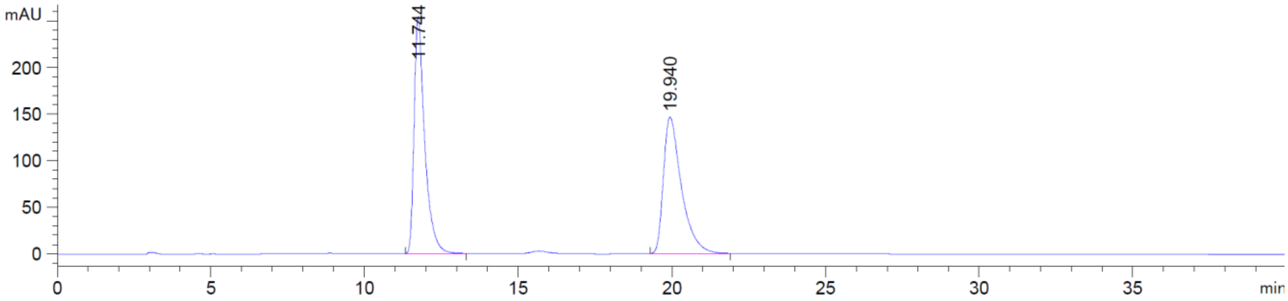

Peak #	RetTime [min]	Type	Width [min]	Area [mAU*s]	Height [mAU]	Area %
1	11.744	BB	0.3562	6113.49414	254.58945	50.4952
2	19.940	BB	0.6092	5993.57617	146.54384	49.5048

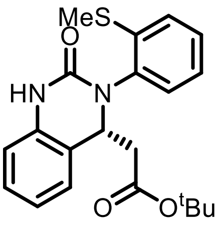

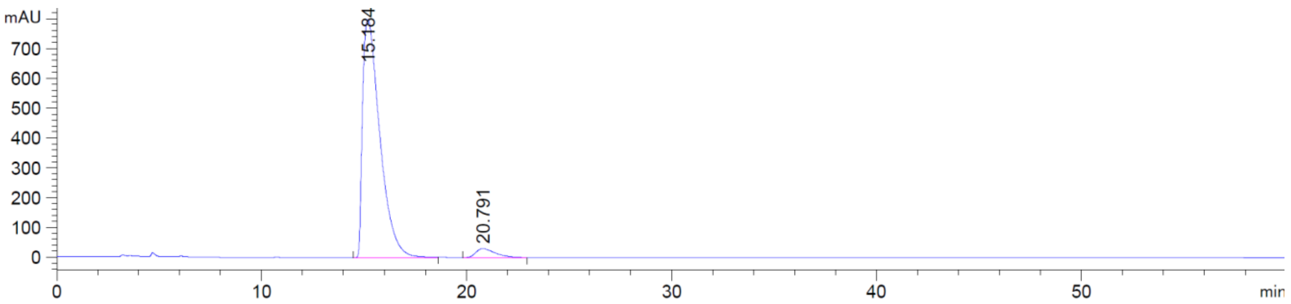

Peak #	RetTime [min]	Type	Width [min]	Area [mAU*s]	Height [mAU]	Area %
1	15.184	BB	0.8896	4.67151e4	799.55908	95.9239
2	20.791	BB	0.9846	1985.07568	29.97701	4.0761

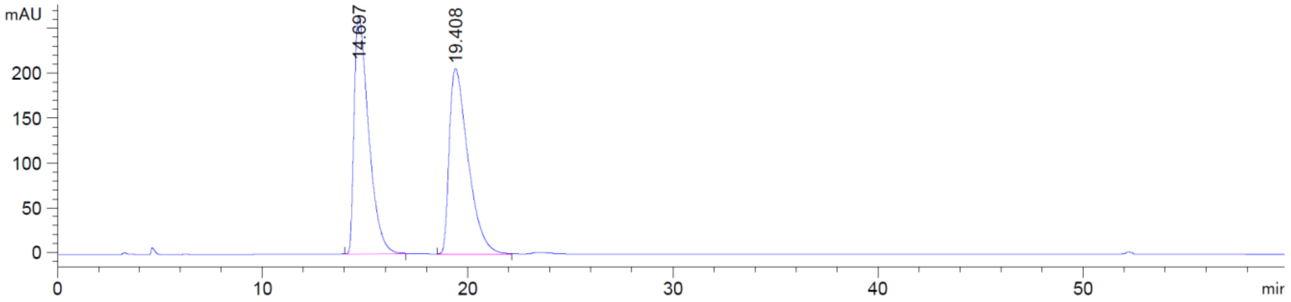

Peak #	RetTime [min]	Type	Width [min]	Area [mAU*s]	Height [mAU]	Area %
1	14.697	BB	0.7555	1.30692e4	264.93997	49.8890
2	19.408	BB	0.9609	1.31274e4	206.71907	50.1110

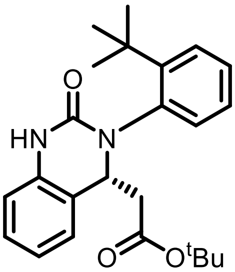

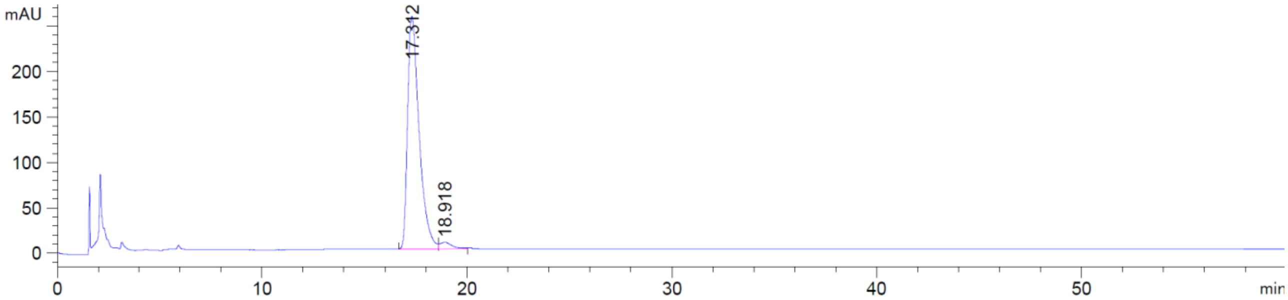

Peak #	RetTime [min]	Type	Width [min]	Area [mAU*s]	Height [mAU]	Area %
1	17.312	BV	0.5917	1.00036e4	256.08664	97.1072
2	18.918	VB	0.5822	298.00882	7.27107	2.8928

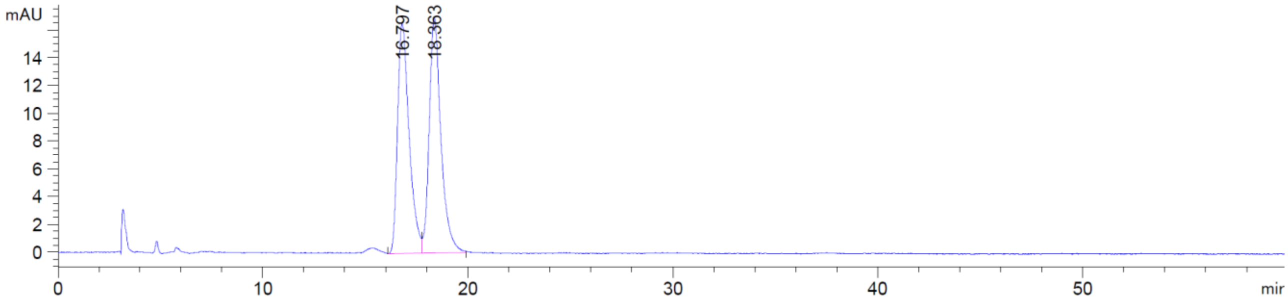

Peak #	RetTime [min]	Type	Width [min]	Area [mAU*s]	Height [mAU]	Area %
1	16.797	VV	0.5904	650.16974	16.54677	48.9865
2	18.363	VB	0.5841	677.07428	17.01893	51.0135

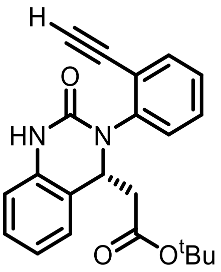

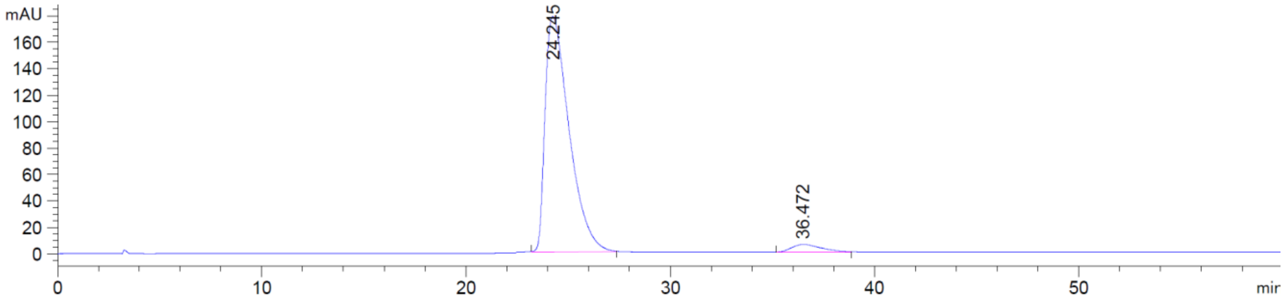

Peak #	RetTime [min]	Type	Width [min]	Area [mAU*s]	Height [mAU]	Area %
1	24.245	BB	1.2403	1.45455e4	178.20059	96.0026
2	36.472	BB	1.2201	605.64490	5.88787	3.9974

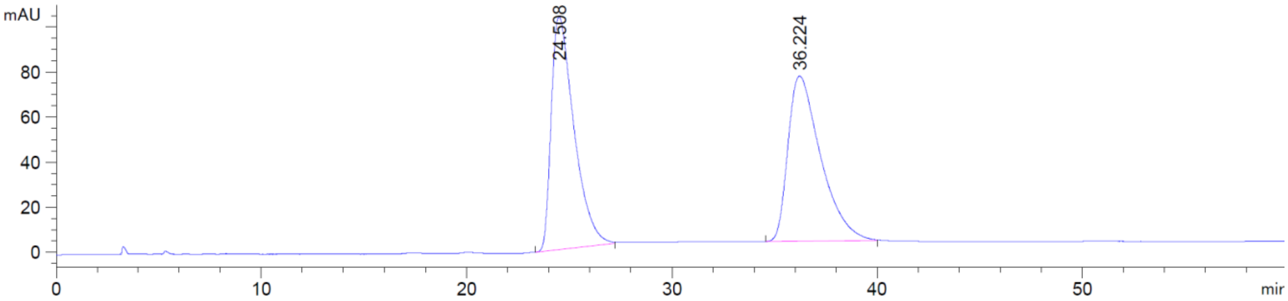

Peak #	RetTime [min]	Type	Width [min]	Area [mAU*s]	Height [mAU]	Area %
1	24.508	BB	1.1684	7975.52930	103.33752	49.5196
2	36.224	BB	1.6173	8130.27979	73.34376	50.4804

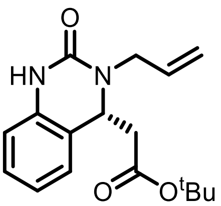

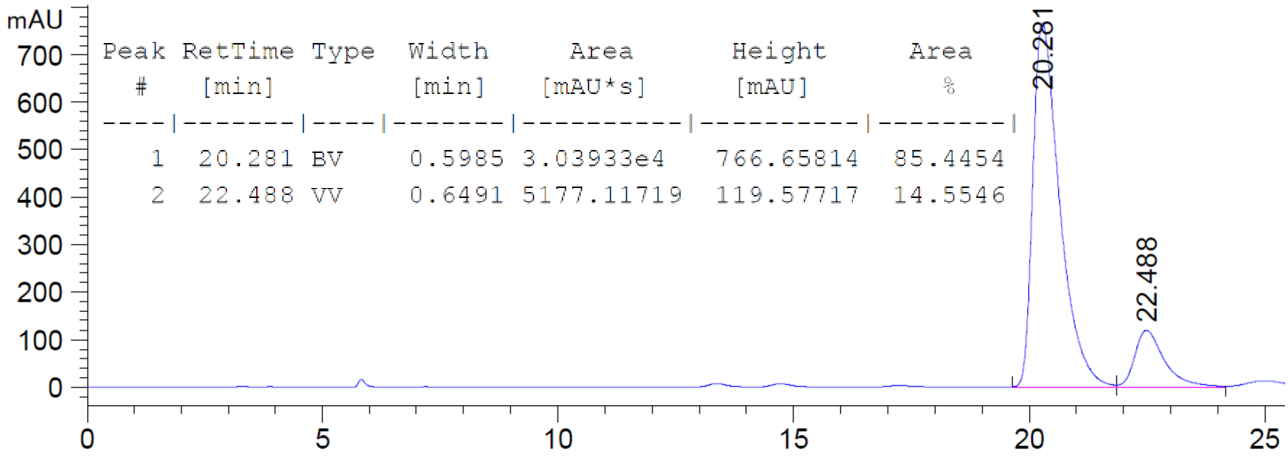

Peak #	RetTime [min]	Type	Width [min]	Area [mAU*s]	Height [mAU]	Area %
1	20.281	BV	0.5985	3.03933e4	766.65814	85.4454
2	22.488	VV	0.6491	5177.11719	119.57717	14.5546

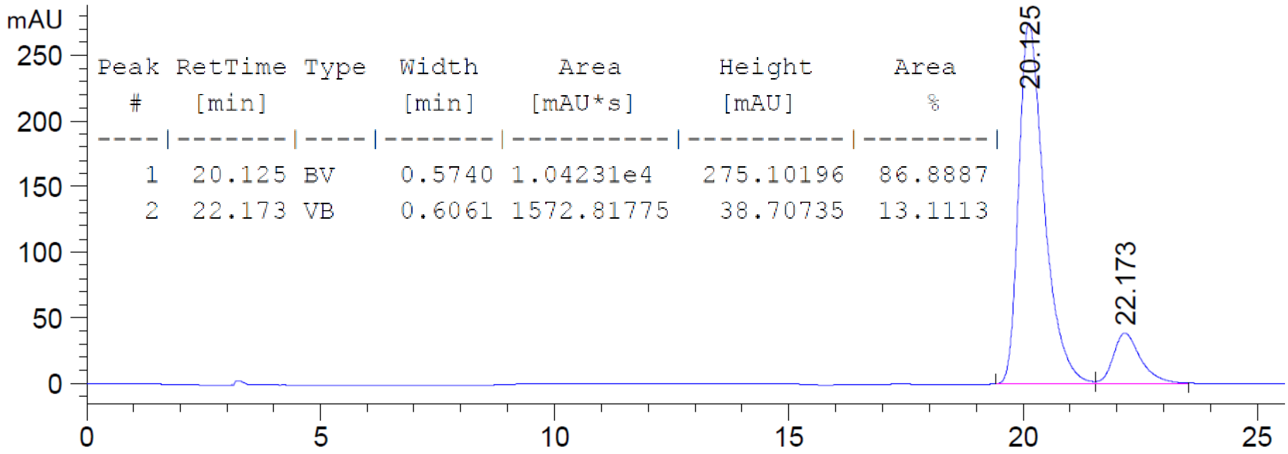

Peak #	RetTime [min]	Type	Width [min]	Area [mAU*s]	Height [mAU]	Area %
1	20.125	BV	0.5740	1.04231e4	275.10196	86.8887
2	22.173	VB	0.6061	1572.81775	38.70735	13.1113

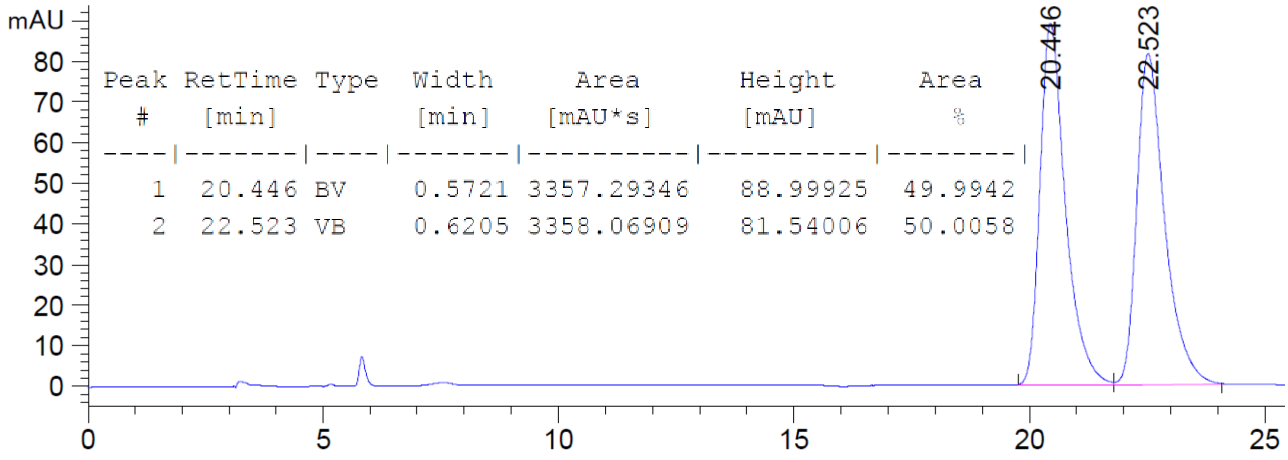

Peak #	RetTime [min]	Type	Width [min]	Area [mAU*s]	Height [mAU]	Area %
1	20.446	BV	0.5721	3357.29346	88.99925	49.9942
2	22.523	VB	0.6205	3358.06909	81.54006	50.0058

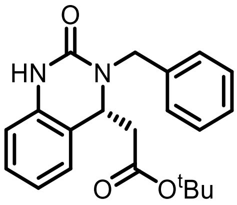

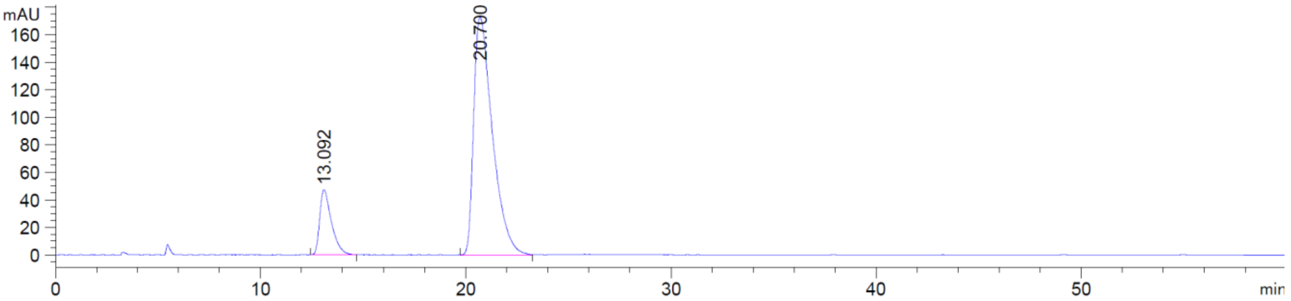

Peak #	RetTime [min]	Type	Width [min]	Area [mAU*s]	Height [mAU]	Area %
1	13.092	BB	0.5926	1884.81555	47.32831	14.6433
2	20.700	BB	0.9563	1.09867e4	173.14452	85.3567

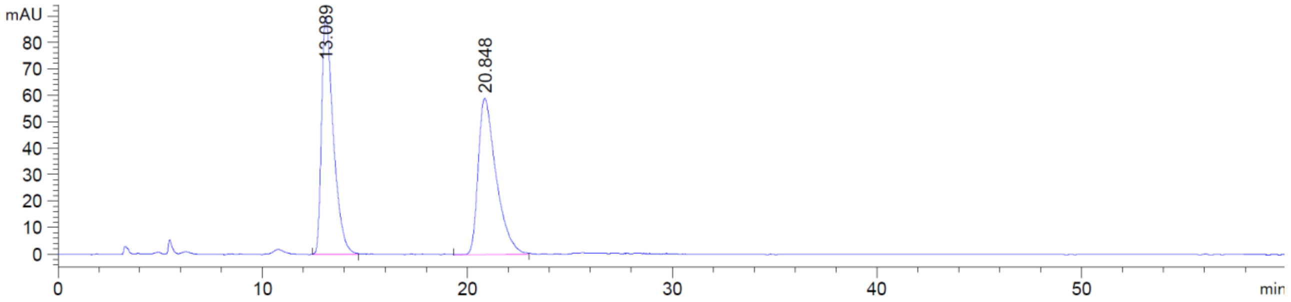

Peak #	RetTime [min]	Type	Width [min]	Area [mAU*s]	Height [mAU]	Area %
1	13.089	BB	0.5972	3581.64282	89.82197	49.7919
2	20.848	VB	0.9017	3611.57690	59.04163	50.2081

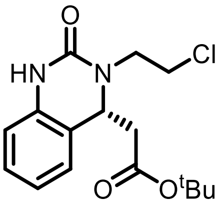

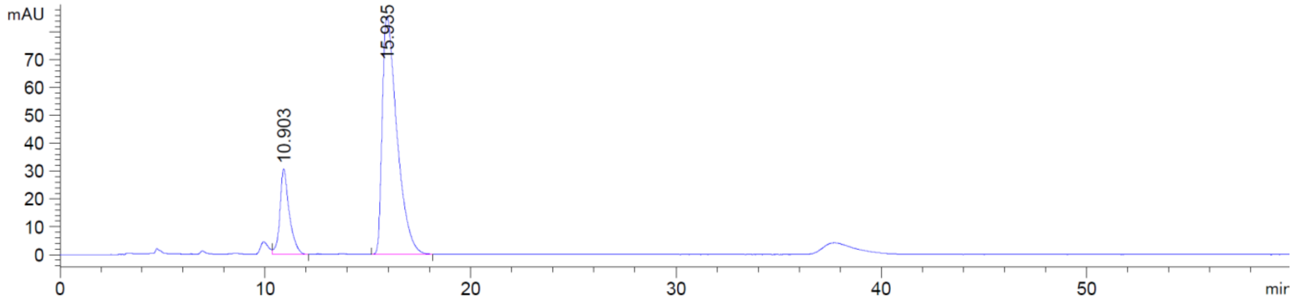

Peak #	RetTime [min]	Type	Width [min]	Area [mAU*s]	Height [mAU]	Area %
1	10.903	VB	0.4583	973.13495	30.65826	18.6126
2	15.935	BB	0.7528	4255.23047	85.47501	81.3874

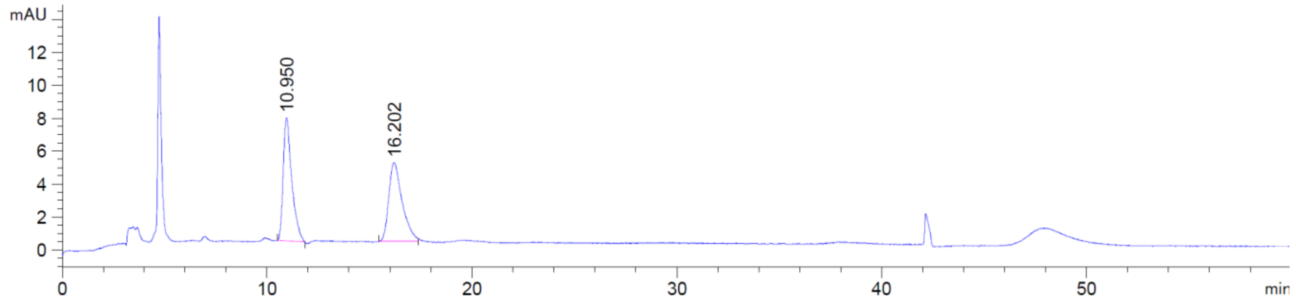

Peak #	RetTime [min]	Type	Width [min]	Area [mAU*s]	Height [mAU]	Area %
1	10.950	BB	0.4197	217.69588	7.47656	49.8538
2	16.202	BB	0.6603	218.97310	4.79977	50.1462

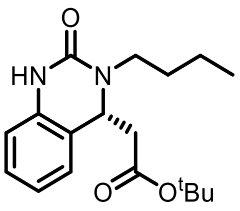

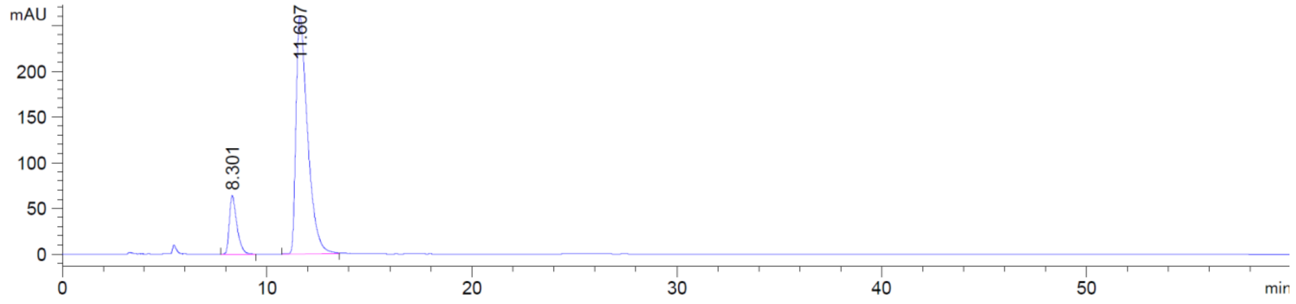

Peak #	RetTime [min]	Type	Width [min]	Area [mAU*s]	Height [mAU]	Area %
1	8.301	VB	0.3819	1644.17554	64.38108	13.8513
2	11.607	BB	0.5952	1.02261e4	259.81476	86.1487

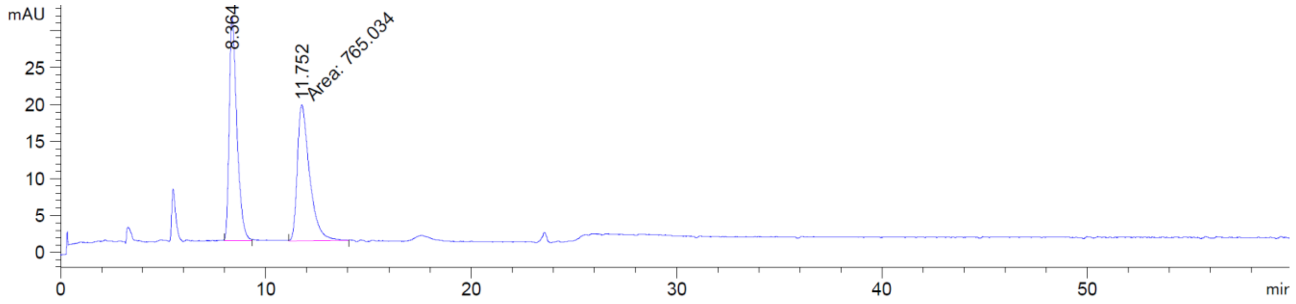

Peak #	RetTime [min]	Type	Width [min]	Area [mAU*s]	Height [mAU]	Area %
1	8.364	BB	0.3846	781.89349	30.34358	50.5449
2	11.752	MM	0.6938	765.03424	18.37742	49.4551

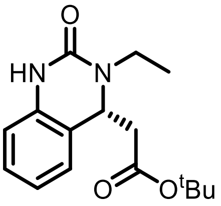

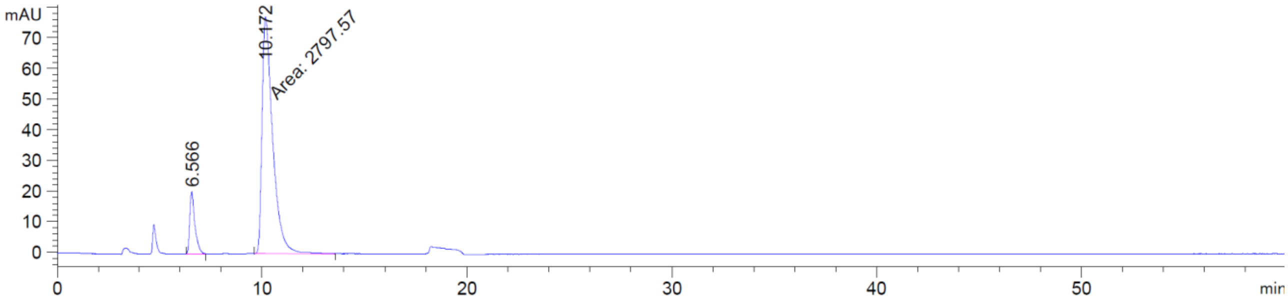

Peak #	RetTime [min]	Type	Width [min]	Area [mAU*s]	Height [mAU]	Area %
1	6.566	BB	0.2577	357.18250	20.26233	11.3220
2	10.172	MM	0.6028	2797.57495	77.35278	88.6780

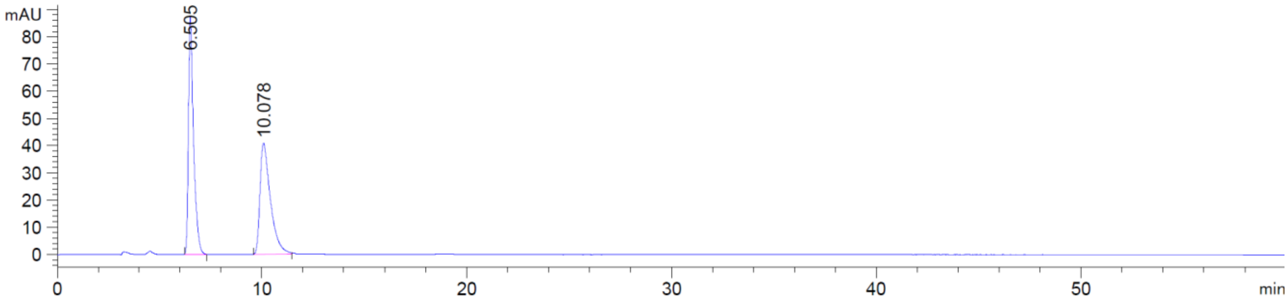

Peak #	RetTime [min]	Type	Width [min]	Area [mAU*s]	Height [mAU]	Area %
1	6.505	BB	0.2554	1521.97437	87.27882	51.2048
2	10.078	BB	0.5224	1450.35571	40.82749	48.7952

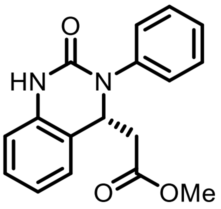

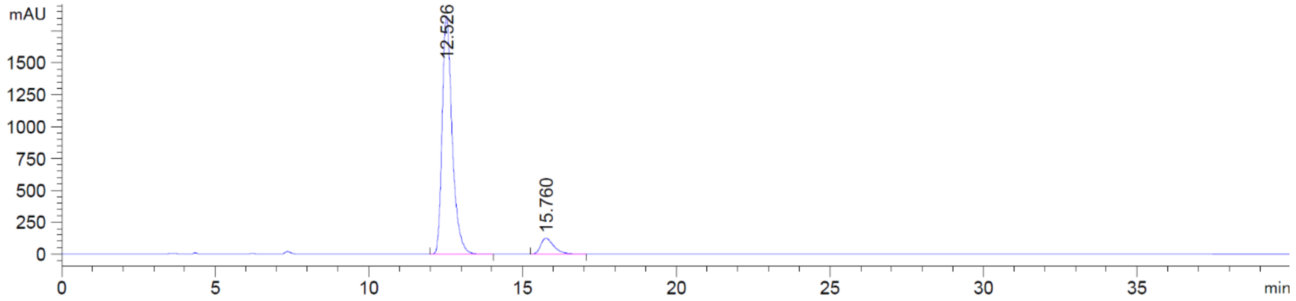

Peak #	RetTime [min]	Type	Width [min]	Area [mAU*s]	Height [mAU]	Area %
1	12.526	VB	0.3446	4.23217e4	1866.19653	91.8926
2	15.760	BB	0.4576	3733.91211	124.61517	8.1074

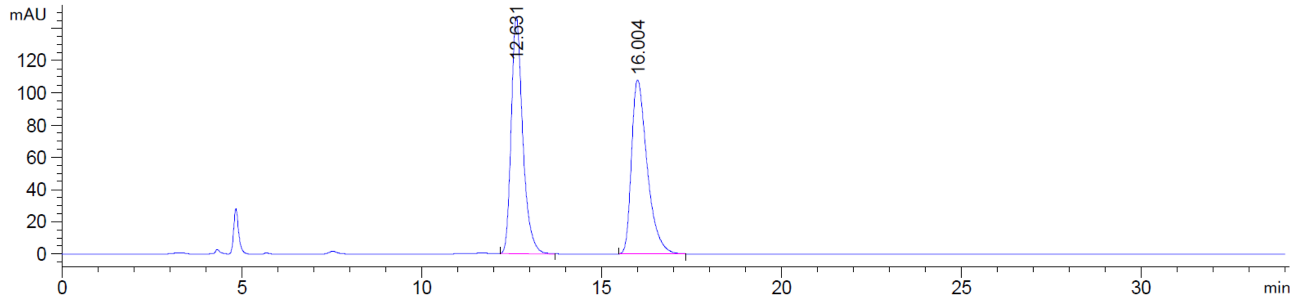

Peak #	RetTime [min]	Type	Width [min]	Area [mAU*s]	Height [mAU]	Area %
1	12.631	BB	0.3360	3279.47534	147.16277	49.9563
2	16.004	BB	0.4626	3285.21094	108.09774	50.0437

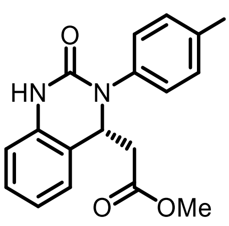

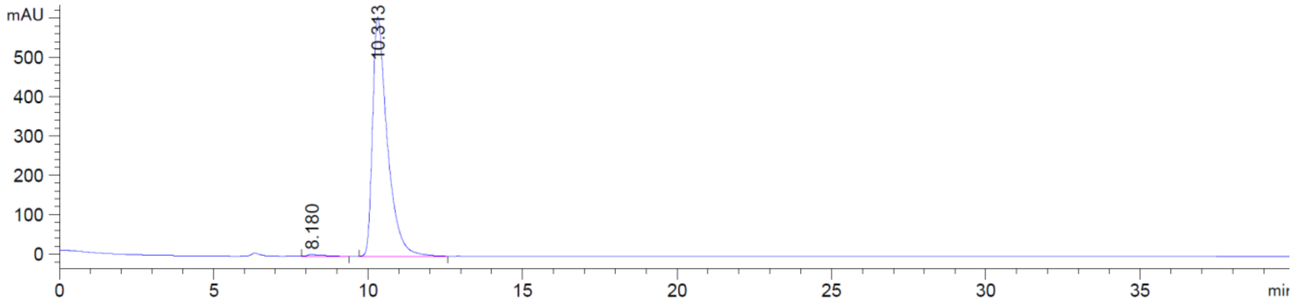

Peak #	RetTime [min]	Type	Width [min]	Area [mAU*s]	Height [mAU]	Area %
1	8.180	VB	0.4567	123.45078	3.74346	0.6078
2	10.313	BB	0.4870	2.01869e4	608.64038	99.3922

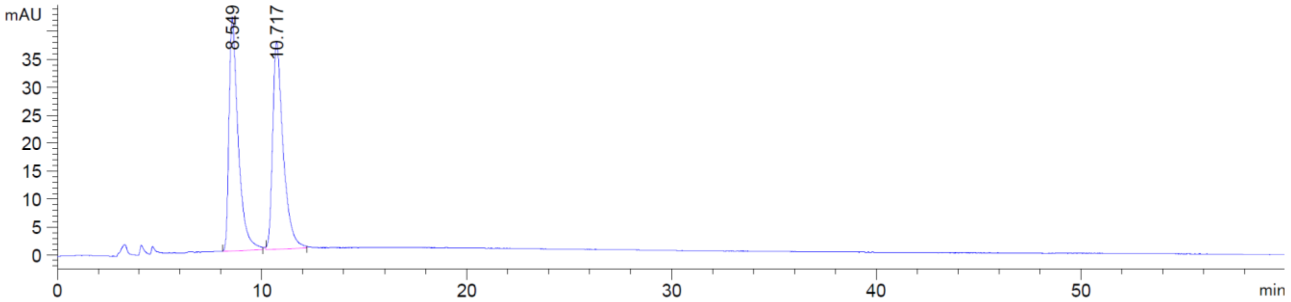

Peak #	RetTime [min]	Type	Width [min]	Area [mAU*s]	Height [mAU]	Area %
1	8.549	BB	0.4556	1305.73450	41.89917	49.7946
2	10.717	BB	0.5185	1316.50635	37.05433	50.2054

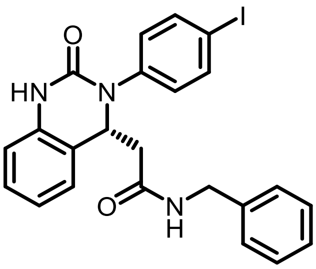

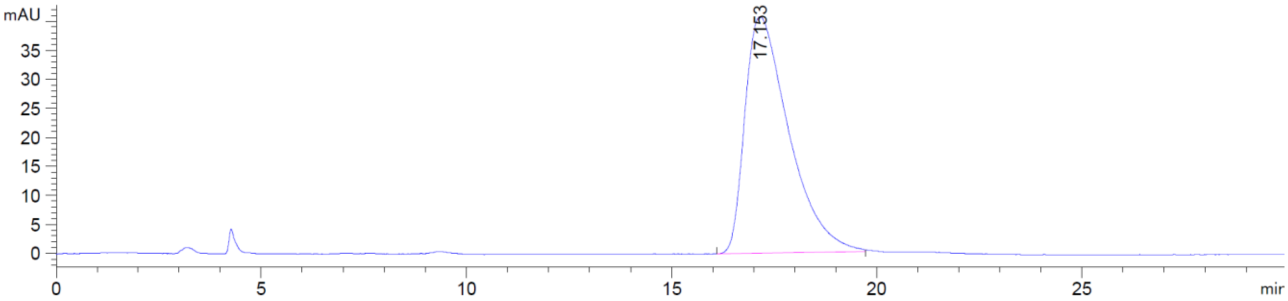

Peak #	RetTime [min]	Type	Width [min]	Area [mAU*s]	Height [mAU]	Area %
1	17.153	BB	1.0682	3050.33008	40.80371	100.0000

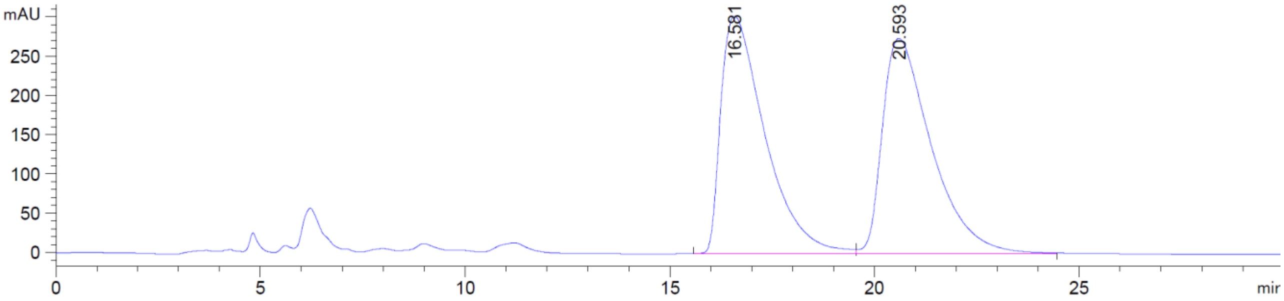

Peak #	RetTime [min]	Type	Width [min]	Area [mAU*s]	Height [mAU]	Area %
1	16.581	BV	1.1436	2.27109e4	302.59476	49.9511
2	20.593	VB	1.2449	2.27554e4	273.99057	50.0489

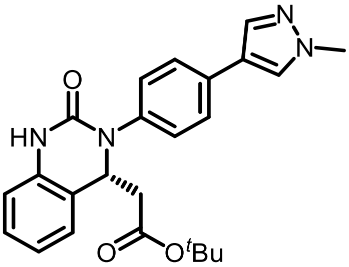

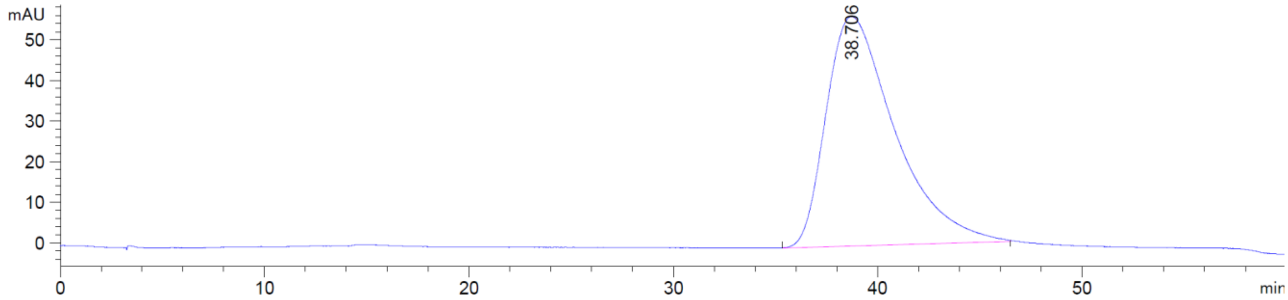

Peak #	RetTime [min]	Type	Width [min]	Area [mAU*s]	Height [mAU]	Area %
1	38.706	BB	2.8101	1.32387e4	56.49171	100.0000

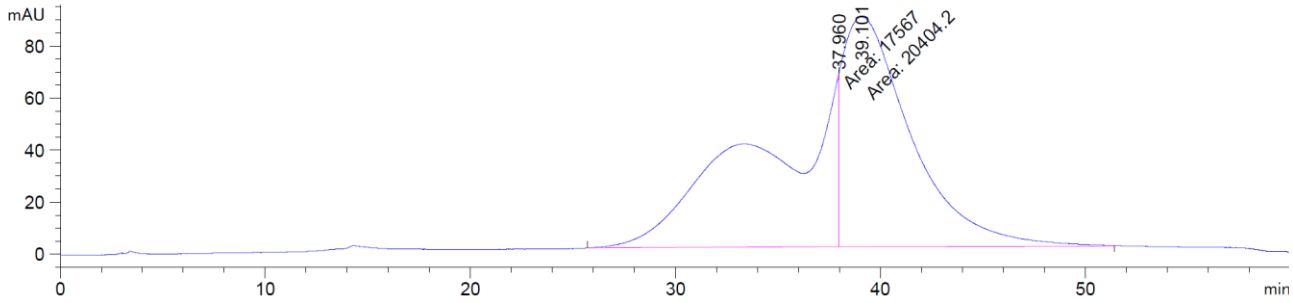

Peak #	RetTime [min]	Type	Width [min]	Area [mAU*s]	Height [mAU]	Area %
1	37.960	MF	4.4274	1.75670e4	66.13029	46.2640
2	39.101	FM	3.8464	2.04042e4	88.41191	53.7360

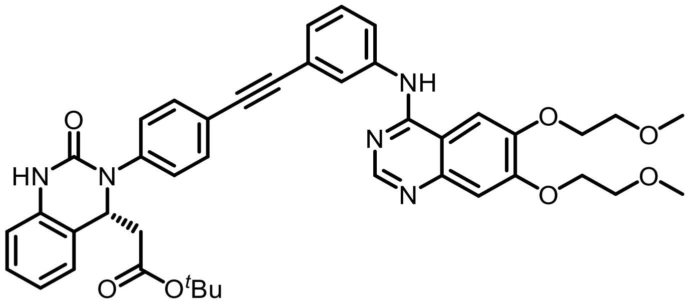

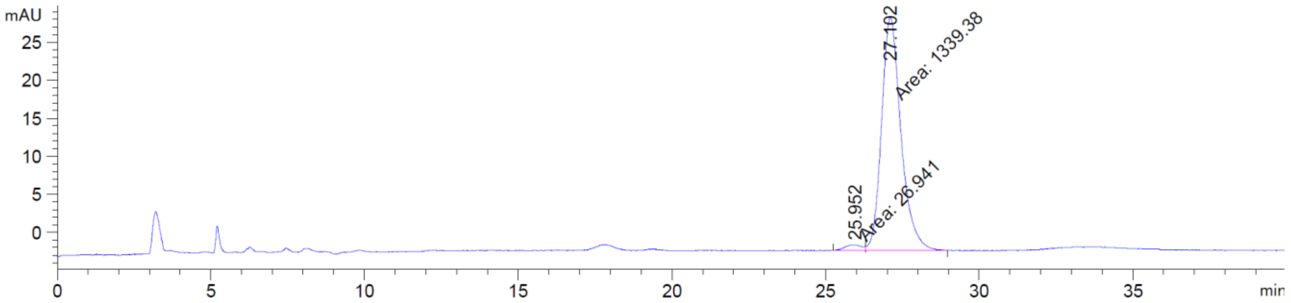

Peak #	RetTime [min]	Type	Width [min]	Area [mAU*s]	Height [mAU]	Area %
1	25.952	MF	0.6461	26.94096	6.94931e-1	1.9718
2	27.102	FM	0.7298	1339.38428	30.58879	98.0282

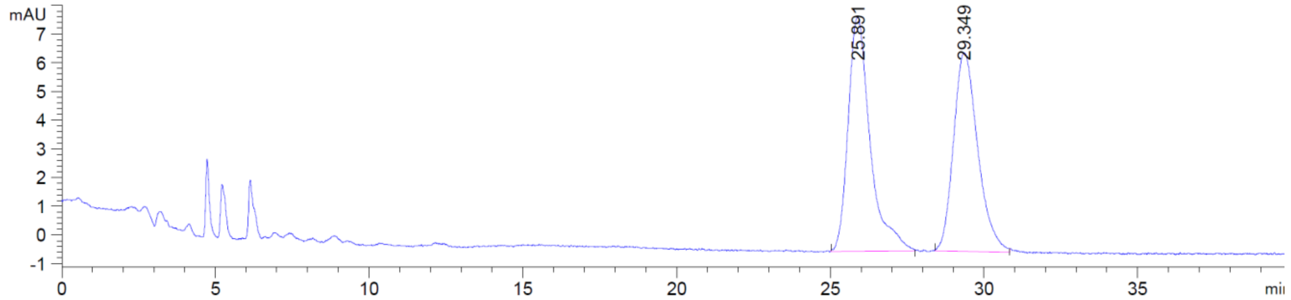

Peak #	RetTime [min]	Type	Width [min]	Area [mAU*s]	Height [mAU]	Area %
1	25.891	BB	0.7299	407.78116	8.17590	51.7861
2	29.349	BB	0.7716	379.65256	6.92469	48.2139

12. References

1. S. Ruppenthal, R. Brückner, *Eur. J. Org. Chem.,* **2018**, 2518.
2. S. Bera, G. Panda, *Org. Biomol. Chem.,* **2014,** *12,* 3976.
3. M. Lu, Q. Lu, J. F. Honek, *Bioorg. Med. Chem. Lett.,* **2017**, *27*, 282
4. D. M. Rotstein, S. D. Gabriel, N. Manser, L. Filonova, F. Padilla, S. Sankuratri, C. Ji, A. de Rosier, M. Dioszegi, G. Heilek, A. Jekle, P. Weller, P. Berry, *Bioorg. Med. Chem. Lett.,* **2010**, *20*, 3219
5. M. S. Manna, S. Mukherjee, *Chem. Sci.,* **2014**, *5*, 1627
6. M. M. Ahire, M. D. Pol, D. S. Kavale, R. G. Gonnade, S. B. Mhaske, *Org. Biomol. Chem.*, **2019**, *17*, 7135
7. T. Sakamoto, Y. Kondo and H. Yamanaka, *Chem. Pharm. Bull.,* **1985***, 11,* 4764
8. S. J. Park, S. J. Park, M. J. Lee, H. Rhima, Y. Kim, J. Lee, B. Y. Chung, J. Y. Lee, *Bioorg. Med. Chem.,* **2006**, *14*, 3502.
9. G. R. Humphrey, S. M. Dalby, T. Andreani, B. Xiang, M. R. Luzung, Z. J. Song, M. Shevlin, M. Christensen, K. M. Belyk, D. M. Tschaen, *Org. Process Res. Dev.,* **2016**, *20*, 1097
10. S. J. Park, M. J. Lee, H. Rhima, Y. Kim, J. Lee, B. Y. Chung, J. Y. Lee, *Bioorg. Med. Chem.,* **2006**, *14*, 3502.
11. P. Molina, E. Aller, A. Lorenzo, *Synthesis,* **1998**, *3*, 283.
12. J. Cosier and A. M. Glazer, *J. Appl. Cryst.,* **1986**, *19*, 105-107.
13. a) L. Palatinus and A. J. van der Lee, *Appl. Crystallogr.* **2008**, *41*, 975–984; b) L. Palatinus, *Acta Crystallogr. Sect. B Struct. Sci. Cryst. Eng. Mater.* **2013**, *69*, 1–16.
14. L. Palatinus, G. J. Chapuis, *Appl. Crystallogr.* **2007**, *40*, 786–790 .
15. a) P. Parois, R.I. Cooper and A.L. Thompson, *Chem. Cent. J.,* **2015**, 9:30; b) R. I. Cooper, A. L. Thompson and D. J. Watkin, *J. Appl. Cryst.* **2010**, *43*, 1100-1107.
16. a) T. Okino, Y. Hoashi, Y. Takemoto, *J. Am. Chem. Soc.* **2003**, *125*, 12672–12673; b) T. Okino, Y. Hoashi, T. Furukawa, X. Xu, Y. Takemoto, *J. Am. Chem. Soc.* **2005**, *127*, 119–125; c) A. Hamza, G. Schubert, T. Soós, I. Pápai, *J. Am. Chem. Soc.* **2006**, *128*, 13151–13160; d) B. Kótai, G. Kardos, A. Hamza, V. Farkas, I. Pápai, T. Soós, *Chem. Eur. J.* **2014**, *20*, 5631–5639.
